# Acupuncture for hormonal readiness and gut microbiota in obese polycystic ovary syndrome: an open-label, randomized controlled trial

**DOI:** 10.3389/fendo.2024.1509152

**Published:** 2024-12-19

**Authors:** Tianyu Wu, Guixing Xu, Xiaojuan Hong, Huaying Fan, Jiuzhi Zeng, Yu Liu, Jinqun Hu, Fanrong Liang, Jie Yang, Jiao Chen

**Affiliations:** ^1^ School of Acupuncture-Moxibustion and Tuina, Chengdu University of Traditional Chinese Medicine, Chengdu, Sichuan, China; ^2^ Affiliated Hospital of Chengdu University of Traditional Chinese Medicine (Sichuan Hospital of Traditional Chinese Medicine), Chengdu, Sichuan, China; ^3^ Sichuan Clinical Research Center of Acupuncture and Moxibustion, Chengdu University of Traditional Chinese Medicine, Chengdu, Sichuan, China; ^4^ Center of Reproductive Medicine, Sichuan Provincial Maternal and Child Health Hospital, Chengdu, Sichuan, China

**Keywords:** acupuncture, hormonal readiness, gut microbiota, obese polycystic ovary syndrome, randomized controlled trial

## Abstract

**Objective:**

To explore whether acupuncture combined with clomiphene can reduce the luteinizing hormone-to follicle-stimulating hormone ratio and impact the gut microbiota in patients with obese polycystic ovary syndrome.

**Methods:**

This open-label, randomized, parallel-group controlled trial included 86 women aged 20–40 years with obese polycystic ovary syndrome and 19 healthy controls. Participants were randomly assigned to either an acupuncture combined with clomiphene group or a clomiphene-only group, with a healthy control group for comparison. The treatment lasted three menstrual cycles, with acupuncture administered three times weekly and clomiphene given daily from day five of menstruation for five consecutive days per cycle. The primary outcome was the change in the luteinizing hormone-to-follicle-stimulating hormone (LH/FSH) ratio. Secondary outcomes included levels of other sex hormones, glucose and lipid metabolism parameters, self-rating anxiety and depression scale scores, and gut microbiota composition.

**Results:**

Intention-to-treat analysis showed that the adjusted mean luteinizing hormone-to follicle-stimulating hormone ratio decrease was -0.8 (95% CI: -1.14 to -0.46) in the acupuncture combined with clomiphene group and -0.22 (95% CI: -0.47 to 0.01) in the clomiphene group. The adjusted between-group difference was 0.53 (95% CI: 0.24 to 0.82, *p* < 0.001). The levels of Agathobacter faecis increased, and those of *Erysipelatoclostridium spiroforme*, *Streptococcus lutetiensis*, and *Lactococcus lactis* decreased after acupuncture combined with clomiphene treatment (*p* < 0.05).

**Conclusion:**

Acupuncture combined with clomiphene may be safe and effective, reduce the luteinizing hormone-to follicle-stimulating hormone ratio, and improve insulin resistance in obese polycystic ovary syndrome, and these outcomes may be related to the gut microbiota.

## 1 Introduction

Polycystic ovary syndrome (PCOS) is the most common endocrine disorder in women of reproductive age (1), characterized by phenotypic or biochemical hyperandrogenism, ovulatory dysfunction, and polycystic ovarian morphology on pelvic imaging (2, 3). PCOS affects up to one in seven women and accounts for over $8 billion in annual healthcare costs in the US (4, 5). In addition, approximately 50% of PCOS patients are obese or overweight (6), which can increase insulin resistance (IR) and compensatory hyperinsulinemia, increasing lipogenesis and decreasing lipolysis, creating a vicious cycle (7–11). Obesity sensitizes sheath cells to luteinizing hormone (LH) stimulation and amplifies functional ovarian hyperandrogenism by upregulating ovarian androgen production (12), and hyperandrogenism is considered a major clinical hallmark of PCOS (13).

Hyperandrogenism enhances the hypothalamic gonadotropin-releasing hormone, suppressing the pulse frequency of LH secretion by suppressing sex steroid-negative feedback, eventually leading to increased LH and androgen levels (12, 14, 15). A high ratio of LH relative to follicle-stimulating hormone (FSH) leads to insufficient follicular development, reduction of dominant follicles, and absence of mature follicles, leading to infertility.

Studies have found that PCOS and obesity are related to the intestinal flora. It is believed that changes in the composition of intestinal microorganisms lead to abnormal metabolites, causing inflammation in the intestines and ovaries, abnormal metabolism in the whole body, and obesity (16–18), which, in turn, affects the hormone levels (19, 20). Although clomiphene is used as a first-line ovulation-induction drug for infertile women with PCOS who desire to have children, 39.22% of women do not ovulate after taking clomiphene, and the live birth rate is only 7.84%, with some noticeable side effects (21). It is even more unfortunate that international and local health agencies and drug manufacturers have not shown interest in the treatment of PCOS (22), and the Food and Drug Administration and European Medicines Agency are yet to approve a drug specifically to treat PCOS (23, 24).

Consequently, an increasing number of women are seeking treatment for PCOS. As part of traditional Chinese medicine, acupuncture and moxibustion have been used clinically to treat reproduction-related diseases in China for a long time (25). Despite a large body of research on various forms of acupuncture, the clinical efficacy of acupuncture for PCOS remains controversial (26, 27). In particular, a high-quality randomized controlled trial (RCT) found that acupuncture did not improve the live birth rate in PCOS patients (28). However, considering that PCOS is usually accompanied by abnormal body weight and that this study carried out a stratified or subgroup analysis to take obesity factors into account, it is still unknown whether acupuncture has different curative effects on obese or overweight PCOS (OPCOS).

In addition, RCTs and meta-analyses have shown that acupuncture may reduce body weight and improve IR in obese patients (29). A systematic review reported that acupuncture has favorable effects on OPCOS based on low-quality evidence (30). However, previous curative effect evaluation methods were not sufficiently objective, and the clinical design and statistical methods were flawed, with inconclusive evidence for the efficacy of acupuncture in improving OPCOS hormone levels. In addition, gut microbes and hormone levels are thought to play key roles in the pathogenesis of OPCOS (17, 31), and acupuncture has been shown to modulate them (32).

Therefore, we hypothesized that acupuncture combined with clomiphene (AC) would be an effective treatment for patients with OPCOS and that acupuncture may improve the clinical symptoms of OPCOS by regulating the intestinal microbiota, thereby regulating the level of LH. Based on improved acupuncture methods, we conducted this multi-center RCT to obtain accurate conclusions about the effects of acupuncture using a high-quality clinical research design and considering more objective efficacy indicators and the possible mechanism of action of acupuncture.

## 2 Methods

### 2.1 Study design

This randomized, open-label, parallel-group controlled trial was conducted between February 2020 and December 2021 at the gynecological outpatient departments of Sichuan Women’s and Children’s Hospital, Sichuan Provincial Hospital of Traditional Chinese Medicine, the Third Affiliated Hospital of Chengdu University of Traditional Chinese Medicine, and Xinan Women’s and Children’s Hospital. This study aimed to compare AC with clomiphene for the treatment of OPCOS. The ethics review board of the Sichuan Traditional Chinese Medicine Regional Ethical Review Committee approved the study protocol (2019KL-075). The trial was registered with the Chinese Clinical Trial Registry (ChiCTR2000029882). All patients provided written informed consent before participation. The study adhered to the Consolidated Standards of Reporting Trials (33) and Standards for Reporting Interventions in Clinical Trials of Acupuncture guidelines (34) for reporting randomized trials. The trial protocol has been published (35).

### 2.2 Participants

From February 2020 to December 2021, participants with persistent OPCOS and healthy subjects were recruited by publishing recruitment advertisements in newspapers or on the information platform of the official website and bulletin boards of various research and recruitment websites by sending out leaflets with recruitment information and conducting free clinics in the community among other activities. Before participation, patients were informed about the study design, the advantages and disadvantages of the treatment, and relevant safety measures that would be used during the trial. All participants provided written informed consent before the start of the study. Physicians with over 3 years of clinical experience performed acupuncture in outpatient clinics. Healthy subjects did not undergo any preliminary treatment and only participated in intestinal microbiome testing. The inclusion and exclusion criteria for the patients and healthy controls are described in **Table 1**.

**Table 1:** The selection criteria for patients and healthy control subjects (HCs).

Patients with OPCOS
Inclusion criteria:
(1) had a diagnosis, at least two of the three symptoms of hypersecretion of androgen, ovulatory dysfunction (Olig ovulation or anovulation), and polycystic ovary morphology, based on ultrasound based on the Revised 2003 Consensus on Diagnostic Criteria and Long-Term Health Risks related to Polycystic Ovary Syndrome established by the Rotterdam ESHRE/ASRM-Sponsored PCOS Consensus Workshop Group35; (2) BMI ≥ 25 kg/m^2^; (3) were aged between 20 and 40 years; (4) suitable for administration of clomiphene as an ovulation induction treatment; (5) voluntarily provided written informed consent prior to randomization.
Exclusion criteria:
(1) with hyperandrogenism due to hyperprolactinemia, thyroid disease, congenital adrenal hyperplasia, or Cushing syndrome; (2) with genital tract malformation, gonadal dysgenesis, and fallopian tube blockage; (3) with pathological endometrial changes, such as uterine malformation and hysteromyoma; (4) with severe heart, liver, renal, pulmonary, hematological, or mental disease; (5) with any allergic condition; (6) patient has participating in other clinical trials; (7) with inflammatory bowel disease, irritable bowel syndrome, autoimmune diseases, chronic inflammatory diseases, or cancer; (8) with gastrointestinal surgery history; (9) with smoking history, alcohol use history; (10) with history of taking antibiotics or probiotics in the past 3 months. Note: Items from (1) to (6) are suitable for clinical observation. Items from (1) to (10) are suitable for metagenomic analysis.

HCs
Inclusion criteria:
(1) female aged 20 to 40 years; (2) BMI ≥ 18.5 kg/m^2^ and ≤ 25 kg/m^2^; (3) with regular menstruation; (4) no primary dysmenorrhea history in the past years; (5) normal blood/stool/urine test; (6) voluntarily provided written informed consent.
Exclusion criteria:
(1) with antibiotics taking history in the past 3 months; (2) with probiotics taking history within 1 month; (3) with smoking history and/or alcohol use; (4) with inflammatory bowel disease, irritable bowel syndrome, autoimmune diseases, cancer, or other diseases that may influence the gut microbiome; (5) with gastrointestinal surgery history; (6) with uncontrolled disease(s) that cause inflammation; (7) HCs is pregnant or lactating; (8) with mental disorders, cognitive disorders, or allergic conditions; (9) HCs has participated in other clinical trials.

### 2.3 Randomization and masking

OPCOS patients were randomly assigned (1:1) to either the AC or clomiphene alone. Independent statisticians (not researchers) used SPSS (version 27.0; IBM Corp., Armonk, NY, USA) to generate random sequences. Each random number was printed and placed in a separate lighttight envelope managed by a third party. When the participants met the established inclusion criteria for the study and voluntarily signed the informed consent form, the clinical research coordinator called a third party to obtain the subjects’ random numbers and grouping information. The acupuncturists then arranged the corresponding treatment plans for the participants. Owing to the nature of acupuncture and the control group used in this study, neither the patients nor practitioners could be blinded. At the same time, as the outcome indicators of this study were objective laboratory indicators, such as the LH-to-FSH ratio (LH/FSH), the outcome evaluators were not blinded. However, after the trial period, the statistician responsible for statistical analysis was blinded to the group assignment to prevent bias.

### 2.4 Intervention

All participants were informed of the benefits of healthy lifestyle habits such as regular exercise and healthy eating. The study group received AC citrate treatment, whereas the control group received only clomiphene citrate. Clomiphene citrate treatment will be administered on the fifth day of menstruation to participants without amenorrhea in the study and control groups. Participants will receive treatment with an initial oral dose of 50 mg for 5 continuous days. The acupuncture treatment will be conducted three times a week from the fifth day of menstruation or withdrawal bleeding until the start of the next menstruation, for up to three menstrual cycles. Acupuncture points will be selected based on the Zang-fu organ system, Yin-Yang theory, and clinical rules for PCOS acupoint selection. Two sets of acupoints will be used; each set will be used on alternate treatments. The first acupoint formula comprises DU-20, DU-24, GB-13, RN-12, ST-25, RN-4, EX-CA-1, KI-12, SP-6, and LR-3. The second acupoint formula comprises BL-23, BL-32, SP-6, and KI-3. Disposable, single-use, sterilized needles (Huatuo, Suzhou Medical Appliance Fact. 215005 Suzhou, China) of sizes 0.25mm × 25mm, 0.25mm × 40mm, and 0.25mm × 50mm will be inserted into the acupoints, and a Deqi sensation obtained by manipulating the needles. Each treatment will last for 30 min; no manipulation will be used once the Deqi sensation is achieved. The acupuncture treatment will be conducted three times a week from the fifth day of menstruation or withdrawal bleeding until the start of the next menstruation. The intervention lasted for three menstrual cycles in both groups. **Supplementary Data Sheet 1** presents detailed intervention methods.

### 2.5 Outcomes

The primary outcome was the change in LH/FSH from baseline to after three menstrual cycles. Data were collected before the start of the study (baseline assessment) and after three menstrual cycles. **Supplementary Data Sheet 1** presents the reasons for selection.

The secondary outcomes included changes in endometrial thickness, follicular diameter, LH, FSH, testosterone, estradiol, prolactin, progesterone, fasting blood glucose, fasting insulin, c peptide, triglyceride, total cholesterol, high-density lipoprotein (HDL), low-density lipoprotein (LDL), and C-reactive protein levels; homeostasis model assessment-IR (HOMA-IR) values; Self-Rating Anxiety Scale (SAS) scores; Self-Rating Depression Scale (SDS) scores; and gut microbiome after three menstrual cycles. Safety results and adverse reactions to acupuncture, such as local subcutaneous bleeding, bruising, hematoma, pain, and fainting, were also documented.

### 2.6 Fecal microbiological outcome

Fecal specimens were collected before the start of the study (baseline assessment) and after three menstrual cycles from patients who met the requirements for collecting intestinal flora in this study. The stool collection methods are detailed in **Supplementary Data Sheet 1**. The method and process of metagenomic sequencing, as well as the splitting, assembling, filtering of sequencing results, removal of chimeras, species analysis, functional analysis, etc. are presented in **Supplementary Data** in detail (S1). Bar plot, and heatmap of taxonomy analysis were used to species composition. The Wilcoxon rank sum test, and linear discriminant analysis effect size were used to analysis the difference for group. We perform Alpha/Beta diversity analysis for each sample/group. The Chao1, Shannon, and Simpson index reflect the alpha diversity of the community. The bray distance of the samples is calculated according to the abundance matrix to measure Beta diversity. We also compared the intestinal flora genes with the KEGG database to obtain each functional gene (KO), and the difference KO between groups was tested using the Wilcoxon rank-sum test. In addition, gene pathway annotations were obtained based on KEGG annotation information, and the Reporter Score37 method was used to analyze pathway differences. It was also compared to other gene databases as a sensitivity analysis.

### 2.7 Statistical analysis

The sample size was calculated based on the results of a previous study to observe acupuncture’s effects on PCOS and noted an average LH/FSH decrease of 0.5 (36). However, analysis of the effects of clomiphene on PCOS in another study showed an average LH/FSH decrease of 0.53 (37). In our study, PASS software (version 15.0, NCSS, LLC, East Kayville, UT, USA) was used for sample size calculation. We expected AC and clomiphene to reduce LH/FSH by 1 and 0.5, respectively, setting the standard deviation (SD) to 0.7. We assumed 80% power to detect a between-group difference in the LH/FSH of 0.5 at a significance level of 0.05. A total of 37 participants were required in each group. Assuming a 15% dropout rate, we recruited 86 patients. In this study, the intention-to-treat (ITT) analysis population, consisting of all patients randomized to either group, was the primary population for efficacy and safety analyses.

Multiple imputations supplemented the missing data for all outcome indicators to complete the ITT analysis dataset. Specifically, the predicted mean-matching model was used, the number of iterations was set to 100, and five ITT datasets were generated. Sensitivity analysis was also performed using patients with complete outcome data (per protocol [PP]). An independent sample t-test was used to analyze continuous variables for baseline data analysis, with the results presented as mean ± SD. The chi-square test was used to analyze categorical variables, with the results presented as numbers and percentages. For the outcome data after three menstrual cycles and changes relative to the baseline, we used an analysis of covariance with age, body mass index, and baseline outcome data as covariates. We plotted a histogram of the residuals to evaluate the normality of the data.

We used the mean, 95% confidence interval (CI), and statistical significance to analyze the outcome indicators. All data analyses were performed using SPSS version 27. This study considered two-tailed *p* < 0.05 as indicating statistical significance for the primary outcome indicators. The Bonferroni method was used for the secondary outcome indicators for multiple comparisons. A total of 20 outcome indicators, with average distribution alphas of 0.05 and *p* < 0.0025, were considered statistically significant. Adverse events (AEs) and serious AEs (SAEs) were also described.

The diversity and relative abundance of the gut microbiota genera and species were analyzed using the Wilcoxon signed-rank sum test and are expressed as medians (interquartile ranges). Linear discriminant analysis effect size (LEfSe) was used to analyze the differences in microbial populations between the AC and control groups, as well as healthy controls, after the treatment while distinguishing the acupuncture-effective samples from the acupuncture-ineffective samples according to the changes in LH/FSH after acupuncture and analyzing the differences in gut flora. Linear discriminant analysis > 2.0 was considered a microorganism with a significant effect. Microbial bioinformatics analysis was performed using R (version 3.1.1, Ross Ihaka and Robert, Auckland, New Zealand). Correlations among clinical variables, bacterial flora, and inflammatory markers were analyzed using linear regression. *p* values ≤0.05 were considered statistically significant. Further details are provided in **Supplementary Data Sheet 1**. Statistical analyses were performed using SPSS version 27.

## 3 Results

### 3.1 Patient characteristics

**Figure 1:**
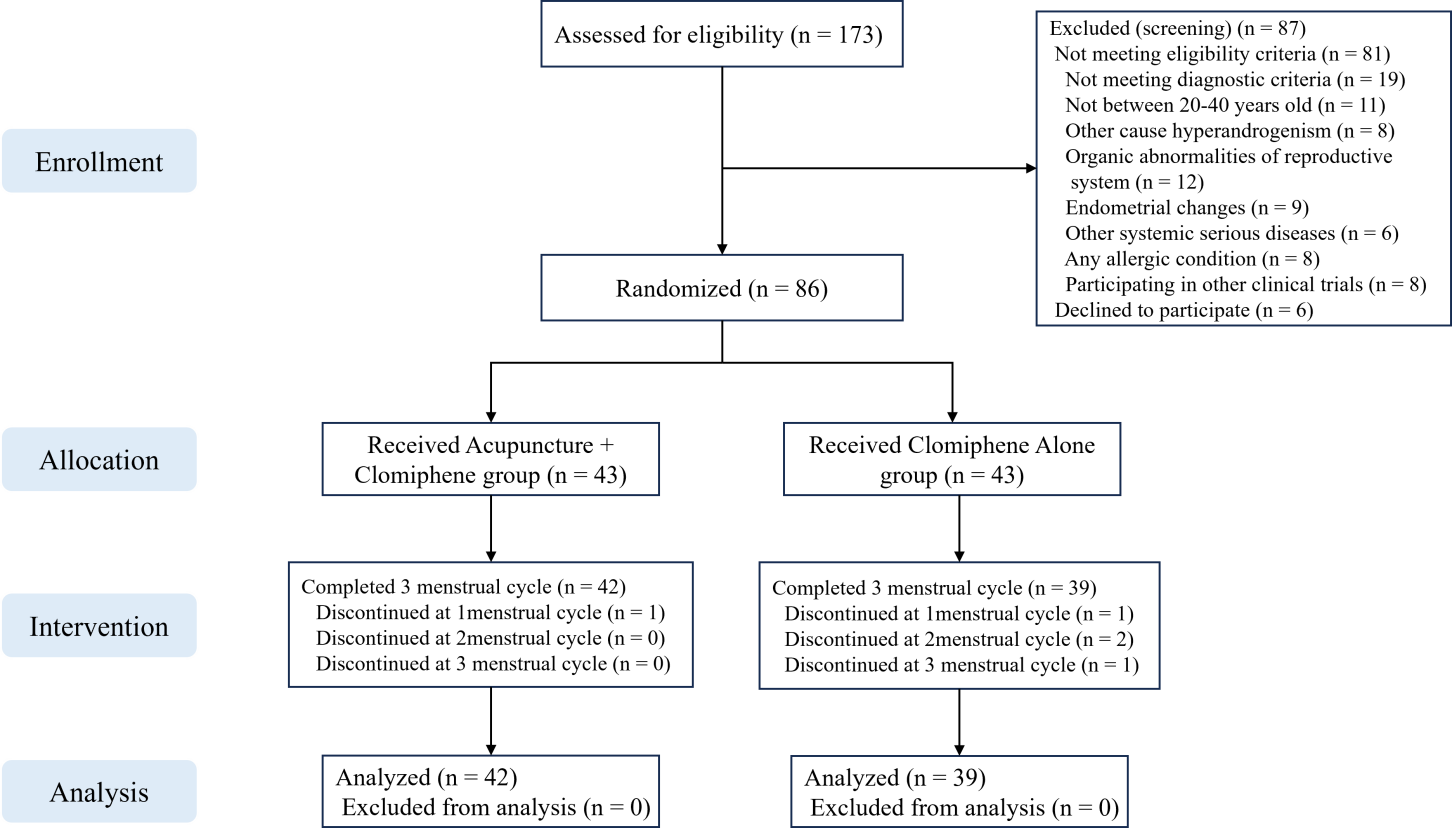
The CONSORT flowchart of the patient flow throughout the study.

A total of 173 patients were screened, of whom 87 were excluded. Finally, 86 patients were randomly assigned to the AC group (mean age: 26.95 [SD: 3.8]) and the clomiphene alone group (mean age: 27.28 [SD: 2.74]). Five (15.0%) patients dropped out during the study, including one (2.3%) in the AC group and four (9.3%) in the clomiphene group (**Figure 1**). The baseline characteristics of the patients are presented in **Table 2**. There were no significant differences between the AC and clomiphene groups regarding any demographic features and no statistically significant differences in health-related indicators such as hormone levels, blood lipid levels, C-reactive protein levels, and SAS and SDS scores. The PP analysis included 81 patients with OPCOS (42 in the AC and 39 in the clomiphene group), and the differences in baseline features between the AC and clomiphene groups were similar to those observed in the ITT analysis (**Supplementary Table S1**).

**Table 2:** Baseline Characteristics of the intention-to-treat population.

Characteristics	Acupuncture+ clomiphene (n = 43)	Clomiphene (n = 43)	*P*
Age (years), mean (SD)	26.95 (3.8)	27.28 (2.74)	0.65
Body mass index (kg/m^2^), mean (SD)	27.43 (1.94)	27.17 (1.36)	0.47
Irregular menstruation (years), mean (SD)	8.44 (5.78)	8.47 (5.78)	0.99
Infertility (years), mean (SD)	1.14 (1.23)	0.91 (0.95)	0.33
Antidiabetic, n (%)	8 (18.6%)	9 (20.9%)	0.78
Endometrial thickness (mm), mean (SD)	5.98 (1.74)	6 (2.17)	0.96
Follicular diameter (mm), mean (SD)	8.86 (1.55)	8.58 (1.74)	0.43
LH (mIU/mL), mean (SD)	12.75 (5.16)	12.28 (5.64)	0.68
FSH (mIU/mL), mean (SD)	5.98 (1.06)	5.8 (1.35)	0.51
LH/FSH, mean (SD)	2.17 (0.85)	2.13 (0.95)	0.86
T (ng/dL), mean (SD)	43.45 (13.32)	40.71 (16.87)	0.40
E2 (pg/mL), mean (SD)	42.37 (14.81)	41.17 (20.52)	0.76
PRL (uIU/mL), mean (SD)	282.03 (60.68)	280.3 (63.41)	0.90
P (ng/mL), mean (SD)	0.31 (0.14)	0.28 (0.16)	0.28
Fasting blood glucose (mmol/L), mean (SD)	5.3 (0.35)	5.16 (0.43)	0.11
Fasting insulin (mIU/L), mean (SD)	13.73 (6.1)	13.06 (6.05)	0.61
HOMA IR, mean (SD)	3.26 (1.53)	2.98 (1.38)	0.38
C Peptide (nmol/L), mean (SD)	2.07 (0.87)	1.86 (0.83)	0.24
Triglyceride (mmol/L), mean (SD)	2.53 (1.05)	2.35 (1.05)	0.45
Total cholesterol (mmol/L), mean (SD)	4.68 (0.8)	4.45 (0.75)	0.16
HDL (mmol/L), mean (SD)	1.46 (0.4)	1.33 (0.47)	0.18
LDL (mmol/L), mean (SD)	3.31 (0.63)	3.12 (0.77)	0.19
C reaction (mg/L), mean (SD)	5.57 (1.98)	4.75 (1.94)	0.06
SAS, mean (SD)	61.4 (7.82)	58.14 (11.16)	0.12
SDS, mean (SD)	58.09 (9.75)	60.53 (8.9)	0.23

### 3.2 Primary outcome

The ITT analysis of the first multiple interpolation set revealed that the adjusted mean decrease in LH/FSH was -0.8 (95%CI: -1.14 to -0.46) in the AC group and -0.22 (95%CI: -0.47 to 0.01) in the clomiphene group (**Supplementary Table S1**). The adjusted between-group difference in the five summarized ITT sets was 0.53 (95%CI: 0.24 to 0.82; *p* < 0.001; **Table 3**). The adjusted mean LH/FSH decreased by -0.77 (95%CI: -1.12 to -0.43) in the AC group and -0.19 (95%CI: -0.46 to 0.07) in the clomiphene group. The adjusted between-group difference was 0.52 (95%CI: 0.22 to 0.82; *p* = 0.001) according to the PP analysis (**Supplementary Table S2**).

**Table 3:** Primary and secondary outcomes.

Outcome	Clomiphene (n = 39)	Acupuncture + Clomiphene (n = 42)	Adjusted difference	*P*
Endometrial thickness, mm				
At three menstrual cycles	6.48 (5.69, 7.28)	7.59 (6.97, 8.22)	-0.99 (-1.92, -0.05)	**0.030**
Change (0-three menstrual cycles)	0.51 (-0.25, 1.27)	1.57 (0.7, 2.43)	-0.99 (-1.92, -0.05)	**0.030**
Follicular diameter, mm				
At three menstrual cycles	15.05 (13.6, 16.5)	18.12 (16.89, 19.34)	-2.82 (-4.59, -1.06)	**0.002**
Change (0-three menstrual cycles)	6.54 (5.12, 7.96)	9.19 (7.85, 10.53)	-2.82 (-4.59, -1.06)	**0.002**
LH, mIU/mL				
At three menstrual cycles	11.23 (10.09, 12.37)	7.38 (6.19, 8.57)	4.09 (2.55, 5.62)	**<0.001**
Change (0-three menstrual cycles)	-0.63 (-2.2, 0.94)	-5.4 (-7.31, -3.5)	4.09 (2.55, 5.63)	**<0.001**
FSH, mIU/mL				
At three menstrual cycles	5.94 (5.52, 6.35)	5.84 (5.27, 6.41)	0.27 (-0.35, 0.89)	0.425
Change (0-three menstrual cycles)	0.22 (-0.12, 0.57)	-0.12 (-0.72, 0.47)	0.27 (-0.34, 0.89)	0.425
LH/FSH				
At three menstrual cycles	1.89 (1.71, 2.06)	1.39 (1.13, 1.64)	0.53 (0.24, 0.82)	**0.001**
Change (0-three menstrual cycles)	-0.19 (-0.46, 0.07)	-0.77 (-1.12, -0.43)	0.53 (0.24, 0.82)	**<0.001**
T, ng/dL				
At three menstrual cycles	45.3 (37.8, 52.79)	18.94 (11.16, 26.72)	25.12 (14.92, 35.33)	**<0.001**
Change (0-three menstrual cycles)	4.42 (-4.76, 13.6)	-24.14 (-32.91, -15.36)	25.09 (14.88, 35.29)	**<0.001**
E2, pg/mL				
At three menstrual cycles	57.31 (48.85, 65.77)	62.43 (53.82, 71.05)	-1.72 (-13.01, 9.57)	0.481
Change (0-three menstrual cycles)	18.8 (8.42, 29.17)	20.48 (11.31, 29.65)	-1.76 (-13.05, 9.54)	0.481
PRL, uIU/mL				
At three menstrual cycles	372.28 (342.74, 401.81)	389.65 (352.22, 427.08)	-13.05 (-57.76, 31.67)	0.415
Change (0-three menstrual cycles)	96.85 (63.59, 130.11)	106.42 (64.19, 148.64)	-13.12 (-57.84, 31.6)	0.415
P, ng/mL				
At three menstrual cycles	0.47 (0.39, 0.54)	0.41 (0.35, 0.47)	0.07 (-0.03, 0.16)	0.266
Change (0-three menstrual cycles)	0.2 (0.12, 0.28)	0.1 (0.02, 0.18)	0.07 (-0.03, 0.16)	0.266
Fasting blood glucose, mmol/L				
At three menstrual cycles	5.22 (5.05, 5.38)	4.91 (4.76, 5.06)	0.3 (0.07, 0.53)	**0.006**
Change (0-three menstrual cycles)	0.05 (-0.12, 0.23)	-0.39 (-0.56, -0.21)	0.3 (0.07, 0.53)	**0.006**
Fasting insulin, mIU/L				
At three menstrual cycles	14.73 (13.01, 16.45)	10.91 (9.2, 12.61)	4.29 (1.94, 6.63)	**0.002**
Change (0-three menstrual cycles)	2.25 (-0.13, 4.65)	-2.96 (-5.53, -0.38)	4.3 (1.95, 6.65)	**0.002**
HOMA IR				
At three menstrual cycles	3.42 (3, 3.84)	2.39 (2, 2.77)	1.13 (0.61, 1.66)	**<0.001**
Change (0-three menstrual cycles)	0.57 (0.03, 1.11)	-0.9 (-1.5, -0.3)	1.14 (0.61, 1.66)	**<0.001**
C Peptide, nmol/L				
At three menstrual cycles	3.17 (2.66, 3.69)	3.21 (2.67, 3.75)	-0.03 (-0.71, 0.66)	0.866
Change (0-three menstrual cycles)	1.25 (0.84, 1.67)	1.11 (0.51, 1.7)	-0.02 (-0.7, 0.66)	0.866
Triglyceride, mmol/L				
At three menstrual cycles	2.66 (2.18, 3.15)	2.45 (2, 2.91)	0.28 (-0.28, 0.85)	0.287
Change (0-three menstrual cycles)	0.23 (-0.2, 0.68)	-0.06 (-0.53, 0.39)	0.28 (-0.29, 0.85)	0.287
Total cholesterol, mmol/L				
At three menstrual cycles	3.87 (3.43, 4.32)	4.24 (3.76, 4.72)	-0.12 (-0.74, 0.49)	0.541
Change (0-three menstrual cycles)	-0.5 (-0.99, -0.01)	-0.43 (-0.92, 0.04)	-0.12 (-0.74, 0.49)	0.541
HDL, mmol/L				
At three menstrual cycles	1.08 (1.02, 1.14)	1.13 (1.07, 1.2)	-0.06 (-0.14, 0.03)	0.215
Change (0-three menstrual cycles)	-0.26 (-0.42, -0.1)	-0.32 (-0.48, -0.17)	-0.06 (-0.15, 0.03)	0.215
LDL, mmol/L				
At three menstrual cycles	3.64 (3.2, 4.07)	4.11 (3.67, 4.55)	-0.42 (-1.06, 0.22)	0.134
Change (0-three menstrual cycles)	0.61 (0.14, 1.09)	0.79 (0.31, 1.27)	-0.42 (-1.06, 0.21)	0.134
C reaction, mg/L				
At three menstrual cycles	4.69 (4, 5.37)	5.22 (4.55, 5.89)	-0.31 (-1.18, 0.56)	0.513
Change (0-three menstrual cycles)	-0.2 (-1.02, 0.6)	-0.34 (-1.03, 0.33)	-0.31 (-1.18, 0.56)	0.513
SAS				
At three menstrual cycles	45 (41.99, 48)	45.39 (42.42, 48.36)	2.44 (-1.27, 6.16)	0.370
Change (0-three menstrual cycles)	-11.97 (-15.64, -8.29)	-16.46 (-19.71, -13.2)	2.42 (-1.3, 6.13)	0.370
SDS				
At three menstrual cycles	49.74 (44.87, 54.6)	48.36 (43.54, 53.18)	1.55 (-4.88, 7.98)	0.702
Change (0-three menstrual cycles)	-10.97 (-16.06, -5.88)	-10.12 (-16.11, -4.12)	1.53 (-4.89, 7.95)	0.702

Bold values represent p < 0.05, indicating statistical significance.

### 3.3 Secondary outcomes

The ITT analysis of the first multiple interpolation set showed that the follicular diameter increased by 9.25 (95%CI: 7.97 to 10.53) in the AC group and 6.53 (95%CI: 5.25 to 7.82) in the clomiphene group (**Supplementary Table S1**). The adjusted between-group difference in the five summarized ITT sets was -2.82 (95%CI: -4.59 to -1.06; *p* = 0.002). The LH decreased by -5.5 (mIU/mL) (95%CI: -7.37 to -3.63) in the AC group and -1.04 (mIU/mL) (95%CI: -2.54 to 0.44) in the clomiphene group (**Supplementary Table S1**). The adjusted between-group difference in the five summarized ITT sets was 4.09 (95%CI: 2.55 to 5.63; *p* < 0.001). The AC group showed a decrease of -24.52 (ng/dL) (95%CI: -33.03 to -16.01), and the clomiphene group showed an increase of 2.31 (ng/dL) (95%CI: -6.3 to 10.93) in testosterone levels. The AC group showed a decrease of -2.77 (mIU/L) (95%CI: -5.25 to -0.29), and the clomiphene group showed an increase of 2.21 (mIU/L) (95%CI: 0 to 4.43) in fasting insulin levels. The AC group showed a decrease of -0.82 (95%CI: -1.4 to -0.24), and the clomiphene group showed an increase of 0.52 (95%CI: 0.03 to 1.02) in HOMA-IR values. There were no statistically significant differences in the other outcomes (**Table 3**; **Supplementary Tables S1**-**S3**).

### 3.4 Fecal microbiological outcome

In total, 59 subjects were included in the metagenomic analysis, with 21 patients in the AC group, 19 in the clomiphene group, and 19 in the healthy control group. The basic information is presented in **Supplementary Table S4**. There were significant differences in the structure, diversity, and function of the intestinal flora between patients with OPCOS and healthy controls. After AC treatment, the abundance of *Erysipelatoclostridium* and *Proteus* decreased at the genus level (*p* < 0.05). In contrast, the abundance of *Agathobacter faecis* increased (*p* < 0.05), and *Erysipelatoclostridium spiroforme*, *Streptococcus lutetiensis*, and *Lactococcus lactis* decreased (*p* < 0.05) at the species level. However, there were no statistically significant differences in terms of diversity. An LEfSe revealed that *A. faecis* was significantly enriched after acupuncture. The functional analysis revealed statistically significant differences in 54 cases of Kyoto Encyclopedia of Genes and Genomes ontology (KO) before and after acupuncture (*p* < 0.05). The top 10 KOs were mainly related to proteins and transporters involved in genetic material metabolism, carbohydrate transport and metabolism, and oxidative stress. See **Supplementary Data Sheets 1** and **2** for the detailed gut microbiota analysis results.

### 3.5 Safety

During the study, there were 55 acupoint bleeding events, which were treated by applying pressure with sterile cotton swabs. There were also 23 local acupoint bruises, seven cases of local pain after acupuncture, and one local hematoma at the acupoint, none of which required further treatment. These AEs are common phenomena in clinical acupuncture, and the treatment methods are also based on the routine clinical treatment methods provided in the textbook “Acupuncture and Moxibustion”. No SAEs occurred during the study period.

## 4 Discussion

This was an open-label, randomized, parallel-group controlled trial investigating the efficacy of AC treatment for OPCOS. The results support the idea that AC modulates hormone levels in OPCOS, although the gut microbiota may play only a minor role in the effects of AC on OPCOS. We found that AC reduced the LH/FSH ratio in patients with OPCOS to a greater extent than clomiphene alone. In addition, there was no difference in drug use between the two groups. Therefore, it is reasonable to assume that the additional improvement effect on the LH/FSH ratio in the AC group may have stemmed from the effects of acupuncture. The study also demonstrated that adding acupuncture did not lead to an increase in AEs, and no participants withdrew from the study due to acupuncture-related adverse effects. This suggests that acupuncture has a favorable safety and compliance profile, with high patient acceptance. The results of the ITT and PP analyses were consistent, further enhancing the reliability of the findings. Thus, considering that the current treatment methods for OPCOS are limited, AC may be used as an expectant treatment.

This study found that AC treatment led to a decrease in LH/FSH from 2.2 (SD = 0.9) to 1.4 (SD = 1.2), mainly because of a decrease in LH levels rather than changes in FSH levels. This is consistent with the results of a previous study that found that acupuncture alone reduced LH/FSH in OPCOS from 3.0 (SD = 0.8) to 1.6 (SD = 0.8) and that there was no difference in the results when compared with those of cyproterone acetate and ethinyl estradiol (38). A Chinese study suggested that acupuncture might modulate the hypothalamic-pituitary-ovarian (HPO) axis (39). HPO axis disorders are one of the main pathological features in PCOS patients. When HPO axis disorders occur, they lead to increased LH release and an elevated LH/FSH ratio (40). Increased pituitary sensitivity to gonadotropin-releasing hormone (GnRH) results in enhanced LH secretion (41). Study has shown that acupoint stimulation raises the levels of beta-endorphin in both the central and peripheral circulatory systems, which inhibits GnRH secretion and thus reduces LH levels (42). Meanwhile, Wang et al. found that EA could alleviate the decrease in GnRH and the increase in LH in PCOS-like rats by inhibiting the upregulation of kisspeptin protein in the hypothalamus and arcuate nucleus (43).

This study also found that AC treatment showed an increase in the follicular diameter from 8.9 (mm) (SD = 1.6) to 18.1 (mm) (SD = 5.6) and improved the quality of follicles when compared with treatment with clomiphene alone. This finding is consistent with the results of several previous studies (44, 45). Additionally, studies have shown that excessive insulin accumulation can increase the GnRH pulse frequency in PCOS patients, upregulate LH receptor binding sites, and reduce circulating sex hormone-binding globulin (SHBG) levels (46, 47). Moreover, AC treatment improved IR in OPCOS patients (48–50). Studies have found that AC affects both glucose and lipid metabolism. However, no effect on lipid metabolism was observed in this study (51). These evidences suggest that AC could improve a variety of symptoms in patients with OPCOS, as evidenced by lowering LH/FSH, increasing follicle diameter and endometrial thickness, lowering T levels, improving insulin resistance, as well as improving fasting blood glucose and fasting insulin levels.

Consistent with previous findings (52, 53), there was also a significant relationship between OPCOS and gut flora. The abundance of *Bacteroidetes* and *Firmicutes* was lower in patients with OPCOS than in healthy individuals. Research suggests that alterations in the gut microbiota lead to a decrease in the metabolism of glycocholic acid and tauro-ursodeoxycholic acid, resulting in reduced levels of interleukin-22 (IL-22), which is closely associated with the pathogenesis of PCOS (52). It has been found that patients with PCOS have reduced abundances of *Firmicutes*, *Proteobacteria*, and *Bacteroidetes (* 54). Furthermore, the reduced *Firmicutes*/*Bacteroidetes* ratio is also associated with obesity (55, 56). Acupuncture may effectively restore the metabolic balance of the gut microbiota in PCOS patients by modulating the brain-gut-microbiome axis (57). In this study, when considering specific species such as *Fusarium*, *Trichinella*, *Streptococcus luteus*, and *Lactococcus lactis*, AC may only induce slight changes in the gut microbiota of PCOS patients. Thursby et al. proposed that changes in the abundance of *Firmicutes* are related to metabolic health (58). Specifically, *Firmicutes* may be involved in the production of short-chain fatty acids (SCFAs) such as propionate and butyrate, which play a regulatory role in metabolic disorders such as hormone imbalance, obesity, and insulin resistance. *Clostridium spiralis*, *Streptococcus luteus*, and *Lactococcus lactis* all belong to the *Firmicutes* phylum, and acupuncture may alleviate PCOS-related pathological manifestations by modulating these microbiota. Additionally, some specific taxa within the *Proteobacteria* phylum, such as *Gammaproteobacteria* and *Mixococcales*, have been shown to correlate with estradiol levels (59). Other studies indicate that certain Proteobacteria taxa may influence obesity traits in PCOS patients (60). As part of the *Proteobacteria* phylum, the abundance of *A. faecalis* improved after acupuncture treatment, potentially alleviating PCOS symptoms. However, there is currently no clear evidence supporting the exact mechanism of this improvement, and further research is needed to explore its potential mechanisms of action.

Although this study provides important insights, certain limitations remain. First, the sample size limitation may affect the robustness of the results. Although a reasonably appropriate sample size was selected for this study, a smaller sample size may impact the results, and future research should consider expanding the sample size to validate the reliability of the findings. Second, since the main objective of this study was to assess the effect of acupuncture combined with clomiphene on obese PCOS, a separate acupuncture group was not included. Future studies could add an acupuncture-only group to investigate the therapeutic effect of acupuncture alone on patients. Finally, this study had a short follow-up period; patients will continue to be monitored over a longer duration in the future to provide a more comprehensive understanding of the treatment’s efficacy and safety.

## 5 Conclusions

This study showed that acupuncture plus clomiphene is a safe and effective treatment that reduces LH/FSH and improves IR in patients with OPCOS and that the gut microbiota may somewhat mediate these effects. The long-term curative effects and mechanisms underlying these effects of acupuncture treatment for OPCOS require further investigation.

## Data Availability

The original contributions presented in the study are included in the article/**Supplementary Material**. Further inquiries can be directed to the corresponding authors.

## Supplementary material

Supplementary Data Sheet 1 Supplement of research methods and gut microbiota analysis

Supplementary Data Sheet 2 All results of metagenomic analysis.

Supplementary Data Sheet 3
**Supplementary Tables S1**-**S4**.

Supplementary Table 1 All organized analysis results.

Supplementary Table 2 Descriptive results for all modeling analyses.

Supplementary Table 3 Statistics for all modeling analyses.

Supplementary Table 4 Baseline characteristics of metagenomic studies.
