# Supplementary material for: Acupuncture for hormonal readiness and gut microbiota in obese polycystic ovary syndrome: an open-label, randomized controlled trial
Source: Front Endocrinol (Lausanne). 2024 Dec 19;15:1509152. doi: 10.3389/fendo.2024.1509152 (PMC11693447; doi:10.3389/fendo.2024.1509152)
Supplement: Supplementary Data Sheet 1 — Supplement of research methods and gut microbiota analysis [file DataSheet1.pdf]

Detailed intervention methods.

Acupuncture treatment

Acupuncture points will be selected based on the Zang-fu organ system, Yin-Yang theory, and clinical rules for PCOS acupoint selection. Two sets of acupoints will be used; each set will be used on alternate treatments. The first acupoint formula comprises DU-20, DU-24, GB-13, RN-12, ST-25, RN-4, EX-CA-1, KI-12, SP-6, and LR-3. The second acupoint formula comprises BL-23, BL-32, SP-6, and KI-3. Disposable, single-use, sterilized needles (Huatuo, Suzhou Medical Appliance Fact. 215005 Suzhou, China) of sizes 0.25mm × 25mm, 0.25mm × 40mm, and 0.25mm × 50mm will be inserted into the acupoints, and a Deqi sensation obtained by manipulating the needles. Table 2 shows the location, depth, and type of insertion for each acupoint. Each treatment will last for 30 min; no manipulation will be used once the Deqi sensation is achieved. The acupuncture treatment will be conducted three times a week from the fifth day of menstruation or withdrawal bleeding until the start of the next menstruation, for up to three menstrual cycles.

Clomiphene citrate treatment

Clomiphene citrate treatment will be administered on the fifth day of menstruation to participants without amenorrhea in the study and control groups. Participants will receive treatment with an initial oral dose of 50 mg for 5 continuous days. If there is an ovulatory response, this dose will be maintained and will be given on the next cycle.

For patients with no ovulatory response, an additional 50 mg dose will be given on the next cycle; the maximum dose of clomiphene citrate will not exceed 150 mg. Patients with amenorrhea will receive the same pharmacological treatment regimen after withdrawal bleeding has been induced by progestin. The treatment will last for three menstrual cycles.

Reasons for selection LH/FSH as primary outcome

FSH and LH are secreted by gonadotroph cells in the anterior pituitary gland. FSH can stimulate the growth and development of follicles and the proliferation of granulosa cells, and make the follicles secrete estrogen under the stimulation of a small amount of LH. LH can promote mature follicle ovulation, corpus luteum formation and secretion of estrogen and progesterone in a certain proportion. The ratio of LH and FSH in PCOS patients is usually abnormal, resulting in abnormal follicle development and no dominant follicle discharge. Therefore, LH/FSH is an important biochemical indicator in PCOS patients.

Stool collection methods and metagenomic analysis

Method of collecting intestinal flora

The subjects were told to defecate into a plastic basin covered with a sterile plastic bag (be careful not to mix urine or other substances into it), and not touch the center of the basin during the whole process, and then the researchers used a 5ml sterile tube About 2g of feces samples were collected from the central part of the feces, then immediately placed in liquid nitrogen for 15 minutes, and then transferred to a -80°C refrigerator for storage for later use.

Metagenomic community profiling

The method and process of metagenomic sequencing, as well as the splitting, assembling, filtering of sequencing results, removal of chimeras, species analysis, functional analysis, etc. are presented in supplementary data in detail (S1). Bar plot, and heatmap of taxonomy analysis were used to species composition. The Wilcoxon rank sum test, and linear discriminant analysis effect size were used to analysis the difference for group. We perform Alpha/Beta diversity analysis for each sample/group. The Chao1, Shannon, and Simpson index reflect the alpha diversity of the community. The bray distance of the samples is calculated according to the abundance matrix to measure Beta diversity. We also compared the intestinal flora genes with the KEGG database to obtain each functional gene (KO), and the difference KO between groups

was tested using the Wilcoxon rank-sum test. In addition, gene pathway annotations were obtained based on KEGG annotation information, and the Reporter Score[37] method was used to analyze pathway differences. It was also compared to other gene databases as a sensitivity analysis.

Gut microbiota analysis results

Group abbreviation: PCOS: Polycystic ovary syndrome, HC: healthy control, A: acupuncture group before treatment, AP: acupuncture group after treatment, B: drug group before treatment, BP: drug group after treatment, AP1: acupuncture group Those who are effective after treatment, AP2: those who are ineffective after treatment in the acupuncture group.

OTU analysis

Through the species annotation of metagenomic data on the gut microbes of 40 overweight/obese PCOS patients and 19 healthy subjects, a total of 20 phyla, 1379 genera, 4512 species and their respective relative abundance information. The histogram of the species in the PCOS group and the healthy group shows that at the phylum level, the intestinal flora of the two groups are dominated by Bacteroidota and Firmicutes, and the sum of the relative abundance of the two groups exceeds 90% (Figure. 4, 5 in supplementary 4), which can be considered as the main constituent species of the intestinal flora of the two groups of subjects. In addition, there were differences in the distribution of Bacteroidota and Firmicutes between the two groups (Figure. 4, 5 in supplementary 4). The distribution of Bacteroidota in the PCOS group was higher than that in the healthy group, and the distribution of Firmicutes in the PCOS group was lower than that in the healthy group. The abundances of Bacteroidota in PCOS group and healthy group were 0.68 and 0.63, and the abundances of Firmicutes were 0.29 and 0.34, respectively (Figure. 4, 5 in supplementary 4). At the genus level,

species with a higher proportion of the intestinal flora community abundance in the two groups included Bacteroides and Prevotella, and the abundance of Bacteroides in the PCOS group was higher than that in the healthy group, while the abundance of Prevotella was lower than that in the healthy group (Figure. 11, 12 in supplementary 4). At the species level, species with a higher proportion of intestinal flora community abundance in the two groups include Bacteroides dorei, Prevotella corpori, Bacteroides plebelus, *etc*. (Figure. 13, 14 in supplementary 4). The relative abundance of strains was higher than that of the healthy group, and the strains belonging to Prevotella were relatively lower than that of the healthy group. After treatment in the acupuncture group, the abundances of Erysipelatoclostridium and Proteus decreased at the genus level ($P<0.05$); the abundances of Agathobacter faecis increased at the species level ($P < 0.05$), and the abundances of Erysipelatoclostridium spiroforme, Streptococcus lutetiensis, and Lactococcus lactis decreased ($P < 0.05$). There was no significant difference in the abundance of other species before and after acupuncture treatment ($P > 0.05$). There was no significant difference in the abundance of all species in the control group before and after all species treatments ($P > 0.05$).

Diversity index analysis

The alpha diversity of gut microbiota was not different between OPCOS patients and healthy controls (Figure. 72, 73, 82, 83, 92, 93, 102, 103, 112 ,113 ,122, 123, 132, 133 in supplementary 4). There were differences in β diversity between PCOS group and healthy group (phylum level: F = 0.04, R2 = 1.06, $P = 0.03$; genus level: F = 0.27, R2

= 1.31, P = 0.03; species level: F = 0.34, R2 = 1.27, P =0.03) (Figure. 142, 147, 152, 157 in supplementary 4). The principal coordinate analysis (PCoA) based on bacterial abundance also showed that the PCOS group and the healthy group's intestinal flora clustered separately, and there was a partial overlap between the two (Figure. 236, 241, 246, 251 in supplementary 4), the interpretation degrees of the analysis results of the phylum level PC1 and PC2 axes were 81.01% and 7.59% respectively; the interpretation degrees of the analysis results of the genus level PC1 and PC2 axes were 44.69% and 18.12% respectively; The explanatory degrees of are 31.21% and 13.34% respectively. According to the Shannon index and Simpson index, there was no statistically significant difference in the diversity of intestinal flora between the acupuncture group and the control group before and after treatment ($P > 0.05$) (Figure. 76, 77, 86, 87, 96, 97, 106, 107, 116, 117, 126, 127, 136, 137 in supplementary 4).

Analysis of metagene species differences

Difference analysis was performed on the species abundance between the healthy group and the PCOS group at the genus and species levels. The results showed (Figure. 163-197 in supplementary 4): At the genus level, compared with the healthy group, the abundance of Bacteroides, Barnesiella, Neobitarella and Parabacteroides in the PCOS group was a significant increase ($P < 0.05$), and the abundance of Akkermansia was significantly decreased ($P < 0.05$). At the species level, compared with the healthy group, the abundance of Bacteroides barnesiae, Bacteroides acidifaciens, Bacteroides dorei, Bacteroides massiliensis, Bacteroides sartorii, Barnesiella intestinihominis,

Neobitarella massiliensis, Odoribacter massiliensis, Parabacteroides distasonis, and Prevotella corporis was significantly increased in the PCOS group (*P* < 0.05), the abundance of Faecalibacterium prausnitzii, Anaerostipes hadrus and Phascolarctobacterium succinatutens was significantly reduced (*P* < 0.05).

Lefse analysis

The intestinal flora of OPCOS was significantly enriched in Bacteroides dorei, Bacteroides massiliensis, Bacteroides sartorii and Bacteroides sp002493165; Parabacteroides distasonis in Parabacteroides; Barnesiella intestinalis intestinihominis in Barnesiella; Neobitarella m in Neobitarella assiliensis. The gut microbiota of HCs were significantly enriched in Phascolarctobacterium, Akkermansia muciniphila and Faecalibacterium prausnitzii. The significantly enriched flora after acupuncture intervention was Agathobacter faecis. The histogram of LDA value distribution is shown in Figure. 162 in supplementary 4.

Function analysis

There were 271 KOs in OPCOS patients that were significantly different from healthy ones (*P* < 0.05), and most of the top 10 most abundant KOs were proteins and transporters involved in genetic material (DNA/RNA) metabolism and glycan metabolism (Figure. 203, 208, 213, 218, 223, 228 in supplementary 4). Using Reporter Score analysis, it was found that there were significant differences in 42 metabolic pathways in the intestinal flora of OPCOS subjects and healthy people, and the

pathways with significant differences in gene abundance at the third level of KEGG metabolic pathways annotated by OPCOS were mostly related to metabolism (Metabolism), among which Among the secondary pathways, carbohydrate metabolism and global and overview maps were more enriched pathways; healthy group differential pathways were involved in metabolism, cellular processes, genetic information processing, environmental information processing and human diseases (Figure. 163-197 in supplementary 4).

There were significant differences in 54 KOs before and after acupuncture treatment ($P<0.05$) (Figure. 158, 159 in supplementary 4). The top 10 KOs were mainly related proteins and transporters involved in genetic material metabolism, carbohydrate transport metabolism, oxidative stress, etc. (Figure. 205, 210, 215, 220, 225, 230 in supplementary 4). Reporter Score method analysis found that after acupuncture treatment, carbohydrate metabolism pathways were more enriched, and pathways related to carbohydrate transport metabolism, ABC transporter and phosphotransferase system (PTS), were also enriched higher after treatment in the experimental group. In addition, pathways related to oxidative stress were also enriched after treatment.
