# Supplementary material for: Acupuncture for hormonal readiness and gut microbiota in obese polycystic ovary syndrome: an open-label, randomized controlled trial
Source: Front Endocrinol (Lausanne). 2024 Dec 19;15:1509152. doi: 10.3389/fendo.2024.1509152 (PMC11693447; doi:10.3389/fendo.2024.1509152)
Supplement: Supplementary Data Sheet 2 — All results of metagenomic analysis. [file DataSheet2.pdf]

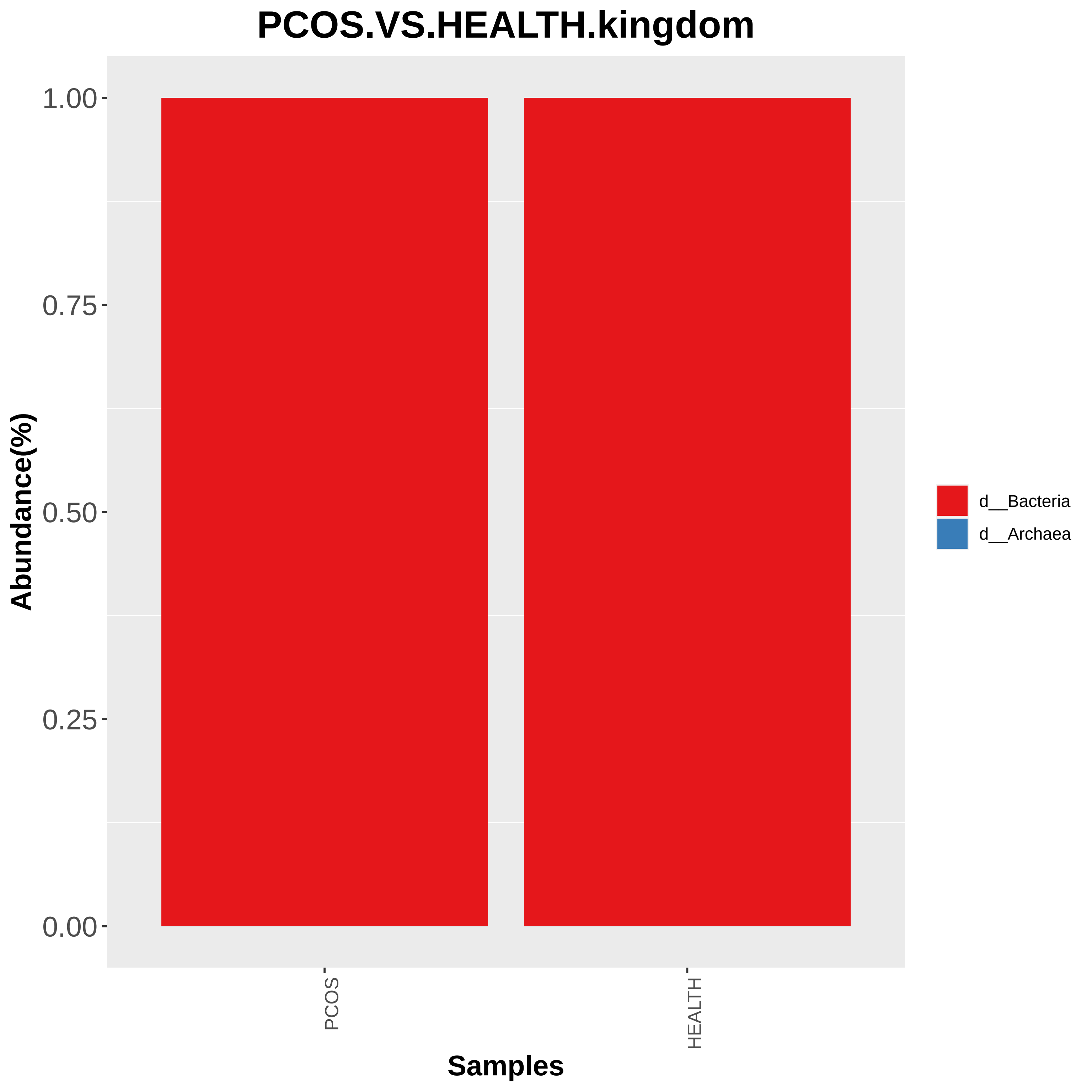
PCOS.VS.HEALTH.kingdom
Abundance(%)
1.00
0.75
0.50
0.25
0.00
PCOS
HEALTH
Samples
d__Bacteria
d__Archaea

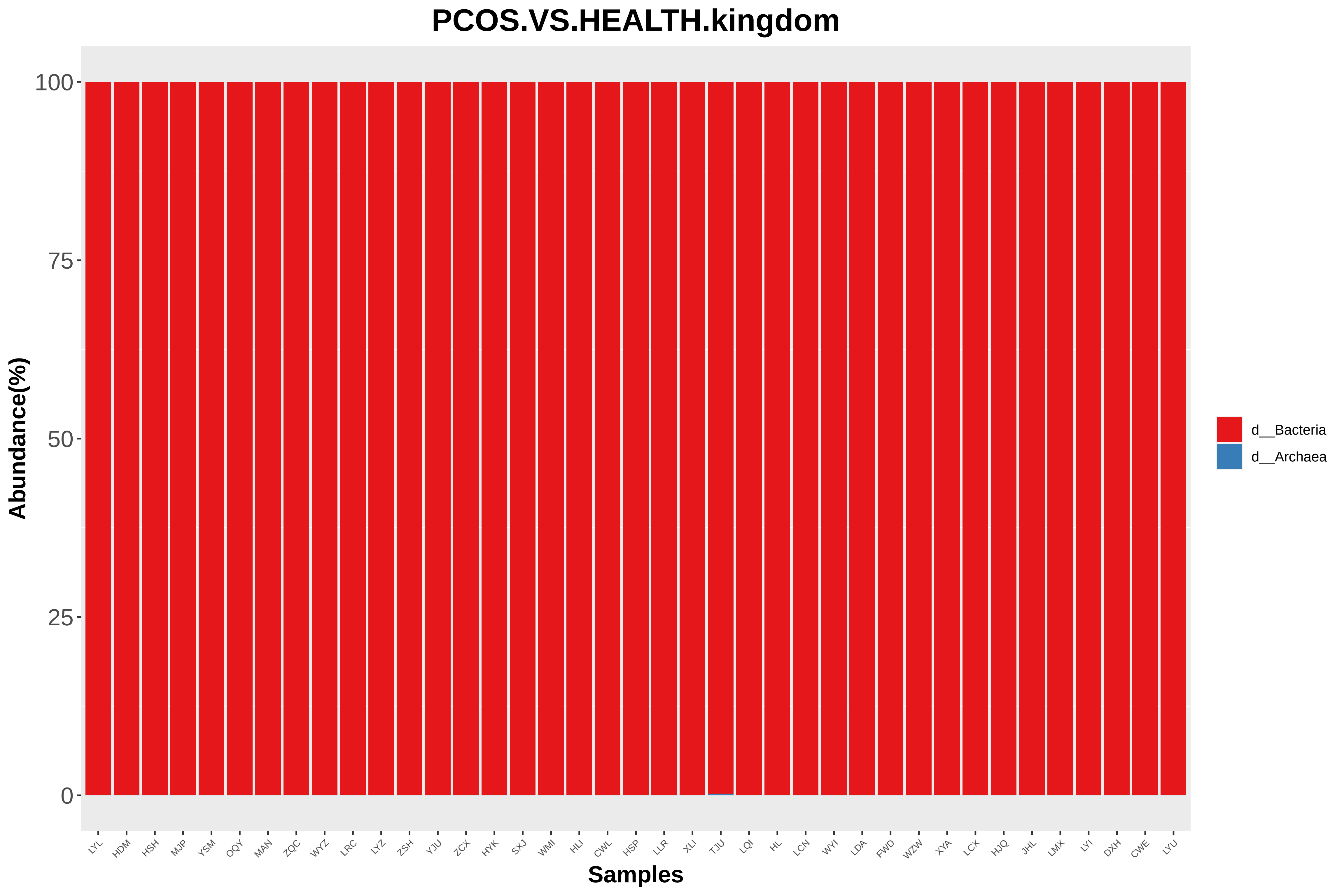
PCOS.VS.HEALTH.kingdom
Abundance(%)
100
75
50
25
0
LYL
HDM
HSH
MJP
YSM
OQY
MAN
ZQC
WYZ
LRC
LYZ
ZSH
YJU
ZCX
HYK
SXJ
WMI
HLI
CWL
HSP
LLR
XLI
TJU
LQI
HL
LCN
WYI
LDA
FWD
WZW
XYA
LCX
HJQ
JHL
LMX
LYI
DXH
CWE
LYU
Samples
d__Bacteria
d__Archaea

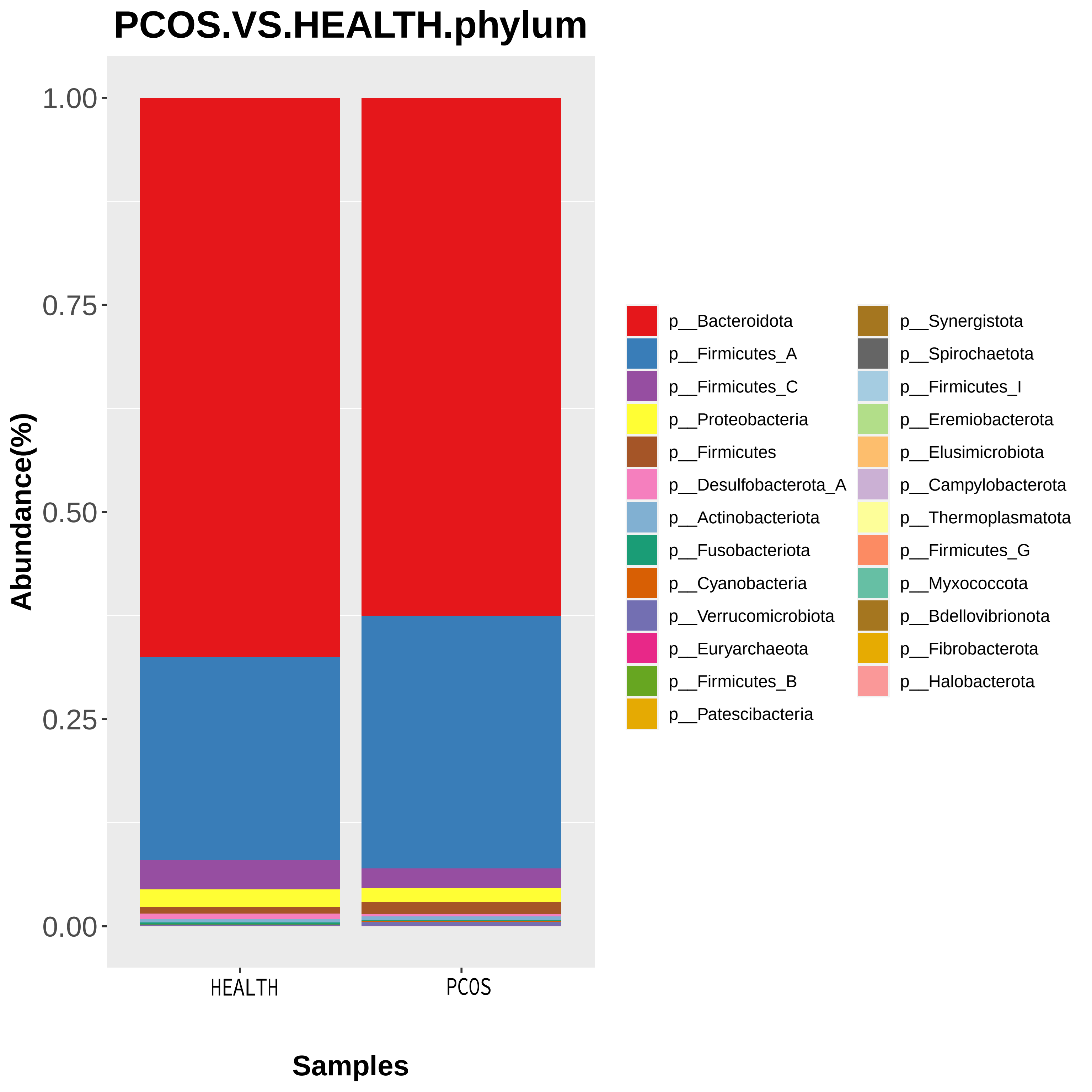

PCOS.VS.HEALTH.phylum
Abundance(%)
1.00
0.75
0.50
0.25
0.00
HEALTH
PCOS
Samples
p__Bacteroidota
p__Firmicutes_A
p__Firmicutes_C
p__Proteobacteria
p__Firmicutes
p__Desulfobacterota_A
p__Actinobacteriota
p__Fusobacteriota
p__Cyanobacteria
p__Verrucomicrobiota
p__Euryarchaeota
p__Firmicutes_B
p__Patescibacteria
p__Synergistota
p__Spirochaetota
p__Firmicutes_I
p__Eremiobacterota
p__Elusimicrobiota
p__Campylobacterota
p__Thermoplasmatota
p__Firmicutes_G
p__Myxococcota
p__Bdellovibrionota
p__Fibrobacterota
p__Halobacterota

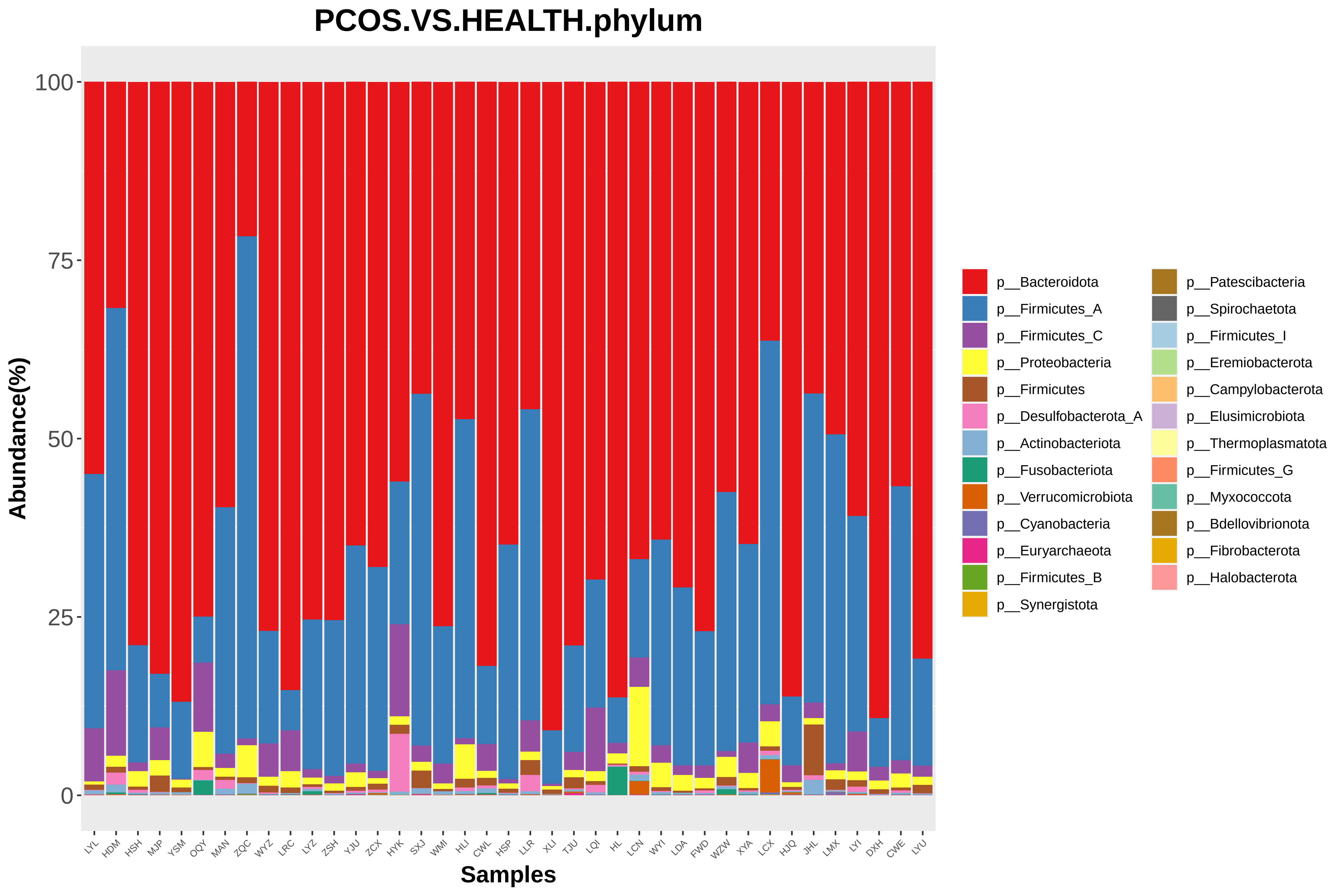
PCOS.VS.HEALTH.phylum
Abundance(%)
100
75
50
25
0
LYL
HDM
HSH
MJP
YSM
OQY
MAN
ZQC
WYZ
LRC
LYZ
ZSH
YJU
ZCX
HYK
SXJ
WMI
HLI
CWL
HSP
LLR
XLI
TJU
LQI
HL
LCN
WYI
LDA
FWD
WZW
XYA
LCX
HJQ
JHL
LMX
LYI
DXH
CWE
LYU
Samples
p__Bacteroidota
p__Firmicutes_A
p__Firmicutes_C
p__Proteobacteria
p__Firmicutes
p__Desulfobacterota_A
p__Actinobacteriota
p__Fusobacteriota
p__Verrucomicrobiota
p__Cyanobacteria
p__Euryarchaeota
p__Firmicutes_B
p__Synergistota
p__Patescibacteria
p__Spirochaetota
p__Firmicutes_I
p__Eremiobacterota
p__Campylobacterota
p__Elusimicrobiota
p__Thermoplasmatota
p__Firmicutes_G
p__Myxococcota
p__Bdellovibrionota
p__Fibrobacterota
p__Halobacterota

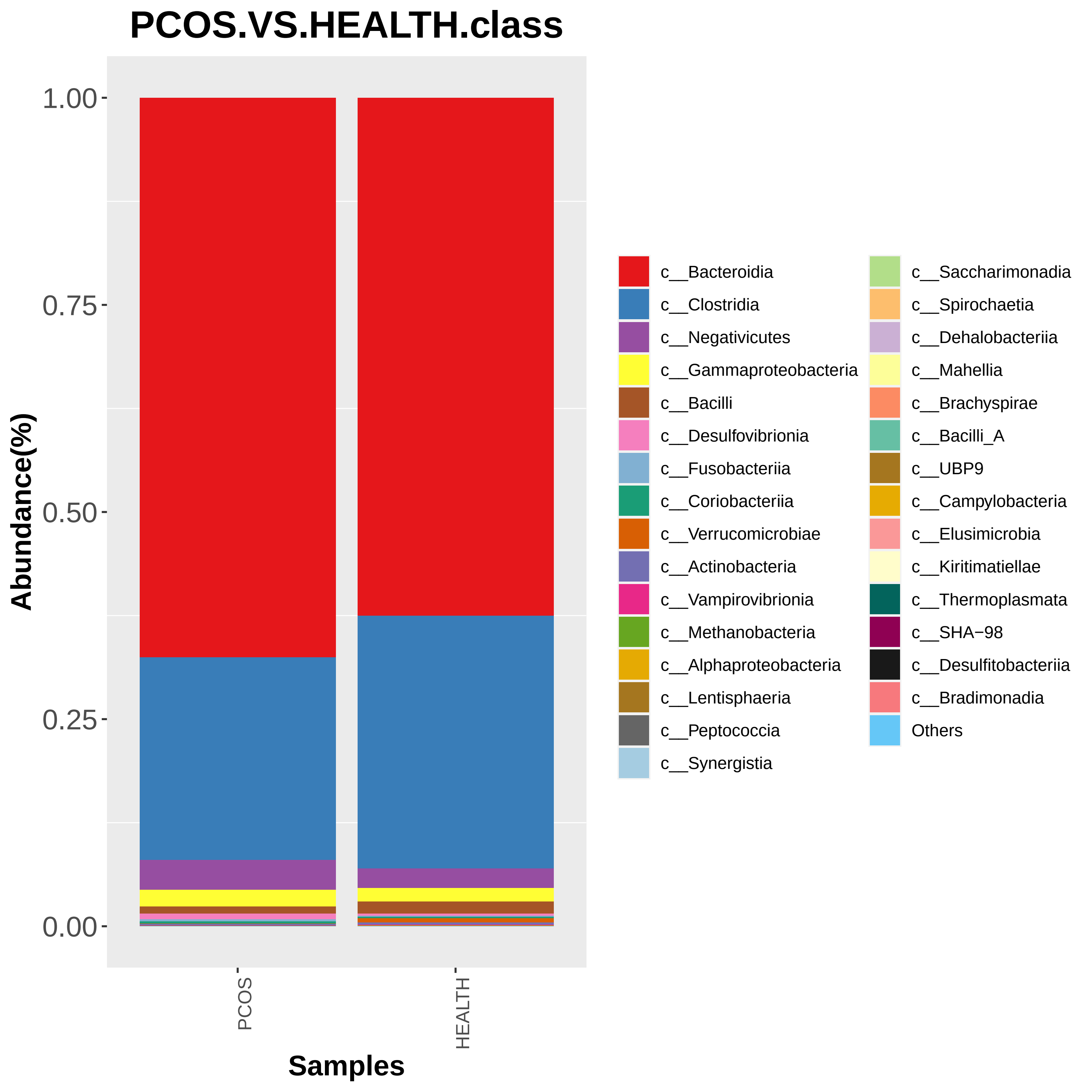
PCOS.VS.HEALTH.class
1.00
0.75
0.50
0.25
0.00
Abundance(%)
PCOS
HEALTH
Samples
c__Bacteroidia
c__Clostridia
c__Negativicutes
c__Gammaproteobacteria
c__Bacilli
c__Desulfovibrionia
c__Fusobacteriia
c__Coriobacteriia
c__Verrucomicrobiae
c__Actinobacteria
c__Vampirovibrionia
c__Methanobacteria
c__Alphaproteobacteria
c__Lentisphaeria
c__Peptococcia
c__Synergistia
c__Saccharimonadia
c__Spirochaetia
c__Dehalobacteriia
c__Mahellia
c__Brachyspirae
c__Bacilli_A
c__UBP9
c__Campylobacteria
c__Elusimicrobia
c__Kiritimatiellae
c__Thermoplasmata
c__SHA−98
c__Desulfitobacteriia
c__Bradimonadia
Others

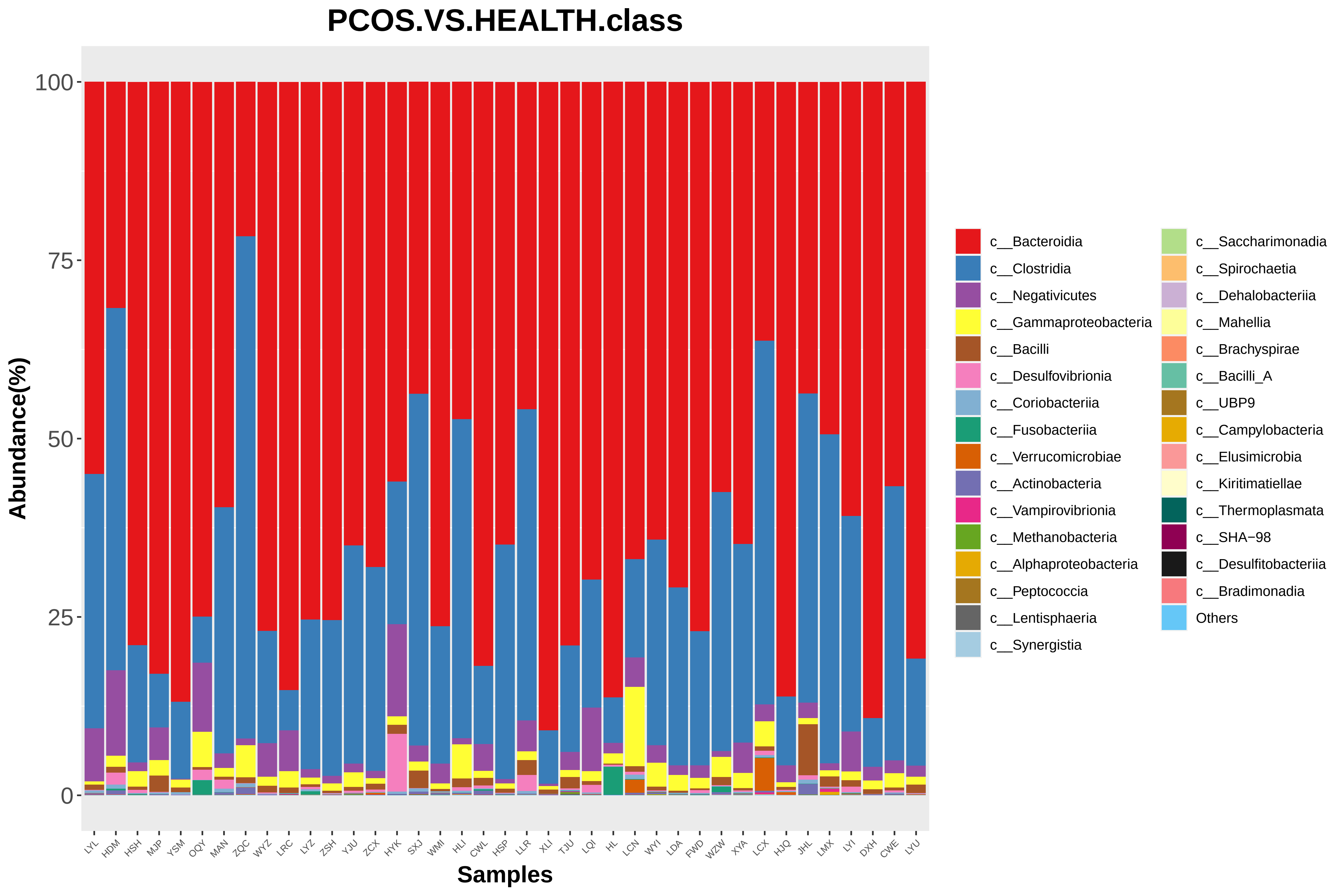
PCOS.VS.HEALTH.class
Abundance(%)
100
75
50
25
0
LYL HDM HSH MJP YSM OQY MAN ZQC WYZ LRC LYZ ZSH YJU ZCX HYK SXJ WMI HLI CWL HSP LLR XLI TJU LQI HL LCN WYI LDA FWD WZW XYA LCX HJQ JHL LMX LYI DXH CWE LYU
Samples
c__Bacteroidia
c__Clostridia
c__Negativicutes
c__Gammaproteobacteria
c__Bacilli
c__Desulfovibrionia
c__Coriobacteriia
c__Fusobacteriia
c__Verrucomicrobiae
c__Actinobacteria
c__Vampirovibrionia
c__Methanobacteria
c__Alphaproteobacteria
c__Peptococcia
c__Lentisphaeria
c__Synergistia
c__Saccharimonadia
c__Spirochaetia
c__Dehalobacteriia
c__Mahellia
c__Brachyspirae
c__Bacilli_A
c__UBP9
c__Campylobacteria
c__Elusimicrobia
c__Kiritimatiellae
c__Thermoplasmata
c__SHA−98
c__Desulfitobacteriia
c__Bradimonadia
Others

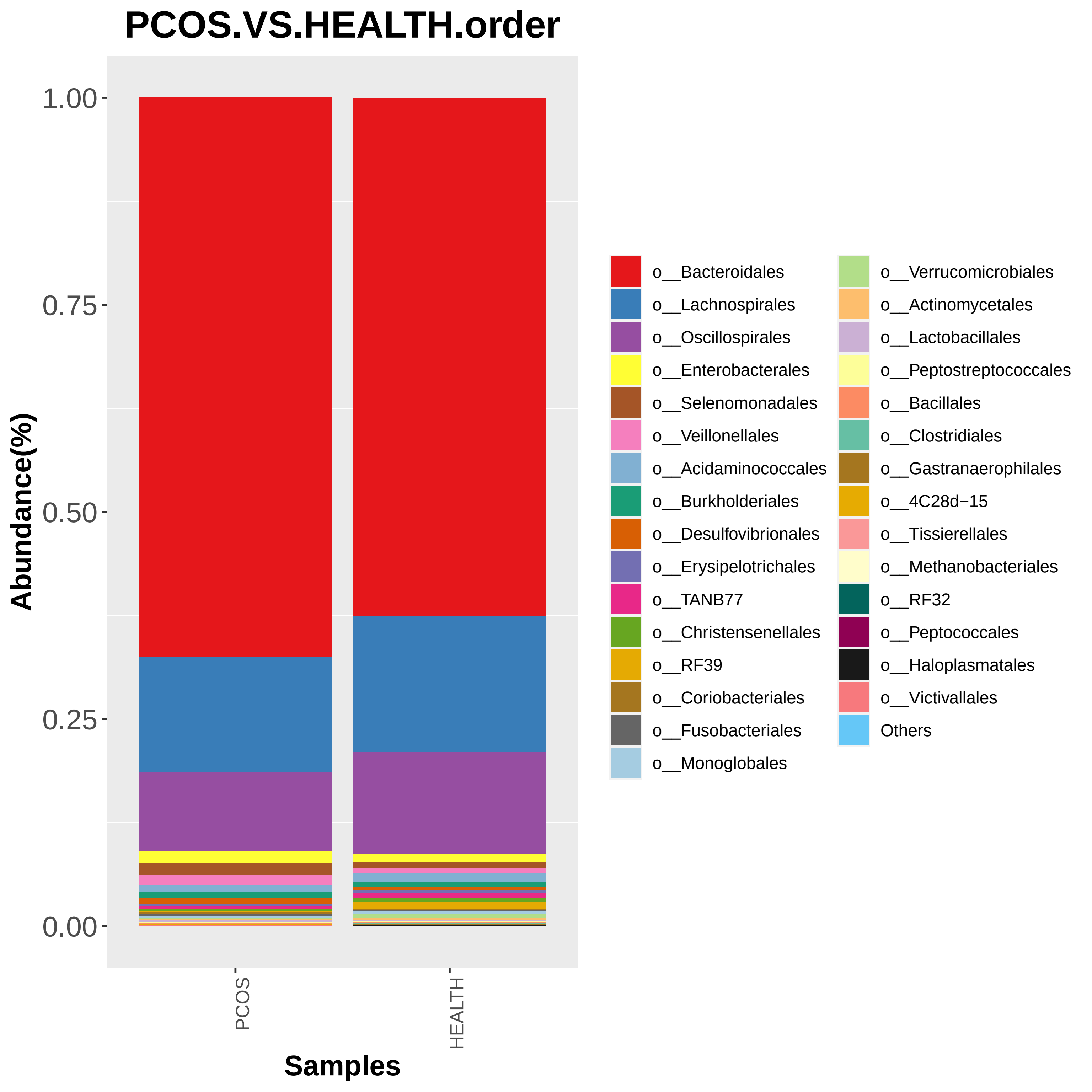
PCOS.VS.HEALTH.order
Abundance(%)
1.00
0.75
0.50
0.25
0.00
PCOS
HEALTH
Samples
o__Bacteroidales
o__Lachnospirales
o__Oscillospirales
o__Enterobacterales
o__Selenomonadales
o__Veillonellales
o__Acidaminococcales
o__Burkholderiales
o__Desulfovibrionales
o__Erysipelotrichales
o__TANB77
o__Christensenellales
o__RF39
o__Coriobacteriales
o__Fusobacteriales
o__Monoglobales
o__Verrucomicrobiales
o__Actinomycetales
o__Lactobacillales
o__Peptostreptococcales
o__Bacillales
o__Clostridiales
o__Gastranaerophilales
o__4C28d−15
o__Tissierellales
o__Methanobacteriales
o__RF32
o__Peptococcales
o__Haloplasmatales
o__Victivallales
Others

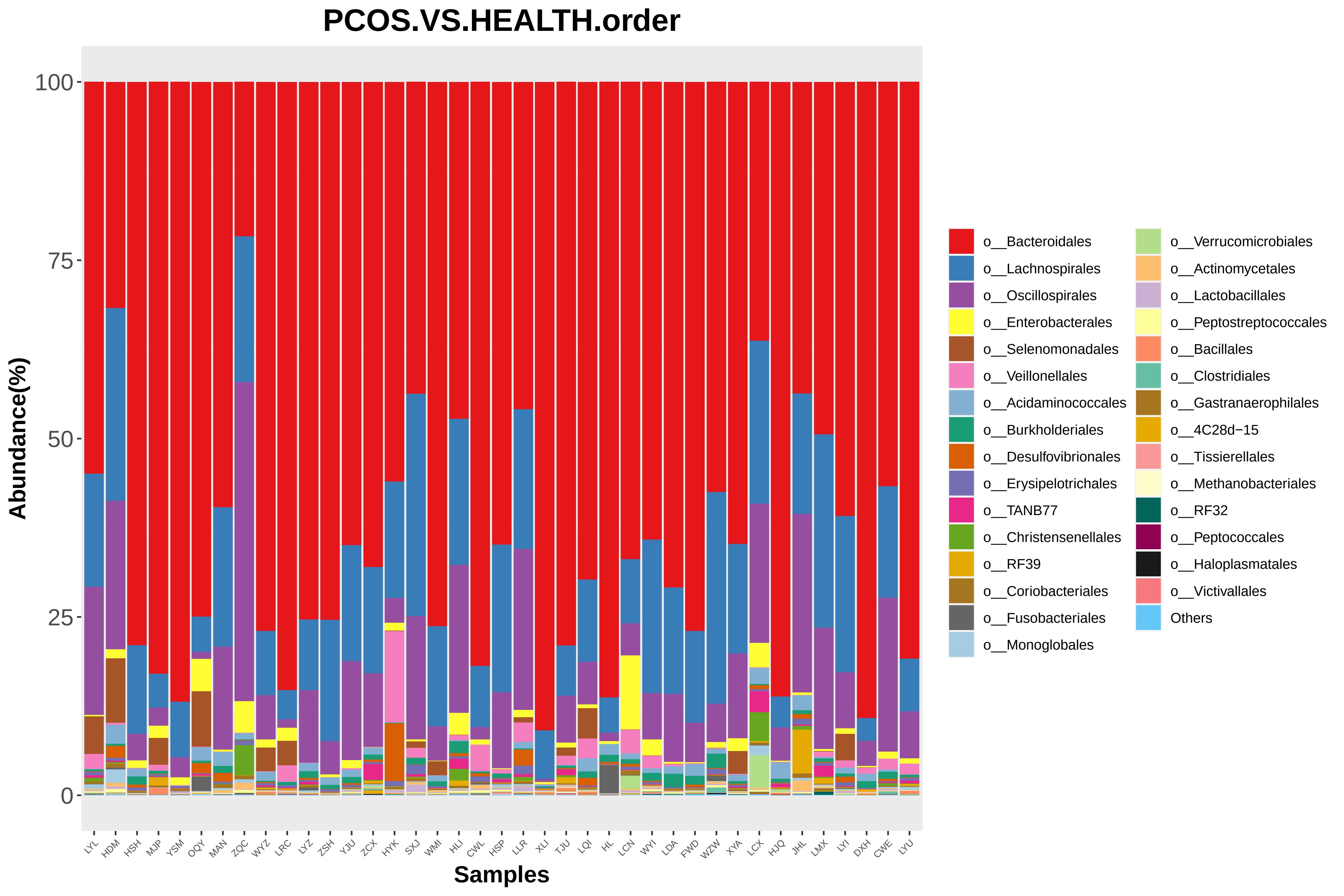
PCOS.VS.HEALTH.order
Abundance(%)
100
75
50
25
0
Samples
LYL HDM HSH MJP YSM OQY MAN ZQC WYZ LRC LYZ ZSH YJU ZCX HYK SXJ WMI HLI CWL HSP LLR XLI TJU LQI HL LCN WYI LDA FWD WZW XYA LCX HJQ JHL LMX LYI DXH CWE LYU
o__Bacteroidales
o__Lachnospirales
o__Oscillospirales
o__Enterobacterales
o__Selenomonadales
o__Veillonellales
o__Acidaminococcales
o__Burkholderiales
o__Desulfovibrionales
o__Erysipelotrichales
o__TANB77
o__Christensenellales
o__RF39
o__Coriobacteriales
o__Fusobacteriales
o__Monoglobales
o__Verrucomicrobiales
o__Actinomycetales
o__Lactobacillales
o__Peptostreptococcales
o__Bacillales
o__Clostridiales
o__Gastranaerophilales
o__4C28d−15
o__Tissierellales
o__Methanobacteriales
o__RF32
o__Peptococcales
o__Haloplasmatales
o__Victivallales
Others

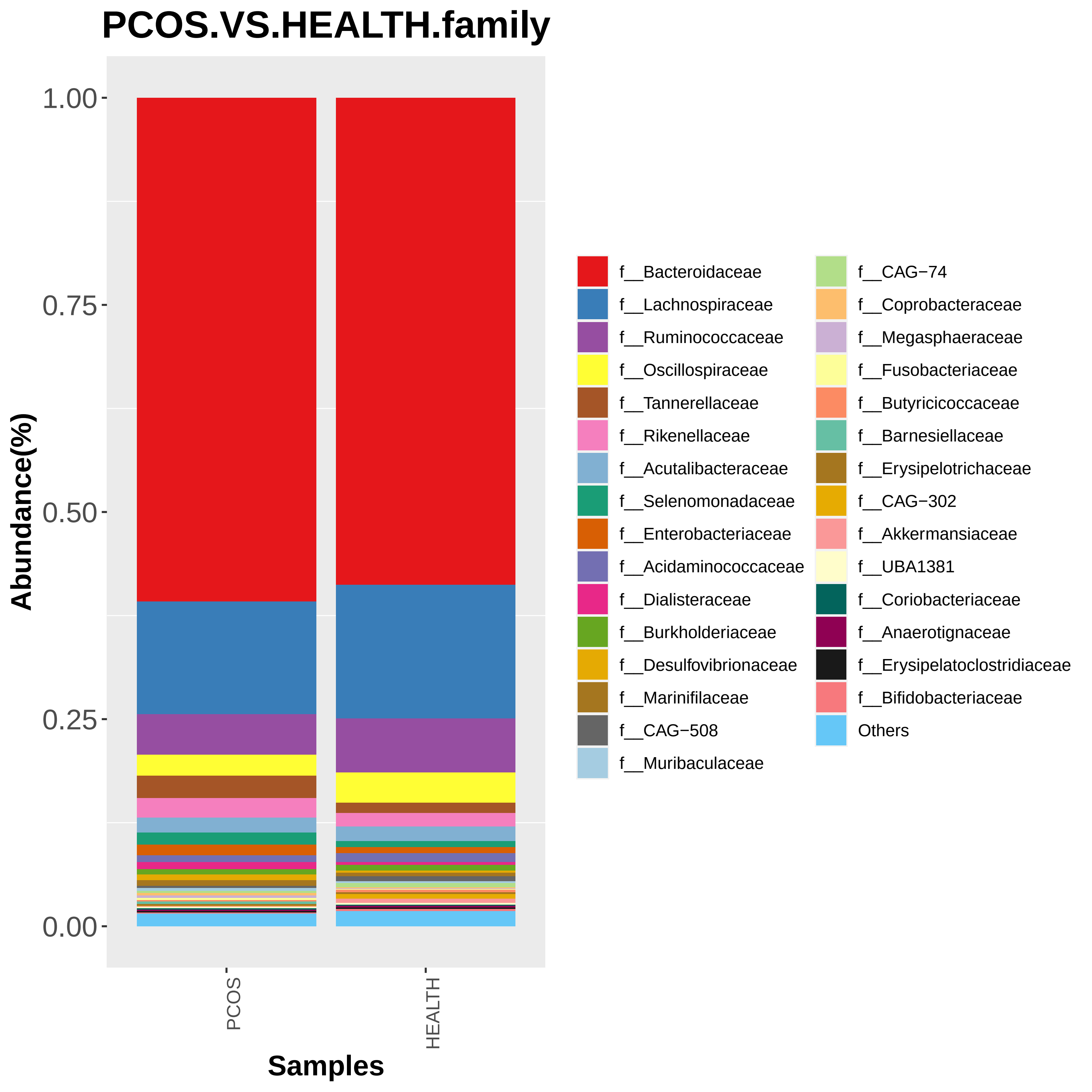

PCOS.VS.HEALTH.family
Abundance(%)
1.00
0.75
0.50
0.25
0.00
PCOS
HEALTH
Samples
f__Bacteroidaceae
f__Lachnospiraceae
f__Ruminococcaceae
f__Oscillospiraceae
f__Tannerellaceae
f__Rikenellaceae
f__Acutalibacteraceae
f__Selenomonadaceae
f__Enterobacteriaceae
f__Acidaminococcaceae
f__Dialisteraceae
f__Burkholderiaceae
f__Desulfovibrionaceae
f__Marinifilaceae
f__CAG−508
f__Muribaculaceae
f__CAG−74
f__Coprobacteraceae
f__Megasphaeraceae
f__Fusobacteriaceae
f__Butyricicoccaceae
f__Barnesiellaceae
f__Erysipelotrichaceae
f__CAG−302
f__Akkermansiaceae
f__UBA1381
f__Coriobacteriaceae
f__Anaerotignaceae
f__Erysipelatoclostridiaceae
f__Bifidobacteriaceae
Others

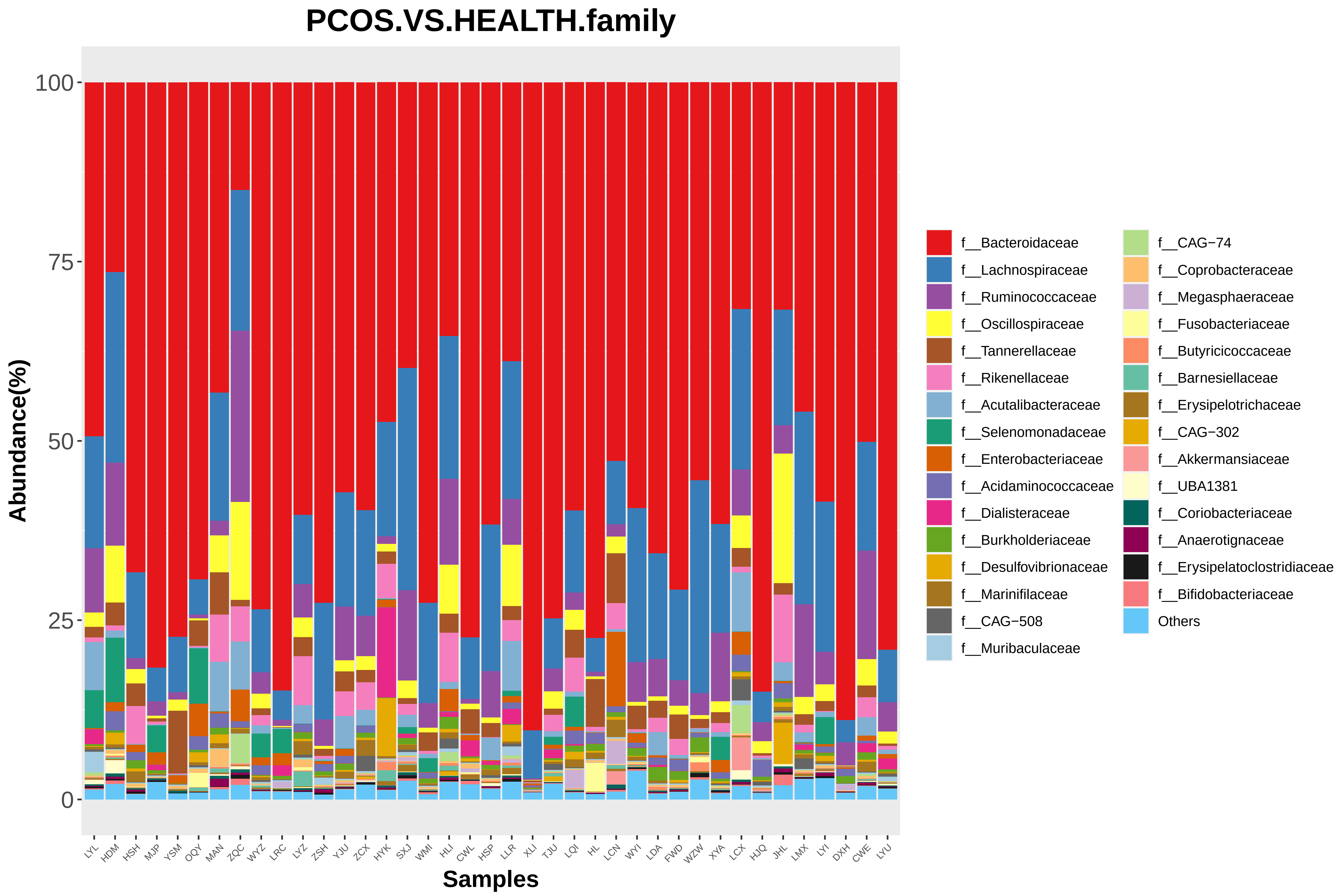

PCOS.VS.HEALTH.family
Abundance(%)
100
75
50
25
0
Samples
LYL
HDM
HSH
MJP
YSM
OQY
MAN
ZQC
WYZ
LRC
LYZ
ZSH
YJU
ZCX
HYK
SXJ
WMI
HLI
CWL
HSP
LLR
XLI
TJU
LQI
HL
LCN
WYI
LDA
FWD
WZW
XYA
LCX
HJQ
JHL
LMX
LYI
DXH
CWE
LYU
f__Bacteroidaceae
f__Lachnospiraceae
f__Ruminococcaceae
f__Oscillospiraceae
f__Tannerellaceae
f__Rikenellaceae
f__Acutalibacteraceae
f__Selenomonadaceae
f__Enterobacteriaceae
f__Acidaminococcaceae
f__Dialisteraceae
f__Burkholderiaceae
f__Desulfovibrionaceae
f__Marinifilaceae
f__CAG−508
f__Muribaculaceae
f__CAG−74
f__Coprobacteraceae
f__Megasphaeraceae
f__Fusobacteriaceae
f__Butyricicoccaceae
f__Barnesiellaceae
f__Erysipelotrichaceae
f__CAG−302
f__Akkermansiaceae
f__UBA1381
f__Coriobacteriaceae
f__Anaerotignaceae
f__Erysipelatoclostridiaceae
f__Bifidobacteriaceae
Others

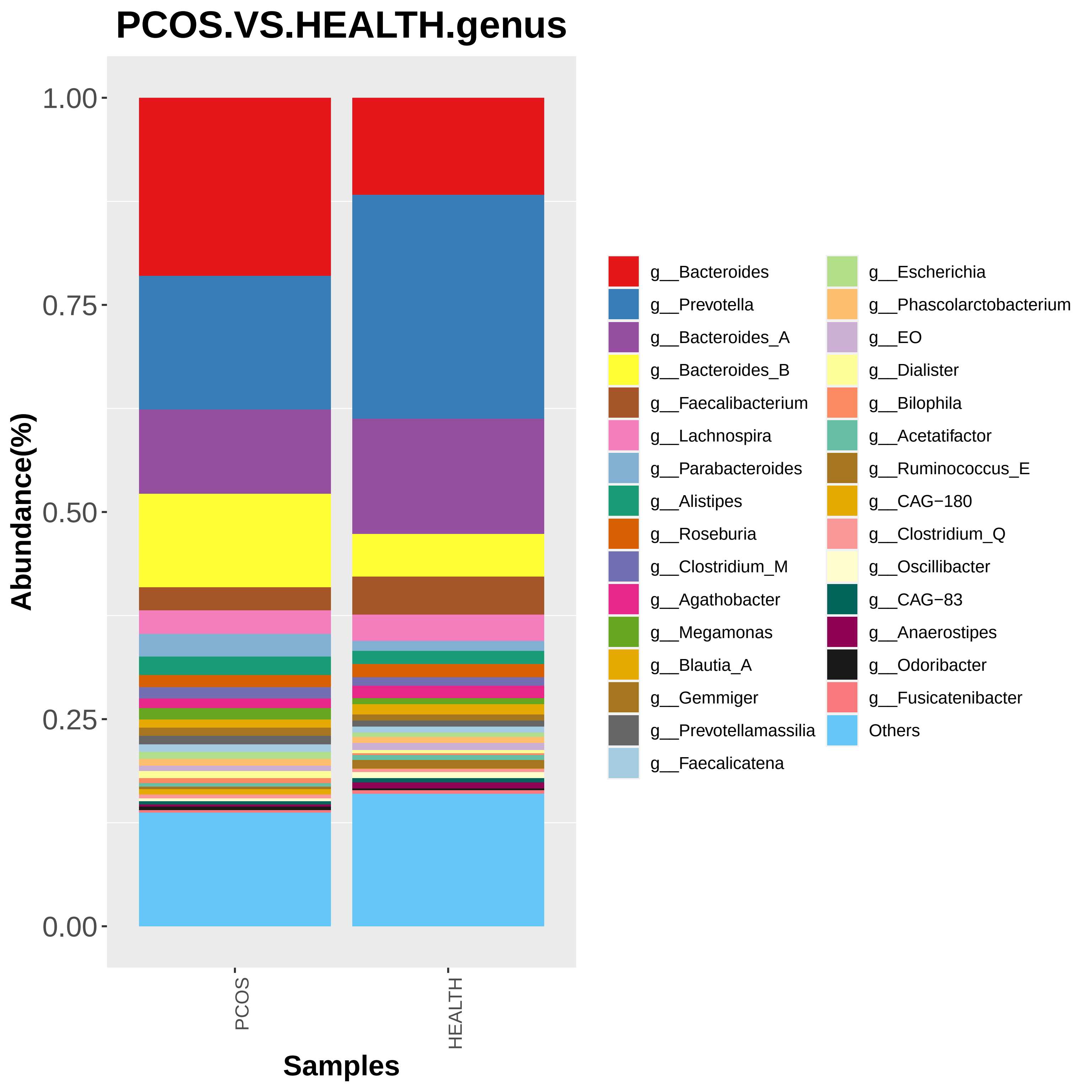

PCOS.VS.HEALTH.genus
Abundance(%)
1.00
0.75
0.50
0.25
0.00
PCOS
HEALTH
Samples
g__Bacteroides
g__Prevotella
g__Bacteroides_A
g__Bacteroides_B
g__Faecalibacterium
g__Lachnospira
g__Parabacteroides
g__Alistipes
g__Roseburia
g__Clostridium_M
g__Agathobacter
g__Megamonas
g__Blautia_A
g__Gemmiger
g__Prevotellamassilia
g__Faecalicatena
g__Escherichia
g__Phascolarctobacterium
g__EO
g__Dialister
g__Bilophila
g__Acetatifactor
g__Ruminococcus_E
g__CAG−180
g__Clostridium_Q
g__Oscillibacter
g__CAG−83
g__Anaerostipes
g__Odoribacter
g__Fusicatenibacter
Others

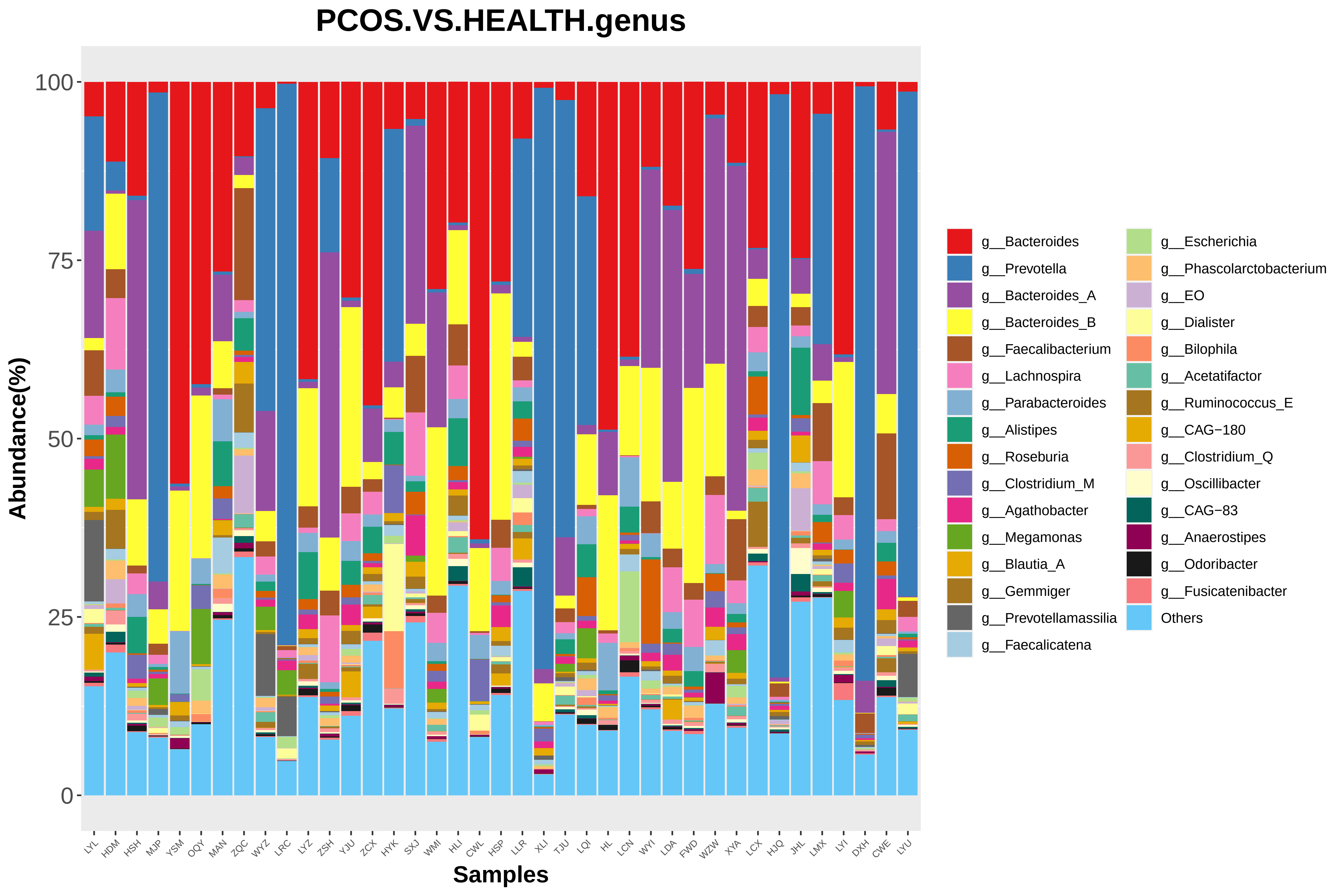
PCOS.VS.HEALTH.genus
Abundance(%)
100
75
50
25
0
Samples
LYL
HDM
HSH
MJP
YSM
OQY
MAN
ZQC
WYZ
LRC
LYZ
ZSH
YJU
ZCX
HYK
SXJ
WMI
HLI
CWL
HSP
LLR
XLI
TJU
LQI
HL
LCN
WYI
LDA
FWD
WZW
XYA
LCX
HJQ
JHL
LMX
LYI
DXH
CWE
LYU
g__Bacteroides
g__Prevotella
g__Bacteroides_A
g__Bacteroides_B
g__Faecalibacterium
g__Lachnospira
g__Parabacteroides
g__Alistipes
g__Roseburia
g__Clostridium_M
g__Agathobacter
g__Megamonas
g__Blautia_A
g__Gemmiger
g__Prevotellamassilia
g__Faecalicatena
g__Escherichia
g__Phascolarctobacterium
g__EO
g__Dialister
g__Bilophila
g__Acetatifactor
g__Ruminococcus_E
g__CAG−180
g__Clostridium_Q
g__Oscillibacter
g__CAG−83
g__Anaerostipes
g__Odoribacter
g__Fusicatenibacter
Others

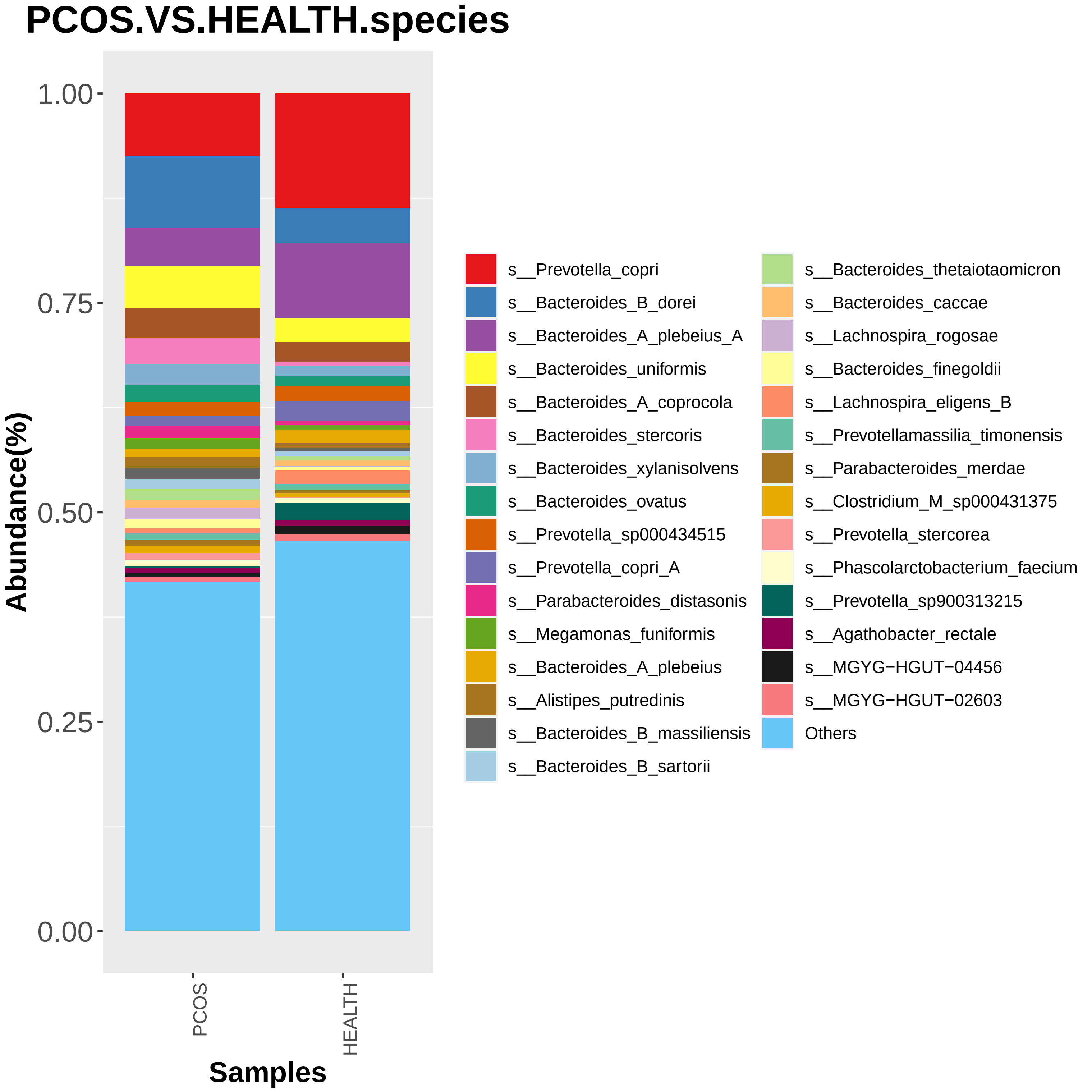
PCOS.VS.HEALTH.species
Abundance(%)
1.00
0.75
0.50
0.25
0.00
PCOS
HEALTH
Samples
s__Prevotella_copri
s__Bacteroides_B_dorei
s__Bacteroides_A_plebeius_A
s__Bacteroides_uniformis
s__Bacteroides_A_coprocola
s__Bacteroides_stercoris
s__Bacteroides_xylanisolvens
s__Bacteroides_ovatus
s__Prevotella_sp000434515
s__Prevotella_copri_A
s__Parabacteroides_distasonis
s__Megamonas_funiformis
s__Bacteroides_A_plebeius
s__Alistipes_putredinis
s__Bacteroides_B_massiliensis
s__Bacteroides_B_sartorii
s__Bacteroides_thetaiotaomicron
s__Bacteroides_caccae
s__Lachnospira_rogosae
s__Bacteroides_finegoldii
s__Lachnospira_eligens_B
s__Prevotellamassilia_timonensis
s__Parabacteroides_merdae
s__Clostridium_M_sp000431375
s__Prevotella_stercorea
s__Phascolarctobacterium_faecium
s__Prevotella_sp900313215
s__Agathobacter_rectale
s__MGYG−HGUT−04456
s__MGYG−HGUT−02603
Others

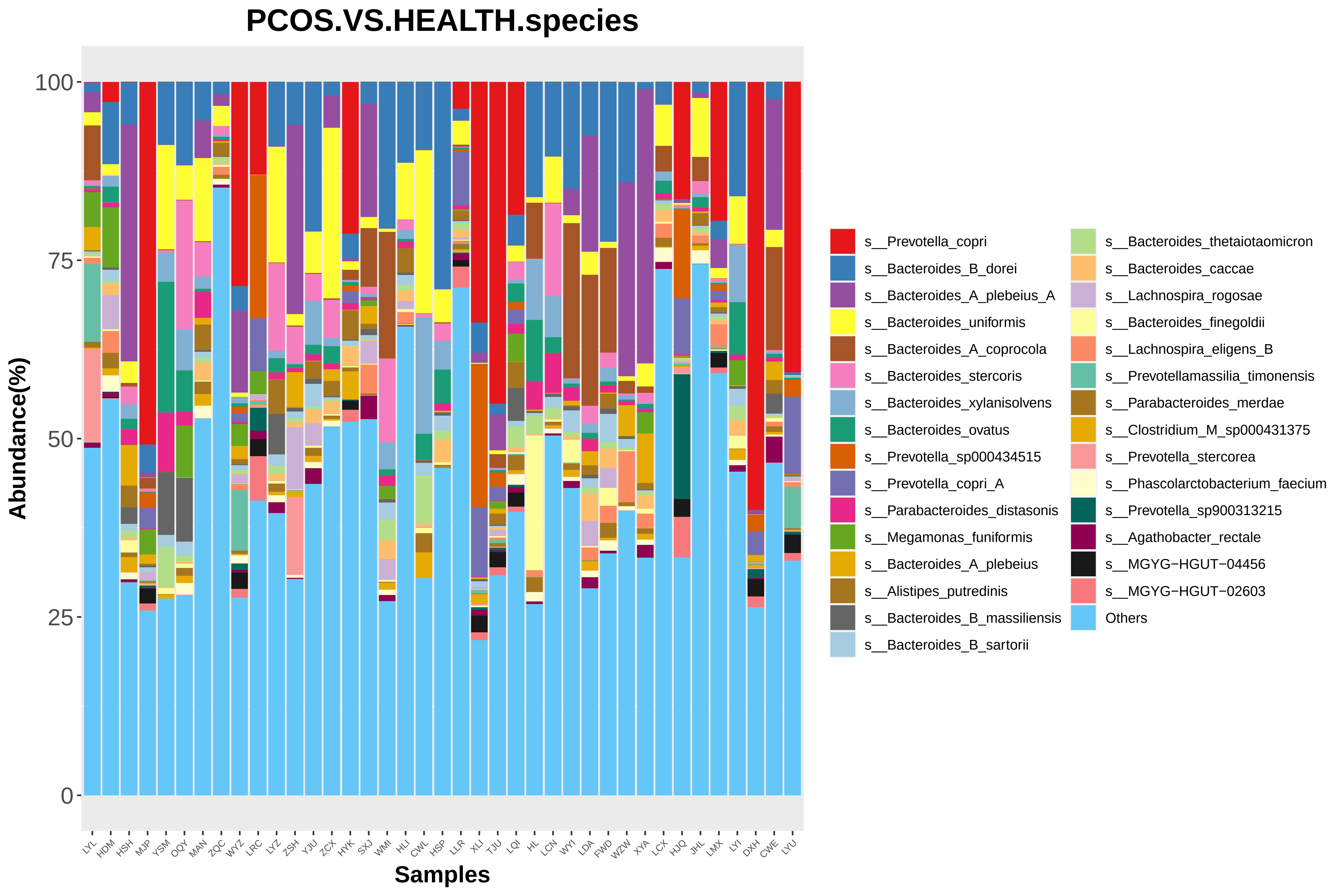

PCOS.VS.HEALTH.species
Abundance(%)
100
75
50
25
0
Samples
LYL
HDM
HSH
MJP
YSM
OQY
MAN
ZQC
WYZ
LRC
LYZ
ZSH
YJU
ZCX
HYK
SXJ
WMI
HLI
CWL
HSP
LLR
XLI
TJU
LQI
HL
LCN
WYI
LDA
FWD
WZW
XYA
LCX
HJQ
JHL
LMX
LYI
DXH
CWE
LYU
s__Prevotella_copri
s__Bacteroides_B_dorei
s__Bacteroides_A_plebeius_A
s__Bacteroides_uniformis
s__Bacteroides_A_coprocola
s__Bacteroides_stercoris
s__Bacteroides_xylanisolvens
s__Bacteroides_ovatus
s__Prevotella_sp000434515
s__Prevotella_copri_A
s__Parabacteroides_distasonis
s__Megamonas_funiformis
s__Bacteroides_A_plebeius
s__Alistipes_putredinis
s__Bacteroides_B_massiliensis
s__Bacteroides_B_sartorii
s__Bacteroides_thetaiotaomicron
s__Bacteroides_caccae
s__Lachnospira_rogosae
s__Bacteroides_finegoldii
s__Lachnospira_eligens_B
s__Prevotellamassilia_timonensis
s__Parabacteroides_merdae
s__Clostridium_M_sp000431375
s__Prevotella_stercorea
s__Phascolarctobacterium_faecium
s__Prevotella_sp900313215
s__Agathobacter_rectale
s__MGYG−HGUT−04456
s__MGYG−HGUT−02603
Others

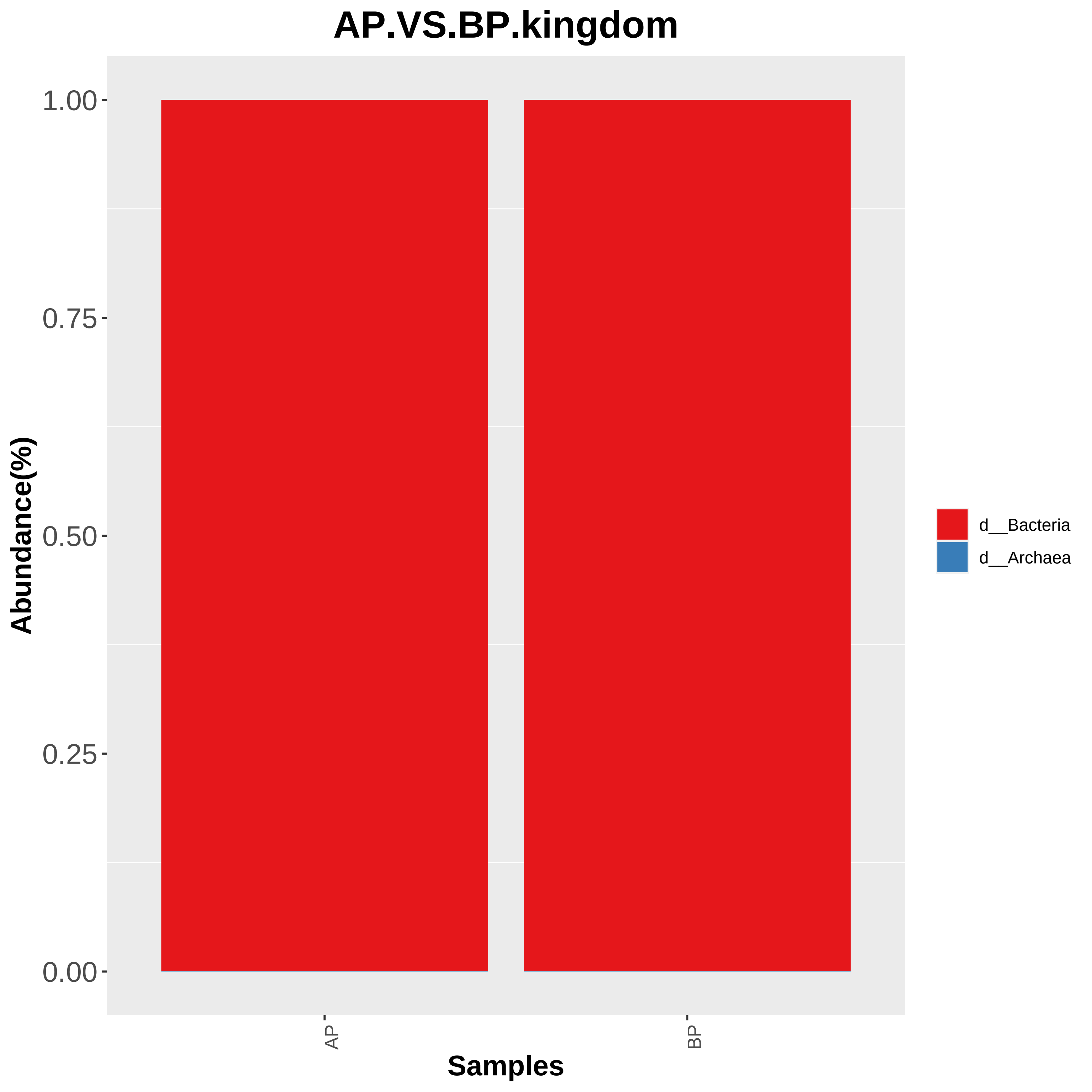

AP.VS.BP.kingdom
Abundance(%)
1.00
0.75
0.50
0.25
0.00
AP
BP
Samples
d__Bacteria
d__Archaea

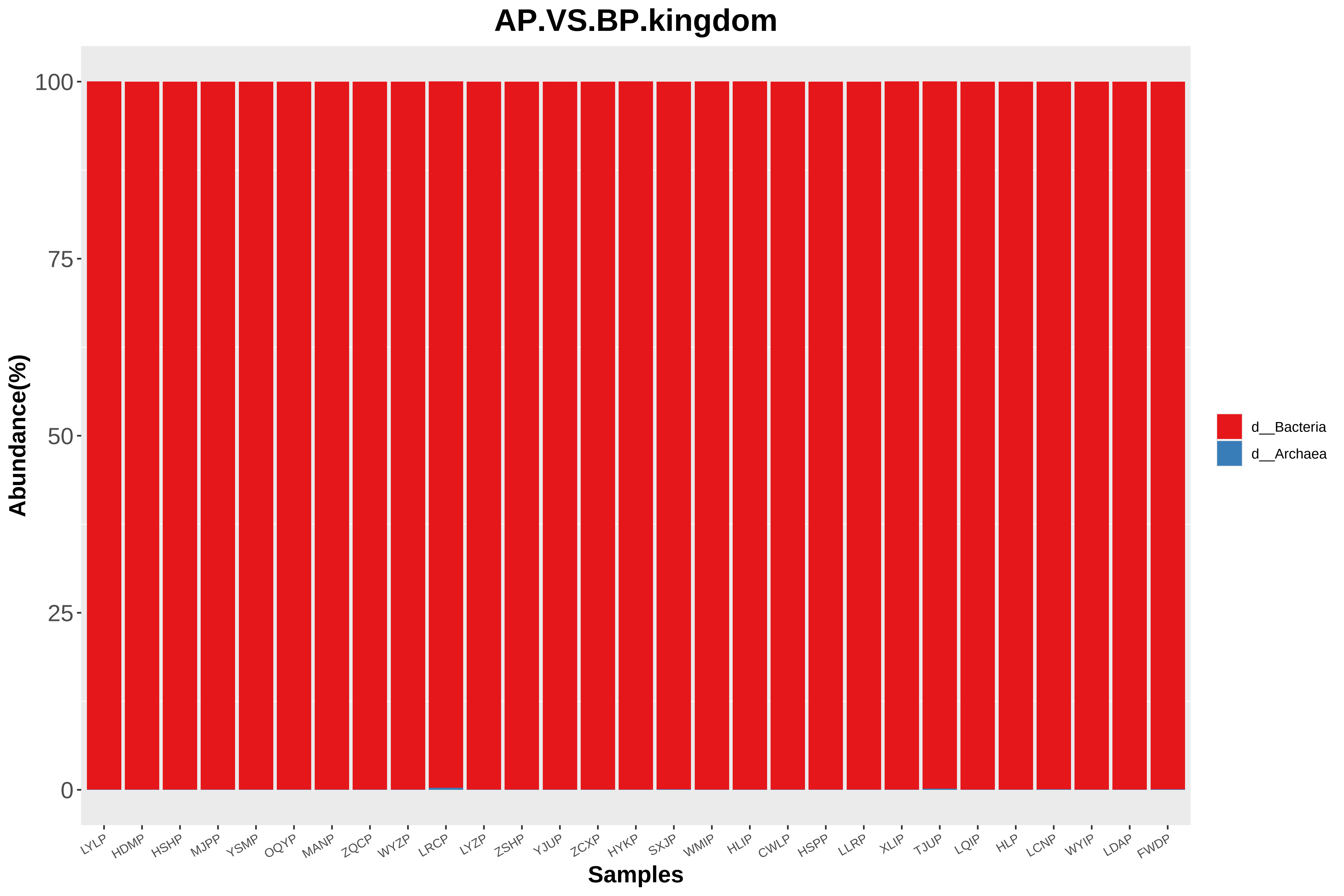

AP.VS.BP.kingdom
Abundance(%)
100
75
50
25
0
LYLP
HDMP
HSHP
MJPP
YSMP
OQYP
MANP
ZQCP
WYZP
LRCP
LYZP
ZSHP
YJUP
ZCXP
HYKP
SXJP
WMIP
HLIP
CWLP
HSPP
LLRP
XLIP
TJUP
LQIP
HLP
LCNP
WYIP
LDAP
FWDP
Samples
d__Bacteria
d__Archaea

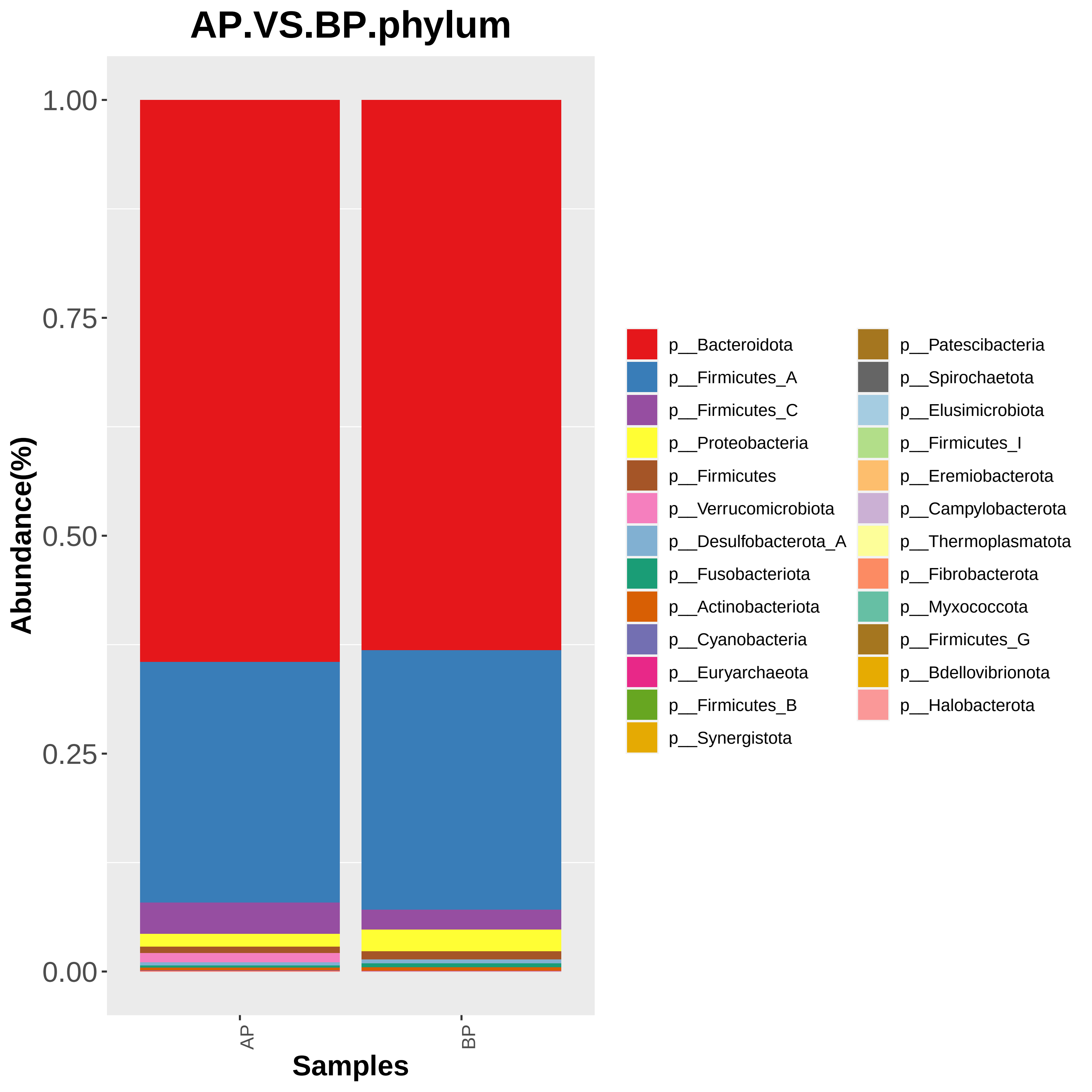
AP.VS.BP.phylum
Abundance(%)
1.00
0.75
0.50
0.25
0.00
AP
BP
Samples
p__Bacteroidota
p__Firmicutes_A
p__Firmicutes_C
p__Proteobacteria
p__Firmicutes
p__Verrucomicrobiota
p__Desulfobacterota_A
p__Fusobacteriota
p__Actinobacteriota
p__Cyanobacteria
p__Euryarchaeota
p__Firmicutes_B
p__Synergistota
p__Patescibacteria
p__Spirochaetota
p__Elusimicrobiota
p__Firmicutes_I
p__Eremiobacterota
p__Campylobacterota
p__Thermoplasmatota
p__Fibrobacterota
p__Myxococcota
p__Firmicutes_G
p__Bdellovibrionota
p__Halobacterota

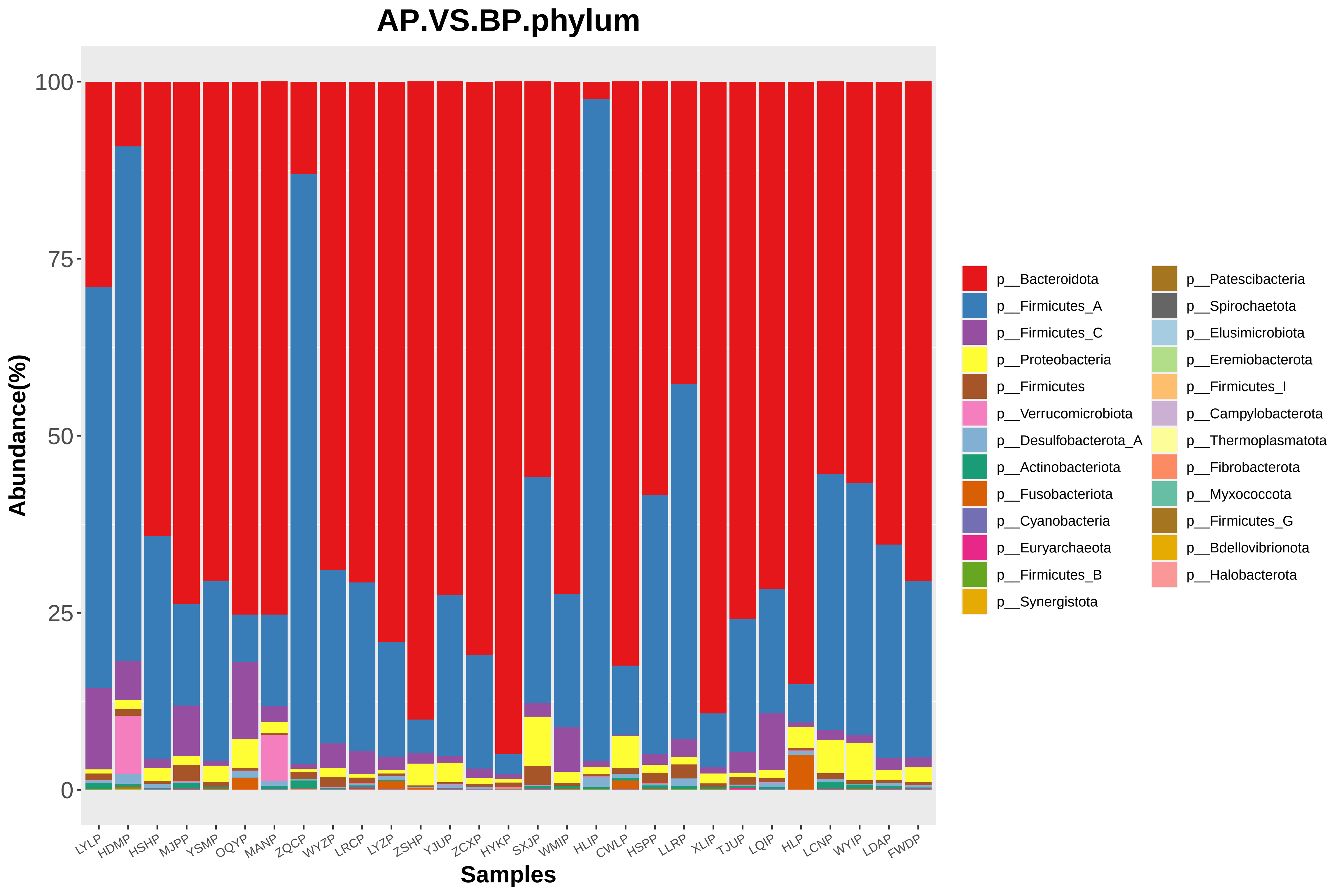

AP.VS.BP.phylum
Abundance(%)
100
75
50
25
0
LYLP
HDMP
HSHP
MJPP
YSMP
OQYP
MANP
ZQCP
WYZP
LRCP
LYZP
ZSHP
YJUP
ZCXP
HYKP
SXJP
WMIP
HLIP
CWLP
HSPP
LLRP
XLIP
TJUP
LQIP
HLP
LCNP
WYIP
LDAP
FWDP
Samples
p__Bacteroidota
p__Firmicutes_A
p__Firmicutes_C
p__Proteobacteria
p__Firmicutes
p__Verrucomicrobiota
p__Desulfobacterota_A
p__Actinobacteriota
p__Fusobacteriota
p__Cyanobacteria
p__Euryarchaeota
p__Firmicutes_B
p__Synergistota
p__Patescibacteria
p__Spirochaetota
p__Elusimicrobiota
p__Eremiobacterota
p__Firmicutes_I
p__Campylobacterota
p__Thermoplasmatota
p__Fibrobacterota
p__Myxococcota
p__Firmicutes_G
p__Bdellovibrionota
p__Halobacterota

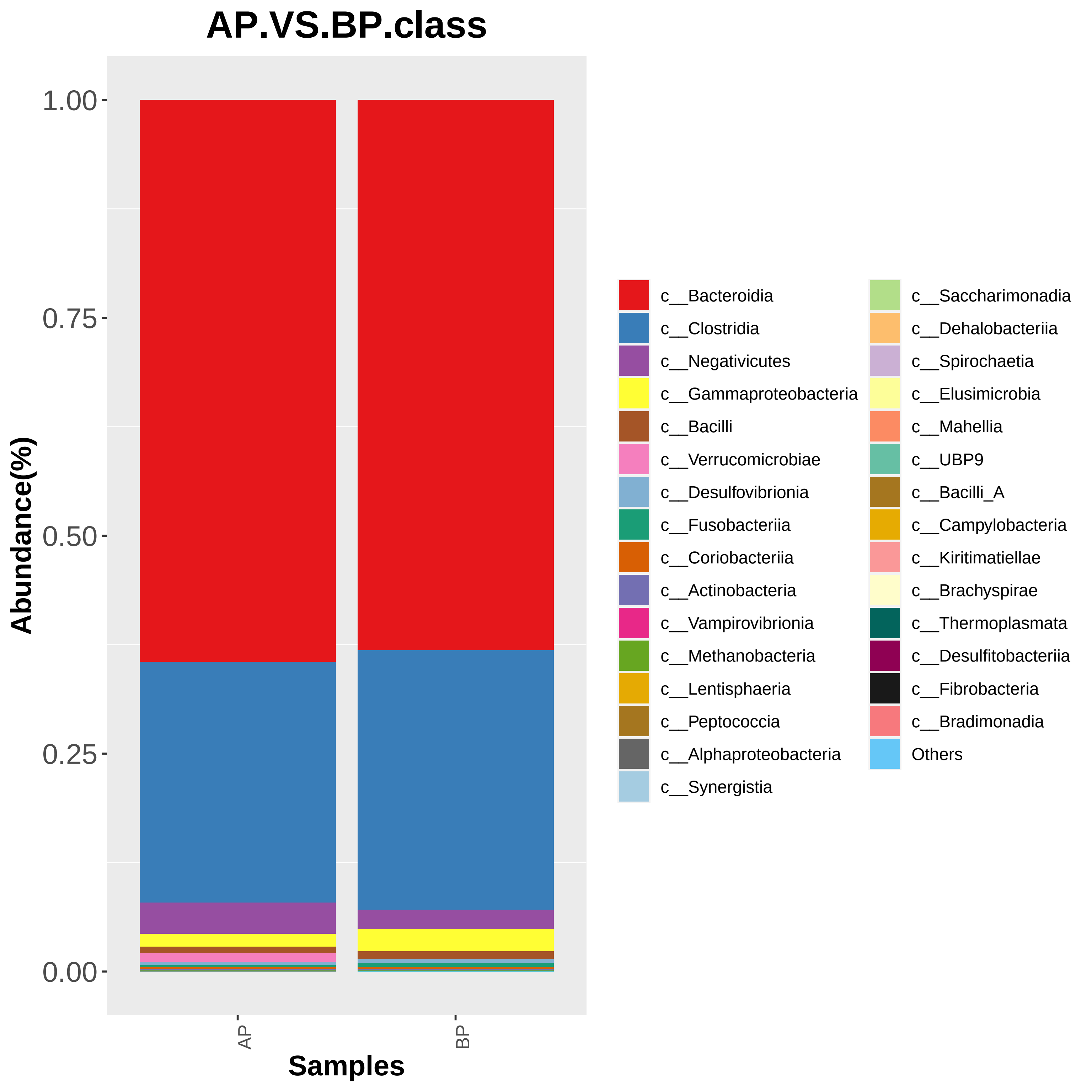
AP.VS.BP.class
Abundance(%)
1.00
0.75
0.50
0.25
0.00
AP
BP
Samples
c__Bacteroidia
c__Clostridia
c__Negativicutes
c__Gammaproteobacteria
c__Bacilli
c__Verrucomicrobiae
c__Desulfovibrionia
c__Fusobacteriia
c__Coriobacteriia
c__Actinobacteria
c__Vampirovibrionia
c__Methanobacteria
c__Lentisphaeria
c__Peptococcia
c__Alphaproteobacteria
c__Synergistia
c__Saccharimonadia
c__Dehalobacteriia
c__Spirochaetia
c__Elusimicrobia
c__Mahellia
c__UBP9
c__Bacilli_A
c__Campylobacteria
c__Kiritimatiellae
c__Brachyspirae
c__Thermoplasmata
c__Desulfitobacteriia
c__Fibrobacteria
c__Bradimonadia
Others

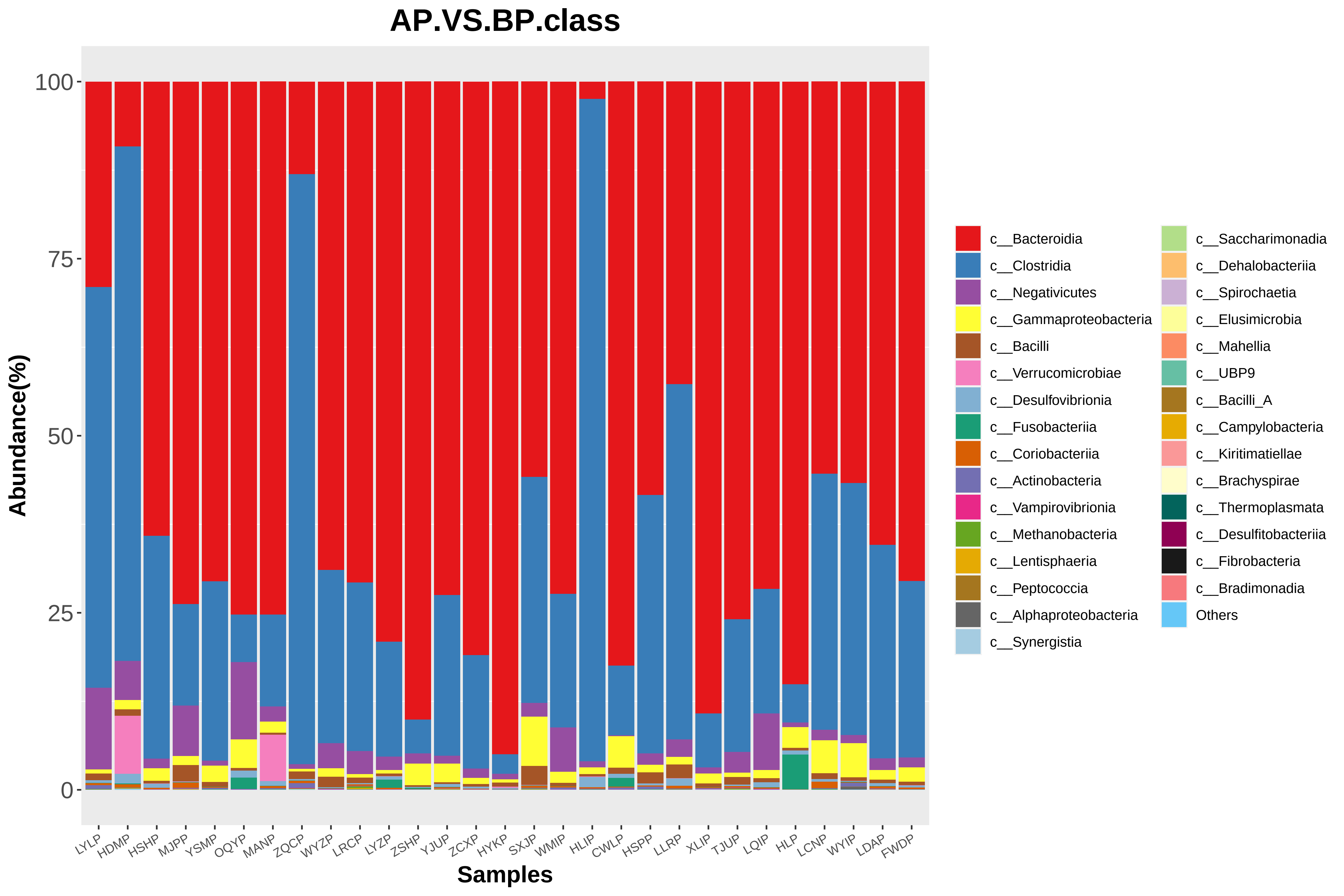
AP.VS.BP.class
Abundance(%)
100
75
50
25
0
LYLP
HDMP
HSHP
MJPP
YSMP
OQYP
MANP
ZQCP
WYZP
LRCP
LYZP
ZSHP
YJUP
ZCXP
HYKP
SXJP
WMIP
HLIP
CWLP
HSPP
LLRP
XLIP
TJUP
LQIP
HLP
LCNP
WYIP
LDAP
FWDP
Samples
c__Bacteroidia
c__Clostridia
c__Negativicutes
c__Gammaproteobacteria
c__Bacilli
c__Verrucomicrobiae
c__Desulfovibrionia
c__Fusobacteriia
c__Coriobacteriia
c__Actinobacteria
c__Vampirovibrionia
c__Methanobacteria
c__Lentisphaeria
c__Peptococcia
c__Alphaproteobacteria
c__Synergistia
c__Saccharimonadia
c__Dehalobacteriia
c__Spirochaetia
c__Elusimicrobia
c__Mahellia
c__UBP9
c__Bacilli_A
c__Campylobacteria
c__Kiritimatiellae
c__Brachyspirae
c__Thermoplasmata
c__Desulfitobacteriia
c__Fibrobacteria
c__Bradimonadia
Others

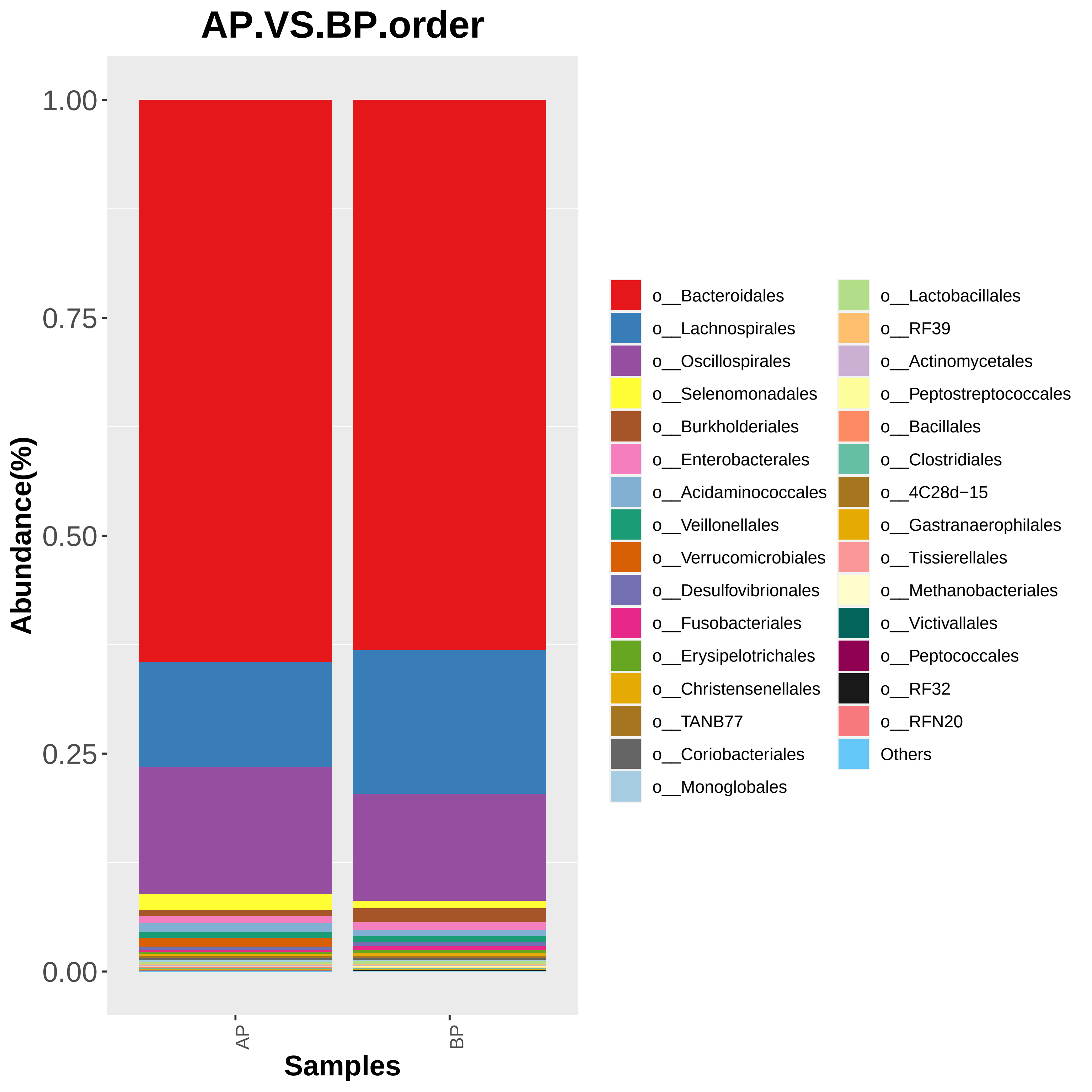

AP.VS.BP.order
Abundance(%)
1.00
0.75
0.50
0.25
0.00
AP
BP
Samples
o__Bacteroidales
o__Lachnospirales
o__Oscillospirales
o__Selenomonadales
o__Burkholderiales
o__Enterobacterales
o__Acidaminococcales
o__Veillonellales
o__Verrucomicrobiales
o__Desulfovibrionales
o__Fusobacteriales
o__Erysipelotrichales
o__Christensenellales
o__TANB77
o__Coriobacteriales
o__Monoglobales
o__Lactobacillales
o__RF39
o__Actinomycetales
o__Peptostreptococcales
o__Bacillales
o__Clostridiales
o__4C28d−15
o__Gastranaerophilales
o__Tissierellales
o__Methanobacteriales
o__Victivallales
o__Peptococcales
o__RF32
o__RFN20
Others

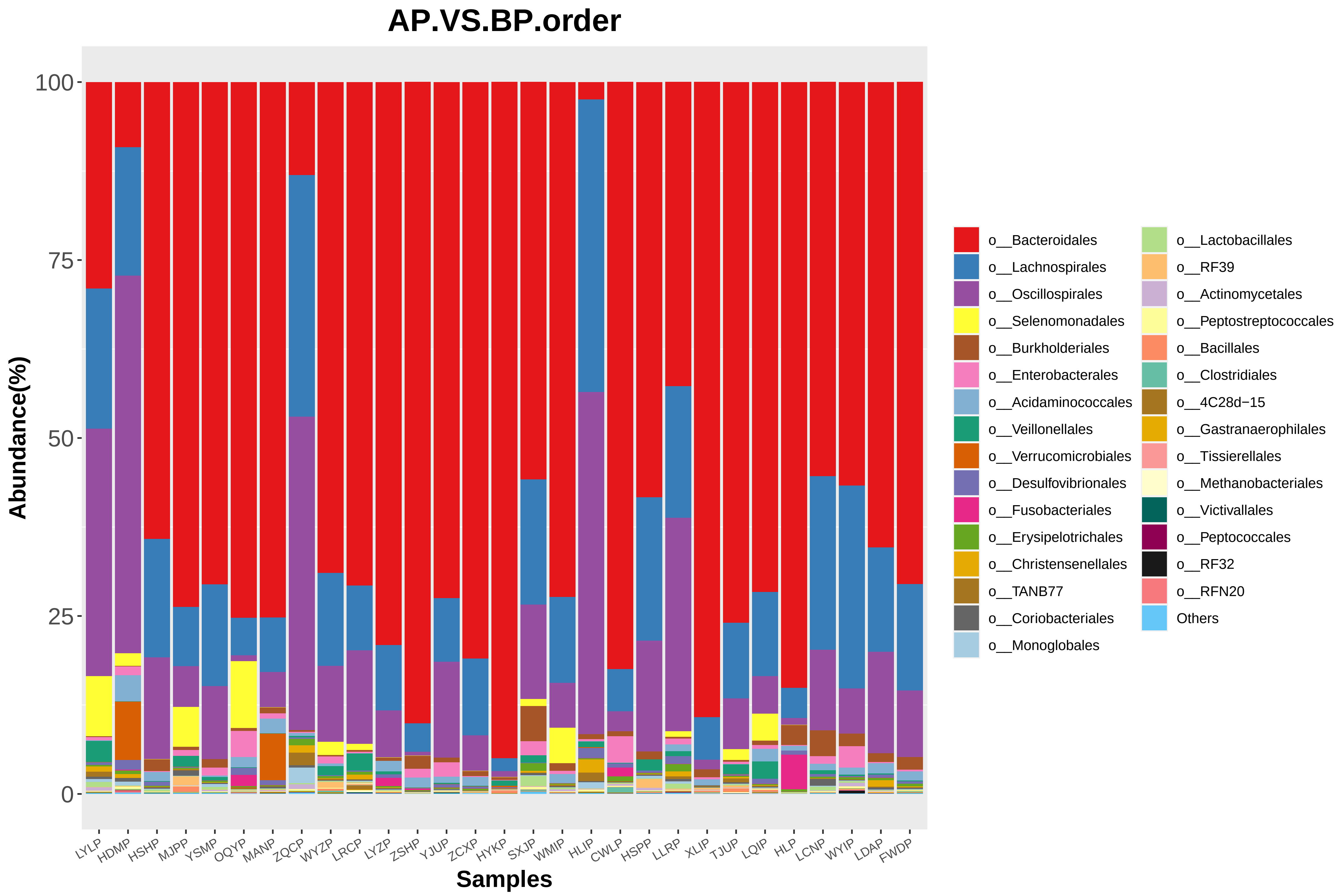
AP.VS.BP.order
Abundance(%)
100
75
50
25
0
LYLP
HDMP
HSHP
MJPP
YSMP
OQYP
MANP
ZQCP
WYZP
LRCP
LYZP
ZSHP
YJUP
ZCXP
HYKP
SXJP
WMIP
HLIP
CWLP
HSPP
LLRP
XLIP
TJUP
LQIP
HLP
LCNP
WYIP
LDAP
FWDP
Samples
o__Bacteroidales
o__Lachnospirales
o__Oscillospirales
o__Selenomonadales
o__Burkholderiales
o__Enterobacterales
o__Acidaminococcales
o__Veillonellales
o__Verrucomicrobiales
o__Desulfovibrionales
o__Fusobacteriales
o__Erysipelotrichales
o__Christensenellales
o__TANB77
o__Coriobacteriales
o__Monoglobales
o__Lactobacillales
o__RF39
o__Actinomycetales
o__Peptostreptococcales
o__Bacillales
o__Clostridiales
o__4C28d−15
o__Gastranaerophilales
o__Tissierellales
o__Methanobacteriales
o__Victivallales
o__Peptococcales
o__RF32
o__RFN20
Others

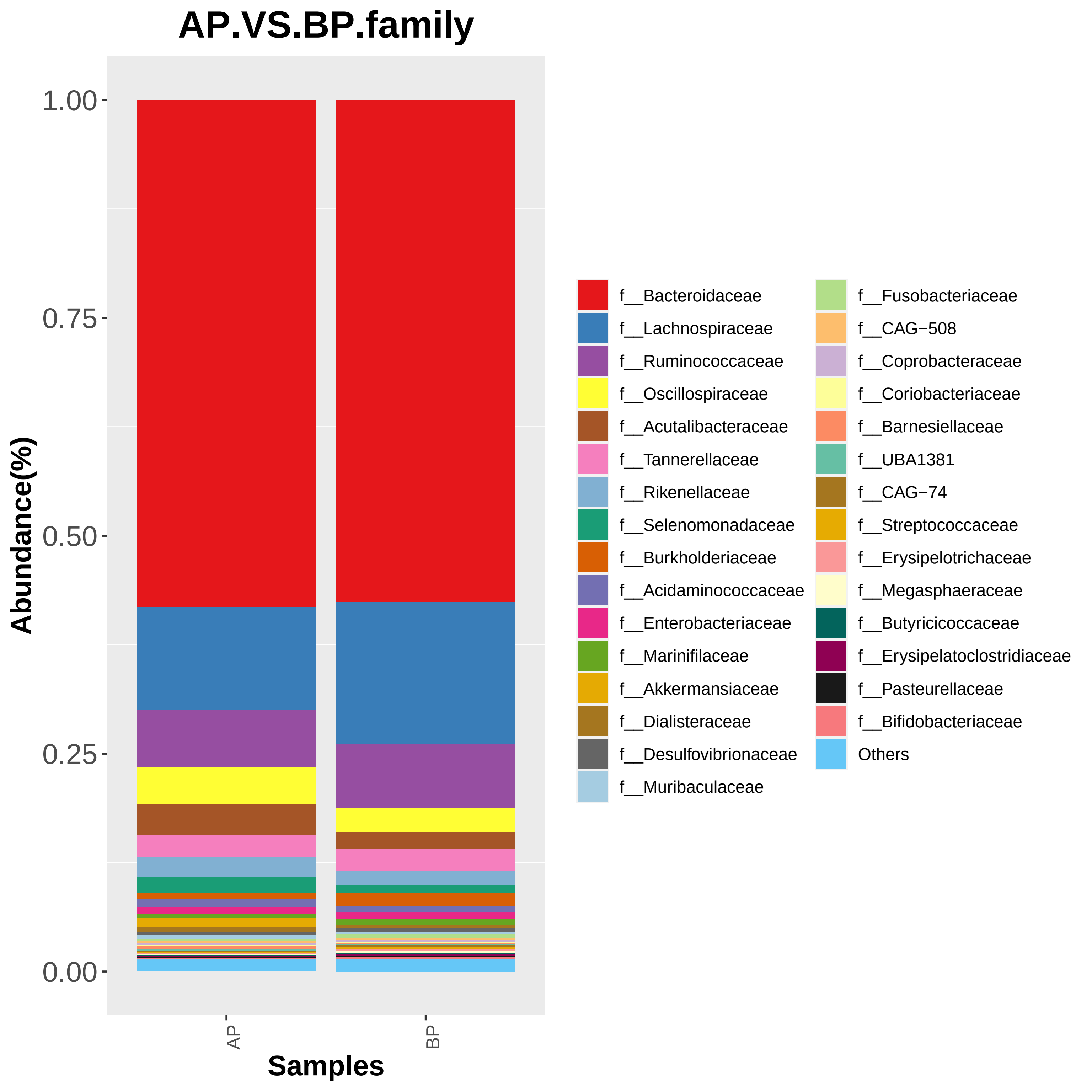
AP.VS.BP.family
Abundance(%)
1.00
0.75
0.50
0.25
0.00
AP
BP
Samples
f__Bacteroidaceae
f__Lachnospiraceae
f__Ruminococcaceae
f__Oscillospiraceae
f__Acutalibacteraceae
f__Tannerellaceae
f__Rikenellaceae
f__Selenomonadaceae
f__Burkholderiaceae
f__Acidaminococcaceae
f__Enterobacteriaceae
f__Marinifilaceae
f__Akkermansiaceae
f__Dialisteraceae
f__Desulfovibrionaceae
f__Muribaculaceae
f__Fusobacteriaceae
f__CAG−508
f__Coprobacteraceae
f__Coriobacteriaceae
f__Barnesiellaceae
f__UBA1381
f__CAG−74
f__Streptococcaceae
f__Erysipelotrichaceae
f__Megasphaeraceae
f__Butyricicoccaceae
f__Erysipelatoclostridiaceae
f__Pasteurellaceae
f__Bifidobacteriaceae
Others

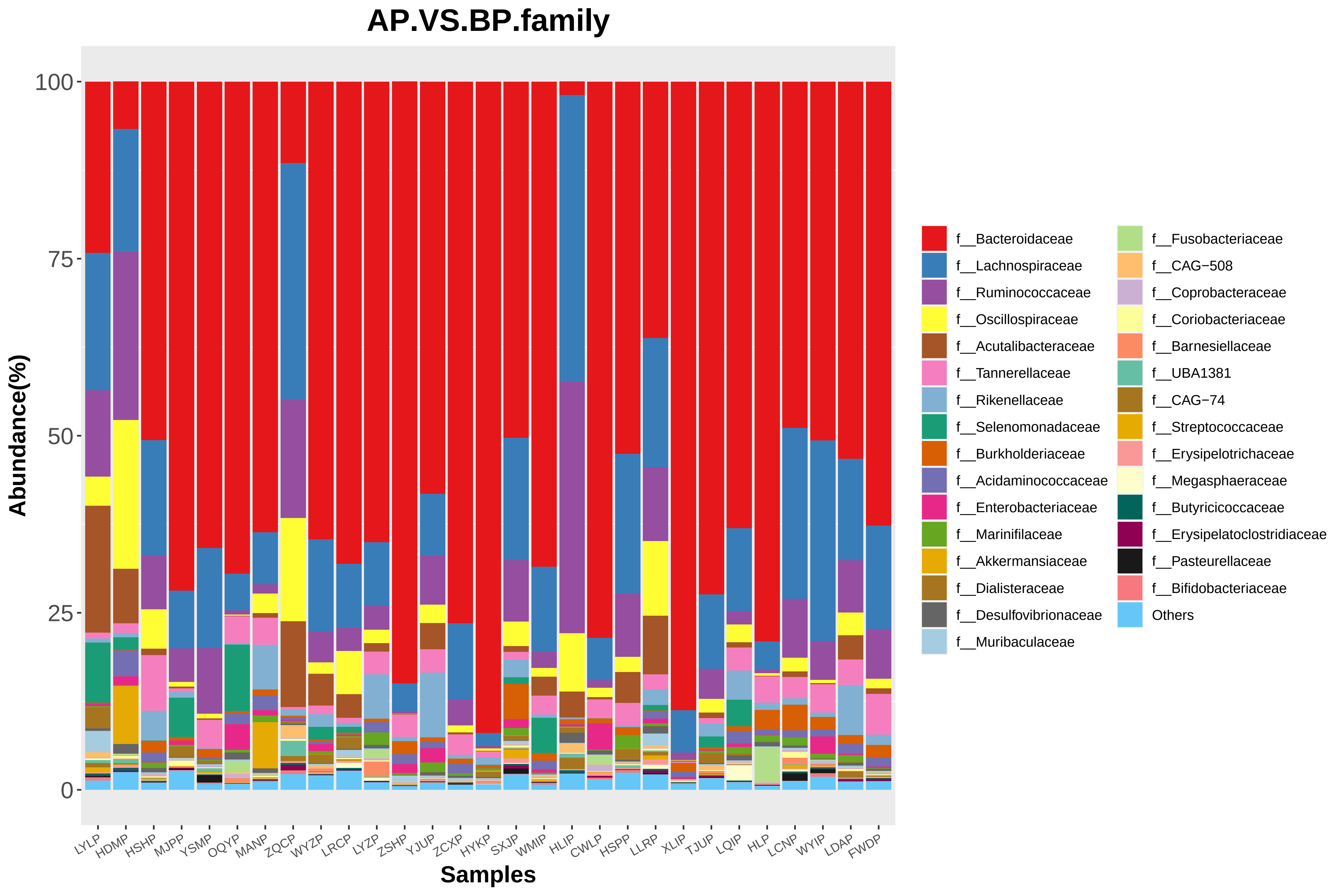
AP.VS.BP.family
Abundance(%)
100
75
50
25
0
LYLP
HDMP
HSHP
MJPP
YSMP
OQYP
MANP
ZQCP
WYZP
LRCP
LYZP
ZSHP
YJUP
ZCXP
HYKP
SXJP
WMIP
HLIP
CWLP
HSPP
LLRP
XLIP
TJUP
LQIP
HLP
LCNP
WYIP
LDAP
FWDP
Samples
f__Bacteroidaceae
f__Lachnospiraceae
f__Ruminococcaceae
f__Oscillospiraceae
f__Acutalibacteraceae
f__Tannerellaceae
f__Rikenellaceae
f__Selenomonadaceae
f__Burkholderiaceae
f__Acidaminococcaceae
f__Enterobacteriaceae
f__Marinifilaceae
f__Akkermansiaceae
f__Dialisteraceae
f__Desulfovibrionaceae
f__Muribaculaceae
f__Fusobacteriaceae
f__CAG−508
f__Coprobacteraceae
f__Coriobacteriaceae
f__Barnesiellaceae
f__UBA1381
f__CAG−74
f__Streptococcaceae
f__Erysipelotrichaceae
f__Megasphaeraceae
f__Butyricicoccaceae
f__Erysipelatoclostridiaceae
f__Pasteurellaceae
f__Bifidobacteriaceae
Others

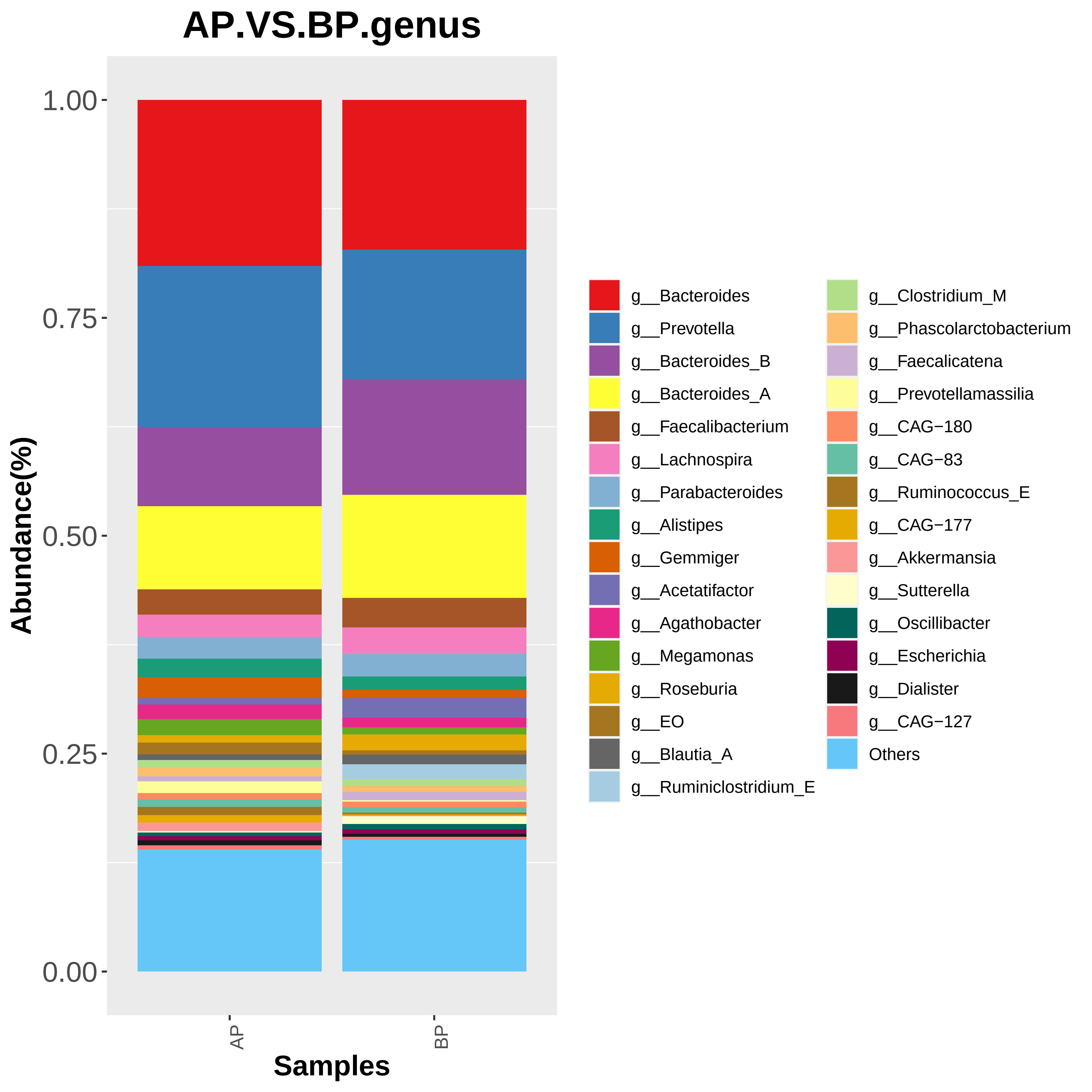
AP.VS.BP.genus
Abundance(%)
1.00
0.75
0.50
0.25
0.00
AP
BP
Samples
g__Bacteroides
g__Prevotella
g__Bacteroides_B
g__Bacteroides_A
g__Faecalibacterium
g__Lachnospira
g__Parabacteroides
g__Alistipes
g__Gemmiger
g__Acetatifactor
g__Agathobacter
g__Megamonas
g__Roseburia
g__EO
g__Blautia_A
g__Ruminiclostridium_E
g__Clostridium_M
g__Phascolarctobacterium
g__Faecalicatena
g__Prevotellamassilia
g__CAG−180
g__CAG−83
g__Ruminococcus_E
g__CAG−177
g__Akkermansia
g__Sutterella
g__Oscillibacter
g__Escherichia
g__Dialister
g__CAG−127
Others

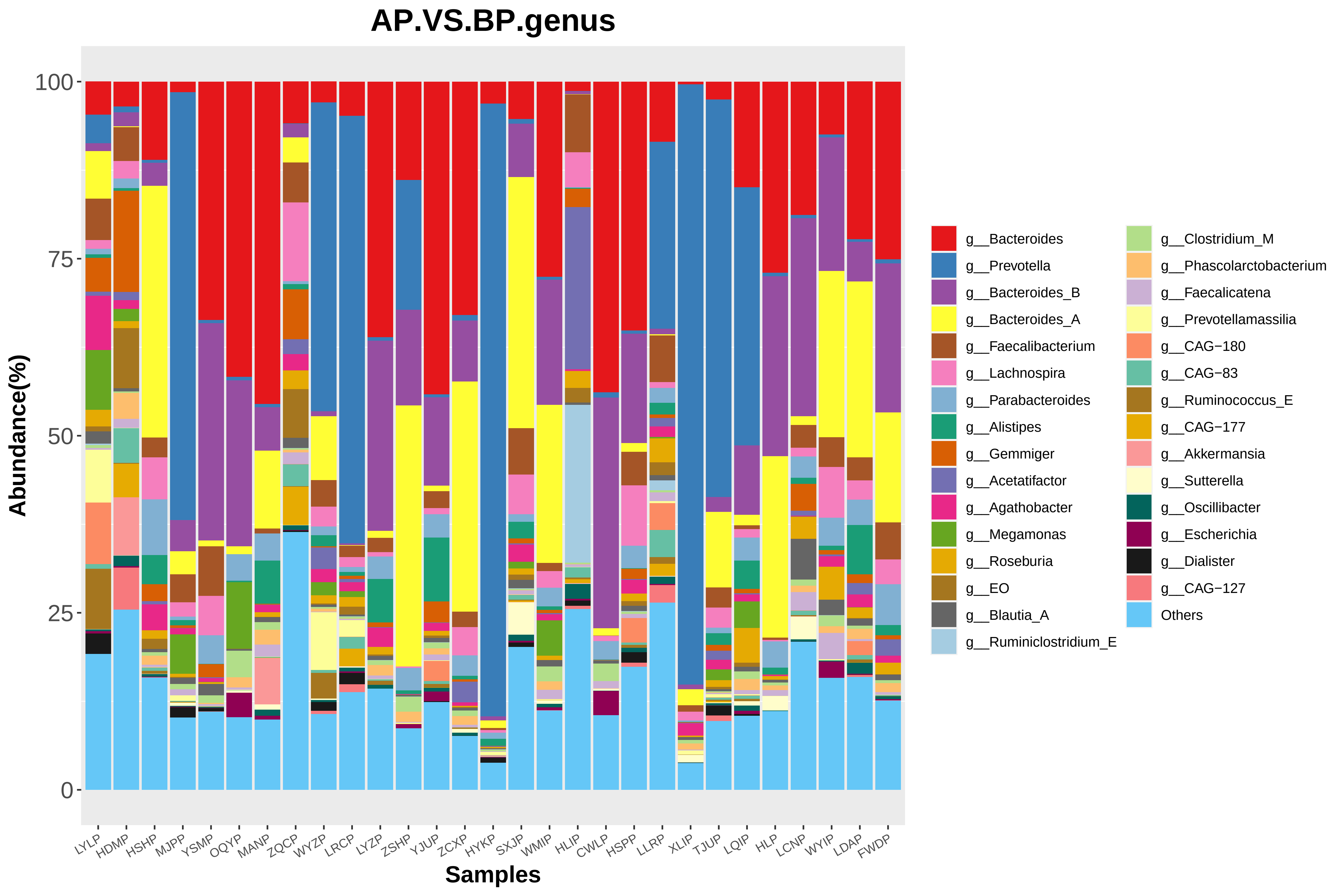

AP.VS.BP.genus
Abundance(%)
100
75
50
25
0
Samples
LYLP
HDMP
HSHP
MJPP
YSMP
OQYP
MANP
ZQCP
WYZP
LRCP
LYZP
ZSHP
YJUP
ZCXP
HYKP
SXJP
WMIP
HLIP
CWLP
HSPP
LLRP
XLIP
TJUP
LQIP
HLP
LCNP
WYIP
LDAP
FWDP
g__Bacteroides
g__Prevotella
g__Bacteroides_B
g__Bacteroides_A
g__Faecalibacterium
g__Lachnospira
g__Parabacteroides
g__Alistipes
g__Gemmiger
g__Acetatifactor
g__Agathobacter
g__Megamonas
g__Roseburia
g__EO
g__Blautia_A
g__Ruminiclostridium_E
g__Clostridium_M
g__Phascolarctobacterium
g__Faecalicatena
g__Prevotellamassilia
g__CAG−180
g__CAG−83
g__Ruminococcus_E
g__CAG−177
g__Akkermansia
g__Sutterella
g__Oscillibacter
g__Escherichia
g__Dialister
g__CAG−127
Others

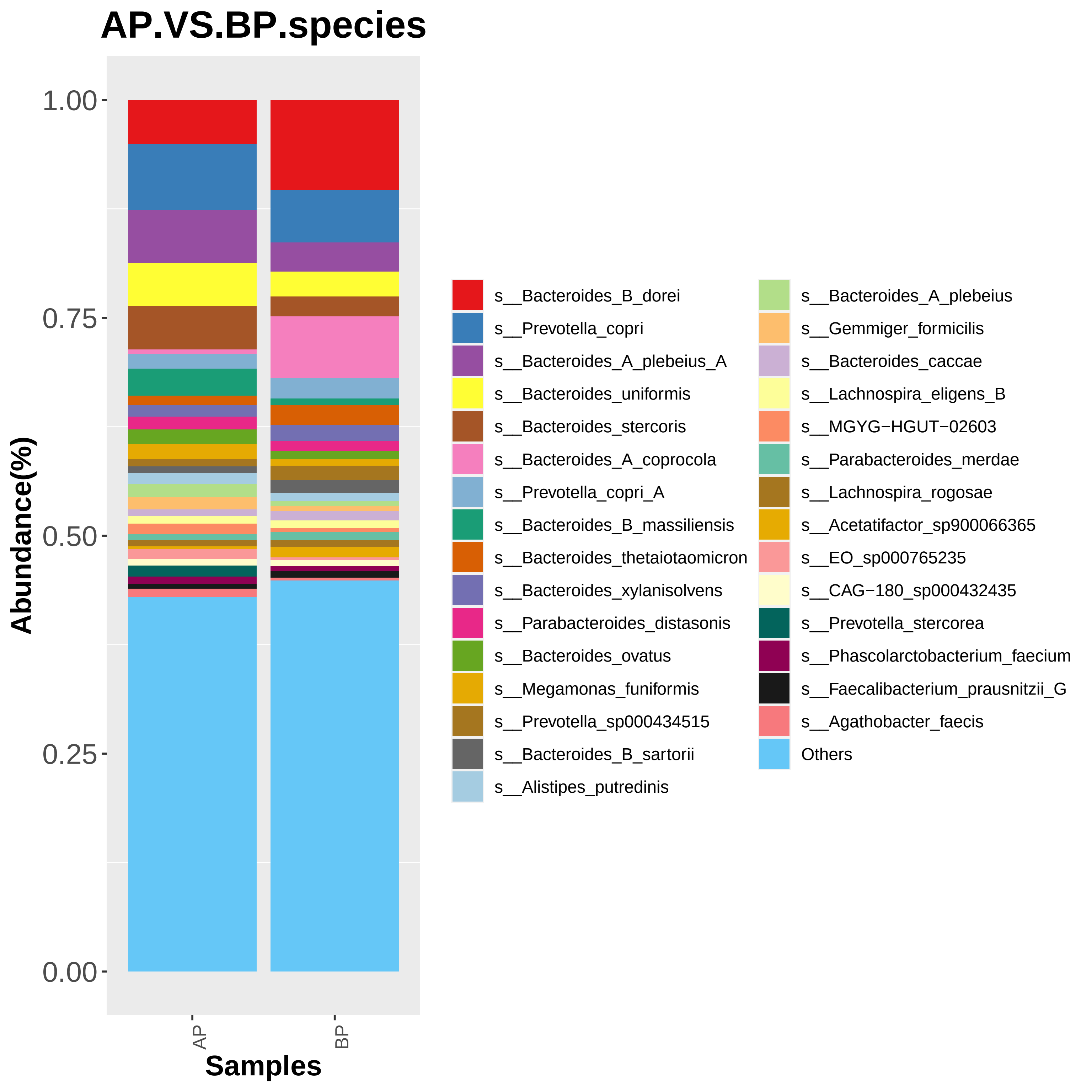
AP.VS.BP.species
Abundance(%)
1.00
0.75
0.50
0.25
0.00
AP
BP
Samples
s__Bacteroides_B_dorei
s__Prevotella_copri
s__Bacteroides_A_plebeius_A
s__Bacteroides_uniformis
s__Bacteroides_stercoris
s__Bacteroides_A_coprocola
s__Prevotella_copri_A
s__Bacteroides_B_massiliensis
s__Bacteroides_thetaiotaomicron
s__Bacteroides_xylanisolvens
s__Parabacteroides_distasonis
s__Bacteroides_ovatus
s__Megamonas_funiformis
s__Prevotella_sp000434515
s__Bacteroides_B_sartorii
s__Alistipes_putredinis
s__Bacteroides_A_plebeius
s__Gemmiger_formicilis
s__Bacteroides_caccae
s__Lachnospira_eligens_B
s__MGYG−HGUT−02603
s__Parabacteroides_merdae
s__Lachnospira_rogosae
s__Acetatifactor_sp900066365
s__EO_sp000765235
s__CAG−180_sp000432435
s__Prevotella_stercorea
s__Phascolarctobacterium_faecium
s__Faecalibacterium_prausnitzii_G
s__Agathobacter_faecis
Others

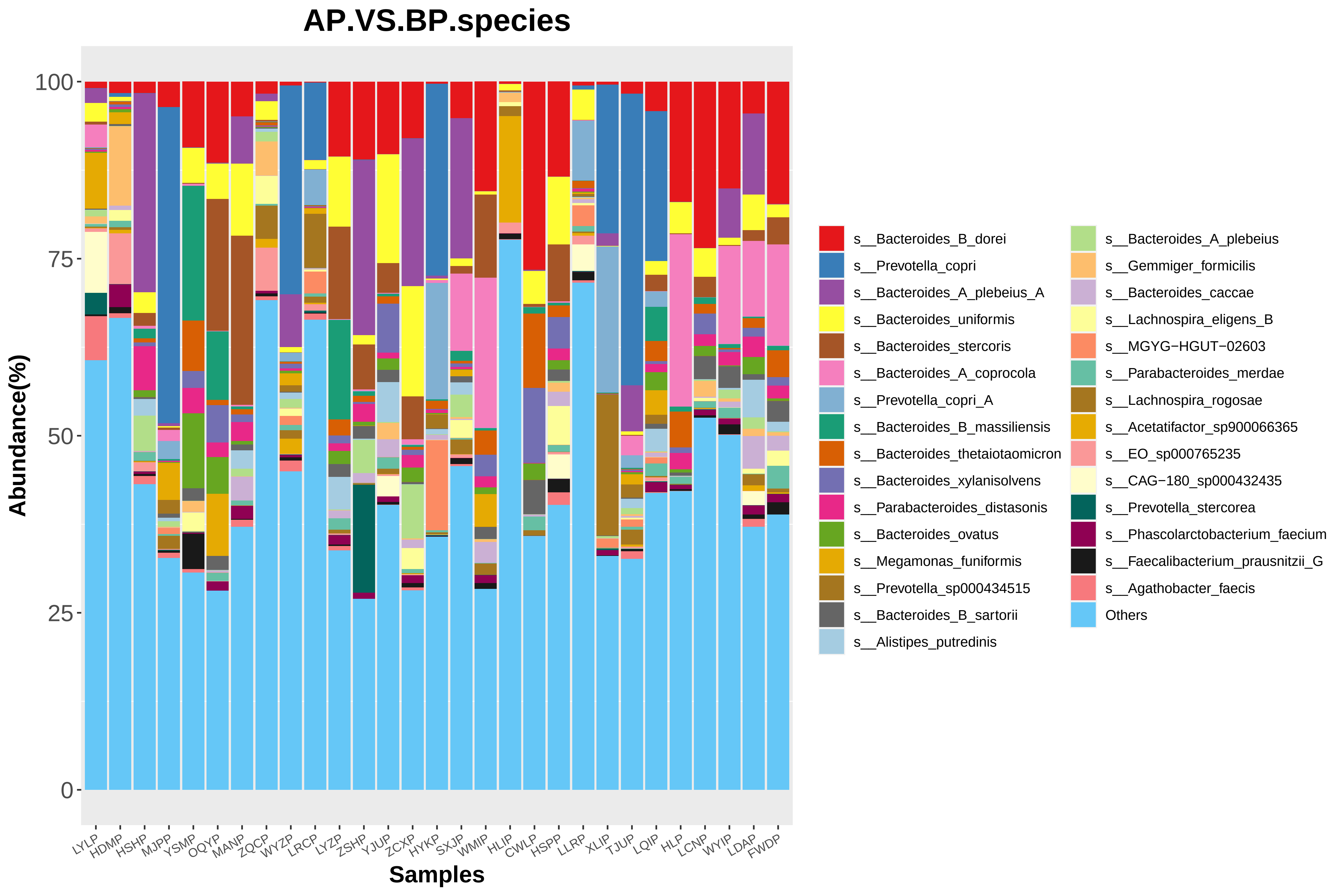
AP.VS.BP.species
Abundance(%)
100
75
50
25
0
Samples
LYLP
HDMP
HSHP
MJPP
YSMP
OQYP
MANP
ZQCP
WYZP
LRCP
LYZP
ZSHP
YJUP
ZCXP
HYKP
SXJP
WMIP
HLIP
CWLP
HSPP
LLRP
XLIP
TJUP
LQIP
HLP
LCNP
WYIP
LDAP
FWDP
s__Bacteroides_B_dorei
s__Prevotella_copri
s__Bacteroides_A_plebeius_A
s__Bacteroides_uniformis
s__Bacteroides_stercoris
s__Bacteroides_A_coprocola
s__Prevotella_copri_A
s__Bacteroides_B_massiliensis
s__Bacteroides_thetaiotaomicron
s__Bacteroides_xylanisolvens
s__Parabacteroides_distasonis
s__Bacteroides_ovatus
s__Megamonas_funiformis
s__Prevotella_sp000434515
s__Bacteroides_B_sartorii
s__Alistipes_putredinis
s__Bacteroides_A_plebeius
s__Gemmiger_formicilis
s__Bacteroides_caccae
s__Lachnospira_eligens_B
s__MGYG−HGUT−02603
s__Parabacteroides_merdae
s__Lachnospira_rogosae
s__Acetatifactor_sp900066365
s__EO_sp000765235
s__CAG−180_sp000432435
s__Prevotella_stercorea
s__Phascolarctobacterium_faecium
s__Faecalibacterium_prausnitzii_G
s__Agathobacter_faecis
Others

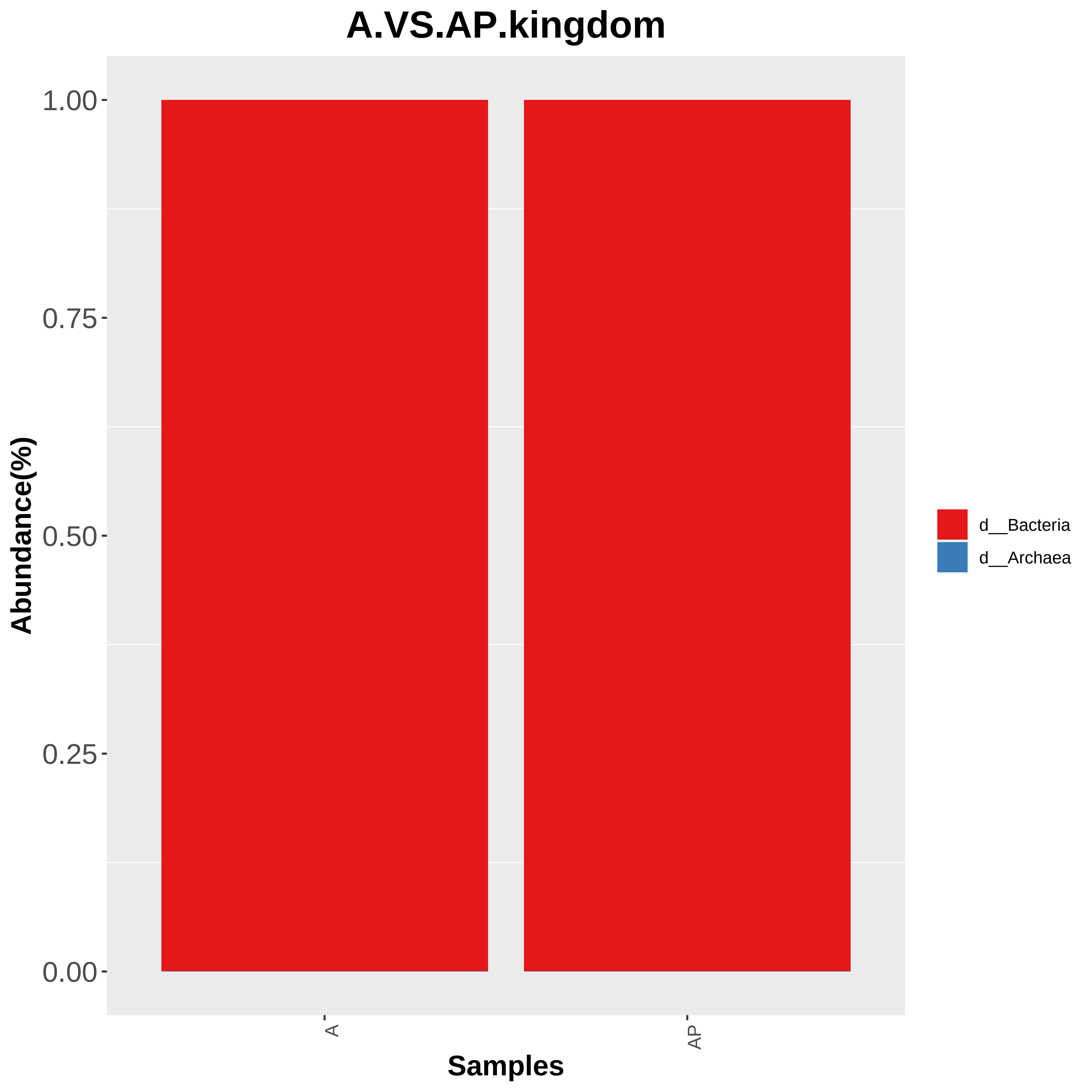
A.VS.AP.kingdom
Abundance(%)
1.00
0.75
0.50
0.25
0.00
A
AP
Samples
d__Bacteria
d__Archaea

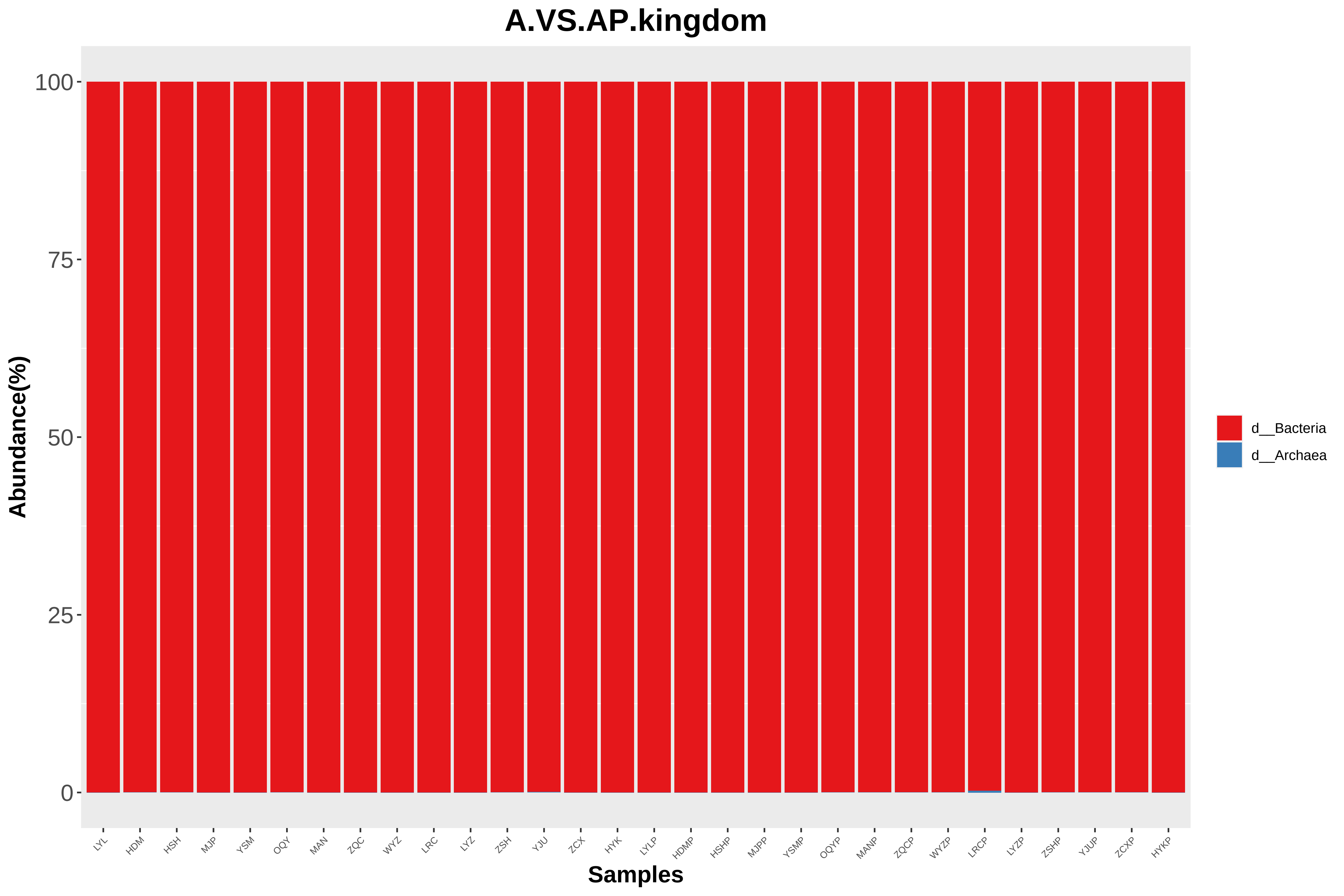

A.VS.AP.kingdom
Abundance(%)
100
75
50
25
0
LYL
HDM
HSH
MJP
YSM
OQY
MAN
ZQC
WYZ
LRC
LYZ
ZSH
YJU
ZCX
HYK
LYLP
HDMP
HSHP
MJPP
YSMP
OQYP
MANP
ZQCP
WYZP
LRCP
LYZP
ZSHP
YJUP
ZCXP
HYKP
Samples
d__Bacteria
d__Archaea

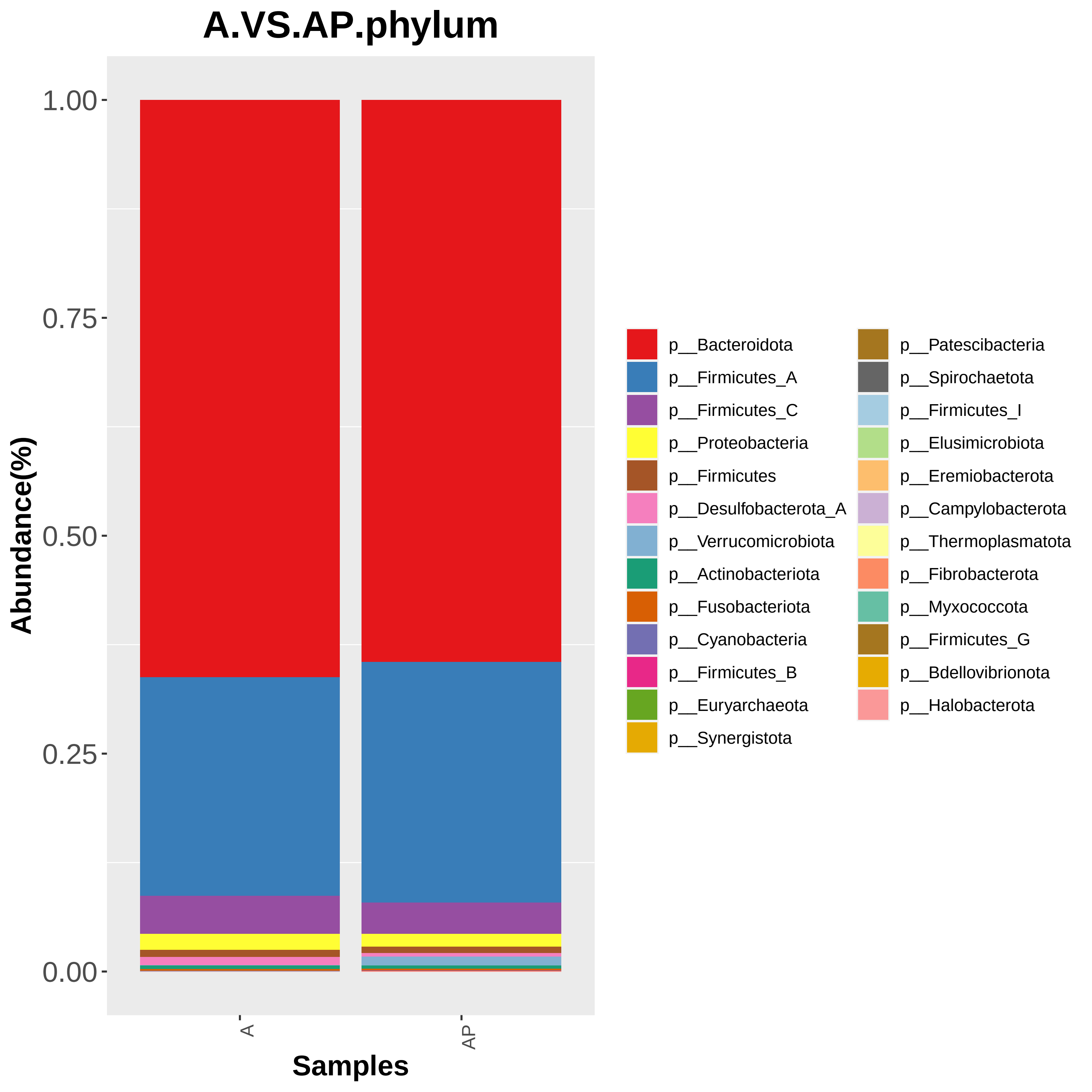

A.VS.AP.phylum
1.00
0.75
0.50
0.25
0.00
Abundance(%)
A
AP
Samples
p__Bacteroidota
p__Firmicutes_A
p__Firmicutes_C
p__Proteobacteria
p__Firmicutes
p__Desulfobacterota_A
p__Verrucomicrobiota
p__Actinobacteriota
p__Fusobacteriota
p__Cyanobacteria
p__Firmicutes_B
p__Euryarchaeota
p__Synergistota
p__Patescibacteria
p__Spirochaetota
p__Firmicutes_I
p__Elusimicrobiota
p__Eremiobacterota
p__Campylobacterota
p__Thermoplasmatota
p__Fibrobacterota
p__Myxococcota
p__Firmicutes_G
p__Bdellovibrionota
p__Halobacterota

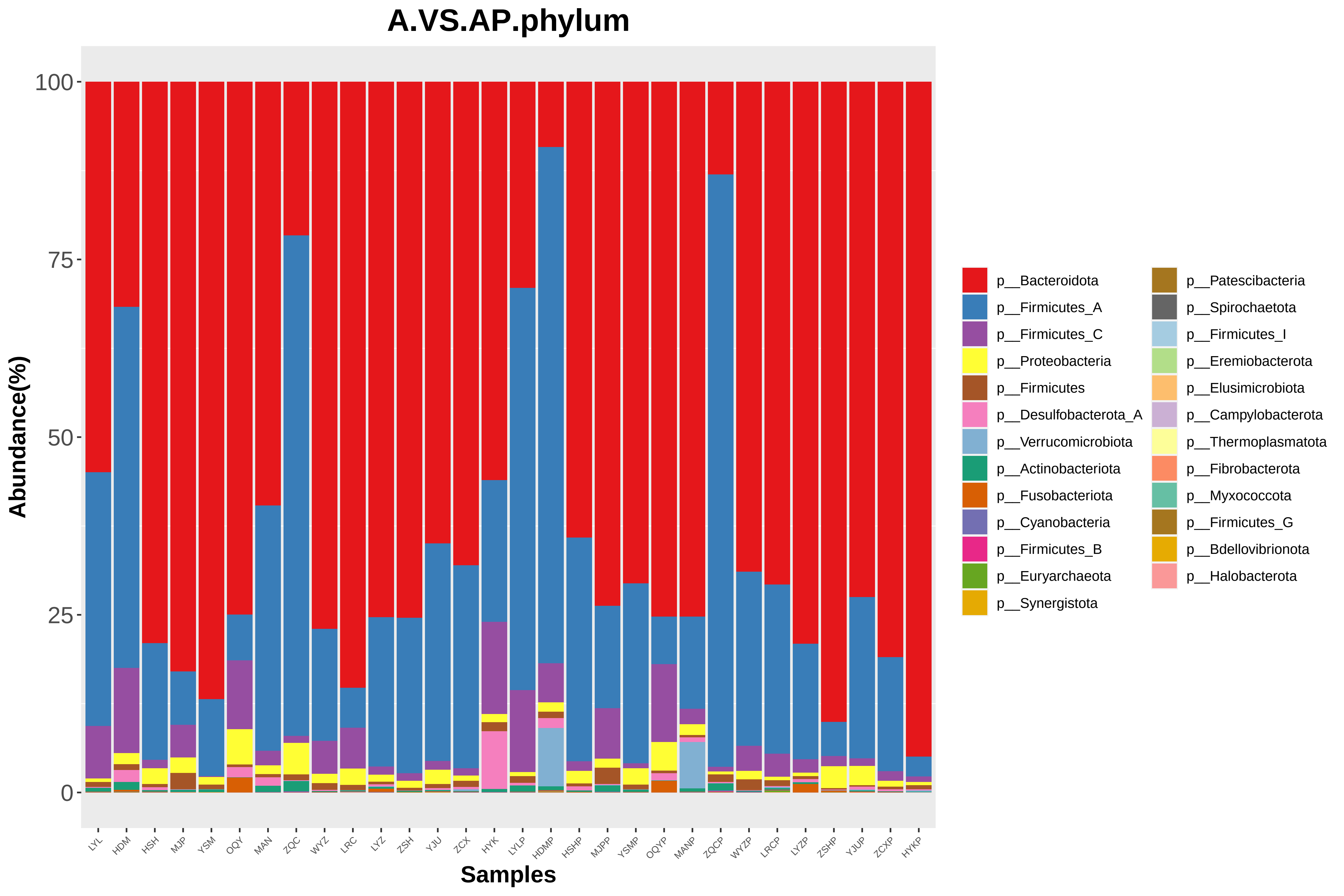

A.VS.AP.phylum
Abundance(%)
100
75
50
25
0
LYL
HDM
HSH
MJP
YSM
OQY
MAN
ZQC
WYZ
LRC
LYZ
ZSH
YJU
ZCX
HYK
LYLP
HDMP
HSHP
MJPP
YSMP
OQYP
MANP
ZQCP
WYZP
LRCP
LYZP
ZSHP
YJUP
ZCXP
HYKP
Samples
p__Bacteroidota
p__Firmicutes_A
p__Firmicutes_C
p__Proteobacteria
p__Firmicutes
p__Desulfobacterota_A
p__Verrucomicrobiota
p__Actinobacteriota
p__Fusobacteriota
p__Cyanobacteria
p__Firmicutes_B
p__Euryarchaeota
p__Synergistota
p__Patescibacteria
p__Spirochaetota
p__Firmicutes_I
p__Eremiobacterota
p__Elusimicrobiota
p__Campylobacterota
p__Thermoplasmatota
p__Fibrobacterota
p__Myxococcota
p__Firmicutes_G
p__Bdellovibrionota
p__Halobacterota

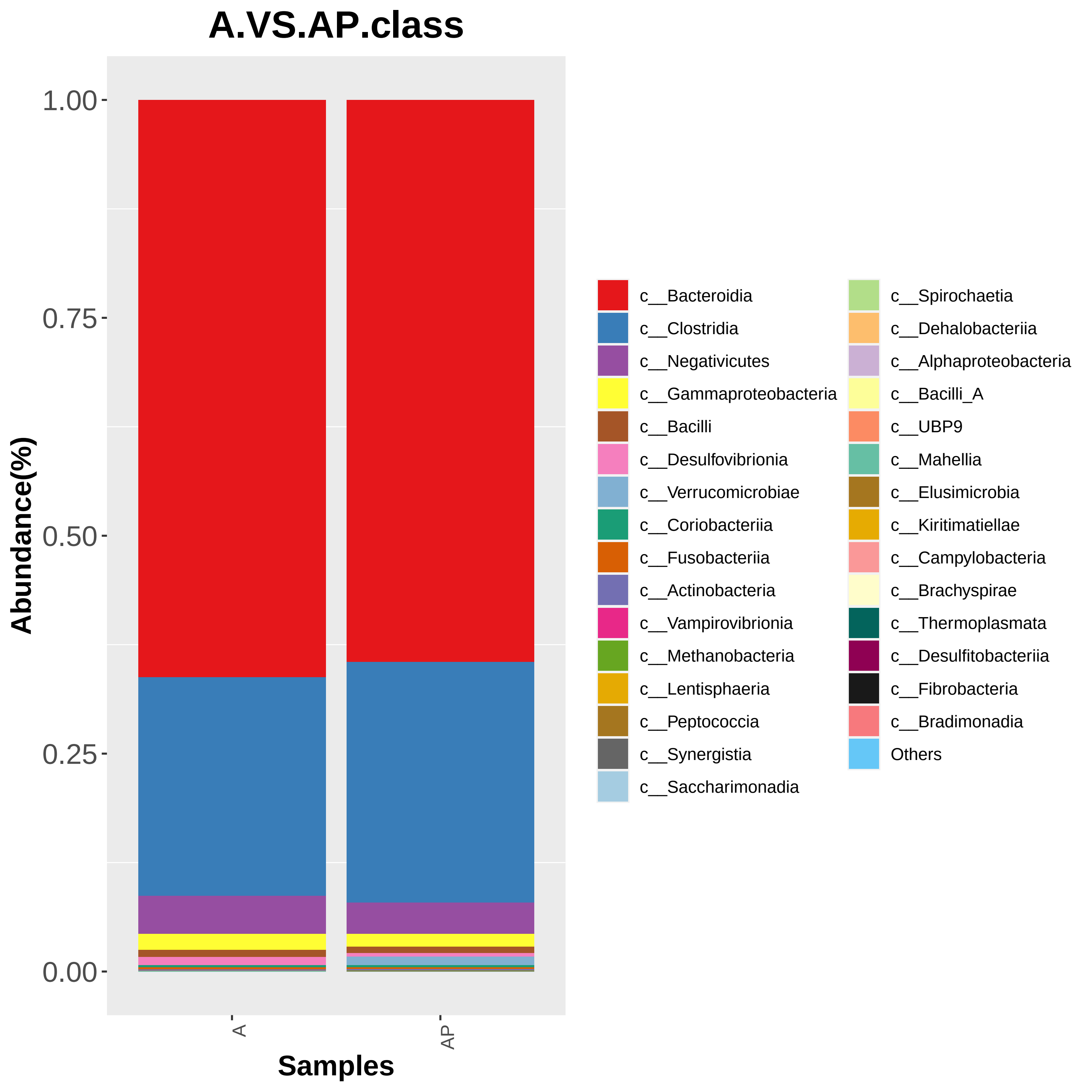
A.VS.AP.class
Abundance(%)
1.00
0.75
0.50
0.25
0.00
A
AP
Samples
c__Bacteroidia
c__Clostridia
c__Negativicutes
c__Gammaproteobacteria
c__Bacilli
c__Desulfovibrionia
c__Verrucomicrobiae
c__Coriobacteriia
c__Fusobacteriia
c__Actinobacteria
c__Vampirovibrionia
c__Methanobacteria
c__Lentisphaeria
c__Peptococcia
c__Synergistia
c__Saccharimonadia
c__Spirochaetia
c__Dehalobacteriia
c__Alphaproteobacteria
c__Bacilli_A
c__UBP9
c__Mahellia
c__Elusimicrobia
c__Kiritimatiellae
c__Campylobacteria
c__Brachyspirae
c__Thermoplasmata
c__Desulfitobacteriia
c__Fibrobacteria
c__Bradimonadia
Others

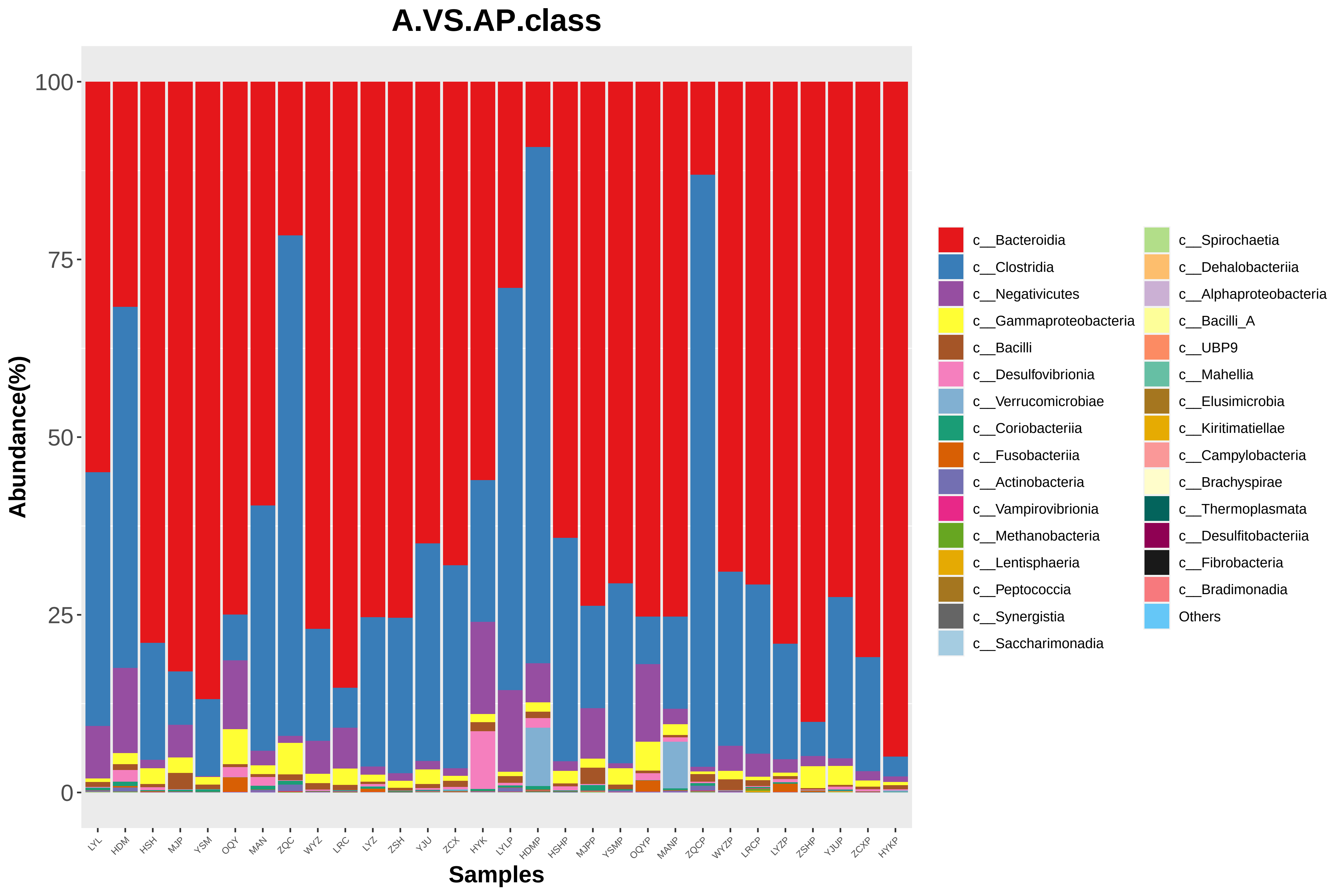
A.VS.AP.class
Abundance(%)
100
75
50
25
0
LYL
HDM
HSH
MJP
YSM
OQY
MAN
ZQC
WYZ
LRC
LYZ
ZSH
YJU
ZCX
HYK
LYLP
HDMP
HSHP
MJPP
YSMP
OQYP
MANP
ZQCP
WYZP
LRCP
LYZP
ZSHP
YJUP
ZCXP
HYKP
Samples
c__Bacteroidia
c__Clostridia
c__Negativicutes
c__Gammaproteobacteria
c__Bacilli
c__Desulfovibrionia
c__Verrucomicrobiae
c__Coriobacteriia
c__Fusobacteriia
c__Actinobacteria
c__Vampirovibrionia
c__Methanobacteria
c__Lentisphaeria
c__Peptococcia
c__Synergistia
c__Saccharimonadia
c__Spirochaetia
c__Dehalobacteriia
c__Alphaproteobacteria
c__Bacilli_A
c__UBP9
c__Mahellia
c__Elusimicrobia
c__Kiritimatiellae
c__Campylobacteria
c__Brachyspirae
c__Thermoplasmata
c__Desulfitobacteriia
c__Fibrobacteria
c__Bradimonadia
Others

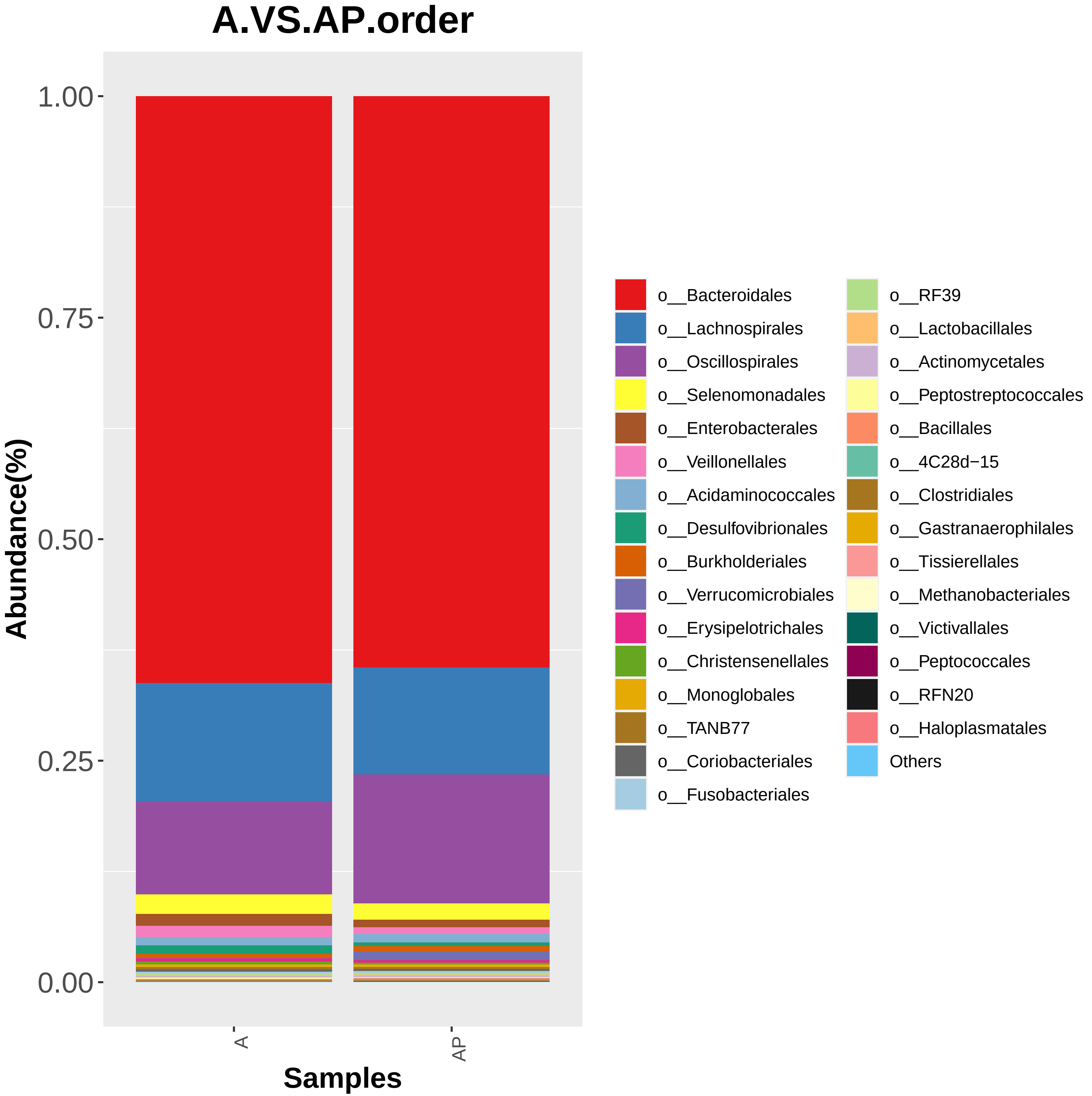

A.VS.AP.order
Abundance(%)
1.00
0.75
0.50
0.25
0.00
A
AP
Samples
o__Bacteroidales
o__Lachnospirales
o__Oscillospirales
o__Selenomonadales
o__Enterobacterales
o__Veillonellales
o__Acidaminococcales
o__Desulfovibrionales
o__Burkholderiales
o__Verrucomicrobiales
o__Erysipelotrichales
o__Christensenellales
o__Monoglobales
o__TANB77
o__Coriobacteriales
o__Fusobacteriales
o__RF39
o__Lactobacillales
o__Actinomycetales
o__Peptostreptococcales
o__Bacillales
o__4C28d−15
o__Clostridiales
o__Gastranaerophilales
o__Tissierellales
o__Methanobacteriales
o__Victivallales
o__Peptococcales
o__RFN20
o__Haloplasmatales
Others

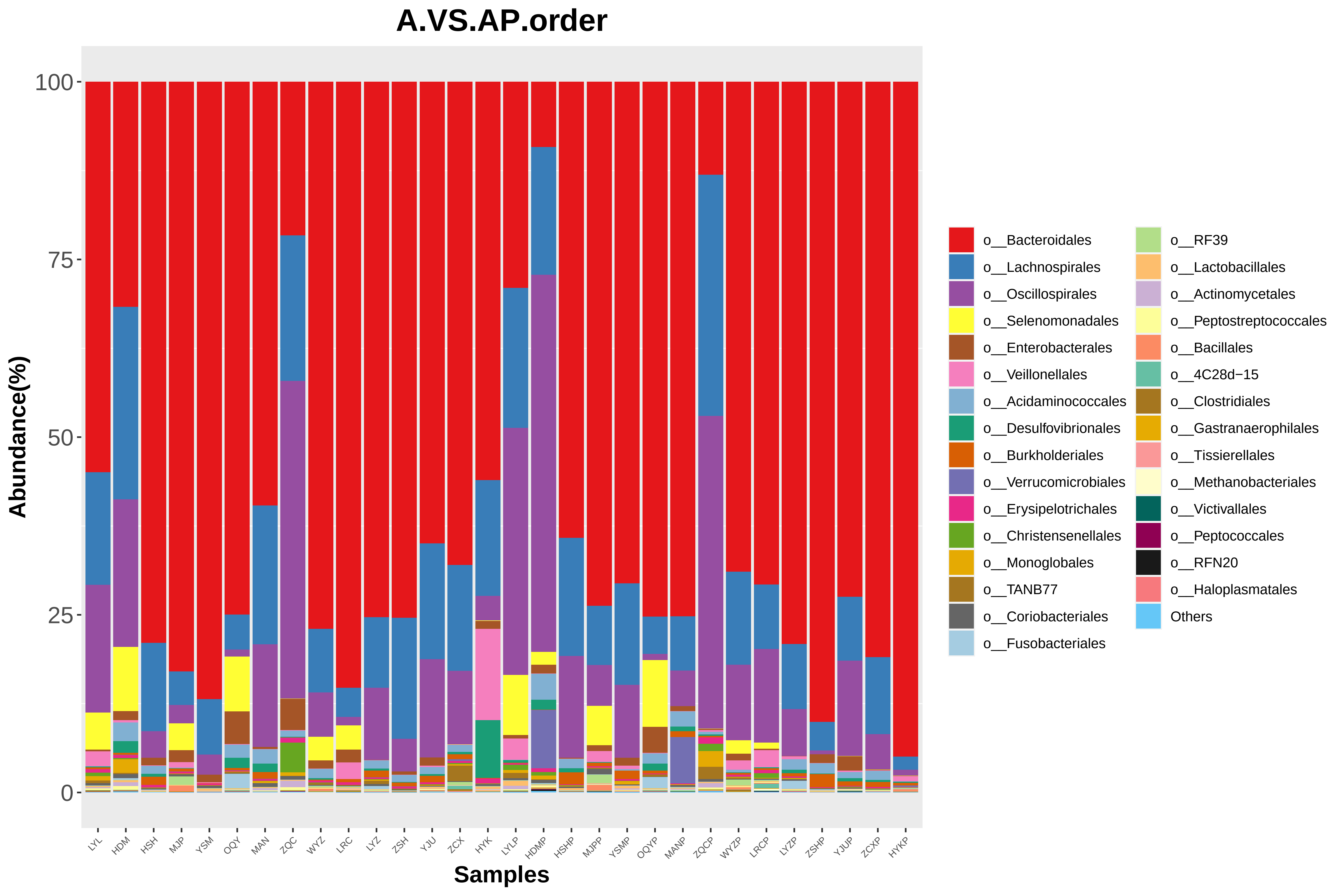
A.VS.AP.order
Abundance(%)
100
75
50
25
0
LYL
HDM
HSH
MJP
YSM
OQY
MAN
ZQC
WYZ
LRC
LYZ
ZSH
YJU
ZCX
HYK
LYLP
HDMP
HSHP
MJPP
YSMP
OQYP
MANP
ZQCP
WYZP
LRCP
LYZP
ZSHP
YJUP
ZCXP
HYKP
Samples
o__Bacteroidales
o__Lachnospirales
o__Oscillospirales
o__Selenomonadales
o__Enterobacterales
o__Veillonellales
o__Acidaminococcales
o__Desulfovibrionales
o__Burkholderiales
o__Verrucomicrobiales
o__Erysipelotrichales
o__Christensenellales
o__Monoglobales
o__TANB77
o__Coriobacteriales
o__Fusobacteriales
o__RF39
o__Lactobacillales
o__Actinomycetales
o__Peptostreptococcales
o__Bacillales
o__4C28d−15
o__Clostridiales
o__Gastranaerophilales
o__Tissierellales
o__Methanobacteriales
o__Victivallales
o__Peptococcales
o__RFN20
o__Haloplasmatales
Others

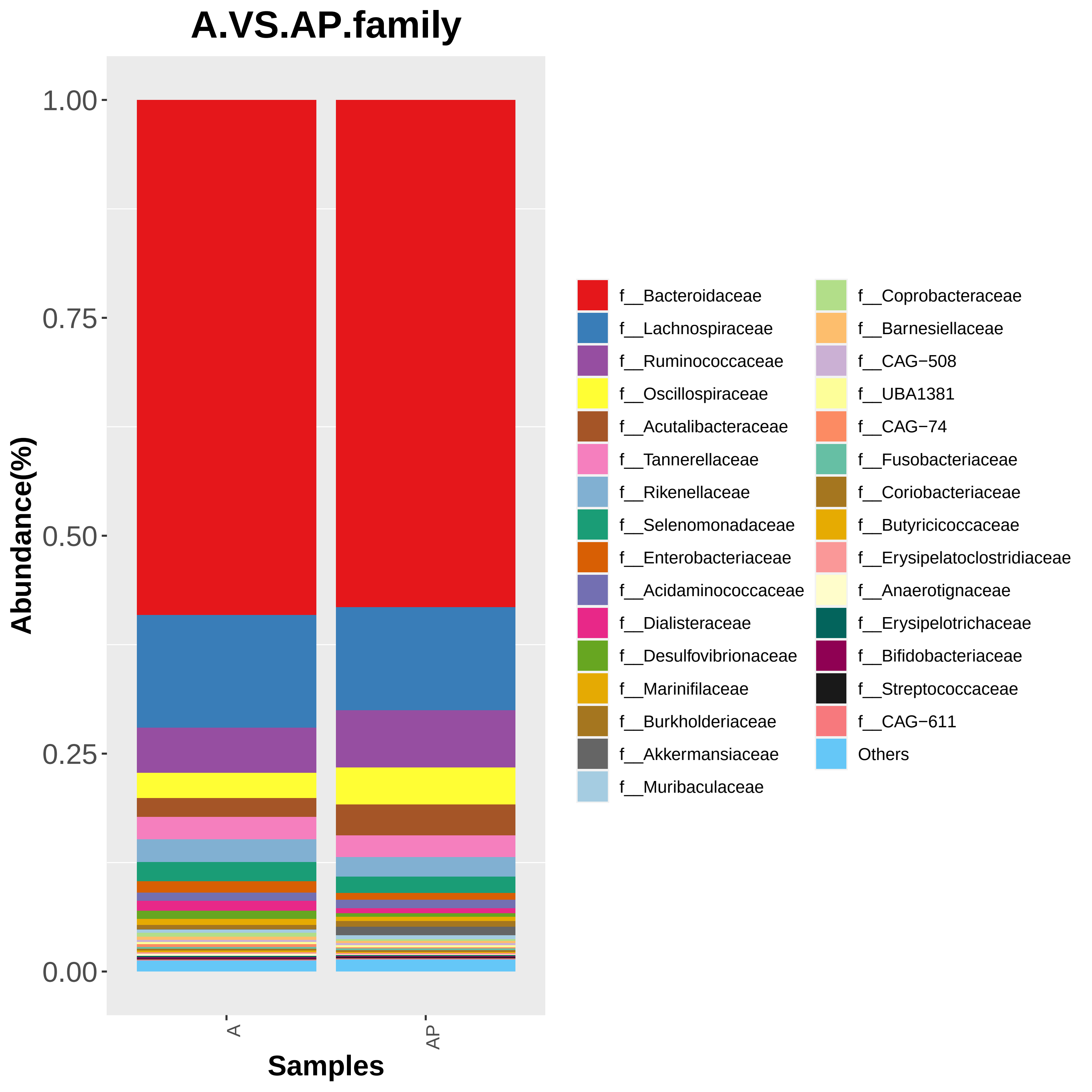

A.VS.AP.family
Abundance(%)
1.00
0.75
0.50
0.25
0.00
A
AP
Samples
f__Bacteroidaceae
f__Lachnospiraceae
f__Ruminococcaceae
f__Oscillospiraceae
f__Acutalibacteraceae
f__Tannerellaceae
f__Rikenellaceae
f__Selenomonadaceae
f__Enterobacteriaceae
f__Acidaminococcaceae
f__Dialisteraceae
f__Desulfovibrionaceae
f__Marinifilaceae
f__Burkholderiaceae
f__Akkermansiaceae
f__Muribaculaceae
f__Coprobacteraceae
f__Barnesiellaceae
f__CAG−508
f__UBA1381
f__CAG−74
f__Fusobacteriaceae
f__Coriobacteriaceae
f__Butyricicoccaceae
f__Erysipelatoclostridiaceae
f__Anaerotignaceae
f__Erysipelotrichaceae
f__Bifidobacteriaceae
f__Streptococcaceae
f__CAG−611
Others

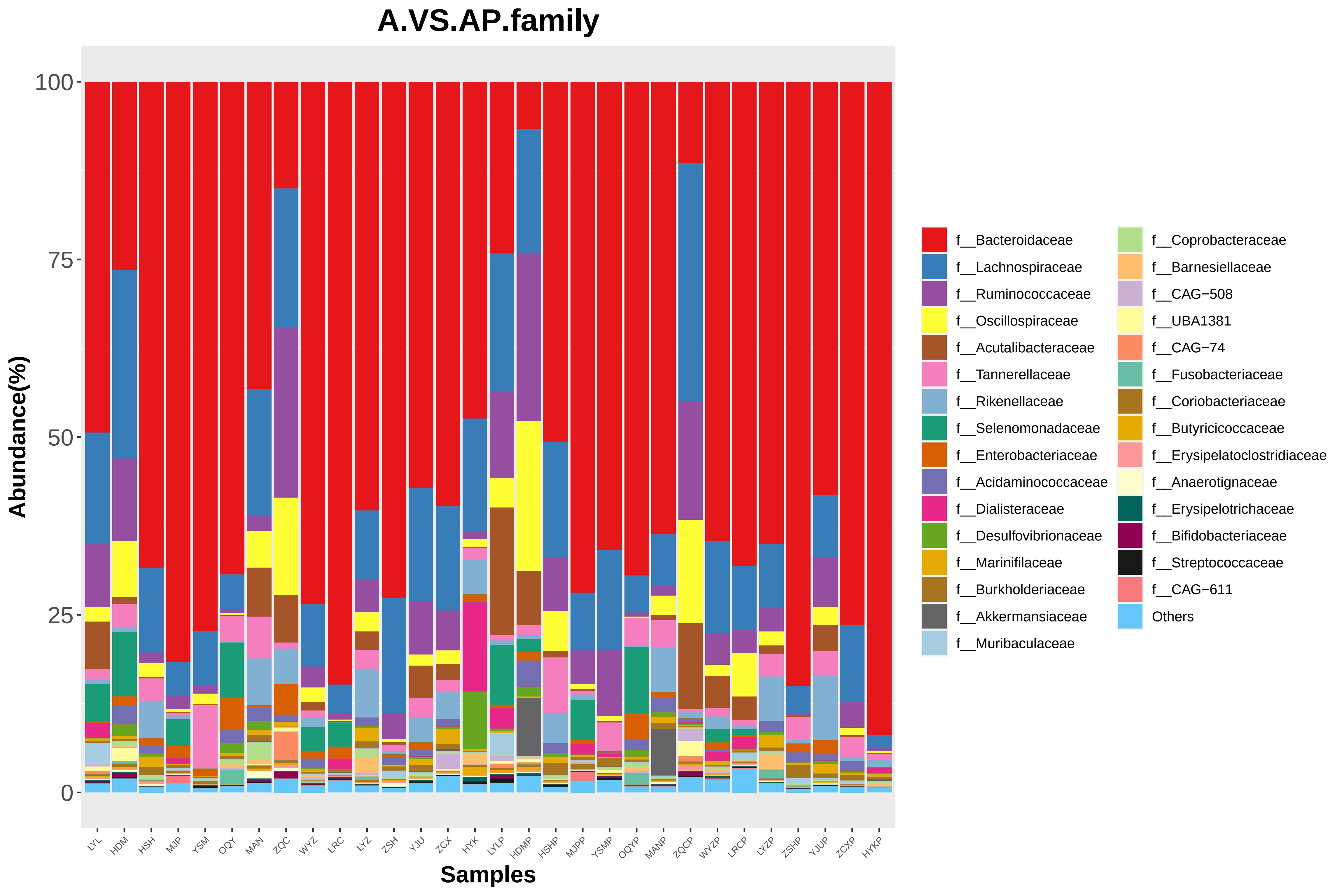
A.VS.AP.family
Abundance(%)
100
75
50
25
0
Samples
LYL
HDM
HSH
MJP
YSM
OQY
MAN
ZQC
WYZ
LRC
LYZ
ZSH
YJU
ZCX
HYK
LYLP
HDMP
HSHP
MJPP
YSMP
OQYP
MANP
ZQCP
WYZP
LRCP
LYZP
ZSHP
YJUP
ZCXP
HYKP
f__Bacteroidaceae
f__Lachnospiraceae
f__Ruminococcaceae
f__Oscillospiraceae
f__Acutalibacteraceae
f__Tannerellaceae
f__Rikenellaceae
f__Selenomonadaceae
f__Enterobacteriaceae
f__Acidaminococcaceae
f__Dialisteraceae
f__Desulfovibrionaceae
f__Marinifilaceae
f__Burkholderiaceae
f__Akkermansiaceae
f__Muribaculaceae
f__Coprobacteraceae
f__Barnesiellaceae
f__CAG−508
f__UBA1381
f__CAG−74
f__Fusobacteriaceae
f__Coriobacteriaceae
f__Butyricicoccaceae
f__Erysipelatoclostridiaceae
f__Anaerotignaceae
f__Erysipelotrichaceae
f__Bifidobacteriaceae
f__Streptococcaceae
f__CAG−611
Others

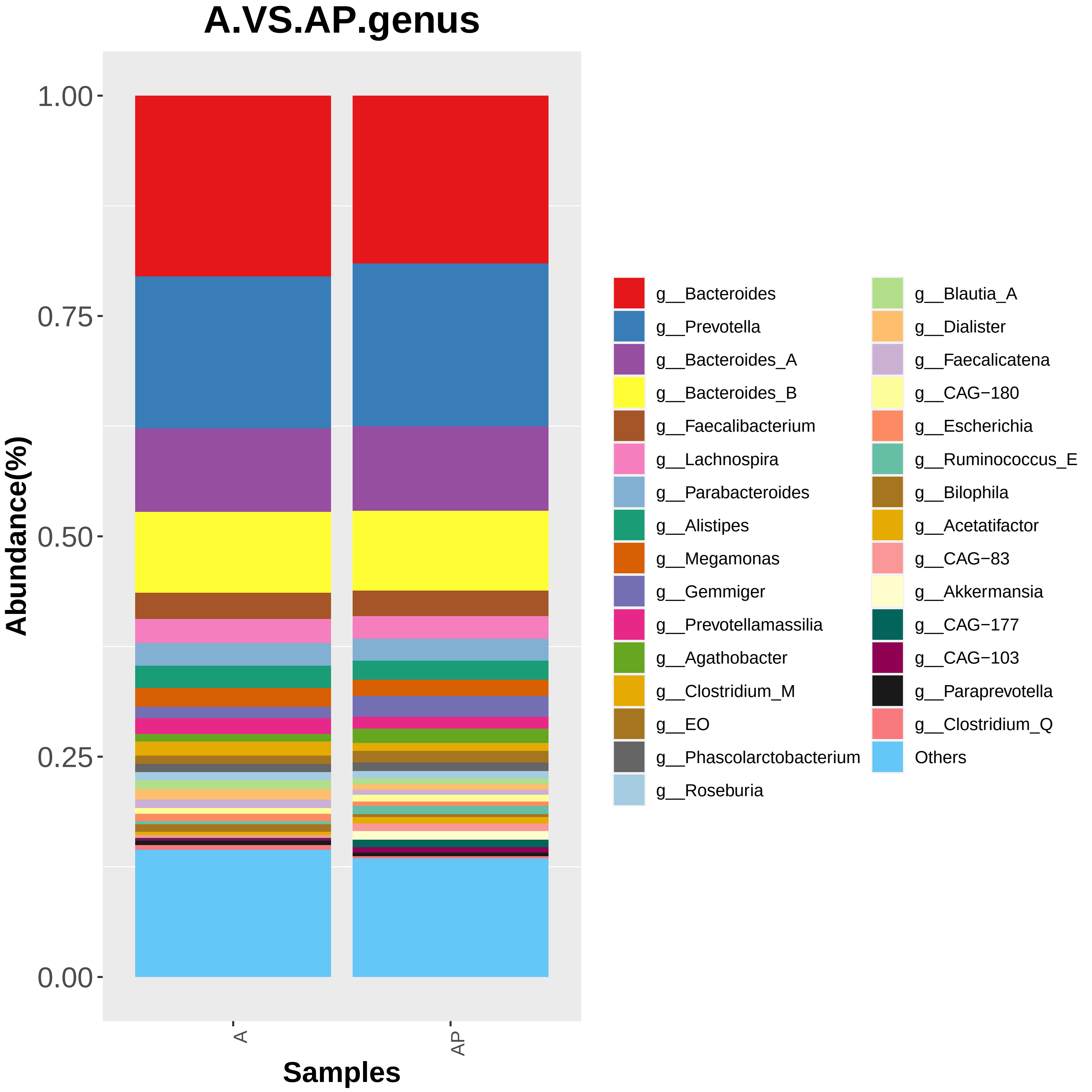

A.VS.AP.genus
Abundance(%)
1.00
0.75
0.50
0.25
0.00
A
AP
Samples
g__Bacteroides
g__Prevotella
g__Bacteroides_A
g__Bacteroides_B
g__Faecalibacterium
g__Lachnospira
g__Parabacteroides
g__Alistipes
g__Megamonas
g__Gemmiger
g__Prevotellamassilia
g__Agathobacter
g__Clostridium_M
g__EO
g__Phascolarctobacterium
g__Roseburia
g__Blautia_A
g__Dialister
g__Faecalicatena
g__CAG−180
g__Escherichia
g__Ruminococcus_E
g__Bilophila
g__Acetatifactor
g__CAG−83
g__Akkermansia
g__CAG−177
g__CAG−103
g__Paraprevotella
g__Clostridium_Q
Others

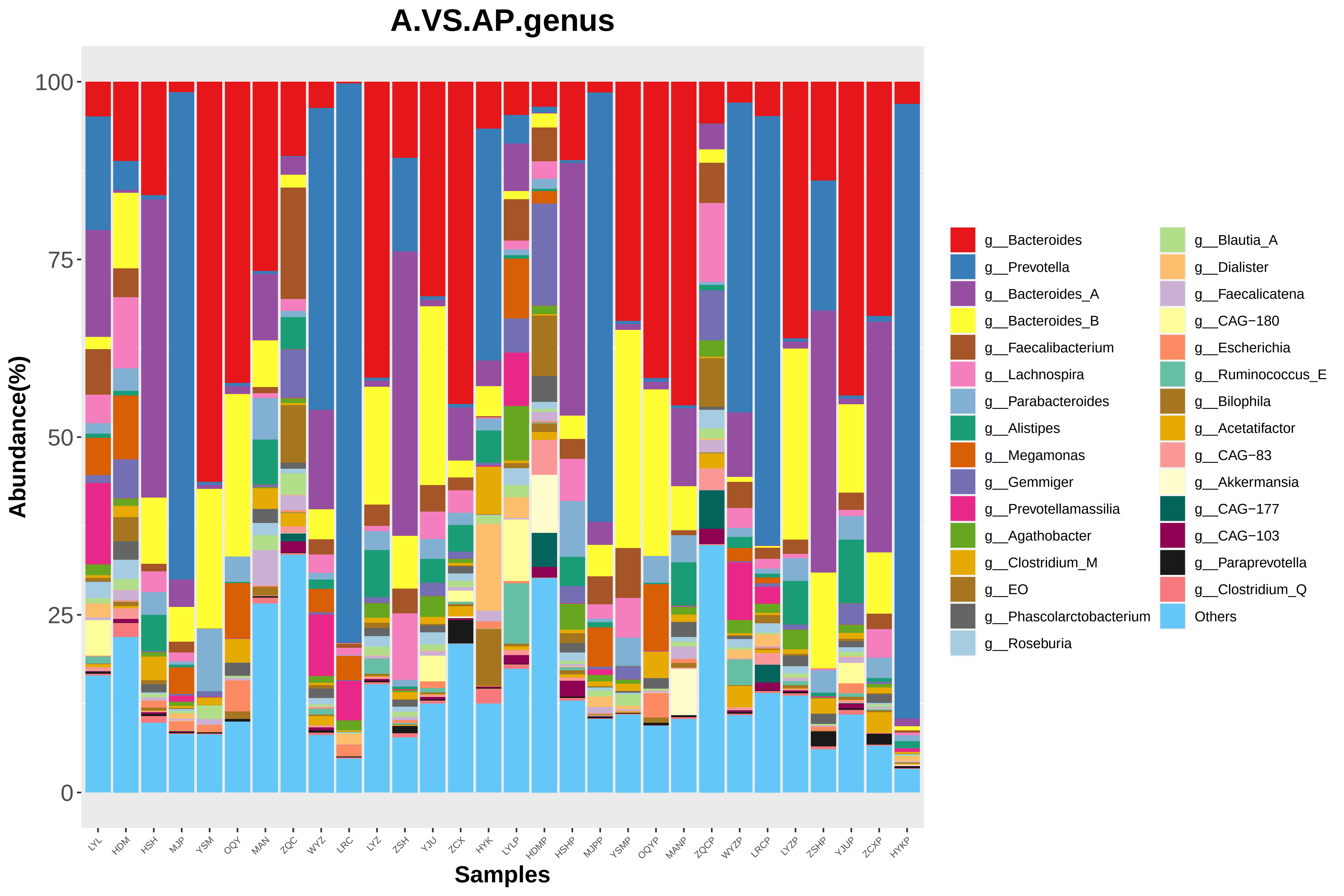

A.VS.AP.genus
Abundance(%)
100
75
50
25
0
LYL
HDM
HSH
MJP
YSM
OQY
MAN
ZQC
WYZ
LRC
LYZ
ZSH
YJU
ZCX
HYK
LYLP
HDMP
HSHP
MJPP
YSMP
OQYP
MANP
ZQCP
WYZP
LRCP
LYZP
ZSHP
YJUP
ZCXP
HYKP
Samples
g__Bacteroides
g__Prevotella
g__Bacteroides_A
g__Bacteroides_B
g__Faecalibacterium
g__Lachnospira
g__Parabacteroides
g__Alistipes
g__Megamonas
g__Gemmiger
g__Prevotellamassilia
g__Agathobacter
g__Clostridium_M
g__EO
g__Phascolarctobacterium
g__Roseburia
g__Blautia_A
g__Dialister
g__Faecalicatena
g__CAG−180
g__Escherichia
g__Ruminococcus_E
g__Bilophila
g__Acetatifactor
g__CAG−83
g__Akkermansia
g__CAG−177
g__CAG−103
g__Paraprevotella
g__Clostridium_Q
Others

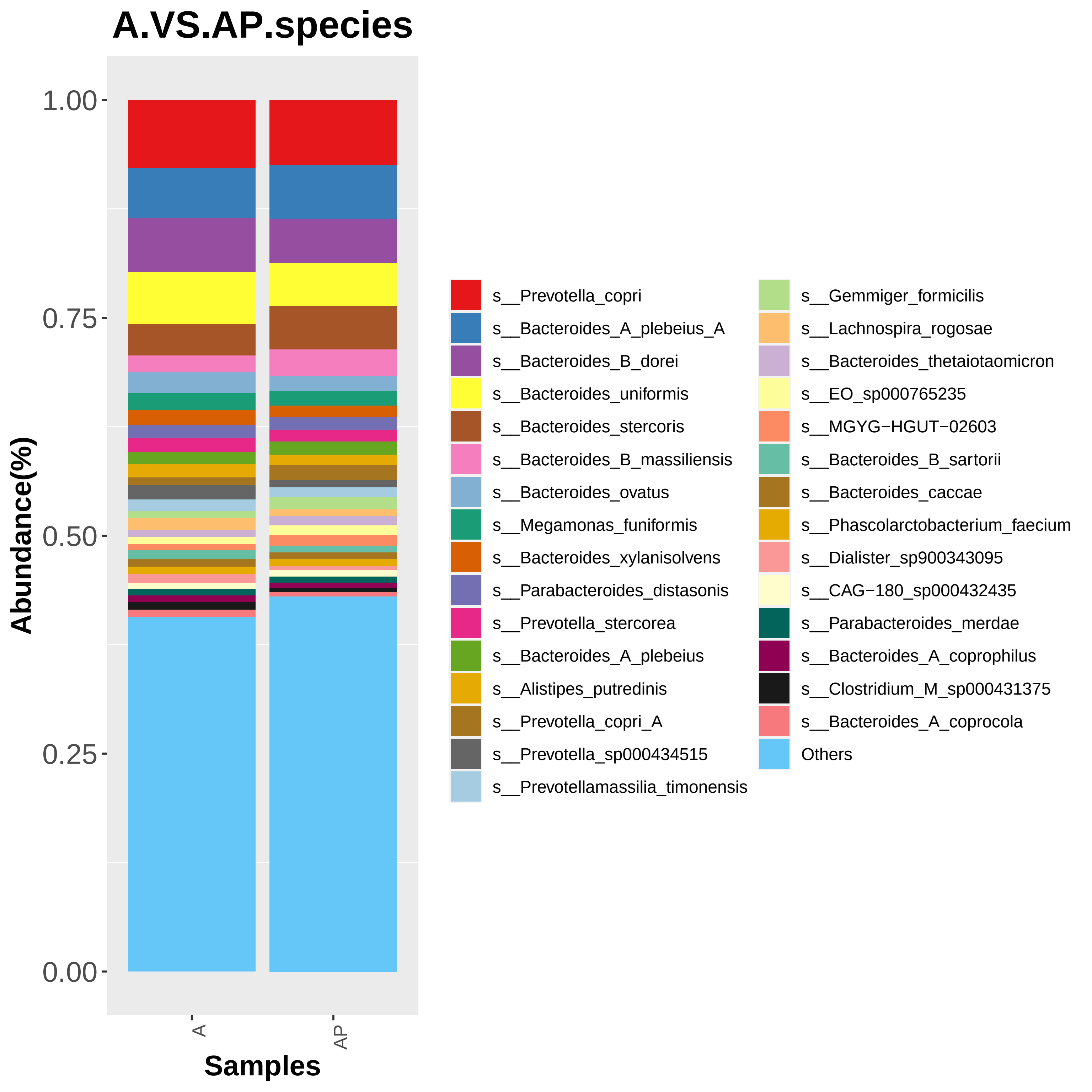

A.VS.AP.species
Abundance(%)
1.00
0.75
0.50
0.25
0.00
A
AP
Samples
s__Prevotella_copri
s__Bacteroides_A_plebeius_A
s__Bacteroides_B_dorei
s__Bacteroides_uniformis
s__Bacteroides_stercoris
s__Bacteroides_B_massiliensis
s__Bacteroides_ovatus
s__Megamonas_funiformis
s__Bacteroides_xylanisolvens
s__Parabacteroides_distasonis
s__Prevotella_stercorea
s__Bacteroides_A_plebeius
s__Alistipes_putredinis
s__Prevotella_copri_A
s__Prevotella_sp000434515
s__Prevotellamassilia_timonensis
s__Gemmiger_formicilis
s__Lachnospira_rogosae
s__Bacteroides_thetaiotaomicron
s__EO_sp000765235
s__MGYG−HGUT−02603
s__Bacteroides_B_sartorii
s__Bacteroides_caccae
s__Phascolarctobacterium_faecium
s__Dialister_sp900343095
s__CAG−180_sp000432435
s__Parabacteroides_merdae
s__Bacteroides_A_coprophilus
s__Clostridium_M_sp000431375
s__Bacteroides_A_coprocola
Others

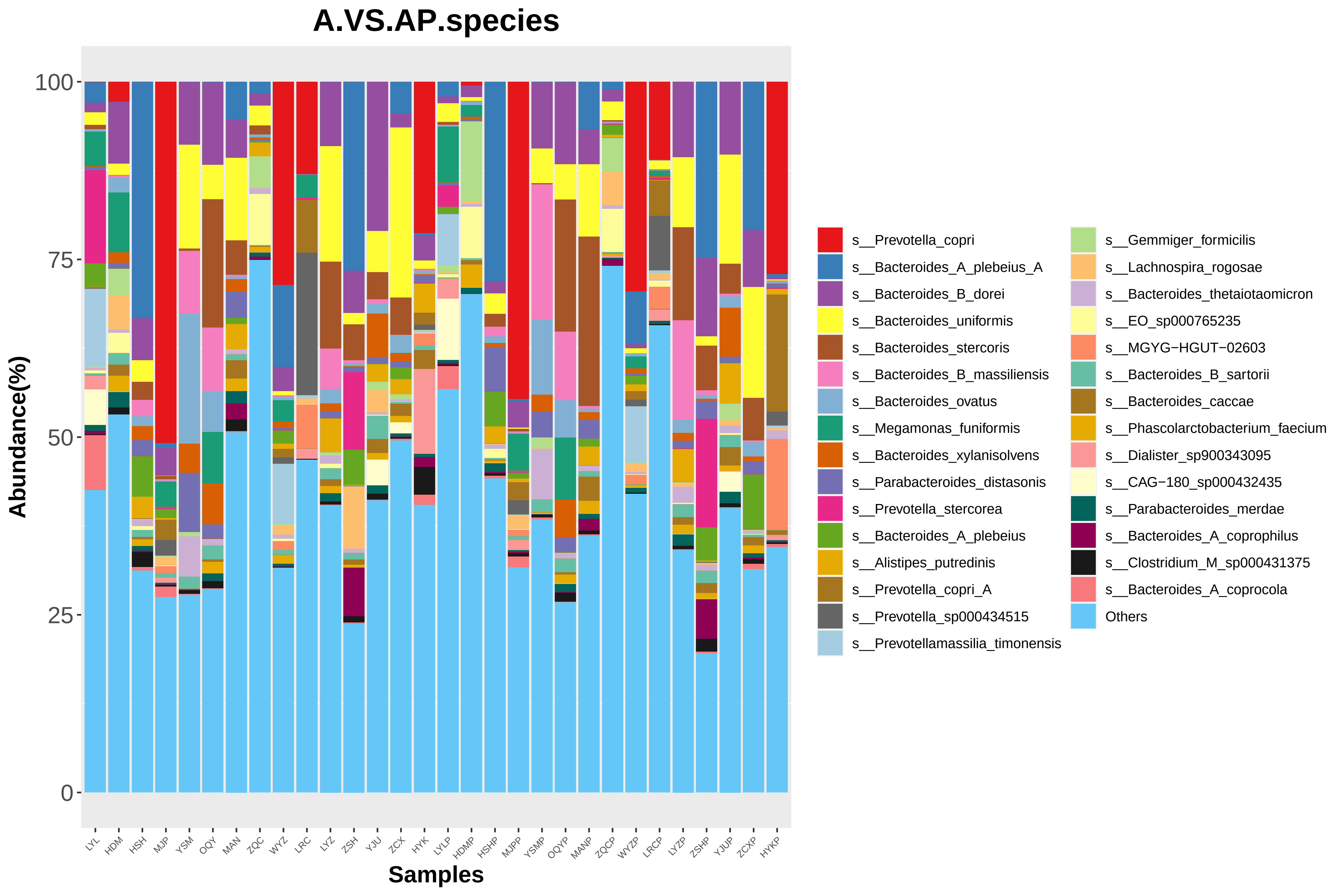
A.VS.AP.species
Abundance(%)
100
75
50
25
0
LYL
HDM
HSH
MJP
YSM
OQY
MAN
ZQC
WYZ
LRC
LYZ
ZSH
YJU
ZCX
HYK
LYLP
HDMP
HSHP
MJPP
YSMP
OQYP
MANP
ZQCP
WYZP
LRCP
LYZP
ZSHP
YJUP
ZCXP
HYKP
Samples
s__Prevotella_copri
s__Bacteroides_A_plebeius_A
s__Bacteroides_B_dorei
s__Bacteroides_uniformis
s__Bacteroides_stercoris
s__Bacteroides_B_massiliensis
s__Bacteroides_ovatus
s__Megamonas_funiformis
s__Bacteroides_xylanisolvens
s__Parabacteroides_distasonis
s__Prevotella_stercorea
s__Bacteroides_A_plebeius
s__Alistipes_putredinis
s__Prevotella_copri_A
s__Prevotella_sp000434515
s__Prevotellamassilia_timonensis
s__Gemmiger_formicilis
s__Lachnospira_rogosae
s__Bacteroides_thetaiotaomicron
s__EO_sp000765235
s__MGYG−HGUT−02603
s__Bacteroides_B_sartorii
s__Bacteroides_caccae
s__Phascolarctobacterium_faecium
s__Dialister_sp900343095
s__CAG−180_sp000432435
s__Parabacteroides_merdae
s__Bacteroides_A_coprophilus
s__Clostridium_M_sp000431375
s__Bacteroides_A_coprocola
Others

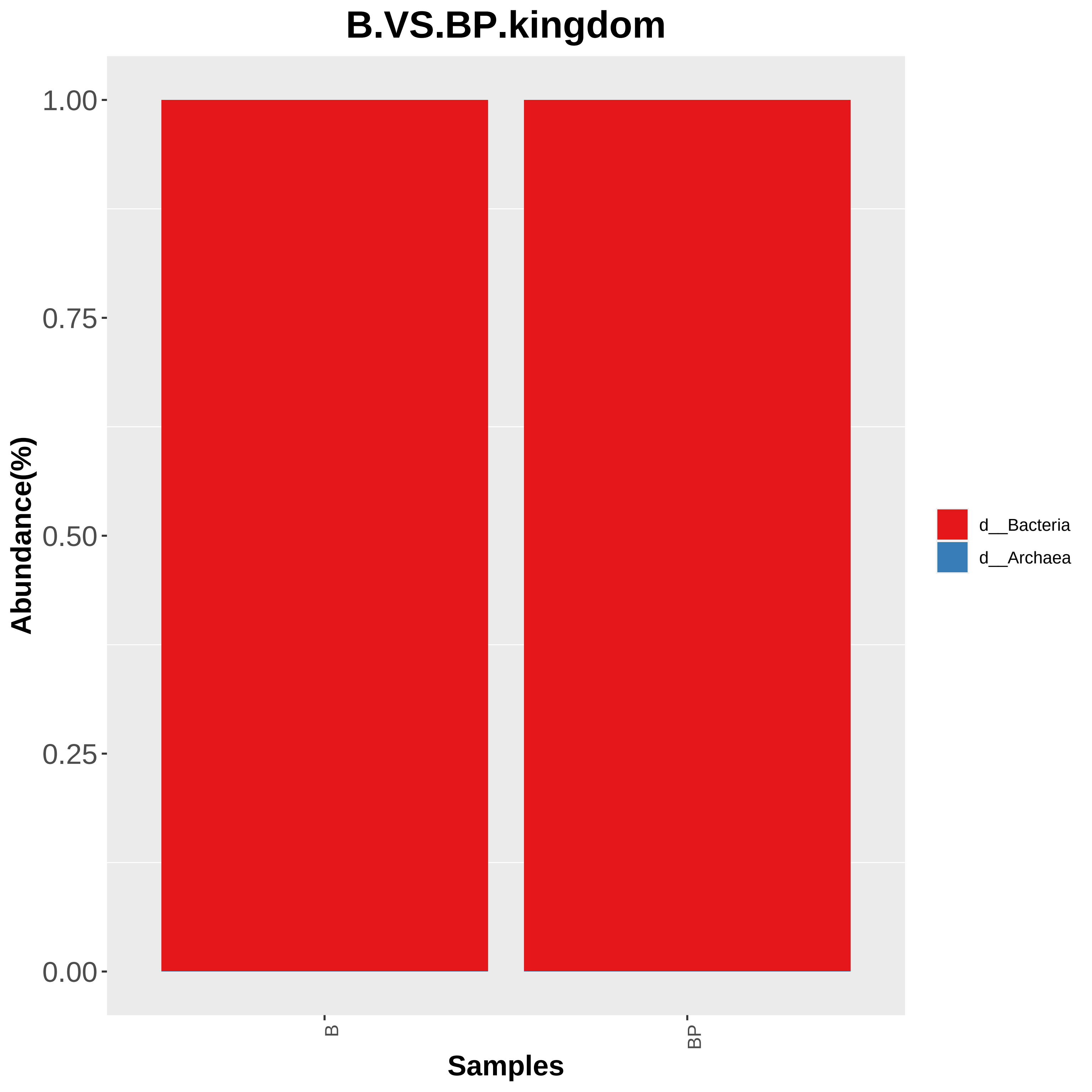

B.VS.BP.kingdom
1.00
0.75
0.50
0.25
0.00
Abundance(%)
B
BP
Samples
d__Bacteria
d__Archaea

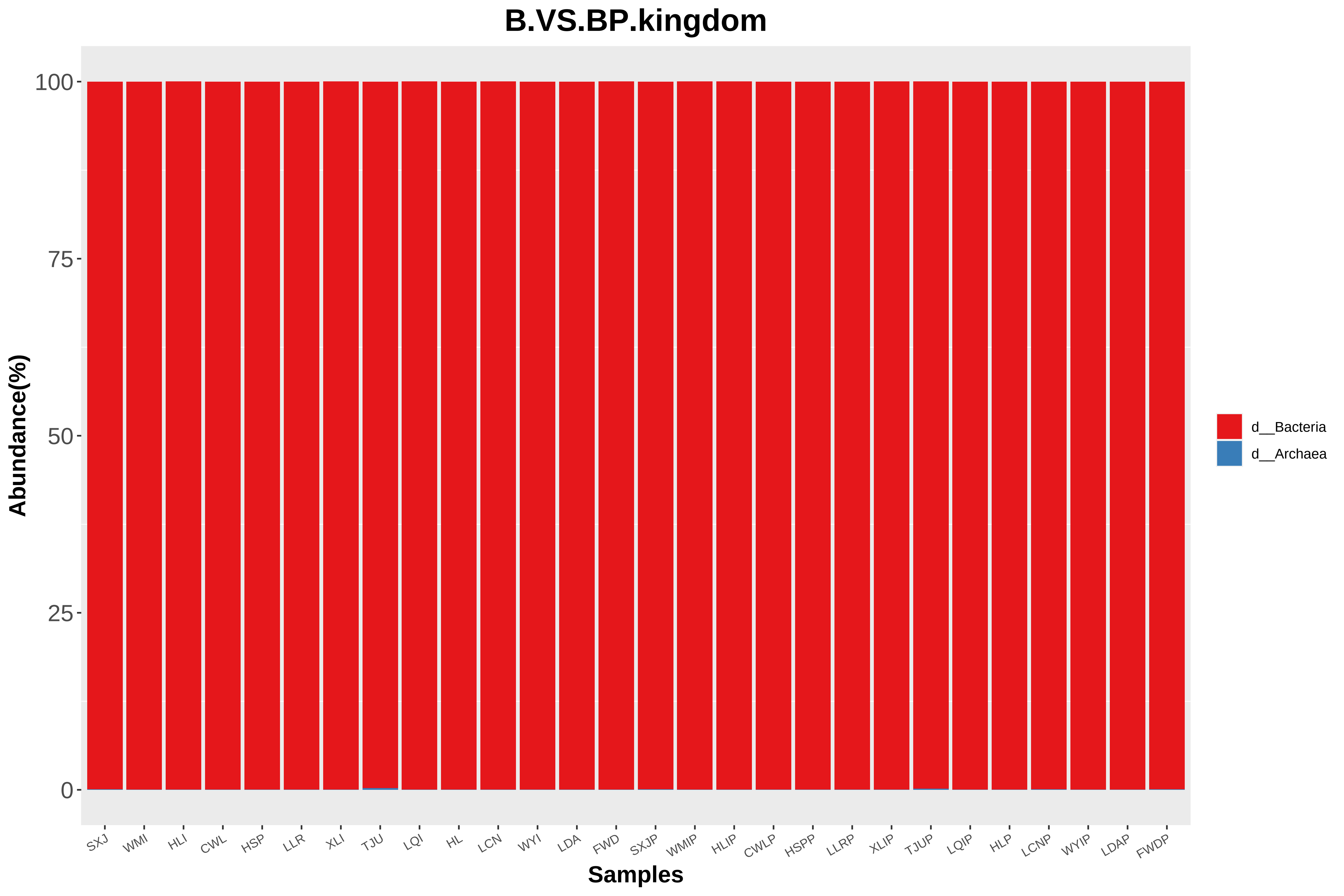
B.VS.BP.kingdom
Abundance(%)
100
75
50
25
0
SXJ
WMI
HLI
CWL
HSP
LLR
XLI
TJU
LQI
HL
LCN
WYI
LDA
FWD
SXJP
WMIP
HLIP
CWLP
HSPP
LLRP
XLIP
TJUP
LQIP
HLP
LCNP
WYIP
LDAP
FWDP
Samples
d__Bacteria
d__Archaea

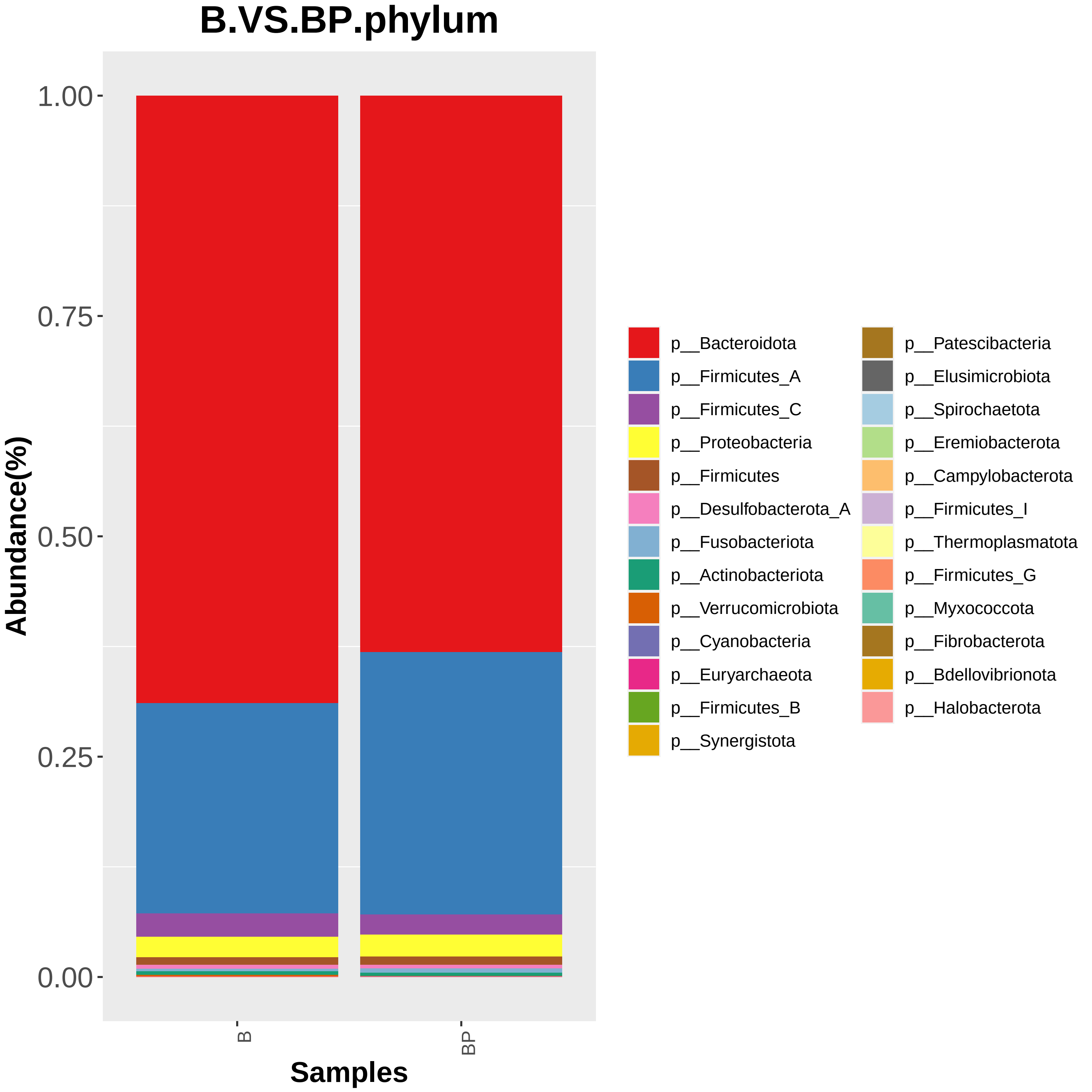
B.VS.BP.phylum
Abundance(%)
1.00
0.75
0.50
0.25
0.00
B
BP
Samples
p__Bacteroidota
p__Firmicutes_A
p__Firmicutes_C
p__Proteobacteria
p__Firmicutes
p__Desulfobacterota_A
p__Fusobacteriota
p__Actinobacteriota
p__Verrucomicrobiota
p__Cyanobacteria
p__Euryarchaeota
p__Firmicutes_B
p__Synergistota
p__Patescibacteria
p__Elusimicrobiota
p__Spirochaetota
p__Eremiobacterota
p__Campylobacterota
p__Firmicutes_I
p__Thermoplasmatota
p__Firmicutes_G
p__Myxococcota
p__Fibrobacterota
p__Bdellovibrionota
p__Halobacterota

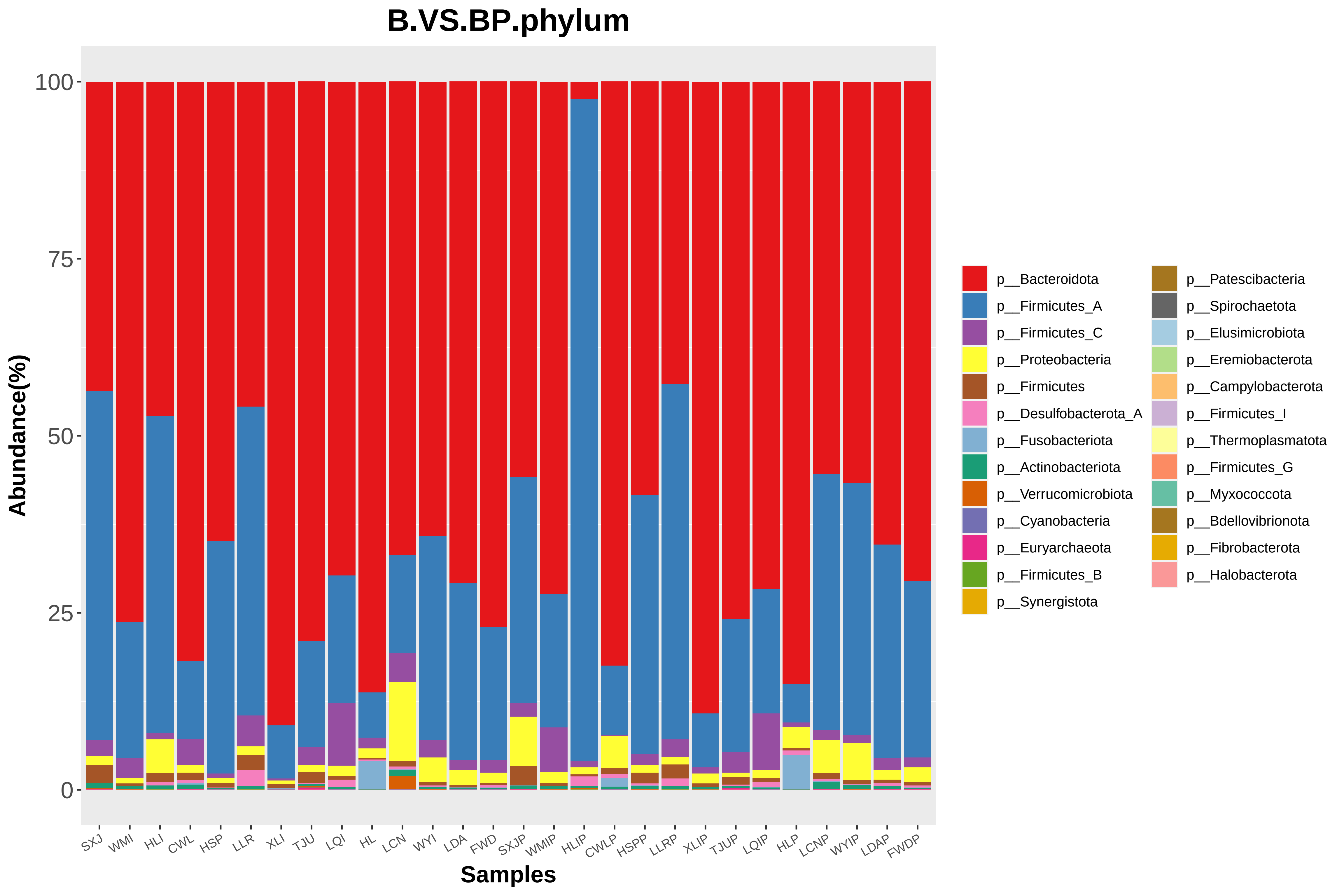
B.VS.BP.phylum
Abundance(%)
100
75
50
25
0
SXJ
WMI
HLI
CWL
HSP
LLR
XLI
TJU
LQI
HL
LCN
WYI
LDA
FWD
SXJP
WMIP
HLIP
CWLP
HSPP
LLRP
XLIP
TJUP
LQIP
HLP
LCNP
WYIP
LDAP
FWDP
Samples
p__Bacteroidota
p__Firmicutes_A
p__Firmicutes_C
p__Proteobacteria
p__Firmicutes
p__Desulfobacterota_A
p__Fusobacteriota
p__Actinobacteriota
p__Verrucomicrobiota
p__Cyanobacteria
p__Euryarchaeota
p__Firmicutes_B
p__Synergistota
p__Patescibacteria
p__Spirochaetota
p__Elusimicrobiota
p__Eremiobacterota
p__Campylobacterota
p__Firmicutes_I
p__Thermoplasmatota
p__Firmicutes_G
p__Myxococcota
p__Bdellovibrionota
p__Fibrobacterota
p__Halobacterota

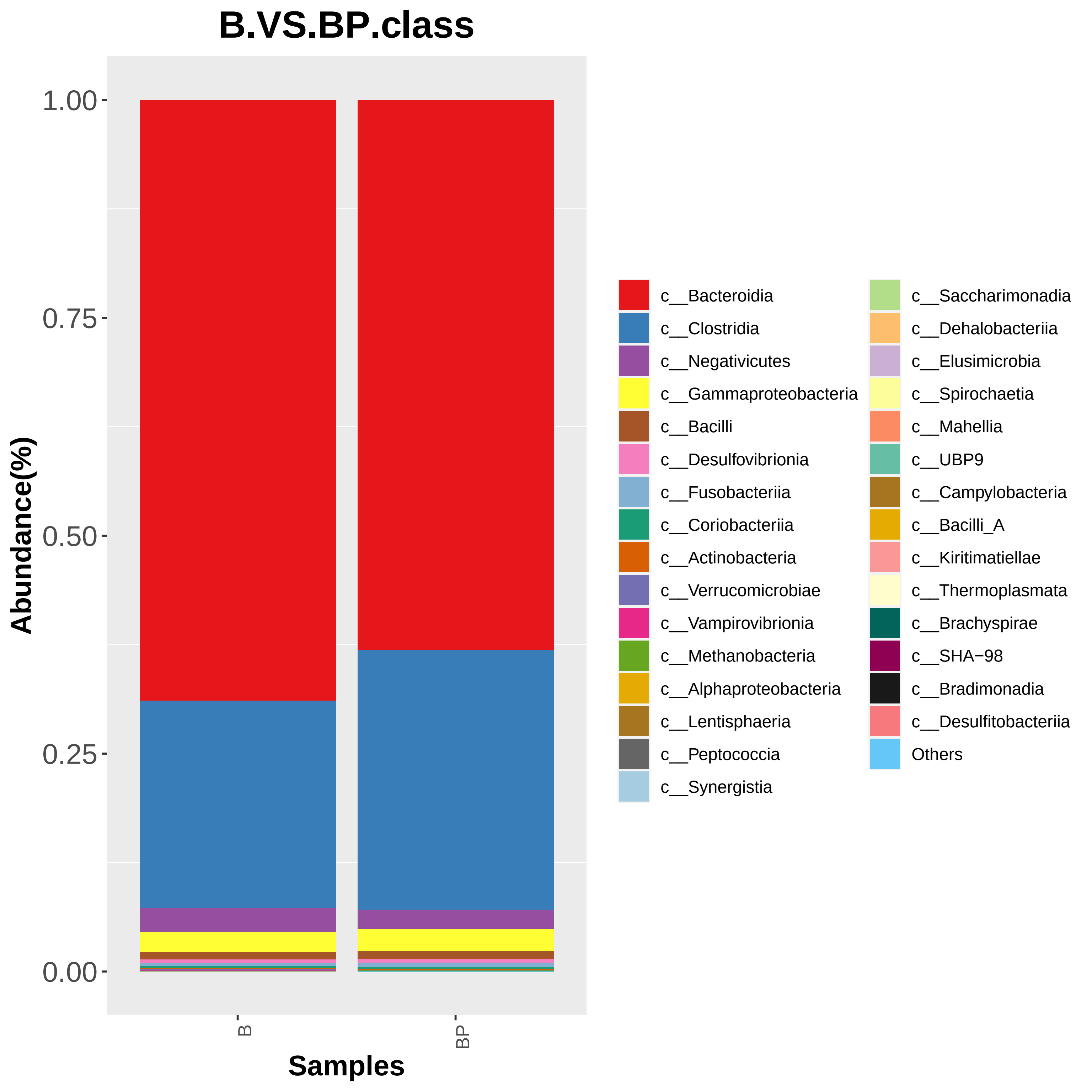
B.VS.BP.class
Abundance(%)
1.00
0.75
0.50
0.25
0.00
B
BP
Samples
c__Bacteroidia
c__Clostridia
c__Negativicutes
c__Gammaproteobacteria
c__Bacilli
c__Desulfovibrionia
c__Fusobacteriia
c__Coriobacteriia
c__Actinobacteria
c__Verrucomicrobiae
c__Vampirovibrionia
c__Methanobacteria
c__Alphaproteobacteria
c__Lentisphaeria
c__Peptococcia
c__Synergistia
c__Saccharimonadia
c__Dehalobacteriia
c__Elusimicrobia
c__Spirochaetia
c__Mahellia
c__UBP9
c__Campylobacteria
c__Bacilli_A
c__Kiritimatiellae
c__Thermoplasmata
c__Brachyspirae
c__SHA−98
c__Bradimonadia
c__Desulfitobacteriia
Others

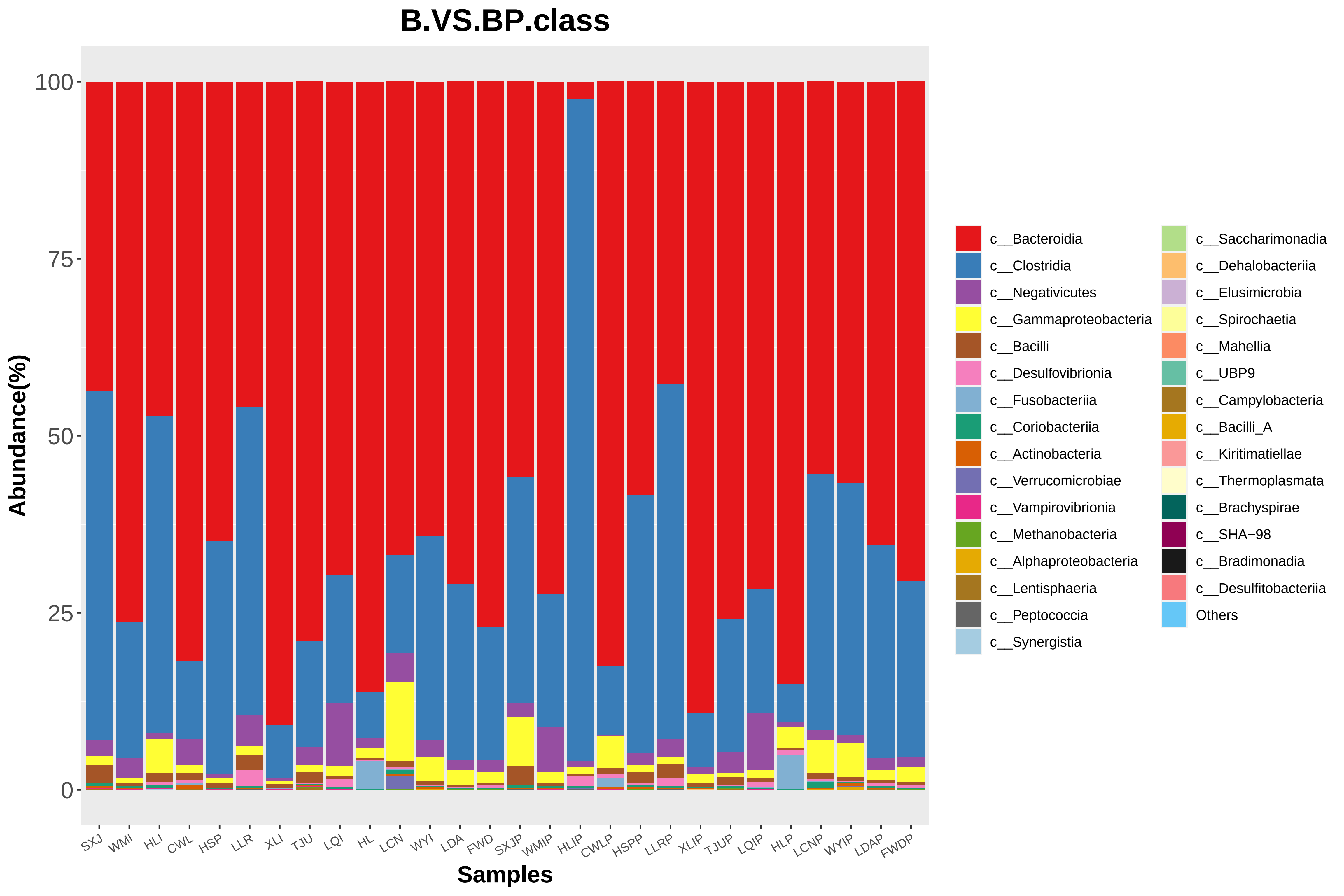

B.VS.BP.class
Abundance(%)
100
75
50
25
0
Samples
SXJ
WMI
HLI
CWL
HSP
LLR
XLI
TJU
LQI
HL
LCN
WYI
LDA
FWD
SXJP
WMIP
HLIP
CWLP
HSPP
LLRP
XLIP
TJUP
LQIP
HLP
LCNP
WYIP
LDAP
FWDP
c__Bacteroidia
c__Clostridia
c__Negativicutes
c__Gammaproteobacteria
c__Bacilli
c__Desulfovibrionia
c__Fusobacteriia
c__Coriobacteriia
c__Actinobacteria
c__Verrucomicrobiae
c__Vampirovibrionia
c__Methanobacteria
c__Alphaproteobacteria
c__Lentisphaeria
c__Peptococcia
c__Synergistia
c__Saccharimonadia
c__Dehalobacteriia
c__Elusimicrobia
c__Spirochaetia
c__Mahellia
c__UBP9
c__Campylobacteria
c__Bacilli_A
c__Kiritimatiellae
c__Thermoplasmata
c__Brachyspirae
c__SHA−98
c__Bradimonadia
c__Desulfitobacteriia
Others

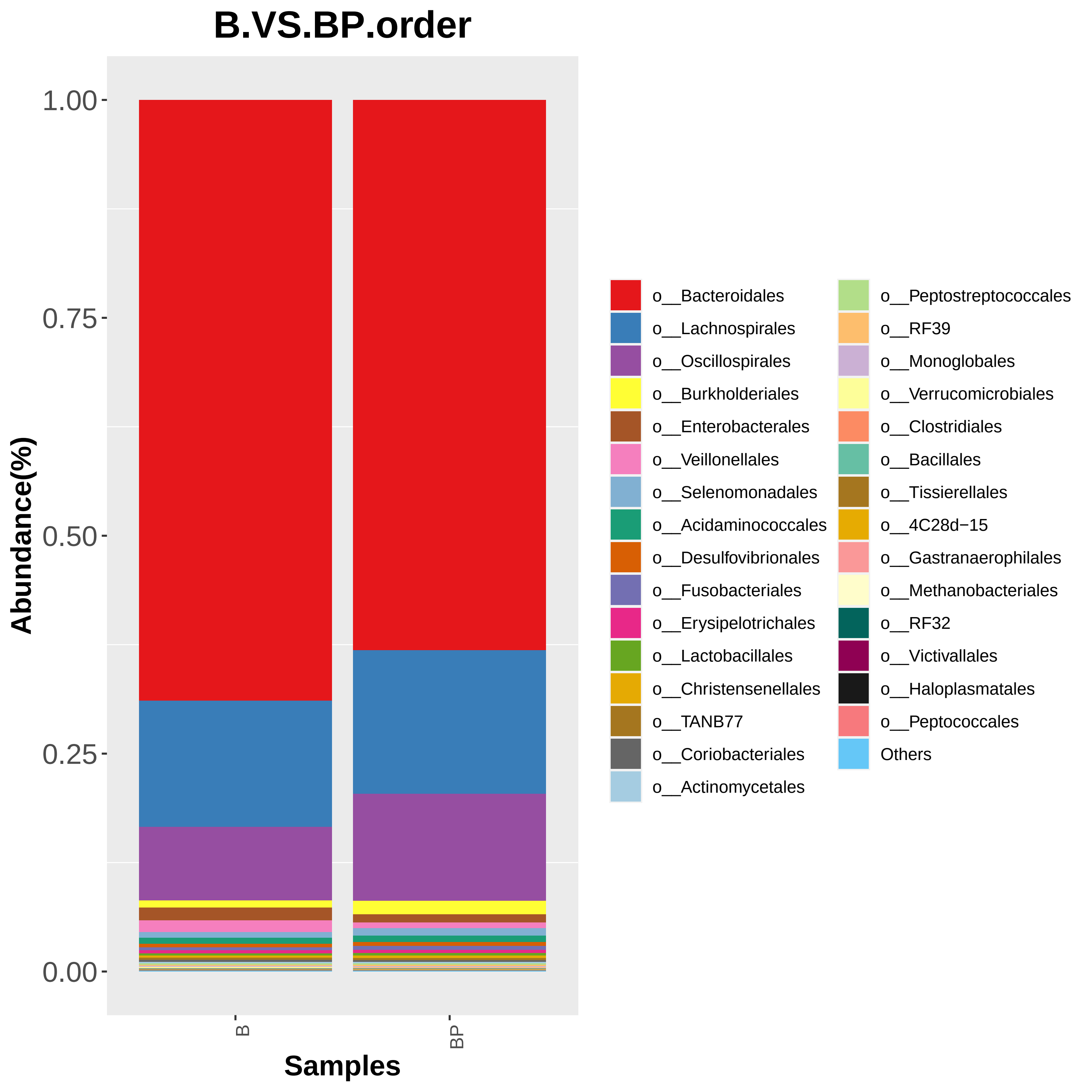
B.VS.BP.order
Abundance(%)
1.00
0.75
0.50
0.25
0.00
B
BP
Samples
o__Bacteroidales
o__Lachnospirales
o__Oscillospirales
o__Burkholderiales
o__Enterobacterales
o__Veillonellales
o__Selenomonadales
o__Acidaminococcales
o__Desulfovibrionales
o__Fusobacteriales
o__Erysipelotrichales
o__Lactobacillales
o__Christensenellales
o__TANB77
o__Coriobacteriales
o__Actinomycetales
o__Peptostreptococcales
o__RF39
o__Monoglobales
o__Verrucomicrobiales
o__Clostridiales
o__Bacillales
o__Tissierellales
o__4C28d−15
o__Gastranaerophilales
o__Methanobacteriales
o__RF32
o__Victivallales
o__Haloplasmatales
o__Peptococcales
Others

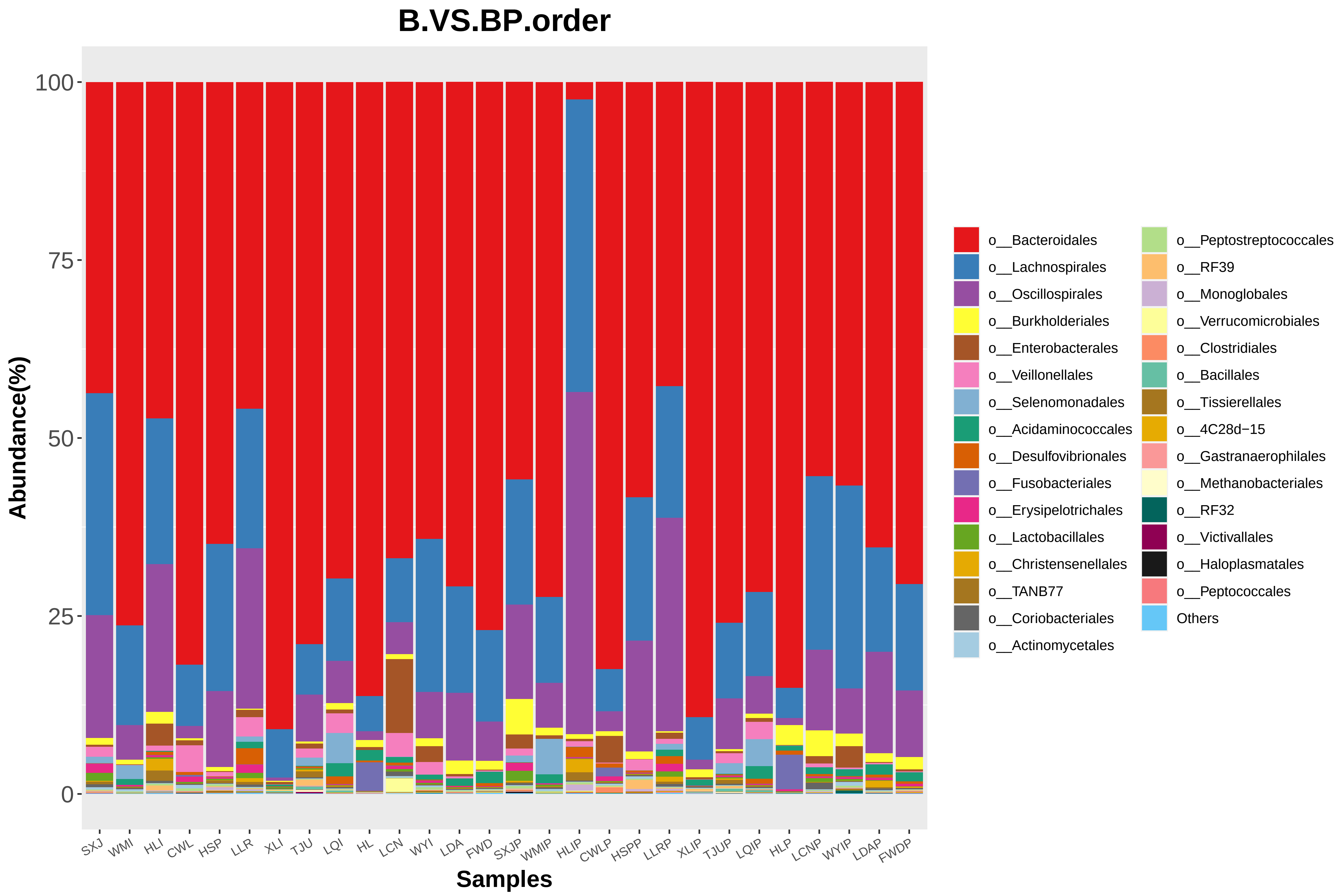
B.VS.BP.order
Abundance(%)
100
75
50
25
0
SXJ
WMI
HLI
CWL
HSP
LLR
XLI
TJU
LQI
HL
LCN
WYI
LDA
FWD
SXJP
WMIP
HLIP
CWLP
HSPP
LLRP
XLIP
TJUP
LQIP
HLP
LCNP
WYIP
LDAP
FWDP
Samples
o__Bacteroidales
o__Lachnospirales
o__Oscillospirales
o__Burkholderiales
o__Enterobacterales
o__Veillonellales
o__Selenomonadales
o__Acidaminococcales
o__Desulfovibrionales
o__Fusobacteriales
o__Erysipelotrichales
o__Lactobacillales
o__Christensenellales
o__TANB77
o__Coriobacteriales
o__Actinomycetales
o__Peptostreptococcales
o__RF39
o__Monoglobales
o__Verrucomicrobiales
o__Clostridiales
o__Bacillales
o__Tissierellales
o__4C28d−15
o__Gastranaerophilales
o__Methanobacteriales
o__RF32
o__Victivallales
o__Haloplasmatales
o__Peptococcales
Others

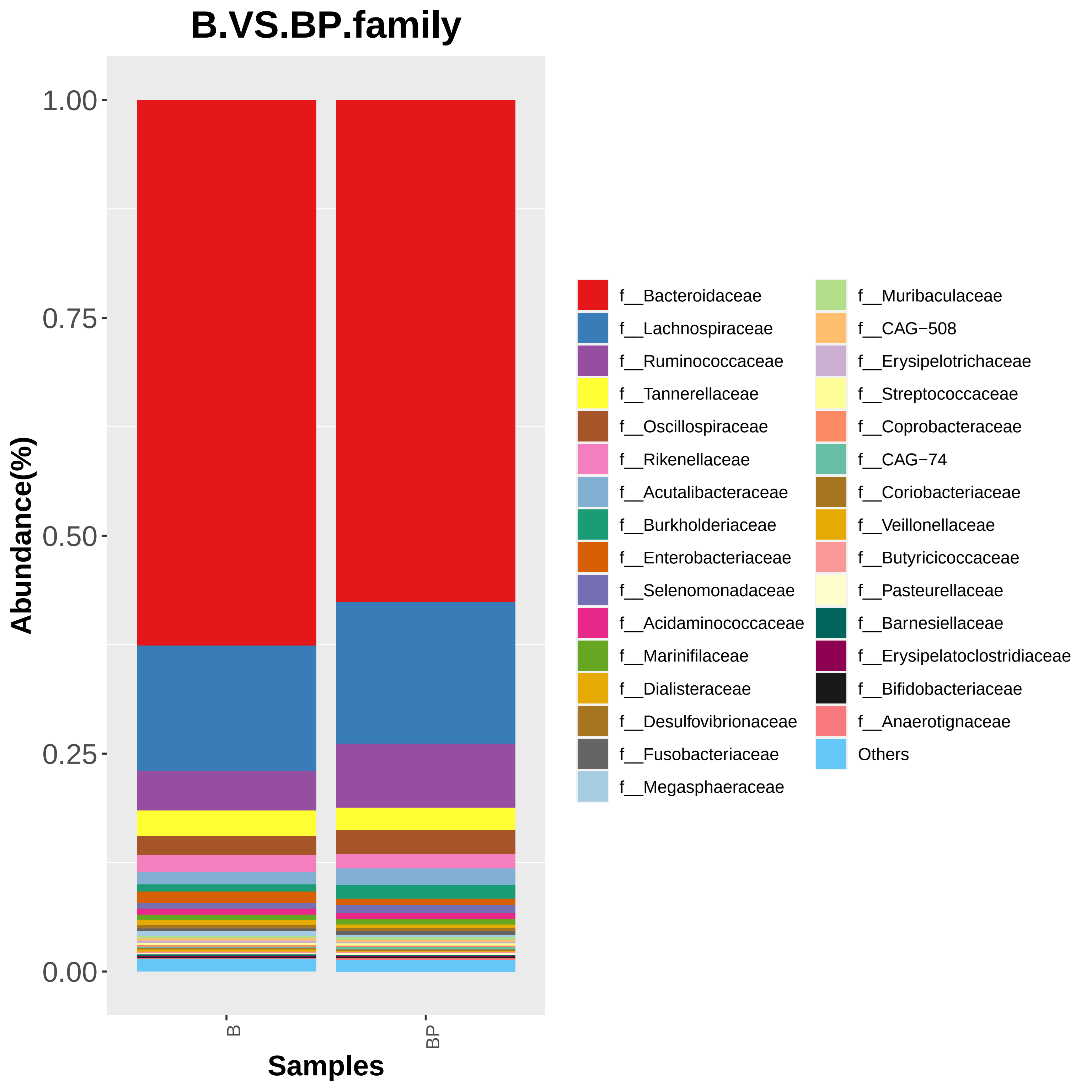
B.VS.BP.family
Abundance(%)
1.00
0.75
0.50
0.25
0.00
B
BP
Samples
f__Bacteroidaceae
f__Lachnospiraceae
f__Ruminococcaceae
f__Tannerellaceae
f__Oscillospiraceae
f__Rikenellaceae
f__Acutalibacteraceae
f__Burkholderiaceae
f__Enterobacteriaceae
f__Selenomonadaceae
f__Acidaminococcaceae
f__Marinifilaceae
f__Dialisteraceae
f__Desulfovibrionaceae
f__Fusobacteriaceae
f__Megasphaeraceae
f__Muribaculaceae
f__CAG−508
f__Erysipelotrichaceae
f__Streptococcaceae
f__Coprobacteraceae
f__CAG−74
f__Coriobacteriaceae
f__Veillonellaceae
f__Butyricicoccaceae
f__Pasteurellaceae
f__Barnesiellaceae
f__Erysipelatoclostridiaceae
f__Bifidobacteriaceae
f__Anaerotignaceae
Others

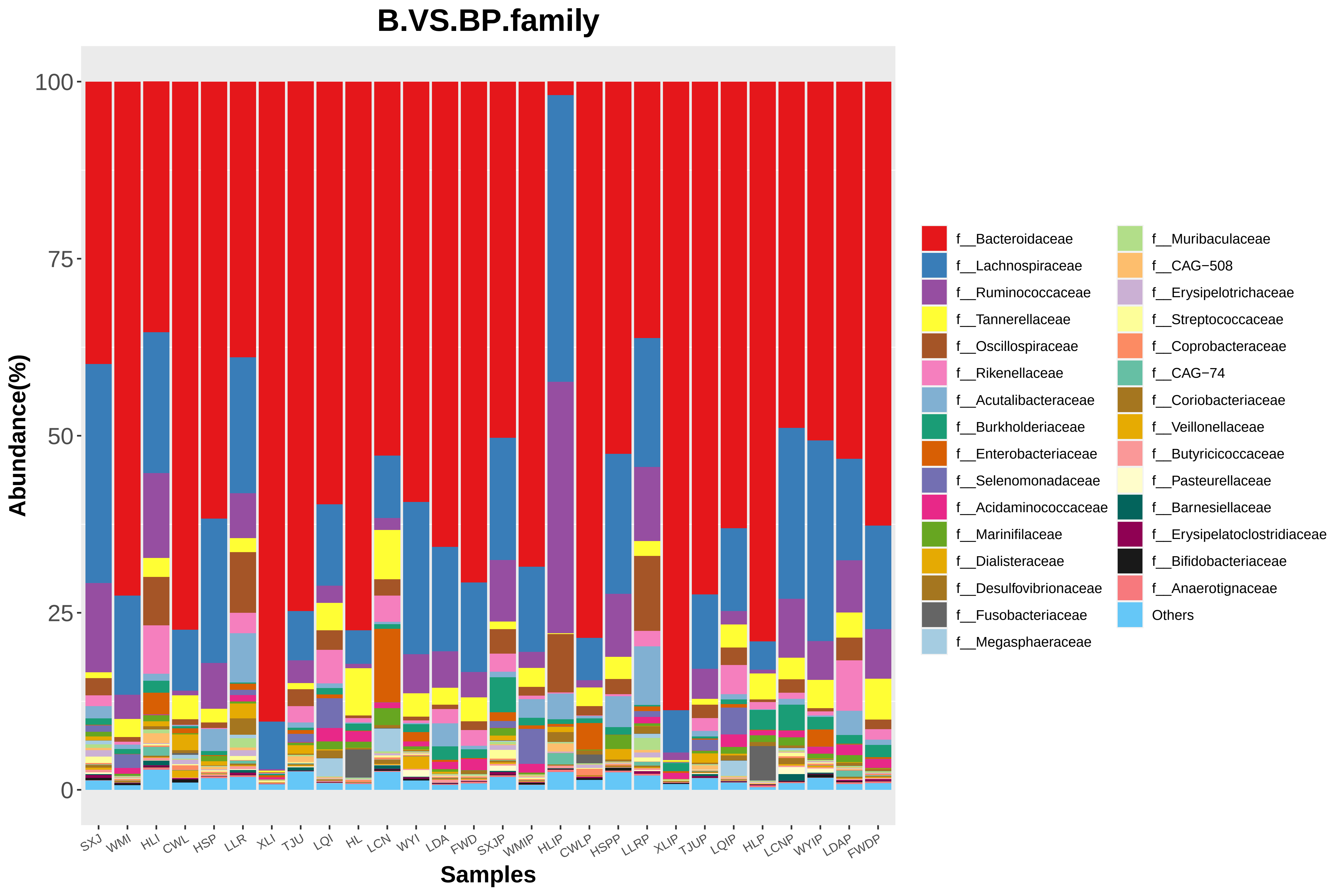

B.VS.BP.family
Abundance(%)
100
75
50
25
0
SXJ
WMI
HLI
CWL
HSP
LLR
XLI
TJU
LQI
HL
LCN
WYI
LDA
FWD
SXJP
WMIP
HLIP
CWLP
HSPP
LLRP
XLIP
TJUP
LQIP
HLP
LCNP
WYIP
LDAP
FWDP
Samples
f__Bacteroidaceae
f__Lachnospiraceae
f__Ruminococcaceae
f__Tannerellaceae
f__Oscillospiraceae
f__Rikenellaceae
f__Acutalibacteraceae
f__Burkholderiaceae
f__Enterobacteriaceae
f__Selenomonadaceae
f__Acidaminococcaceae
f__Marinifilaceae
f__Dialisteraceae
f__Desulfovibrionaceae
f__Fusobacteriaceae
f__Megasphaeraceae
f__Muribaculaceae
f__CAG−508
f__Erysipelotrichaceae
f__Streptococcaceae
f__Coprobacteraceae
f__CAG−74
f__Coriobacteriaceae
f__Veillonellaceae
f__Butyricicoccaceae
f__Pasteurellaceae
f__Barnesiellaceae
f__Erysipelatoclostridiaceae
f__Bifidobacteriaceae
f__Anaerotignaceae
Others

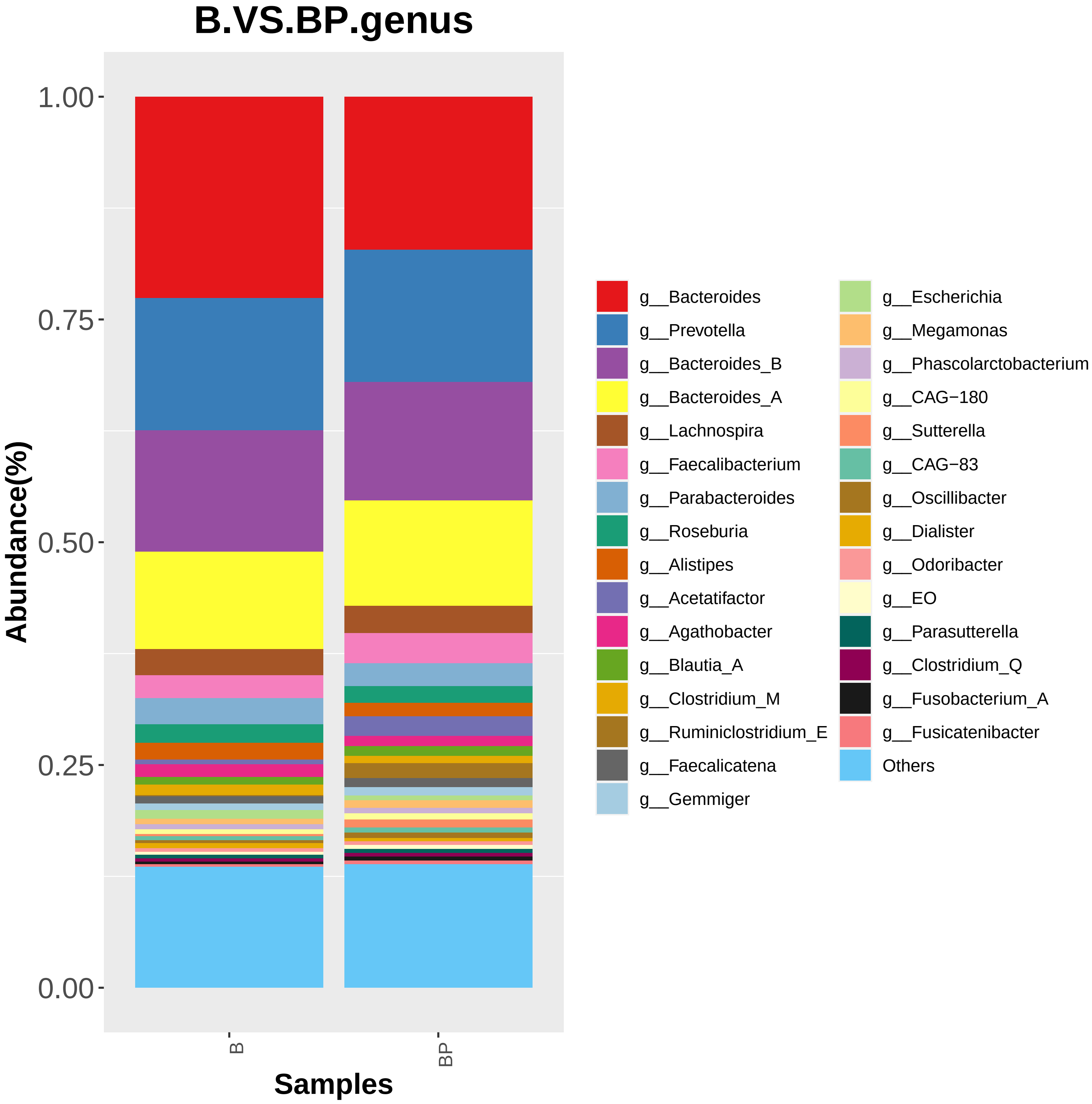
B.VS.BP.genus
Abundance(%)
1.00
0.75
0.50
0.25
0.00
B
BP
Samples
g__Bacteroides
g__Prevotella
g__Bacteroides_B
g__Bacteroides_A
g__Lachnospira
g__Faecalibacterium
g__Parabacteroides
g__Roseburia
g__Alistipes
g__Acetatifactor
g__Agathobacter
g__Blautia_A
g__Clostridium_M
g__Ruminiclostridium_E
g__Faecalicatena
g__Gemmiger
g__Escherichia
g__Megamonas
g__Phascolarctobacterium
g__CAG−180
g__Sutterella
g__CAG−83
g__Oscillibacter
g__Dialister
g__Odoribacter
g__EO
g__Parasutterella
g__Clostridium_Q
g__Fusobacterium_A
g__Fusicatenibacter
Others

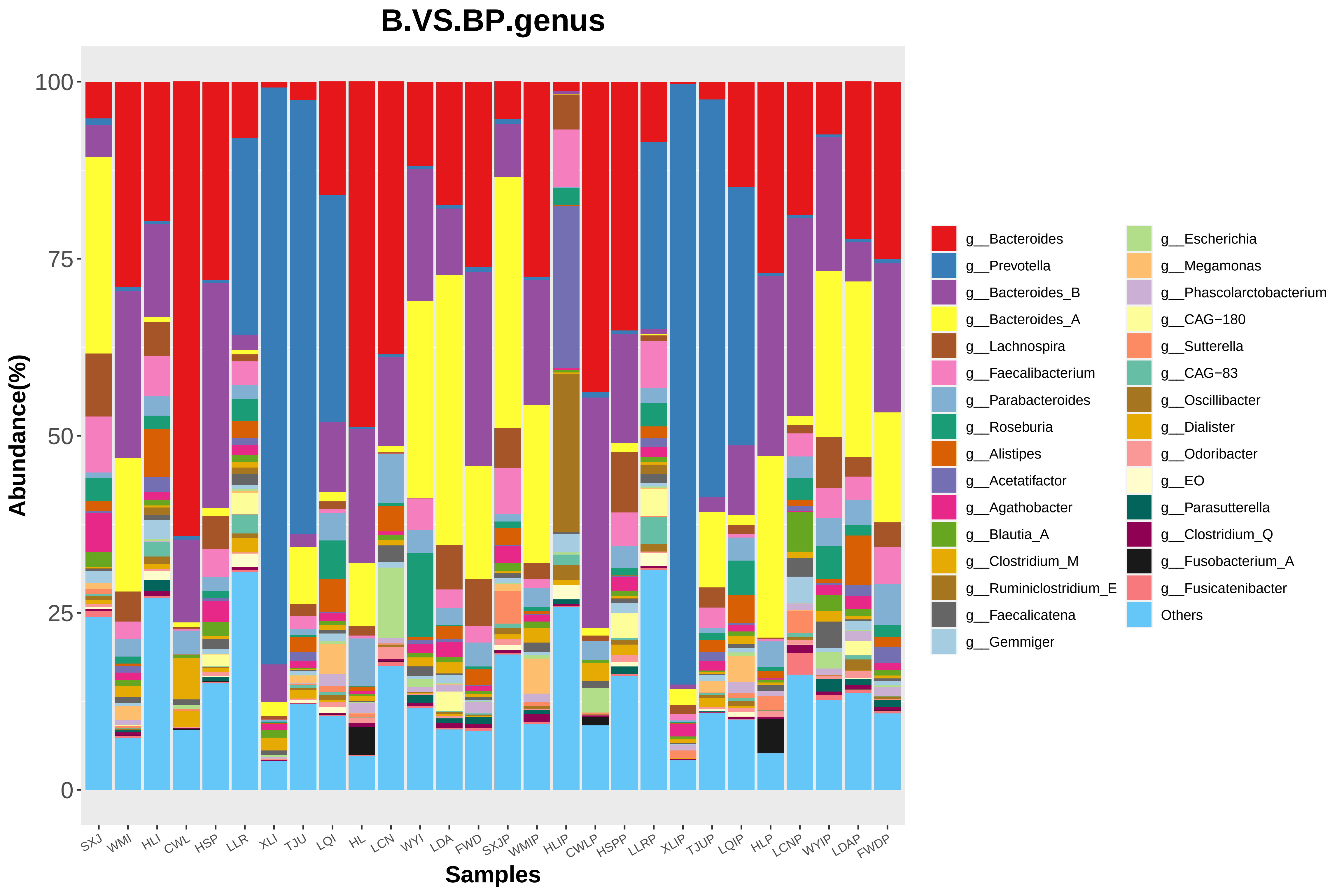

B.VS.BP.genus
Abundance(%)
100
75
50
25
0
SXJ
WMI
HLI
CWL
HSP
LLR
XLI
TJU
LQI
HL
LCN
WYI
LDA
FWD
SXJP
WMIP
HLIP
CWLP
HSPP
LLRP
XLIP
TJUP
LQIP
HLP
LCNP
WYIP
LDAP
FWDP
Samples
g__Bacteroides
g__Prevotella
g__Bacteroides_B
g__Bacteroides_A
g__Lachnospira
g__Faecalibacterium
g__Parabacteroides
g__Roseburia
g__Alistipes
g__Acetatifactor
g__Agathobacter
g__Blautia_A
g__Clostridium_M
g__Ruminiclostridium_E
g__Faecalicatena
g__Gemmiger
g__Escherichia
g__Megamonas
g__Phascolarctobacterium
g__CAG−180
g__Sutterella
g__CAG−83
g__Oscillibacter
g__Dialister
g__Odoribacter
g__EO
g__Parasutterella
g__Clostridium_Q
g__Fusobacterium_A
g__Fusicatenibacter
Others

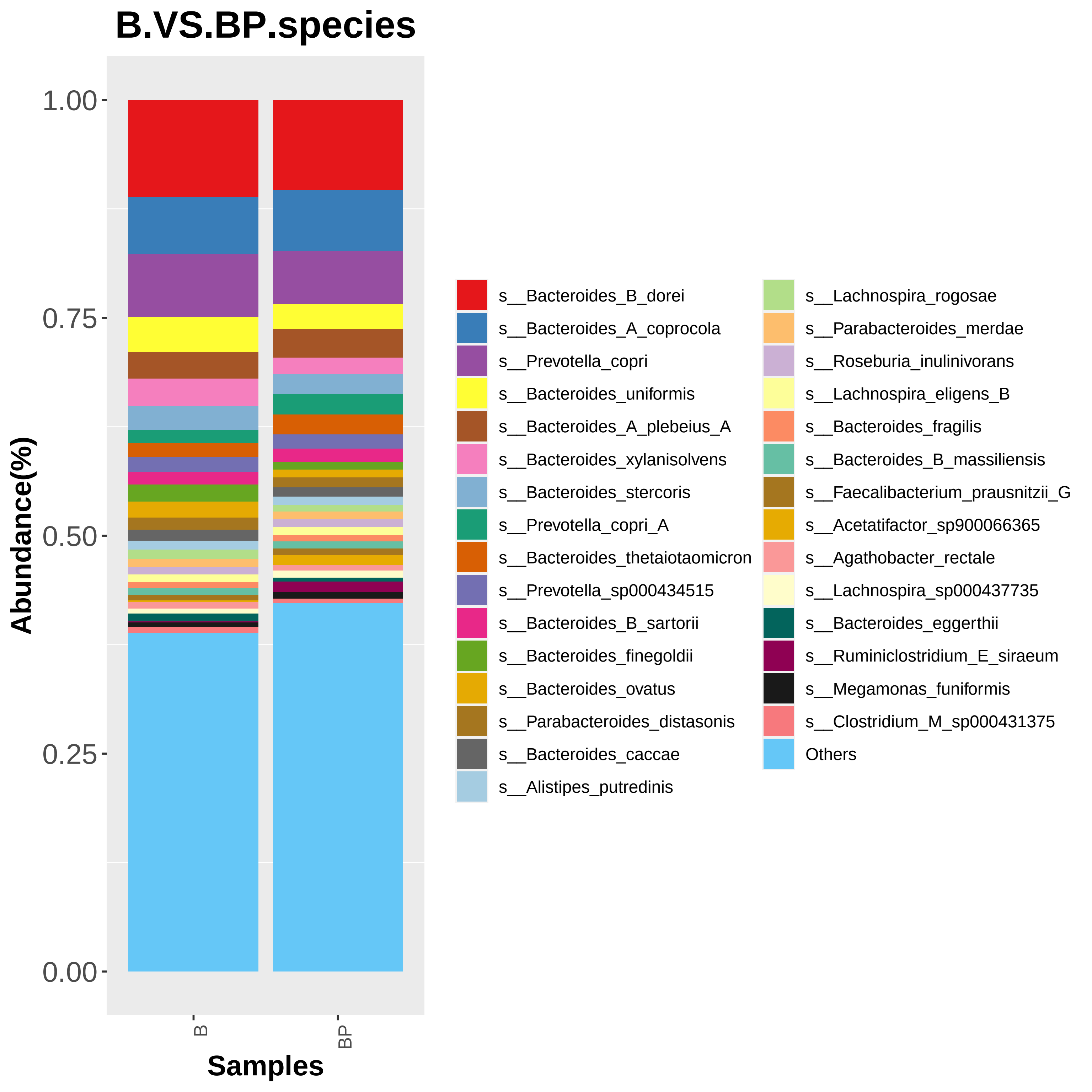
B.VS.BP.species
Abundance(%)
1.00
0.75
0.50
0.25
0.00
B
BP
Samples
s__Bacteroides_B_dorei
s__Bacteroides_A_coprocola
s__Prevotella_copri
s__Bacteroides_uniformis
s__Bacteroides_A_plebeius_A
s__Bacteroides_xylanisolvens
s__Bacteroides_stercoris
s__Prevotella_copri_A
s__Bacteroides_thetaiotaomicron
s__Prevotella_sp000434515
s__Bacteroides_B_sartorii
s__Bacteroides_finegoldii
s__Bacteroides_ovatus
s__Parabacteroides_distasonis
s__Bacteroides_caccae
s__Alistipes_putredinis
s__Lachnospira_rogosae
s__Parabacteroides_merdae
s__Roseburia_inulinivorans
s__Lachnospira_eligens_B
s__Bacteroides_fragilis
s__Bacteroides_B_massiliensis
s__Faecalibacterium_prausnitzii_G
s__Acetatifactor_sp900066365
s__Agathobacter_rectale
s__Lachnospira_sp000437735
s__Bacteroides_eggerthii
s__Ruminiclostridium_E_siraeum
s__Megamonas_funiformis
s__Clostridium_M_sp000431375
Others

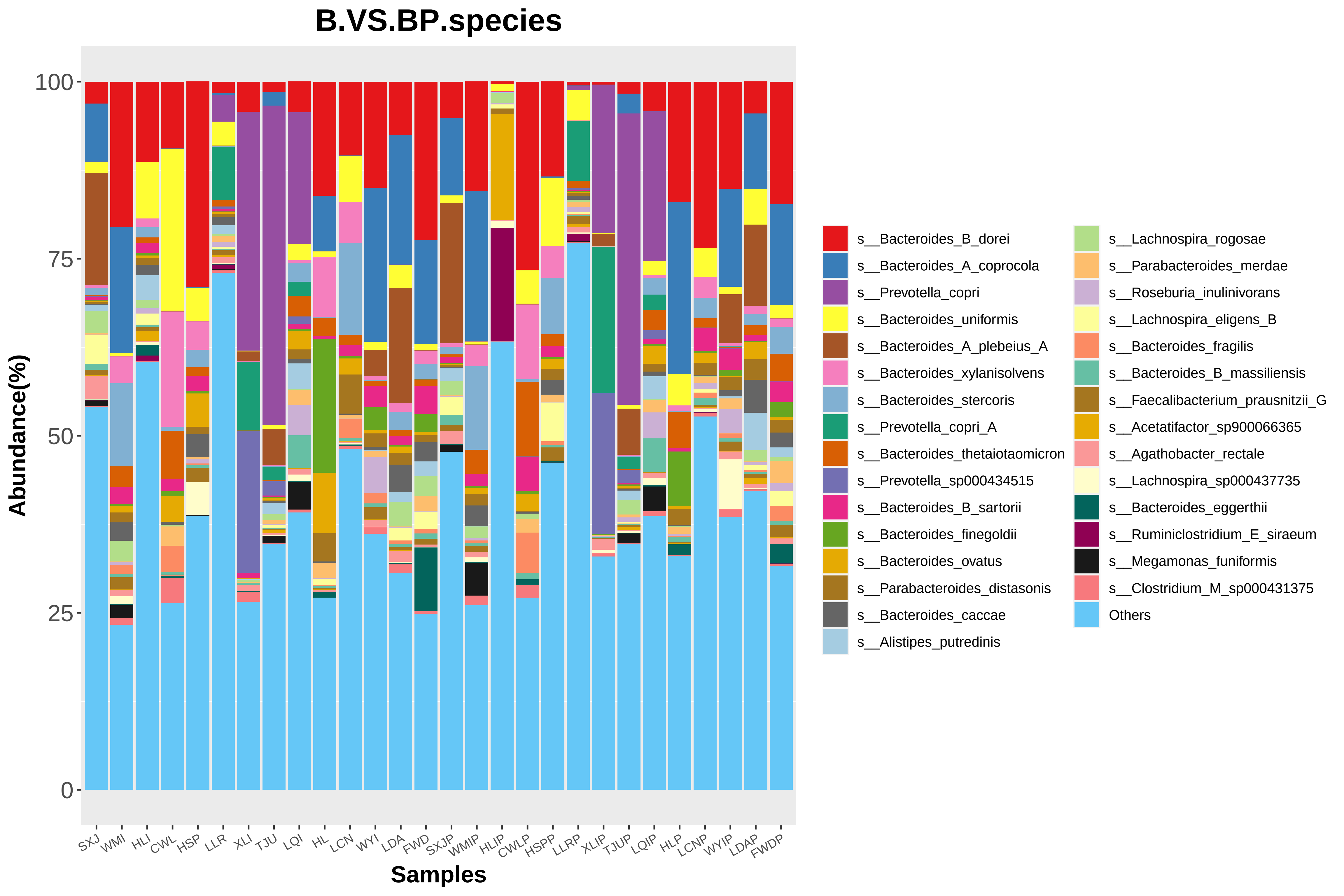
B.VS.BP.species
Abundance(%)
100
75
50
25
0
SXJ
WMI
HLI
CWL
HSP
LLR
XLI
TJU
LQI
HL
LCN
WYI
LDA
FWD
SXJP
WMIP
HLIP
CWLP
HSPP
LLRP
XLIP
TJUP
LQIP
HLP
LCNP
WYIP
LDAP
FWDP
Samples
s__Bacteroides_B_dorei
s__Bacteroides_A_coprocola
s__Prevotella_copri
s__Bacteroides_uniformis
s__Bacteroides_A_plebeius_A
s__Bacteroides_xylanisolvens
s__Bacteroides_stercoris
s__Prevotella_copri_A
s__Bacteroides_thetaiotaomicron
s__Prevotella_sp000434515
s__Bacteroides_B_sartorii
s__Bacteroides_finegoldii
s__Bacteroides_ovatus
s__Parabacteroides_distasonis
s__Bacteroides_caccae
s__Alistipes_putredinis
s__Lachnospira_rogosae
s__Parabacteroides_merdae
s__Roseburia_inulinivorans
s__Lachnospira_eligens_B
s__Bacteroides_fragilis
s__Bacteroides_B_massiliensis
s__Faecalibacterium_prausnitzii_G
s__Acetatifactor_sp900066365
s__Agathobacter_rectale
s__Lachnospira_sp000437735
s__Bacteroides_eggerthii
s__Ruminiclostridium_E_siraeum
s__Megamonas_funiformis
s__Clostridium_M_sp000431375
Others

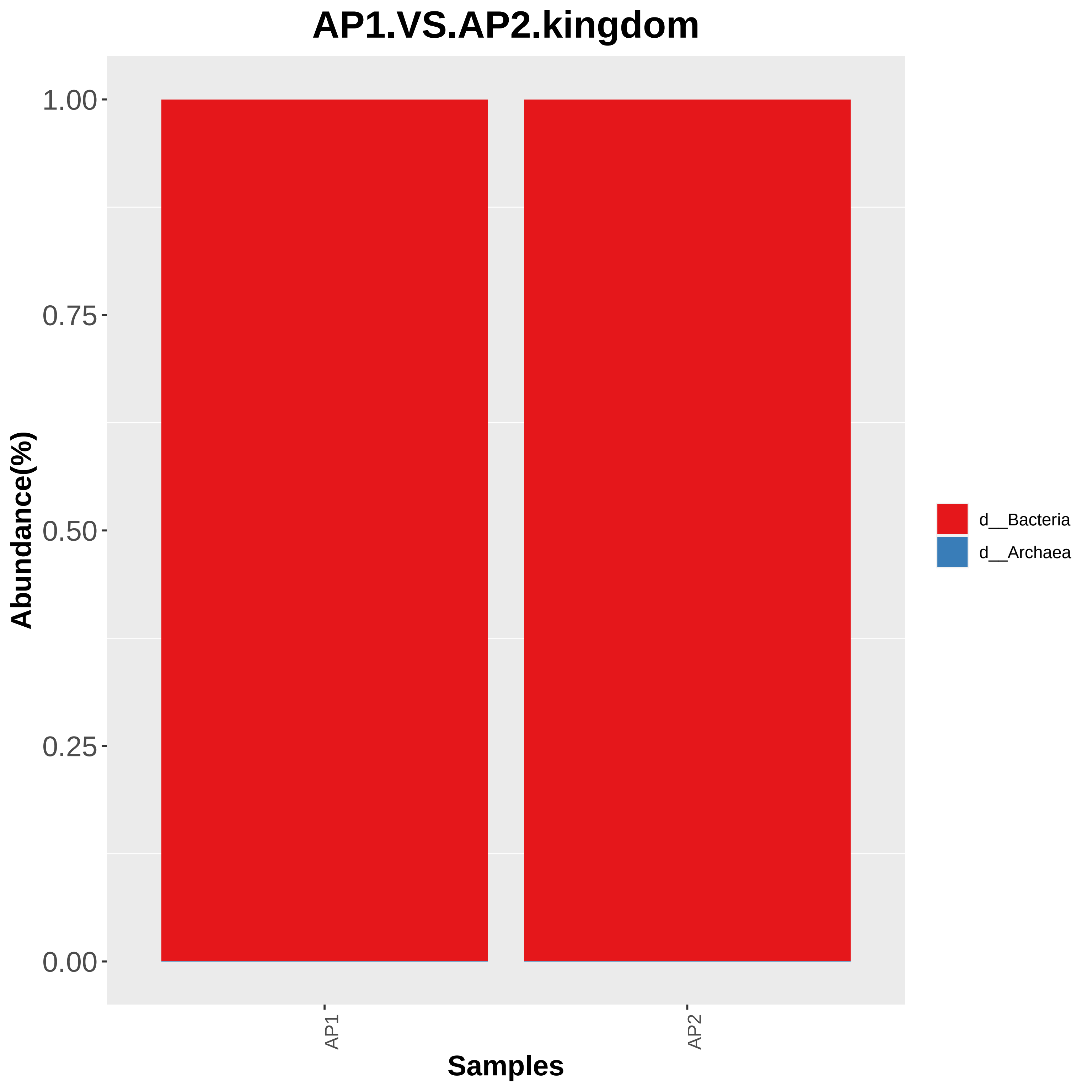
AP1.VS.AP2.kingdom
Abundance(%)
1.00
0.75
0.50
0.25
0.00
AP1
AP2
Samples
d__Bacteria
d__Archaea

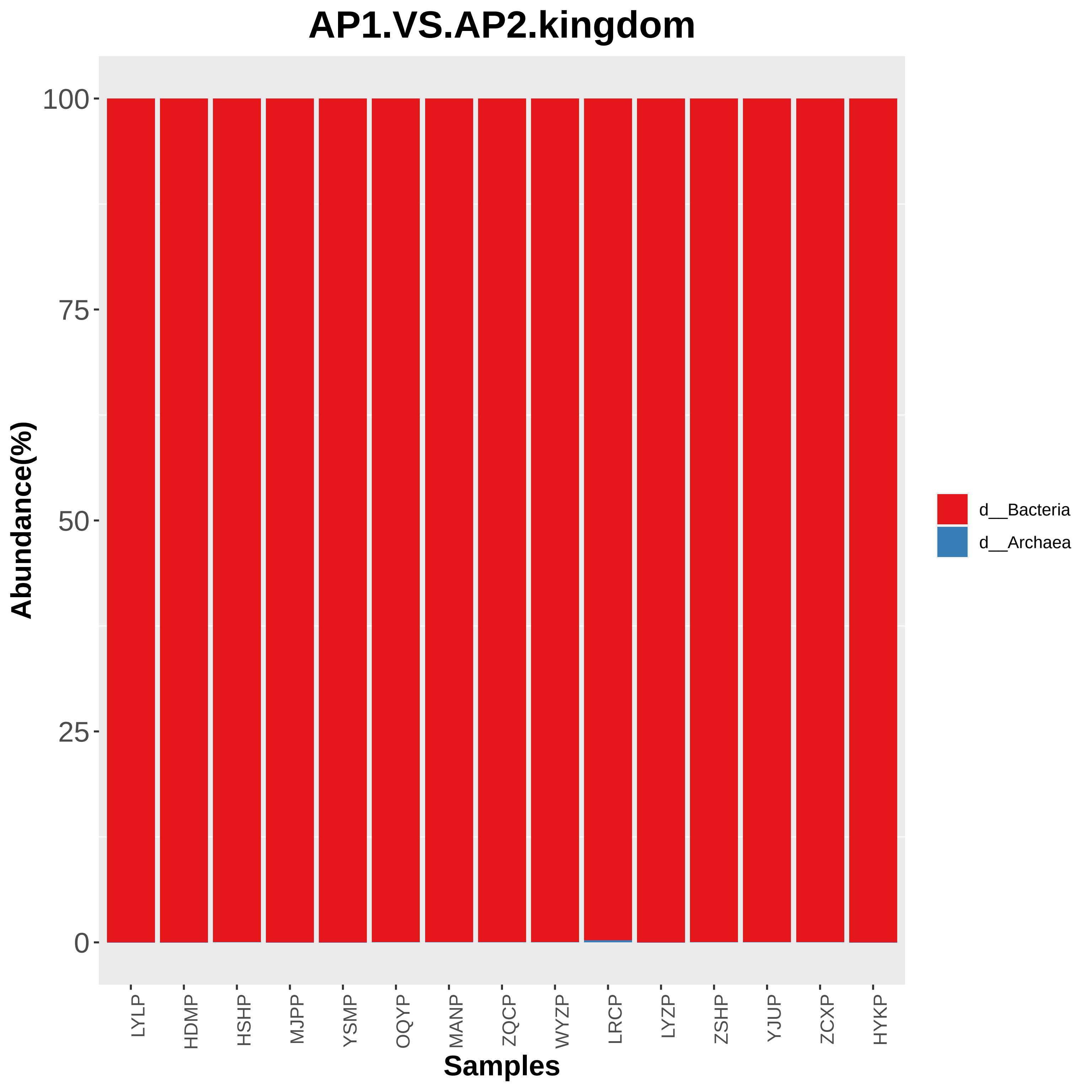
AP1.VS.AP2.kingdom
Abundance(%)
100
75
50
25
0
LYLP
HDMP
HSHP
MJPP
YSMP
OQYP
MANP
ZQCP
WYZP
LRCP
LYZP
ZSHP
YJUP
ZCXP
HYKP
Samples
d__Bacteria
d__Archaea

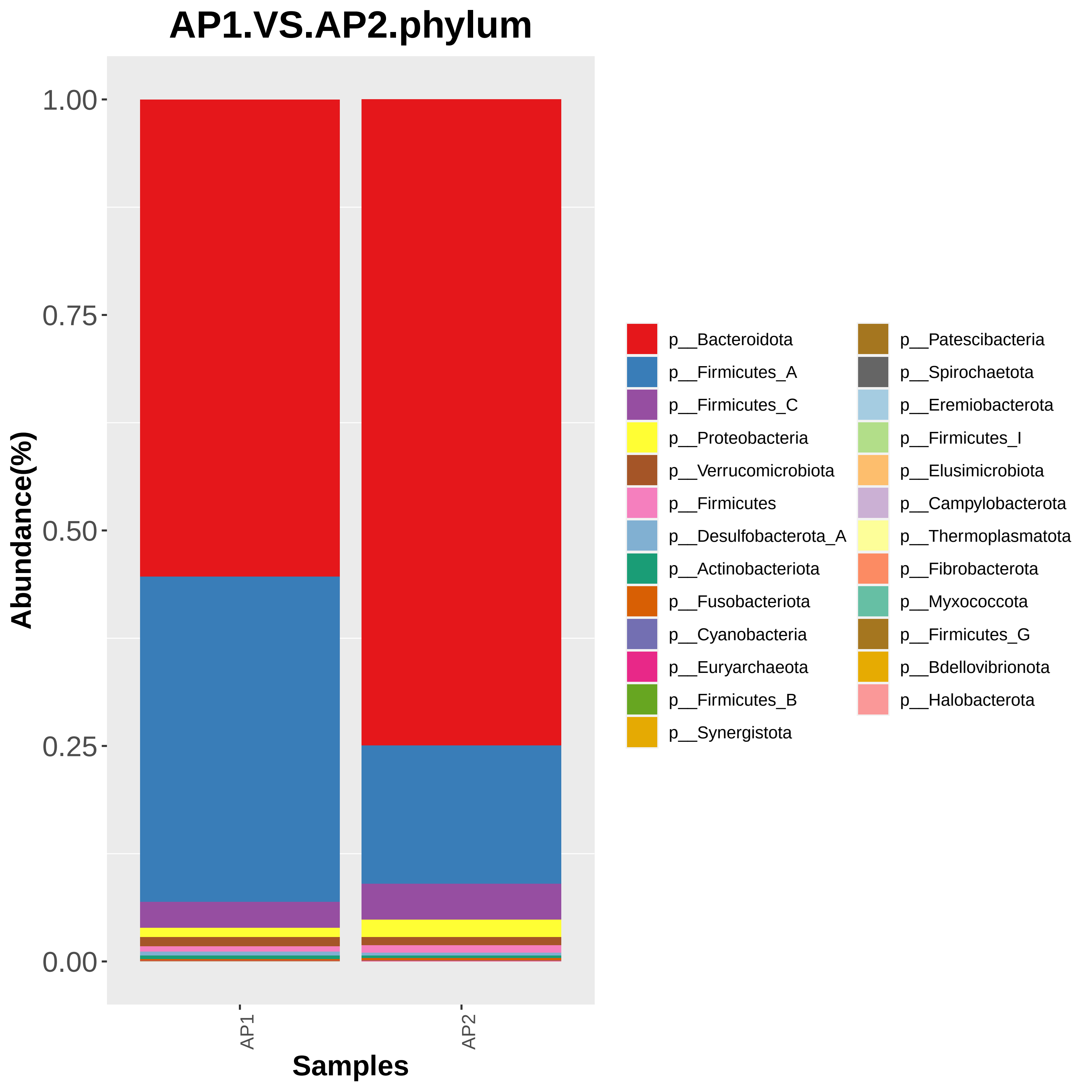
AP1.VS.AP2.phylum
Abundance(%)
1.00
0.75
0.50
0.25
0.00
AP1
AP2
Samples
p__Bacteroidota
p__Firmicutes_A
p__Firmicutes_C
p__Proteobacteria
p__Verrucomicrobiota
p__Firmicutes
p__Desulfobacterota_A
p__Actinobacteriota
p__Fusobacteriota
p__Cyanobacteria
p__Euryarchaeota
p__Firmicutes_B
p__Synergistota
p__Patescibacteria
p__Spirochaetota
p__Eremiobacterota
p__Firmicutes_I
p__Elusimicrobiota
p__Campylobacterota
p__Thermoplasmatota
p__Fibrobacterota
p__Myxococcota
p__Firmicutes_G
p__Bdellovibrionota
p__Halobacterota

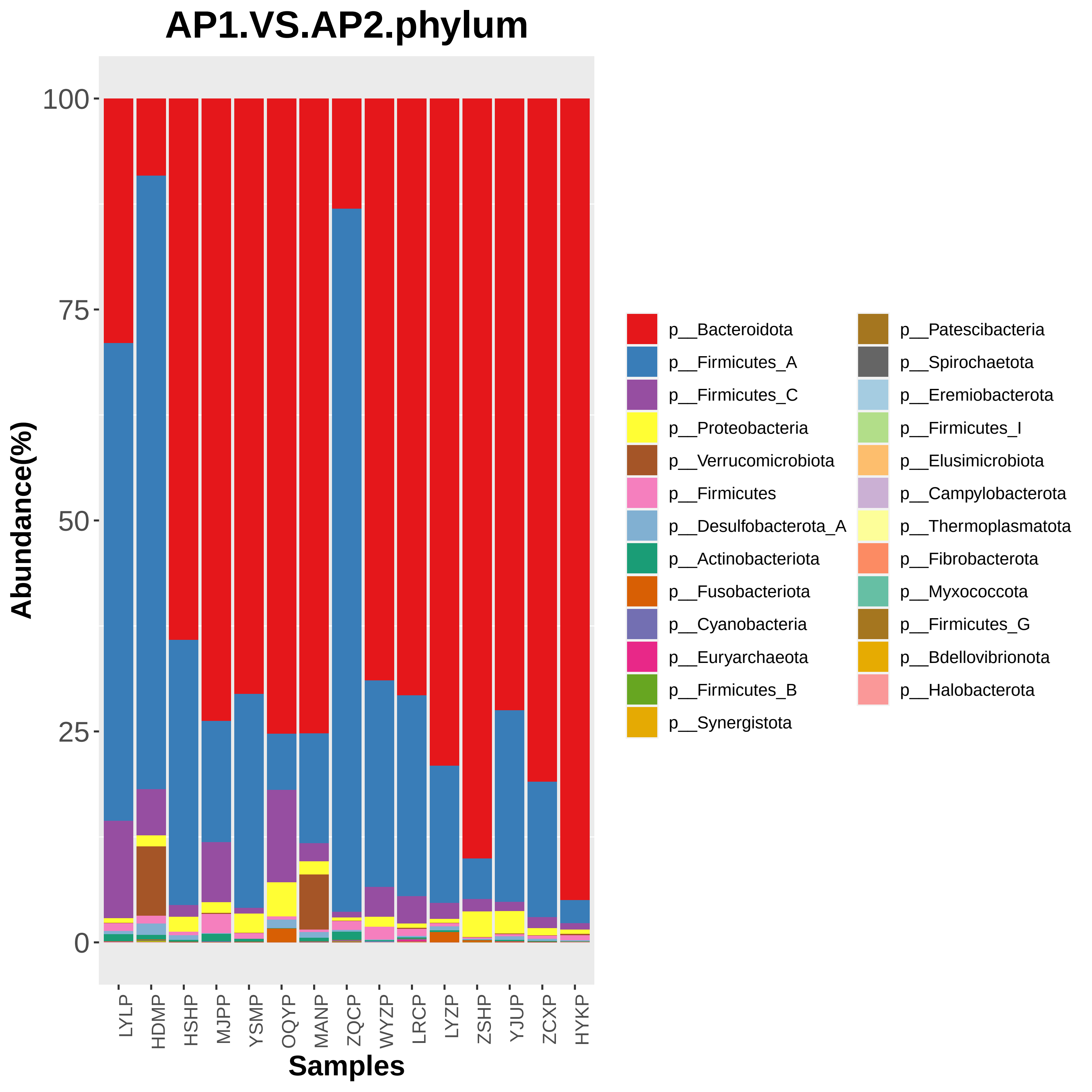
AP1.VS.AP2.phylum
Abundance(%)
100
75
50
25
0
LYLP
HDMP
HSHP
MJPP
YSMP
OQYP
MANP
ZQCP
WYZP
LRCP
LYZP
ZSHP
YJUP
ZCXP
HYKP
Samples
p__Bacteroidota
p__Firmicutes_A
p__Firmicutes_C
p__Proteobacteria
p__Verrucomicrobiota
p__Firmicutes
p__Desulfobacterota_A
p__Actinobacteriota
p__Fusobacteriota
p__Cyanobacteria
p__Euryarchaeota
p__Firmicutes_B
p__Synergistota
p__Patescibacteria
p__Spirochaetota
p__Eremiobacterota
p__Firmicutes_I
p__Elusimicrobiota
p__Campylobacterota
p__Thermoplasmatota
p__Fibrobacterota
p__Myxococcota
p__Firmicutes_G
p__Bdellovibrionota
p__Halobacterota

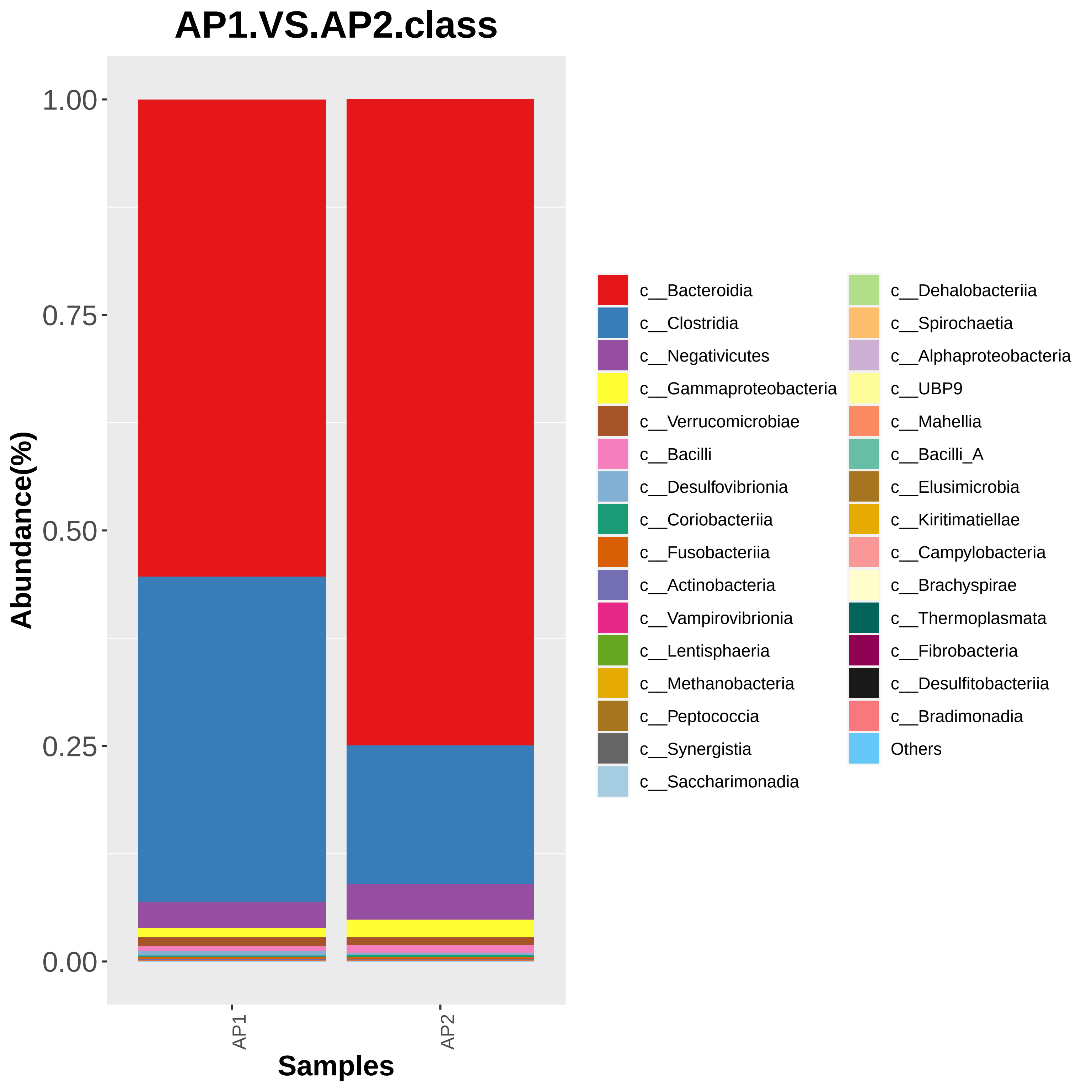

AP1.VS.AP2.class
Abundance(%)
1.00
0.75
0.50
0.25
0.00
AP1
AP2
Samples
c__Bacteroidia
c__Clostridia
c__Negativicutes
c__Gammaproteobacteria
c__Verrucomicrobiae
c__Bacilli
c__Desulfovibrionia
c__Coriobacteriia
c__Fusobacteriia
c__Actinobacteria
c__Vampirovibrionia
c__Lentisphaeria
c__Methanobacteria
c__Peptococcia
c__Synergistia
c__Saccharimonadia
c__Dehalobacteriia
c__Spirochaetia
c__Alphaproteobacteria
c__UBP9
c__Mahellia
c__Bacilli_A
c__Elusimicrobia
c__Kiritimatiellae
c__Campylobacteria
c__Brachyspirae
c__Thermoplasmata
c__Fibrobacteria
c__Desulfitobacteriia
c__Bradimonadia
Others

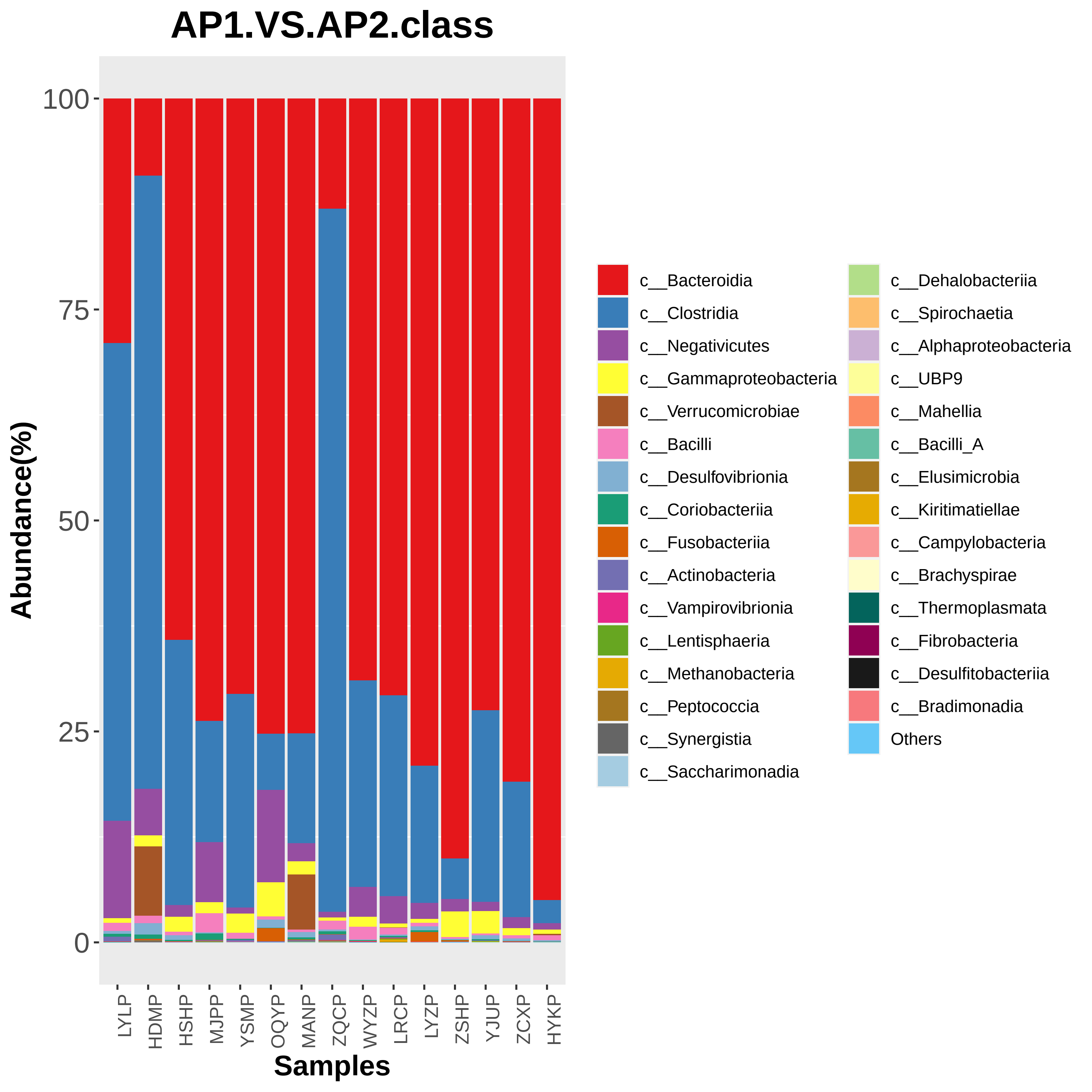

AP1.VS.AP2.class
Abundance(%)
100
75
50
25
0
LYLP
HDMP
HSHP
MJPP
YSMP
OQYP
MANP
ZQCP
WYZP
LRCP
LYZP
ZSHP
YJUP
ZCXP
HYKP
Samples
c__Bacteroidia
c__Clostridia
c__Negativicutes
c__Gammaproteobacteria
c__Verrucomicrobiae
c__Bacilli
c__Desulfovibrionia
c__Coriobacteriia
c__Fusobacteriia
c__Actinobacteria
c__Vampirovibrionia
c__Lentisphaeria
c__Methanobacteria
c__Peptococcia
c__Synergistia
c__Saccharimonadia
c__Dehalobacteriia
c__Spirochaetia
c__Alphaproteobacteria
c__UBP9
c__Mahellia
c__Bacilli_A
c__Elusimicrobia
c__Kiritimatiellae
c__Campylobacteria
c__Brachyspirae
c__Thermoplasmata
c__Fibrobacteria
c__Desulfitobacteriia
c__Bradimonadia
Others

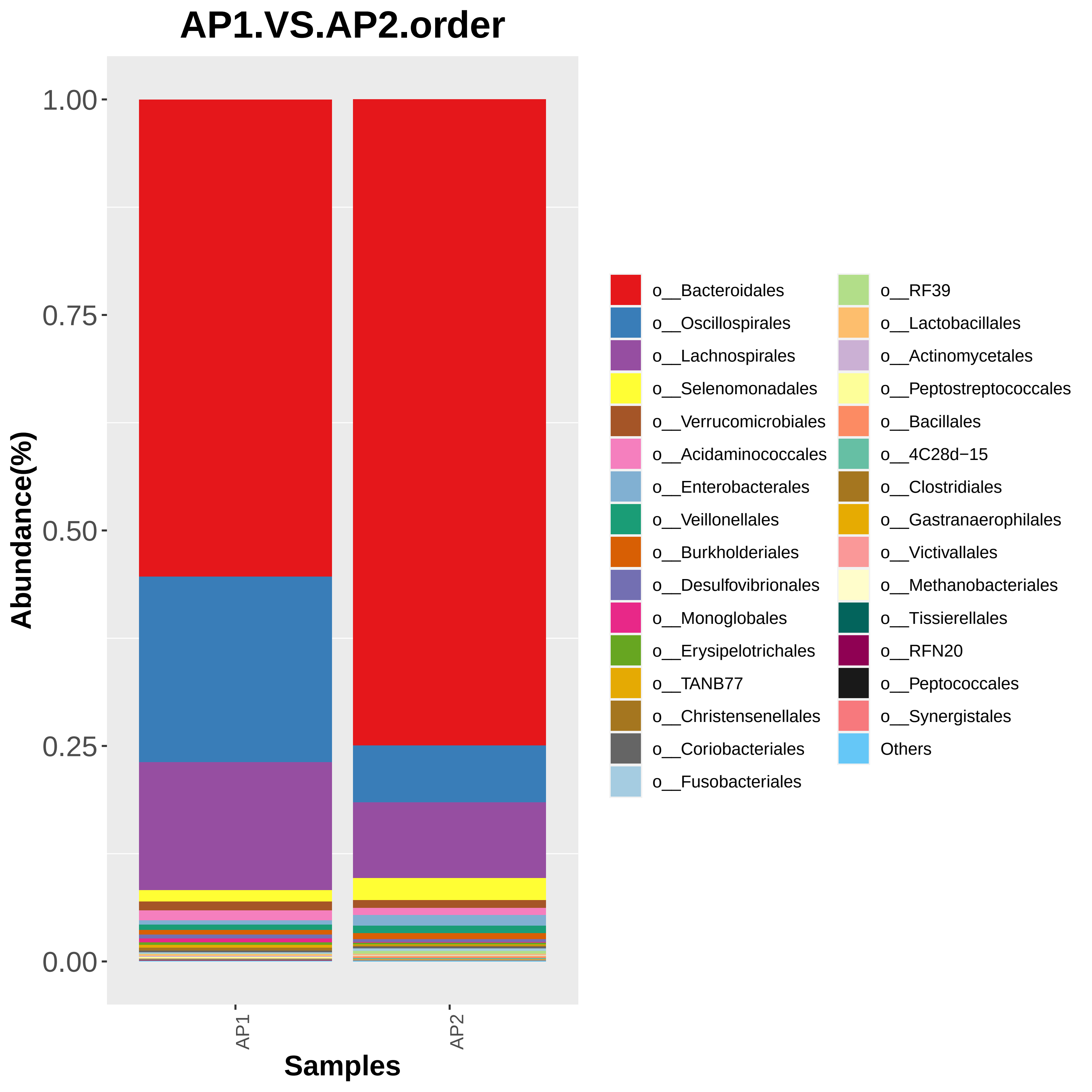

AP1.VS.AP2.order
Abundance(%)
1.00
0.75
0.50
0.25
0.00
AP1
AP2
Samples
o__Bacteroidales
o__Oscillospirales
o__Lachnospirales
o__Selenomonadales
o__Verrucomicrobiales
o__Acidaminococcales
o__Enterobacterales
o__Veillonellales
o__Burkholderiales
o__Desulfovibrionales
o__Monoglobales
o__Erysipelotrichales
o__TANB77
o__Christensenellales
o__Coriobacteriales
o__Fusobacteriales
o__RF39
o__Lactobacillales
o__Actinomycetales
o__Peptostreptococcales
o__Bacillales
o__4C28d−15
o__Clostridiales
o__Gastranaerophilales
o__Victivallales
o__Methanobacteriales
o__Tissierellales
o__RFN20
o__Peptococcales
o__Synergistales
Others

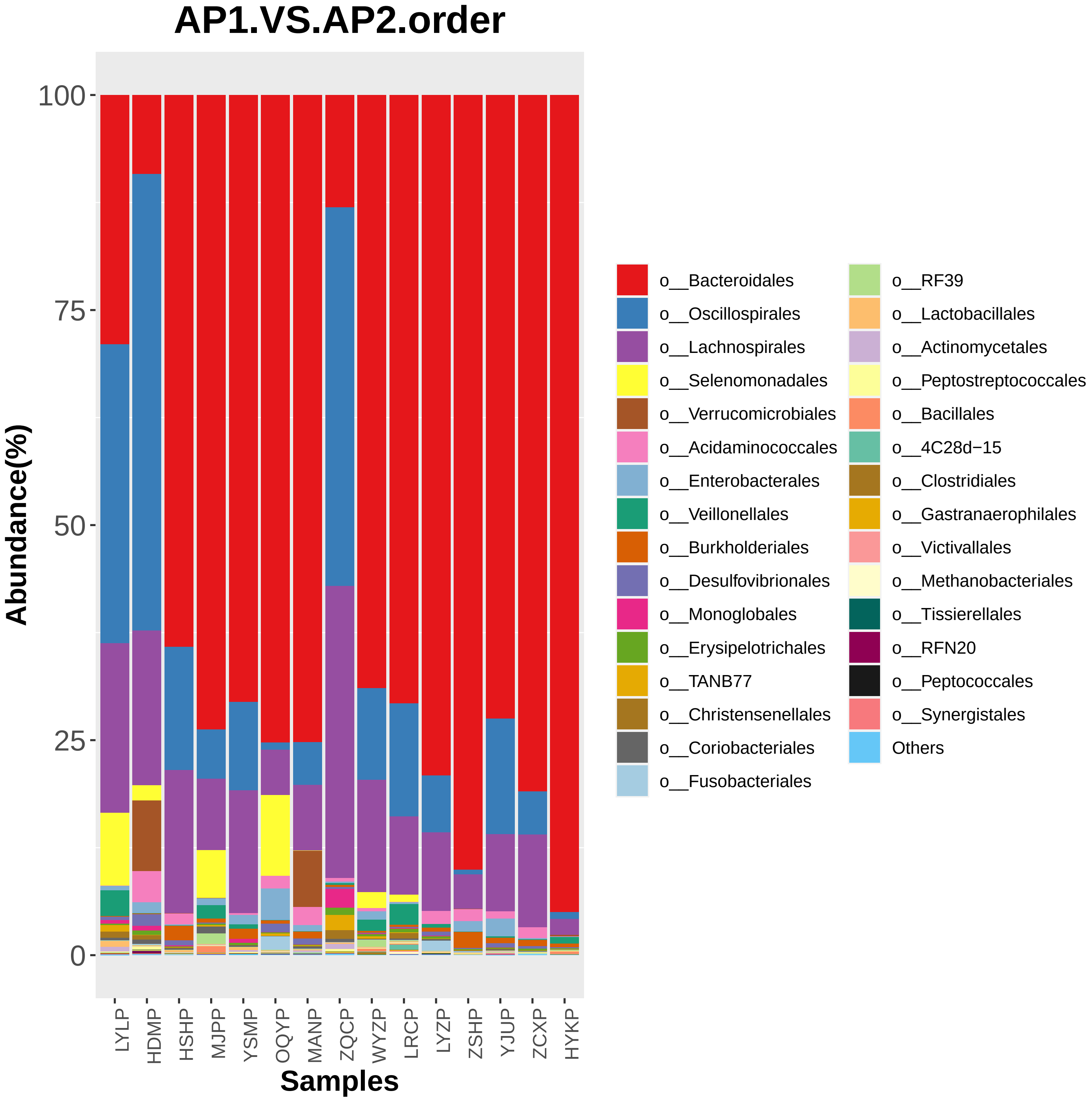

AP1.VS.AP2.order
Abundance(%)
100
75
50
25
0
LYLP
HDMP
HSHP
MJPP
YSMP
OQYP
MANP
ZQCP
WYZP
LRCP
LYZP
ZSHP
YJUP
ZCXP
HYKP
Samples
o__Bacteroidales
o__Oscillospirales
o__Lachnospirales
o__Selenomonadales
o__Verrucomicrobiales
o__Acidaminococcales
o__Enterobacterales
o__Veillonellales
o__Burkholderiales
o__Desulfovibrionales
o__Monoglobales
o__Erysipelotrichales
o__TANB77
o__Christensenellales
o__Coriobacteriales
o__Fusobacteriales
o__RF39
o__Lactobacillales
o__Actinomycetales
o__Peptostreptococcales
o__Bacillales
o__4C28d−15
o__Clostridiales
o__Gastranaerophilales
o__Victivallales
o__Methanobacteriales
o__Tissierellales
o__RFN20
o__Peptococcales
o__Synergistales
Others

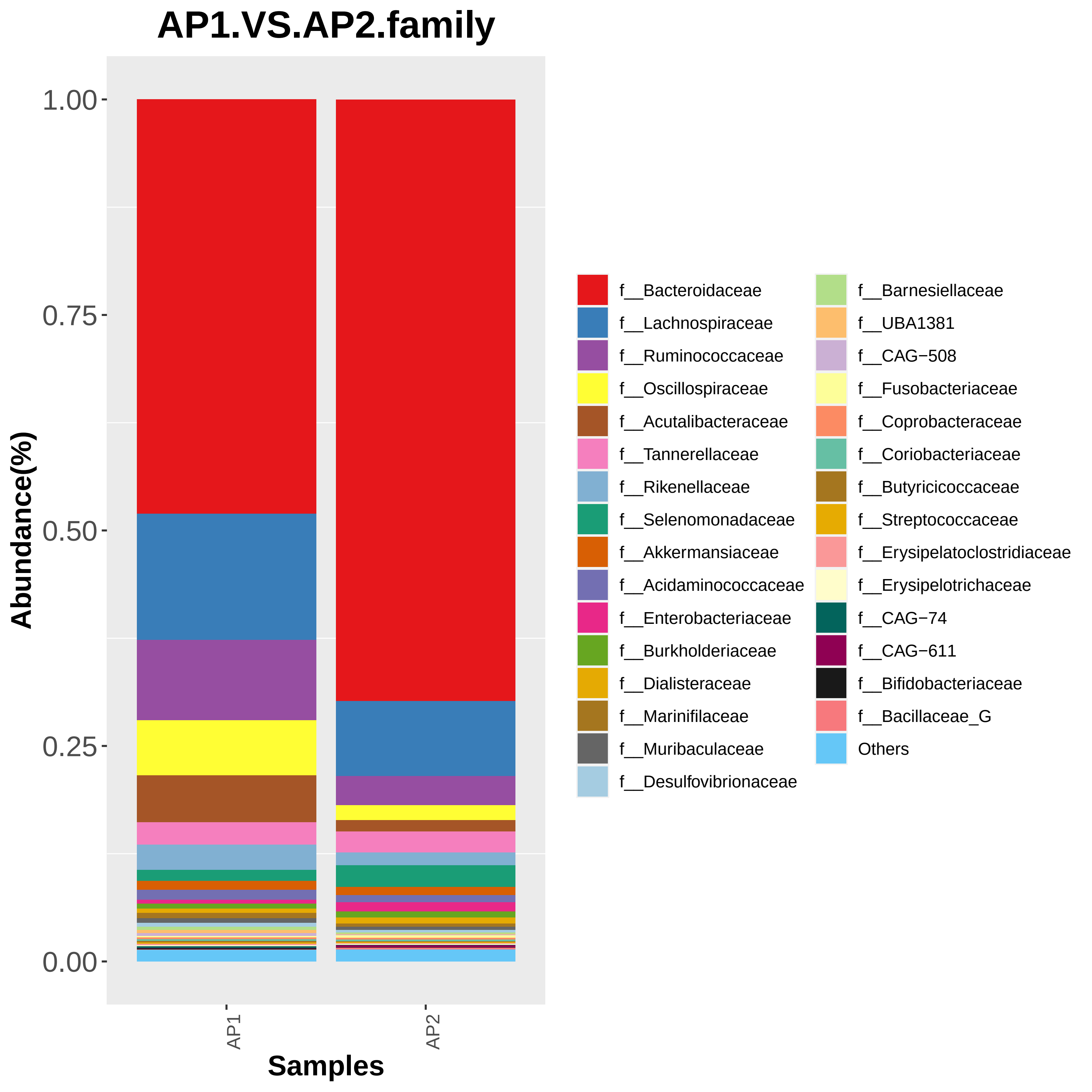
AP1.VS.AP2.family
Abundance(%)
1.00
0.75
0.50
0.25
0.00
AP1
AP2
Samples
f__Bacteroidaceae
f__Lachnospiraceae
f__Ruminococcaceae
f__Oscillospiraceae
f__Acutalibacteraceae
f__Tannerellaceae
f__Rikenellaceae
f__Selenomonadaceae
f__Akkermansiaceae
f__Acidaminococcaceae
f__Enterobacteriaceae
f__Burkholderiaceae
f__Dialisteraceae
f__Marinifilaceae
f__Muribaculaceae
f__Desulfovibrionaceae
f__Barnesiellaceae
f__UBA1381
f__CAG−508
f__Fusobacteriaceae
f__Coprobacteraceae
f__Coriobacteriaceae
f__Butyricicoccaceae
f__Streptococcaceae
f__Erysipelatoclostridiaceae
f__Erysipelotrichaceae
f__CAG−74
f__CAG−611
f__Bifidobacteriaceae
f__Bacillaceae_G
Others

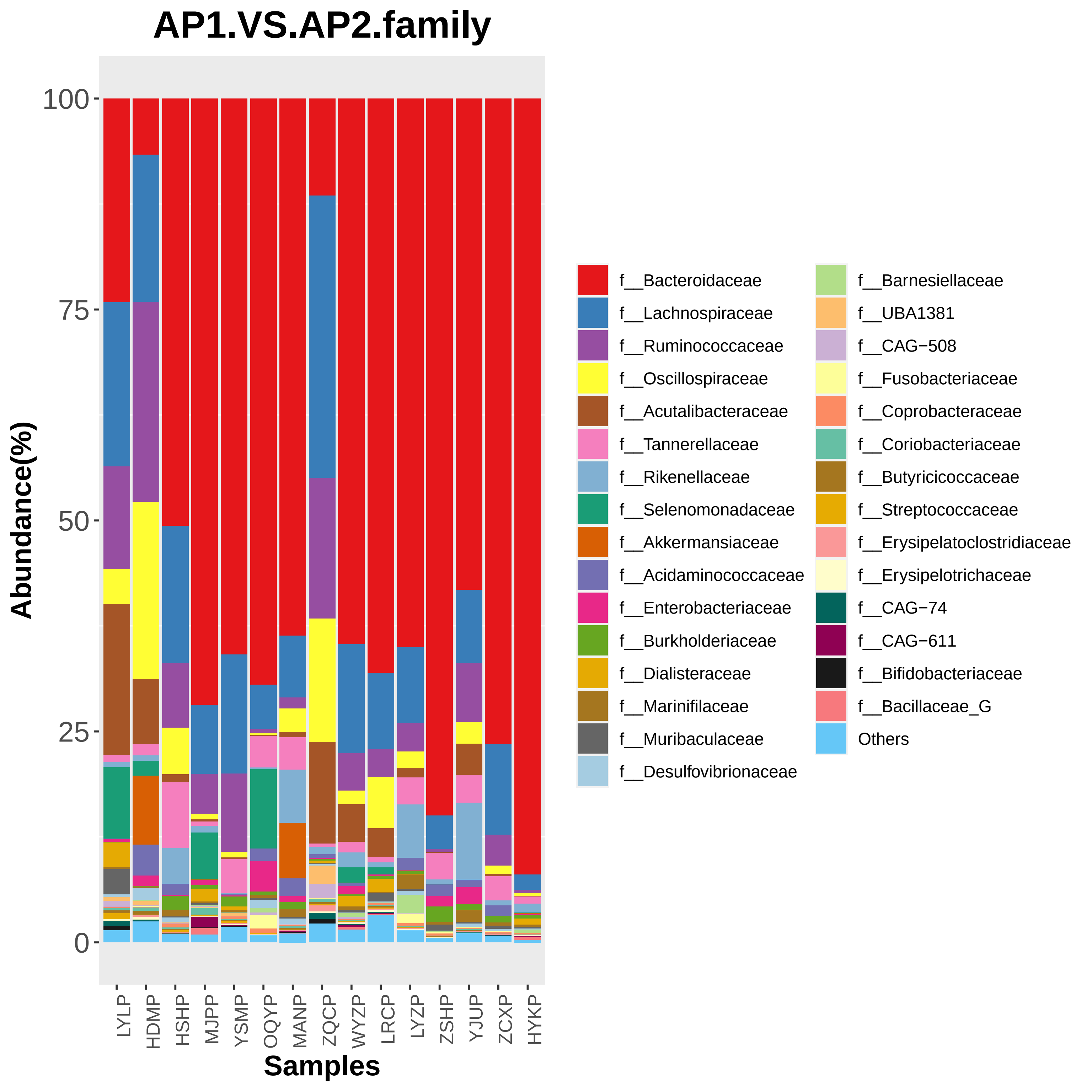
AP1.VS.AP2.family
Abundance(%)
100
75
50
25
0
LYLP
HDMP
HSHP
MJPP
YSMP
OQYP
MANP
ZQCP
WYZP
LRCP
LYZP
ZSHP
YJUP
ZCXP
HYKP
Samples
f__Bacteroidaceae
f__Lachnospiraceae
f__Ruminococcaceae
f__Oscillospiraceae
f__Acutalibacteraceae
f__Tannerellaceae
f__Rikenellaceae
f__Selenomonadaceae
f__Akkermansiaceae
f__Acidaminococcaceae
f__Enterobacteriaceae
f__Burkholderiaceae
f__Dialisteraceae
f__Marinifilaceae
f__Muribaculaceae
f__Desulfovibrionaceae
f__Barnesiellaceae
f__UBA1381
f__CAG−508
f__Fusobacteriaceae
f__Coprobacteraceae
f__Coriobacteriaceae
f__Butyricicoccaceae
f__Streptococcaceae
f__Erysipelatoclostridiaceae
f__Erysipelotrichaceae
f__CAG−74
f__CAG−611
f__Bifidobacteriaceae
f__Bacillaceae_G
Others

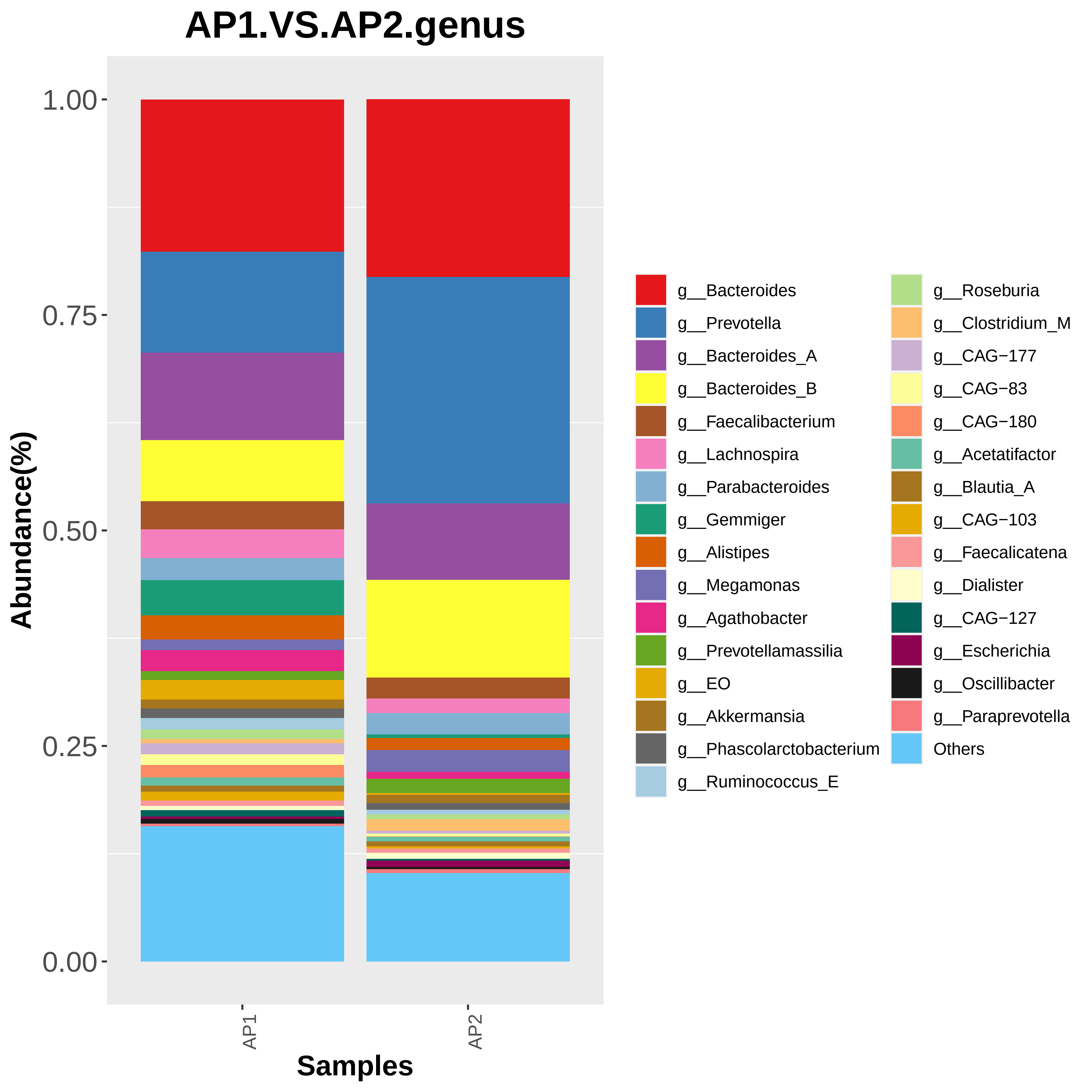

AP1.VS.AP2.genus
Abundance(%)
1.00
0.75
0.50
0.25
0.00
AP1
AP2
Samples
g__Bacteroides
g__Prevotella
g__Bacteroides_A
g__Bacteroides_B
g__Faecalibacterium
g__Lachnospira
g__Parabacteroides
g__Gemmiger
g__Alistipes
g__Megamonas
g__Agathobacter
g__Prevotellamassilia
g__EO
g__Akkermansia
g__Phascolarctobacterium
g__Ruminococcus_E
g__Roseburia
g__Clostridium_M
g__CAG−177
g__CAG−83
g__CAG−180
g__Acetatifactor
g__Blautia_A
g__CAG−103
g__Faecalicatena
g__Dialister
g__CAG−127
g__Escherichia
g__Oscillibacter
g__Paraprevotella
Others

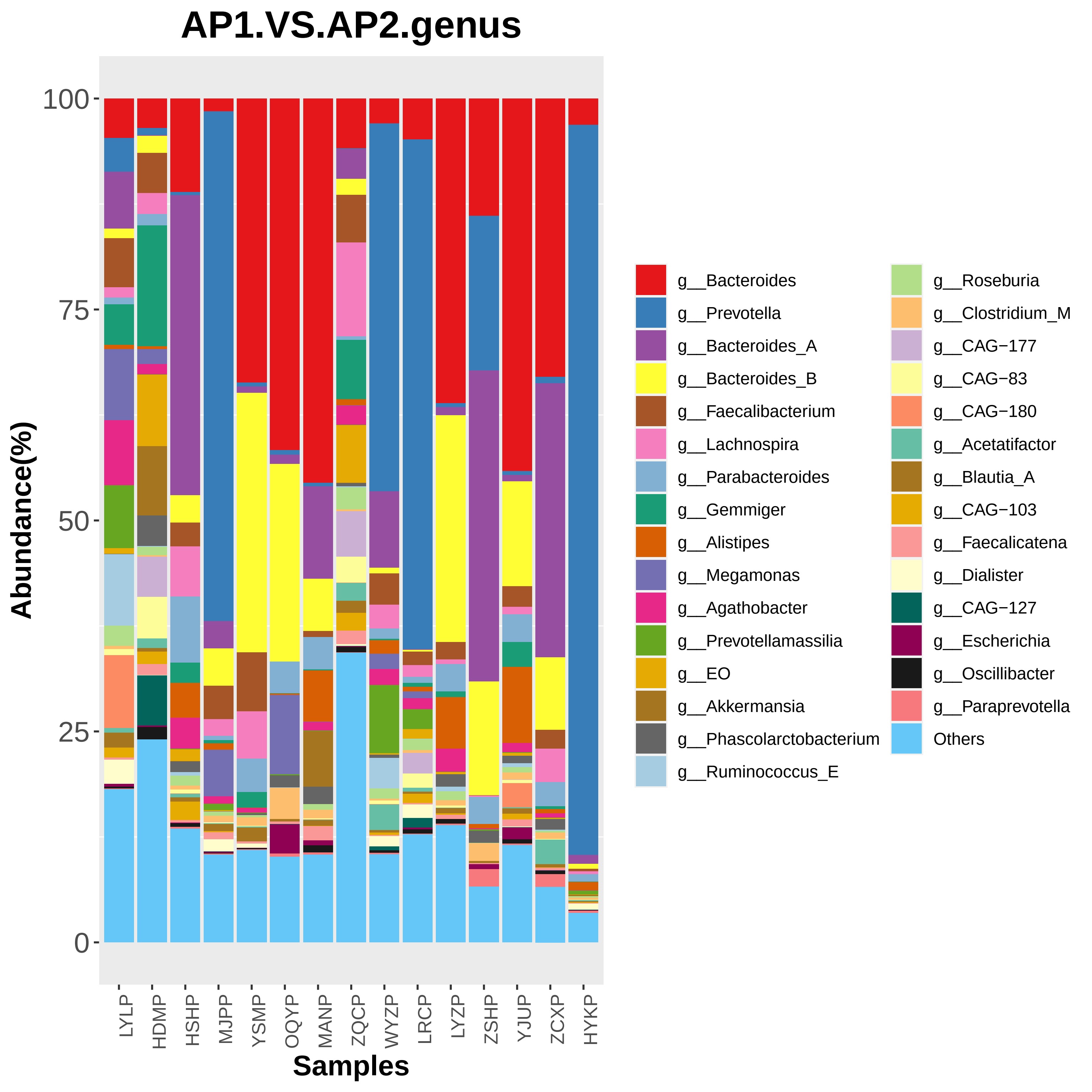
AP1.VS.AP2.genus
Abundance(%)
100
75
50
25
0
LYLP
HDMP
HSHP
MJPP
YSMP
OQYP
MANP
ZQCP
WYZP
LRCP
LYZP
ZSHP
YJUP
ZCXP
HYKP
Samples
g__Bacteroides
g__Prevotella
g__Bacteroides_A
g__Bacteroides_B
g__Faecalibacterium
g__Lachnospira
g__Parabacteroides
g__Gemmiger
g__Alistipes
g__Megamonas
g__Agathobacter
g__Prevotellamassilia
g__EO
g__Akkermansia
g__Phascolarctobacterium
g__Ruminococcus_E
g__Roseburia
g__Clostridium_M
g__CAG−177
g__CAG−83
g__CAG−180
g__Acetatifactor
g__Blautia_A
g__CAG−103
g__Faecalicatena
g__Dialister
g__CAG−127
g__Escherichia
g__Oscillibacter
g__Paraprevotella
Others

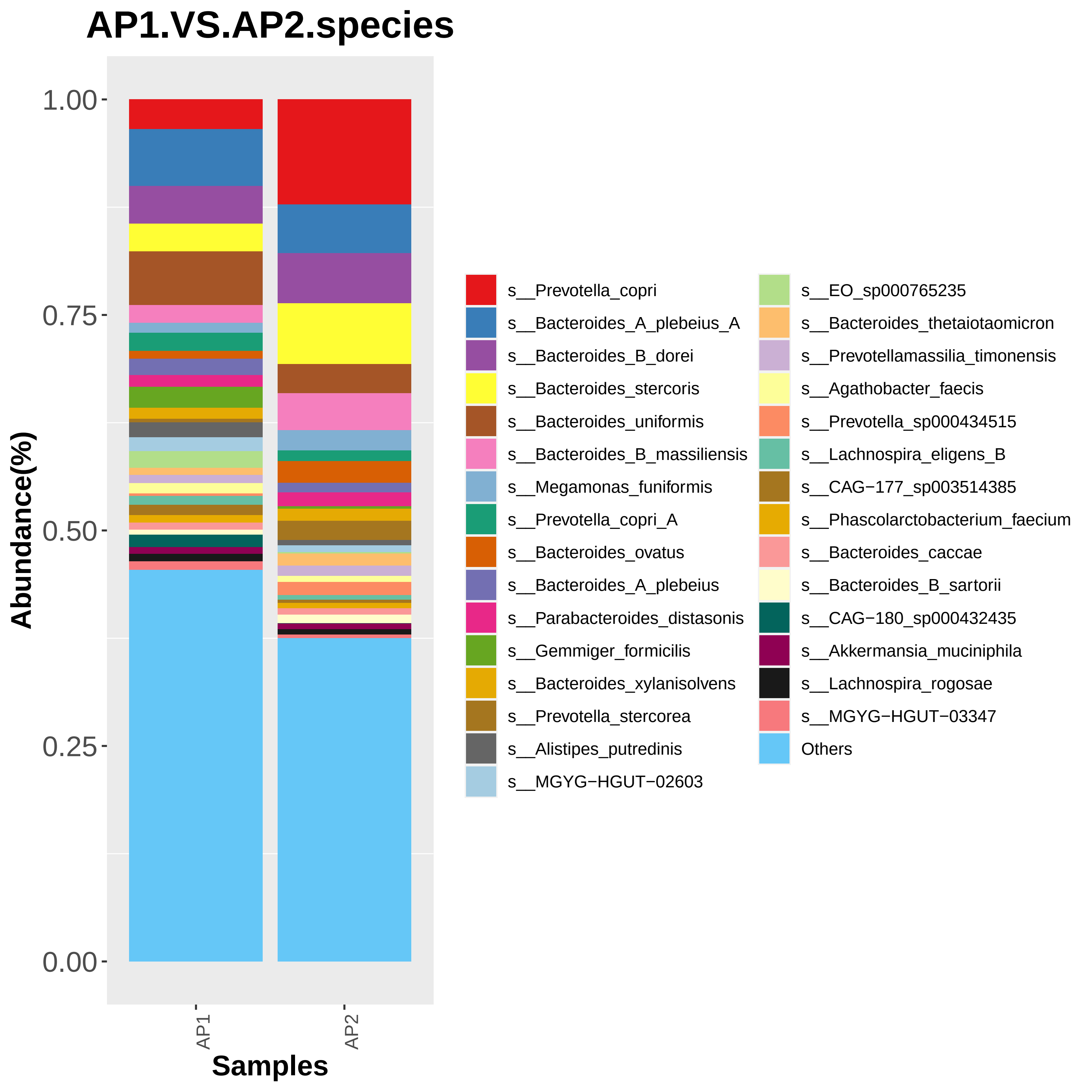

AP1.VS.AP2.species
Abundance(%)
1.00
0.75
0.50
0.25
0.00
AP1
AP2
Samples
s__Prevotella_copri
s__Bacteroides_A_plebeius_A
s__Bacteroides_B_dorei
s__Bacteroides_stercoris
s__Bacteroides_uniformis
s__Bacteroides_B_massiliensis
s__Megamonas_funiformis
s__Prevotella_copri_A
s__Bacteroides_ovatus
s__Bacteroides_A_plebeius
s__Parabacteroides_distasonis
s__Gemmiger_formicilis
s__Bacteroides_xylanisolvens
s__Prevotella_stercorea
s__Alistipes_putredinis
s__MGYG−HGUT−02603
s__EO_sp000765235
s__Bacteroides_thetaiotaomicron
s__Prevotellamassilia_timonensis
s__Agathobacter_faecis
s__Prevotella_sp000434515
s__Lachnospira_eligens_B
s__CAG−177_sp003514385
s__Phascolarctobacterium_faecium
s__Bacteroides_caccae
s__Bacteroides_B_sartorii
s__CAG−180_sp000432435
s__Akkermansia_muciniphila
s__Lachnospira_rogosae
s__MGYG−HGUT−03347
Others

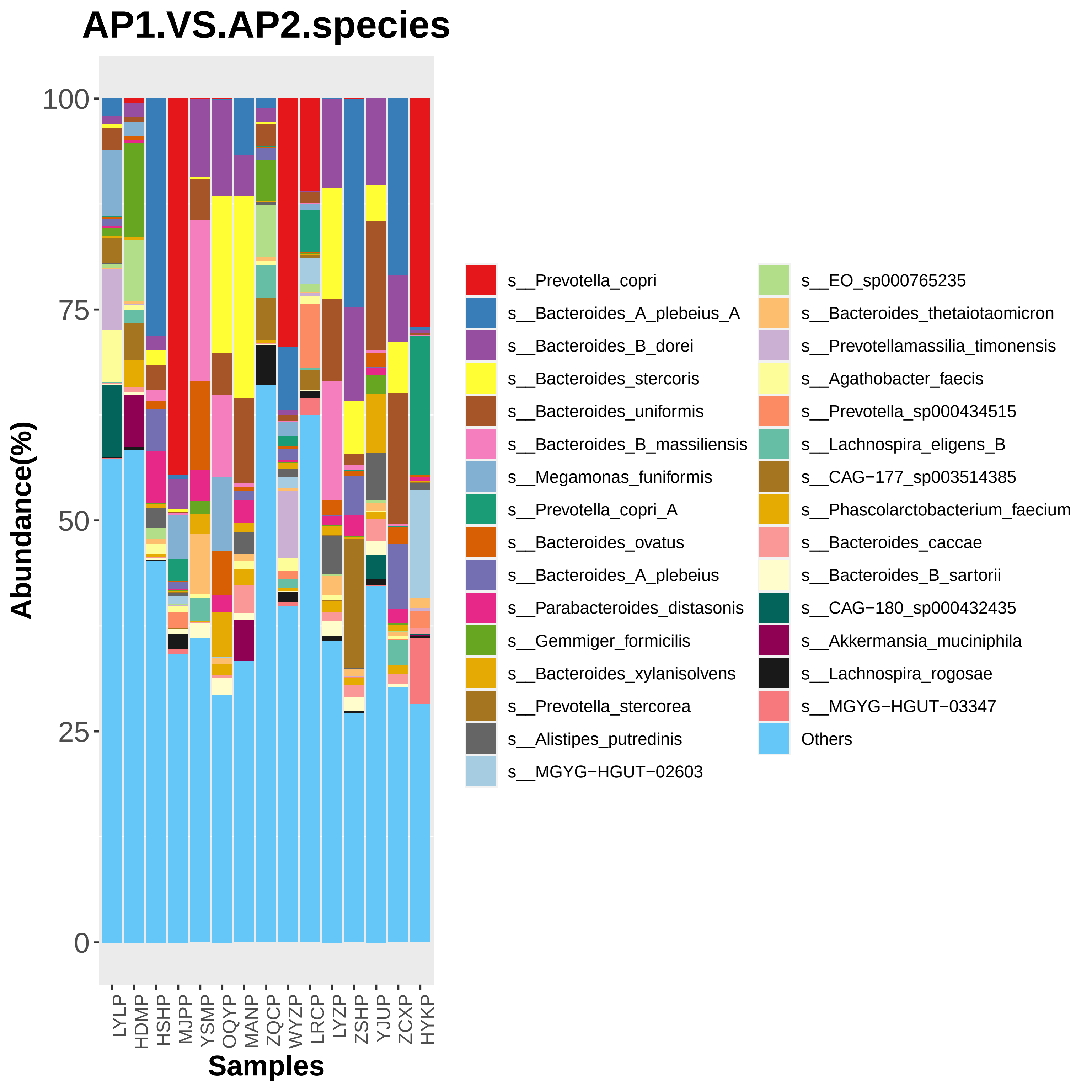
AP1.VS.AP2.species
Abundance(%)
100
75
50
25
0
LYLP
HDMP
HSHP
MJPP
YSMP
OQYP
MANP
ZQCP
WYZP
LRCP
LYZP
ZSHP
YJUP
ZCXP
HYKP
Samples
s__Prevotella_copri
s__Bacteroides_A_plebeius_A
s__Bacteroides_B_dorei
s__Bacteroides_stercoris
s__Bacteroides_uniformis
s__Bacteroides_B_massiliensis
s__Megamonas_funiformis
s__Prevotella_copri_A
s__Bacteroides_ovatus
s__Bacteroides_A_plebeius
s__Parabacteroides_distasonis
s__Gemmiger_formicilis
s__Bacteroides_xylanisolvens
s__Prevotella_stercorea
s__Alistipes_putredinis
s__MGYG−HGUT−02603
s__EO_sp000765235
s__Bacteroides_thetaiotaomicron
s__Prevotellamassilia_timonensis
s__Agathobacter_faecis
s__Prevotella_sp000434515
s__Lachnospira_eligens_B
s__CAG−177_sp003514385
s__Phascolarctobacterium_faecium
s__Bacteroides_caccae
s__Bacteroides_B_sartorii
s__CAG−180_sp000432435
s__Akkermansia_muciniphila
s__Lachnospira_rogosae
s__MGYG−HGUT−03347
Others

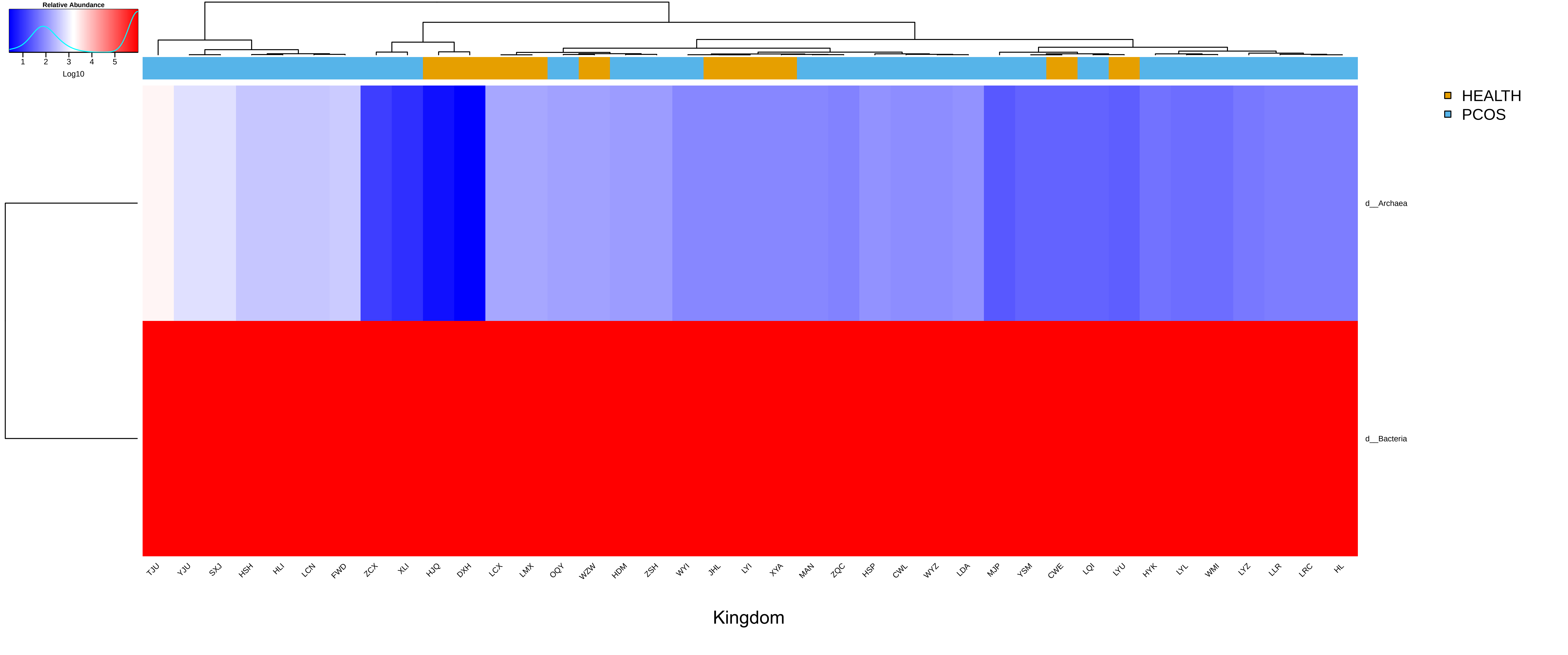
Relative Abundance
1 2 3 4 5
Log10
HEALTH
PCOS
d__Archaea
d__Bacteria
TJU YJU SXJ HSH HLI LCN FWD ZCX XLI HJQ DXH LCX LMX OQY WZW HDM ZSH WYI JHL LYI XYA MAN ZQC HSP CWL WYZ LDA MJP YSM CWE LQI LYU HYK LYL WMI LYZ LLR LRC HL
Kingdom

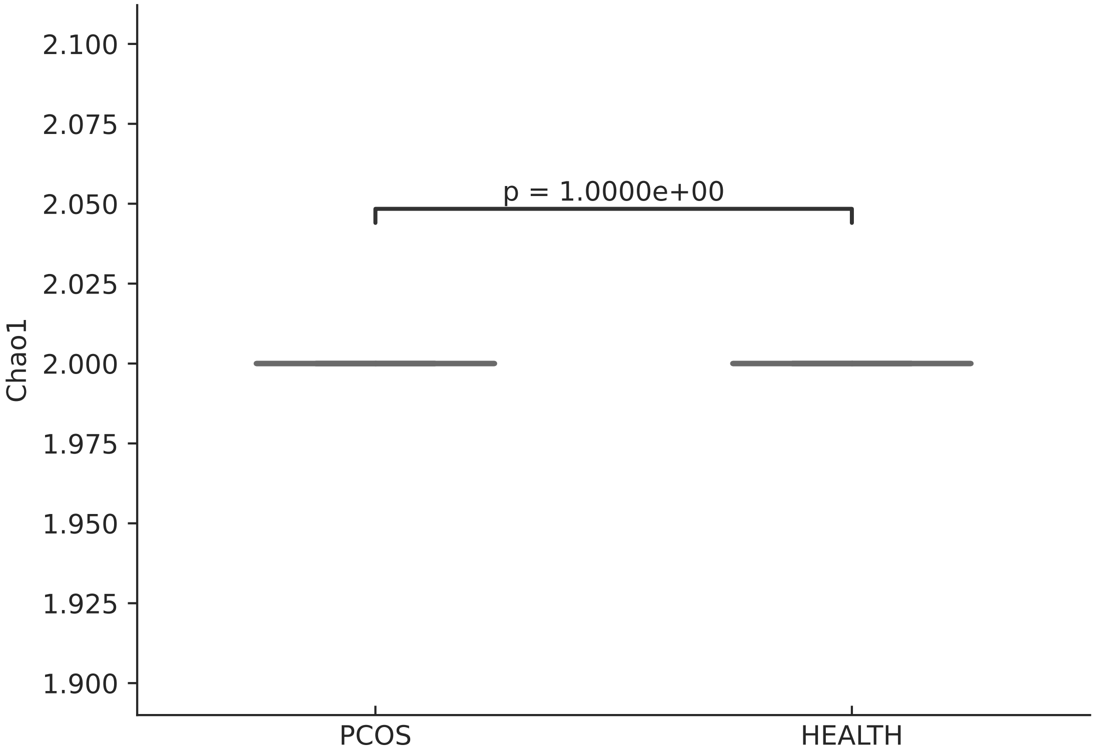

Alpha diversity of kingdom

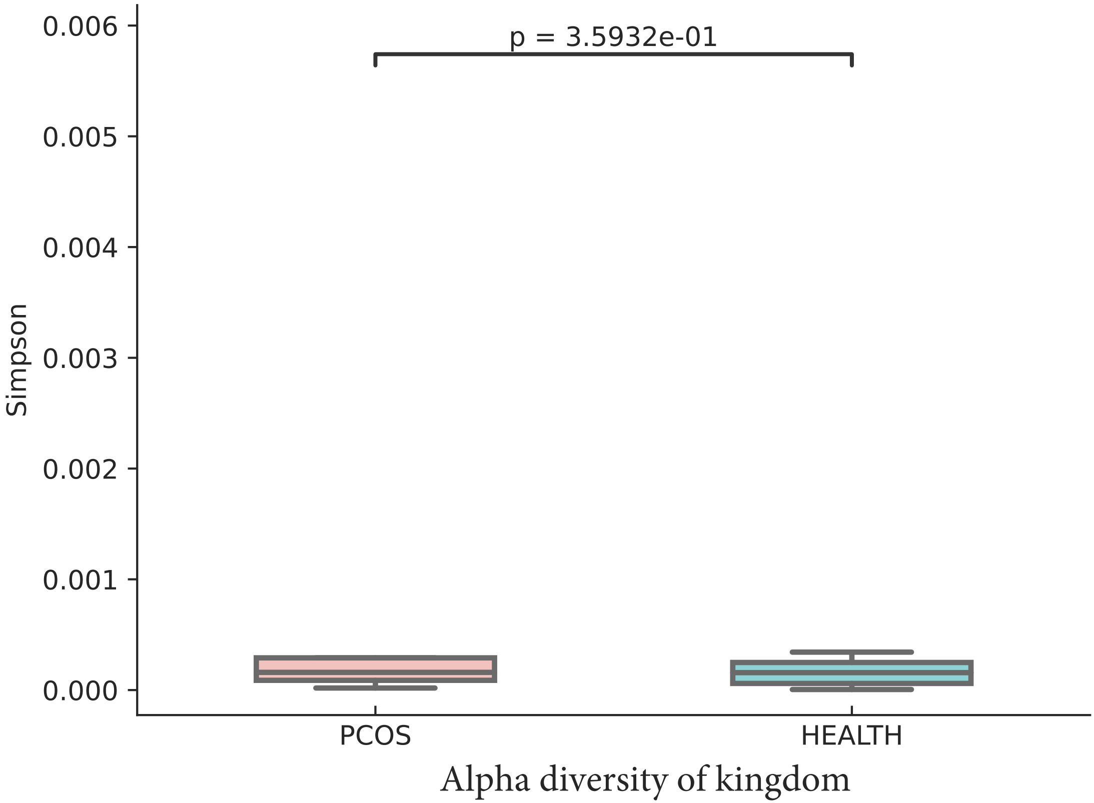
p = 3.5932e-01
0.006
0.005
0.004
0.003
0.002
0.001
0.000
Simpson
PCOS
HEALTH
Alpha diversity of kingdom

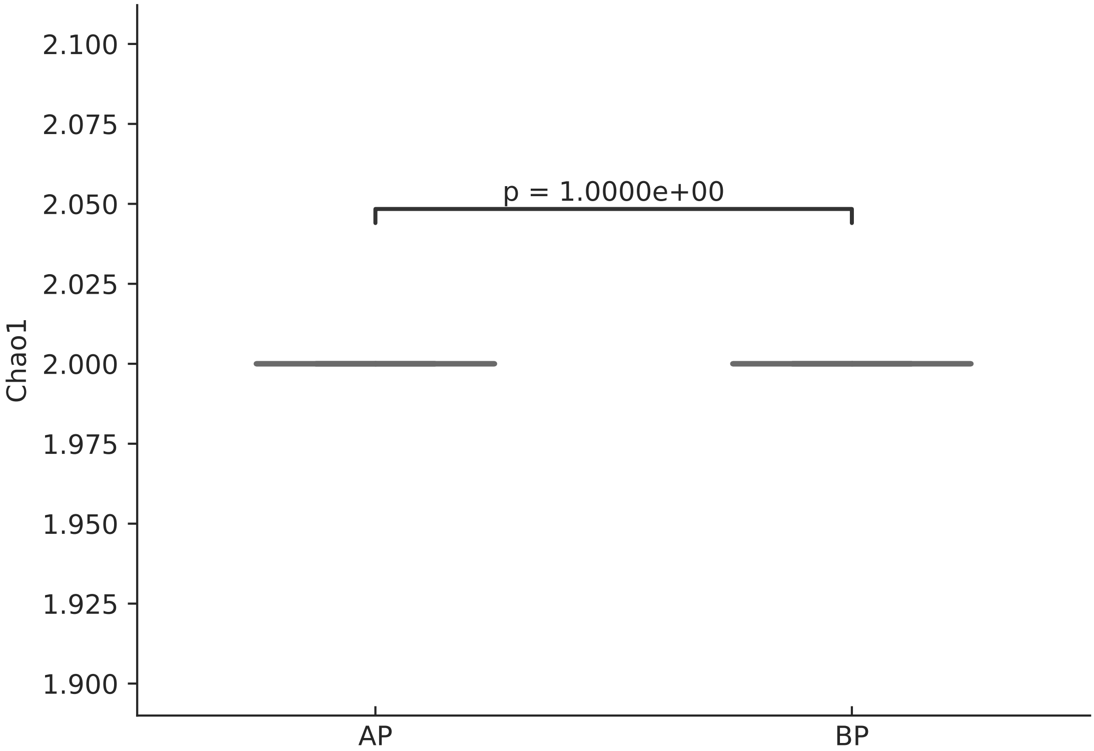

Alpha diversity of kingdom

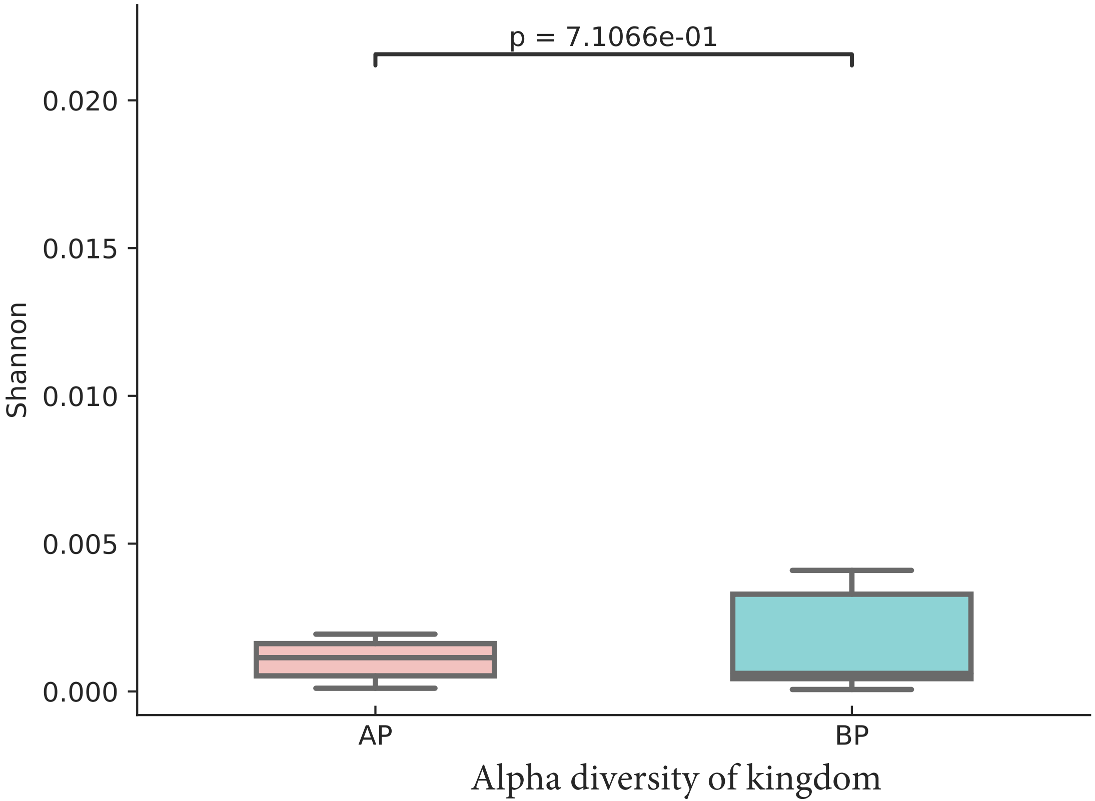

p = 7.1066e-01
0.020
0.015
0.010
0.005
0.000
Shannon
AP
BP
Alpha diversity of kingdom

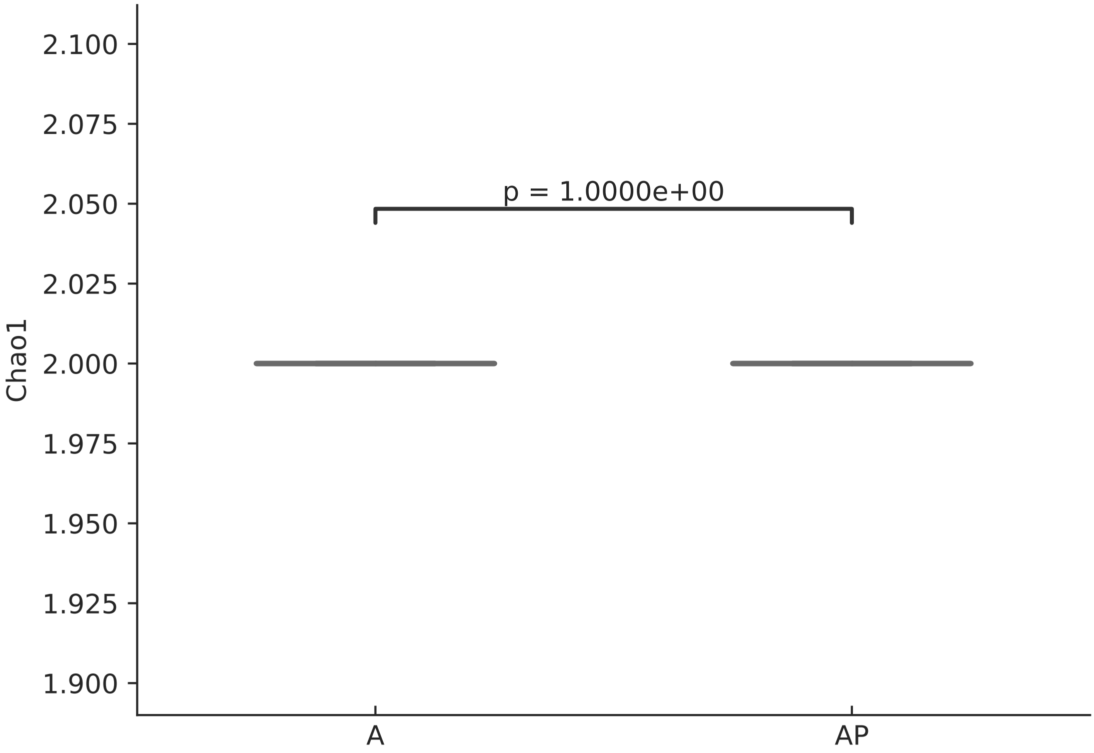

Alpha diversity of kingdom

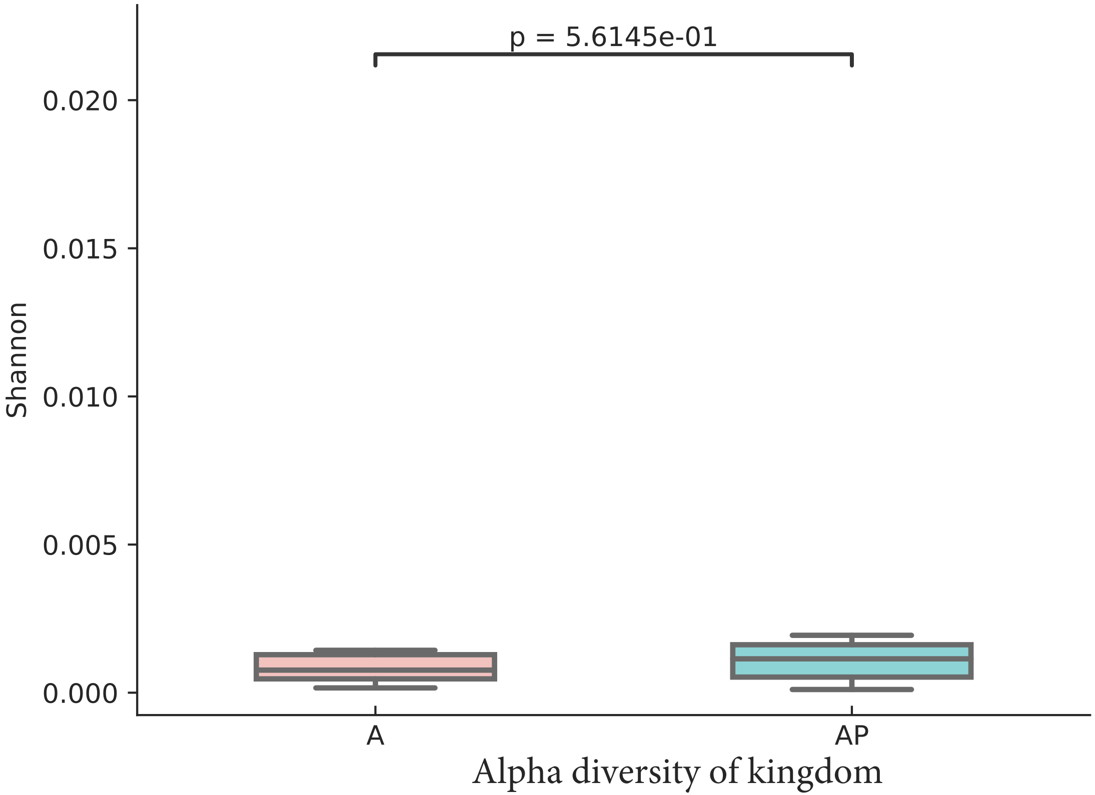
p = 5.6145e-01
0.020
0.015
0.010
0.005
0.000
Shannon
A
AP
Alpha diversity of kingdom

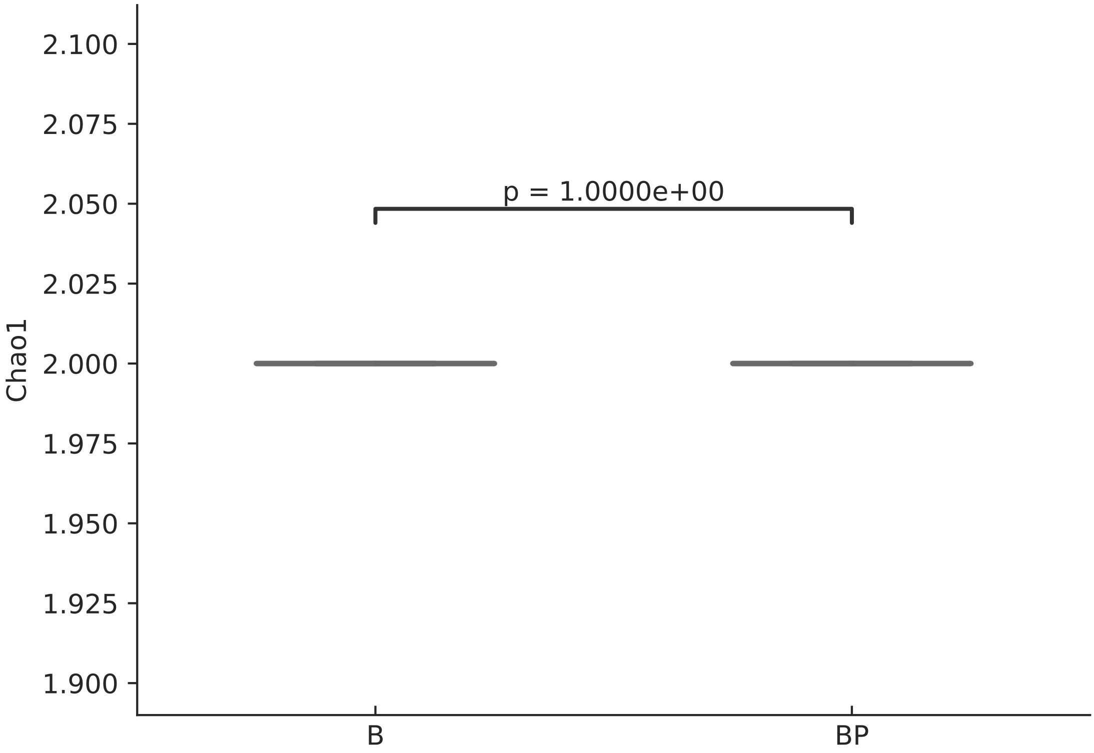

Alpha diversity of kingdom

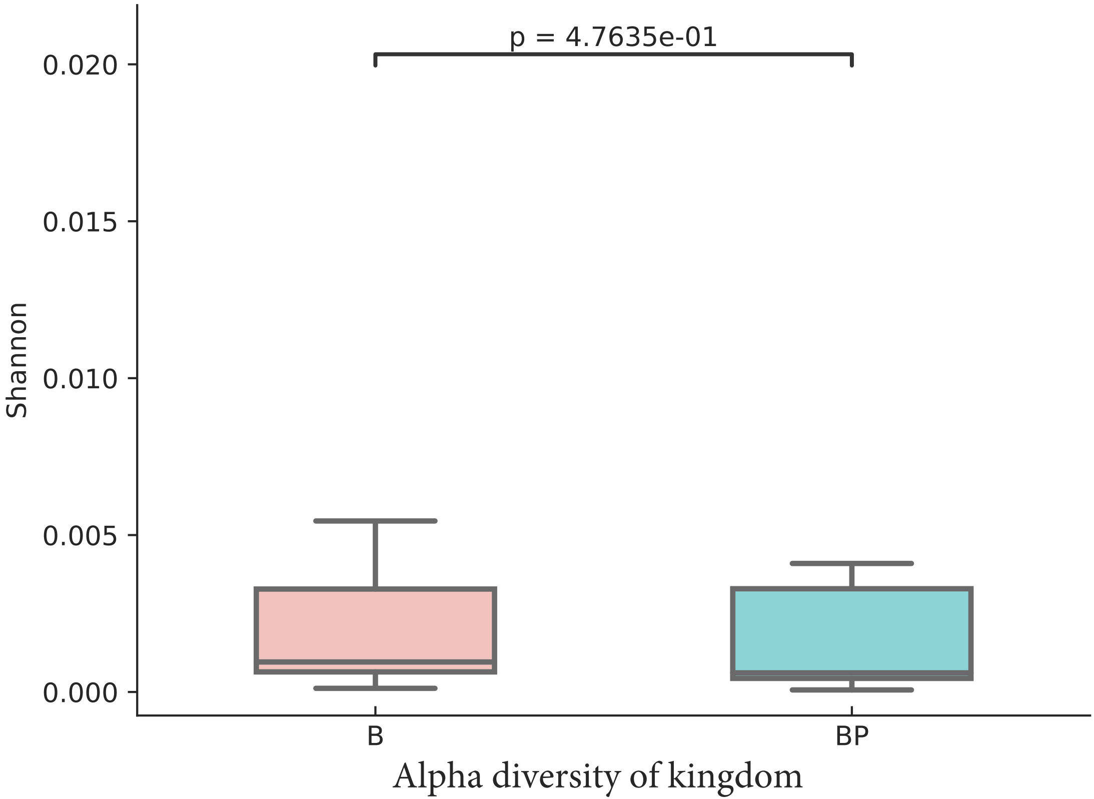
p = 4.7635e-01
0.020
0.015
0.010
0.005
0.000
Shannon
B
BP
Alpha diversity of kingdom

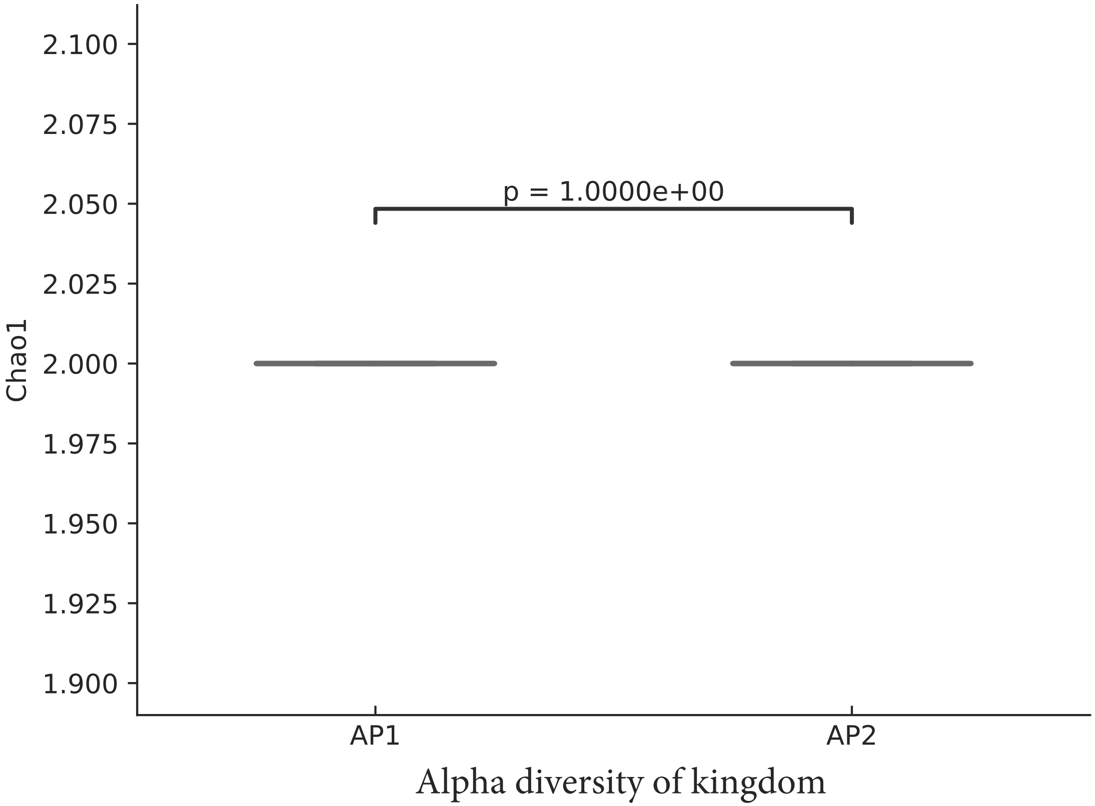
p = 1.0000e+00
Chao1
2.100
2.075
2.050
2.025
2.000
1.975
1.950
1.925
1.900
AP1
AP2
Alpha diversity of kingdom

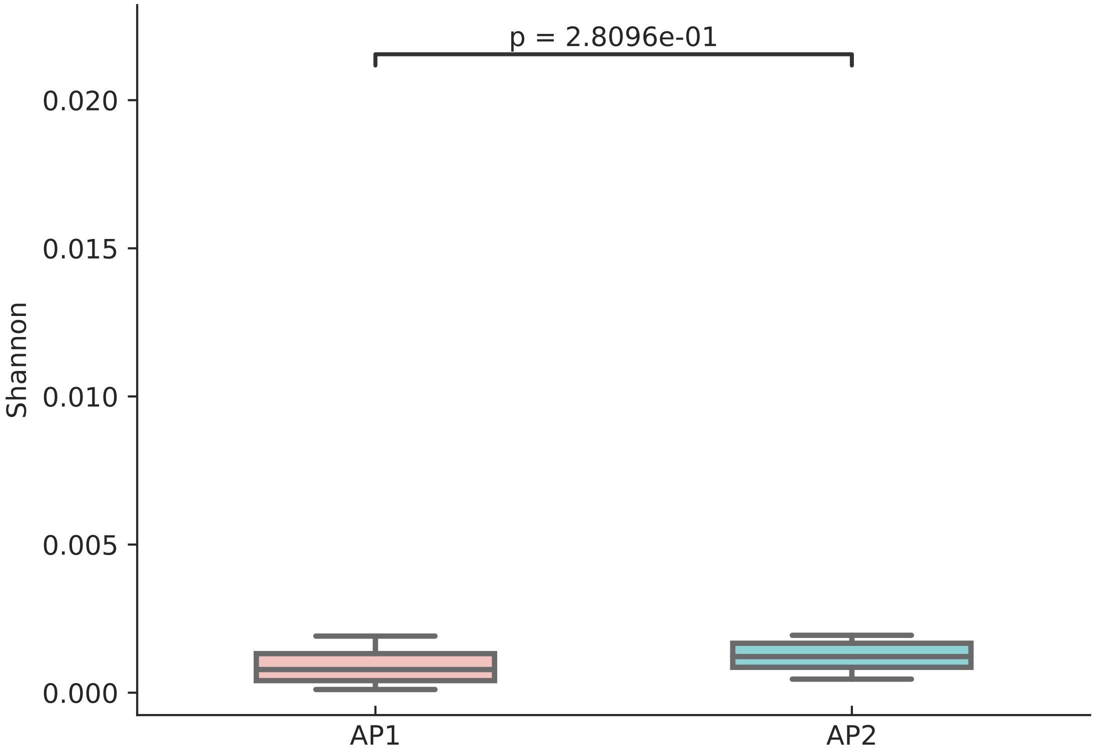

Alpha diversity of kingdom

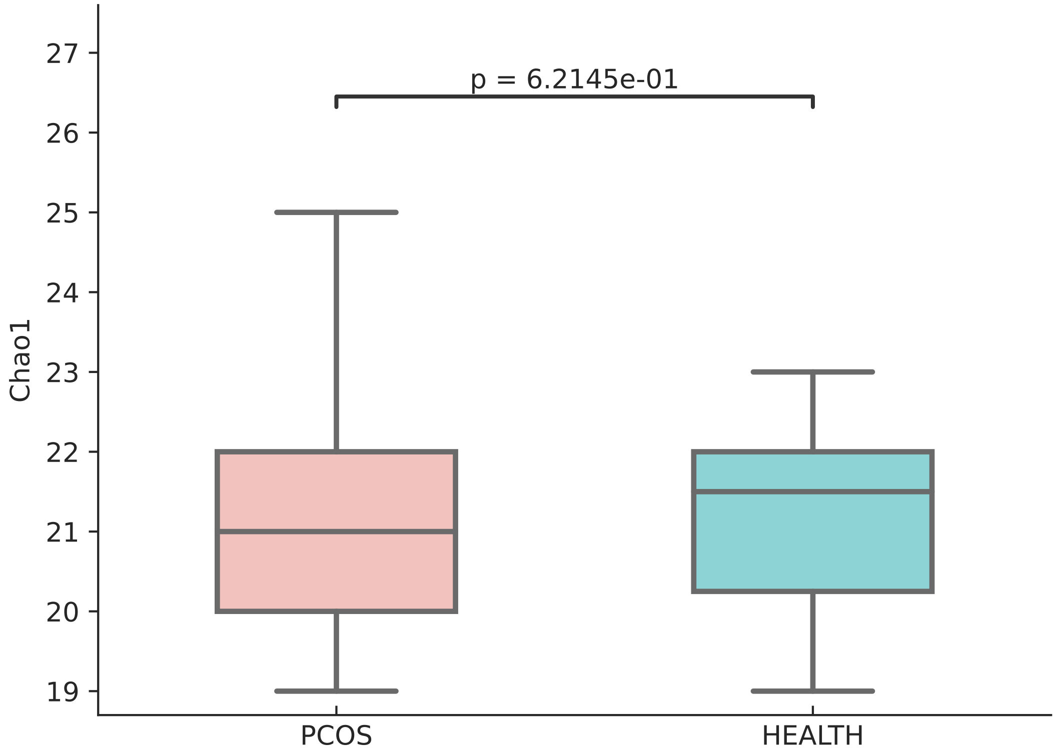

Alpha diversity of phylum

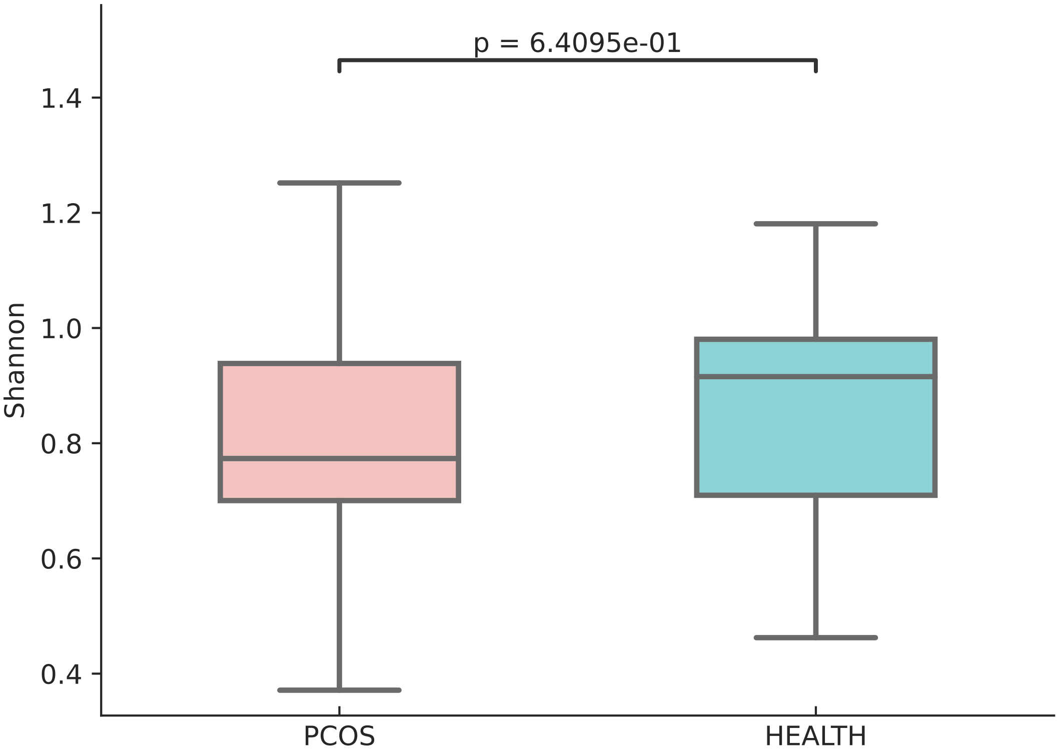

Alpha diversity of phylum

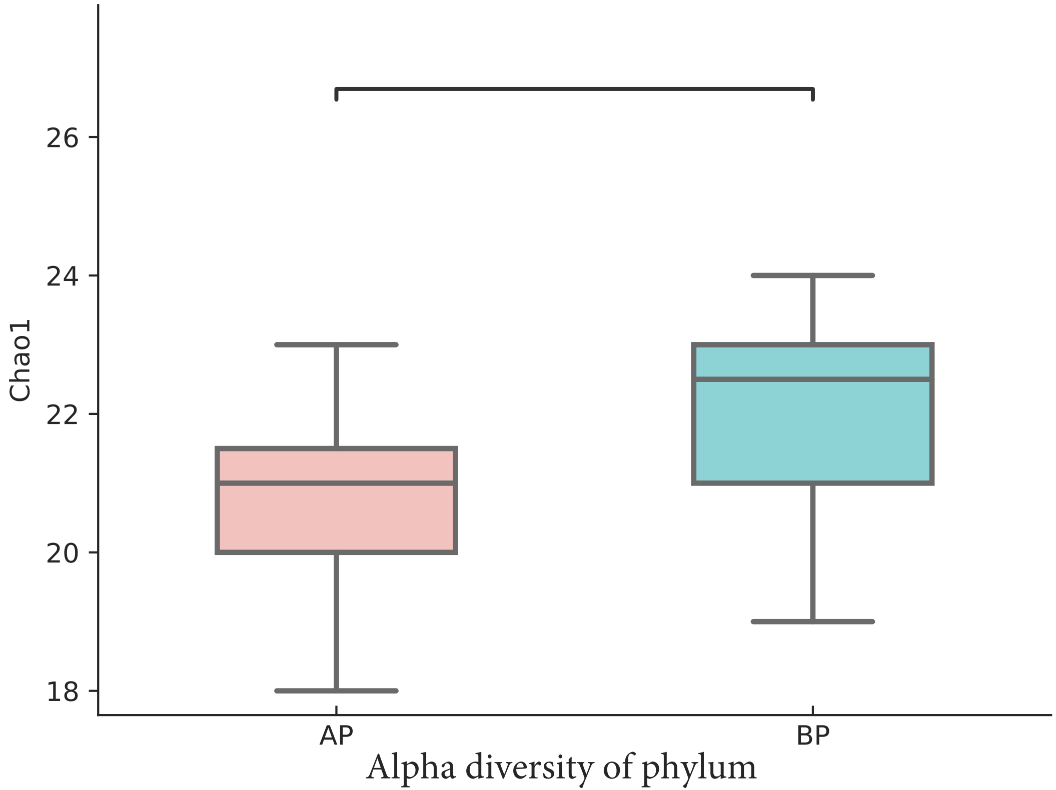

Chao1
26
24
22
20
18
AP
BP
Alpha diversity of phylum

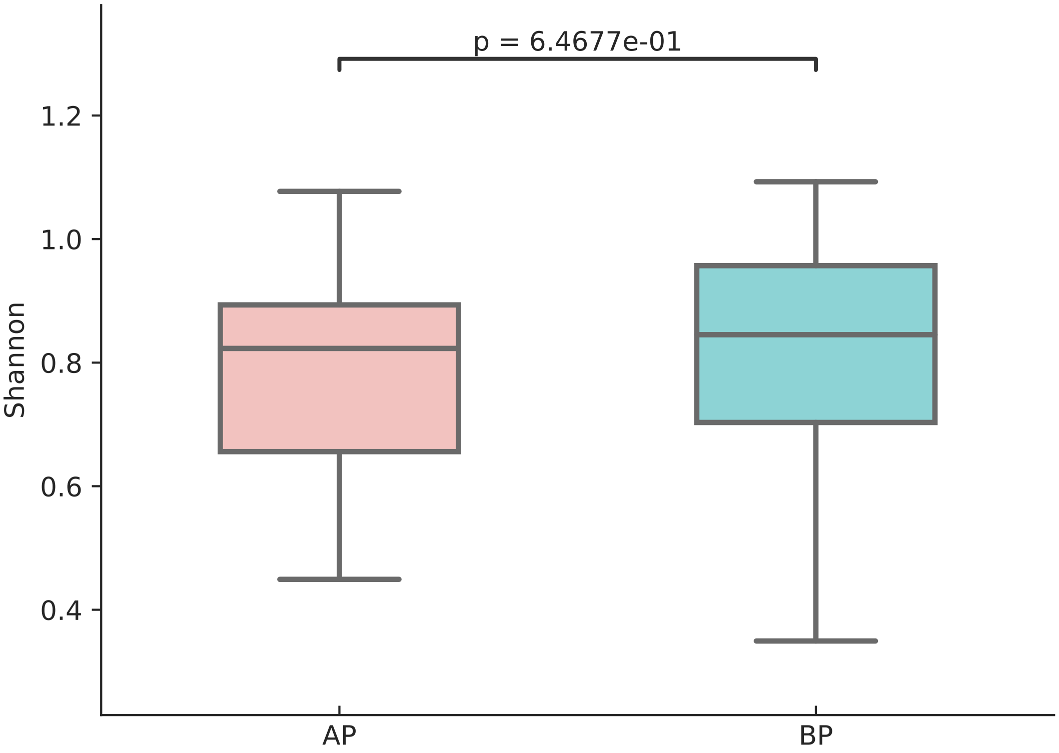

Alpha diversity of phylum

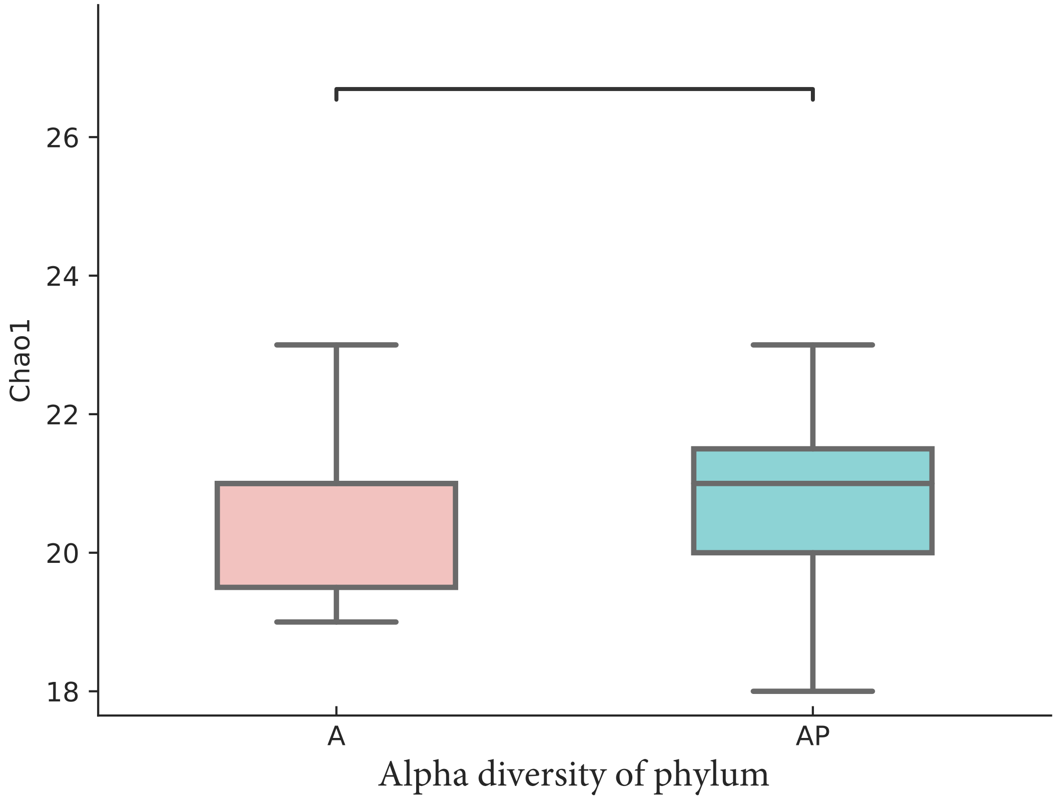

Chao1
26
24
22
20
18
A
AP
Alpha diversity of phylum

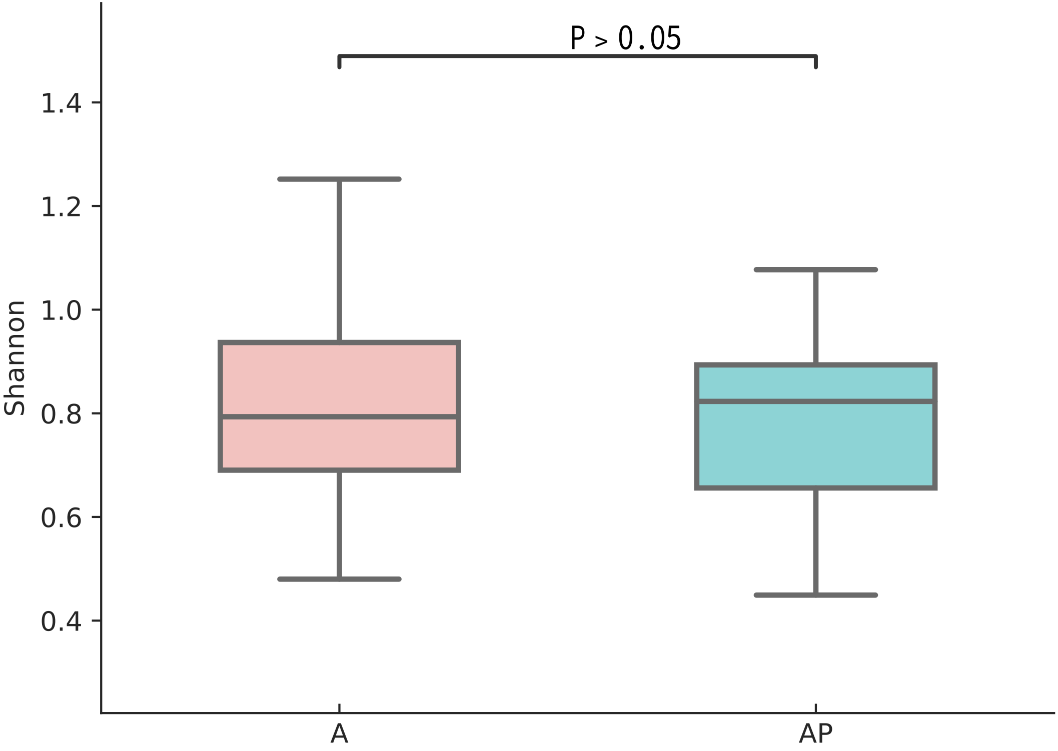

Alpha diversity of phylum

a

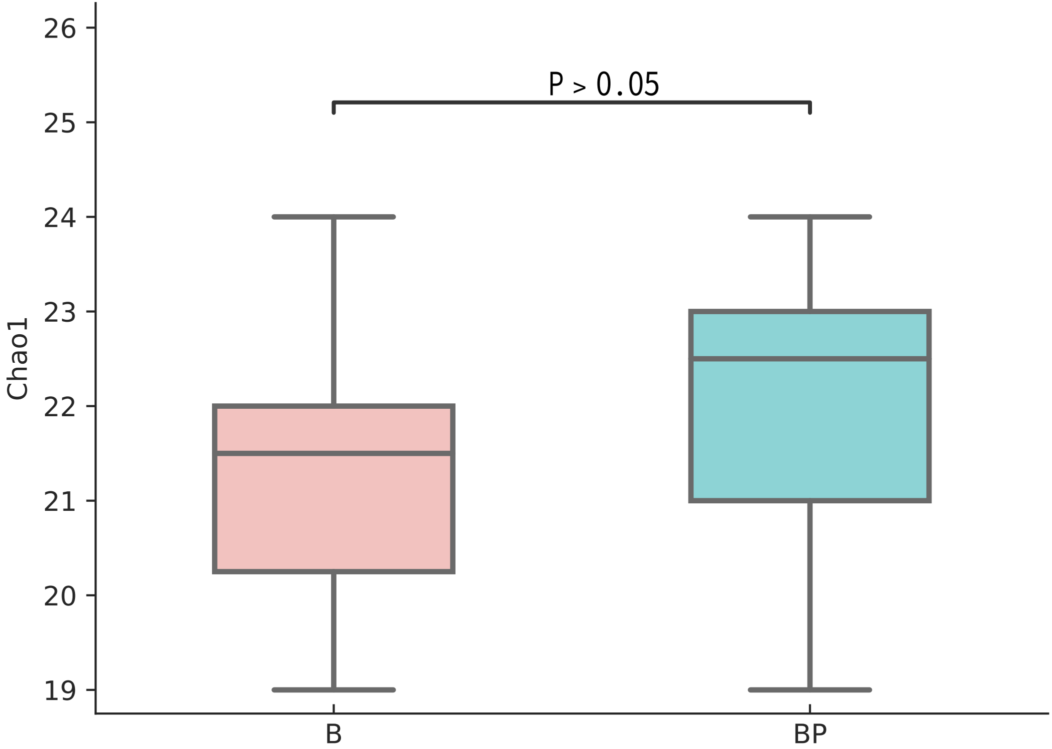

a

Alpha diversity of phylum

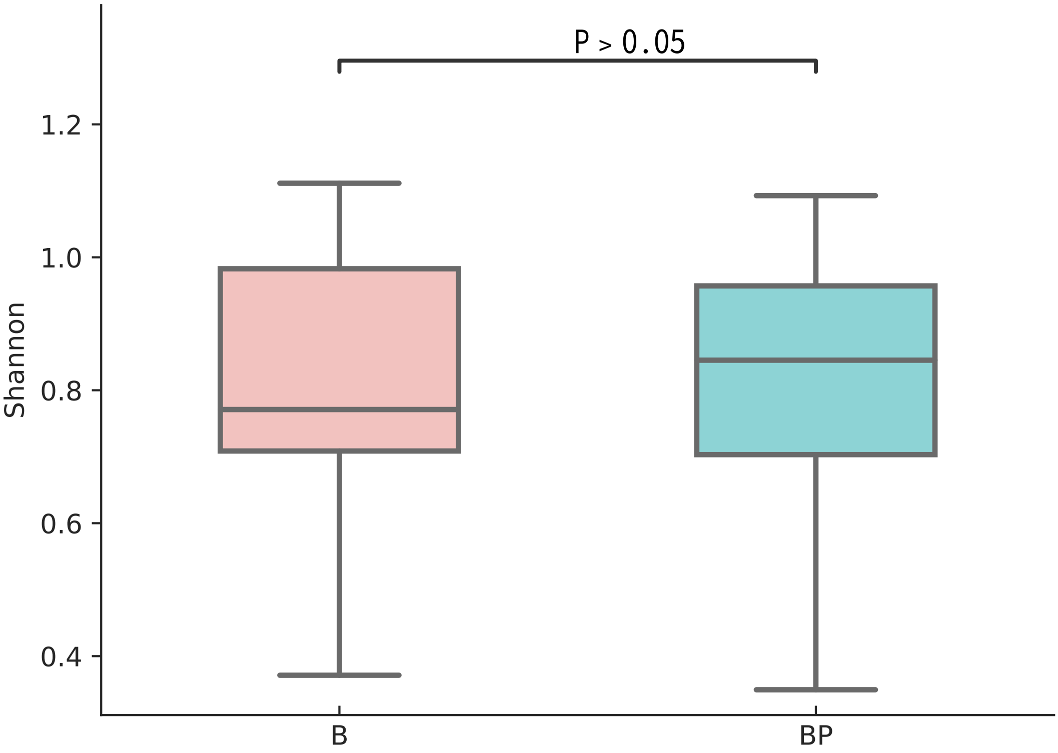

a

Alpha diversity of phylum

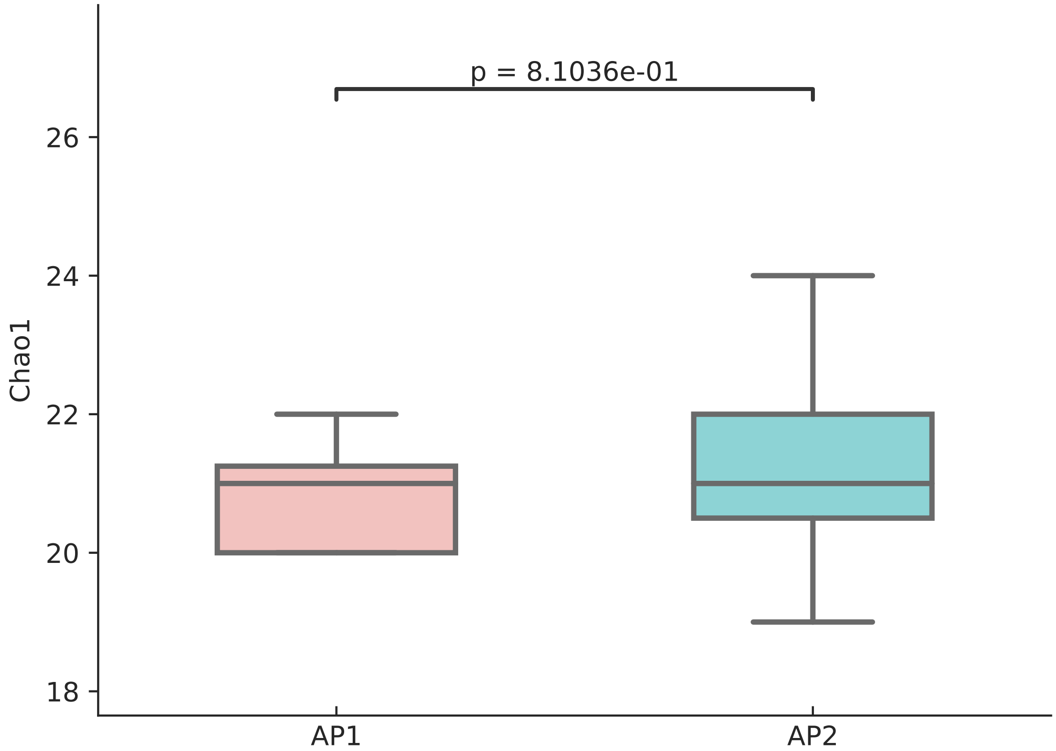

Alpha diversity of phylum

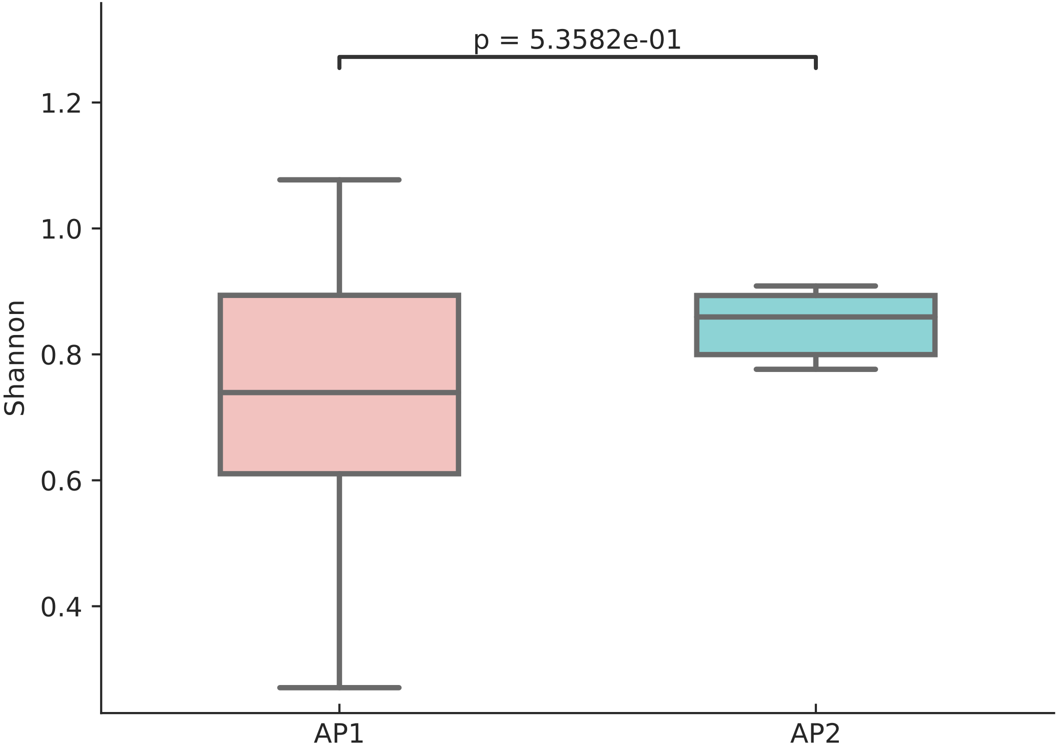

Alpha diversity of phylum

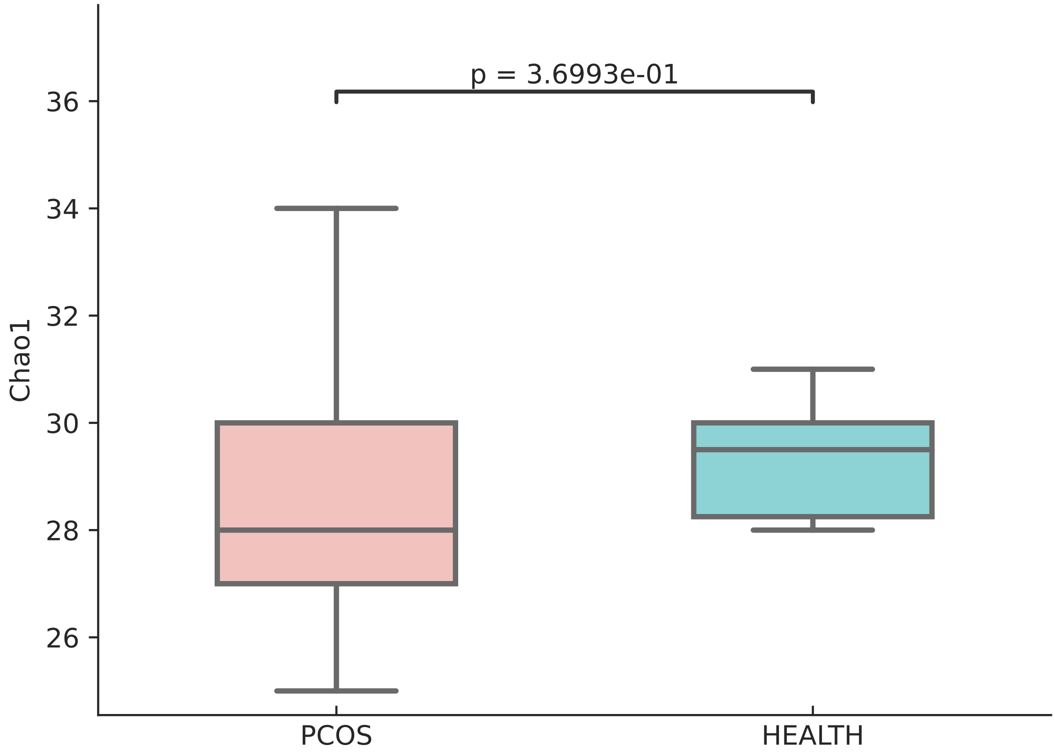

Alpha diversity of class

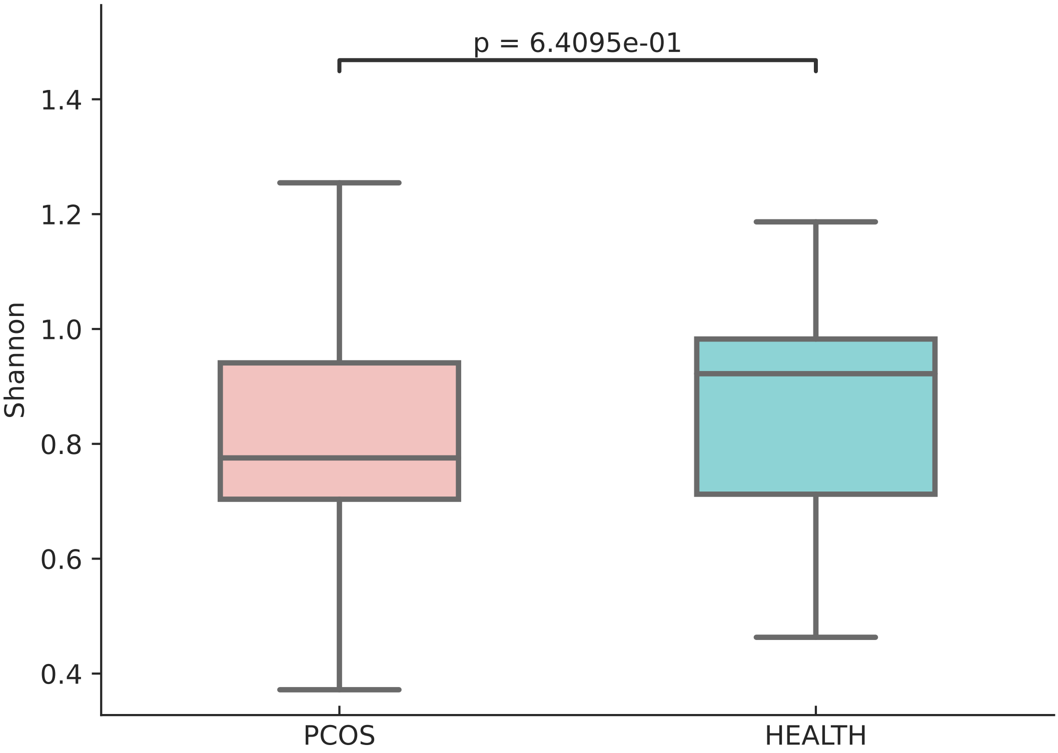

Alpha diversity of class

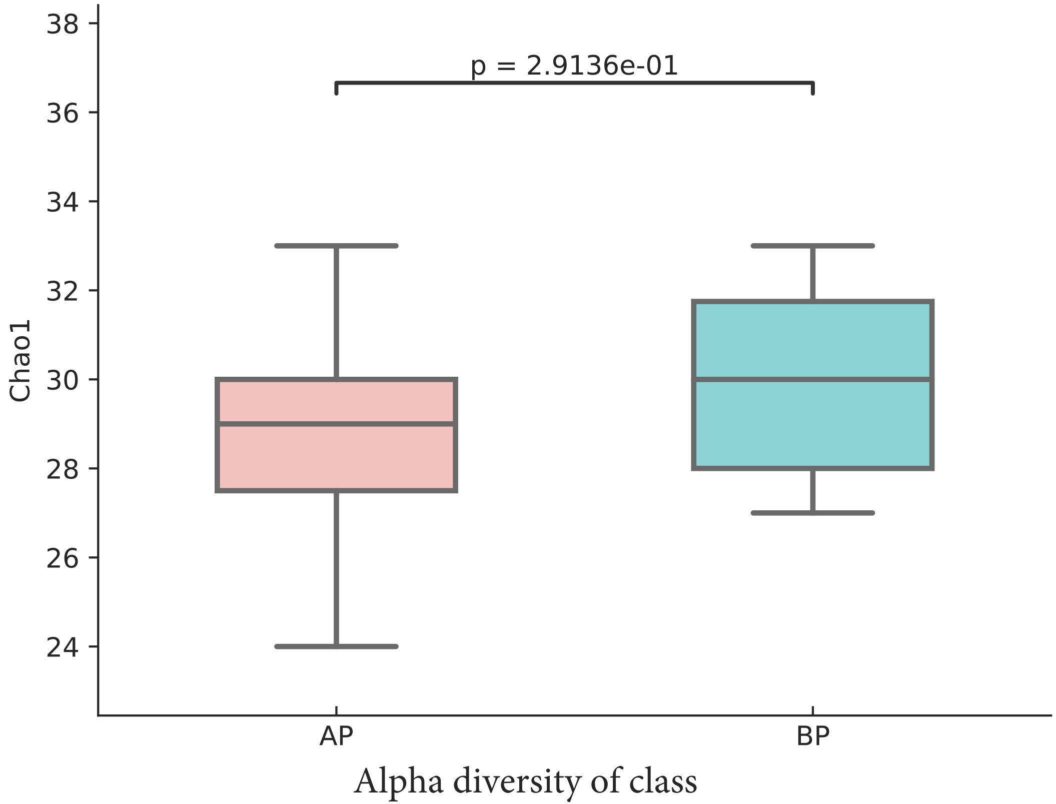
p = 2.9136e-01
38
36
34
32
30
28
26
24
Chao1
AP
BP
Alpha diversity of class

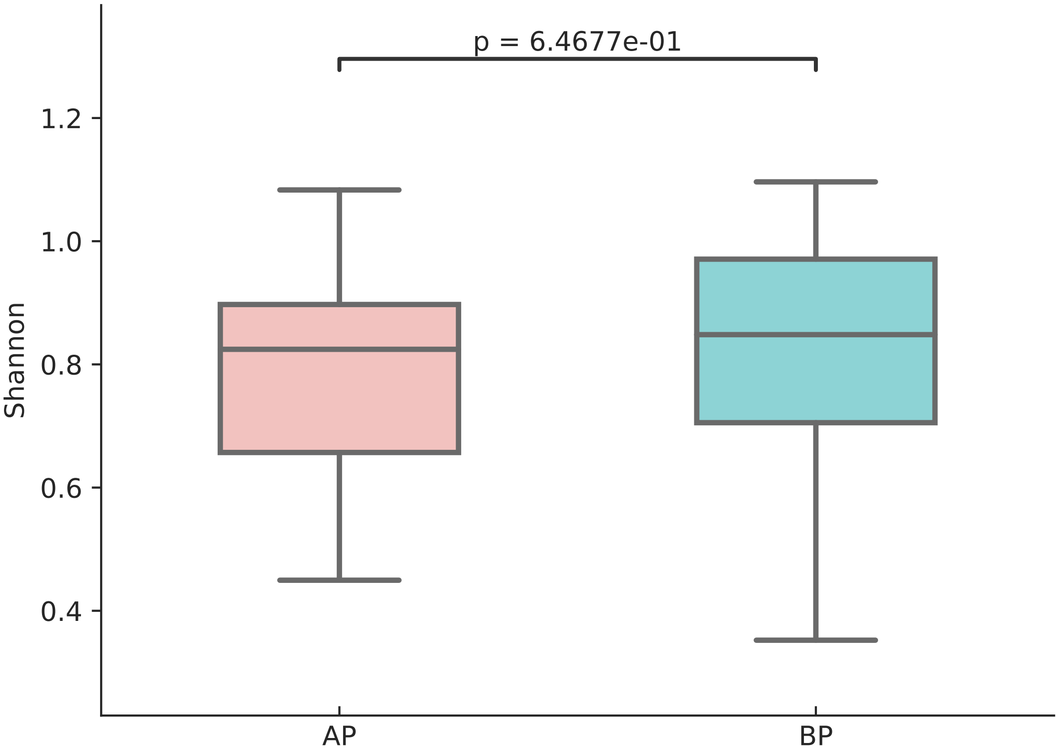

Alpha diversity of class

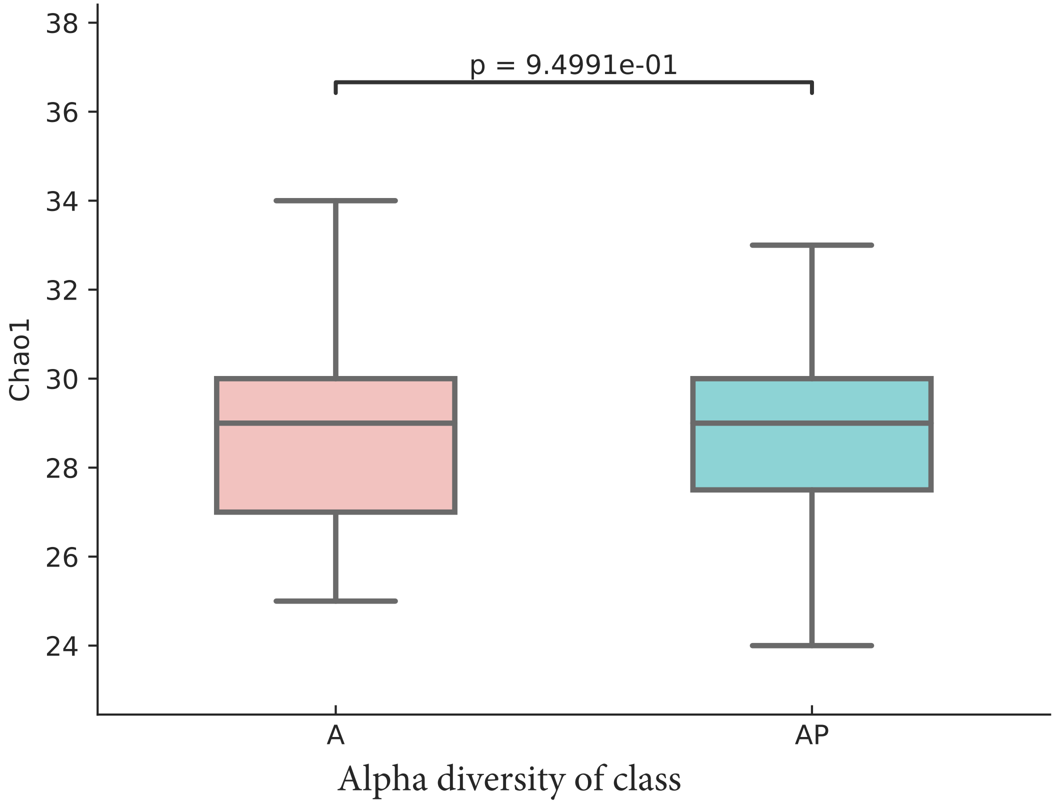
p = 9.4991e-01
Chao1
38
36
34
32
30
28
26
24
A
AP
Alpha diversity of class

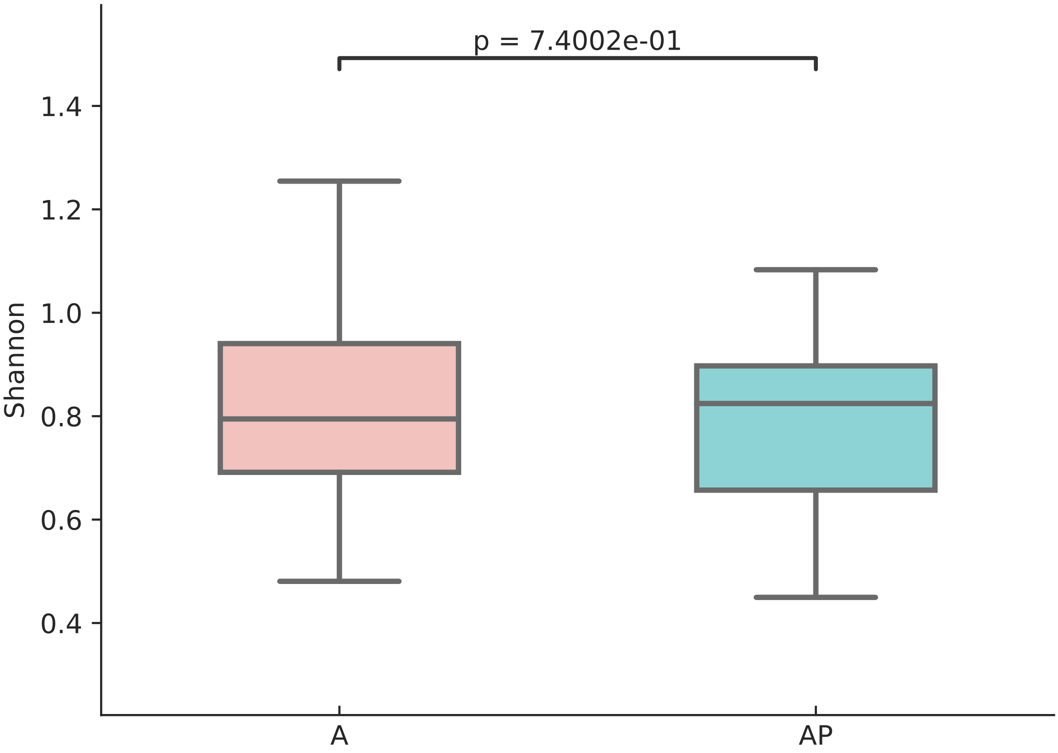

Alpha diversity of class

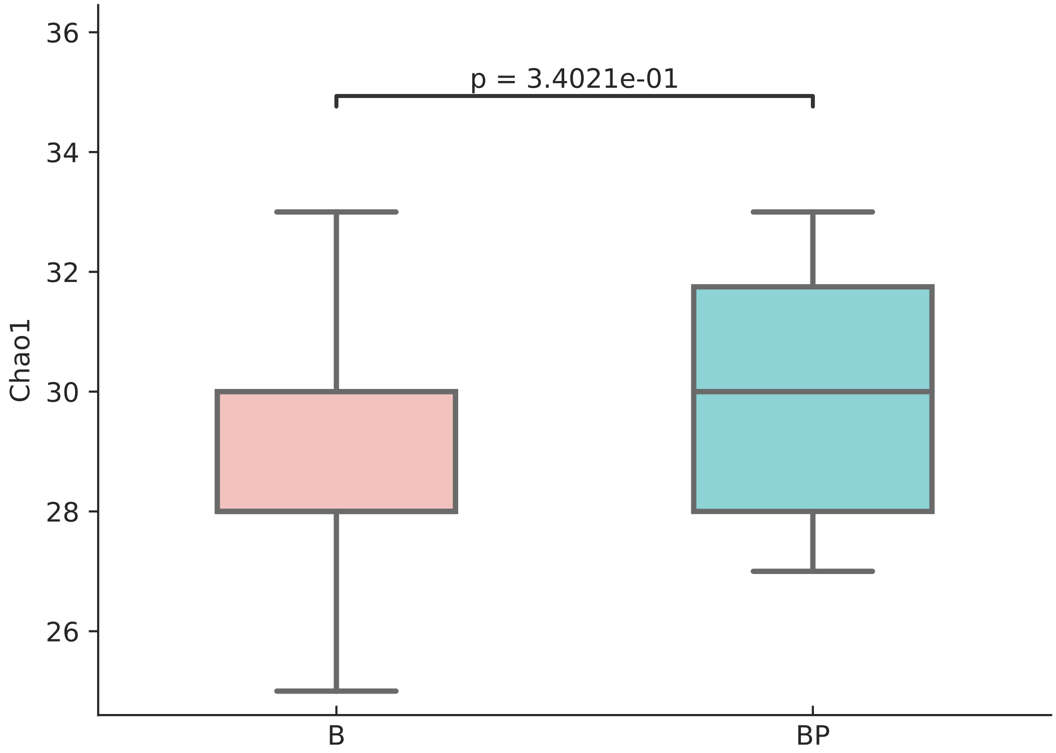

Alpha diversity of class

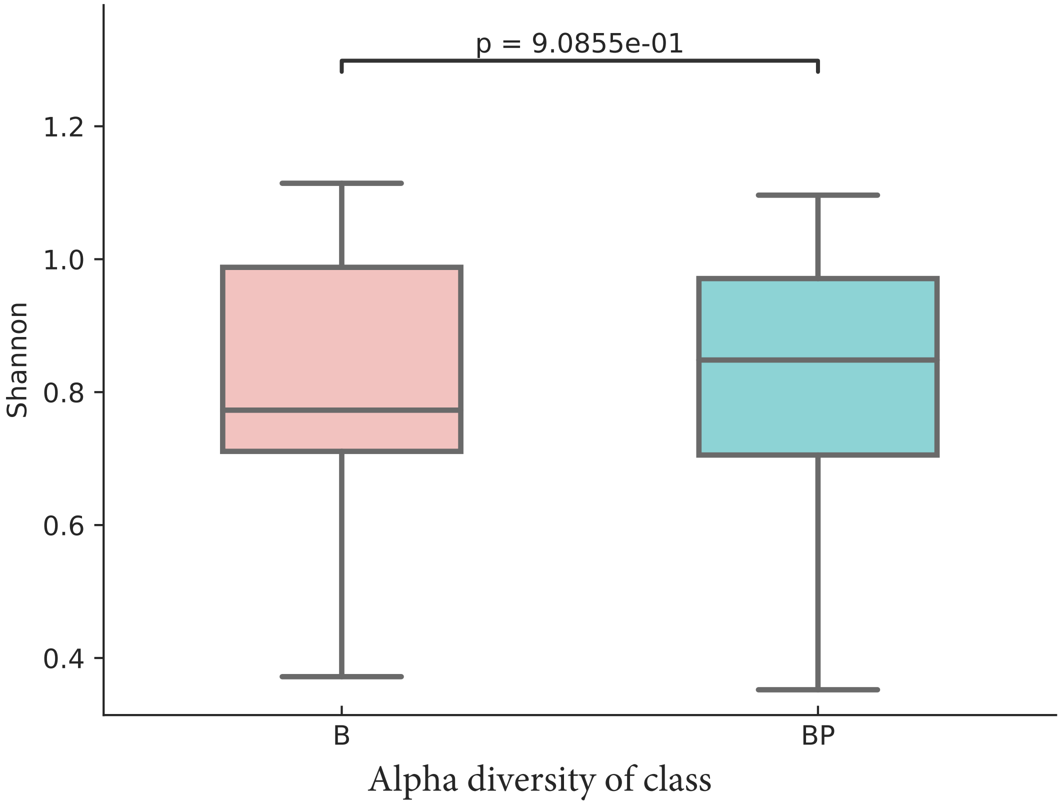
p = 9.0855e-01
Shannon
1.2
1.0
0.8
0.6
0.4
B
BP
Alpha diversity of class

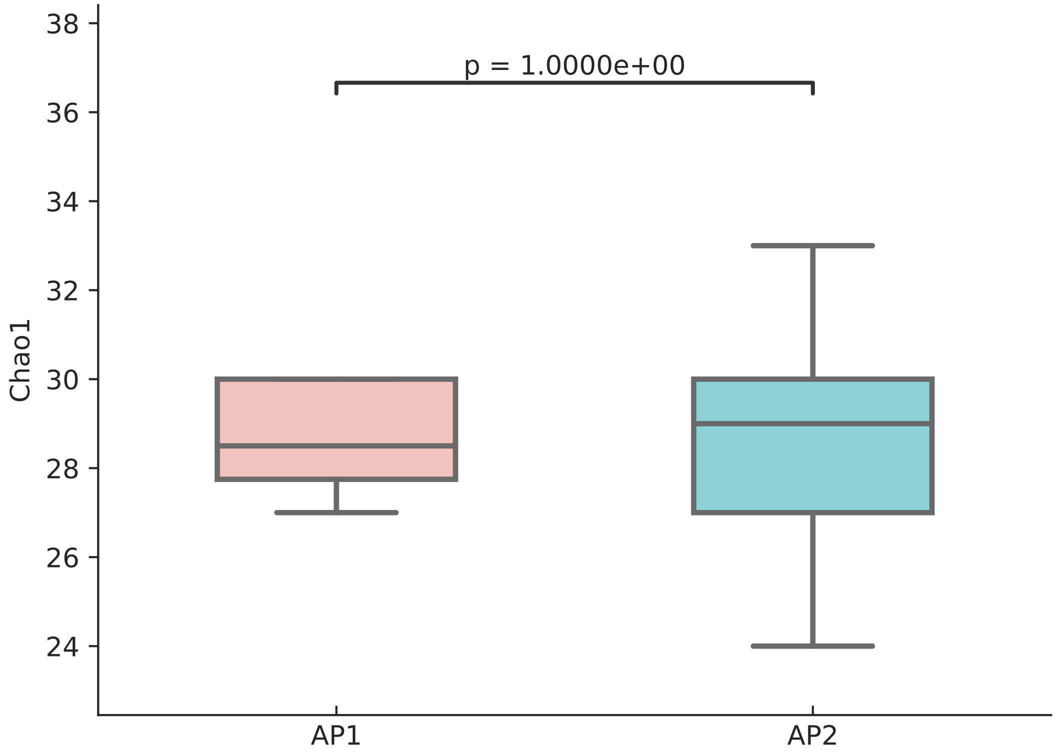

Alpha diversity of class

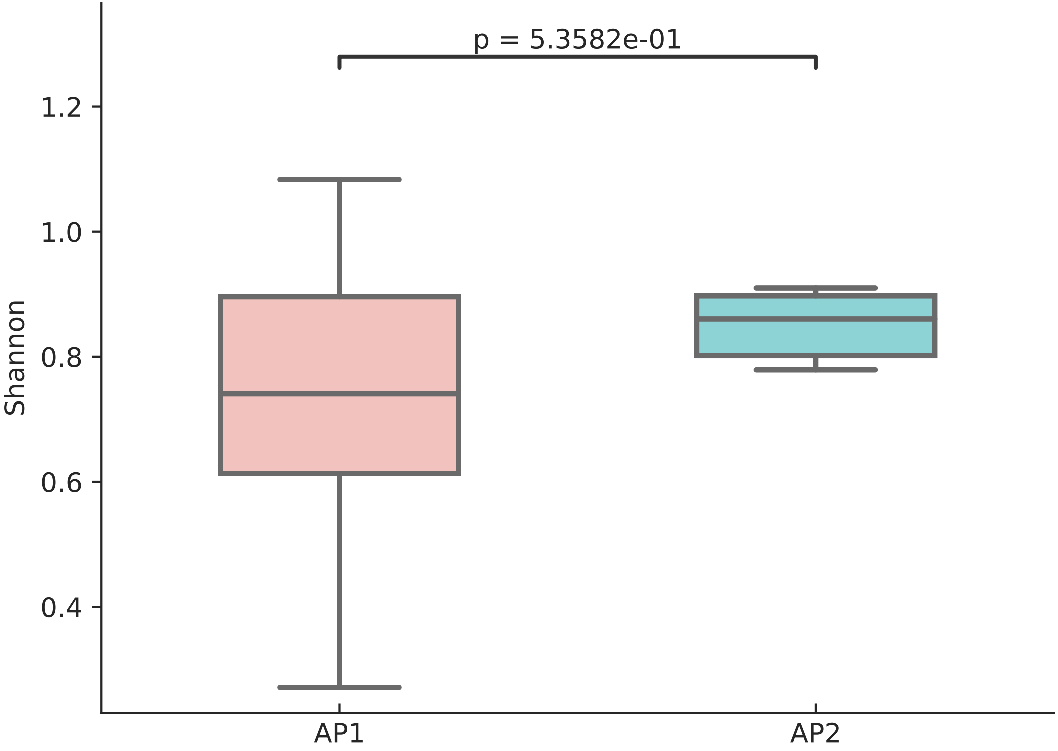

Alpha diversity of class

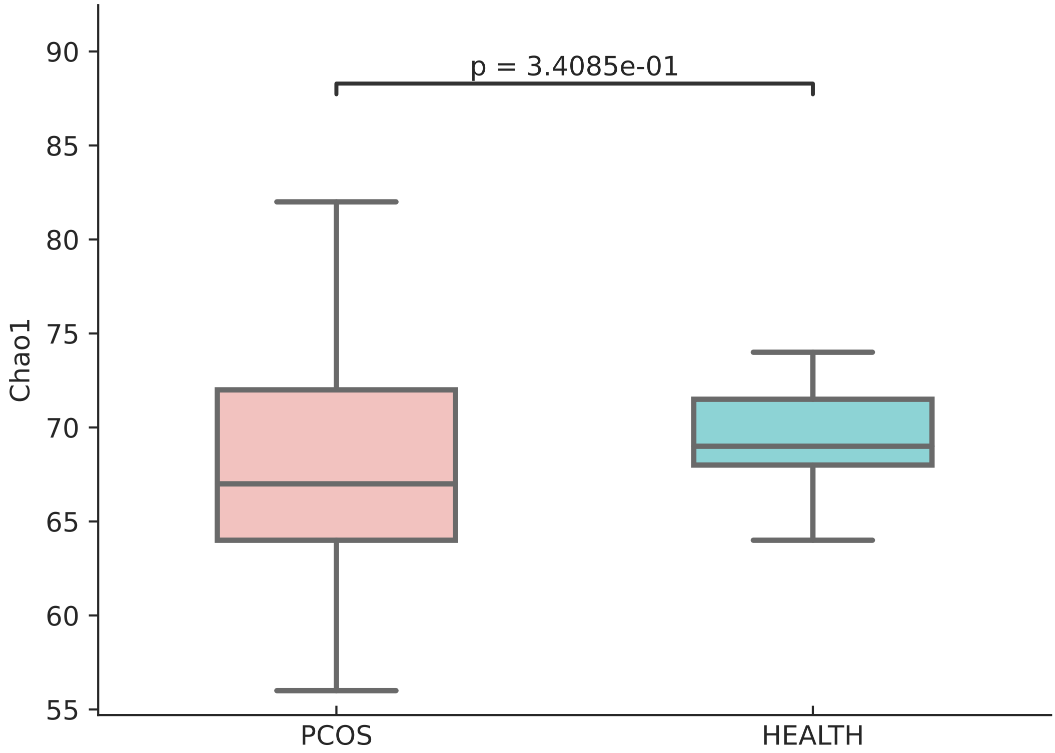

Alpha diversity of order

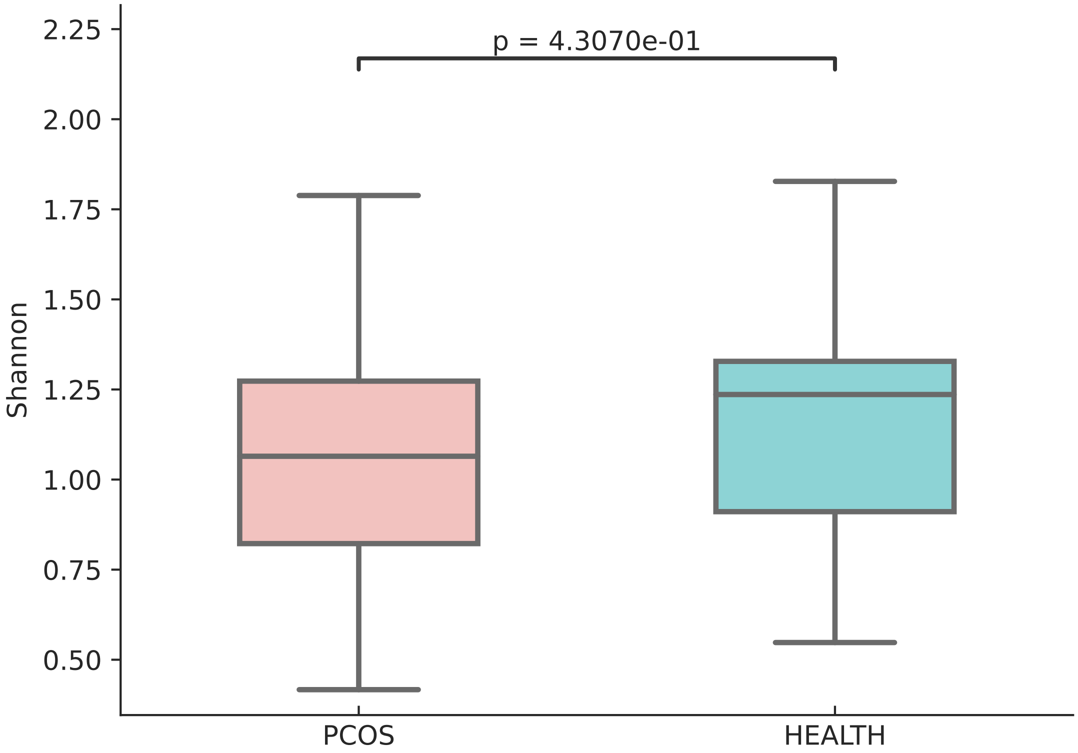

Alpha diversity of order

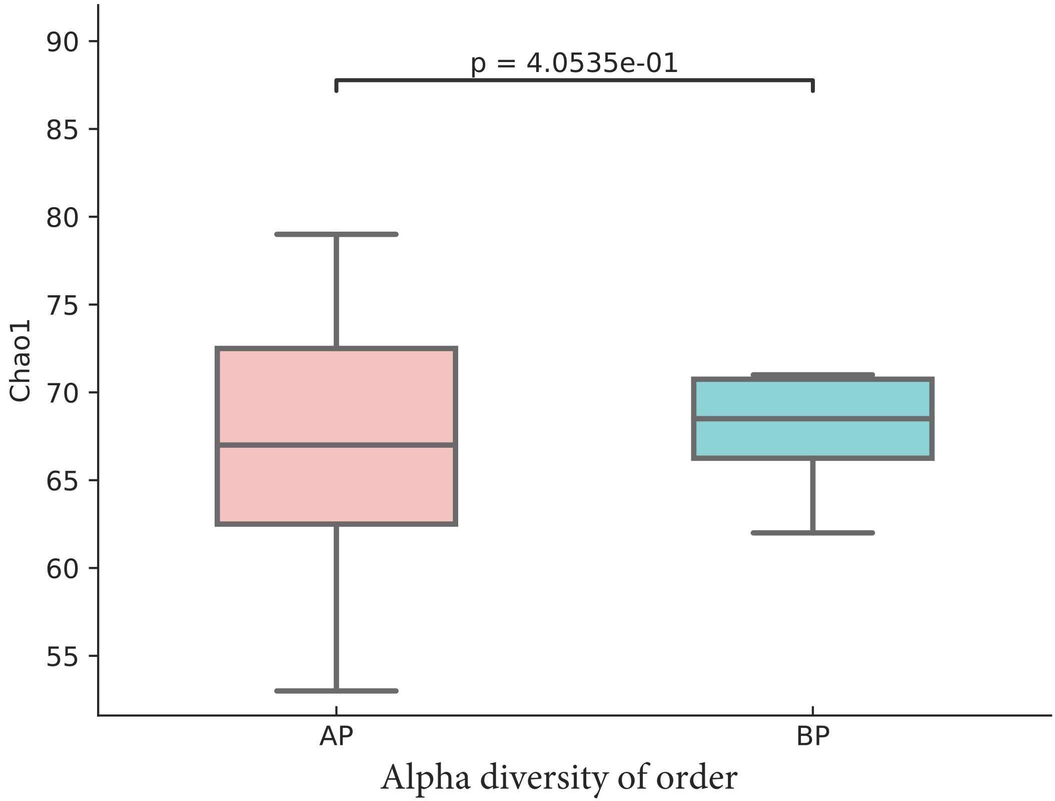
p = 4.0535e-01
90
85
80
75
70
65
60
55
Chao1
AP
BP
Alpha diversity of order

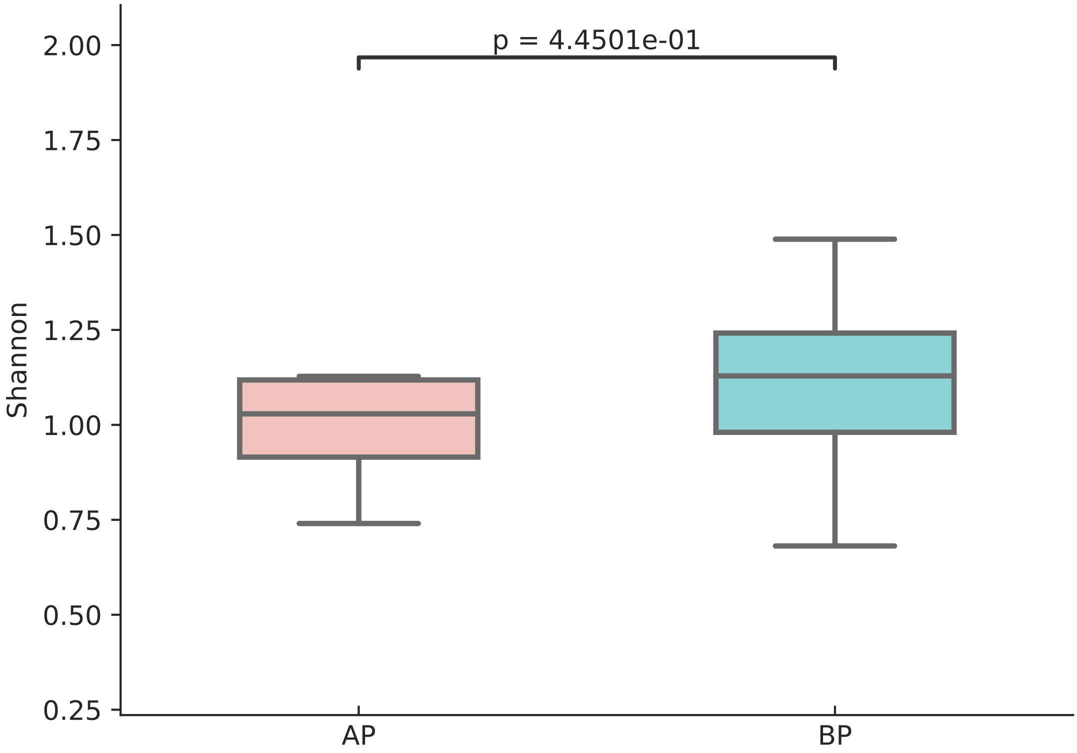

Alpha diversity of order

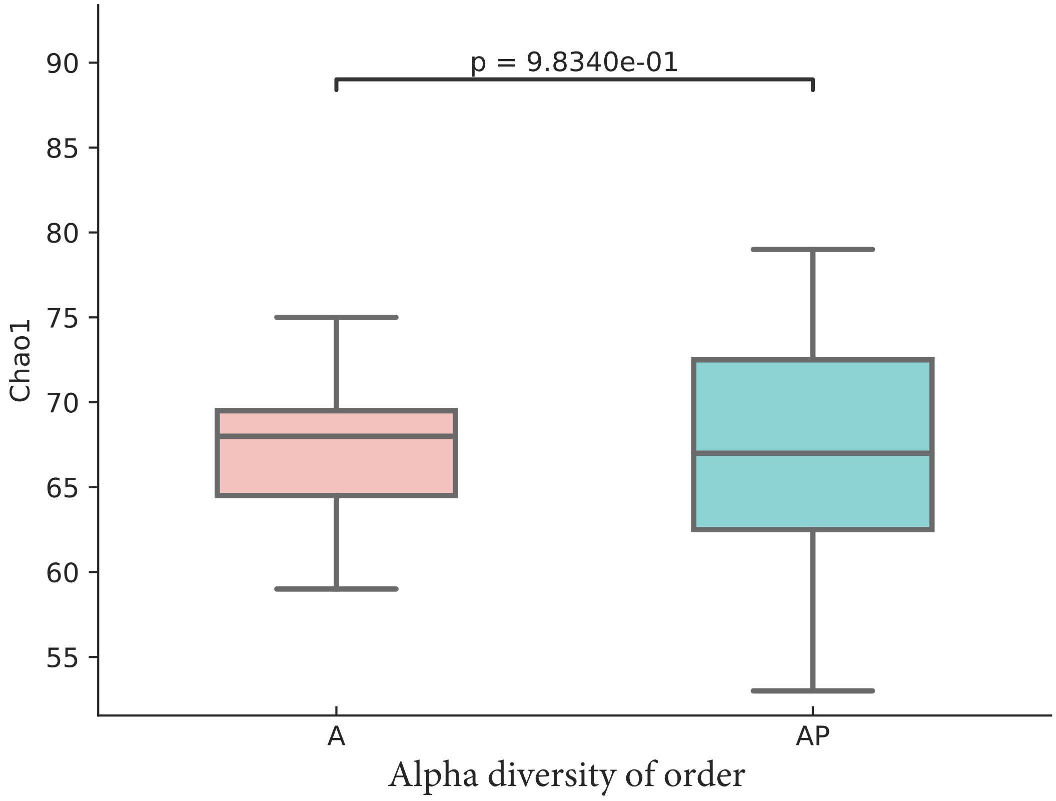
p = 9.8340e-01
Chao1
90
85
80
75
70
65
60
55
A
AP
Alpha diversity of order

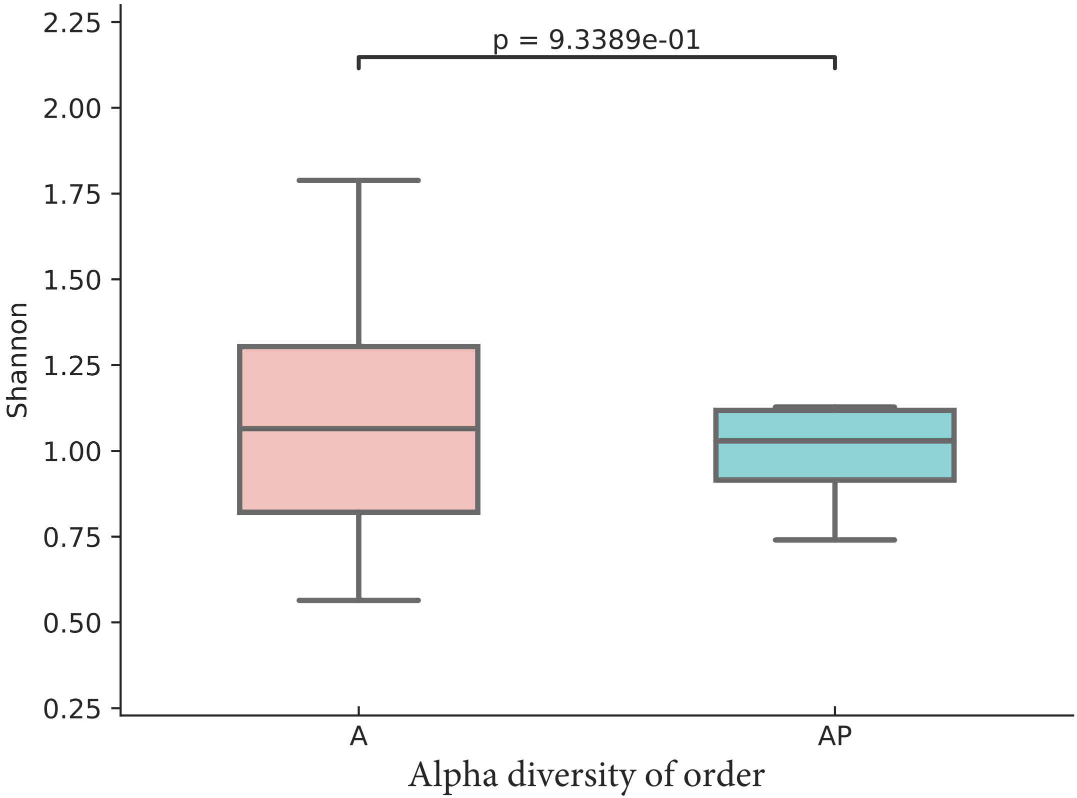

p = 9.3389e-01
Shannon
2.25
2.00
1.75
1.50
1.25
1.00
0.75
0.50
0.25
A
AP
Alpha diversity of order

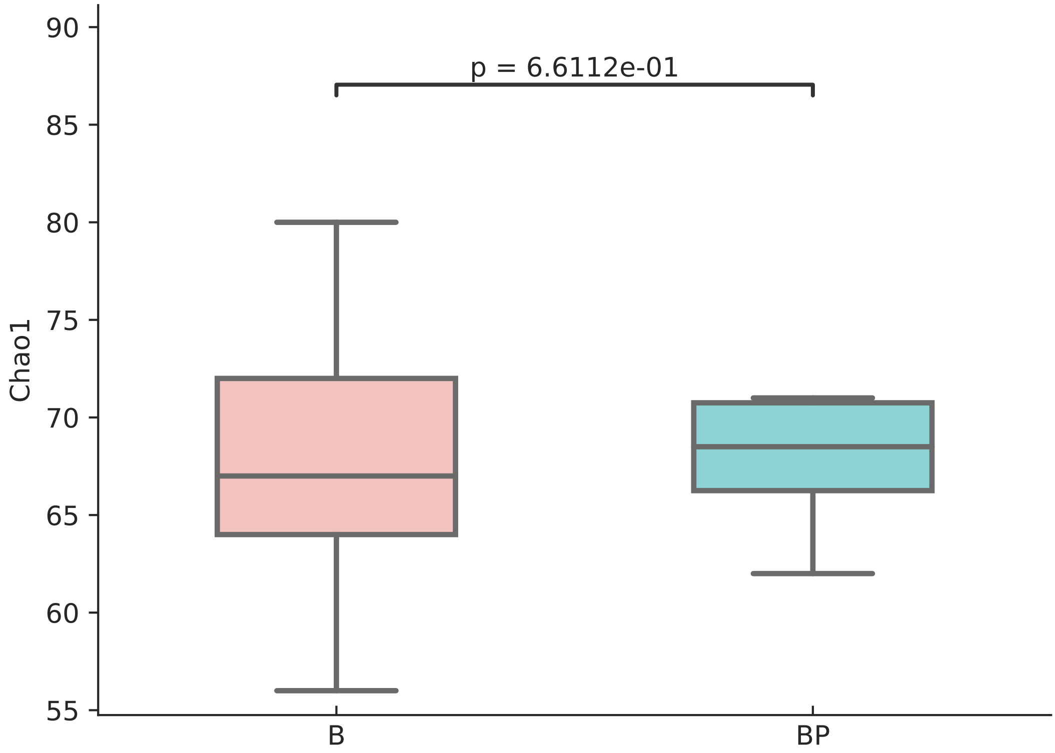

Alpha diversity of order

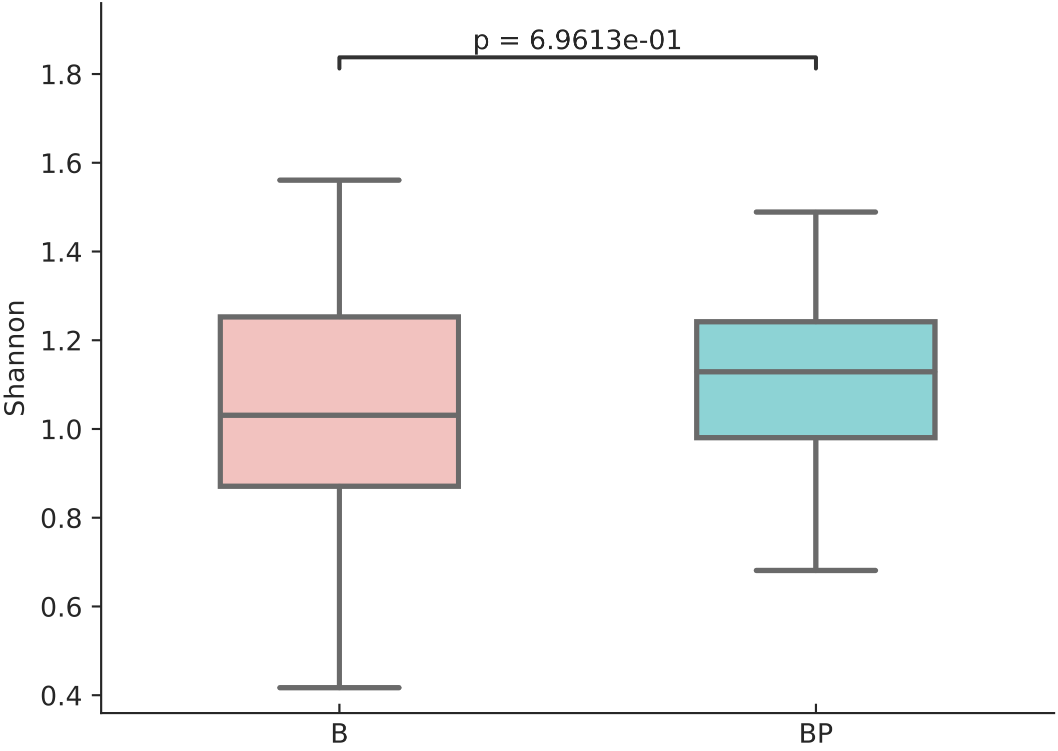

Alpha diversity of order

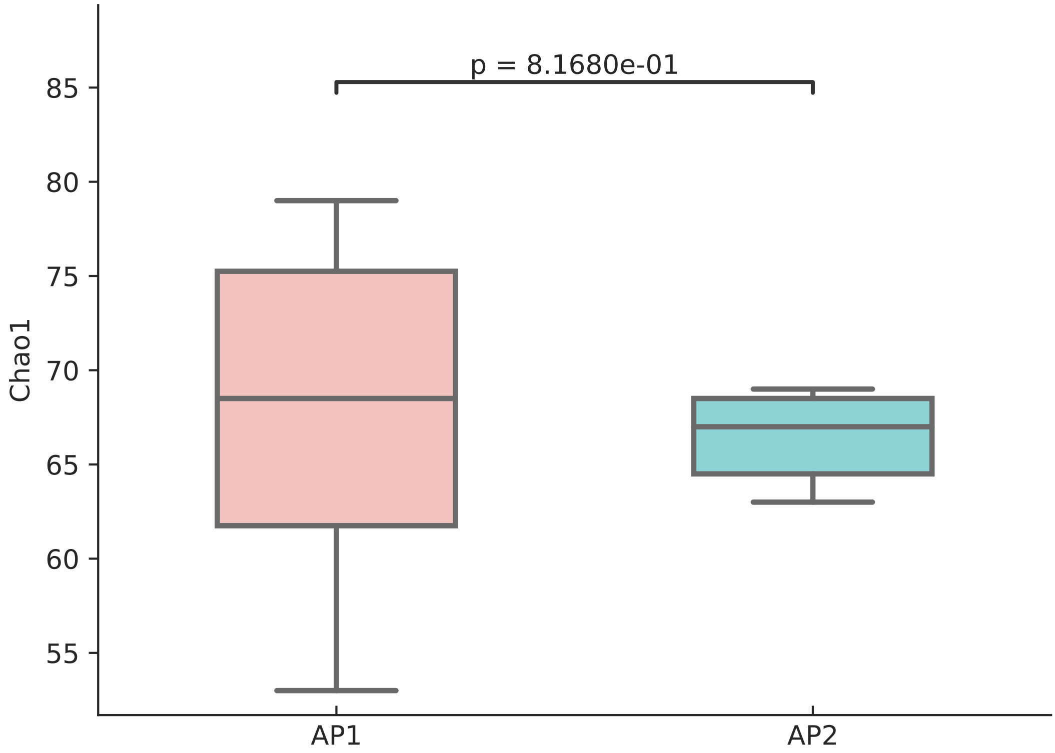

Alpha diversity of order

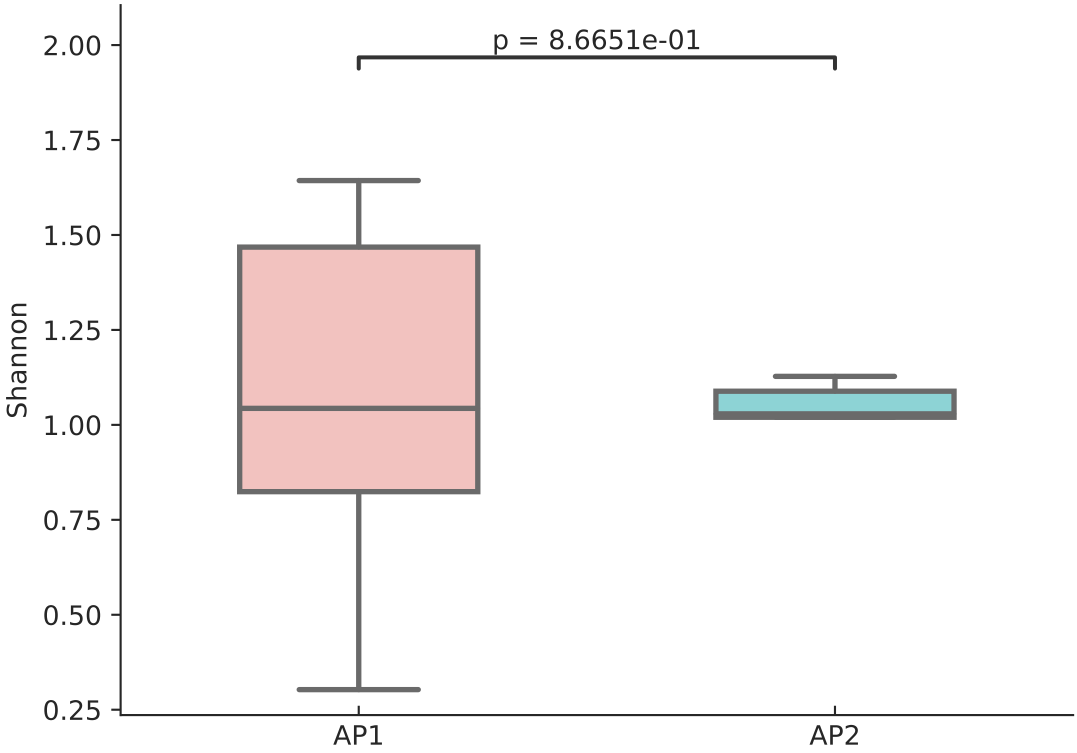

Alpha diversity of order

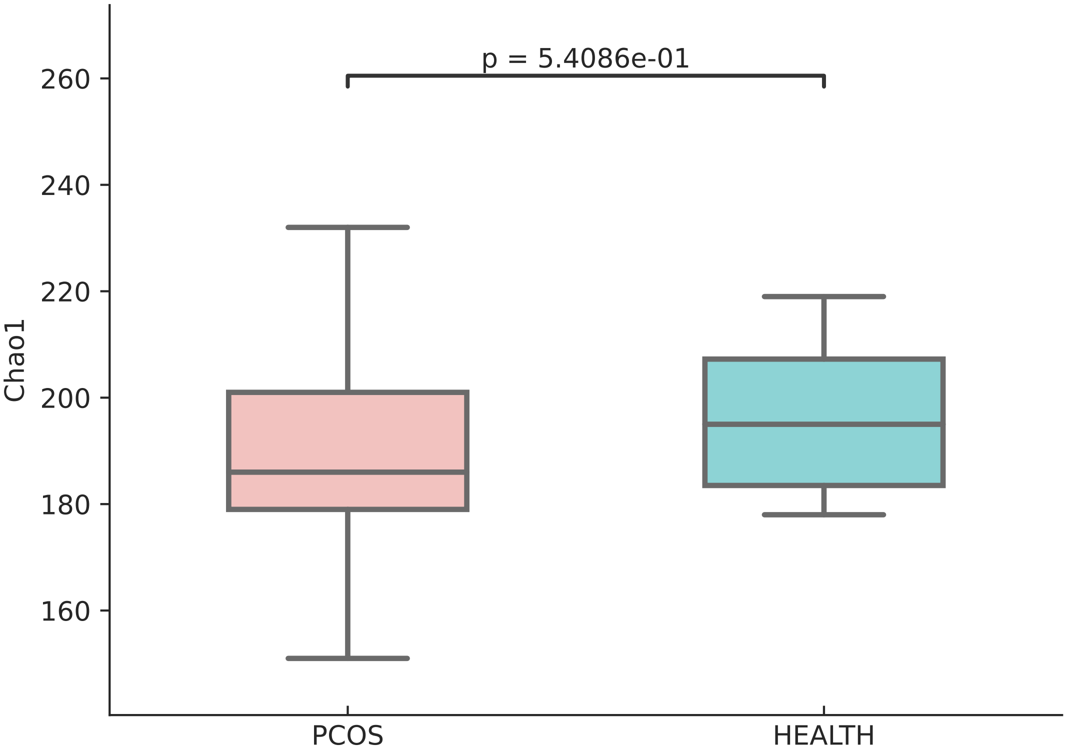

Alpha diversity of famliy

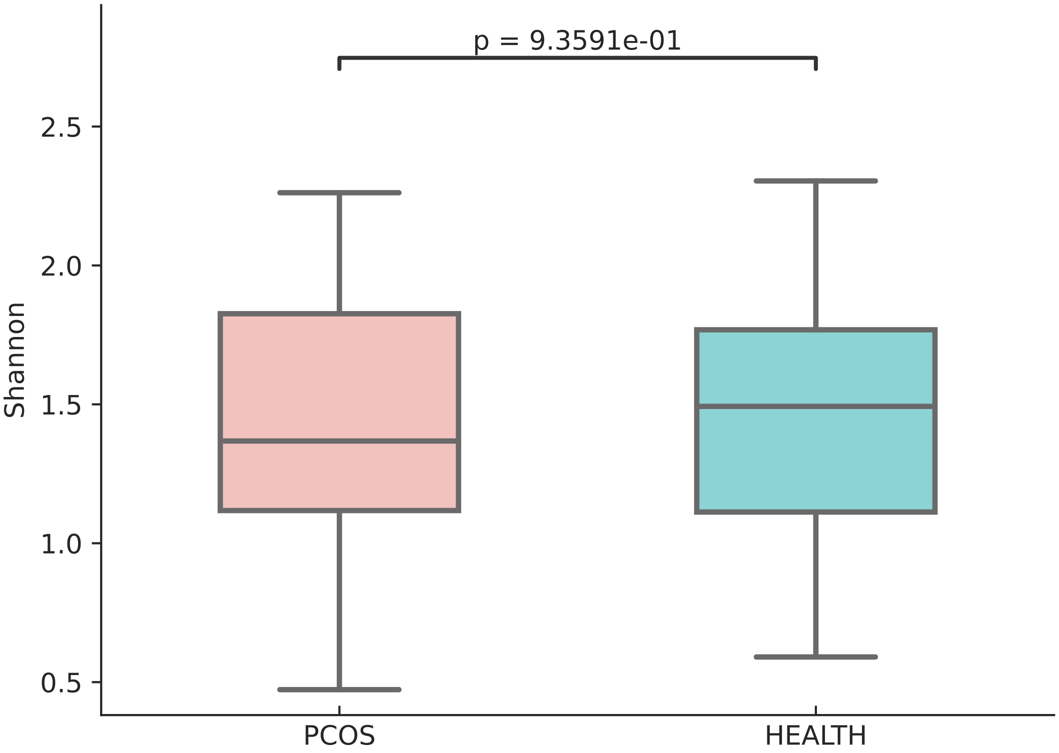

Alpha diversity of famliy

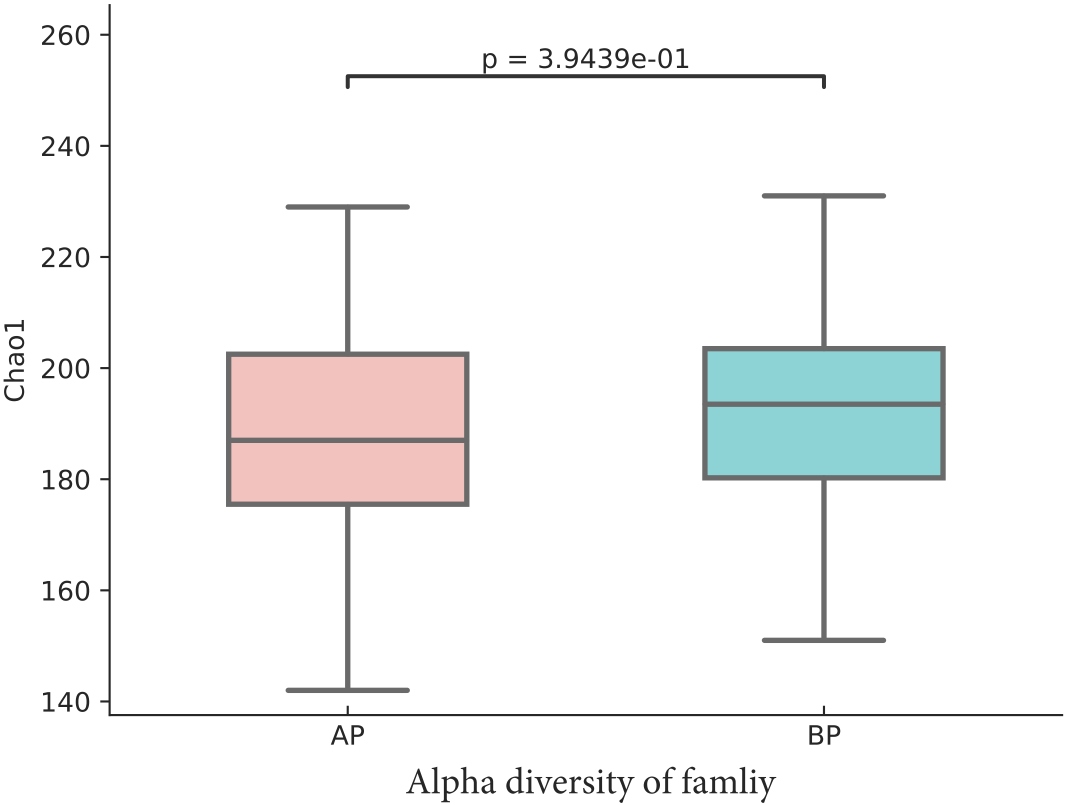

p = 3.9439e-01
260
240
220
200
180
160
140
Chao1
AP
BP
Alpha diversity of famliy

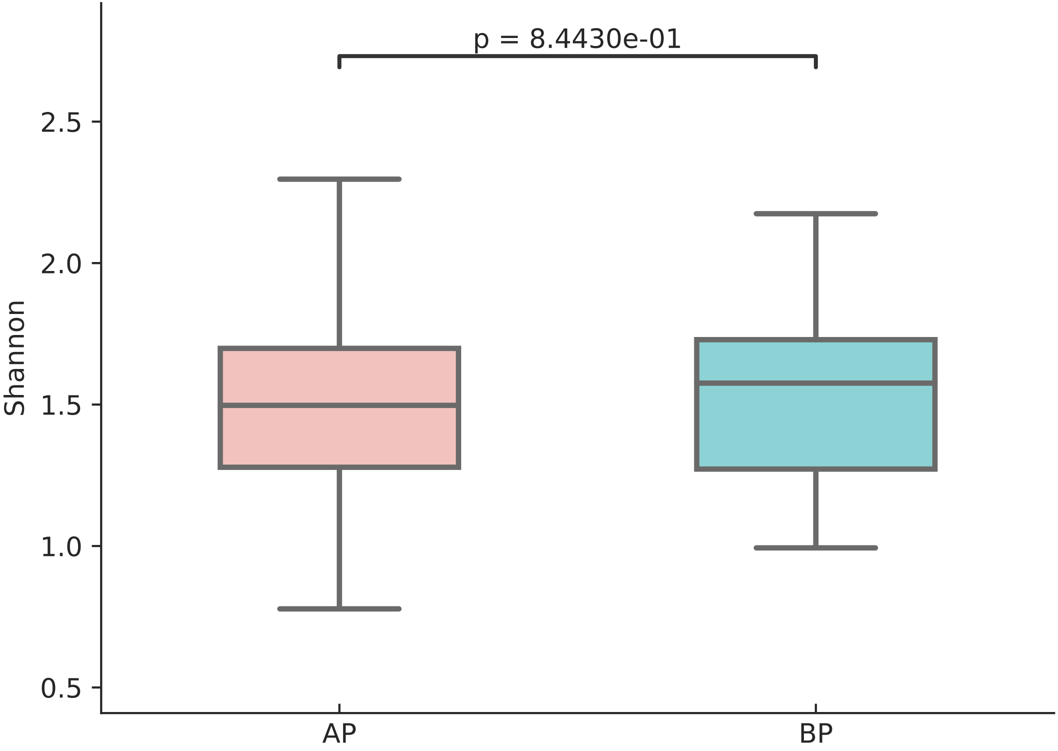

Alpha diversity of famliy

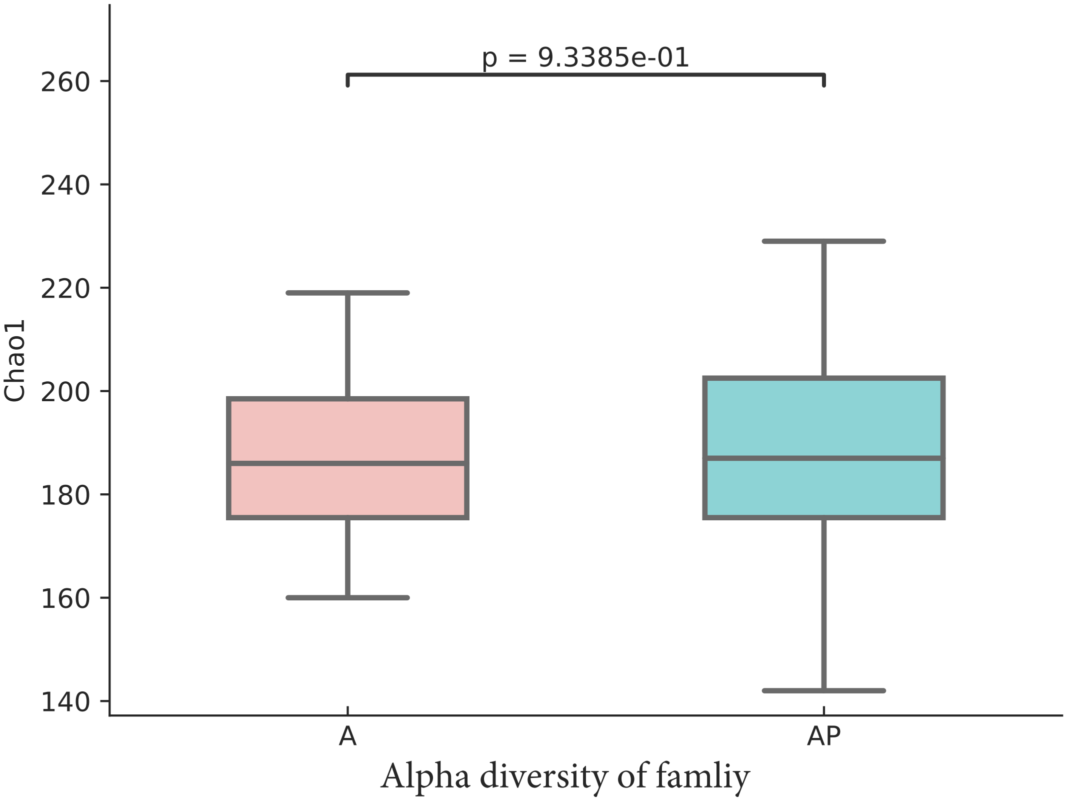
p = 9.3385e-01
260
240
220
200
180
160
140
Chao1
A
AP
Alpha diversity of famliy

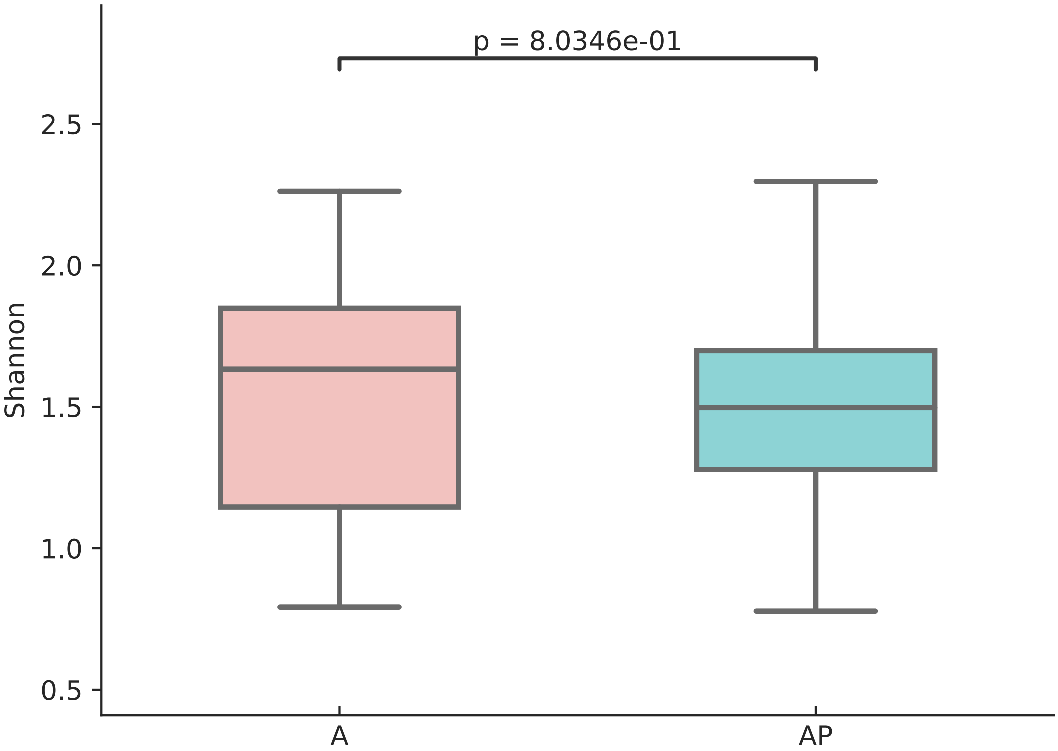

Alpha diversity of famliy

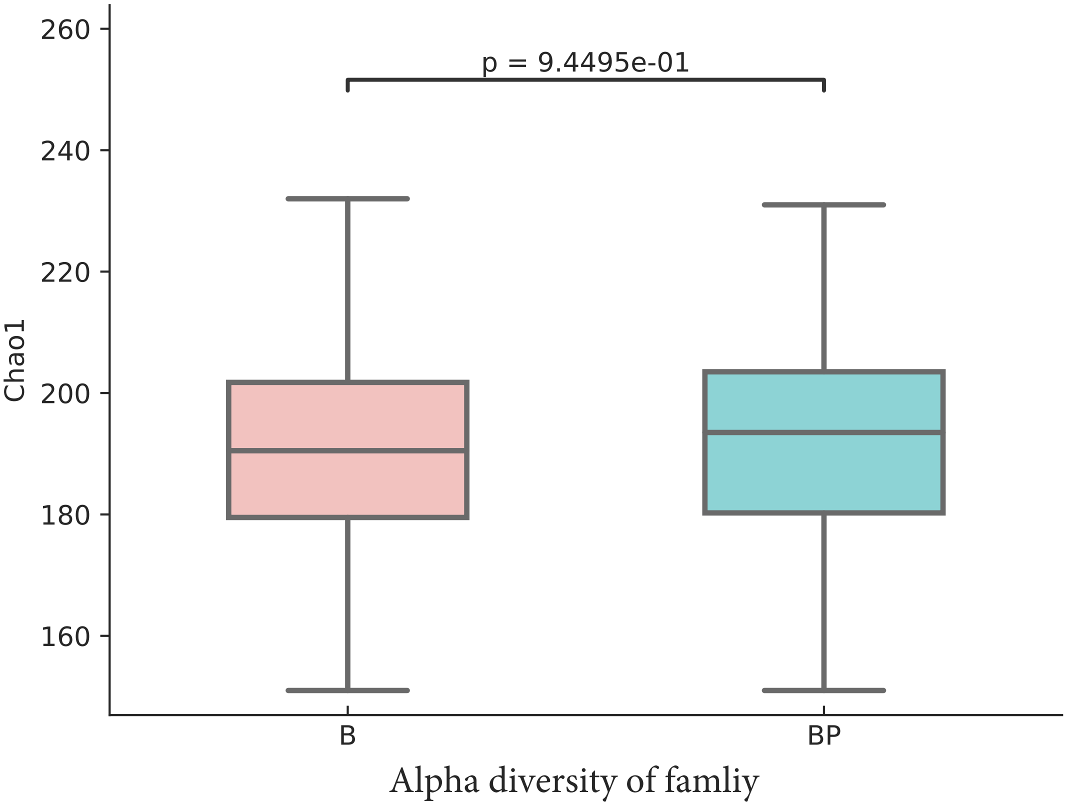
p = 9.4495e-01
Chao1
260
240
220
200
180
160
B
BP
Alpha diversity of famliy

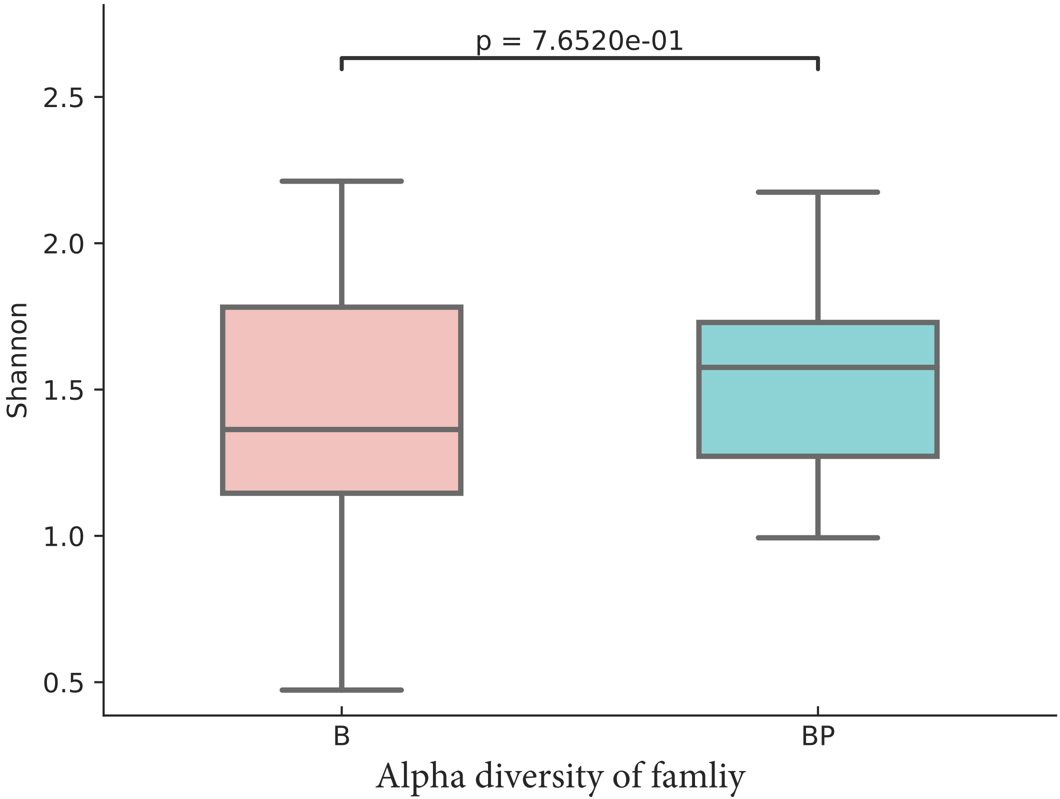
p = 7.6520e-01
2.5
2.0
1.5
1.0
0.5
Shannon
B
BP
Alpha diversity of famliy

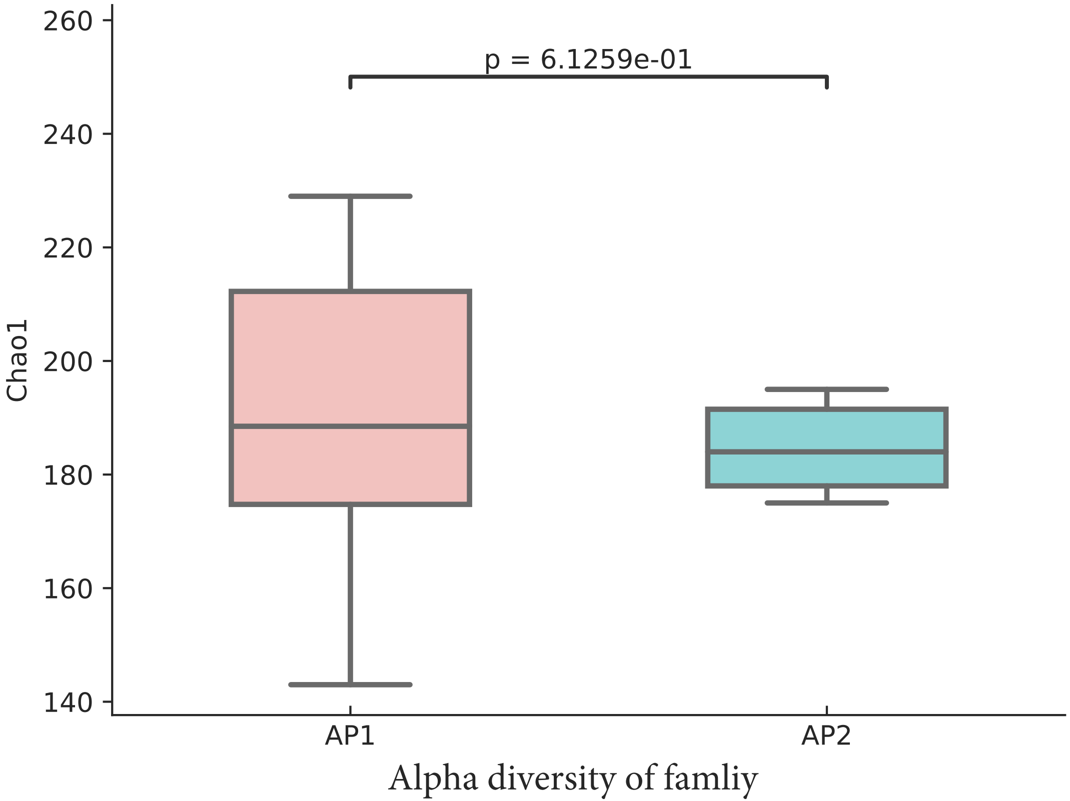

p = 6.1259e-01
260
240
220
200
180
160
140
Chao1
AP1
AP2
Alpha diversity of famliy

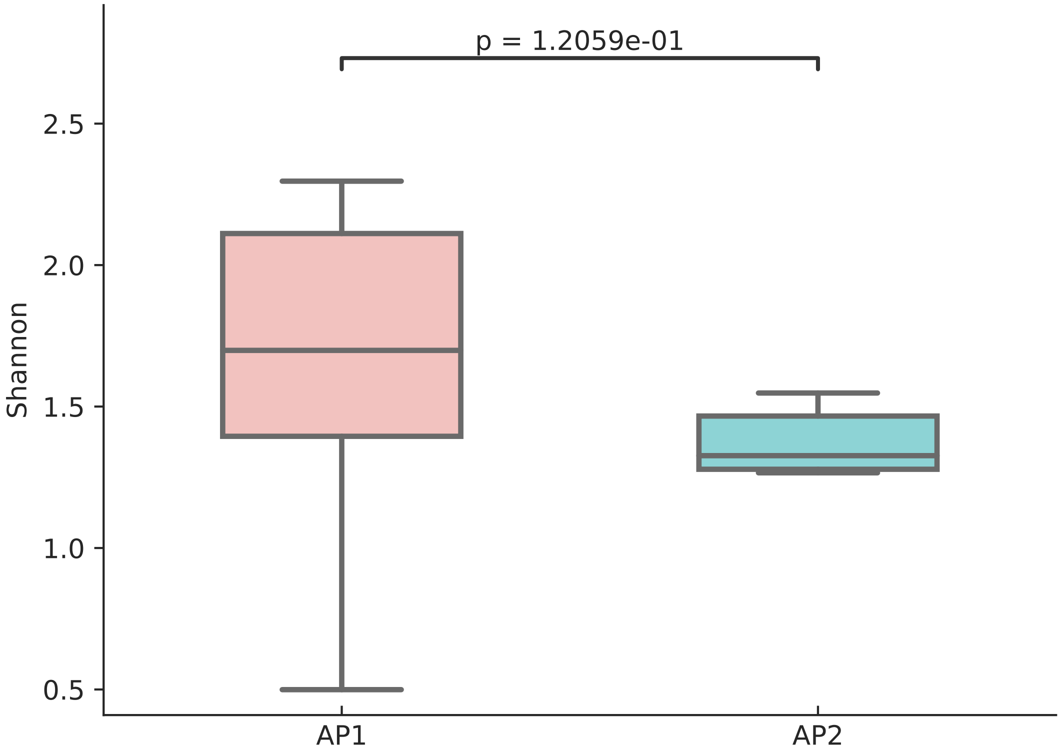

Alpha diversity of famliy

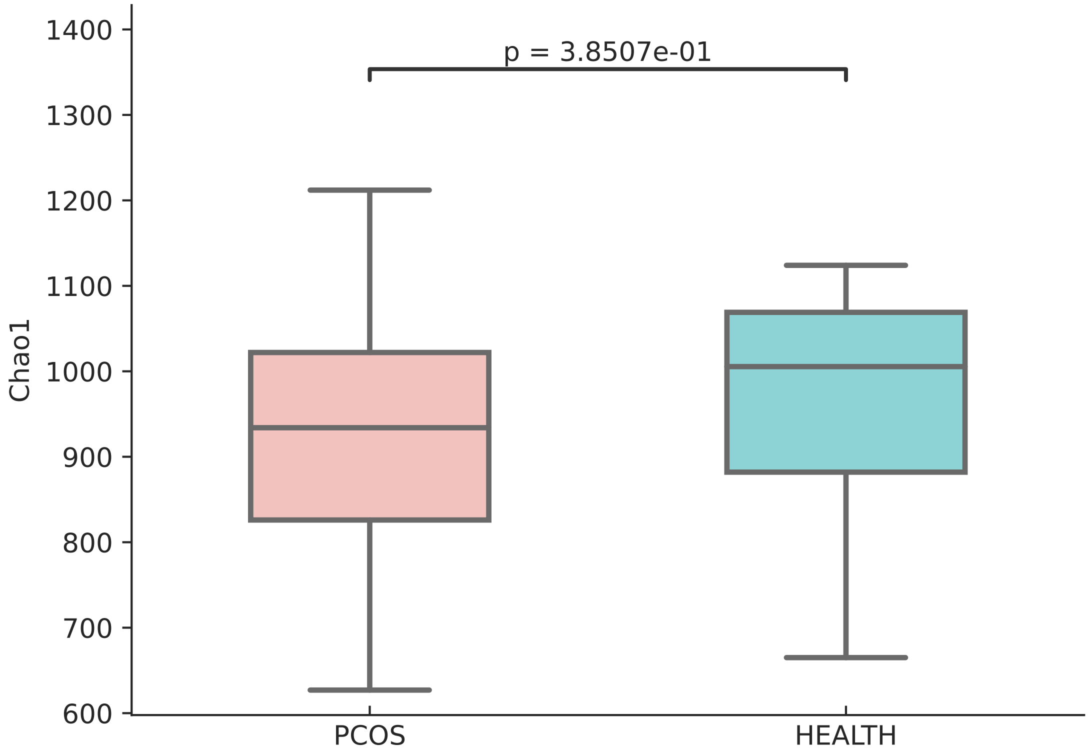

Alpha diversity of genus

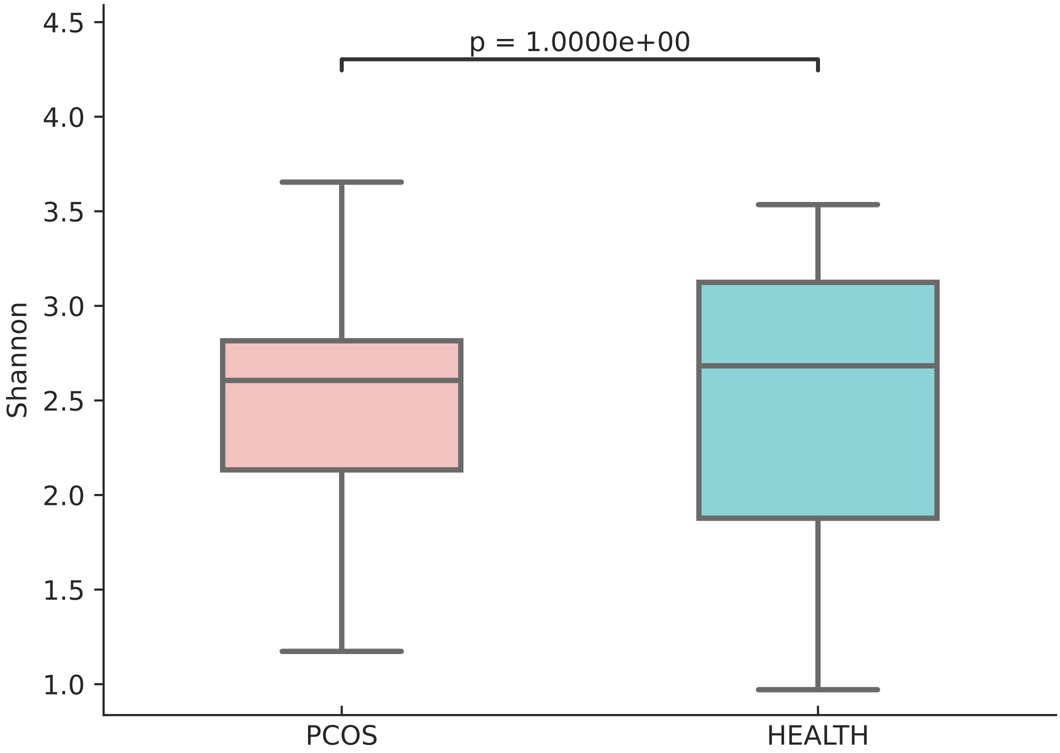

Alpha diversity of genus

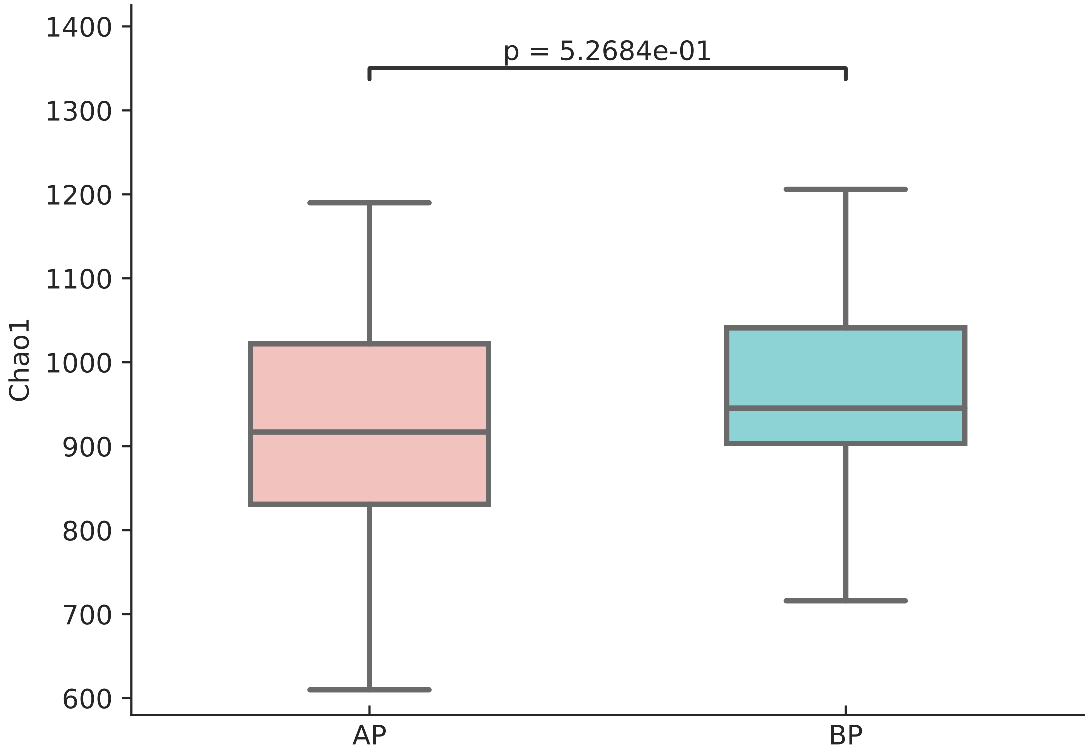

Alpha diversity of genus

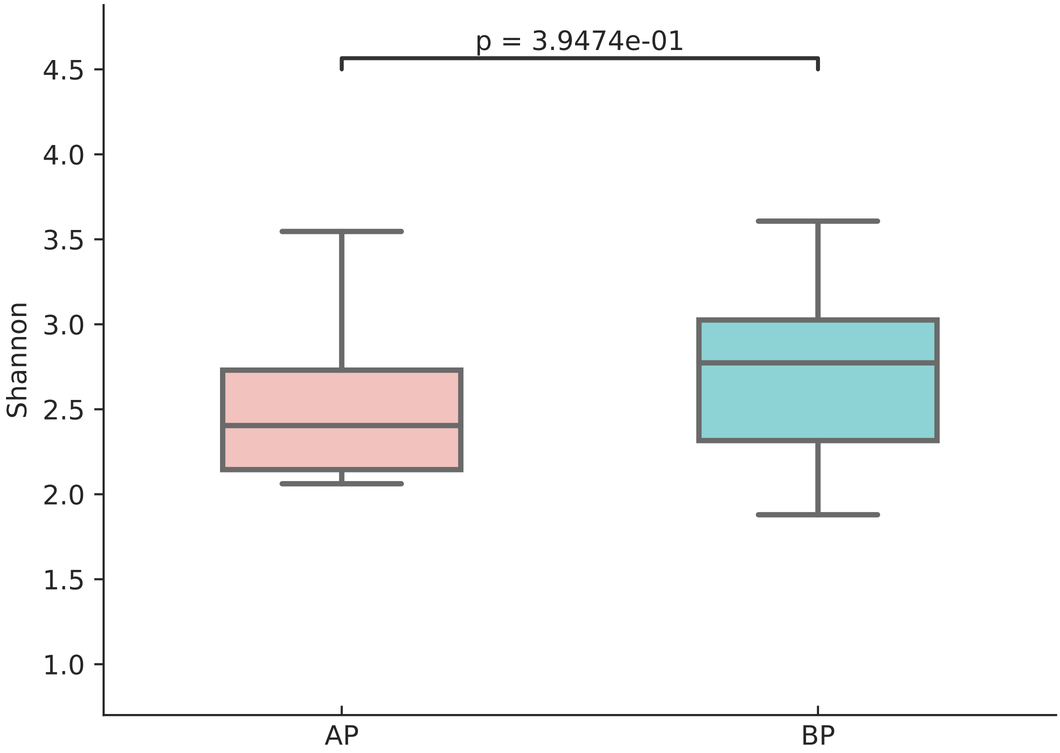

Alpha diversity of genus

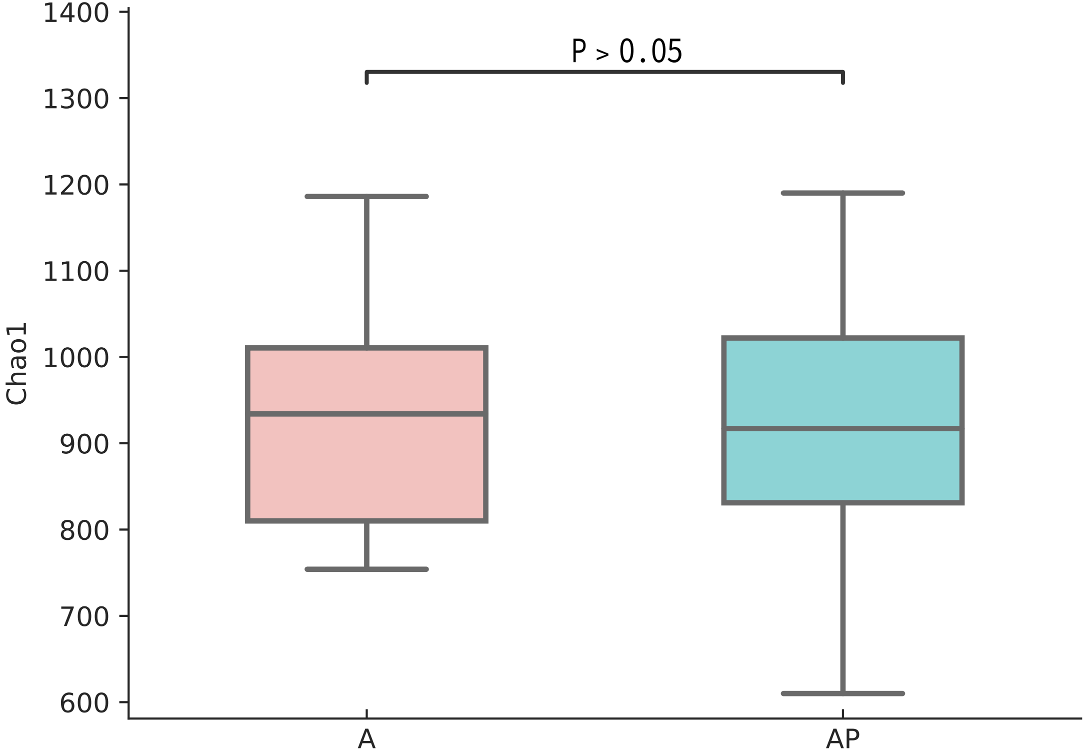

Alpha diversity of genus
b

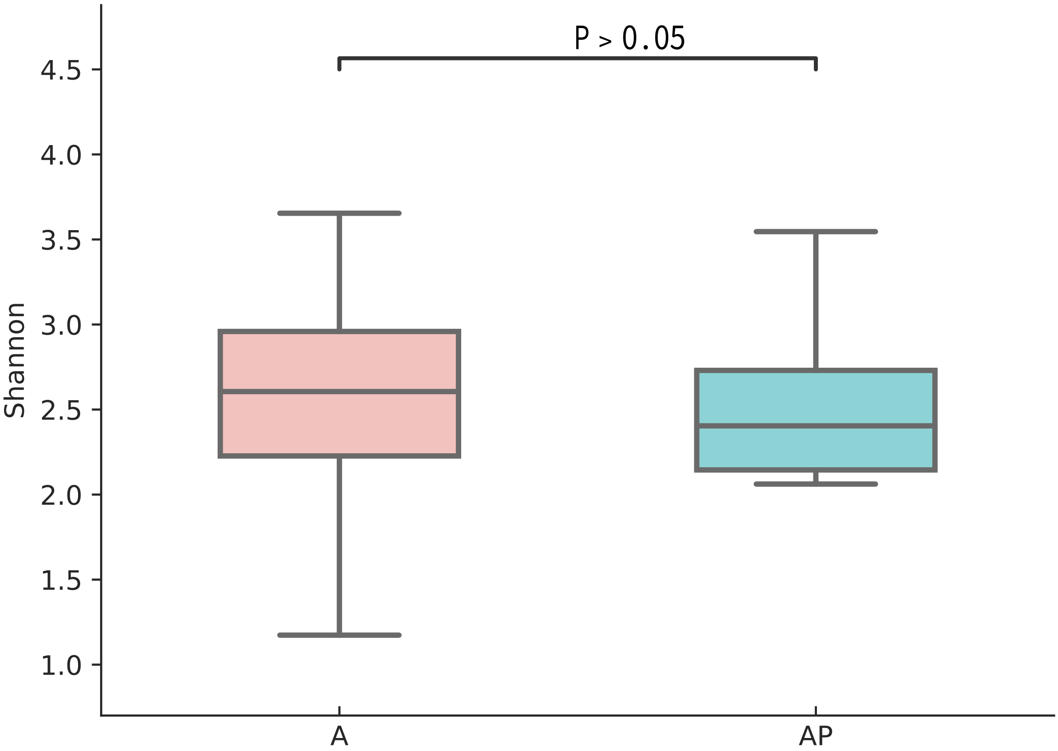

Alpha diversity of genus

b

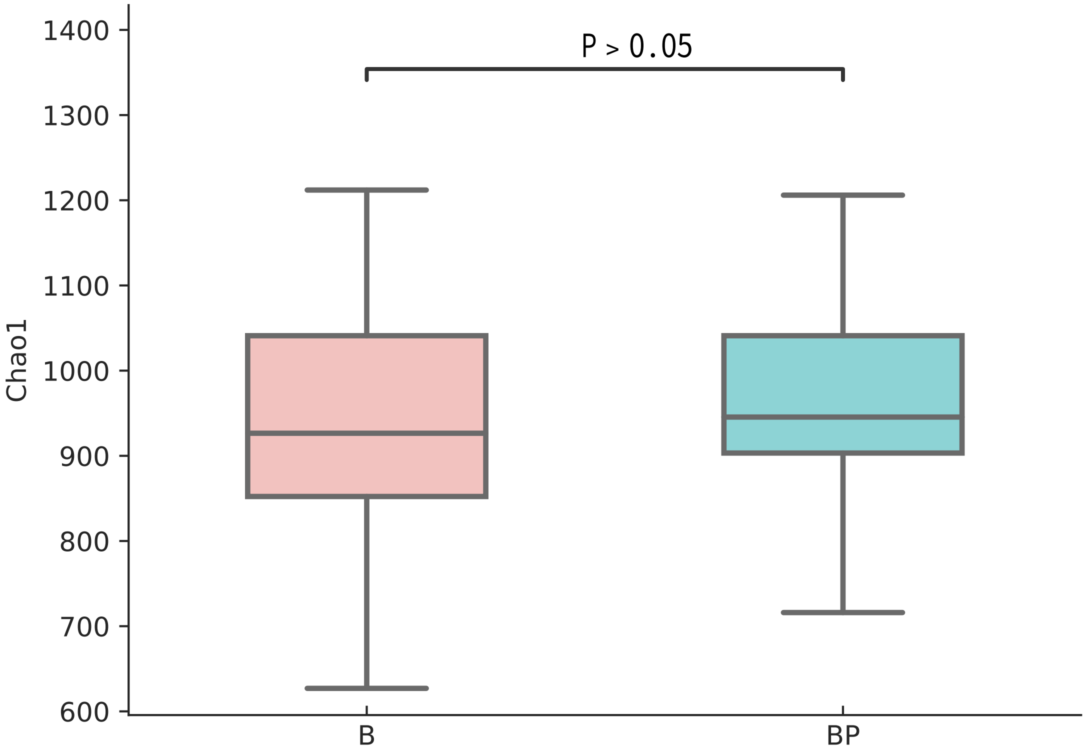

b

Alpha diversity of genus

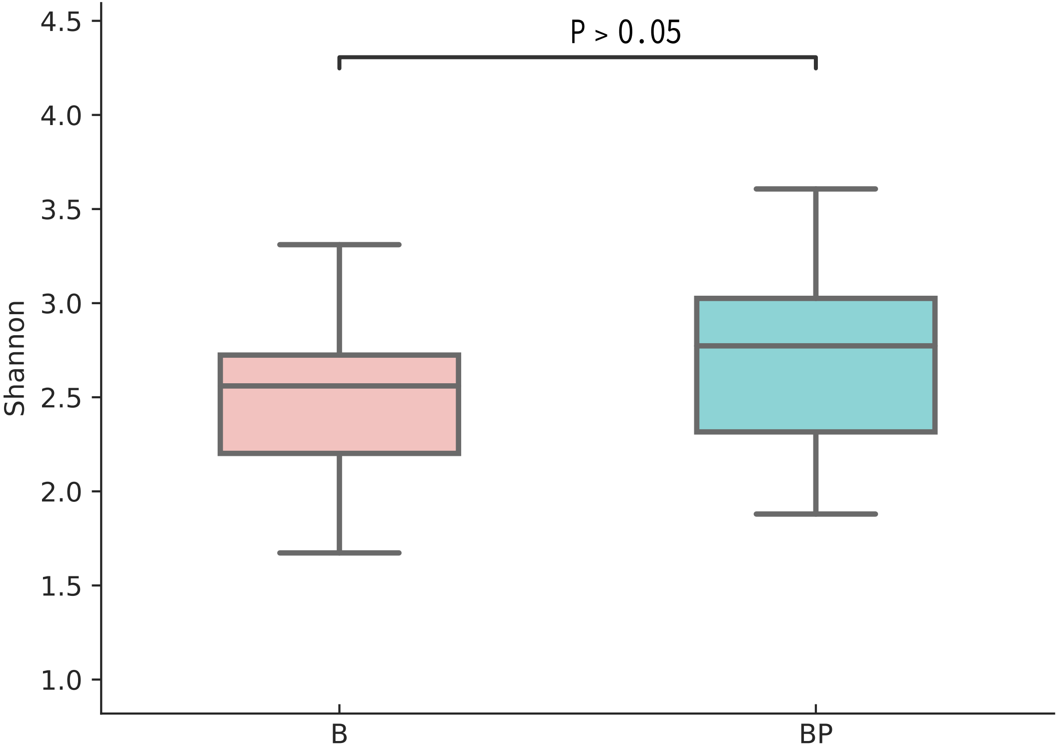

b

Alpha diversity of genus

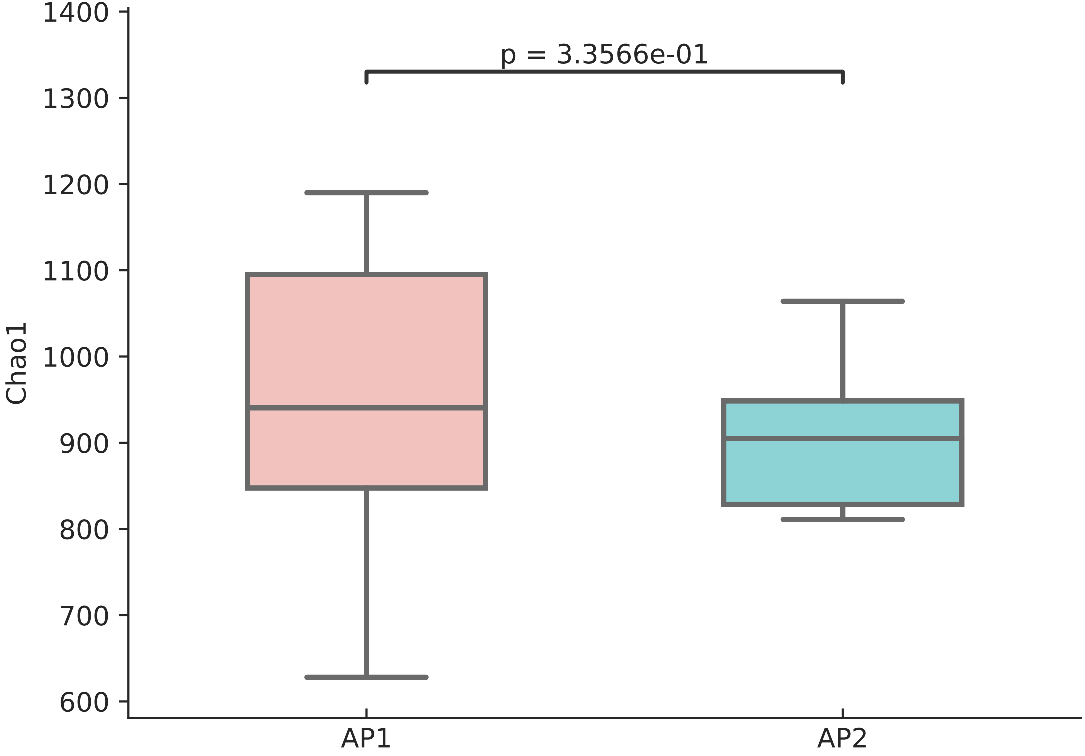

Alpha diversity of genus

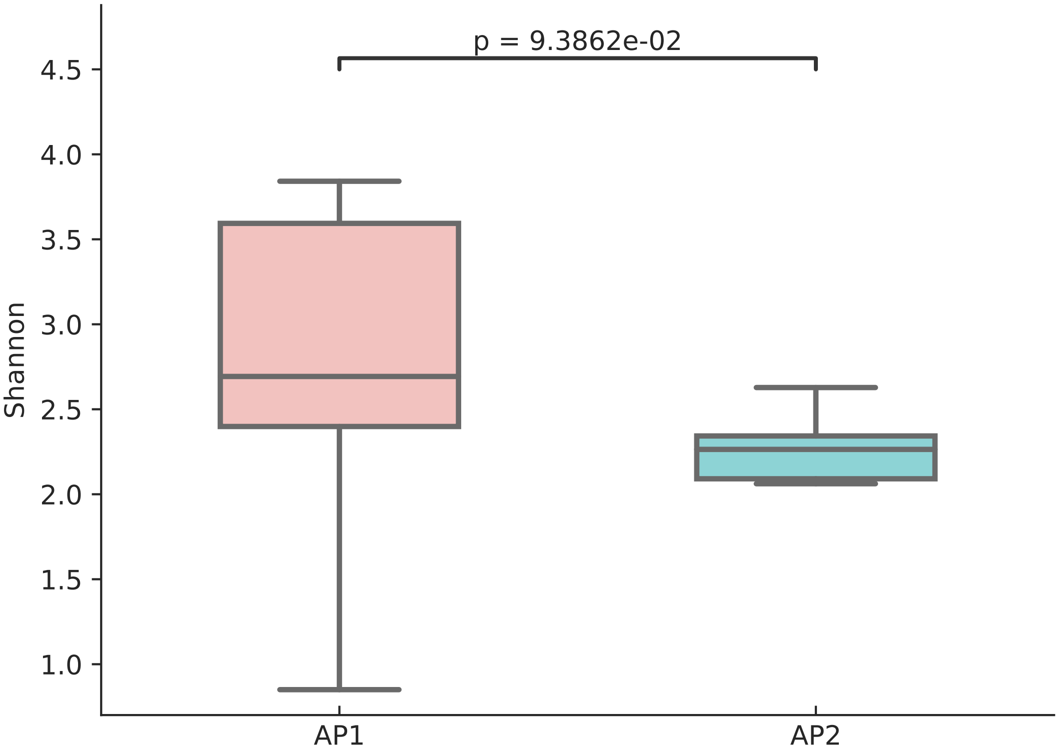

Alpha diversity of genus

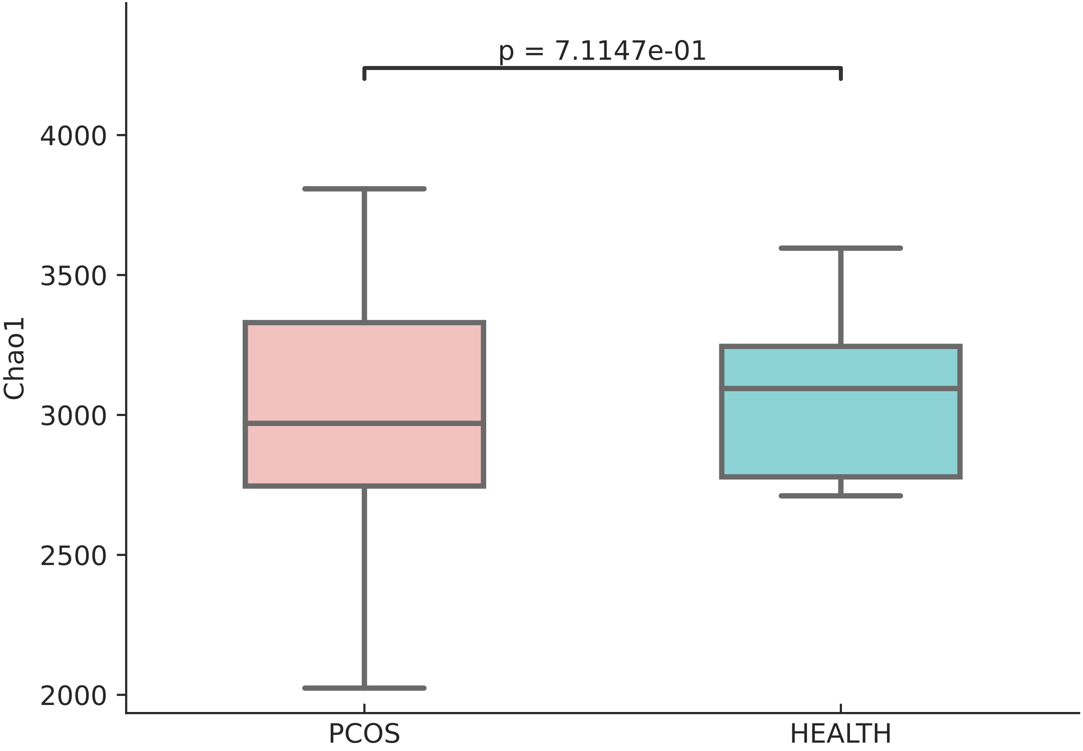

Alpha diversity of species

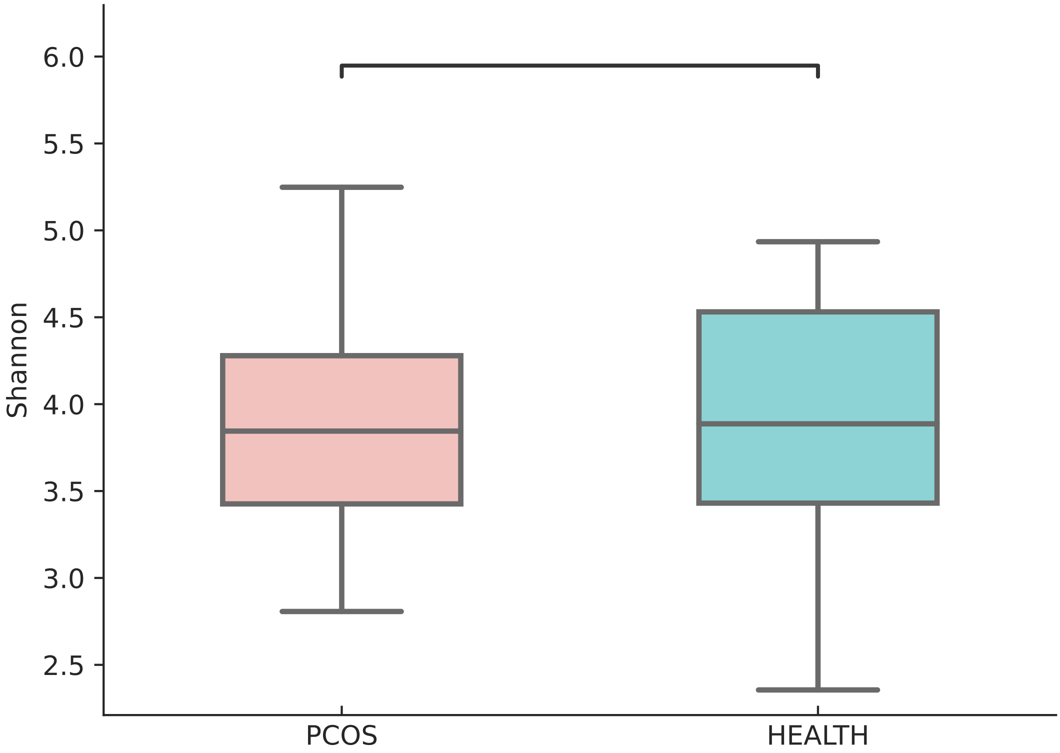

Alpha diversity of species

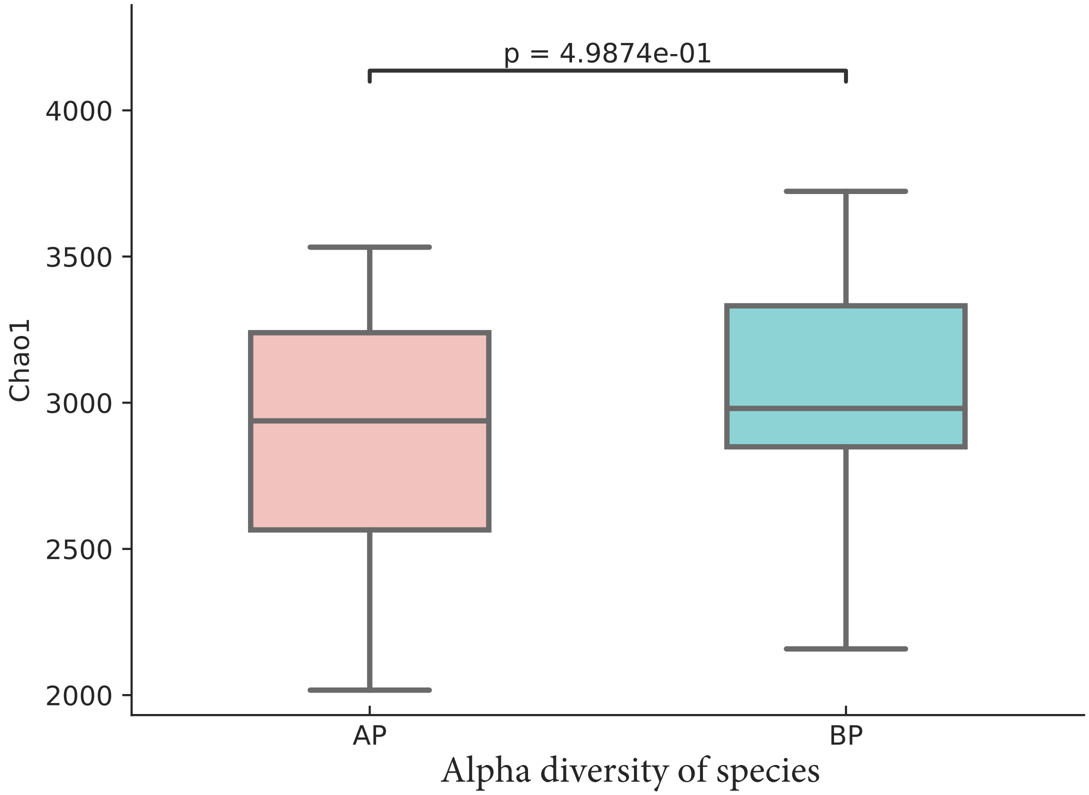
p = 4.9874e-01
4000
3500
3000
2500
2000
Chao1
AP
BP
Alpha diversity of species

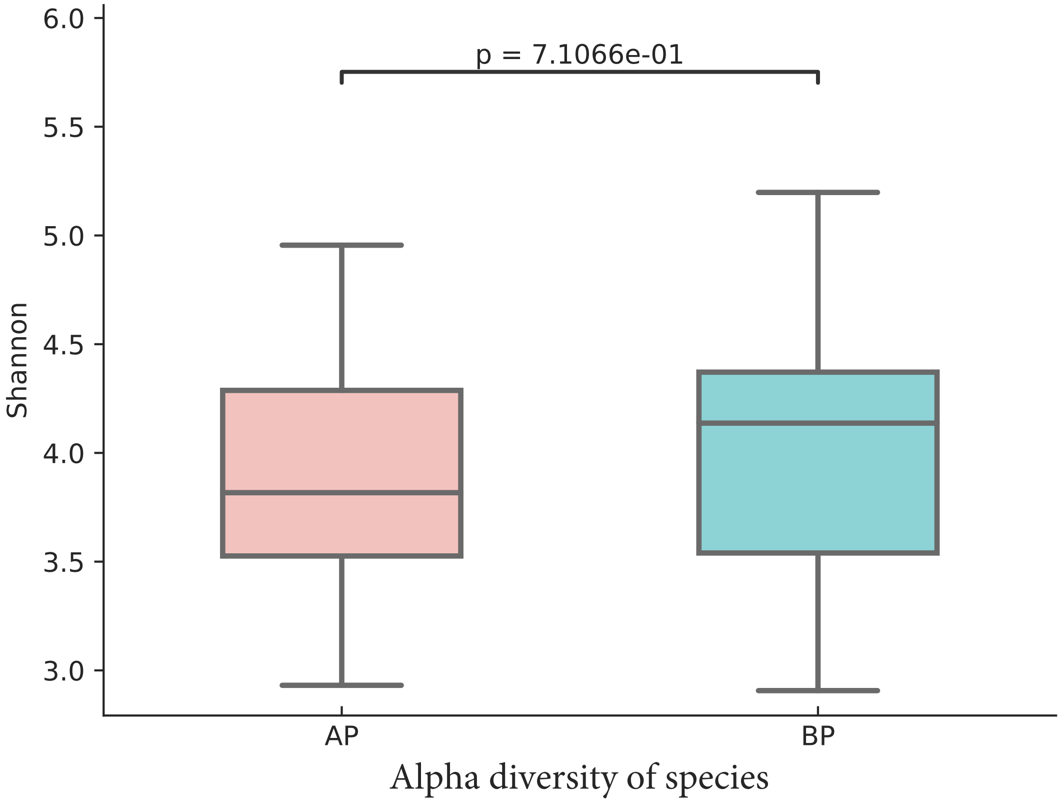
p = 7.1066e-01
Shannon
6.0
5.5
5.0
4.5
4.0
3.5
3.0
AP
BP
Alpha diversity of species

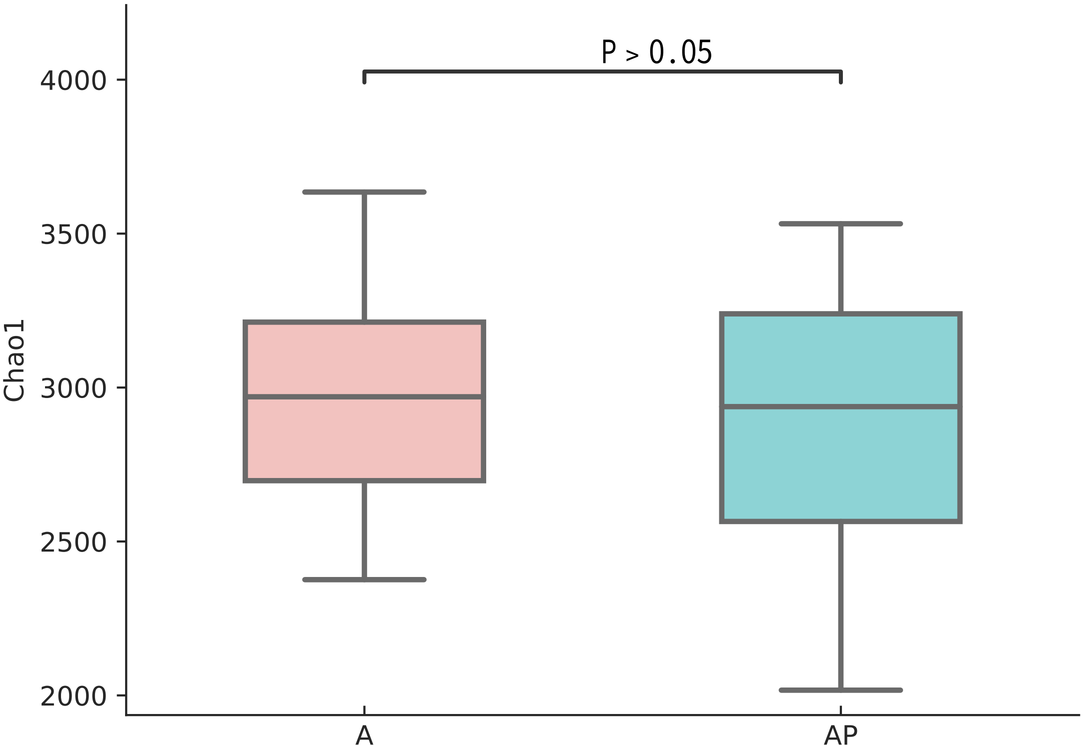

Alpha diversity of species
c

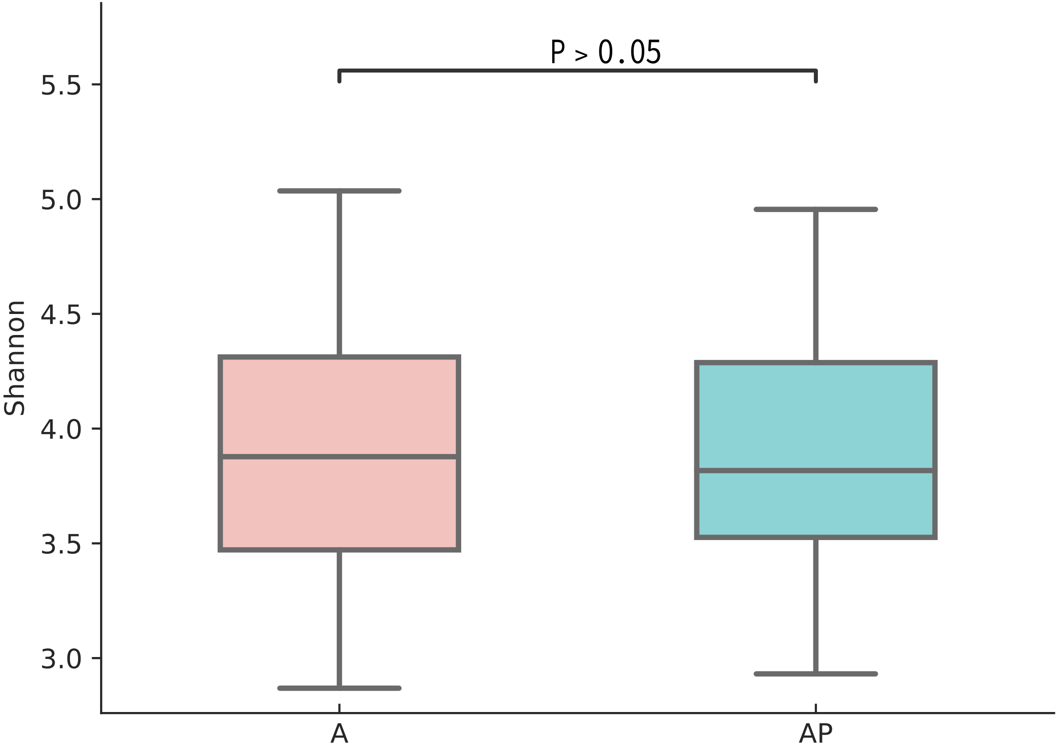

Alpha diversity of species

c

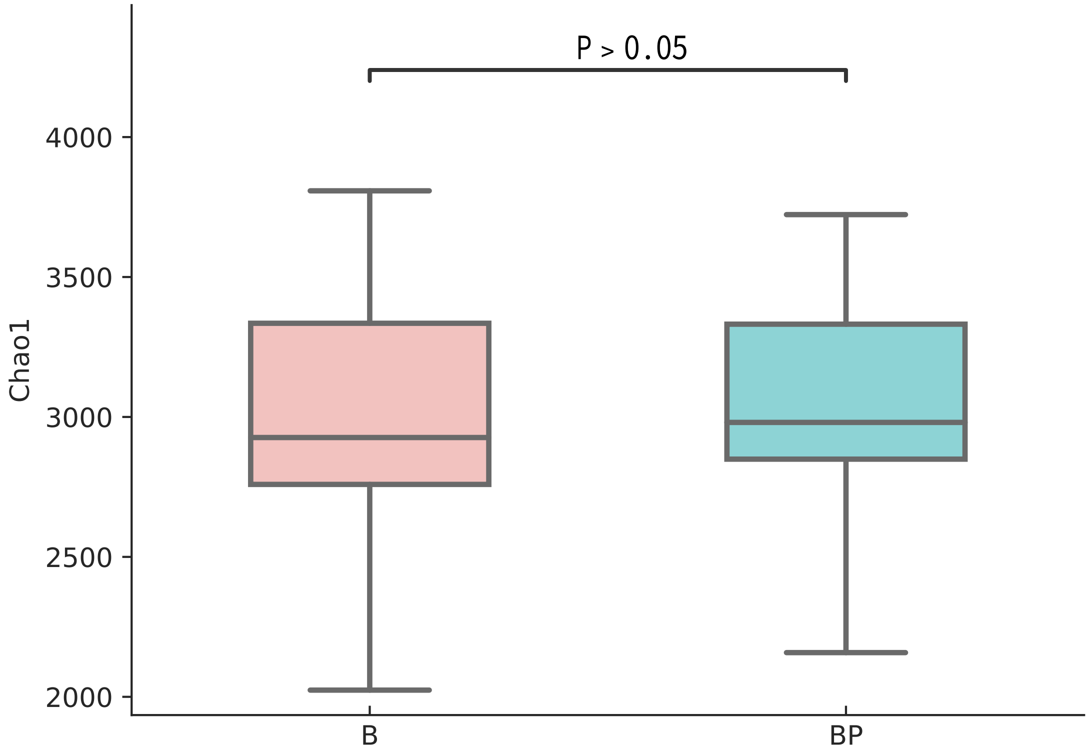

c

Alpha diversity of species

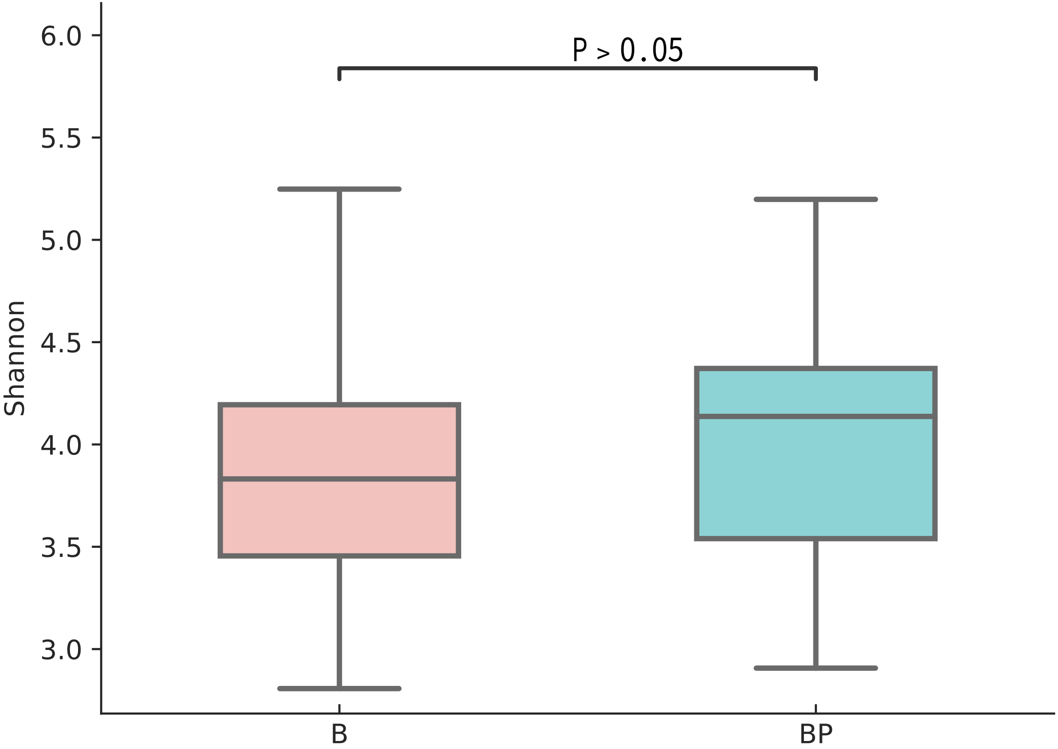

c

Alpha diversity of species

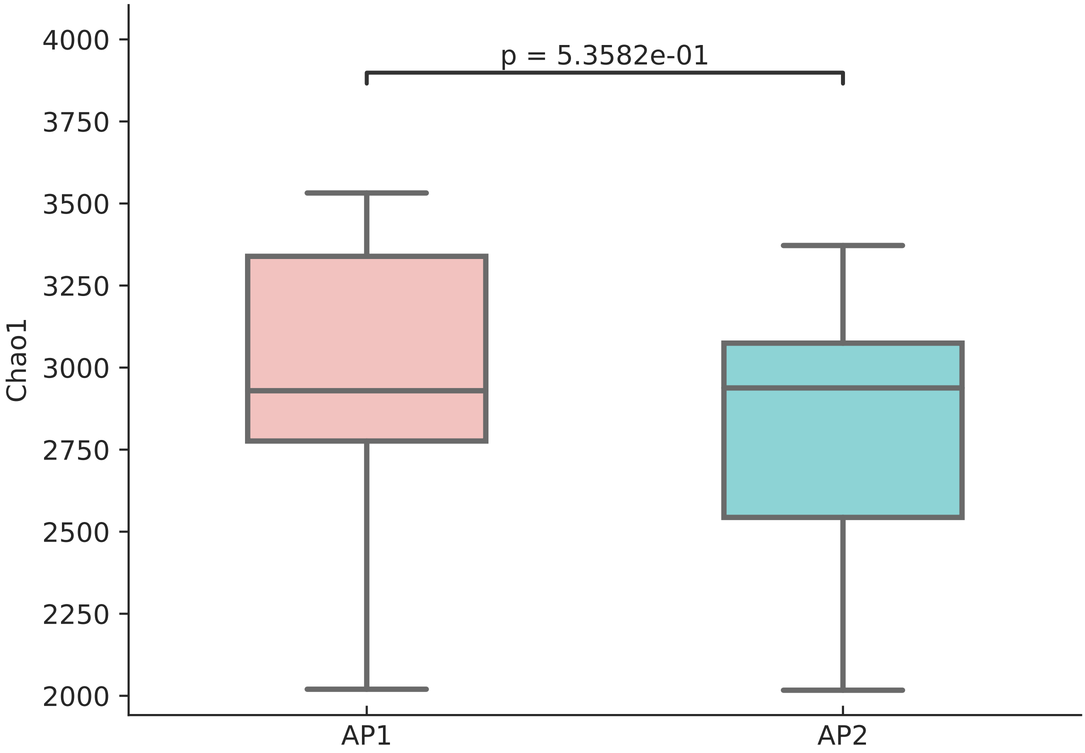

Alpha diversity of species

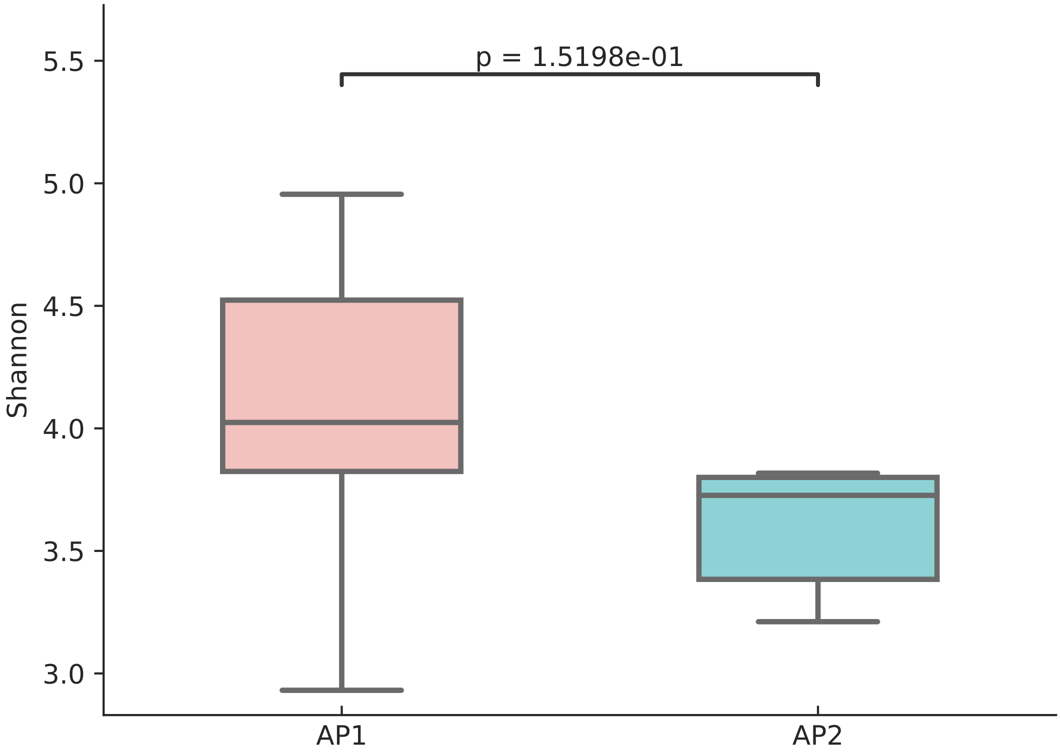

Alpha diversity of species

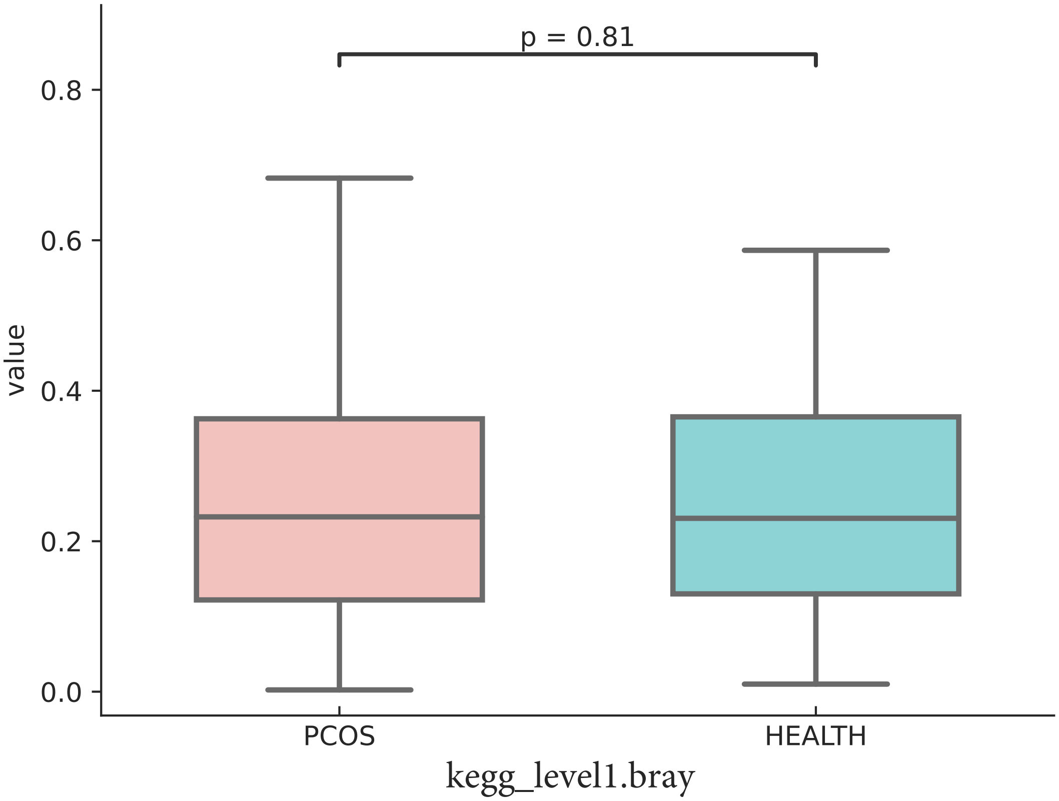
p = 0.81
0.8
0.6
0.4
0.2
0.0
value
PCOS
HEALTH
kegg_level1.bray

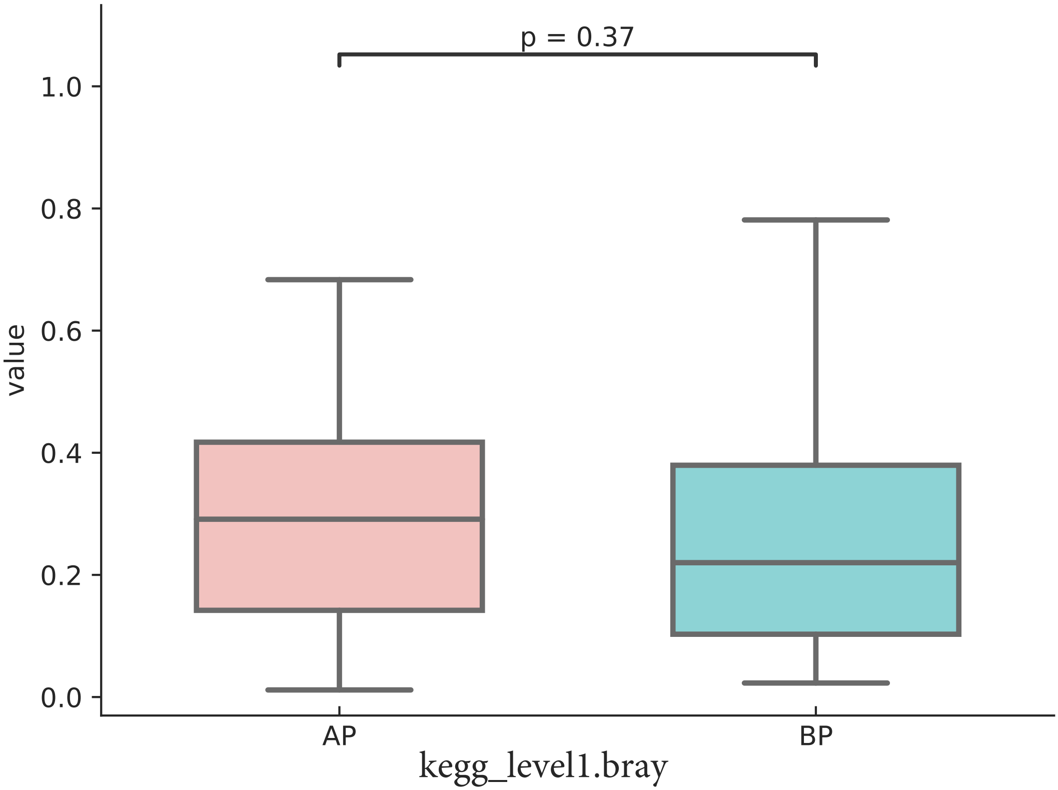
p = 0.37
1.0
0.8
0.6
0.4
0.2
0.0
value
AP
BP
kegg_level1.bray

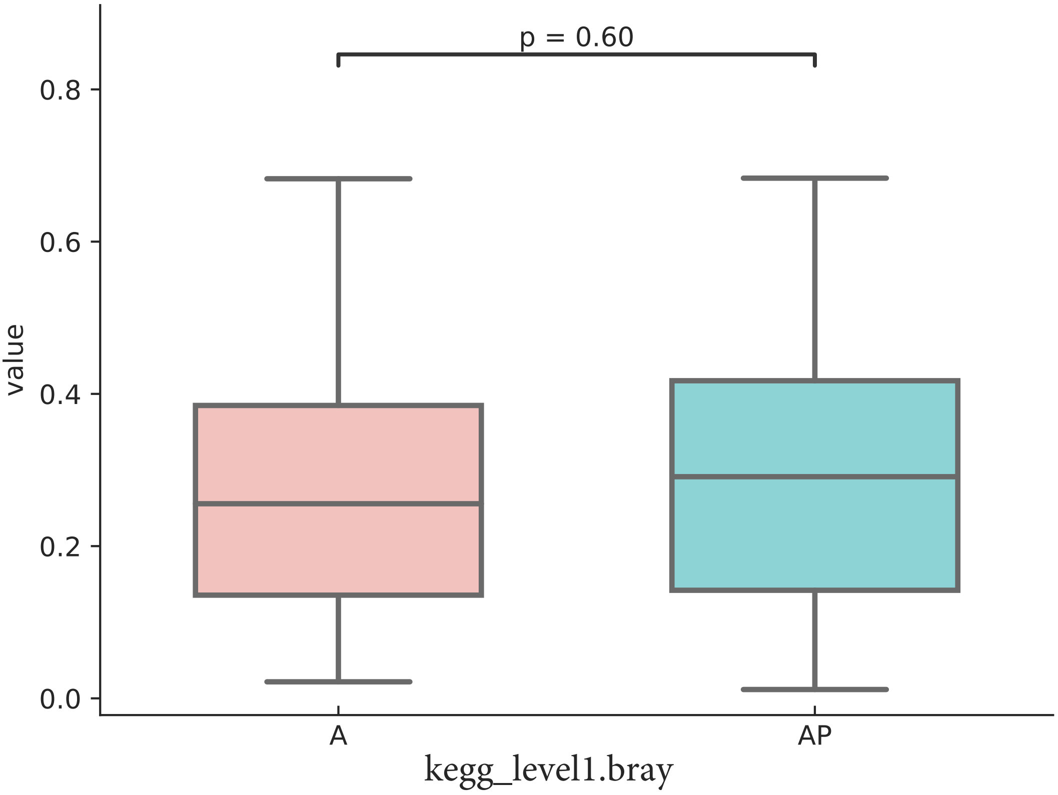

p = 0.60
0.8
0.6
0.4
0.2
0.0
value
A
AP
kegg_level1.bray

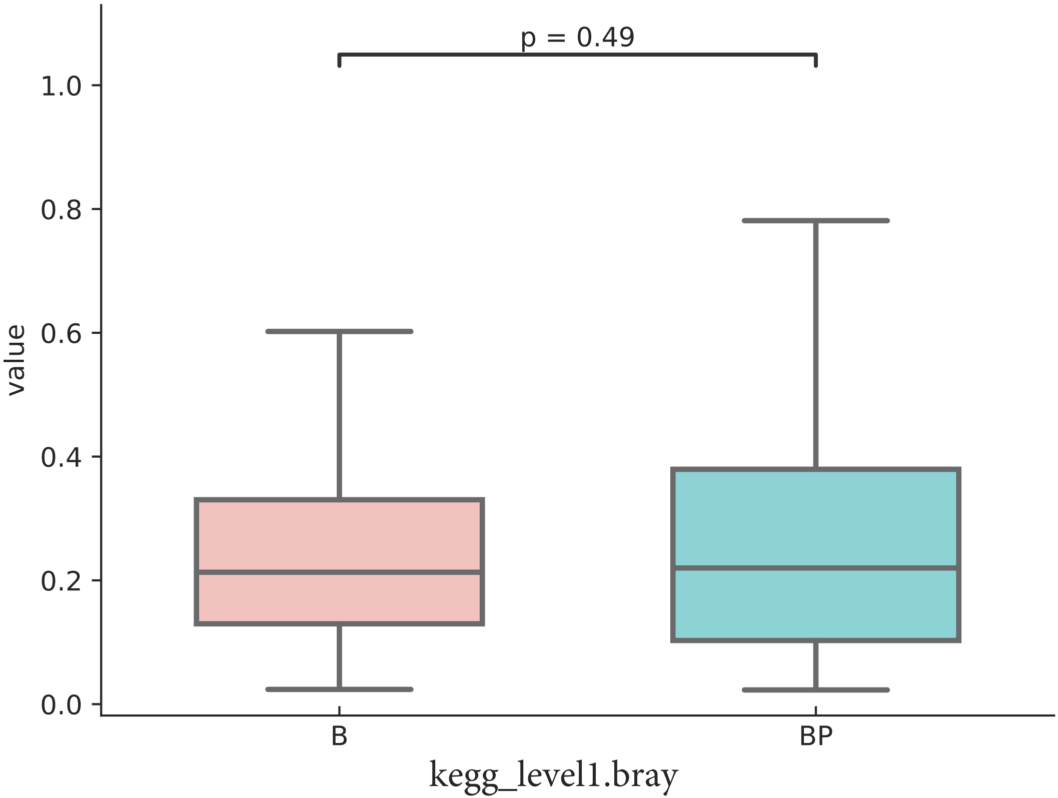

p = 0.49
1.0
0.8
0.6
0.4
0.2
0.0
value
B
BP
kegg_level1.bray

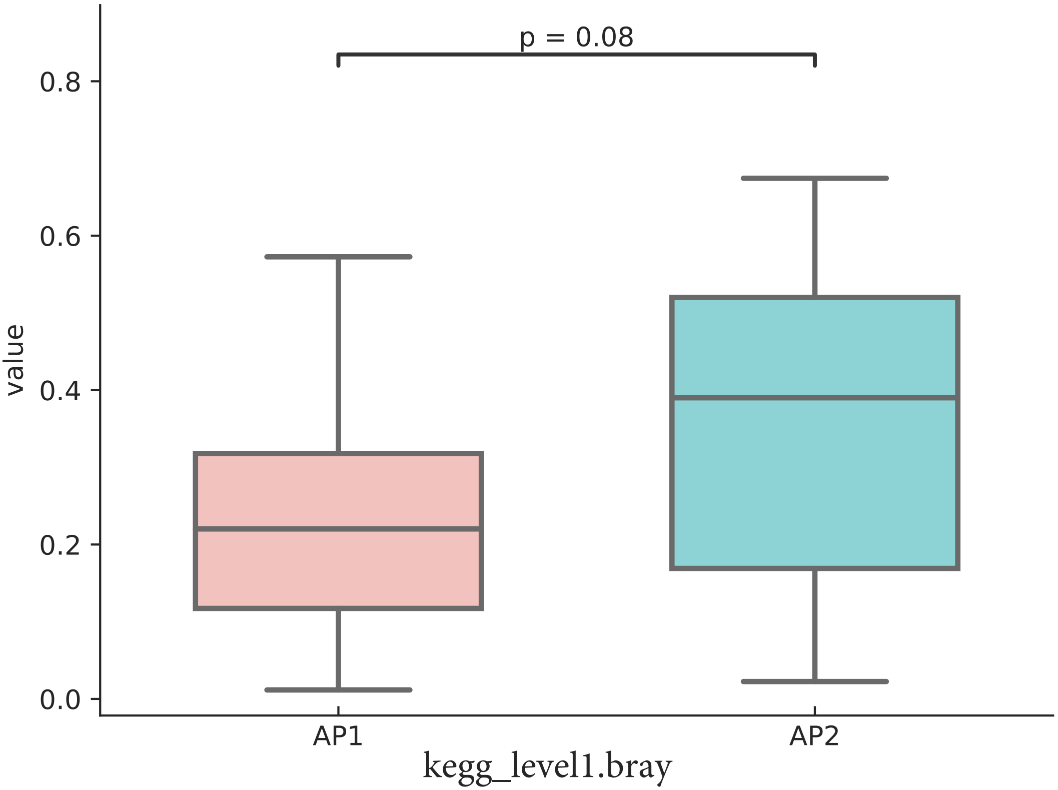
p = 0.08
0.8
0.6
0.4
0.2
0.0
value
AP1
AP2
kegg_level1.bray

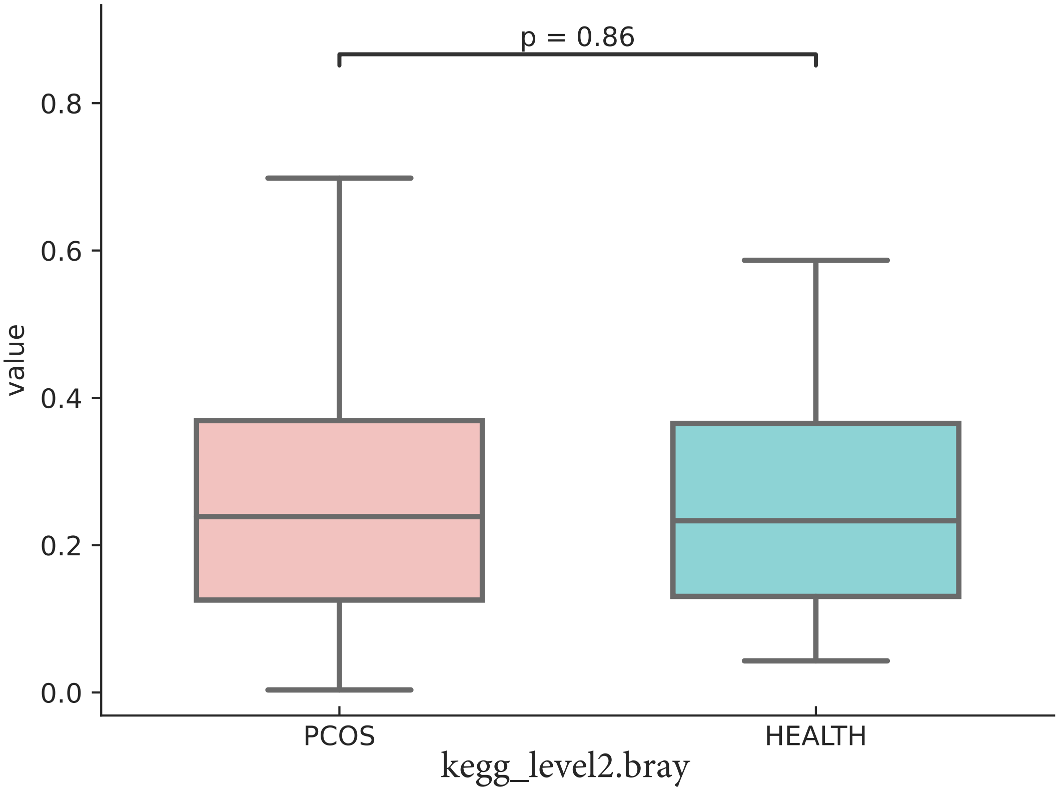
p = 0.86
0.8
0.6
0.4
0.2
0.0
value
PCOS
HEALTH
kegg_level2.bray

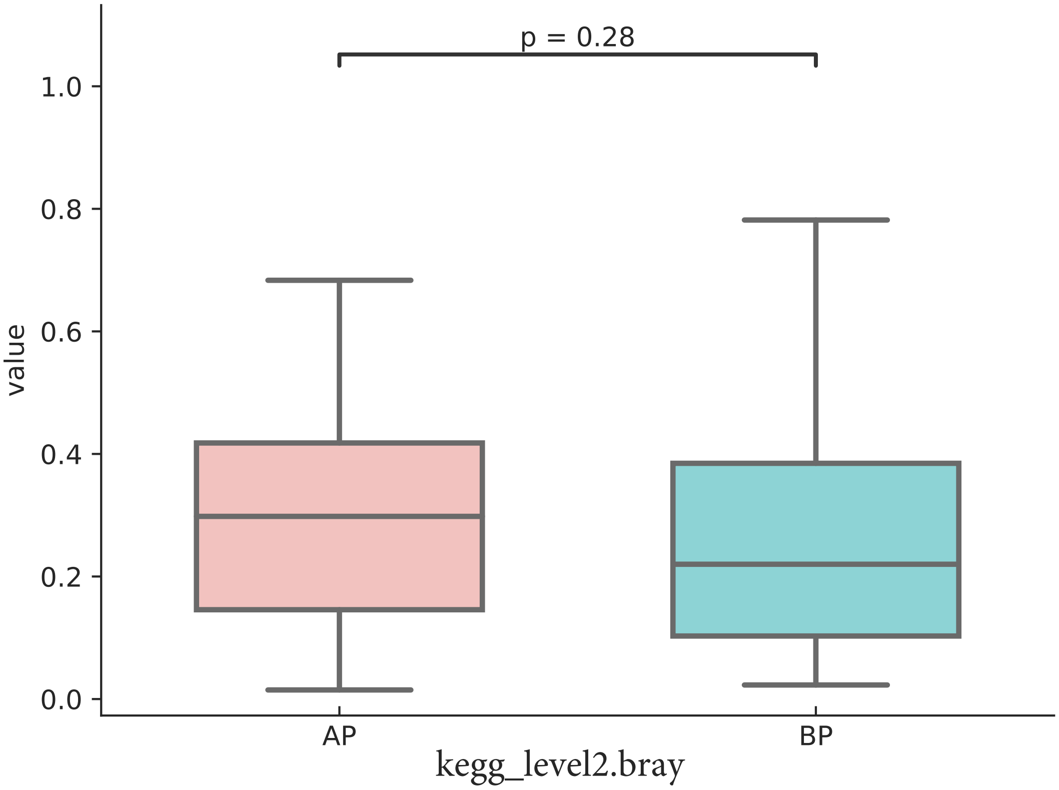

p = 0.28
1.0
0.8
0.6
0.4
0.2
0.0
value
AP
BP
kegg_level2.bray

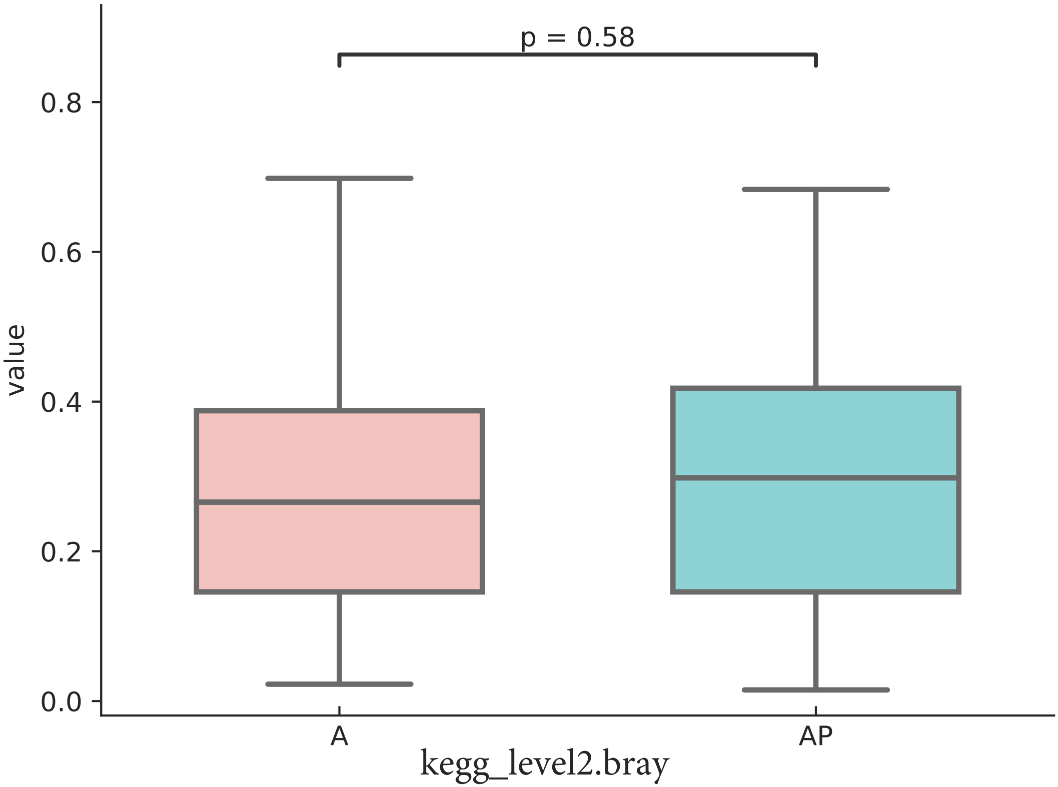
p = 0.58
value
0.0
0.2
0.4
0.6
0.8
A
AP
kegg_level2.bray

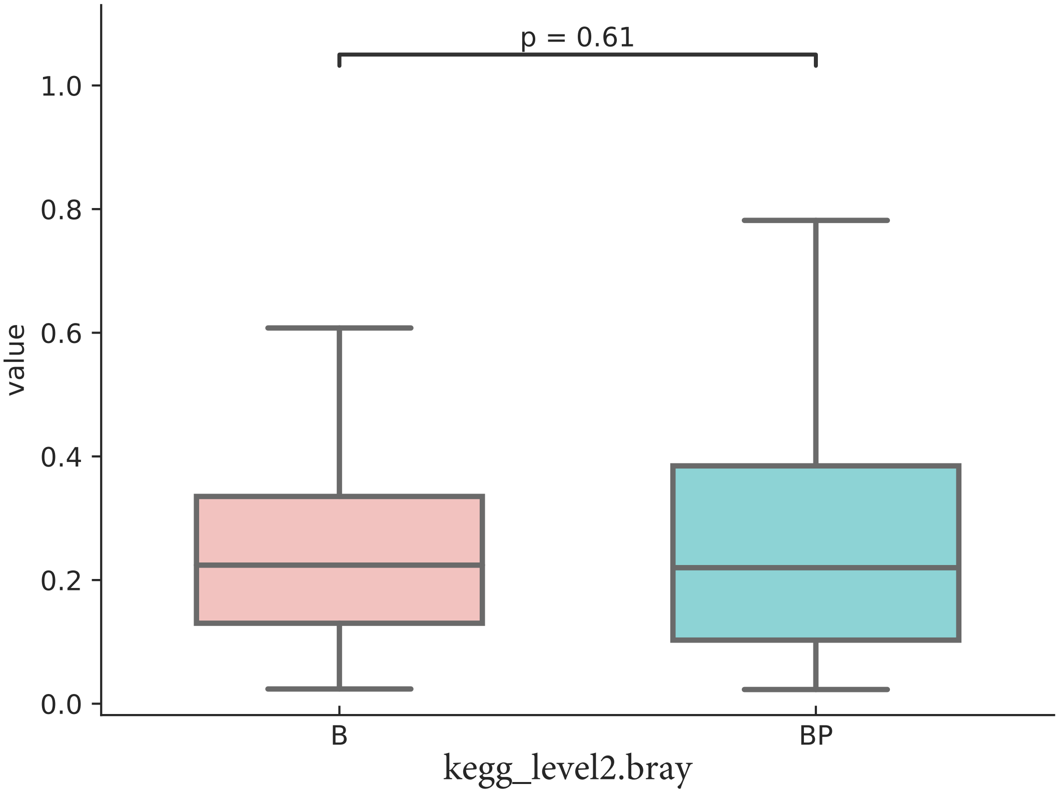

p = 0.61
1.0
0.8
0.6
0.4
0.2
0.0
value
B
BP
kegg_level2.bray

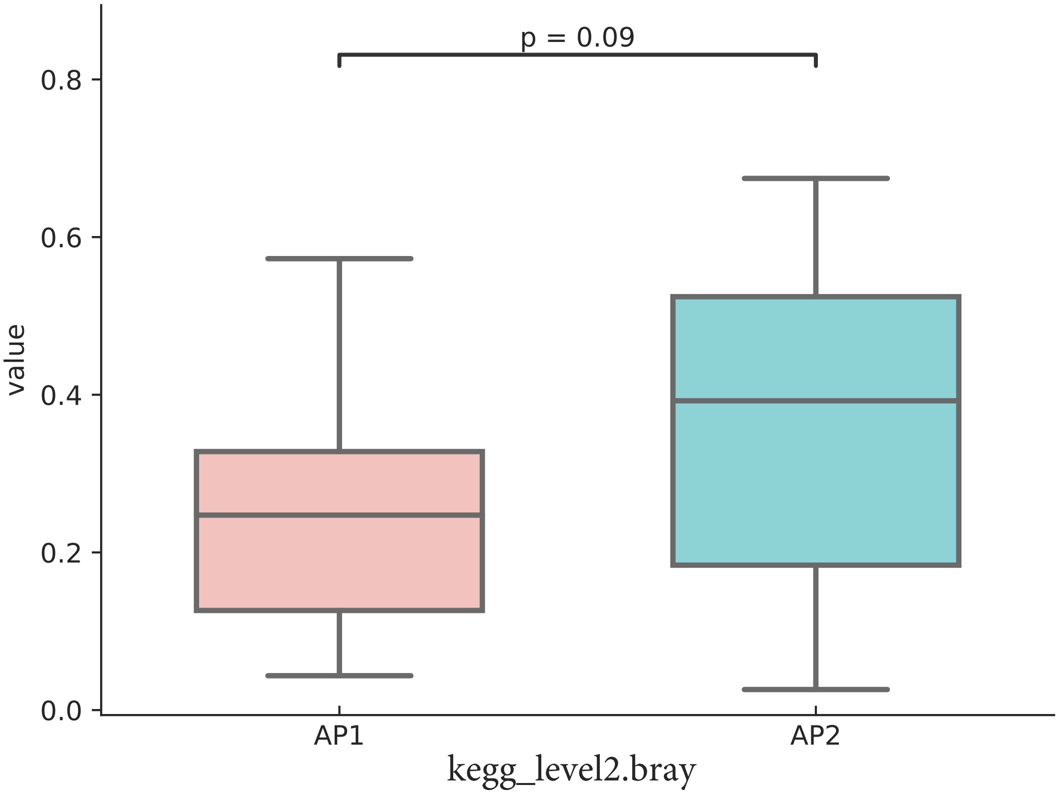
p = 0.09
0.8
0.6
0.4
0.2
0.0
value
AP1
AP2
kegg_level2.bray

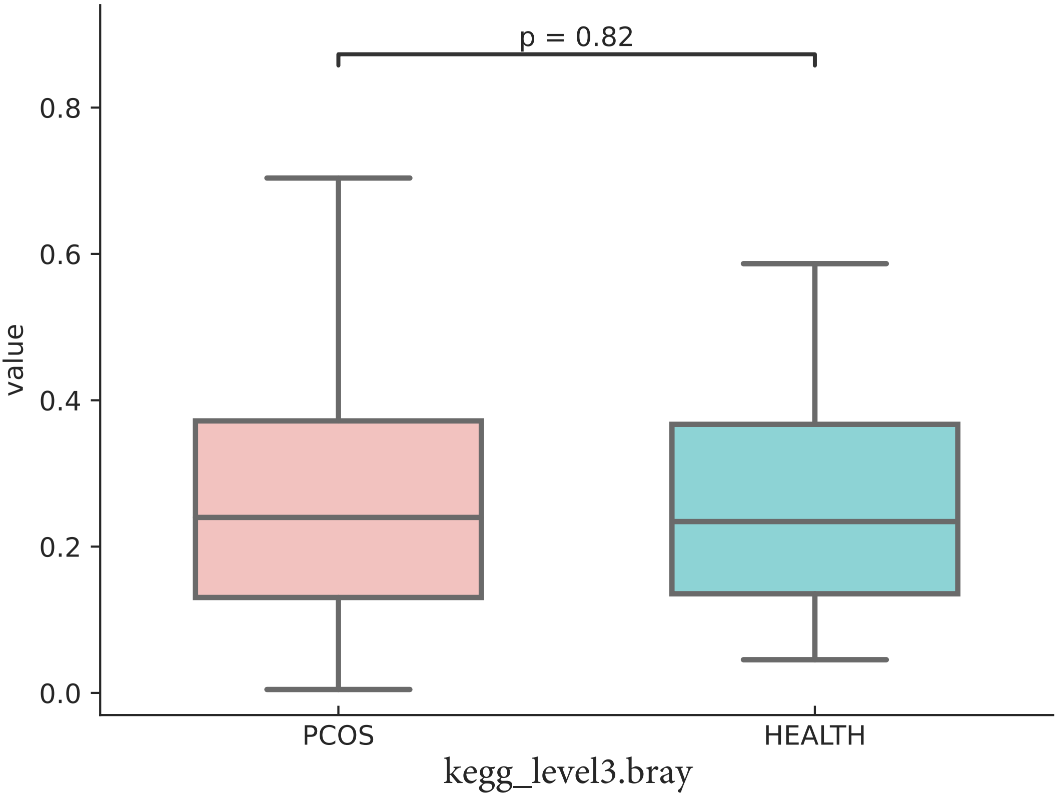
p = 0.82
0.8
0.6
0.4
0.2
0.0
value
PCOS
HEALTH
kegg_level3.bray

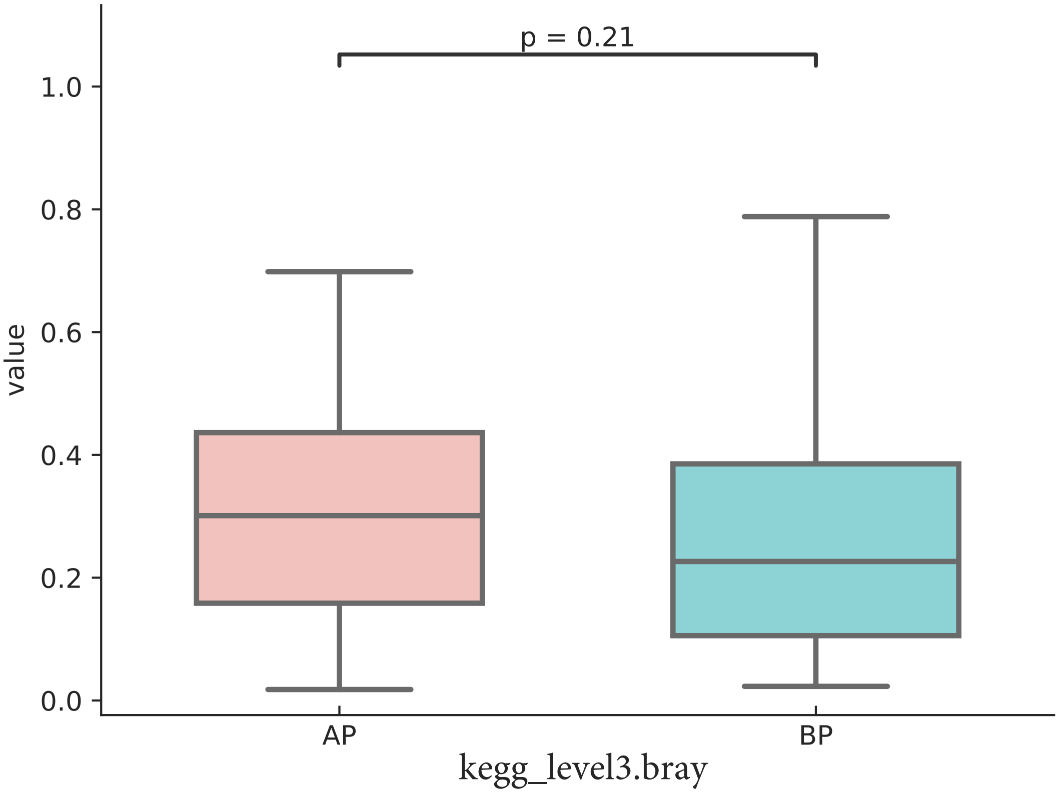
p = 0.21
1.0
0.8
0.6
0.4
0.2
0.0
value
AP
BP
kegg_level3.bray

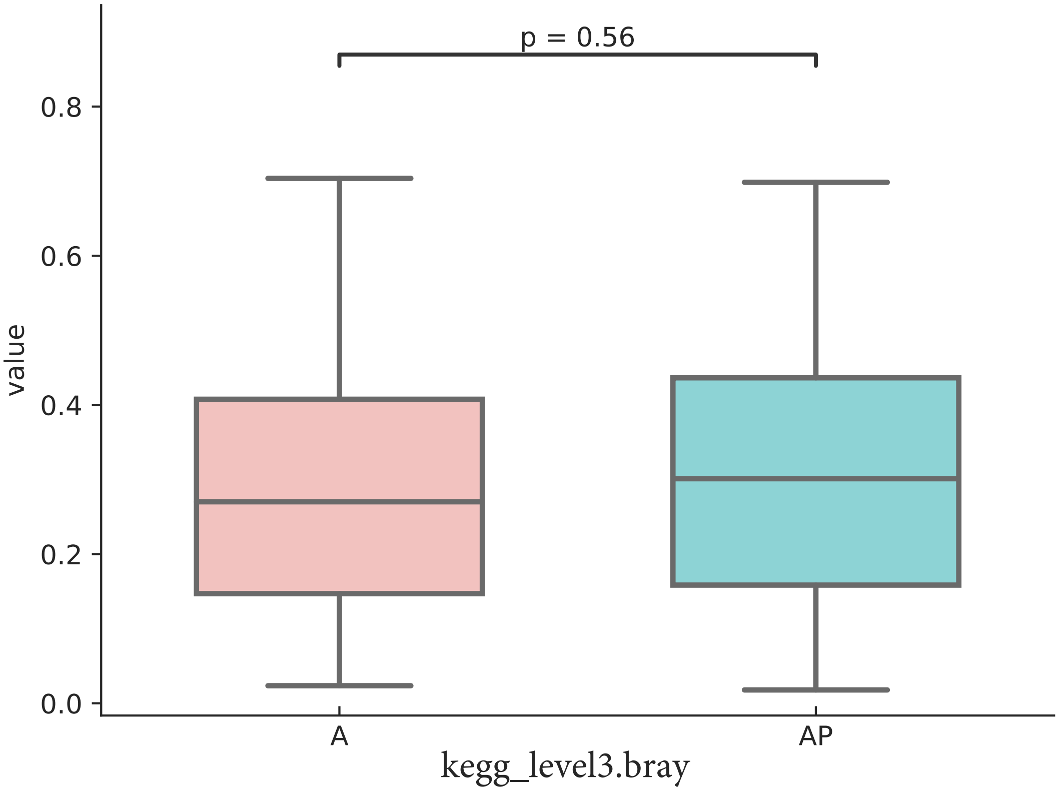
p = 0.56
0.8
0.6
0.4
0.2
0.0
value
A
AP
kegg_level3.bray

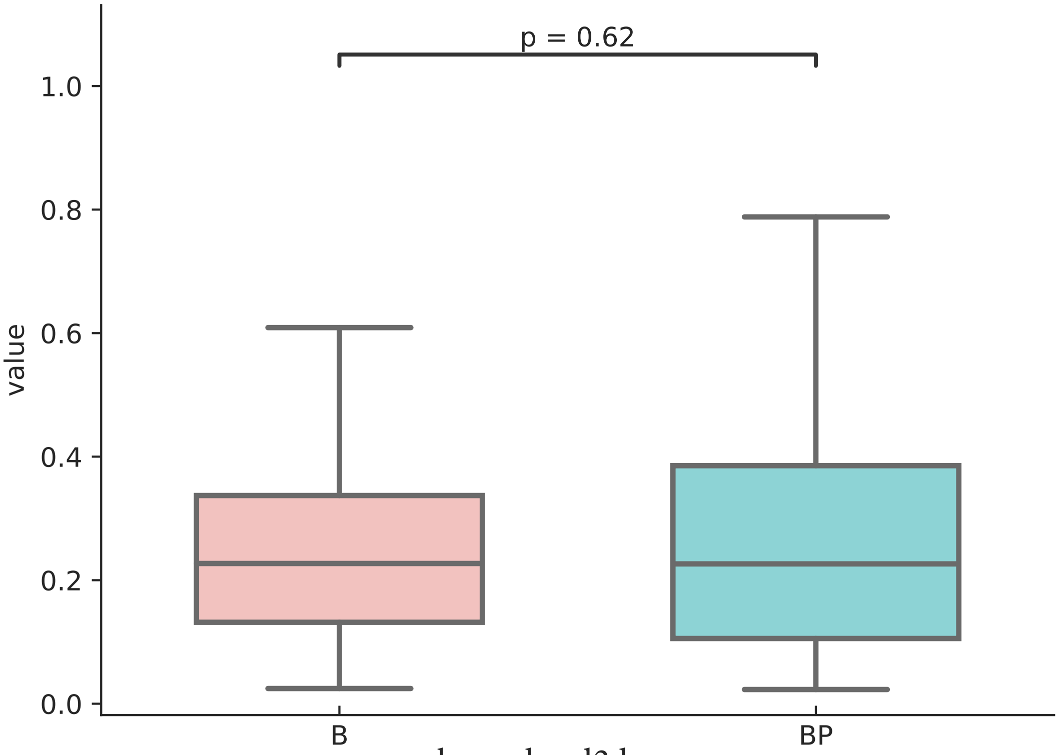

kegg_level3.bray

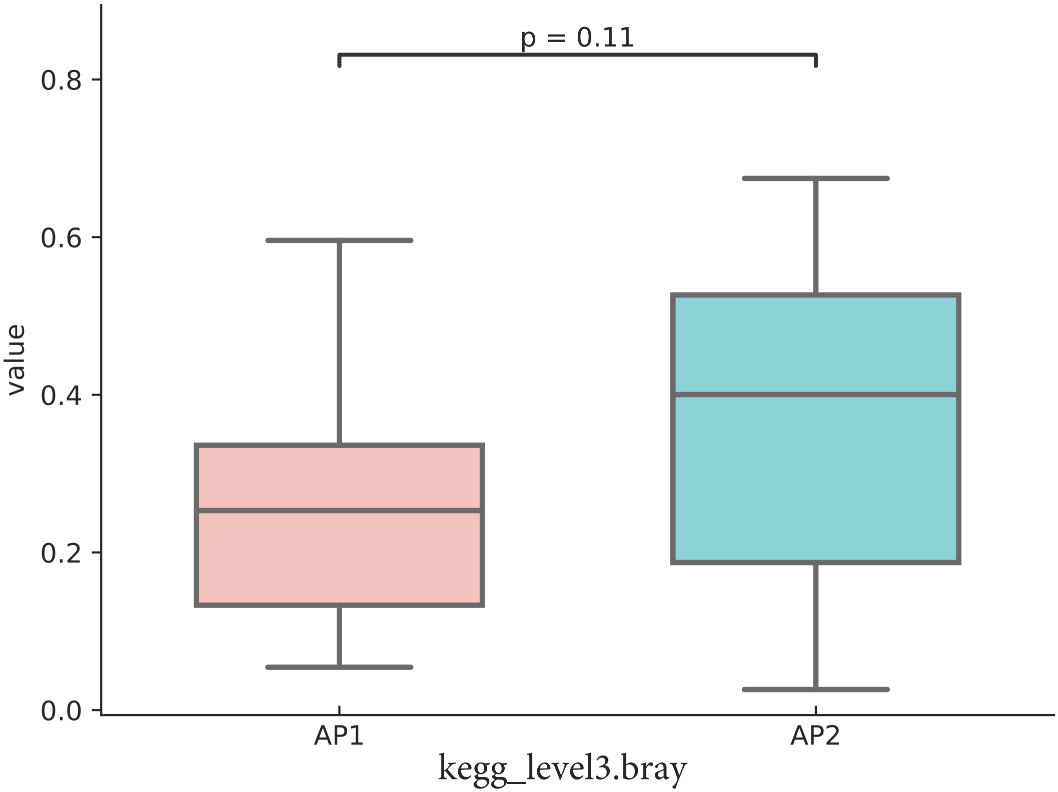
p = 0.11
0.8
0.6
0.4
0.2
0.0
value
AP1
AP2
kegg_level3.bray

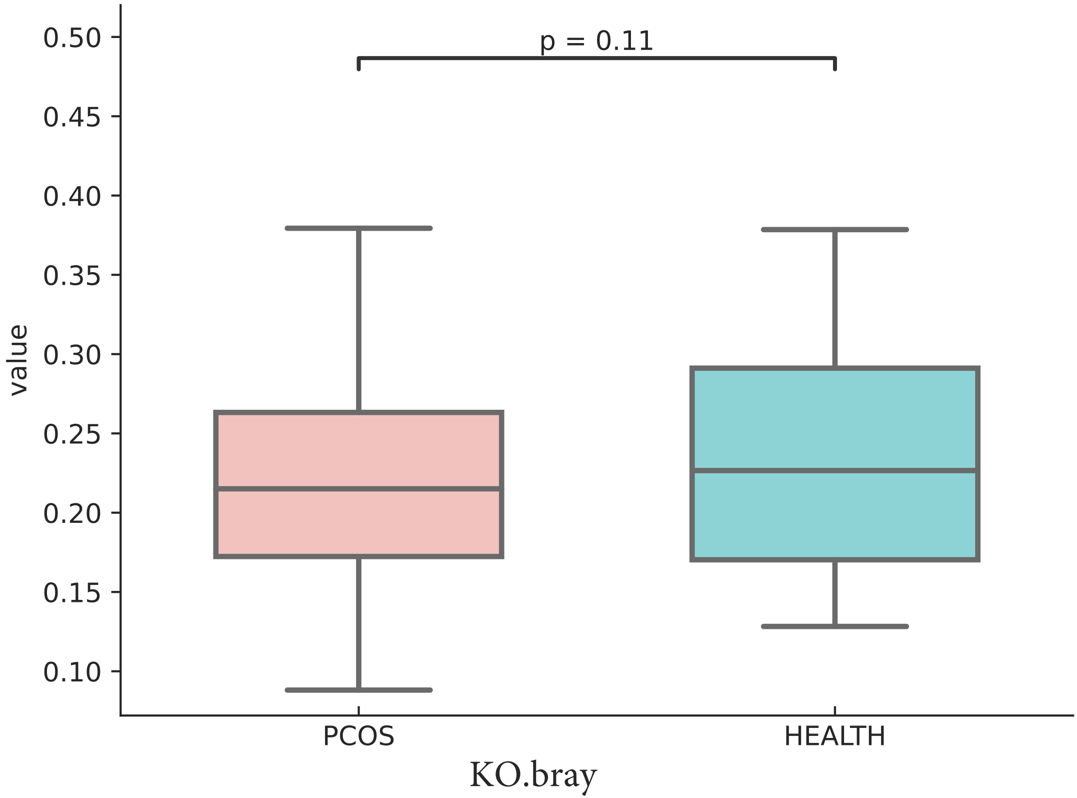

p = 0.11
0.50
0.45
0.40
0.35
0.30
0.25
0.20
0.15
0.10
value
PCOS
HEALTH
KO.bray

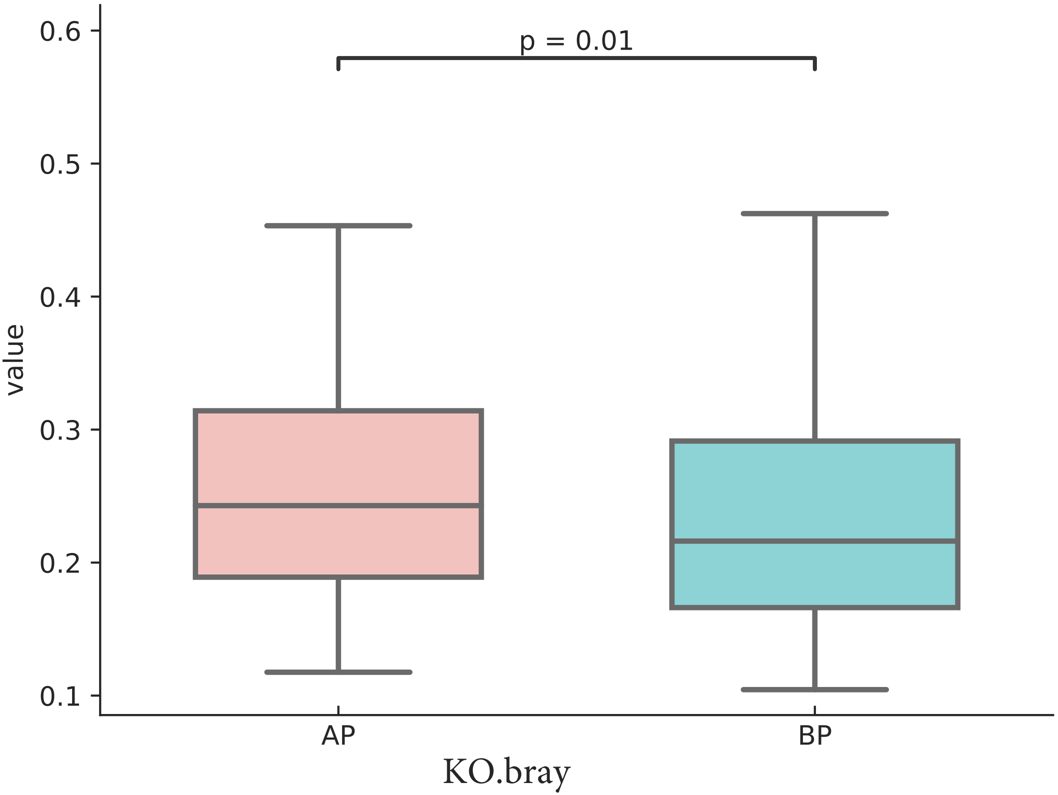

p = 0.01
0.6
0.5
0.4
0.3
0.2
0.1
value
AP
BP
KO.bray

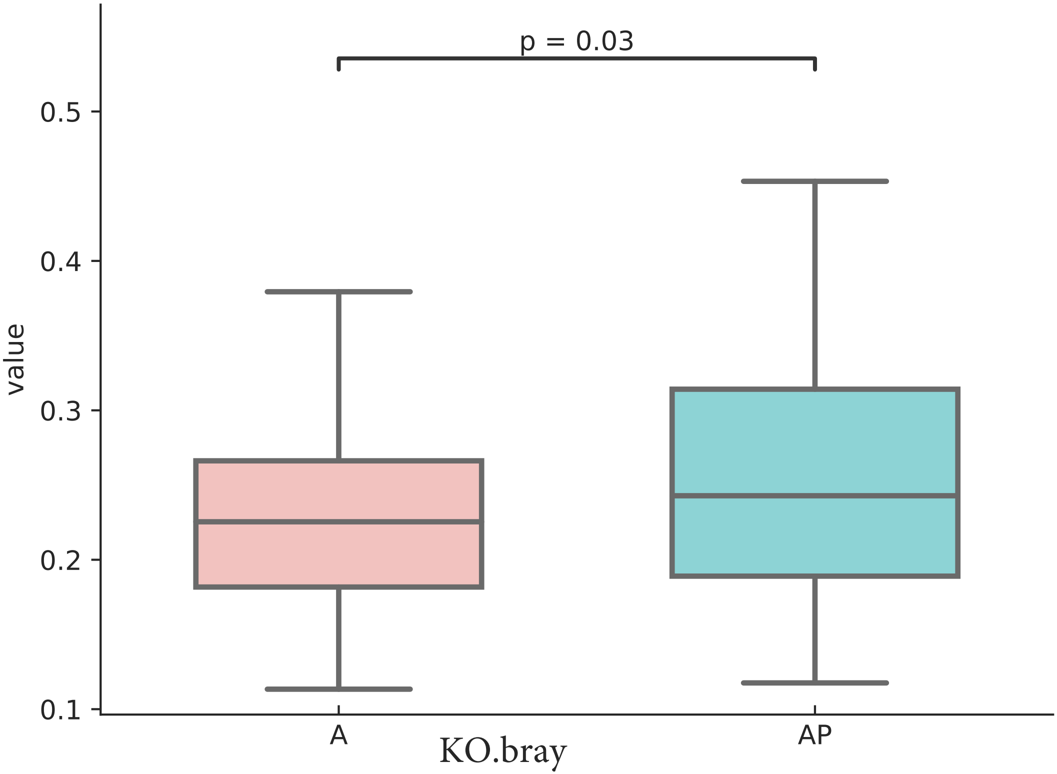

KO.bray

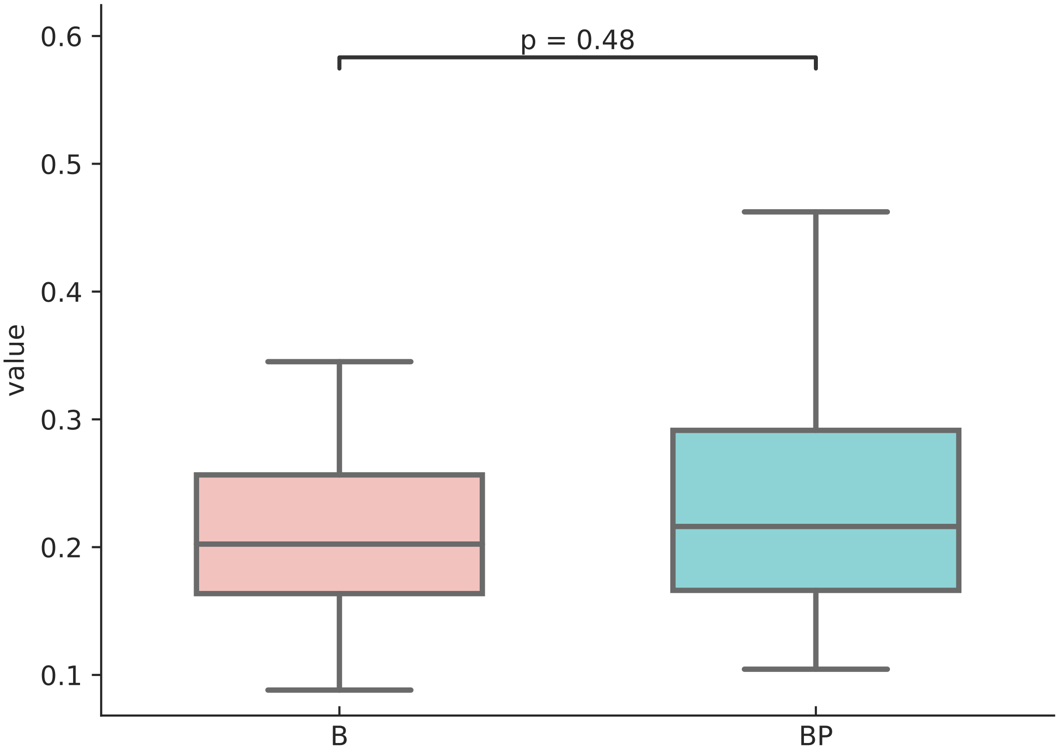

KO.bray

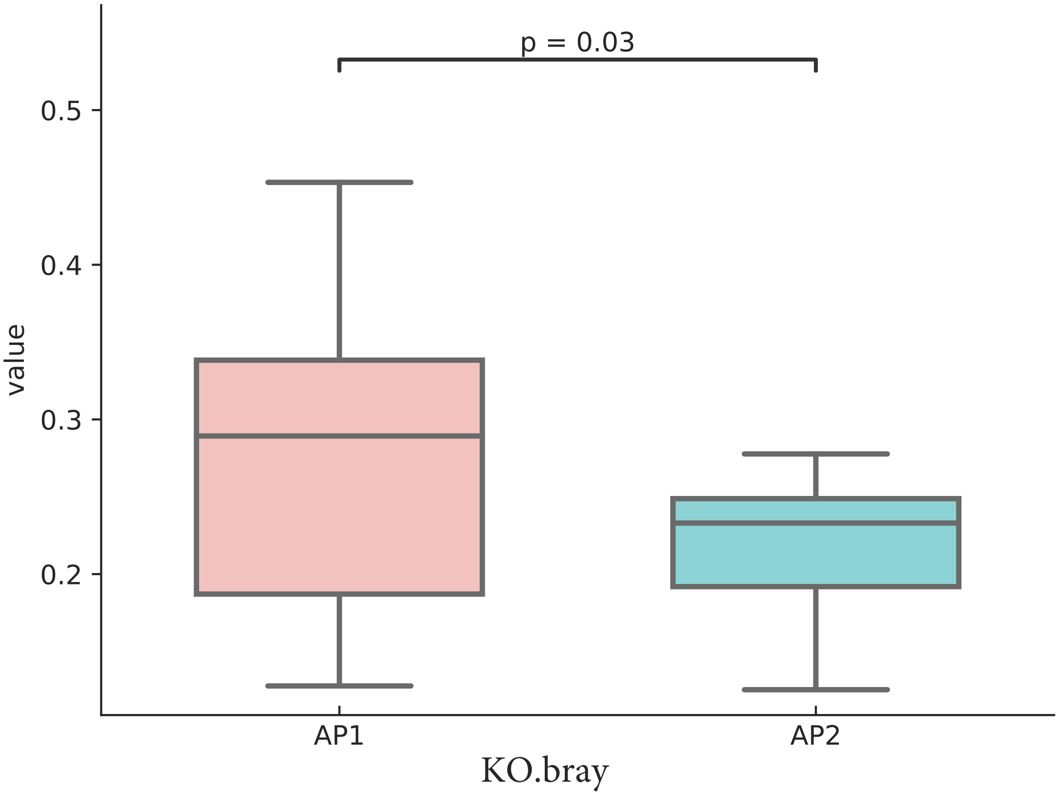
p = 0.03
0.5
0.4
0.3
0.2
value
AP1
AP2
KO.bray

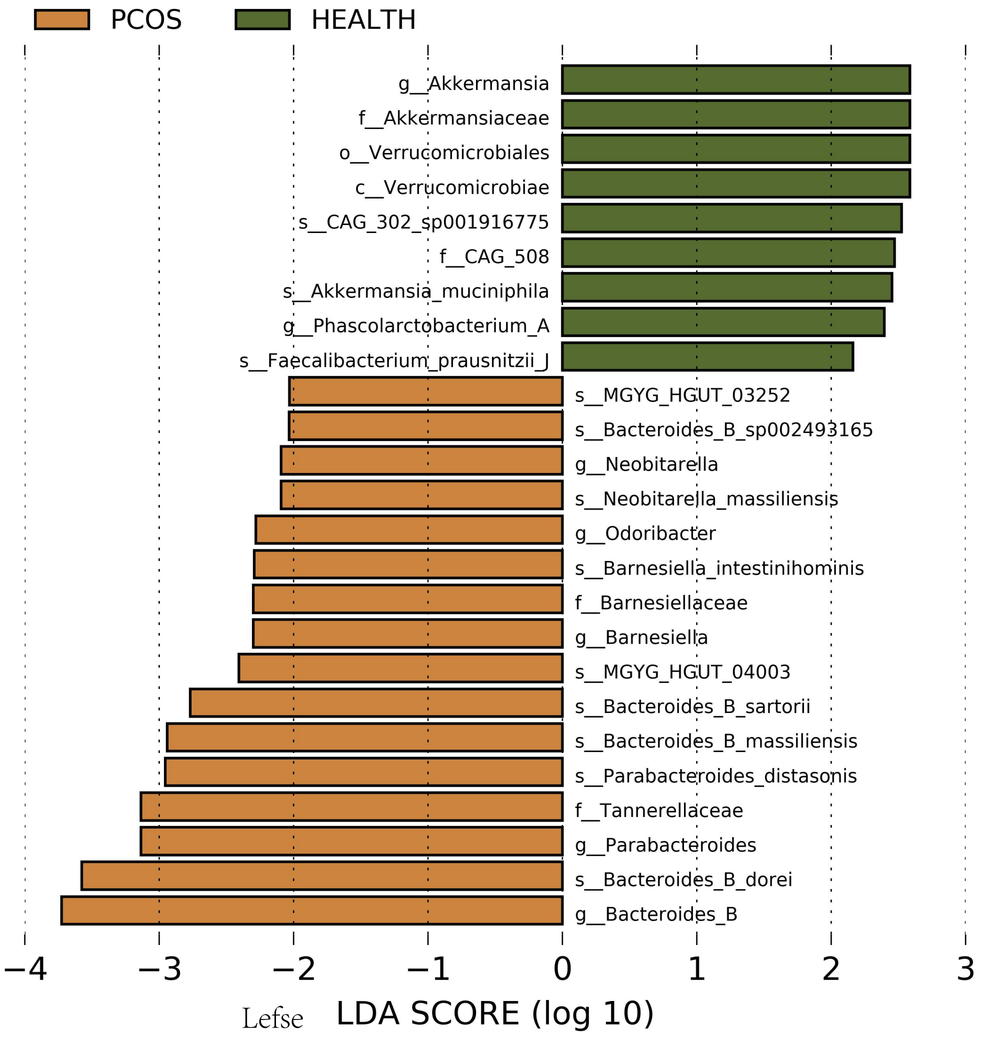
PCOS
HEALTH
g__Akkermansia
f__Akkermansiaceae
o__Verrucomicrobiales
c__Verrucomicrobiae
s__CAG_302_sp001916775
f__CAG_508
s__Akkermansia_muciniphila
g__Phascolarctobacterium_A
s__Faecalibacterium_prausnitzii_J
s__MGYG_HGUT_03252
s__Bacteroides_B_sp002493165
g__Neobitarella
s__Neobitarella_massiliensis
g__Odoribacter
s__Barnesiella_intestinihominis
f__Barnesiellaceae
g__Barnesiella
s__MGYG_HGUT_04003
s__Bacteroides_B_sartorii
s__Bacteroides_B_massiliensis
s__Parabacteroides_distasonis
f__Tannerellaceae
g__Parabacteroides
s__Bacteroides_B_dorei
g__Bacteroides_B
−4
−3
−2
−1
0
1
2
3
Lefse
LDA SCORE (log 10)

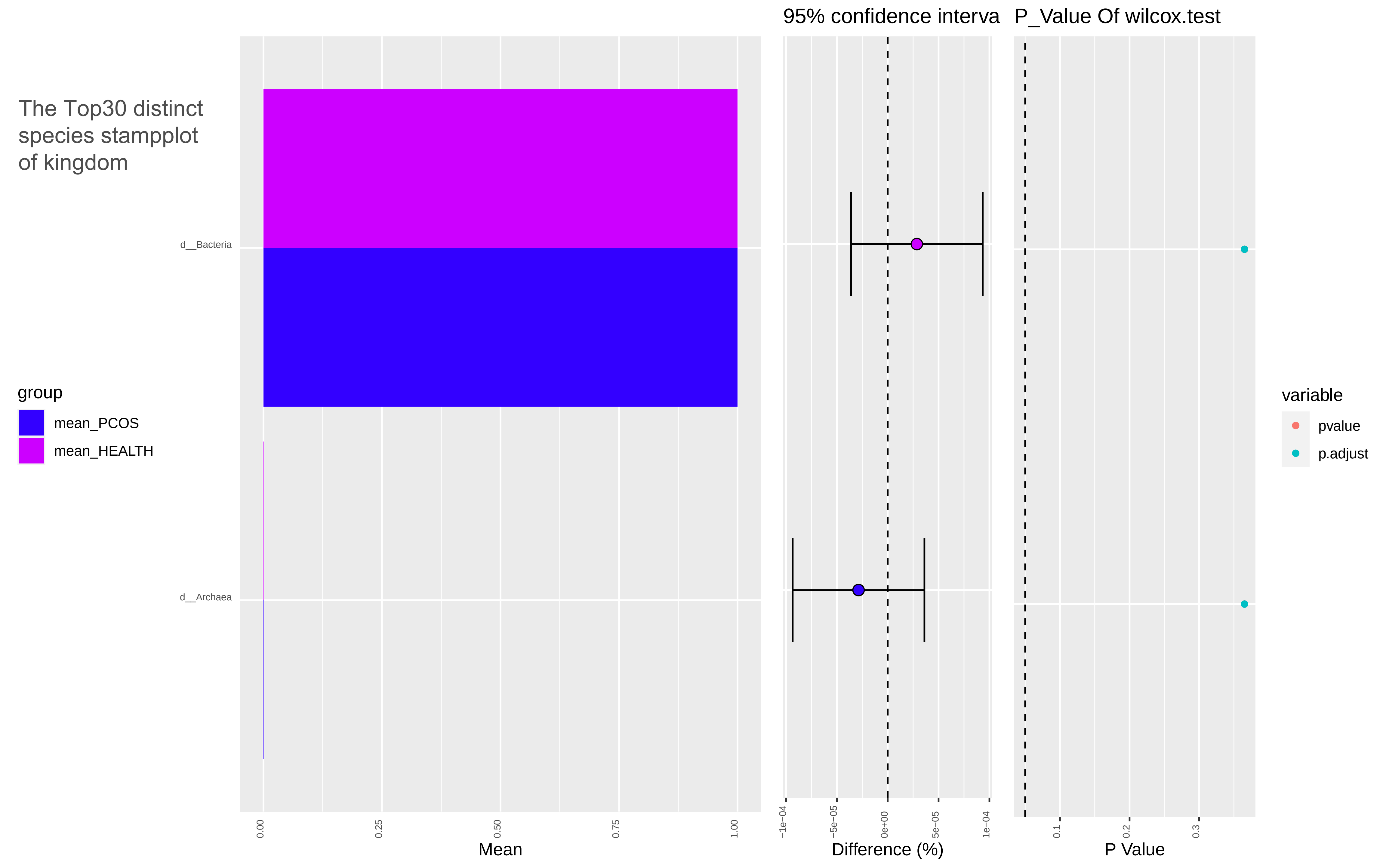

The Top30 distinct species stampplot of kingdom
95% confidence interva
P_Value Of wilcox.test
d__Bacteria
d__Archaea
group
mean_PCOS
mean_HEALTH
variable
pvalue
p.adjust
0.00
0.25
0.50
0.75
1.00
Mean
−1e−04
−5e−05
0e+00
5e−05
1e−04
Difference (%)
0.1
0.2
0.3
P Value

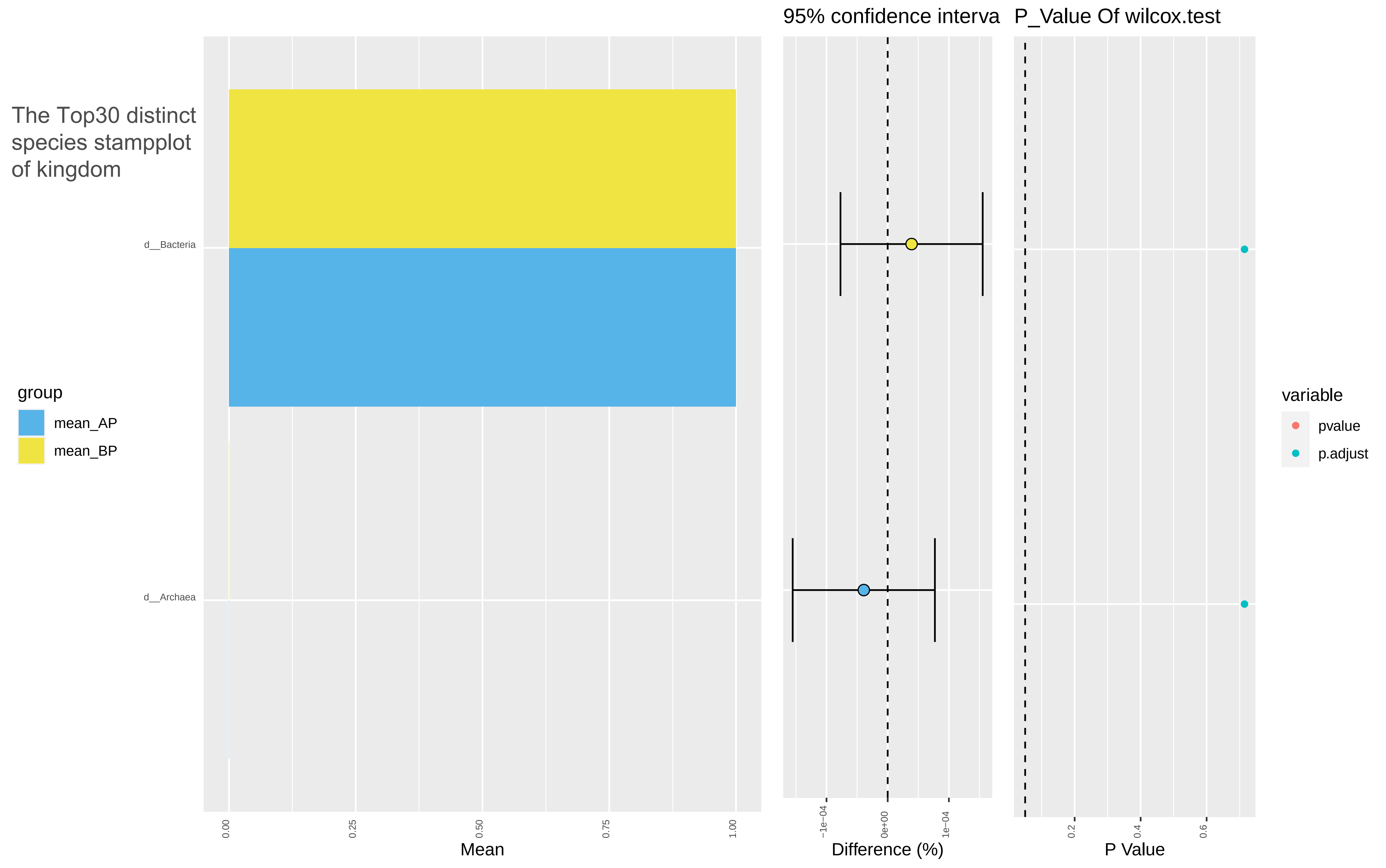

The Top30 distinct species stamppplot of kingdom
d__Bacteria
d__Archaea
group
mean_AP
mean_BP
0.00
0.25
0.50
0.75
1.00
Mean
95% confidence interva
−1e−04
0e+00
1e−04
Difference (%)
P_Value Of wilcox.test
0.2
0.4
0.6
P Value
variable
pvalue
p.adjust

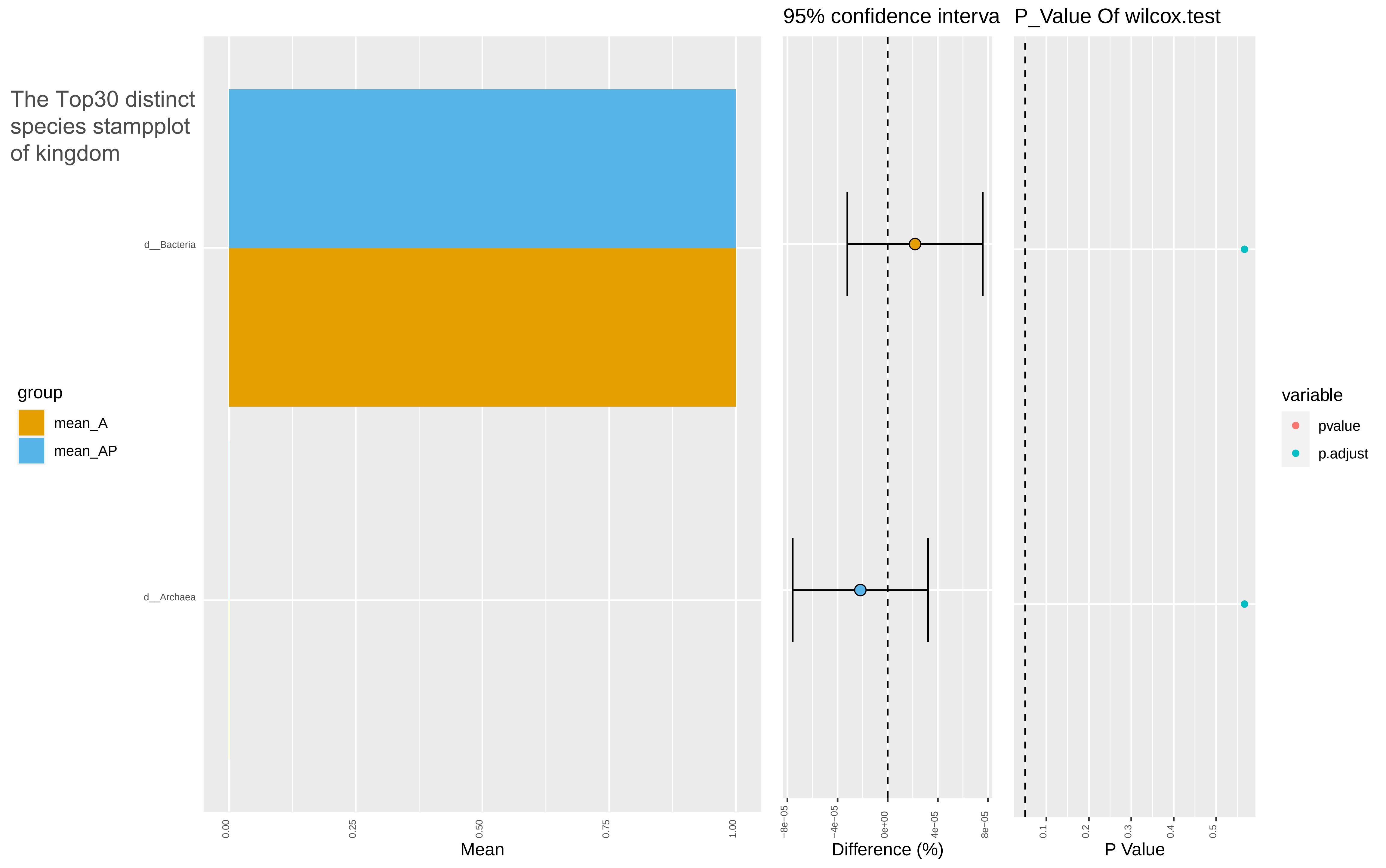

The Top30 distinct species stampplot of kingdom
95% confidence interva
P_Value Of wilcox.test
d__Bacteria
d__Archaea
group
mean_A
mean_AP
variable
pvalue
p.adjust
0.00
0.25
0.50
0.75
1.00
Mean
−8e−05
−4e−05
0e+00
4e−05
8e−05
Difference (%)
0.1
0.2
0.3
0.4
0.5
P Value

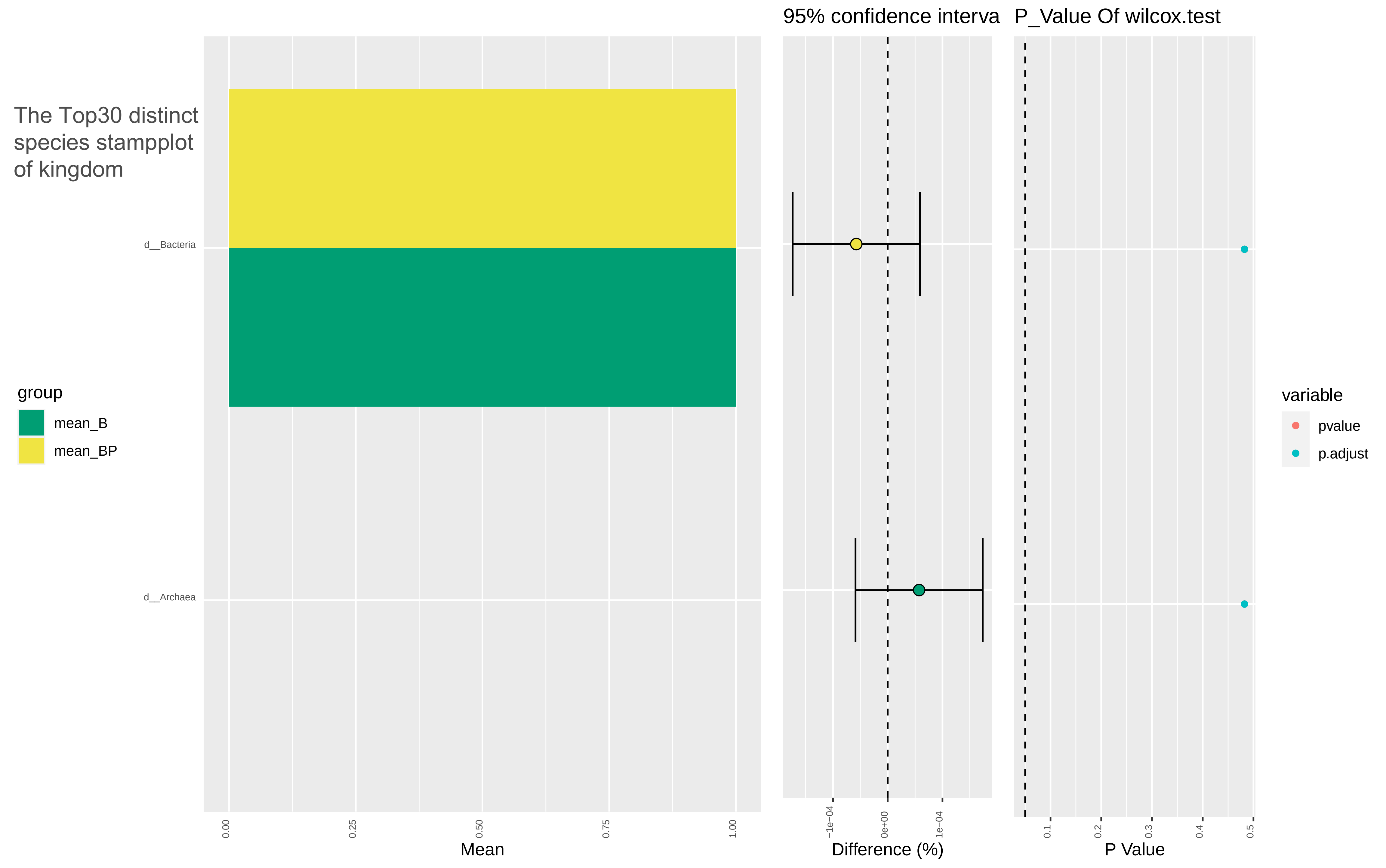

The Top30 distinct species stampplot of kingdom
95% confidence interva
P_Value Of wilcox.test
d__Bacteria
d__Archaea
group
mean_B
mean_BP
variable
pvalue
p.adjust
0.00
0.25
0.50
0.75
1.00
Mean
−1e−04
0e+00
1e−04
Difference (%)
0.1
0.2
0.3
0.4
0.5
P Value

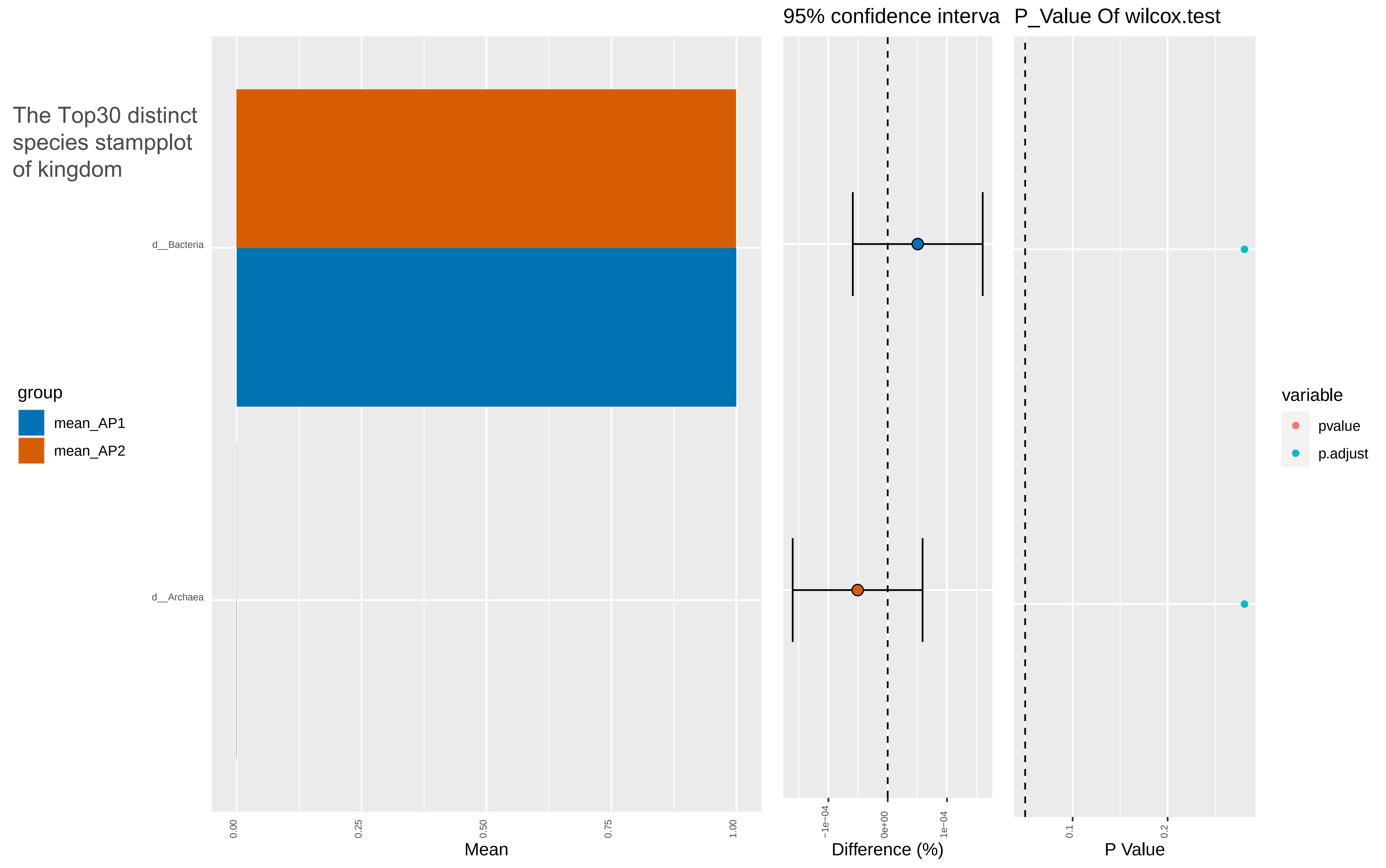

95% confidence interva
P_Value Of wilcox.test
The Top30 distinct species stampplot of kingdom
d__Bacteria
d__Archaea
group
mean_AP1
mean_AP2
variable
pvalue
p.adjust
0.00
0.25
0.50
0.75
1.00
Mean
−1e−04
0e+00
1e−04
Difference (%)
0.1
0.2
P Value

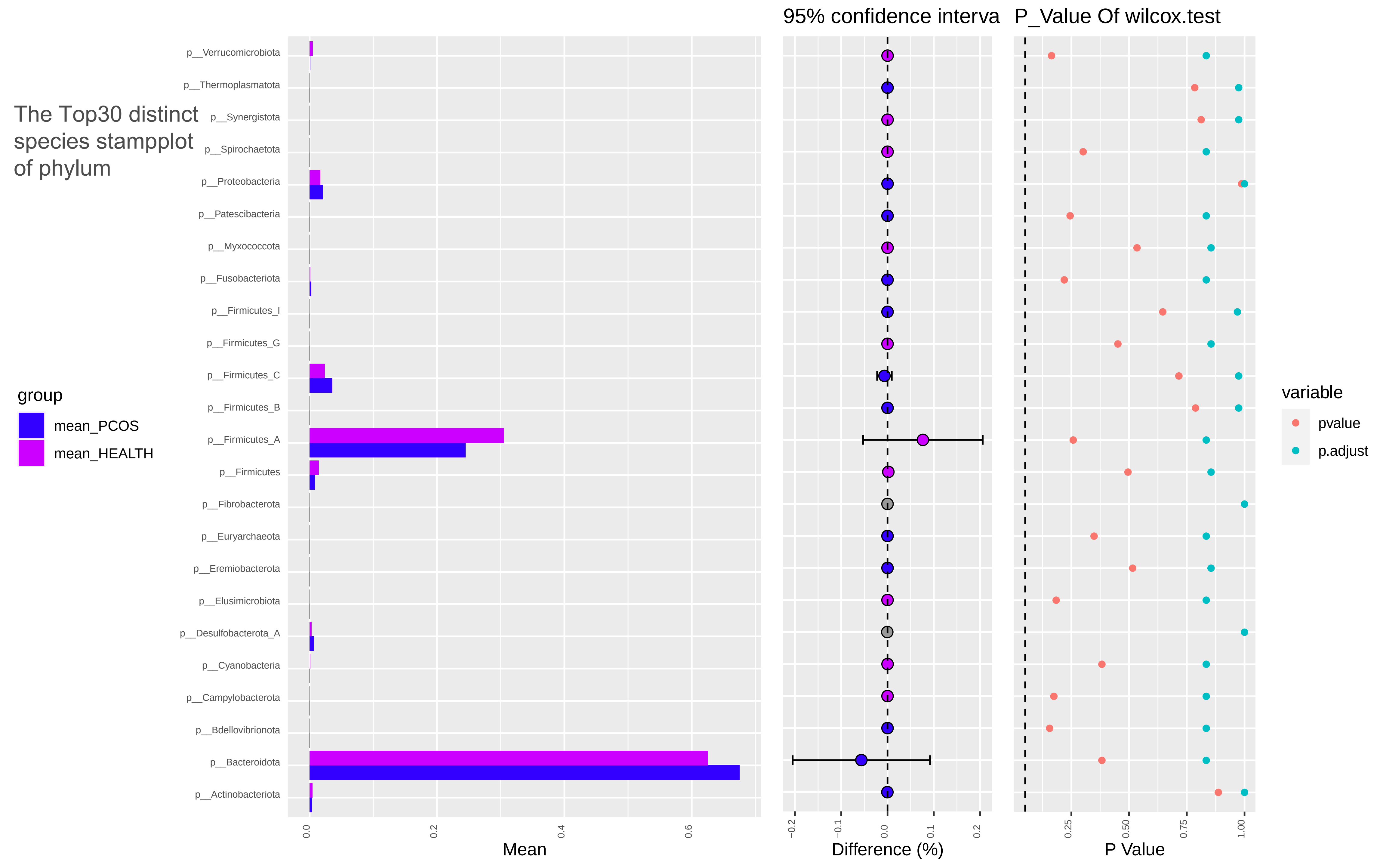
The Top30 distinct species stampplot of phylum
95% confidence interva
P_Value Of wilcox.test
p__Verrucomicrobiota
p__Thermoplasmatota
p__Synergistota
p__Spirochaetota
p__Proteobacteria
p__Patescibacteria
p__Myxococcota
p__Fusobacteriota
p__Firmicutes_I
p__Firmicutes_G
p__Firmicutes_C
p__Firmicutes_B
p__Firmicutes_A
p__Firmicutes
p__Fibrobacterota
p__Euryarchaeota
p__Eremiobacterota
p__Elusimicrobiota
p__Desulfobacterota_A
p__Cyanobacteria
p__Campylobacterota
p__Bdellovibrionota
p__Bacteroidota
p__Actinobacteriota
group
mean_PCOS
mean_HEALTH
0.0
0.2
0.4
0.6
Mean
-0.2
-0.1
0.0
0.1
0.2
Difference (%)
0.25
0.50
0.75
1.00
P Value
variable
pvalue
p.adjust

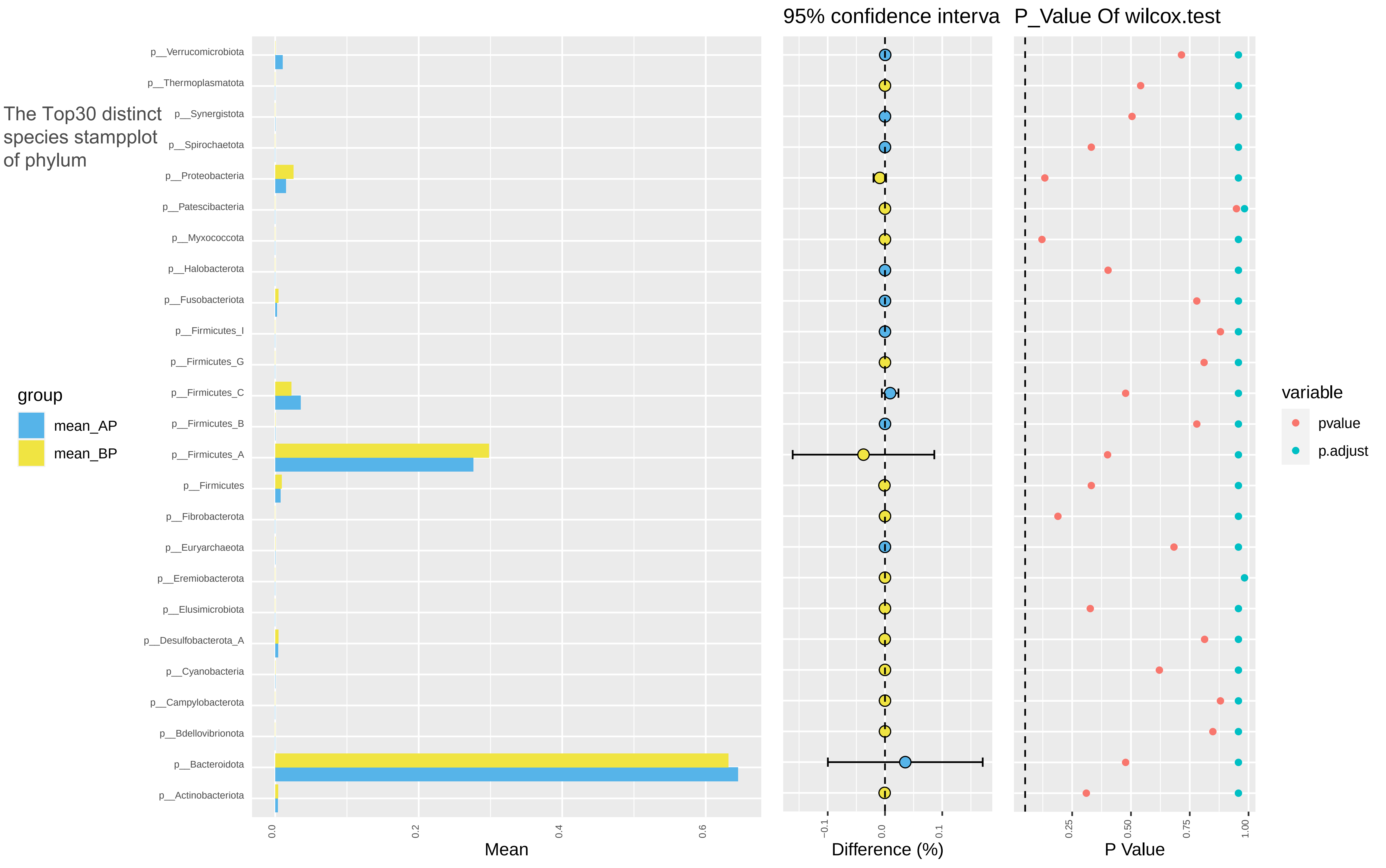

The Top30 distinct species stampplot of phylum
95% confidence interva
P_Value Of wilcox.test
p__Verrucomicrobiota
p__Thermoplasmatota
p__Synergistota
p__Spirochaetota
p__Proteobacteria
p__Patescibacteria
p__Myxococcota
p__Halobacterota
p__Fusobacteriota
p__Firmicutes_I
p__Firmicutes_G
p__Firmicutes_C
p__Firmicutes_B
p__Firmicutes_A
p__Firmicutes
p__Fibrobacterota
p__Euryarchaeota
p__Eremiobacterota
p__Elusimicrobiota
p__Desulfobacterota_A
p__Cyanobacteria
p__Campylobacterota
p__Bdellovibrionota
p__Bacteroidota
p__Actinobacteriota
group
mean_AP
mean_BP
variable
pvalue
p.adjust
0.0
0.2
0.4
0.6
Mean
−0.1
0.0
0.1
Difference (%)
0.25
0.50
0.75
1.00
P Value

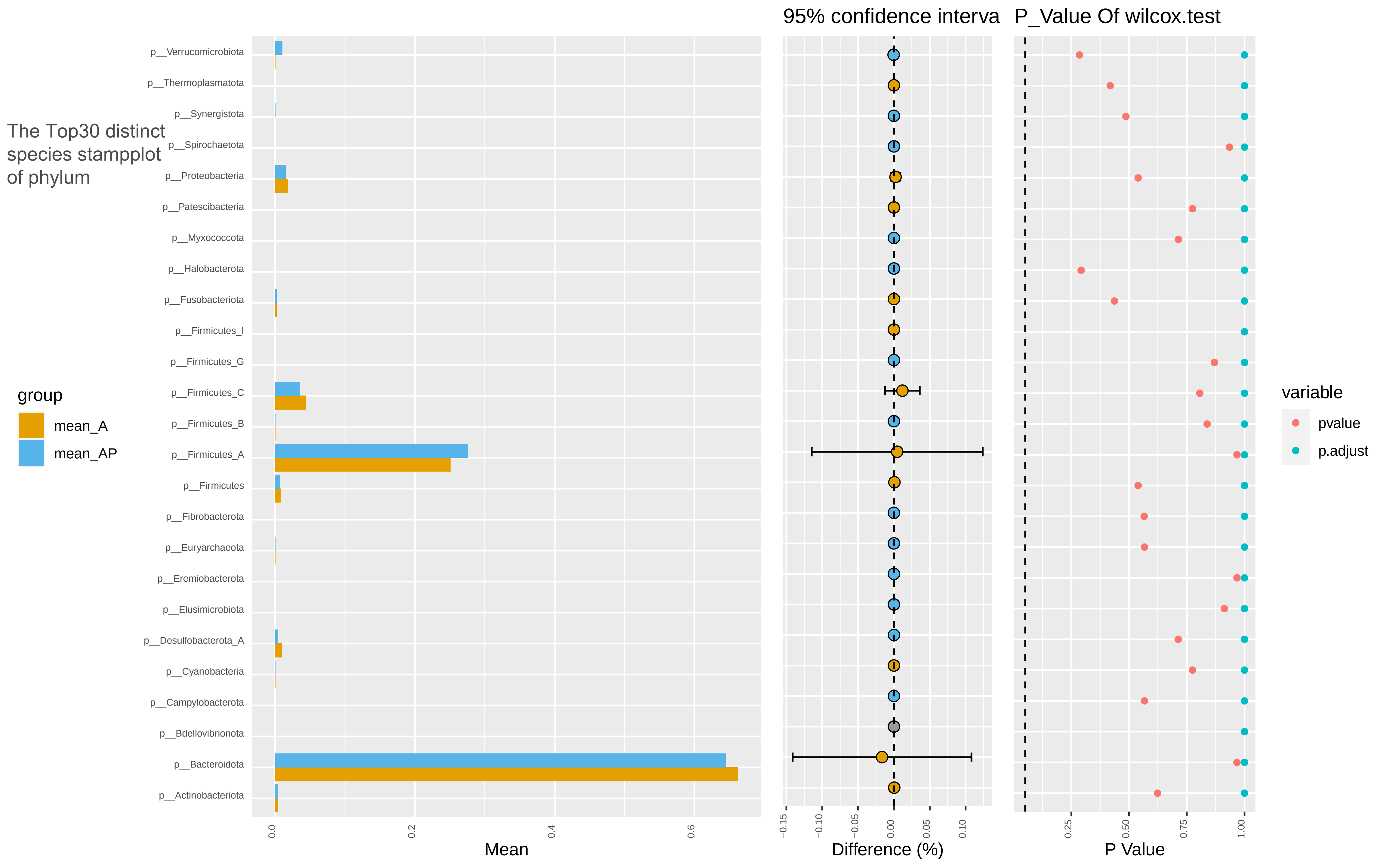

The Top30 distinct species stampplot of phylum
95% confidence interva
P_Value Of wilcox.test
p__Verrucomicrobiota
p__Thermoplasmatota
p__Synergistota
p__Spirochaetota
p__Proteobacteria
p__Patescibacteria
p__Myxococcota
p__Halobacterota
p__Fusobacteriota
p__Firmicutes_I
p__Firmicutes_G
p__Firmicutes_C
p__Firmicutes_B
p__Firmicutes_A
p__Firmicutes
p__Fibrobacterota
p__Euryarchaeota
p__Eremiobacterota
p__Elusimicrobiota
p__Desulfobacterota_A
p__Cyanobacteria
p__Campylobacterota
p__Bdellovibrionota
p__Bacteroidota
p__Actinobacteriota
group
mean_A
mean_AP
variable
pvalue
p.adjust
0.0
0.2
0.4
0.6
Mean
-0.15
-0.10
-0.05
0.00
0.05
0.10
Difference (%)
0.25
0.50
0.75
1.00
P Value

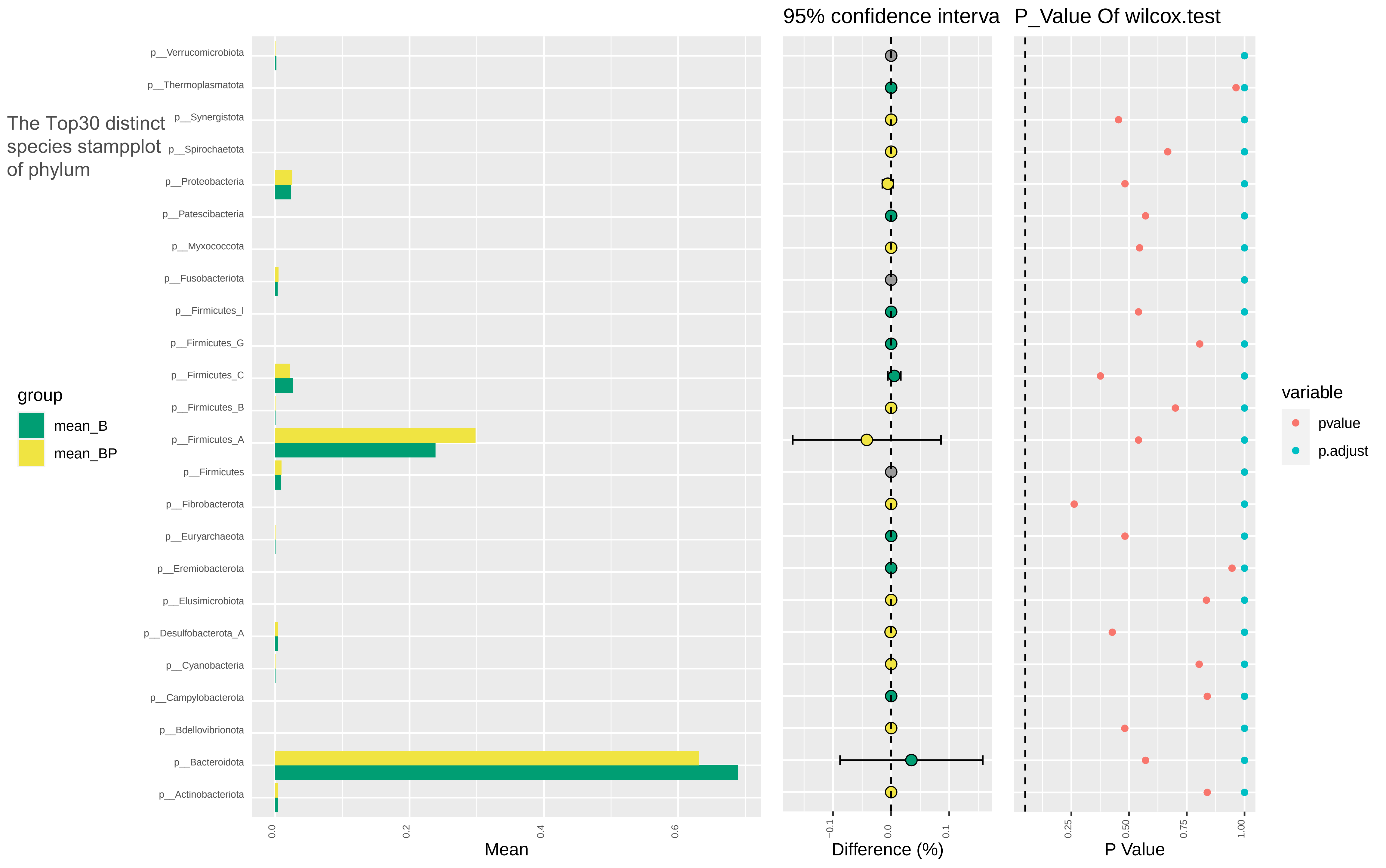

The Top30 distinct species stampplot of phylum
group
mean_B
mean_BP
95% confidence interva
P_Value Of wilcox.test
variable
pvalue
p.adjust
p__Verrucomicrobiota
p__Thermoplasmatota
p__Synergistota
p__Spirochaetota
p__Proteobacteria
p__Patescibacteria
p__Myxococcota
p__Fusobacteriota
p__Firmicutes_I
p__Firmicutes_G
p__Firmicutes_C
p__Firmicutes_B
p__Firmicutes_A
p__Firmicutes
p__Fibrobacterota
p__Euryarchaeota
p__Eremiobacterota
p__Elusimicrobiota
p__Desulfobacterota_A
p__Cyanobacteria
p__Campylobacterota
p__Bdellovibrionota
p__Bacteroidota
p__Actinobacteriota
0.0
0.2
0.4
0.6
Mean
−0.1
0.0
0.1
Difference (%)
0.25
0.50
0.75
1.00
P Value

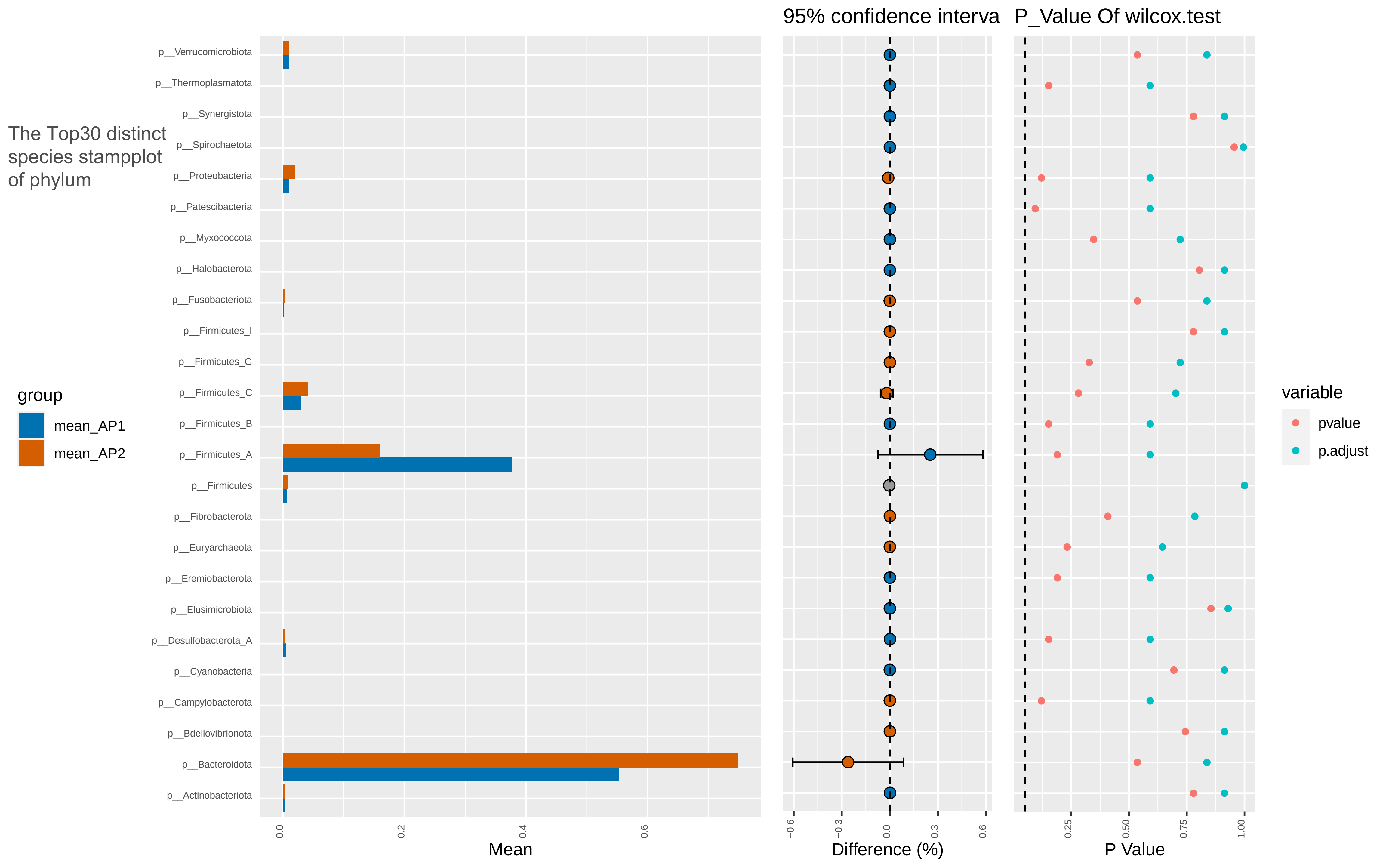

The Top30 distinct species stampplot of phylum
group
mean_AP1
mean_AP2
p__Verrucomicrobiota
p__Thermoplasmatota
p__Synergistota
p__Spirochaetota
p__Proteobacteria
p__Patescibacteria
p__Myxococcota
p__Halobacterota
p__Fusobacteriota
p__Firmicutes_I
p__Firmicutes_G
p__Firmicutes_C
p__Firmicutes_B
p__Firmicutes_A
p__Firmicutes
p__Fibrobacterota
p__Euryarchaeota
p__Eremiobacterota
p__Elusimicrobiota
p__Desulfobacterota_A
p__Cyanobacteria
p__Campylobacterota
p__Bdellovibrionota
p__Bacteroidota
p__Actinobacteriota
0.0
0.2
0.4
0.6
Mean
95% confidence interva
−0.6
−0.3
0.0
0.3
0.6
Difference (%)
P_Value Of wilcox.test
0.25
0.50
0.75
1.00
P Value
variable
pvalue
p.adjust

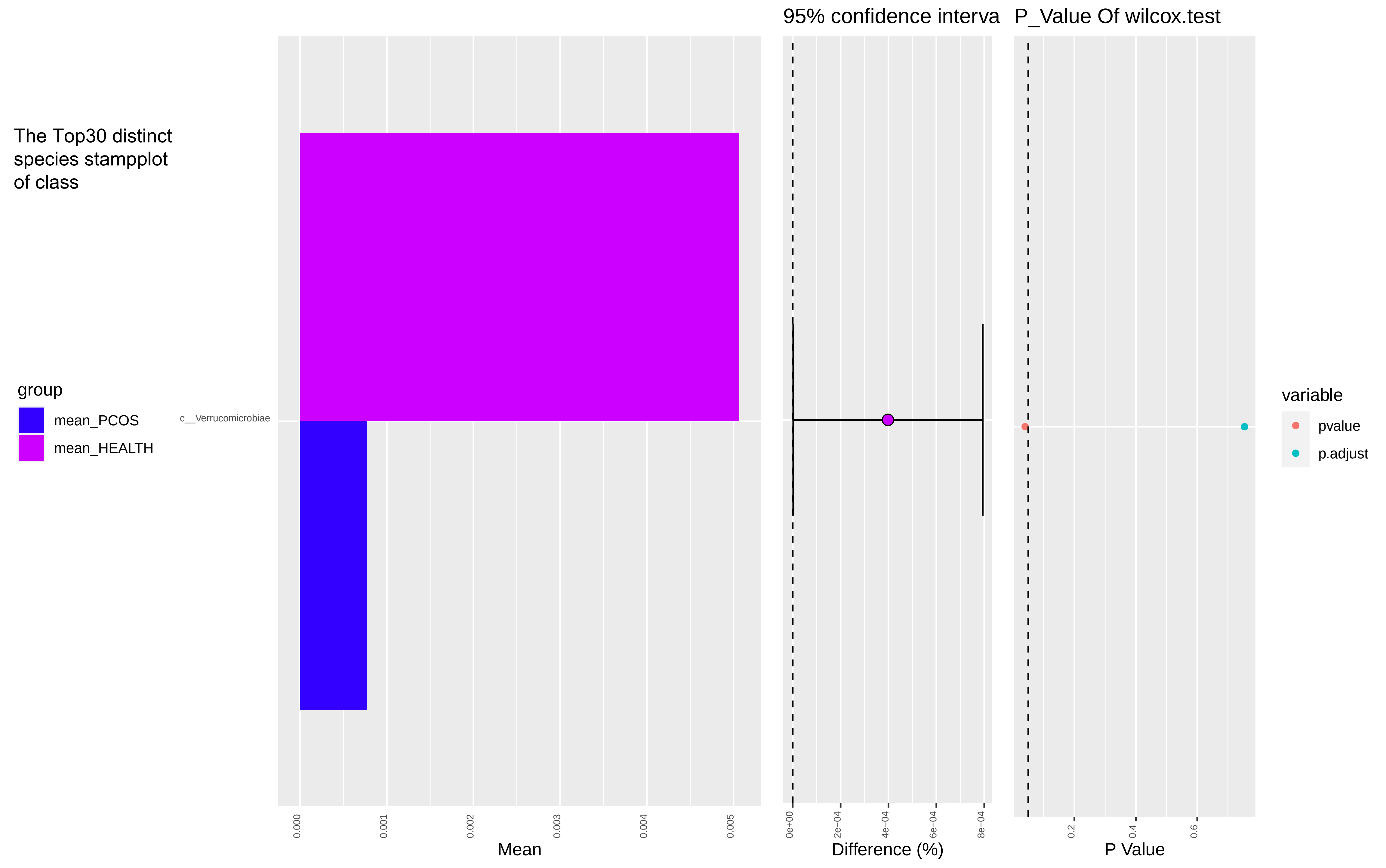

The Top30 distinct species stampplot of class
group
mean_PCOS
mean_HEALTH
c__Verrucomicrobiae
0.000
0.001
0.002
0.003
0.004
0.005
Mean
95% confidence interva
0e+00
2e−04
4e−04
6e−04
8e−04
Difference (%)
P_Value Of wilcox.test
0.2
0.4
0.6
P Value
variable
pvalue
p.adjust

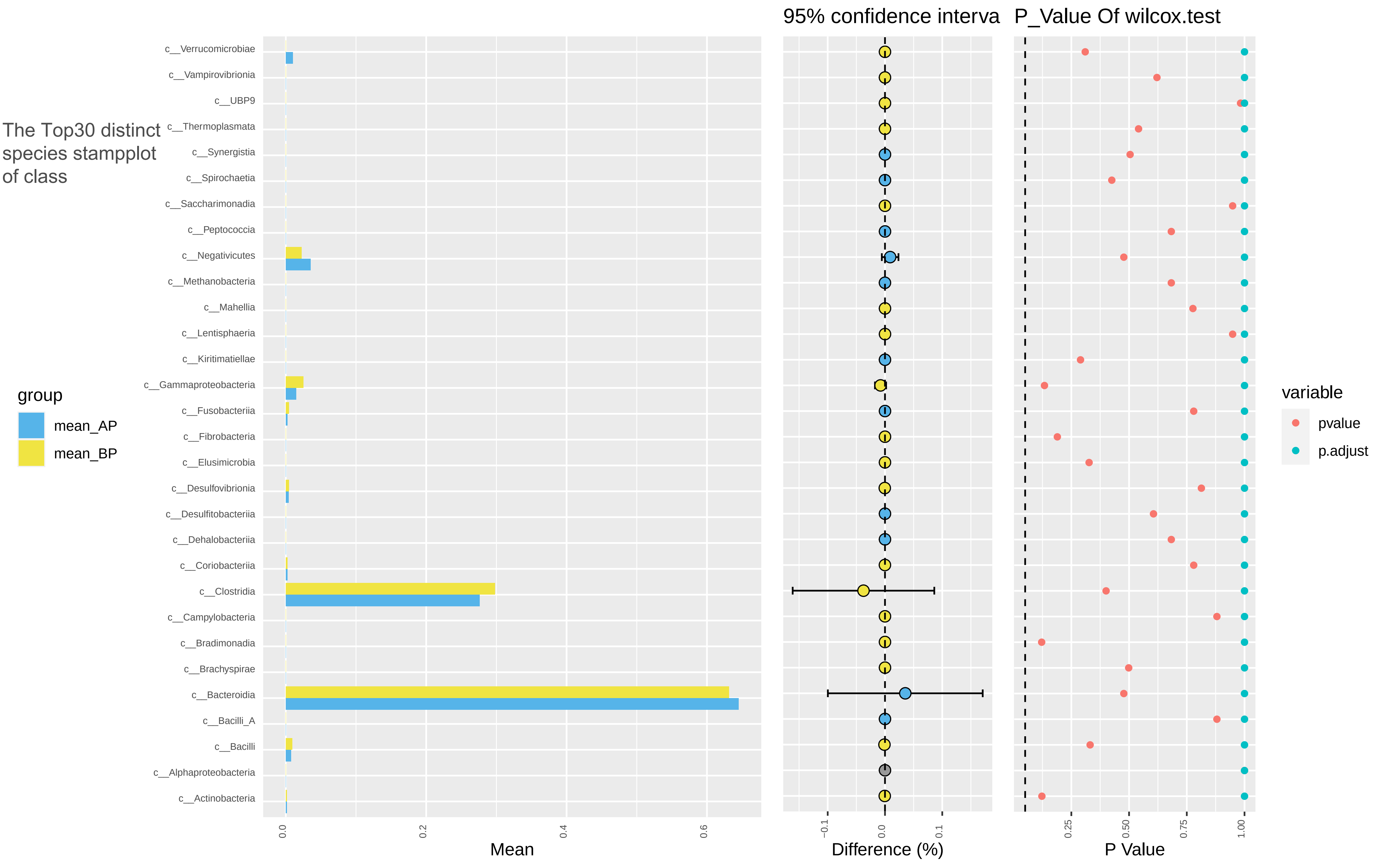

The Top30 distinct species stampplot of class
95% confidence interva
P_Value Of wilcox.test
c__Verrucomicrobiae
c__Vampirovibrionia
c__UBP9
c__Thermoplasmata
c__Synergistia
c__Spirochaetia
c__Saccharimonadia
c__Peptococcia
c__Negativicutes
c__Methanobacteria
c__Mahellia
c__Lentisphaeria
c__Kiritimatiellae
c__Gammaproteobacteria
c__Fusobacteriia
c__Fibrobacteria
c__Elusimicrobia
c__Desulfovibrionia
c__Desulfitobacteriia
c__Dehalobacteriia
c__Coriobacteriia
c__Clostridia
c__Campylobacteria
c__Bradimonadia
c__Brachyspirae
c__Bacteroidia
c__Bacilli_A
c__Bacilli
c__Alphaproteobacteria
c__Actinobacteria
group
mean_AP
mean_BP
0.0
0.2
0.4
0.6
Mean
-0.1
0.0
0.1
Difference (%)
0.25
0.50
0.75
1.00
P Value
variable
pvalue
p.adjust

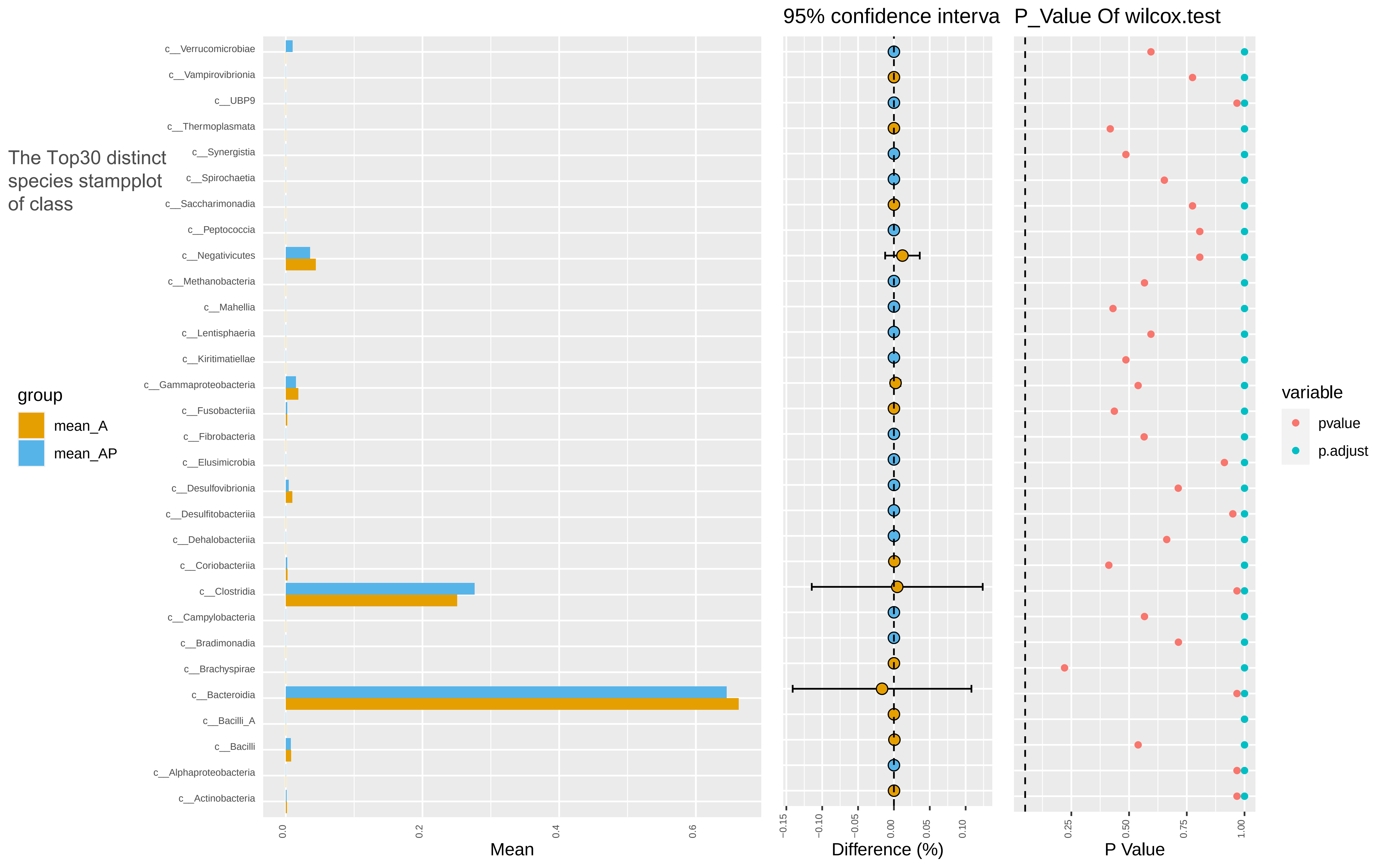

The Top30 distinct species stampplot of class
group
mean_A
mean_AP
c__Verrucomicrobiae
c__Vampirovibrionia
c__UBP9
c__Thermoplasmata
c__Synergistia
c__Spirochaetia
c__Saccharimonadia
c__Peptococcia
c__Negativicutes
c__Methanobacteria
c__Mahellia
c__Lentisphaeria
c__Kiritimatiellae
c__Gammaproteobacteria
c__Fusobacteriia
c__Fibrobacteria
c__Elusimicrobia
c__Desulfovibrionia
c__Desulfitobacteriia
c__Dehalobacteriia
c__Coriobacteriia
c__Clostridia
c__Campylobacteria
c__Bradimonadia
c__Brachyspirae
c__Bacteroidia
c__Bacilli_A
c__Bacilli
c__Alphaproteobacteria
c__Actinobacteria
0.0
0.2
0.4
0.6
Mean
95% confidence interva
−0.15
−0.10
−0.05
0.00
0.05
0.10
Difference (%)
P_Value Of wilcox.test
0.25
0.50
0.75
1.00
P Value
variable
pvalue
p.adjust

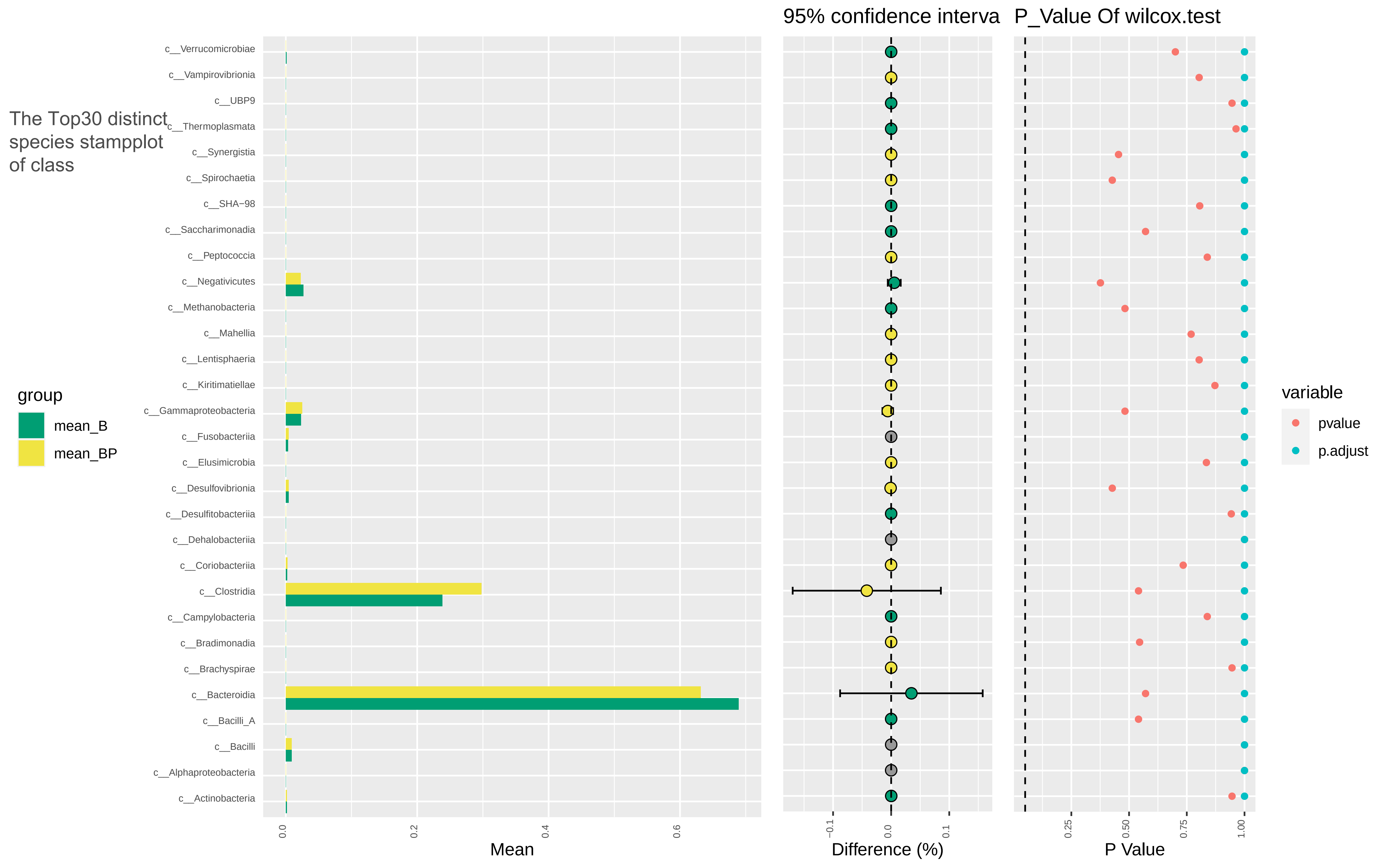

The Top30 distinct species stampplot of class
95% confidence interva
P_Value Of wilcox.test
c__Verrucomicrobiae
c__Vampirovibrionia
c__UBP9
c__Thermoplasmata
c__Synergistia
c__Spirochaetia
c__SHA−98
c__Saccharimonadia
c__Peptococcia
c__Negativicutes
c__Methanobacteria
c__Mahellia
c__Lentisphaeria
c__Kiritimatiellae
c__Gammaproteobacteria
c__Fusobacteriia
c__Elusimicrobia
c__Desulfovibrionia
c__Desulfitobacteriia
c__Dehalobacteriia
c__Coriobacteriia
c__Clostridia
c__Campylobacteria
c__Bradimonadia
c__Brachyspirae
c__Bacteroidia
c__Bacilli_A
c__Bacilli
c__Alphaproteobacteria
c__Actinobacteria
group
mean_B
mean_BP
0.0
0.2
0.4
0.6
Mean
−0.1
0.0
0.1
Difference (%)
0.25
0.50
0.75
1.00
P Value
variable
pvalue
p.adjust

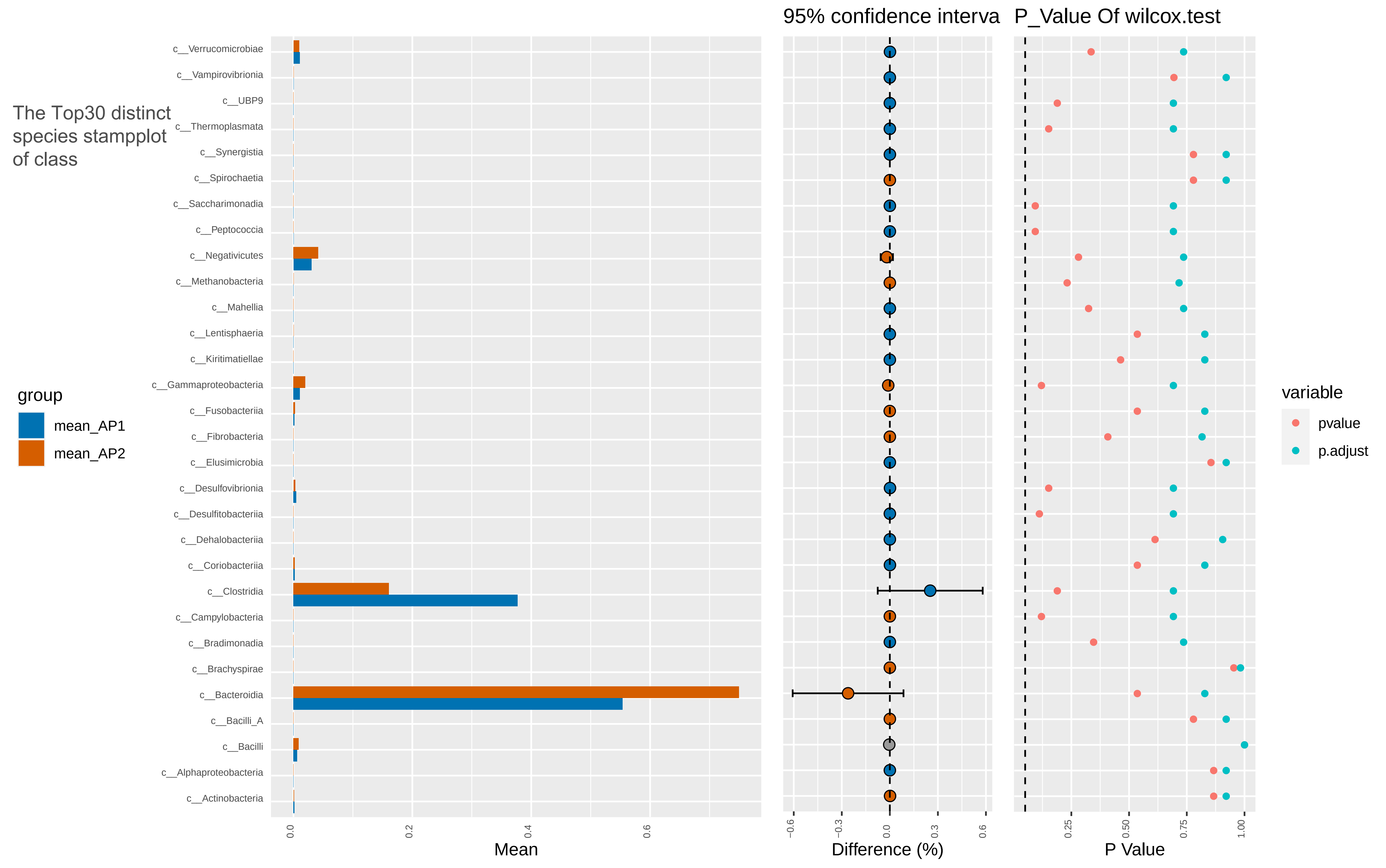

The Top30 distinct species stampplot of class
95% confidence interva
P_Value Of wilcox.test
c__Verrucomicrobiae
c__Vampirovibrionia
c__UBP9
c__Thermoplasmata
c__Synergistia
c__Spirochaetia
c__Saccharimonadia
c__Peptococcia
c__Negativicutes
c__Methanobacteria
c__Mahellia
c__Lentisphaeria
c__Kiritimatiellae
c__Gammaproteobacteria
c__Fusobacteriia
c__Fibrobacteria
c__Elusimicrobia
c__Desulfovibrionia
c__Desulfitobacteriia
c__Dehalobacteriia
c__Coriobacteriia
c__Clostridia
c__Campylobacteria
c__Bradimonadia
c__Brachyspirae
c__Bacteroidia
c__Bacilli_A
c__Bacilli
c__Alphaproteobacteria
c__Actinobacteria
group
mean_AP1
mean_AP2
0.0
0.2
0.4
0.6
Mean
−0.6
−0.3
0.0
0.3
0.6
Difference (%)
0.25
0.50
0.75
1.00
P Value
variable
pvalue
p.adjust

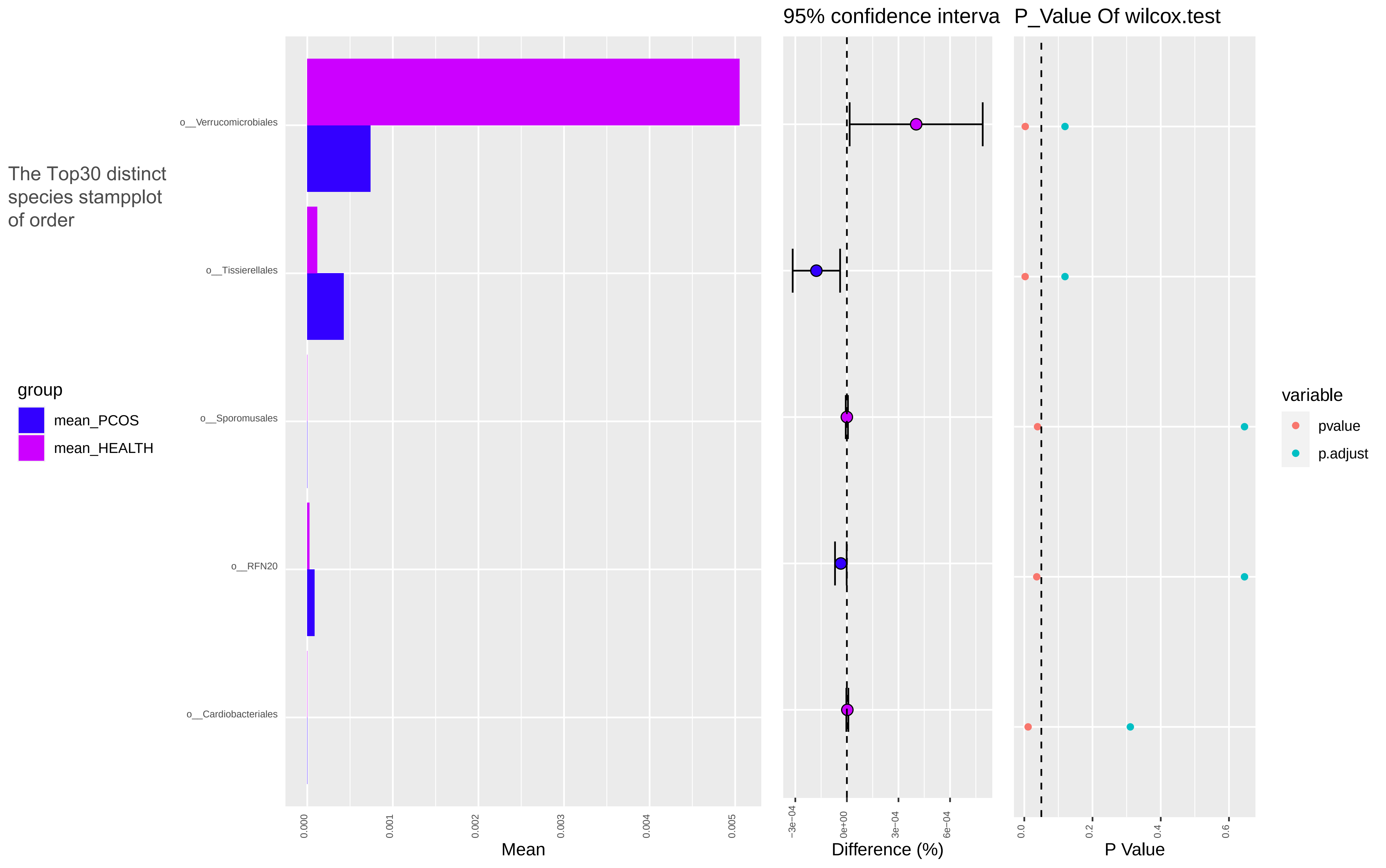

The Top30 distinct species stampplot of order
group
mean_PCOS
mean_HEALTH
o__Verrucomicrobiales
o__Tissierellales
o__Sporomusales
o__RFN20
o__Cardiobacteriales
0.000
0.001
0.002
0.003
0.004
0.005
Mean
95% confidence interva
−3e−04
0e+00
3e−04
6e−04
Difference (%)
P_Value Of wilcox.test
0.0
0.2
0.4
0.6
P Value
variable
pvalue
p.adjust

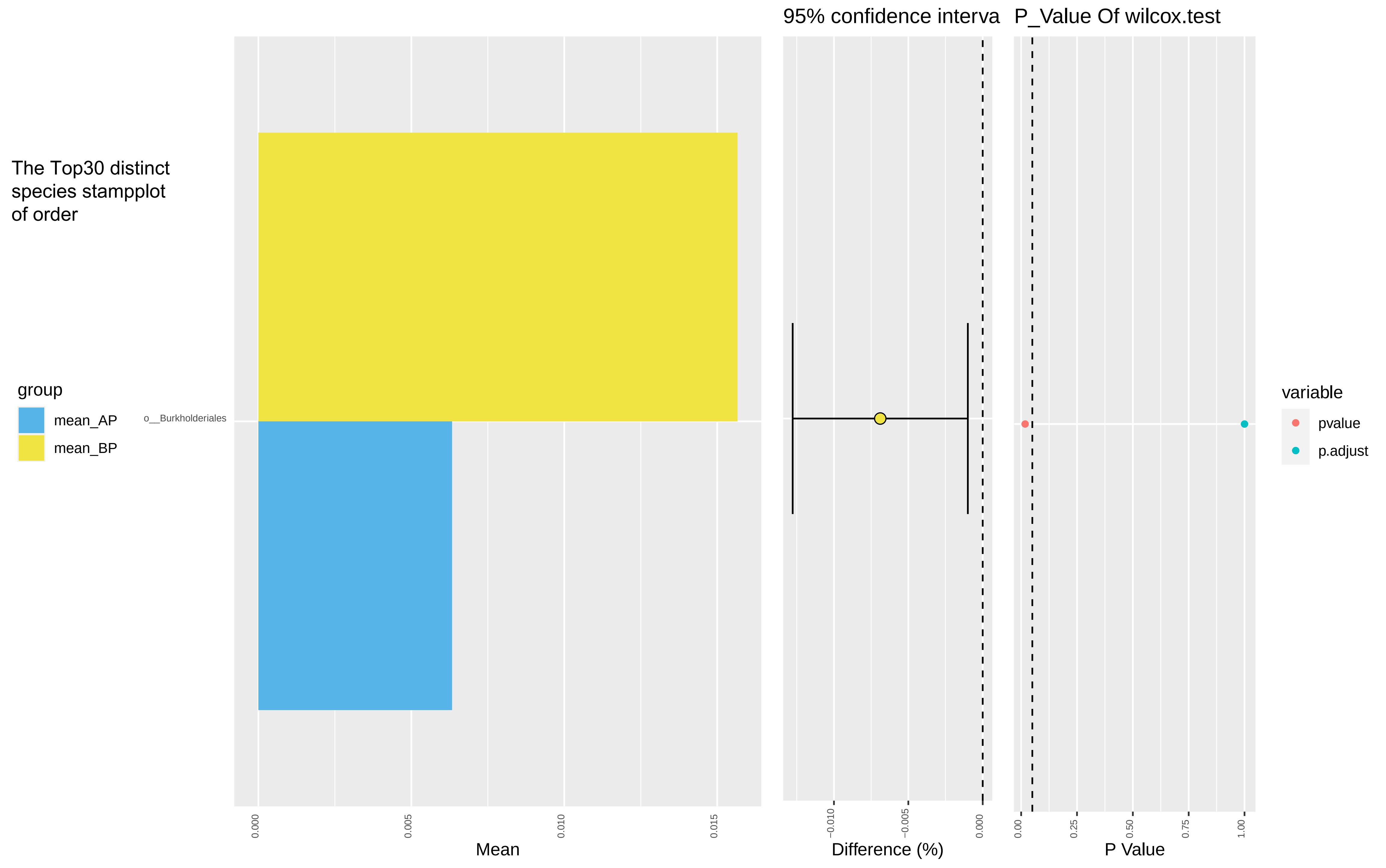

The Top30 distinct species stampplot of order
group
mean_AP
mean_BP
o__Burkholderiales
0.000
0.005
0.010
0.015
Mean
95% confidence interva
−0.010
−0.005
0.000
Difference (%)
P_Value Of wilcox.test
0.00
0.25
0.50
0.75
1.00
P Value
variable
pvalue
p.adjust

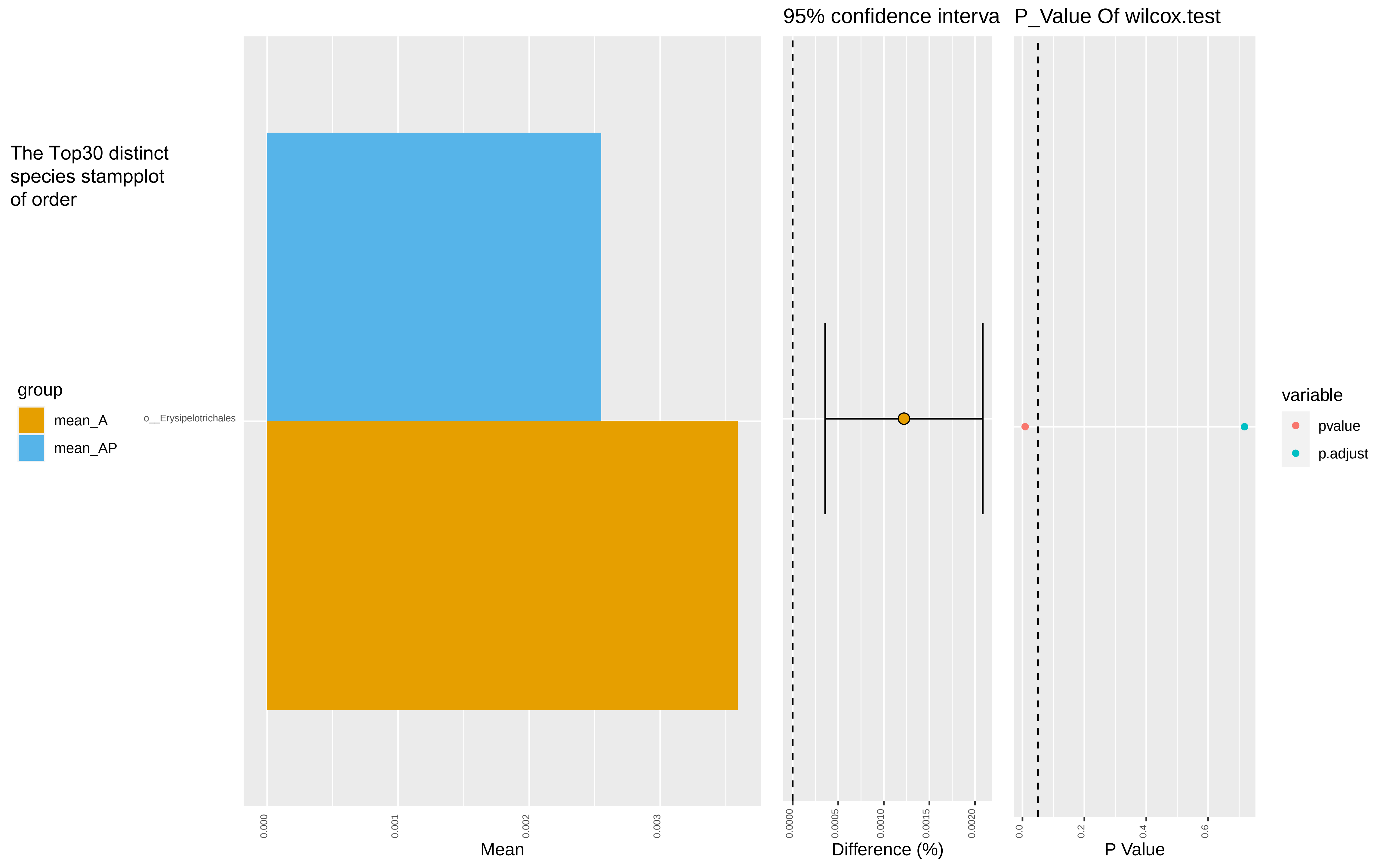

95% confidence interva
P_Value Of wilcox.test
The Top30 distinct species stampplot of order
group
mean_A
mean_AP
o__Erysipelotrichales
0.000
0.001
0.002
0.003
Mean
0.0000
0.0005
0.0010
0.0015
0.0020
Difference (%)
0.0
0.2
0.4
0.6
P Value
variable
pvalue
p.adjust

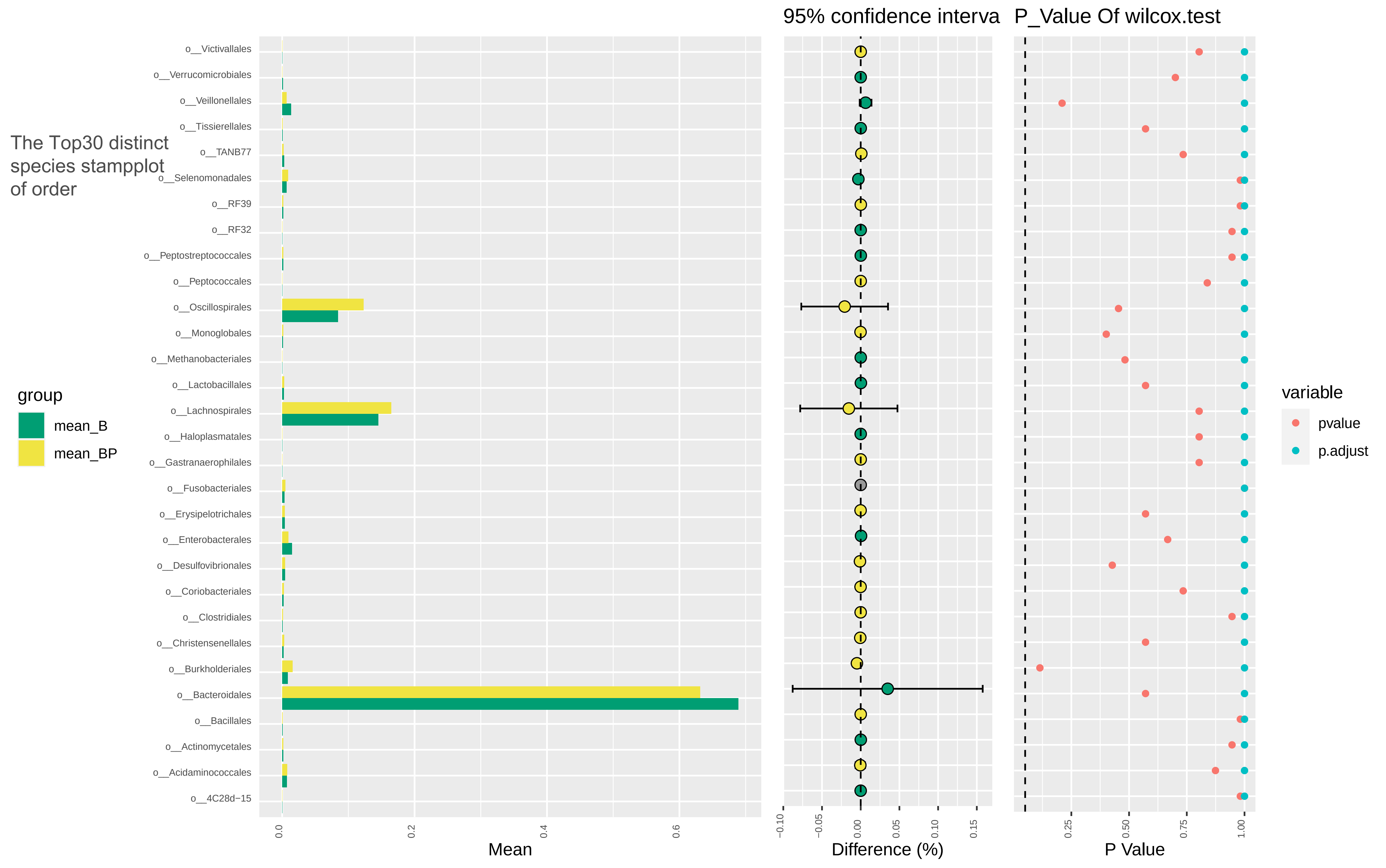

95% confidence interva
P_Value Of wilcox.test
The Top30 distinct species stampplot of order
o__Victivallales
o__Verrucomicrobiales
o__Veillonellales
o__Tissierellales
o__TANB77
o__Selenomonadales
o__RF39
o__RF32
o__Peptostreptococcales
o__Peptococcales
o__Oscillospirales
o__Monoglobales
o__Methanobacteriales
o__Lactobacillales
o__Lachnospirales
o__Haloplasmatales
o__Gastranaerophilales
o__Fusobacteriales
o__Erysipelotrichales
o__Enterobacterales
o__Desulfovibrionales
o__Coriobacteriales
o__Clostridiales
o__Christensenellales
o__Burkholderiales
o__Bacteroidales
o__Bacillales
o__Actinomycetales
o__Acidaminococcales
o__4C28d−15
group
mean_B
mean_BP
0.0
0.2
0.4
0.6
Mean
−0.10
−0.05
0.00
0.05
0.10
0.15
Difference (%)
0.25
0.50
0.75
1.00
P Value
variable
pvalue
p.adjust

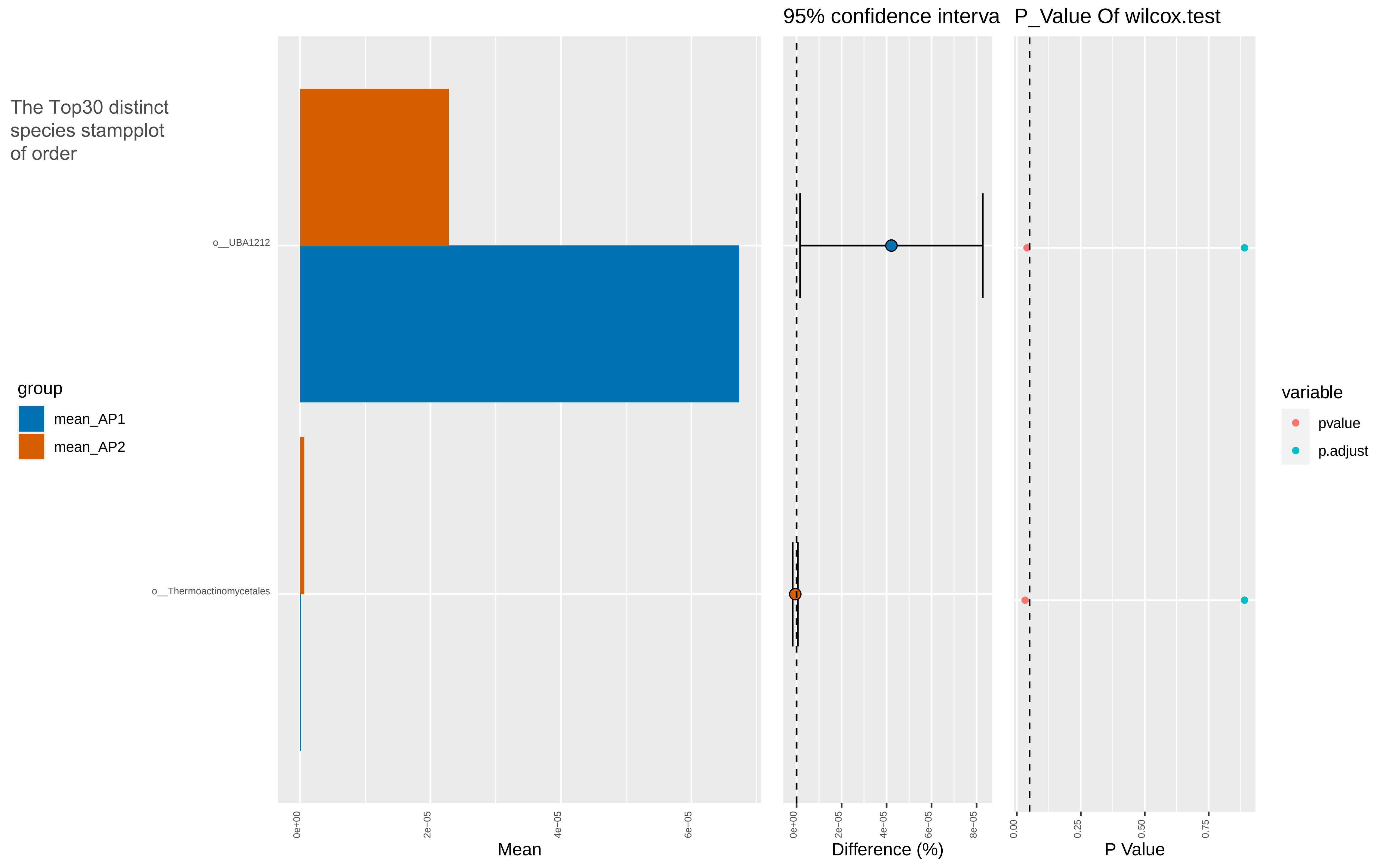
95% confidence interva
P_Value Of wilcox.test
The Top30 distinct species stampplot of order
o__UBA1212
o__Thermoactinomycetales
group
mean_AP1
mean_AP2
variable
pvalue
p.adjust
0e+00
2e−05
4e−05
6e−05
Mean
0e+00
2e−05
4e−05
6e−05
8e−05
Difference (%)
0.00
0.25
0.50
0.75
P Value

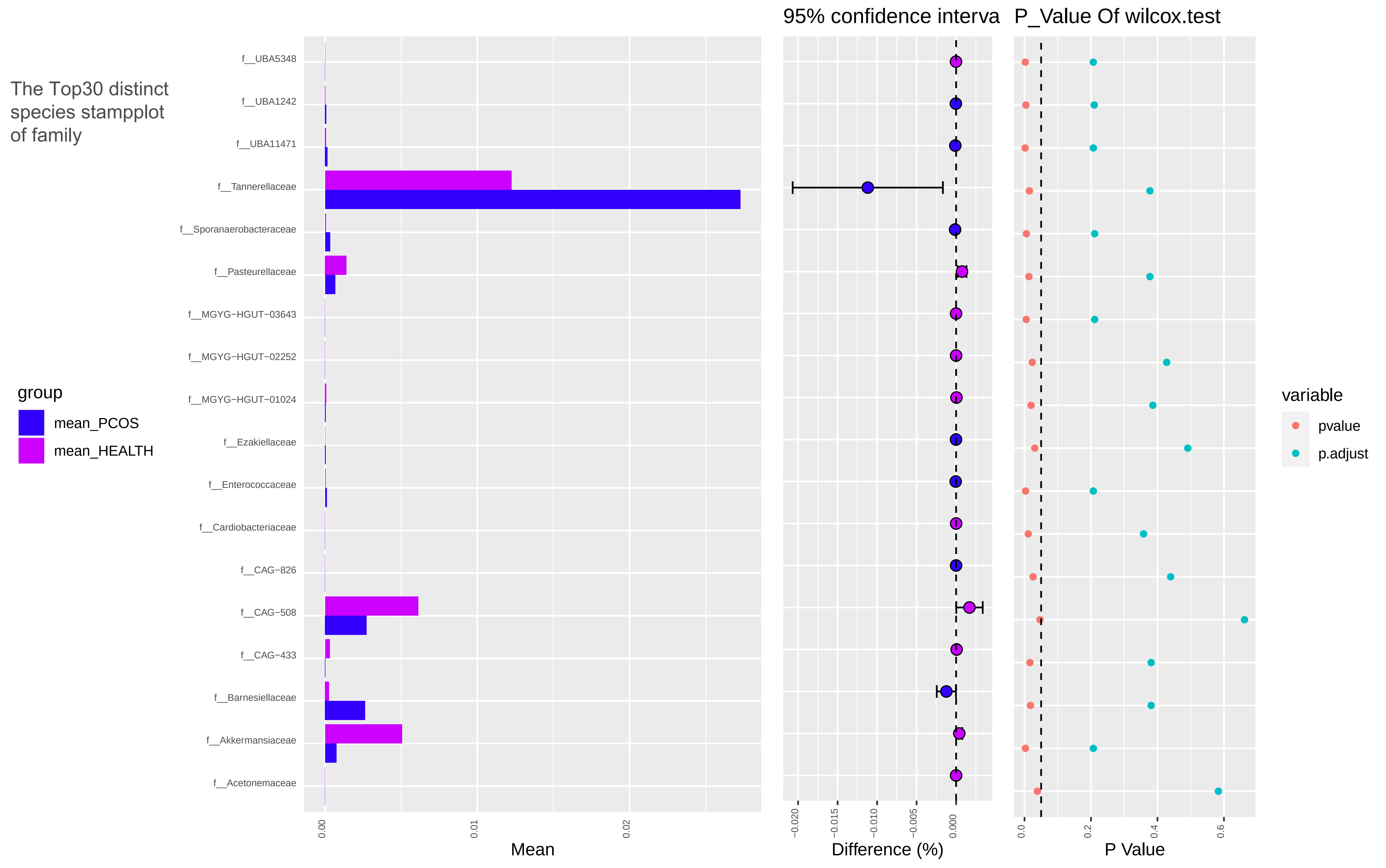

The Top30 distinct species stampplot of family
95% confidence interva
P_Value Of wilcox.test
f__UBA5348
f__UBA1242
f__UBA11471
f__Tannerellaceae
f__Sporanaerobacteraceae
f__Pasteurellaceae
f__MGYG−HGUT−03643
f__MGYG−HGUT−02252
f__MGYG−HGUT−01024
f__Ezakiellaceae
f__Enterococcaceae
f__Cardiobacteriaceae
f__CAG−826
f__CAG−508
f__CAG−433
f__Barnesiellaceae
f__Akkermansiaceae
f__Acetonemaceae
group
mean_PCOS
mean_HEALTH
0.00
0.01
0.02
Mean
−0.020
−0.015
−0.010
−0.005
0.000
Difference (%)
0.0
0.2
0.4
0.6
P Value
variable
pvalue
p.adjust

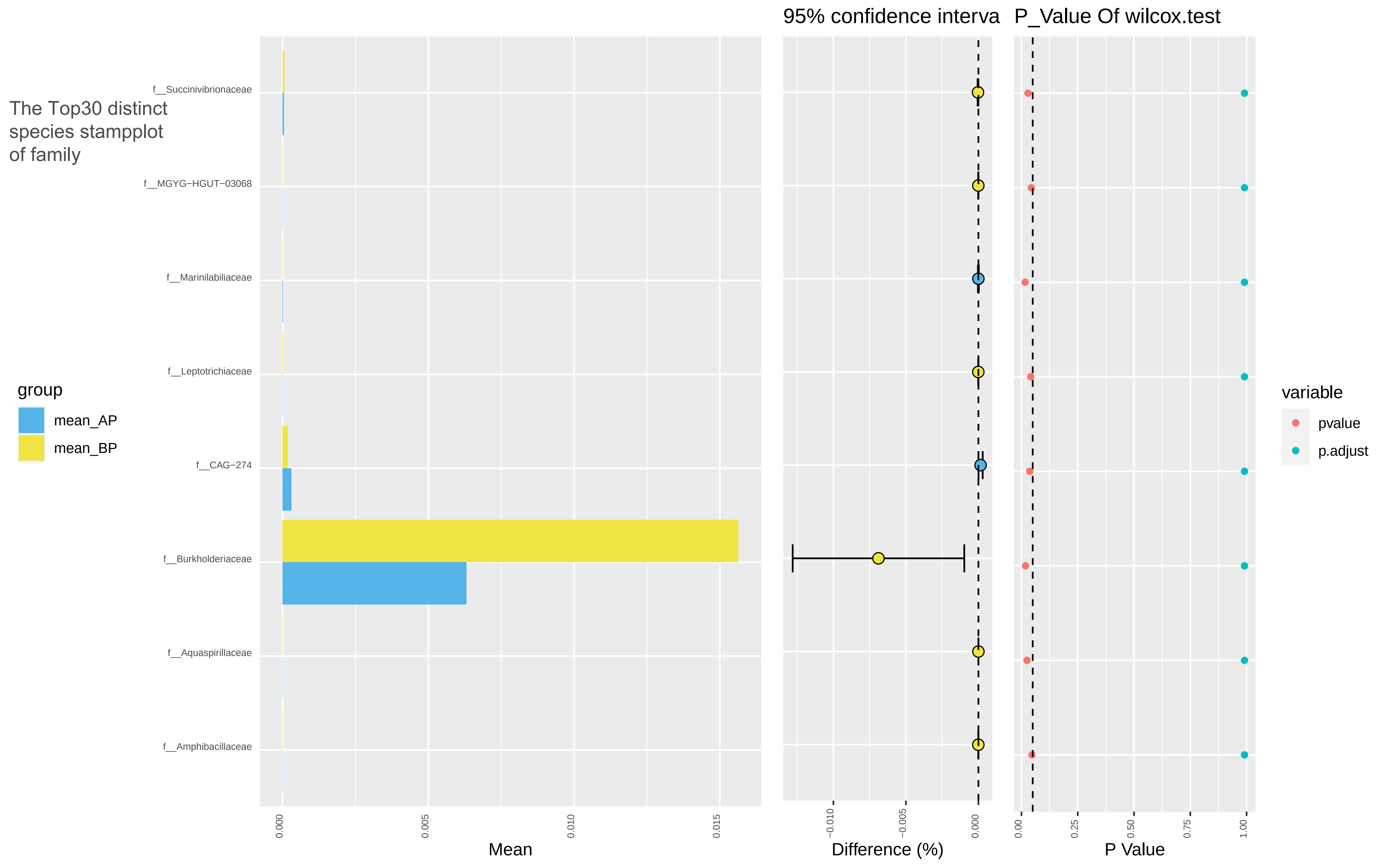

The Top30 distinct species stampplot of family
95% confidence interva
P_Value Of wilcox.test
f__Succinivibrionaceae
f__MGYG−HGUT−03068
f__Marinilabiliaceae
f__Leptotrichiaceae
f__CAG−274
f__Burkholderiaceae
f__Aquaspirillaceae
f__Amphibacillaceae
group
mean_AP
mean_BP
variable
pvalue
p.adjust
0.000
0.005
0.010
0.015
Mean
−0.010
−0.005
0.000
Difference (%)
0.00
0.25
0.50
0.75
1.00
P Value

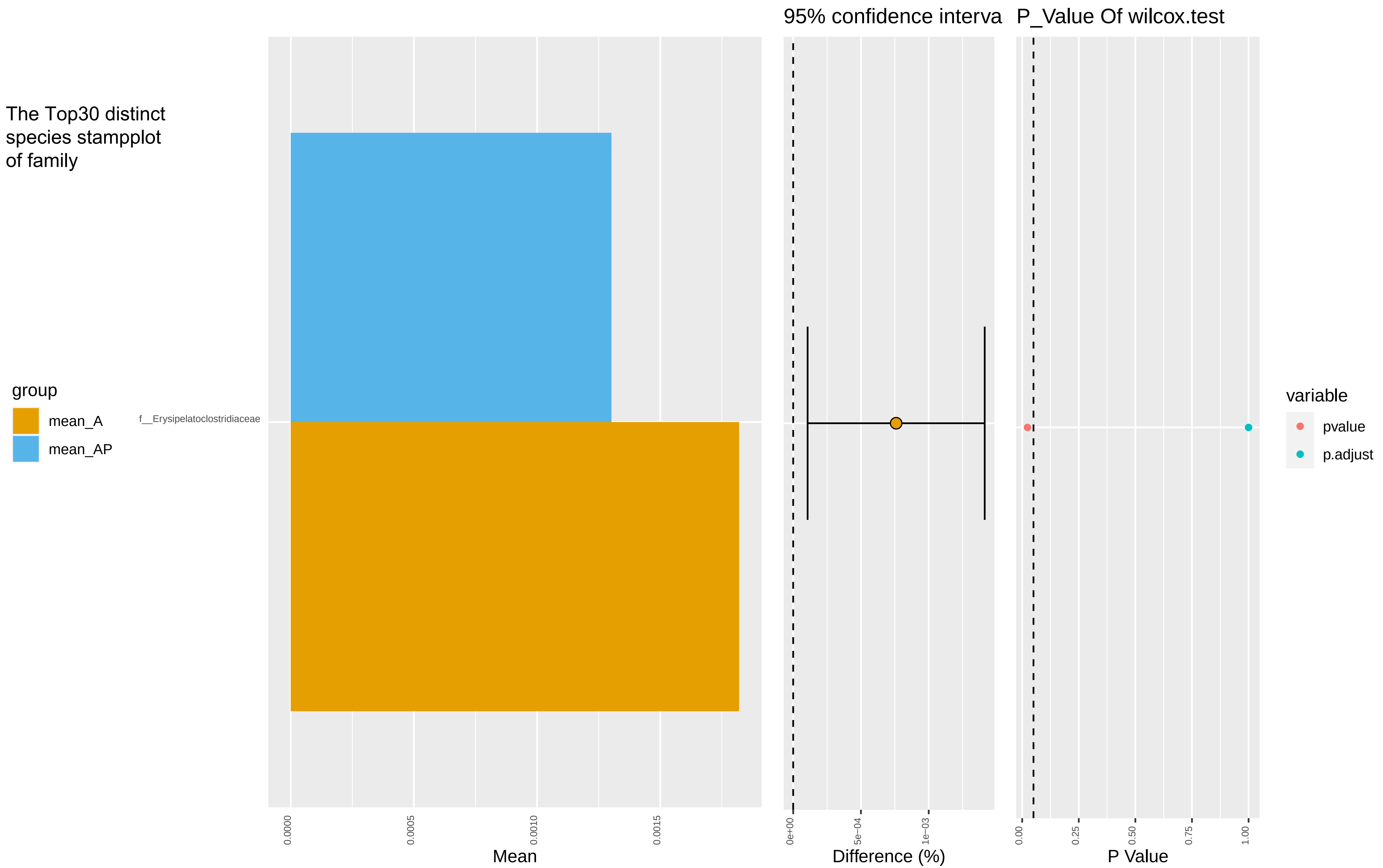

The Top30 distinct species stampplot of family
group
mean_A
mean_AP
f__Erysipelatoclostridiaceae
0.0000
0.0005
0.0010
0.0015
Mean
95% confidence interva
0e+00
5e−04
1e−03
Difference (%)
P_Value Of wilcox.test
0.00
0.25
0.50
0.75
1.00
P Value
variable
pvalue
p.adjust

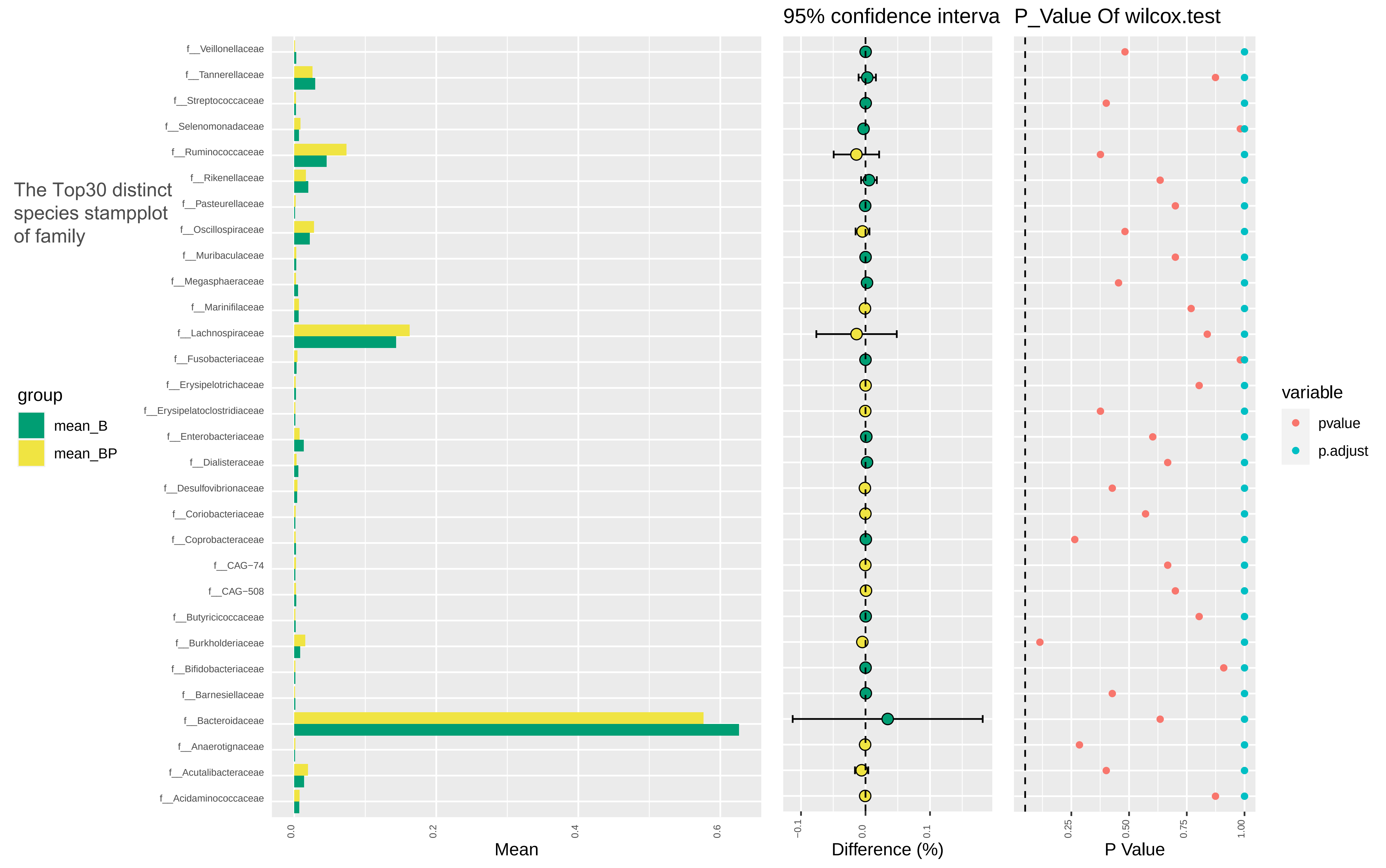

The Top30 distinct species stampplot of family
group
mean_B
mean_BP
f__Veillonellaceae
f__Tannerellaceae
f__Streptococcaceae
f__Selenomonadaceae
f__Ruminococcaceae
f__Rikenellaceae
f__Pasteurellaceae
f__Oscillospiraceae
f__Muribaculaceae
f__Megasphaeraceae
f__Marinifilaceae
f__Lachnospiraceae
f__Fusobacteriaceae
f__Erysipelotrichaceae
f__Erysipelatoclostridiaceae
f__Enterobacteriaceae
f__Dialisteraceae
f__Desulfovibrionaceae
f__Coriobacteriaceae
f__Coprobacteraceae
f__CAG−74
f__CAG−508
f__Butyricicoccaceae
f__Burkholderiaceae
f__Bifidobacteriaceae
f__Barnesiellaceae
f__Bacteroidaceae
f__Anaerotignaceae
f__Acutalibacteraceae
f__Acidaminococcaceae
0.0
0.2
0.4
0.6
Mean
95% confidence interva
−0.1
0.0
0.1
Difference (%)
P_Value Of wilcox.test
0.25
0.50
0.75
1.00
P Value
variable
pvalue
p.adjust

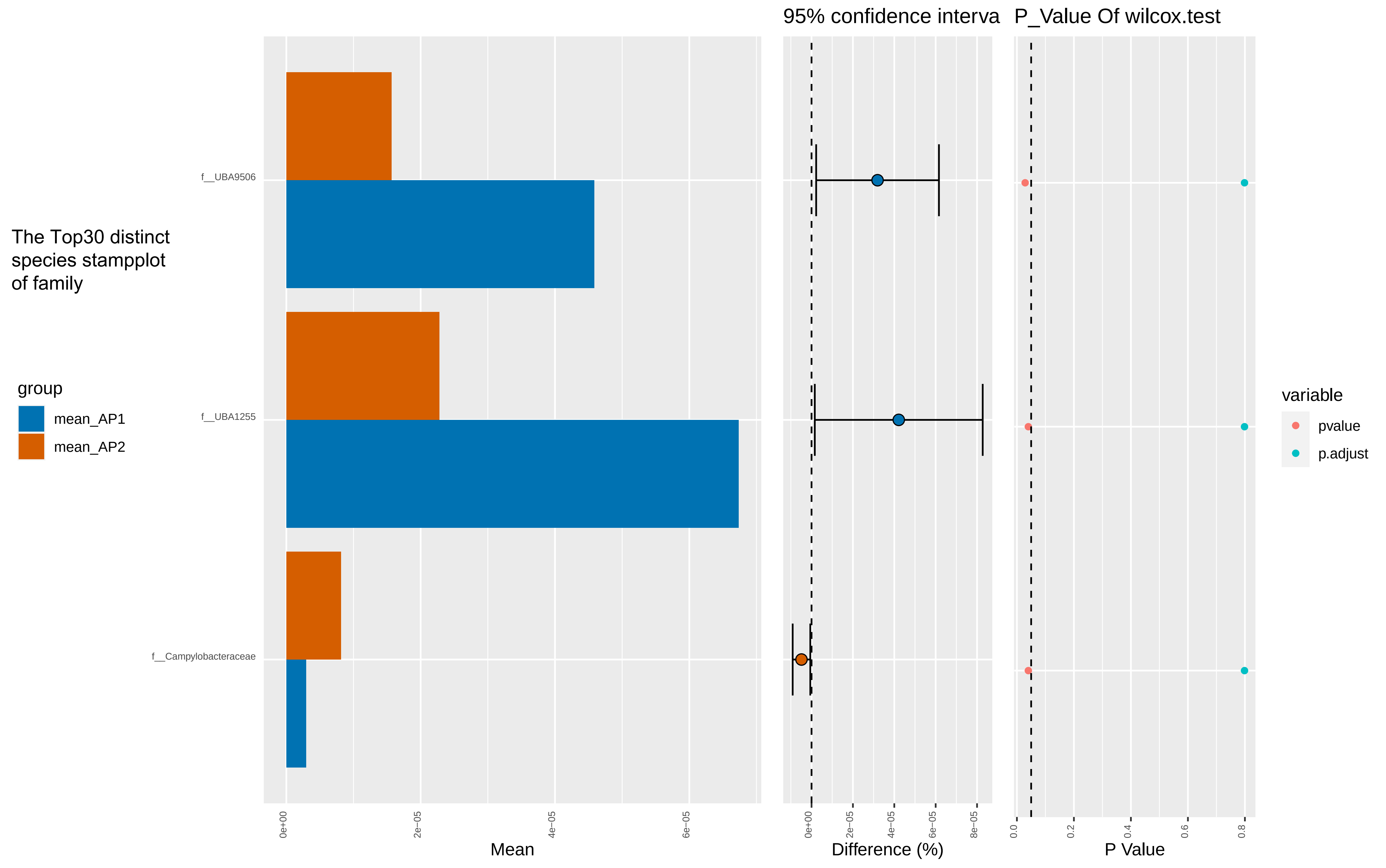

95% confidence interva
P_Value Of wilcox.test
The Top30 distinct species stampplot of family
group
mean_AP1
mean_AP2
f__UBA9506
f__UBA1255
f__Campylobacteraceae
0e+00
2e−05
4e−05
6e−05
Mean
0e+00
2e−05
4e−05
6e−05
8e−05
Difference (%)
0.0
0.2
0.4
0.6
0.8
P Value
variable
pvalue
p.adjust

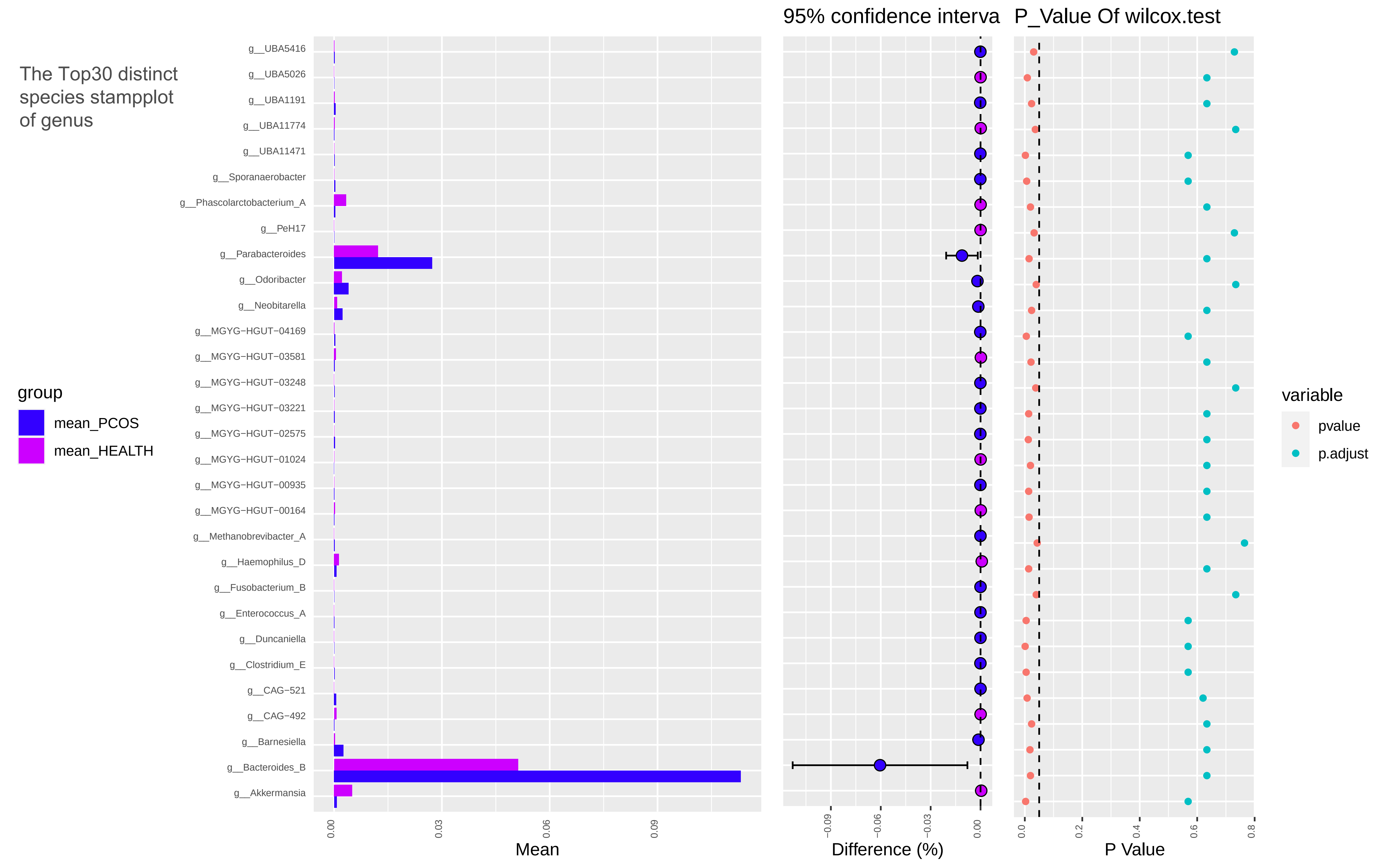

The Top30 distinct species stampplot of genus
group
mean_PCOS
mean_HEALTH
95% confidence interva
P_Value Of wilcox.test
variable
pvalue
p.adjust
g__UBA5416
g__UBA5026
g__UBA1191
g__UBA11774
g__UBA11471
g__Sporanaerobacter
g__Phascolarctobacterium_A
g__PeH17
g__Parabacteroides
g__Odoribacter
g__Neobitarella
g__MGYG−HGUT−04169
g__MGYG−HGUT−03581
g__MGYG−HGUT−03248
g__MGYG−HGUT−03221
g__MGYG−HGUT−02575
g__MGYG−HGUT−01024
g__MGYG−HGUT−00935
g__MGYG−HGUT−00164
g__Methanobrevibacter_A
g__Haemophilus_D
g__Fusobacterium_B
g__Enterococcus_A
g__Duncaniella
g__Clostridium_E
g__CAG−521
g__CAG−492
g__Barnesiella
g__Bacteroides_B
g__Akkermansia
0.00
0.03
0.06
0.09
Mean
−0.09
−0.06
−0.03
0.00
Difference (%)
0.0
0.2
0.4
0.6
0.8
P Value

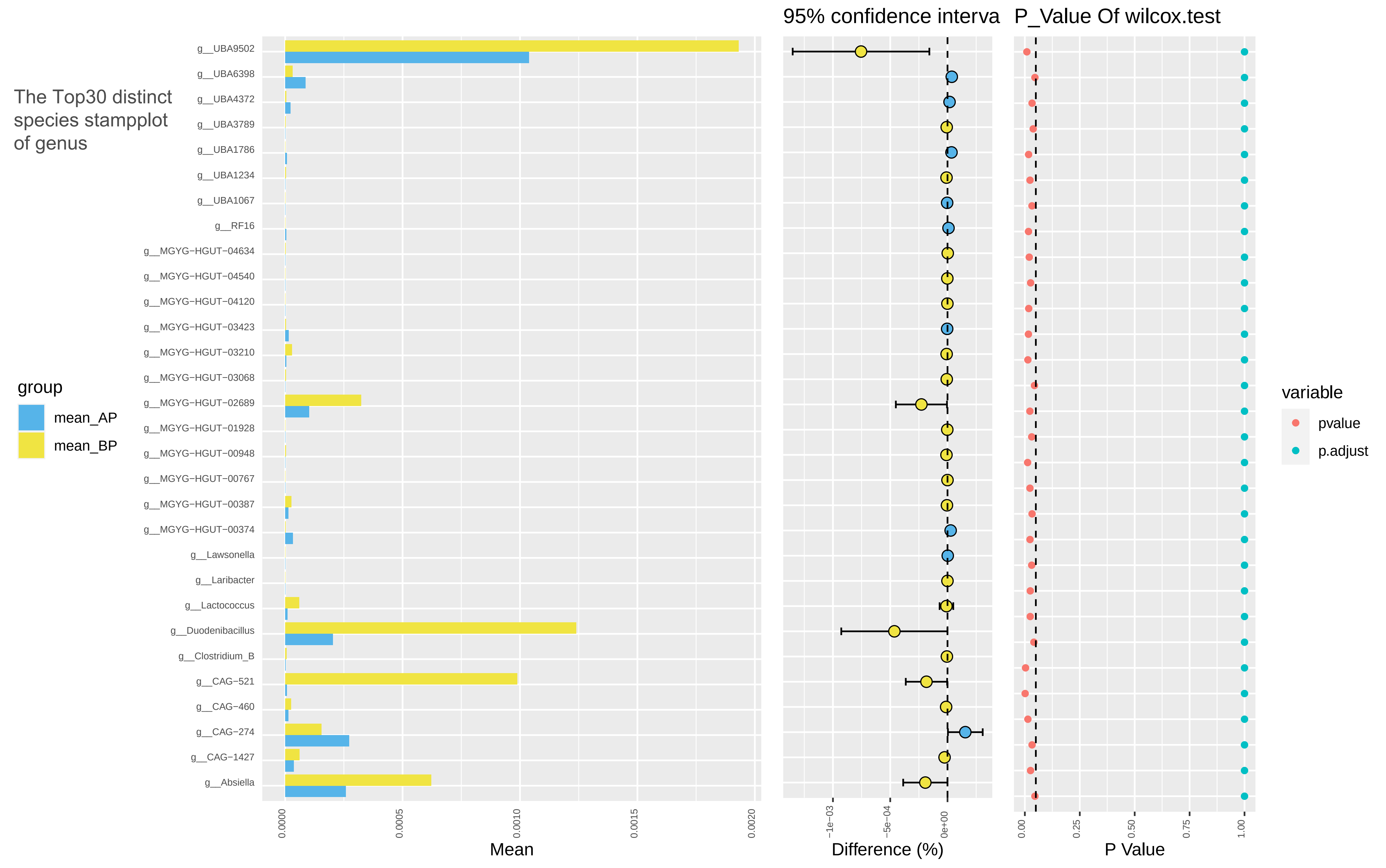

The Top30 distinct species stampplot of genus
95% confidence interva
P_Value Of wilcox.test
g__UBA9502
g__UBA6398
g__UBA4372
g__UBA3789
g__UBA1786
g__UBA1234
g__UBA1067
g__RF16
g__MGYG−HGUT−04634
g__MGYG−HGUT−04540
g__MGYG−HGUT−04120
g__MGYG−HGUT−03423
g__MGYG−HGUT−03210
g__MGYG−HGUT−03068
g__MGYG−HGUT−02689
g__MGYG−HGUT−01928
g__MGYG−HGUT−00948
g__MGYG−HGUT−00767
g__MGYG−HGUT−00387
g__MGYG−HGUT−00374
g__Lawsonella
g__Laribacter
g__Lactococcus
g__Duodenibacillus
g__Clostridium_B
g__CAG−521
g__CAG−460
g__CAG−274
g__CAG−1427
g__Absiella
group
mean_AP
mean_BP
0.0000
0.0005
0.0010
0.0015
0.0020
Mean
−1e−03
−5e−04
0e+00
Difference (%)
0.00
0.25
0.50
0.75
1.00
P Value
variable
pvalue
p.adjust

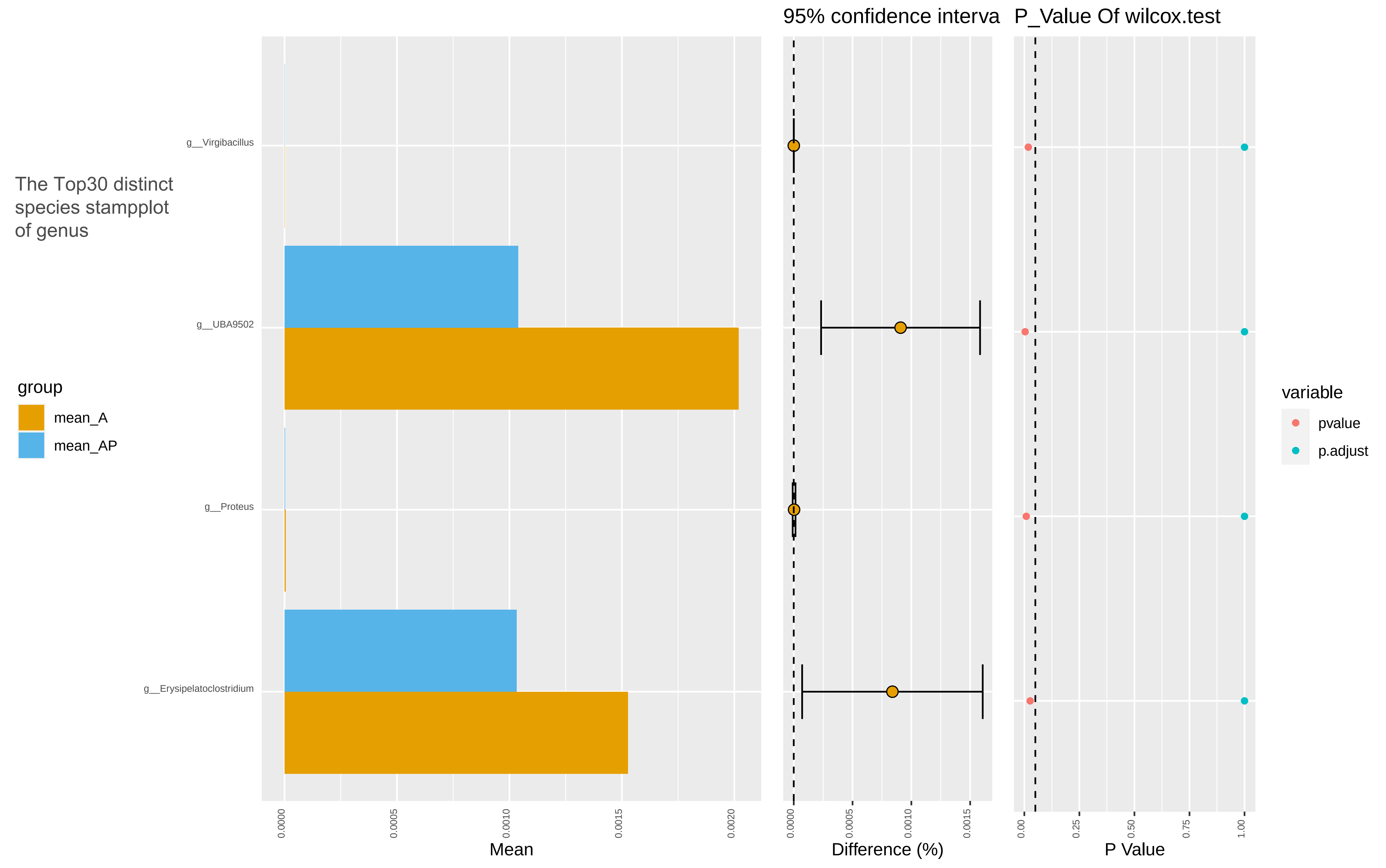

The Top30 distinct species stampplot of genus
95% confidence interva
P_Value Of wilcox.test
g__Virgibacillus
g__UBA9502
g__Proteus
g__Erysipelatoclostridium
group
mean_A
mean_AP
variable
pvalue
p.adjust
0.0000
0.0005
0.0010
0.0015
0.0020
Mean
Difference (%)
0.00
0.25
0.50
0.75
1.00
P Value

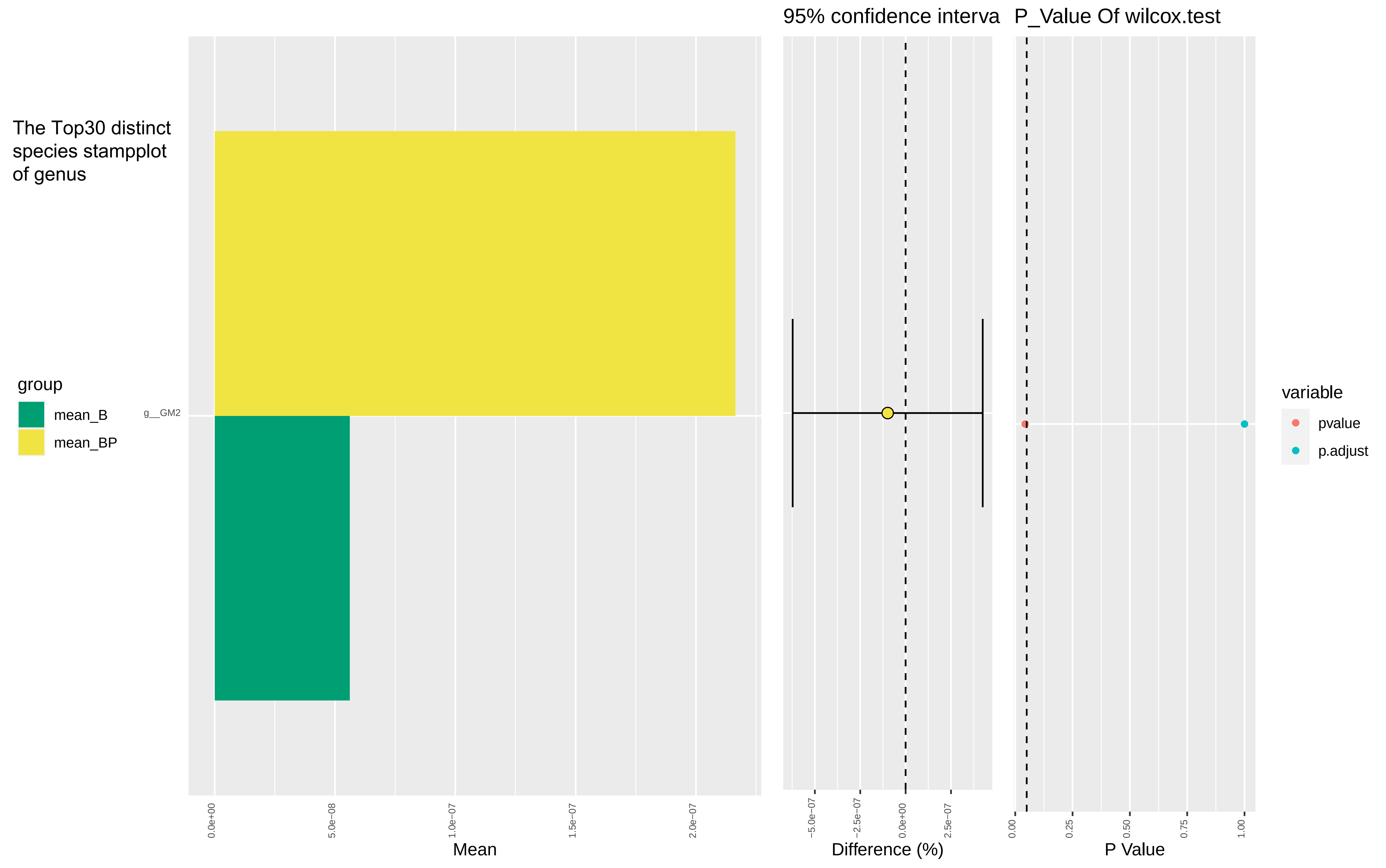

The Top30 distinct species stampplot of genus
95% confidence interva
P_Value Of wilcox.test
group
mean_B
mean_BP
g__GM2
Mean
0.0e+00
5.0e−08
1.0e−07
1.5e−07
2.0e−07
Difference (%)
−5.0e−07
−2.5e−07
0.0e+00
2.5e−07
P Value
0.00
0.25
0.50
0.75
1.00
variable
pvalue
p.adjust

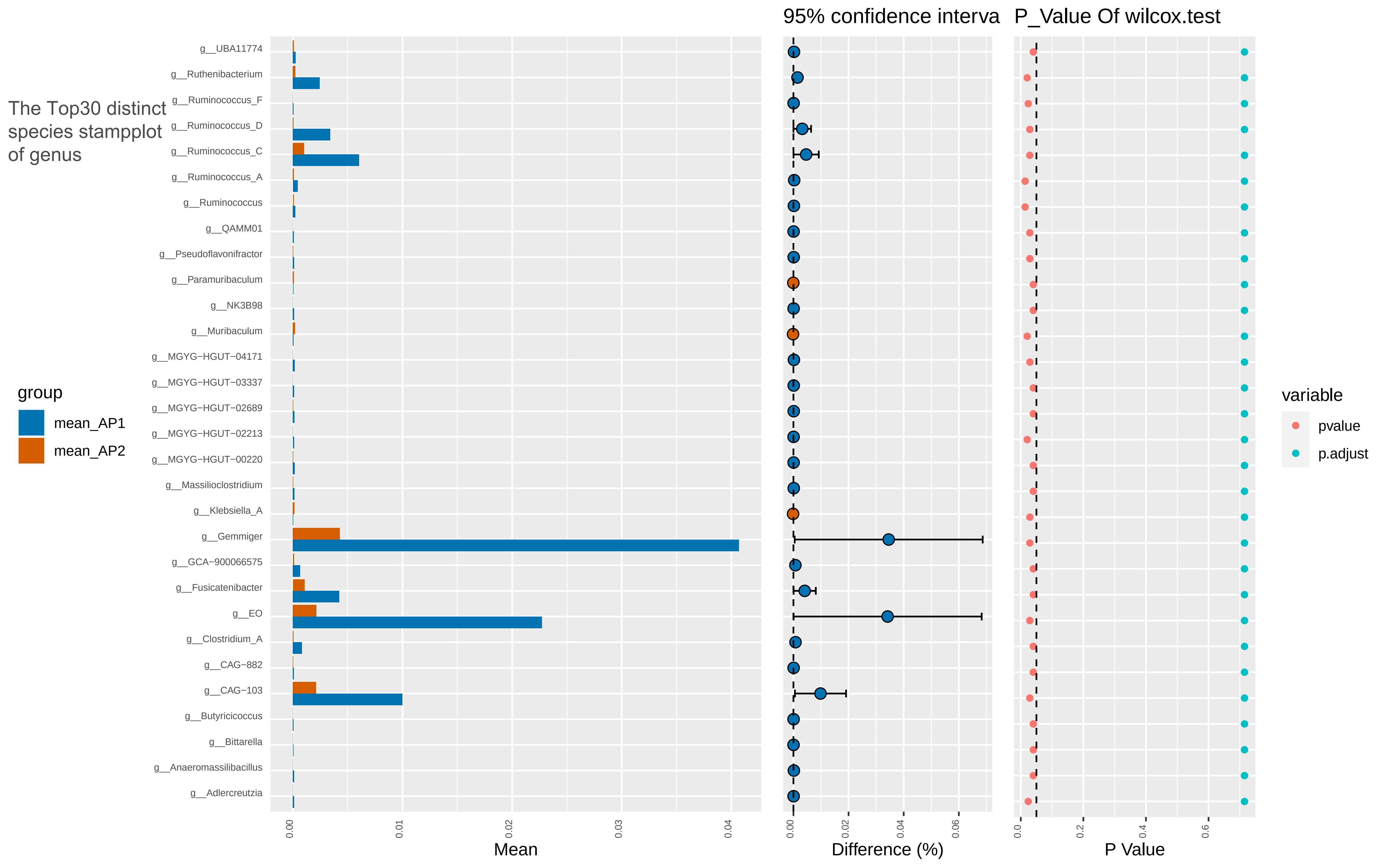

95% confidence interva
P_Value Of wilcox.test
The Top30 distinct species stampplot of genus
g__UBA11774
g__Ruthenibacterium
g__Ruminococcus_F
g__Ruminococcus_D
g__Ruminococcus_C
g__Ruminococcus_A
g__Ruminococcus
g__QAMM01
g__Pseudoflavonifractor
g__Paramuribaculum
g__NK3B98
g__Muribaculum
g__MGYG−HGUT−04171
g__MGYG−HGUT−03337
g__MGYG−HGUT−02689
g__MGYG−HGUT−02213
g__MGYG−HGUT−00220
g__Massilioclostridium
g__Klebsiella_A
g__Gemmiger
g__GCA−900066575
g__Fusicatenibacter
g__EO
g__Clostridium_A
g__CAG−882
g__CAG−103
g__Butyricicoccus
g__Bittarella
g__Anaeromassilibacillus
g__Adlercreutzia
group
mean_AP1
mean_AP2
variable
pvalue
p.adjust
0.00
0.01
0.02
0.03
0.04
Mean
0.00
0.02
0.04
0.06
Difference (%)
0.0
0.2
0.4
0.6
P Value

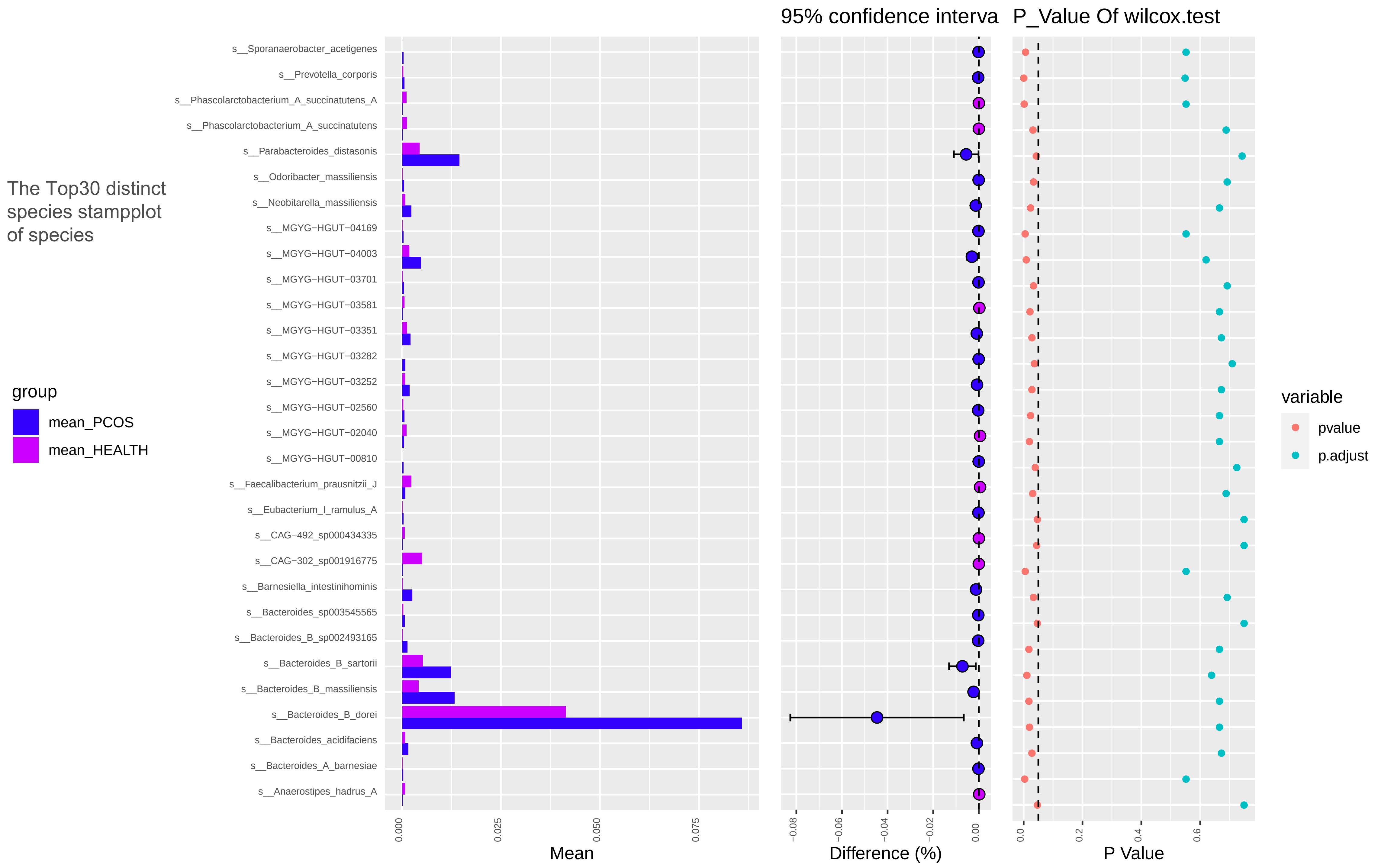

The Top30 distinct species stampplot of species
95% confidence interva
P_Value Of wilcox.test
group
mean_PCOS
mean_HEALTH
variable
pvalue
p.adjust
s__Sporanaerobacter_acetigenes
s__Prevotella_corporis
s__Phascolarctobacterium_A_succinatutens_A
s__Phascolarctobacterium_A_succinatutens
s__Parabacteroides_distasonis
s__Odoribacter_massiliensis
s__Neobitarella_massiliensis
s__MGYG-HGUT-04169
s__MGYG-HGUT-04003
s__MGYG-HGUT-03701
s__MGYG-HGUT-03581
s__MGYG-HGUT-03351
s__MGYG-HGUT-03282
s__MGYG-HGUT-03252
s__MGYG-HGUT-02560
s__MGYG-HGUT-02040
s__MGYG-HGUT-00810
s__Faecalibacterium_prausnitzii_J
s__Eubacterium_I_ramulus_A
s__CAG-492_sp000434335
s__CAG-302_sp001916775
s__Barnesiella_intestinihominis
s__Bacteroides_sp003545565
s__Bacteroides_B_sp002493165
s__Bacteroides_B_sartorii
s__Bacteroides_B_massiliensis
s__Bacteroides_B_dorei
s__Bacteroides_acidifaciens
s__Bacteroides_A_barnesiae
s__Anaerostipes_hadrus_A
0.000
0.025
0.050
0.075
Mean
-0.08
-0.06
-0.04
-0.02
0.00
Difference (%)
0.0
0.2
0.4
0.6
P Value

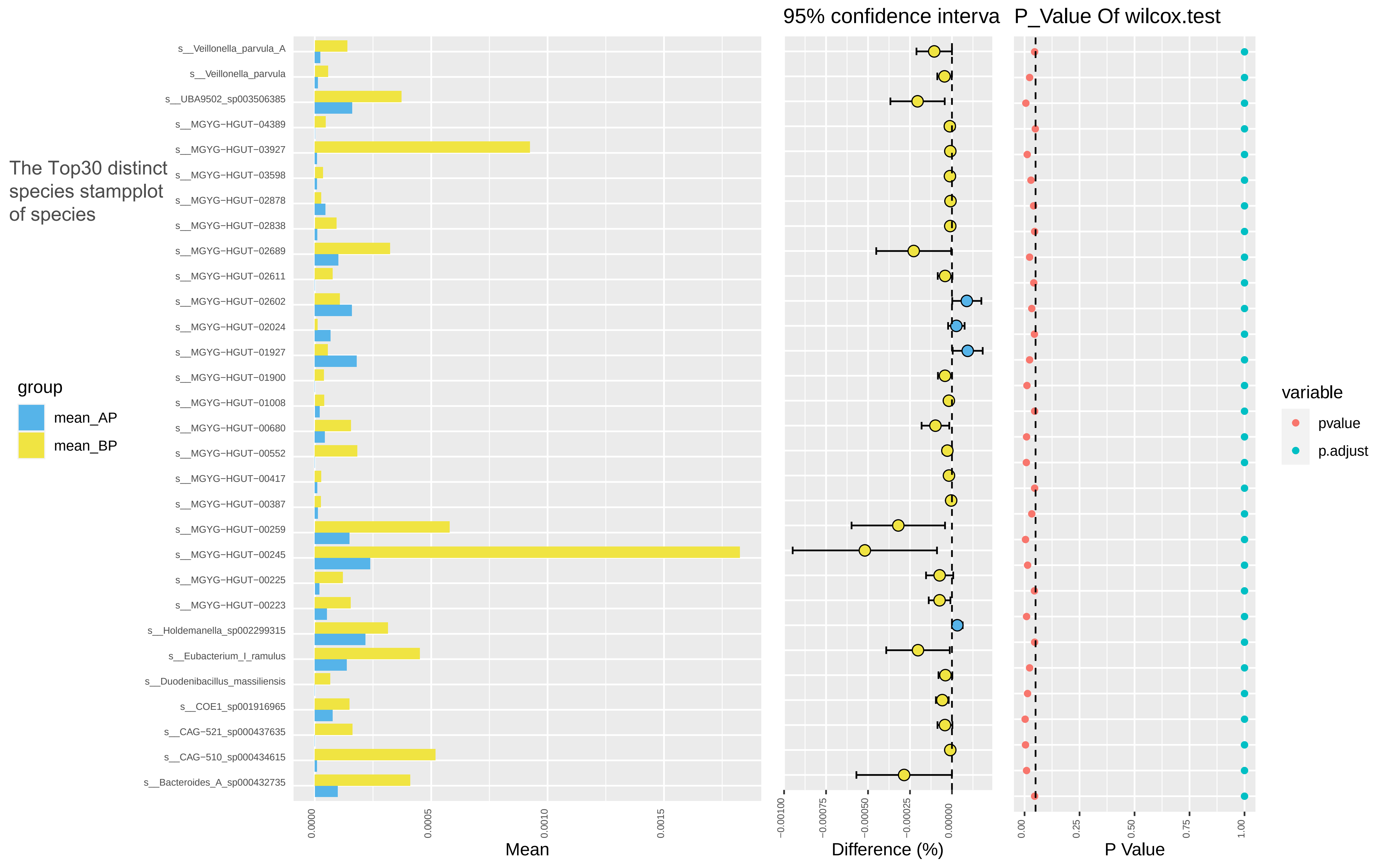

The Top30 distinct species stampplot of species
95% confidence interva
P_Value Of wilcox.test
s__Veillonella_parvula_A
s__Veillonella_parvula
s__UBA9502_sp003506385
s__MGYG-HGUT-04389
s__MGYG-HGUT-03927
s__MGYG-HGUT-03598
s__MGYG-HGUT-02878
s__MGYG-HGUT-02838
s__MGYG-HGUT-02689
s__MGYG-HGUT-02611
s__MGYG-HGUT-02602
s__MGYG-HGUT-02024
s__MGYG-HGUT-01927
s__MGYG-HGUT-01900
s__MGYG-HGUT-01008
s__MGYG-HGUT-00680
s__MGYG-HGUT-00552
s__MGYG-HGUT-00417
s__MGYG-HGUT-00387
s__MGYG-HGUT-00259
s__MGYG-HGUT-00245
s__MGYG-HGUT-00225
s__MGYG-HGUT-00223
s__Holdemanella_sp002299315
s__Eubacterium_I_ramulus
s__Duodenibacillus_massiliensis
s__COE1_sp001916965
s__CAG-521_sp000437635
s__CAG-510_sp000434615
s__Bacteroides_A_sp000432735
group
mean_AP
mean_BP
0.0000
0.0005
0.0010
0.0015
Mean
-0.00100
-0.00075
-0.00050
-0.00025
0.00000
Difference (%)
0.00
0.25
0.50
0.75
1.00
P Value
variable
pvalue
p.adjust

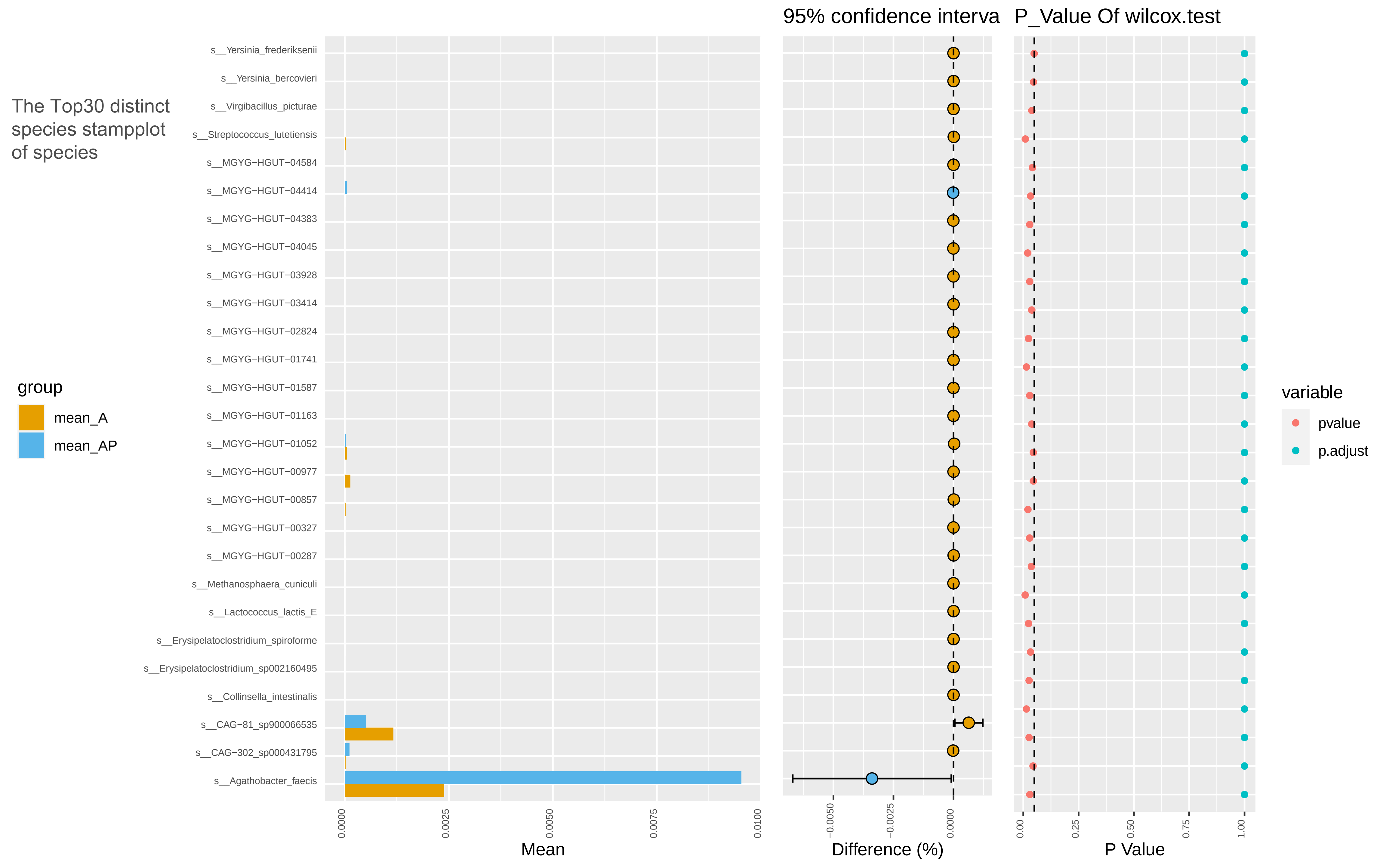

The Top30 distinct species stampplot of species
95% confidence interva
P_Value Of wilcox.test
s__Yersinia_frederiksenii
s__Yersinia_bercovieri
s__Virgibacillus_picturae
s__Streptococcus_lutetiensis
s__MGYG−HGUT−04584
s__MGYG−HGUT−04414
s__MGYG−HGUT−04383
s__MGYG−HGUT−04045
s__MGYG−HGUT−03928
s__MGYG−HGUT−03414
s__MGYG−HGUT−02824
s__MGYG−HGUT−01741
s__MGYG−HGUT−01587
s__MGYG−HGUT−01163
s__MGYG−HGUT−01052
s__MGYG−HGUT−00977
s__MGYG−HGUT−00857
s__MGYG−HGUT−00327
s__MGYG−HGUT−00287
s__Methanosphaera_cuniculi
s__Lactococcus_lactis_E
s__Erysipelatoclostridium_spiroforme
s__Erysipelatoclostridium_sp002160495
s__Collinsella_intestinalis
s__CAG−81_sp900066535
s__CAG−302_sp000431795
s__Agathobacter_faecis
group
mean_A
mean_AP
0.0000
0.0025
0.0050
0.0075
0.0100
Mean
−0.0050
−0.0025
0.0000
Difference (%)
0.00
0.25
0.50
0.75
1.00
P Value
variable
pvalue
p.adjust

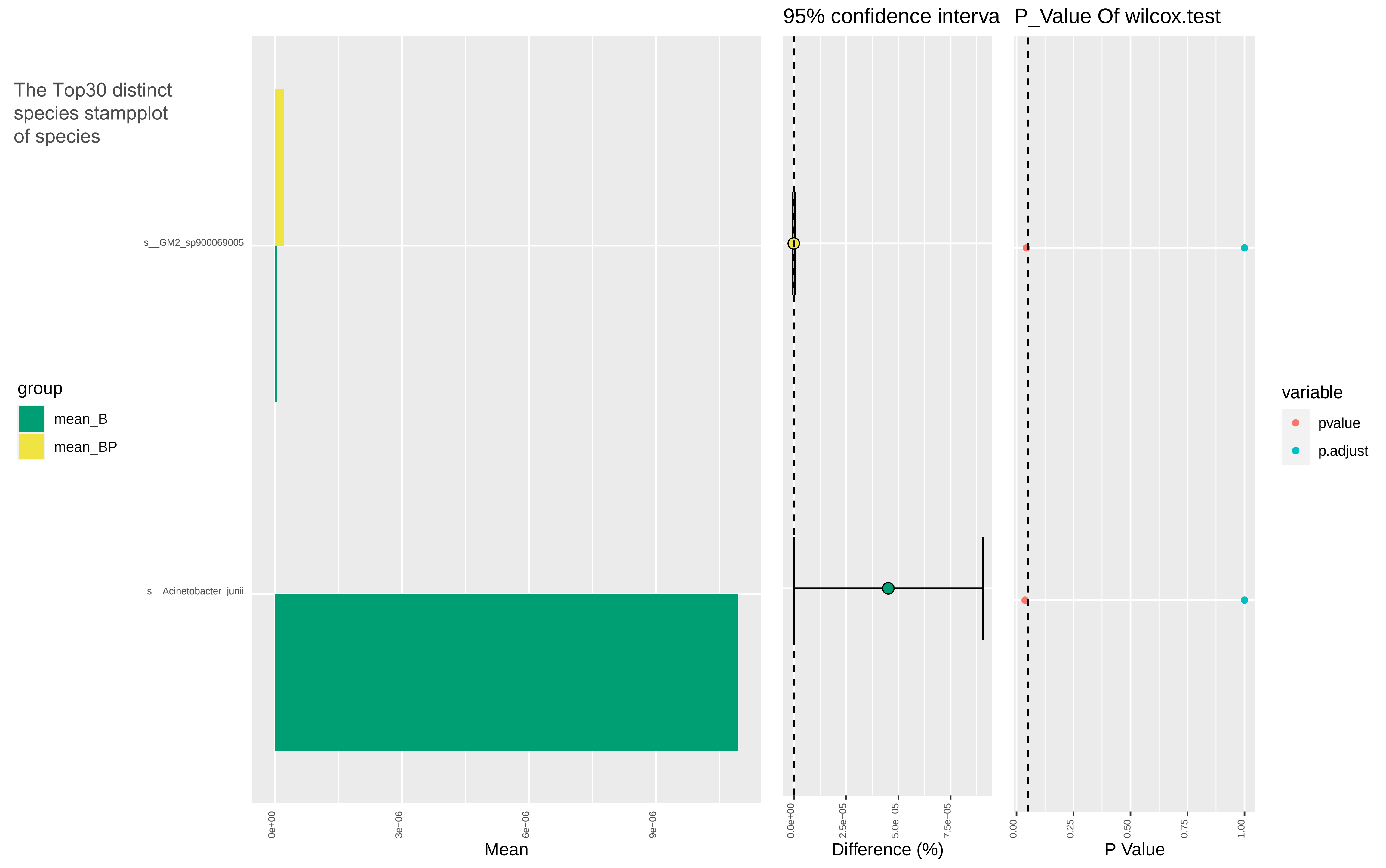

The Top30 distinct species stampplot of species
95% confidence interva
P_Value Of wilcox.test
s__GM2_sp900069005
s__Acinetobacter_junii
group
mean_B
mean_BP
variable
pvalue
p.adjust
0e+00
3e-06
6e-06
9e-06
Mean
0.0e+00
2.5e-05
5.0e-05
7.5e-05
Difference (%)
0.00
0.25
0.50
0.75
1.00
P Value

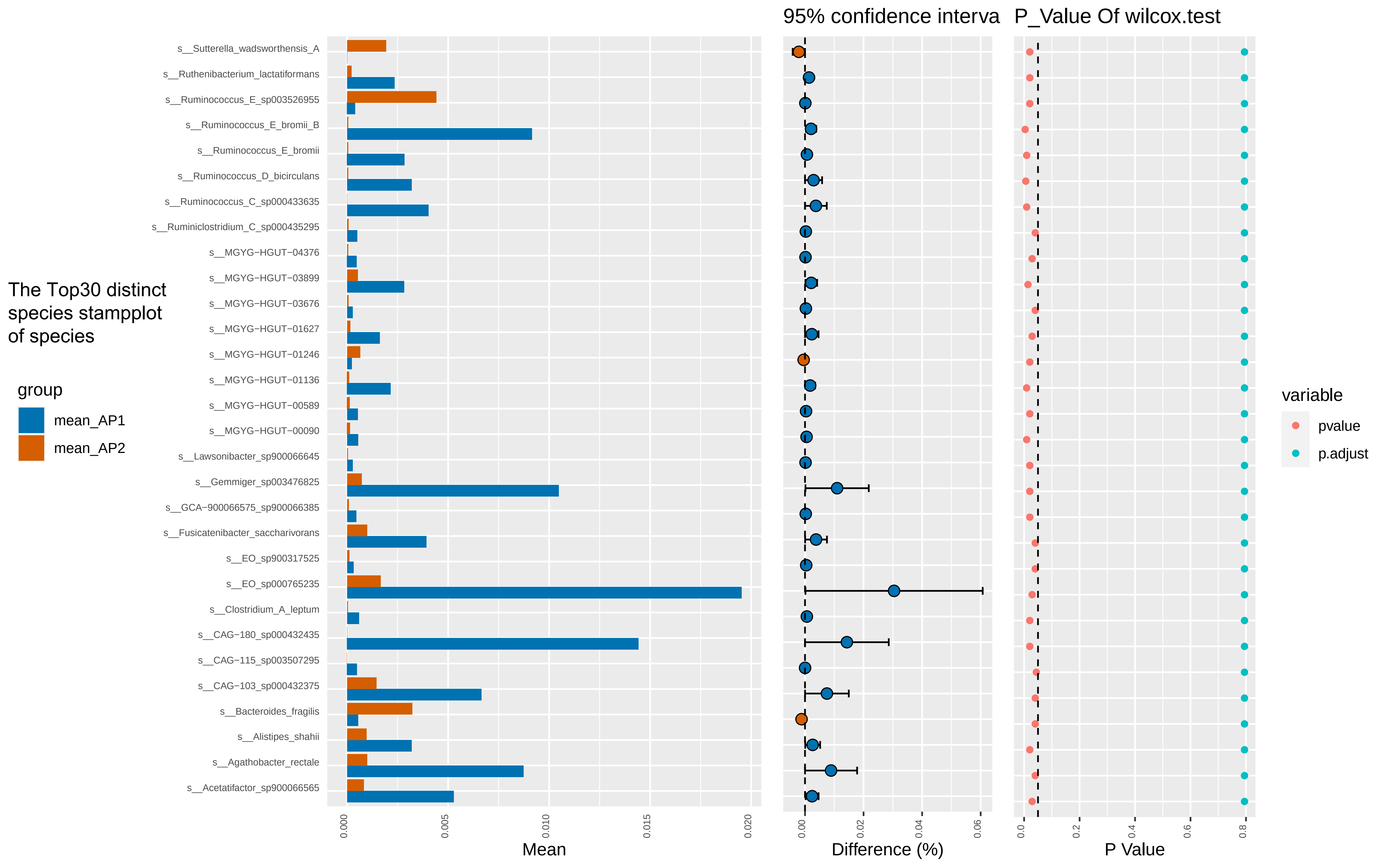
The Top30 distinct species stampplot of species
group
mean_AP1
mean_AP2
95% confidence interva
P_Value Of wilcox.test
s__Sutterella_wadsworthensis_A
s__Ruthenibacterium_lactatiformans
s__Ruminococcus_E_sp003526955
s__Ruminococcus_E_bromii_B
s__Ruminococcus_E_bromii
s__Ruminococcus_D_bicirculans
s__Ruminococcus_C_sp000433635
s__Ruminiclostridium_C_sp000435295
s__MGYG−HGUT−04376
s__MGYG−HGUT−03899
s__MGYG−HGUT−03676
s__MGYG−HGUT−01627
s__MGYG−HGUT−01246
s__MGYG−HGUT−01136
s__MGYG−HGUT−00589
s__MGYG−HGUT−00090
s__Lawsonibacter_sp900066645
s__Gemmiger_sp003476825
s__GCA−900066575_sp900066385
s__Fusicatenibacter_saccharivorans
s__EO_sp900317525
s__EO_sp000765235
s__Clostridium_A_leptum
s__CAG−180_sp000432435
s__CAG−115_sp003507295
s__CAG−103_sp000432375
s__Bacteroides_fragilis
s__Alistipes_shahii
s__Agathobacter_rectale
s__Acetatifactor_sp900066565
0.000
0.005
0.010
0.015
0.020
Mean
0.00
0.02
0.04
0.06
Difference (%)
0.0
0.2
0.4
0.6
0.8
P Value
variable
pvalue
p.adjust

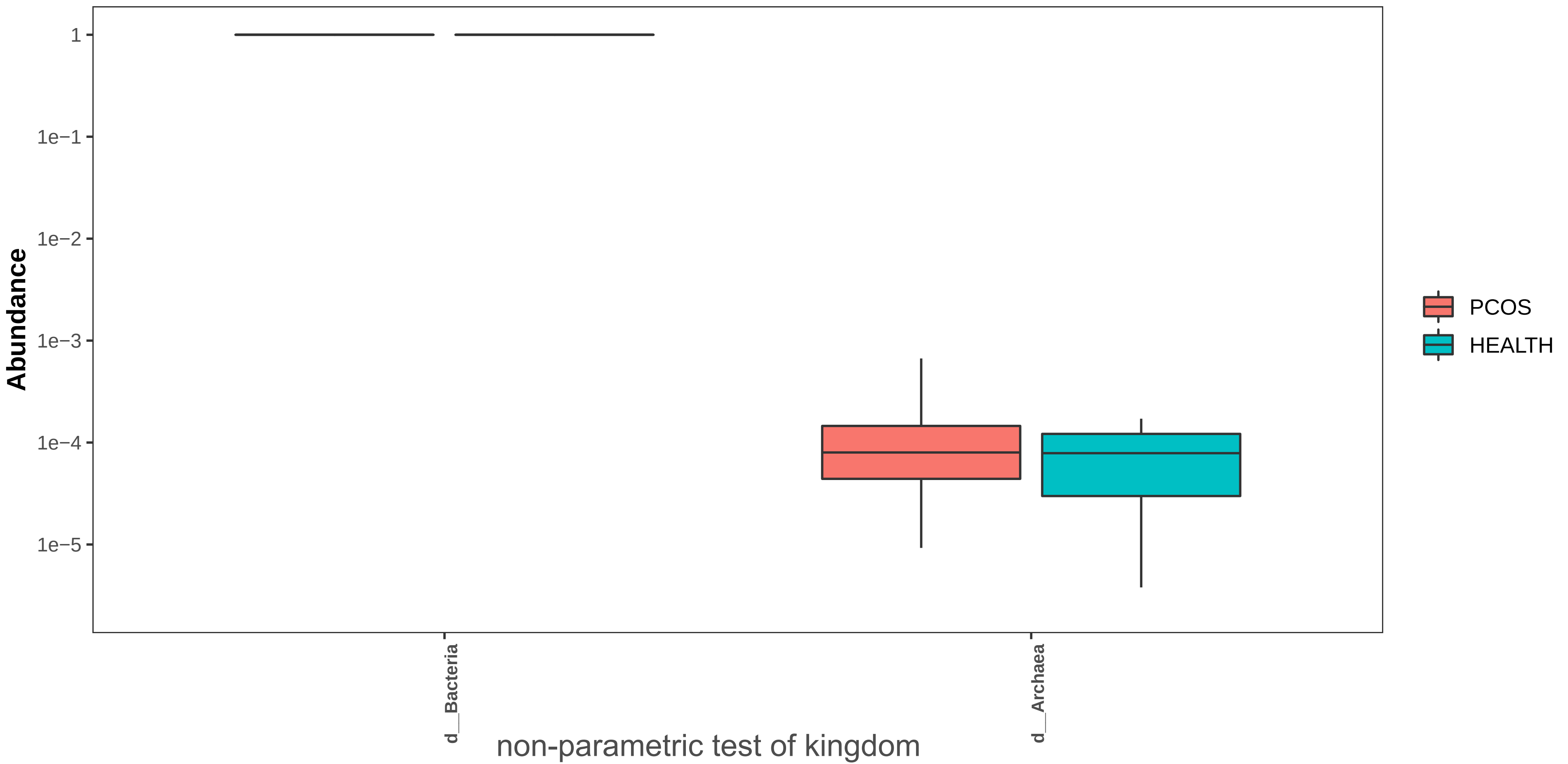

1
1e−1
1e−2
1e−3
1e−4
1e−5
Abundance
d__Bacteria
d__Archaea
non-parametric test of kingdom
PCOS
HEALTH

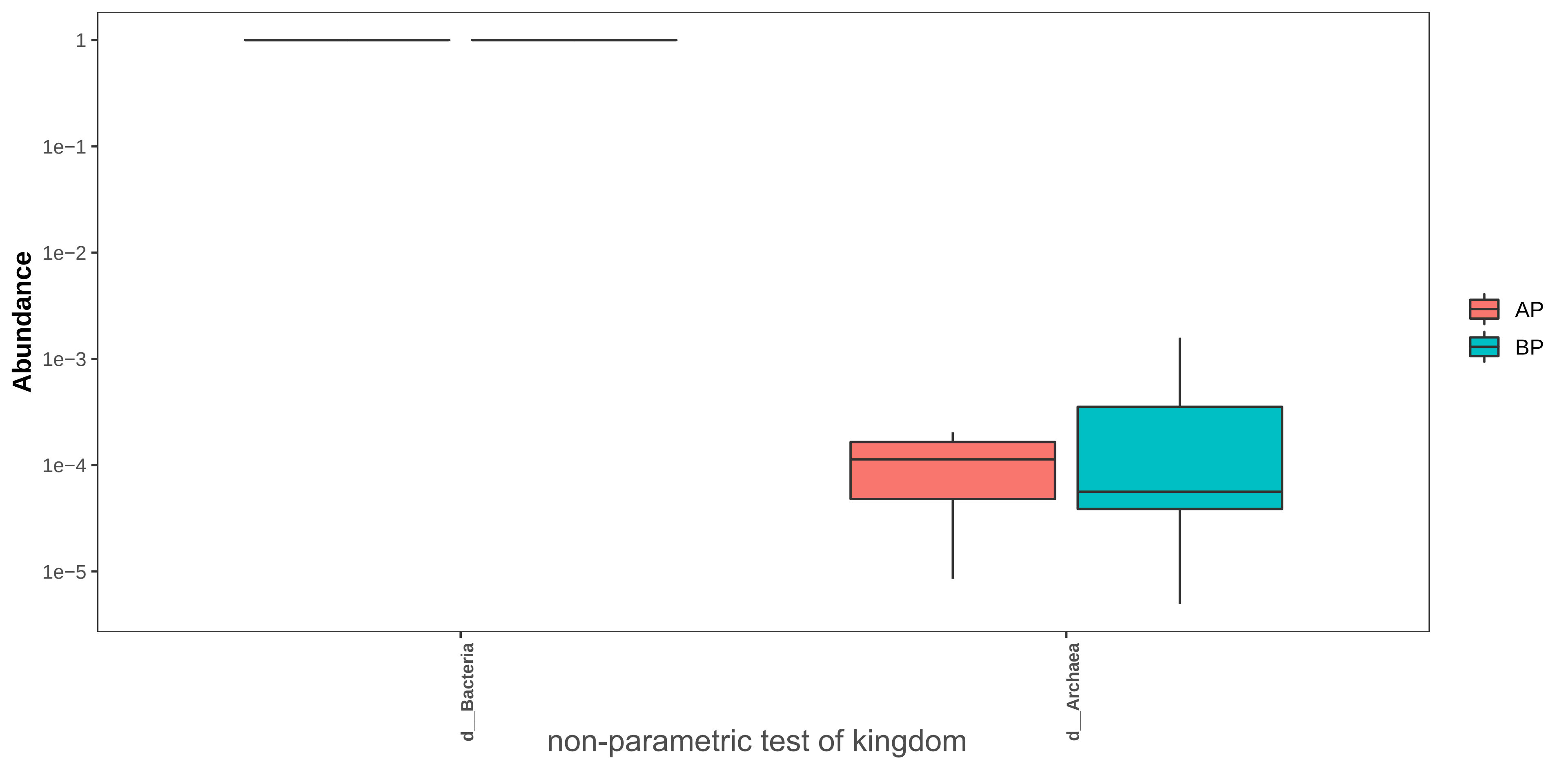

Abundance
1
1e−1
1e−2
1e−3
1e−4
1e−5
d__Bacteria
d__Archaea
non-parametric test of kingdom
AP
BP

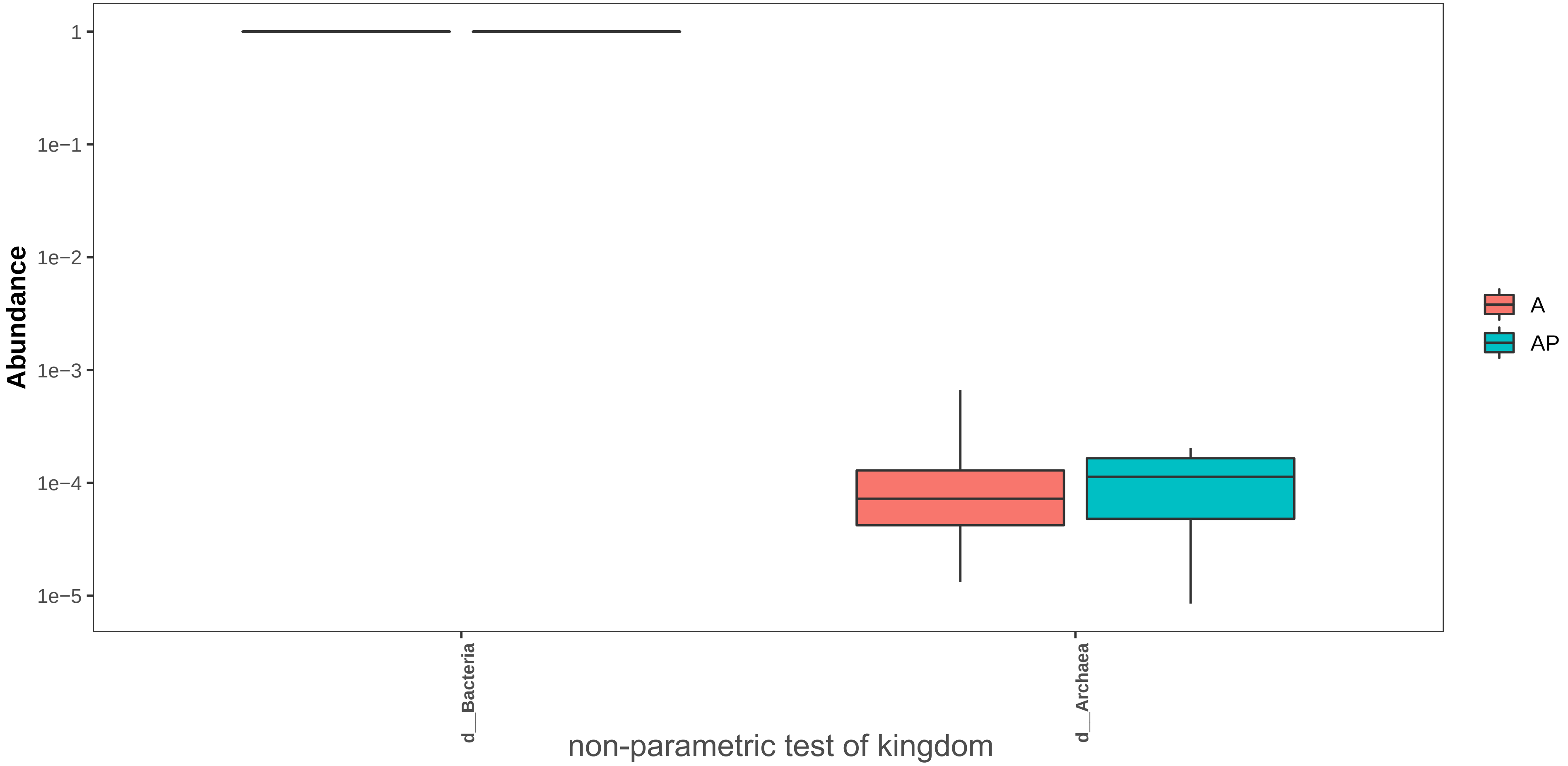

Abundance
1
1e−1
1e−2
1e−3
1e−4
1e−5
d__Bacteria
d__Archaea
non-parametric test of kingdom
A
AP

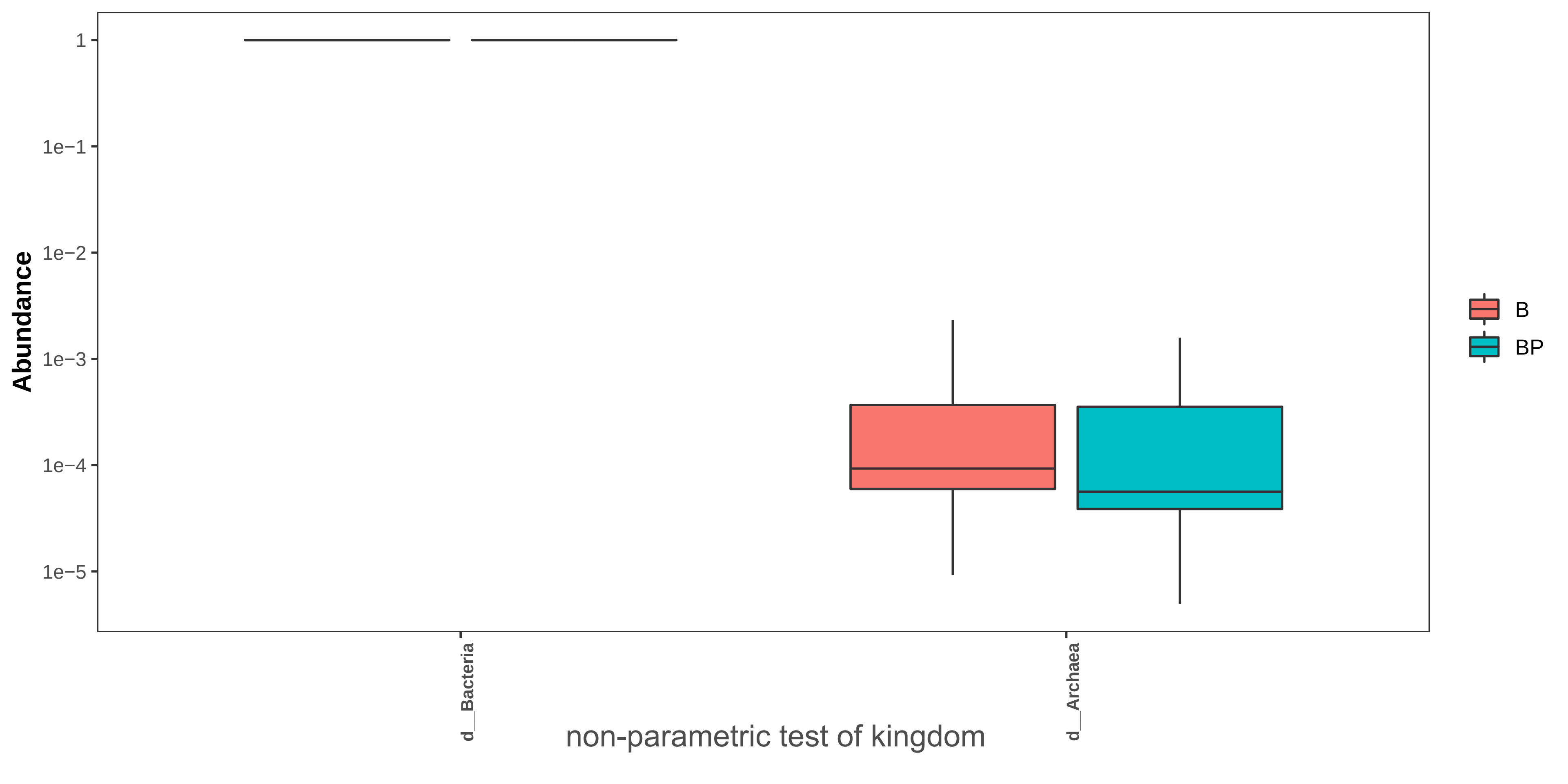

Abundance
1
1e−1
1e−2
1e−3
1e−4
1e−5
d__Bacteria
d__Archaea
non-parametric test of kingdom
B
BP

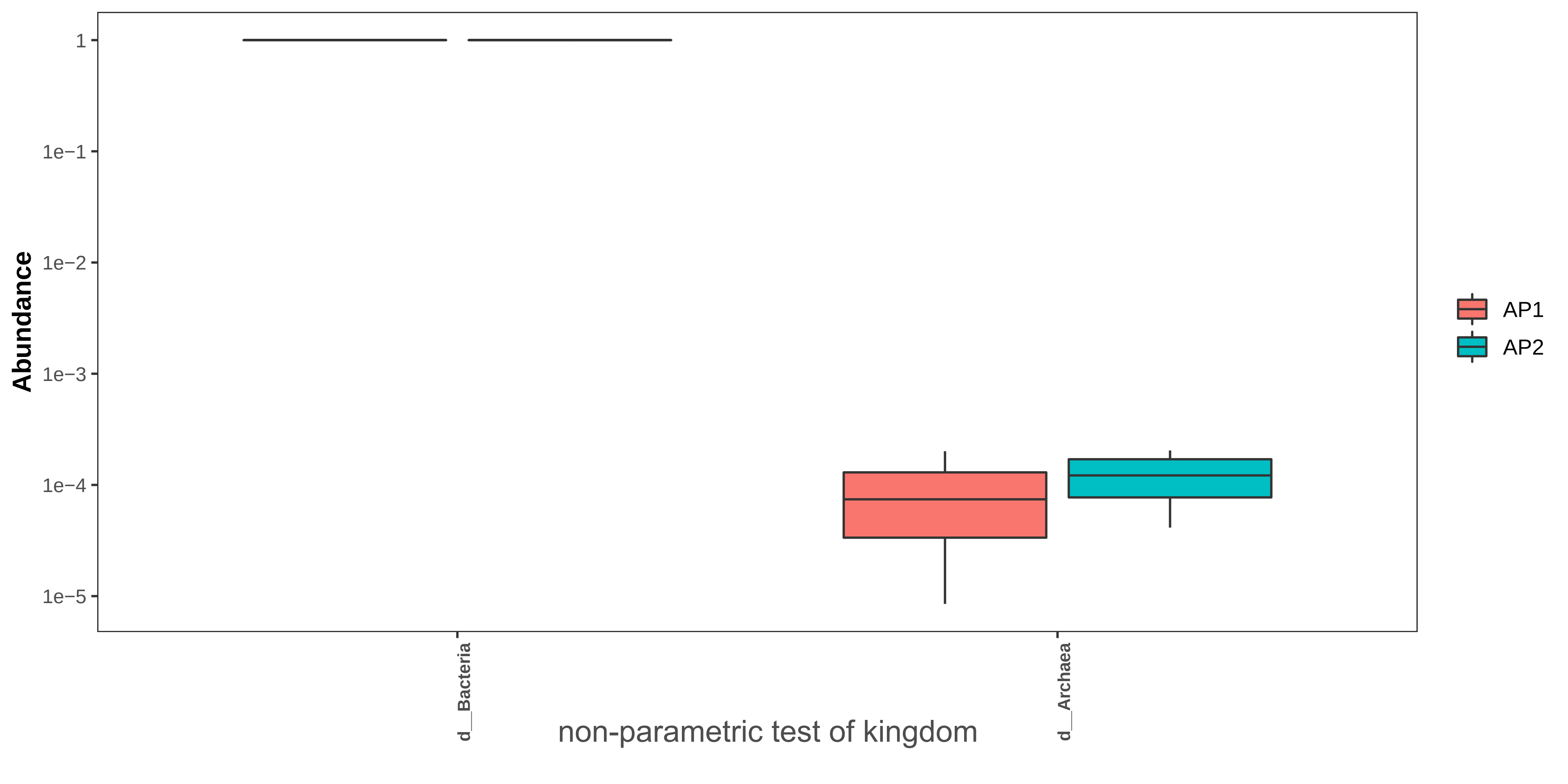

Abundance
1
1e−1
1e−2
1e−3
1e−4
1e−5
d__Bacteria
d__Archaea
non-parametric test of kingdom
AP1
AP2

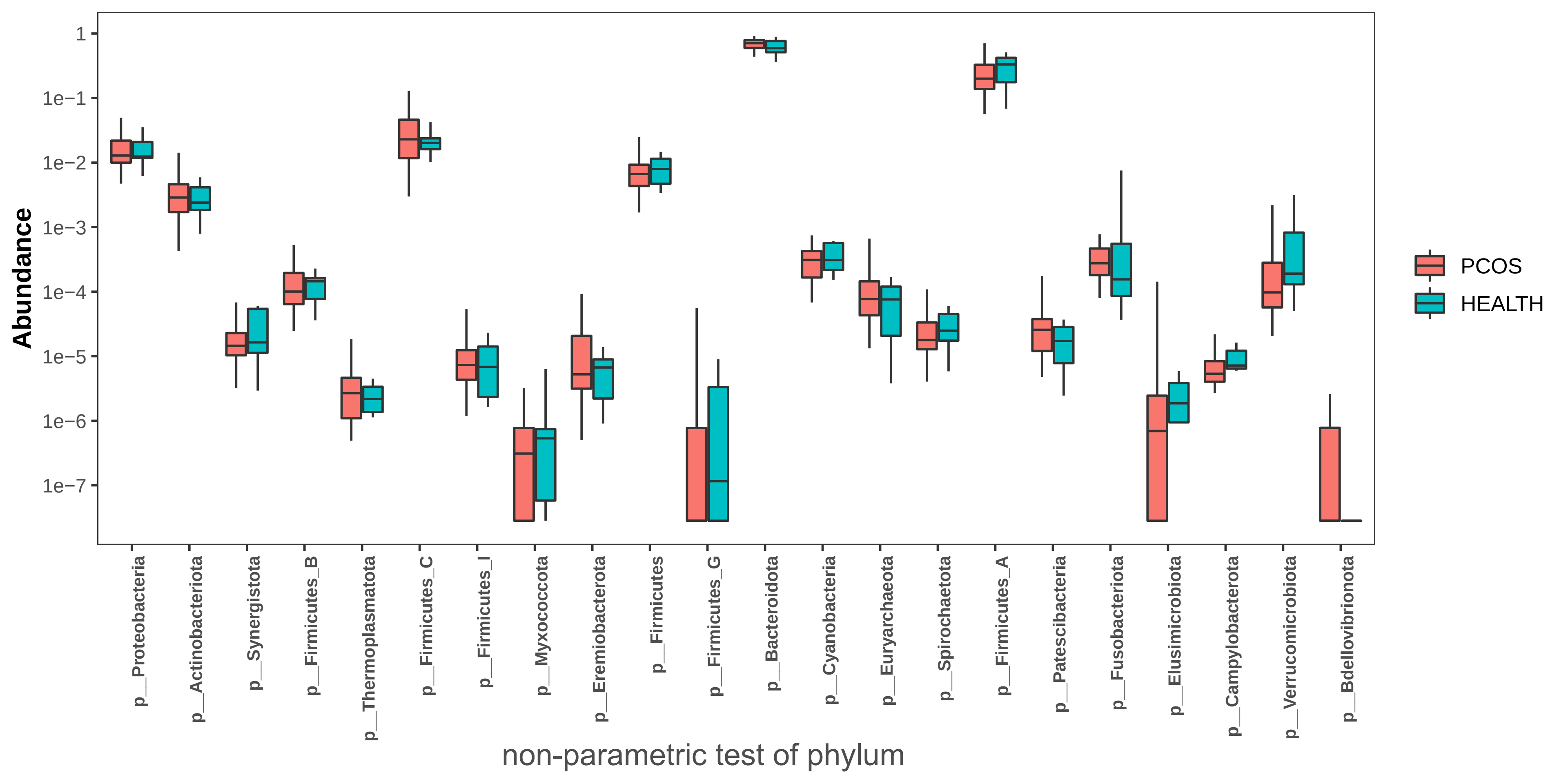

Abundance
1
1e−1
1e−2
1e−3
1e−4
1e−5
1e−6
1e−7
PCOS
HEALTH
p__Proteobacteria
p__Actinobacteriota
p__Synergistota
p__Firmicutes_B
p__Thermoplasmatota
p__Firmicutes_C
p__Firmicutes_I
p__Myxococcota
p__Eremiobacterota
p__Firmicutes
p__Firmicutes_G
p__Bacteroidota
p__Cyanobacteria
p__Euryarchaeota
p__Spirochaetota
p__Firmicutes_A
p__Patescibacteria
p__Fusobacteriota
p__Elusimicrobiota
p__Campylobacterota
p__Verrucomicrobiota
p__Bdellovibrionota
non-parametric test of phylum

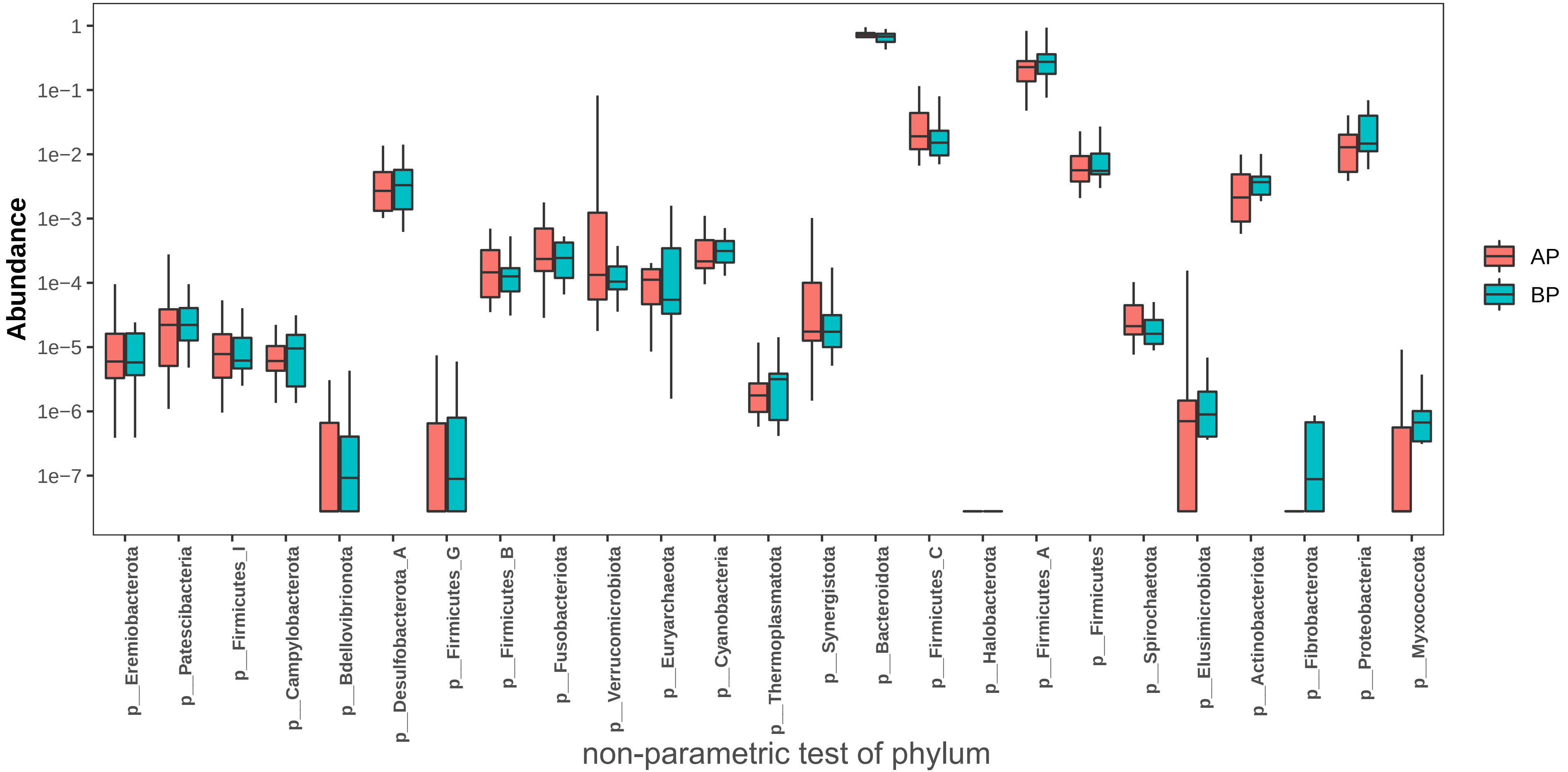
Abundance
1
1e−1
1e−2
1e−3
1e−4
1e−5
1e−6
1e−7
p__Eremiobacterota
p__Patescibacteria
p__Firmicutes_I
p__Campylobacterota
p__Bdellovibrionota
p__Desulfobacterota_A
p__Firmicutes_G
p__Firmicutes_B
p__Fusobacteriota
p__Verrucomicrobiota
p__Euryarchaeota
p__Cyanobacteria
p__Thermoplasmatota
p__Synergistota
p__Bacteroidota
p__Firmicutes_C
p__Halobacterota
p__Firmicutes_A
p__Firmicutes
p__Spirochaetota
p__Elusimicrobiota
p__Actinobacteriota
p__Fibrobacterota
p__Proteobacteria
p__Myxococcota
non-parametric test of phylum
AP
BP

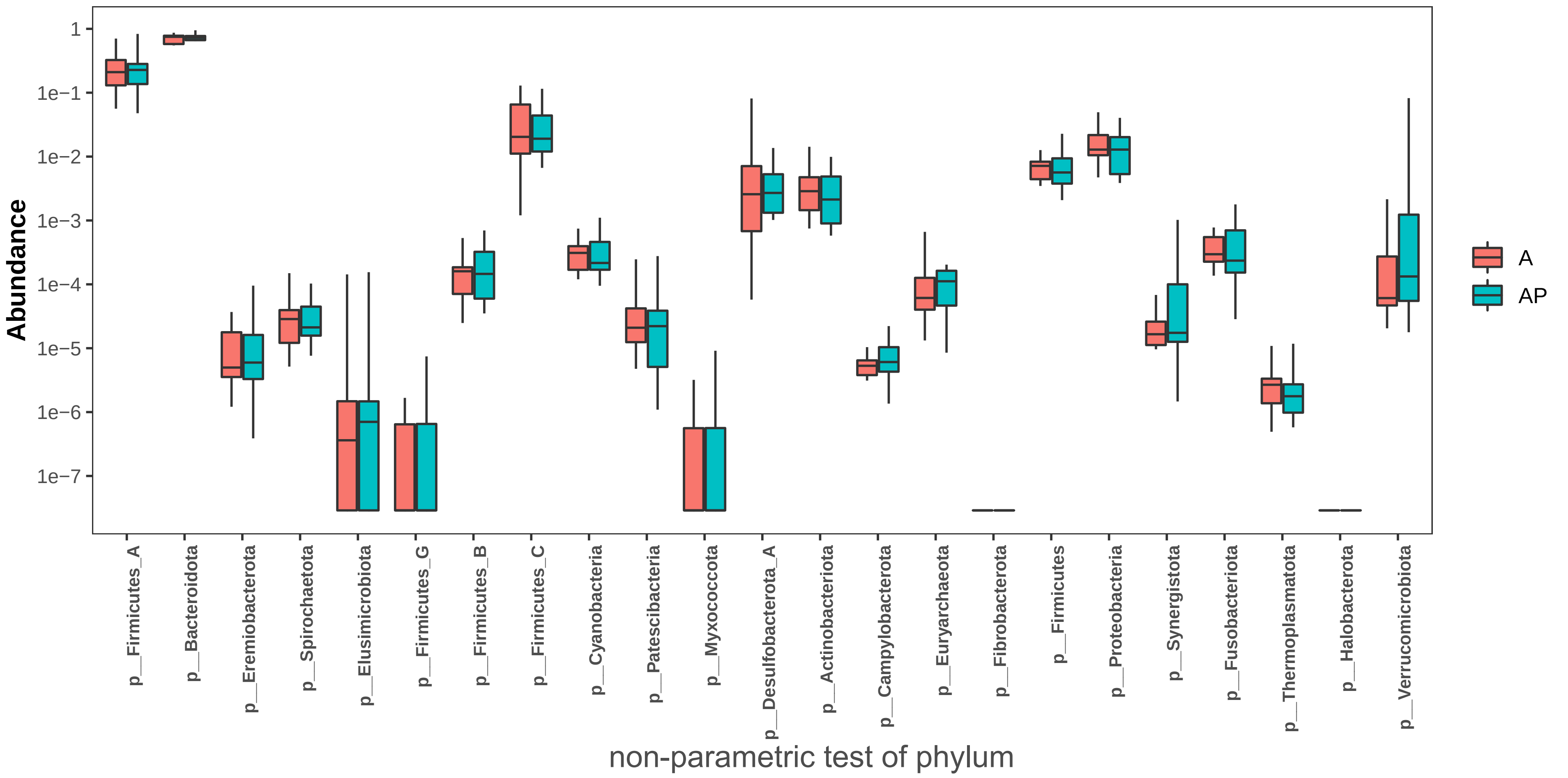

Abundance
1
1e−1
1e−2
1e−3
1e−4
1e−5
1e−6
1e−7
p__Firmicutes_A
p__Bacteroidota
p__Eremiobacterota
p__Spirochaetota
p__Elusimicrobiota
p__Firmicutes_G
p__Firmicutes_B
p__Firmicutes_C
p__Cyanobacteria
p__Patescibacteria
p__Myxococcota
p__Desulfobacterota_A
p__Actinobacteriota
p__Campylobacterota
p__Euryarchaeota
p__Fibrobacterota
p__Firmicutes
p__Proteobacteria
p__Synergistota
p__Fusobacteriota
p__Thermoplasmatota
p__Halobacterota
p__Verrucomicrobiota
non-parametric test of phylum
A
AP

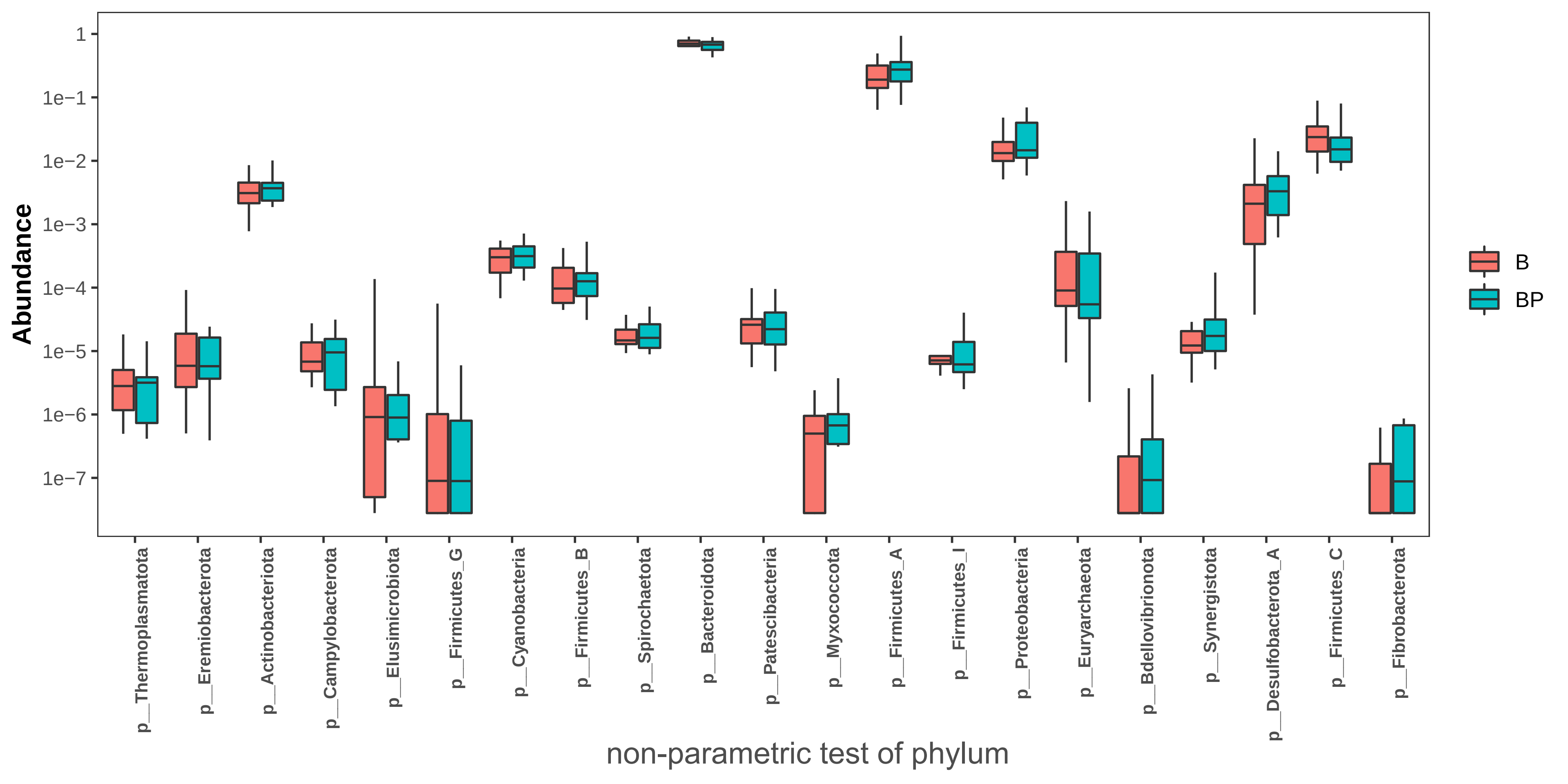

Abundance
1
1e−1
1e−2
1e−3
1e−4
1e−5
1e−6
1e−7
p__Thermoplasmatota
p__Eremiobacterota
p__Actinobacteriota
p__Campylobacterota
p__Elusimicrobiota
p__Firmicutes_G
p__Cyanobacteria
p__Firmicutes_B
p__Spirochaetota
p__Bacteroidota
p__Patescibacteria
p__Myxococcota
p__Firmicutes_A
p__Firmicutes_I
p__Proteobacteria
p__Euryarchaeota
p__Bdellovibrionota
p__Synergistota
p__Desulfobacterota_A
p__Firmicutes_C
p__Fibrobacterota
B
BP
non-parametric test of phylum

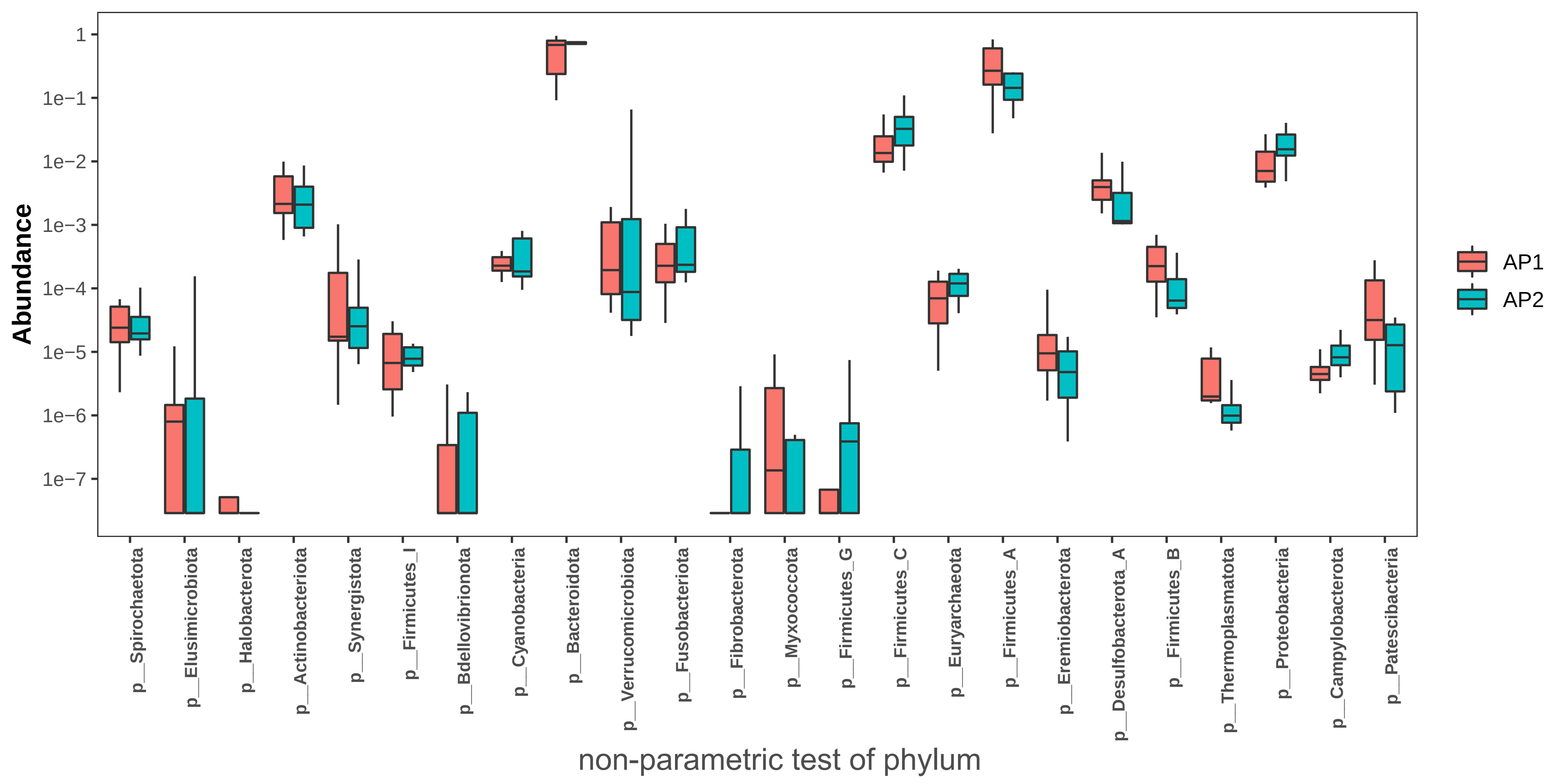

Abundance
1
1e−1
1e−2
1e−3
1e−4
1e−5
1e−6
1e−7
AP1
AP2
p__Spirochaetota
p__Elusimicrobiota
p__Halobacterota
p__Actinobacteriota
p__Synergistota
p__Firmicutes_I
p__Bdellovibrionota
p__Cyanobacteria
p__Bacteroidota
p__Verrucomicrobiota
p__Fusobacteriota
p__Fibrobacterota
p__Myxococcota
p__Firmicutes_G
p__Firmicutes_C
p__Euryarchaeota
p__Firmicutes_A
p__Eremiobacterota
p__Desulfobacterota_A
p__Firmicutes_B
p__Thermoplasmatota
p__Proteobacteria
p__Campylobacterota
p__Patescibacteria
non-parametric test of phylum

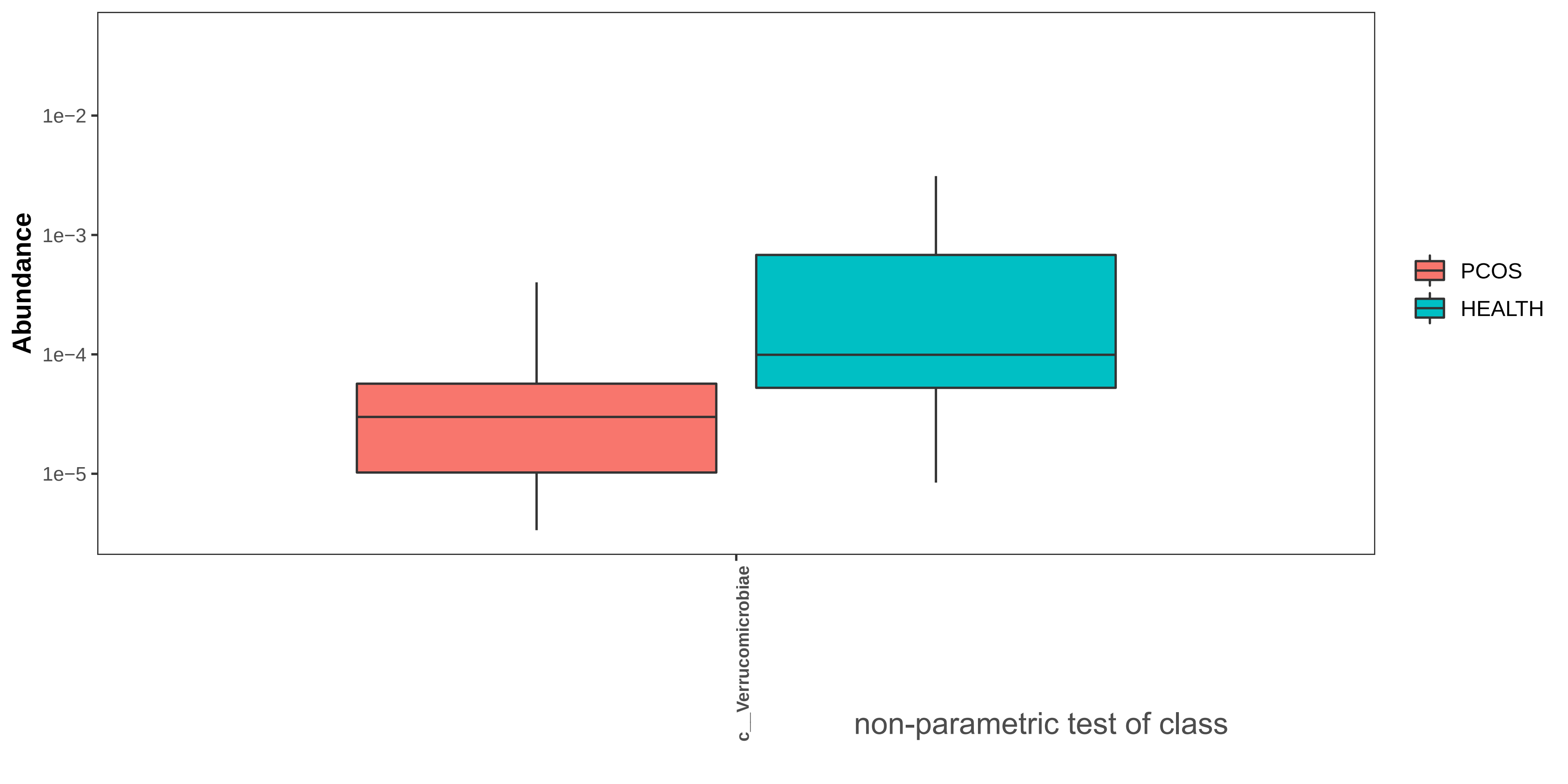

Abundance
1e−2
1e−3
1e−4
1e−5
PCOS
HEALTH
c__Verrucomicrobiae
non-parametric test of class

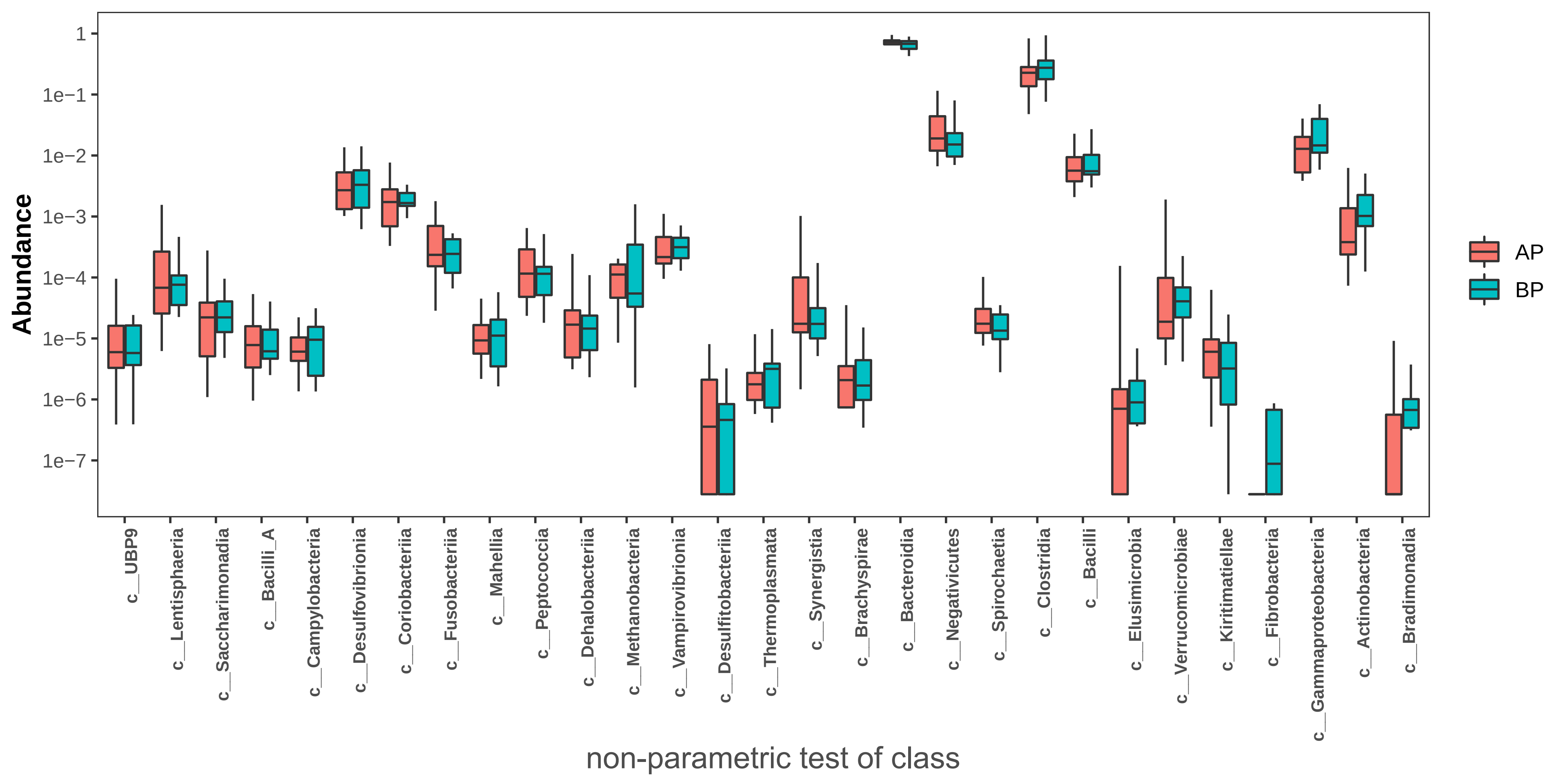

Abundance
1
1e−1
1e−2
1e−3
1e−4
1e−5
1e−6
1e−7
c__UBP9
c__Lentisphaeria
c__Saccharimonadia
c__Bacilli_A
c__Campylobacteria
c__Desulfovibrionia
c__Coriobacteriia
c__Fusobacteriia
c__Mahellia
c__Peptococcia
c__Dehalobacteriia
c__Methanobacteria
c__Vampirovibrionia
c__Desulfitobacteriia
c__Thermoplasmata
c__Synergistia
c__Brachyspirae
c__Bacteroidia
c__Negativicutes
c__Spirochaetia
c__Clostridia
c__Bacilli
c__Elusimicrobia
c__Verrucomicrobiae
c__Kiritimatiellae
c__Fibrobacteria
c__Gammaproteobacteria
c__Actinobacteria
c__Bradimonadia
AP
BP
non-parametric test of class

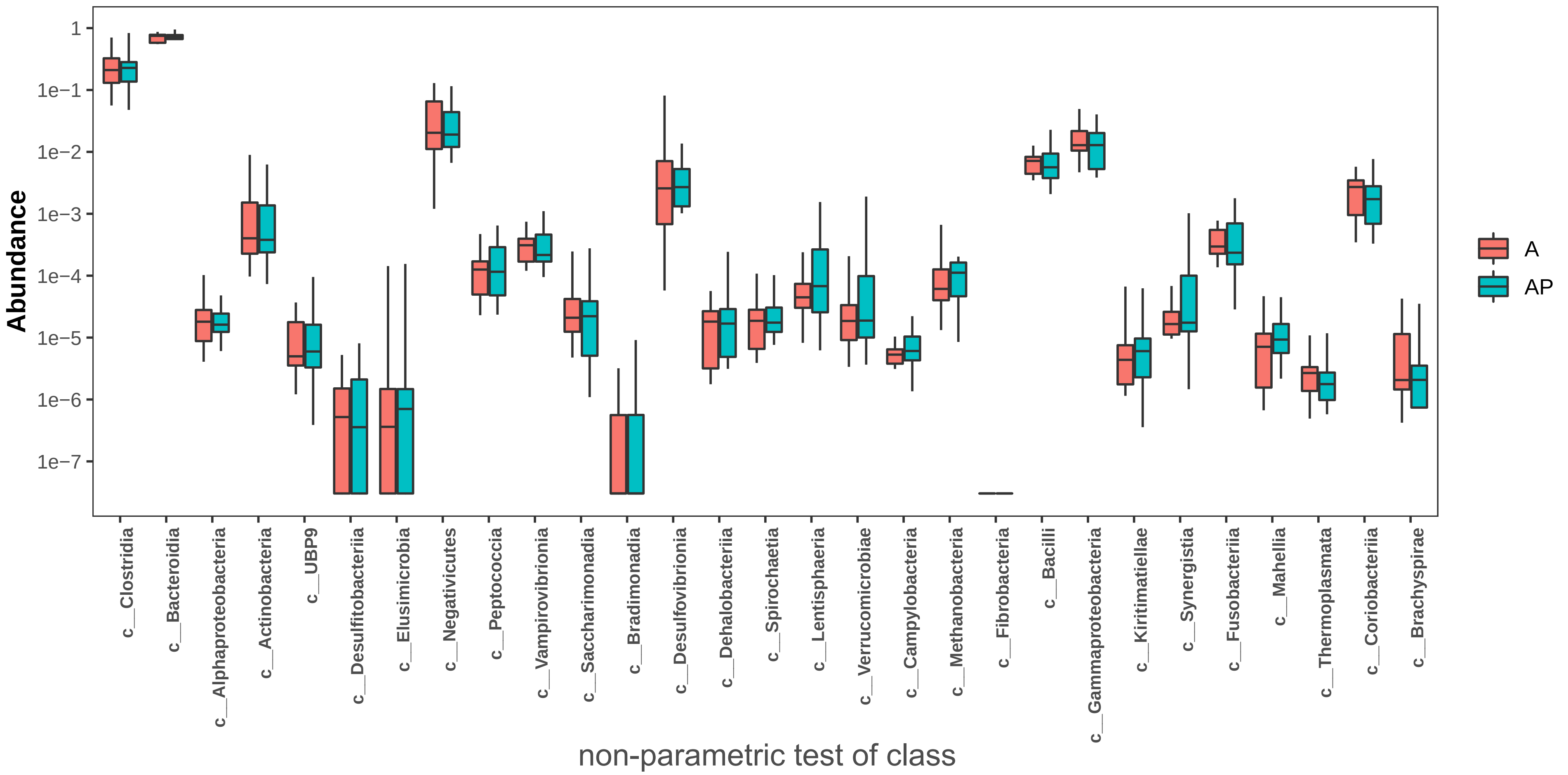
Abundance
1
1e−1
1e−2
1e−3
1e−4
1e−5
1e−6
1e−7
c__Clostridia
c__Bacteroidia
c__Alphaproteobacteria
c__Actinobacteria
c__UBP9
c__Desulfitobacteriia
c__Elusimicrobia
c__Negativicutes
c__Peptococcia
c__Vampirovibrionia
c__Saccharimonadia
c__Bradimonadia
c__Desulfovibrionia
c__Dehalobacteriia
c__Spirochaetia
c__Lentisphaeria
c__Verrucomicrobiae
c__Campylobacteria
c__Methanobacteria
c__Fibrobacteria
c__Bacilli
c__Gammaproteobacteria
c__Kiritimatiellae
c__Synergistia
c__Fusobacteriia
c__Mahellia
c__Thermoplasmata
c__Coriobacteriia
c__Brachyspirae
non-parametric test of class
A
AP

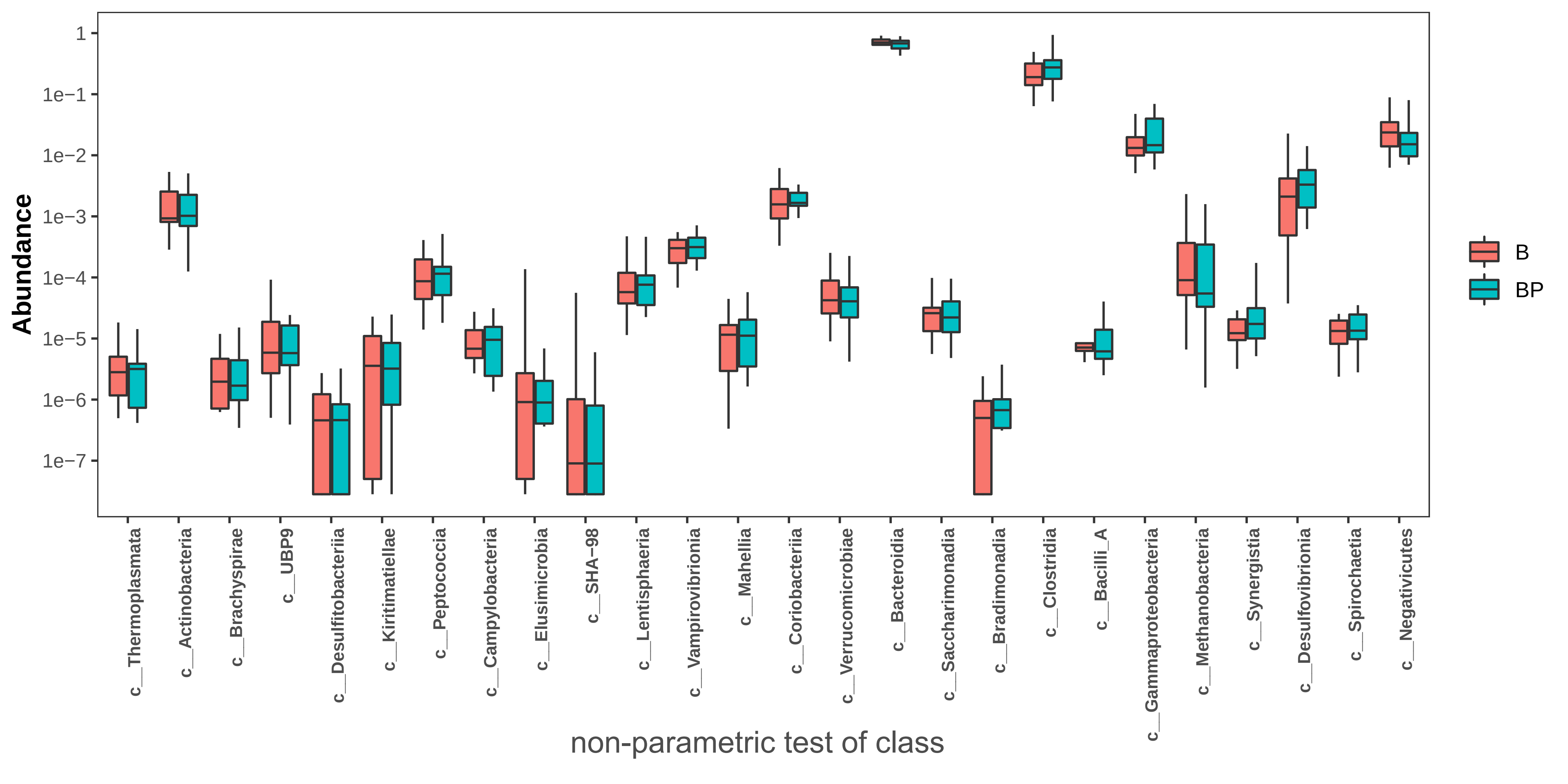

Abundance
1
1e−1
1e−2
1e−3
1e−4
1e−5
1e−6
1e−7
B
BP
c__Thermoplasmata
c__Actinobacteria
c__Brachyspirae
c__UBP9
c__Desulfitobacteriia
c__Kiritimatiellae
c__Peptococcia
c__Campylobacteria
c__Elusimicrobia
c__SHA−98
c__Lentisphaeria
c__Vampirovibrionia
c__Mahellia
c__Coriobacteriia
c__Verrucomicrobiae
c__Bacteroidia
c__Saccharimonadia
c__Bradimonadia
c__Clostridia
c__Bacilli_A
c__Gammaproteobacteria
c__Methanobacteria
c__Synergistia
c__Desulfovibrionia
c__Spirochaetia
c__Negativicutes
non-parametric test of class

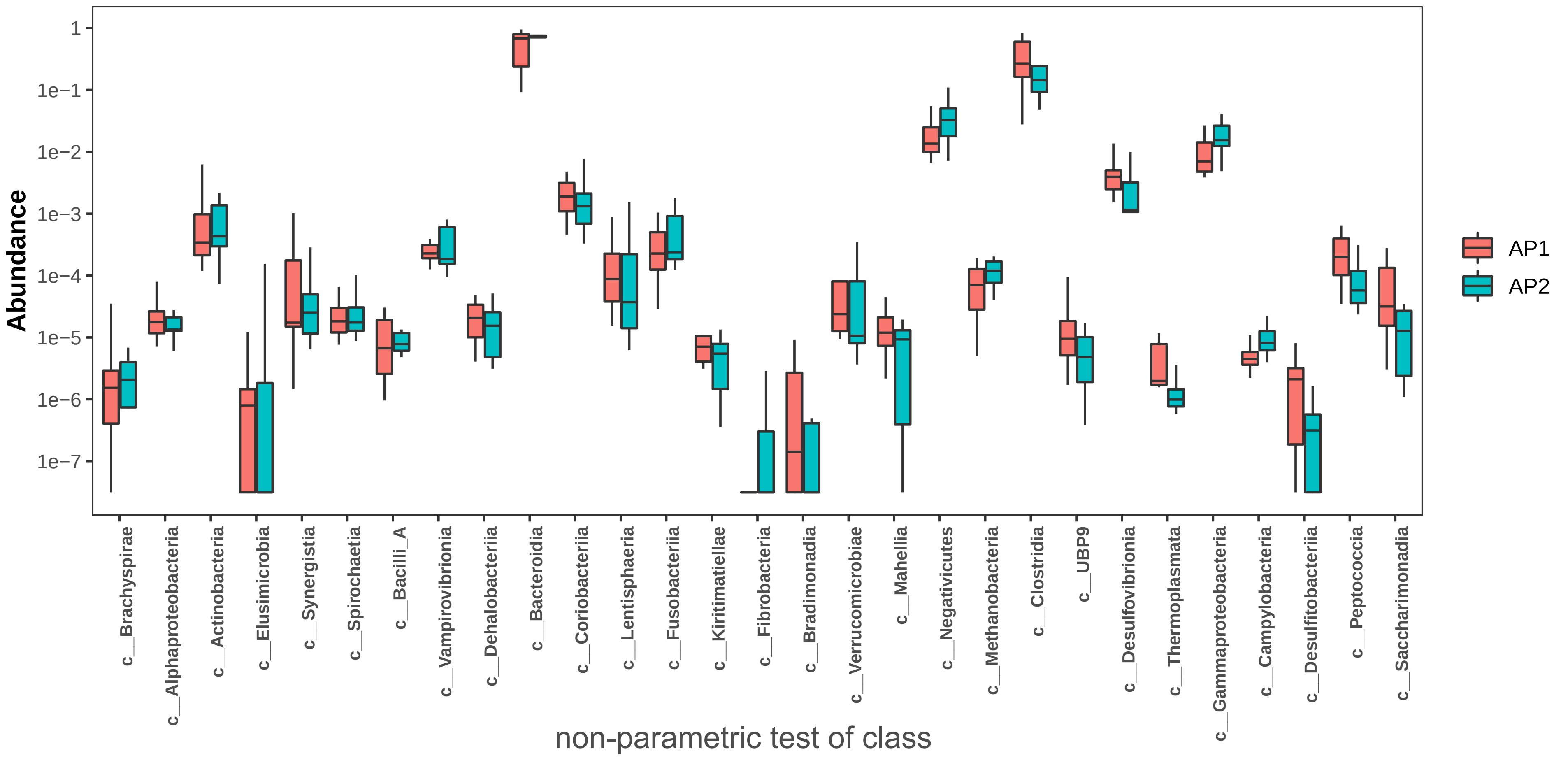

Abundance
1
1e−1
1e−2
1e−3
1e−4
1e−5
1e−6
1e−7
AP1
AP2
c__Brachyspirae
c__Alphaproteobacteria
c__Actinobacteria
c__Elusimicrobia
c__Synergistia
c__Spirochaetia
c__Bacilli_A
c__Vampirovibrionia
c__Dehalobacteriia
c__Bacteroidia
c__Coriobacteriia
c__Lentisphaeria
c__Fusobacteriia
c__Kiritimatiellae
c__Fibrobacteria
c__Bradimonadia
c__Verrucomicrobiae
c__Mahellia
c__Negativicutes
c__Methanobacteria
c__Clostridia
c__UBP9
c__Desulfovibrionia
c__Thermoplasmata
c__Gammaproteobacteria
c__Campylobacteria
c__Desulfitobacteriia
c__Peptococcia
c__Saccharimonadia
non-parametric test of class

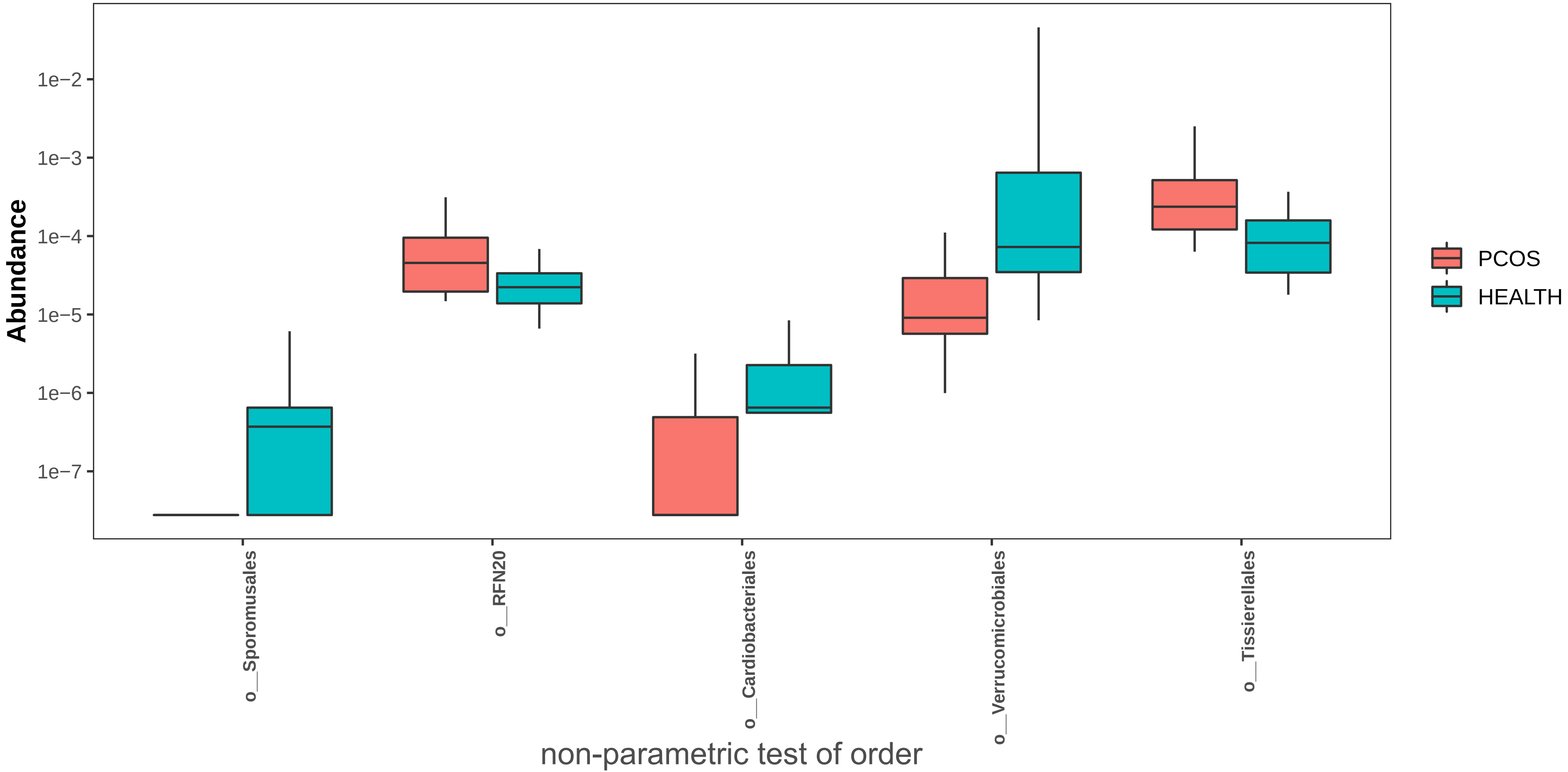

Abundance
1e−2
1e−3
1e−4
1e−5
1e−6
1e−7
o__Sporomusales
o__RFN20
o__Cardiobacteriales
o__Verrucomicrobiales
o__Tissierellales
non-parametric test of order
PCOS
HEALTH

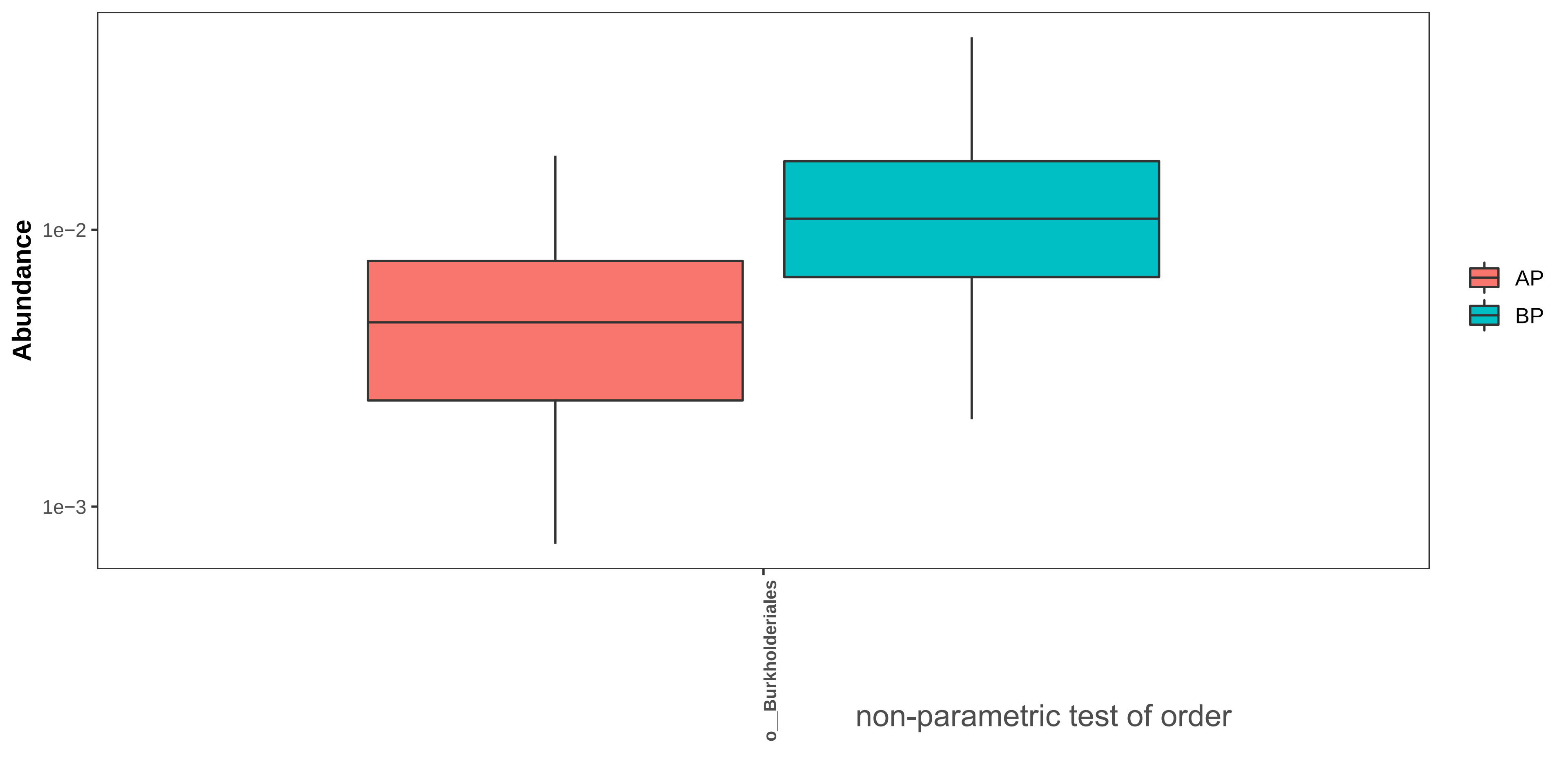
Abundance
1e−2
1e−3
o__Burkholderiales
non-parametric test of order
AP
BP

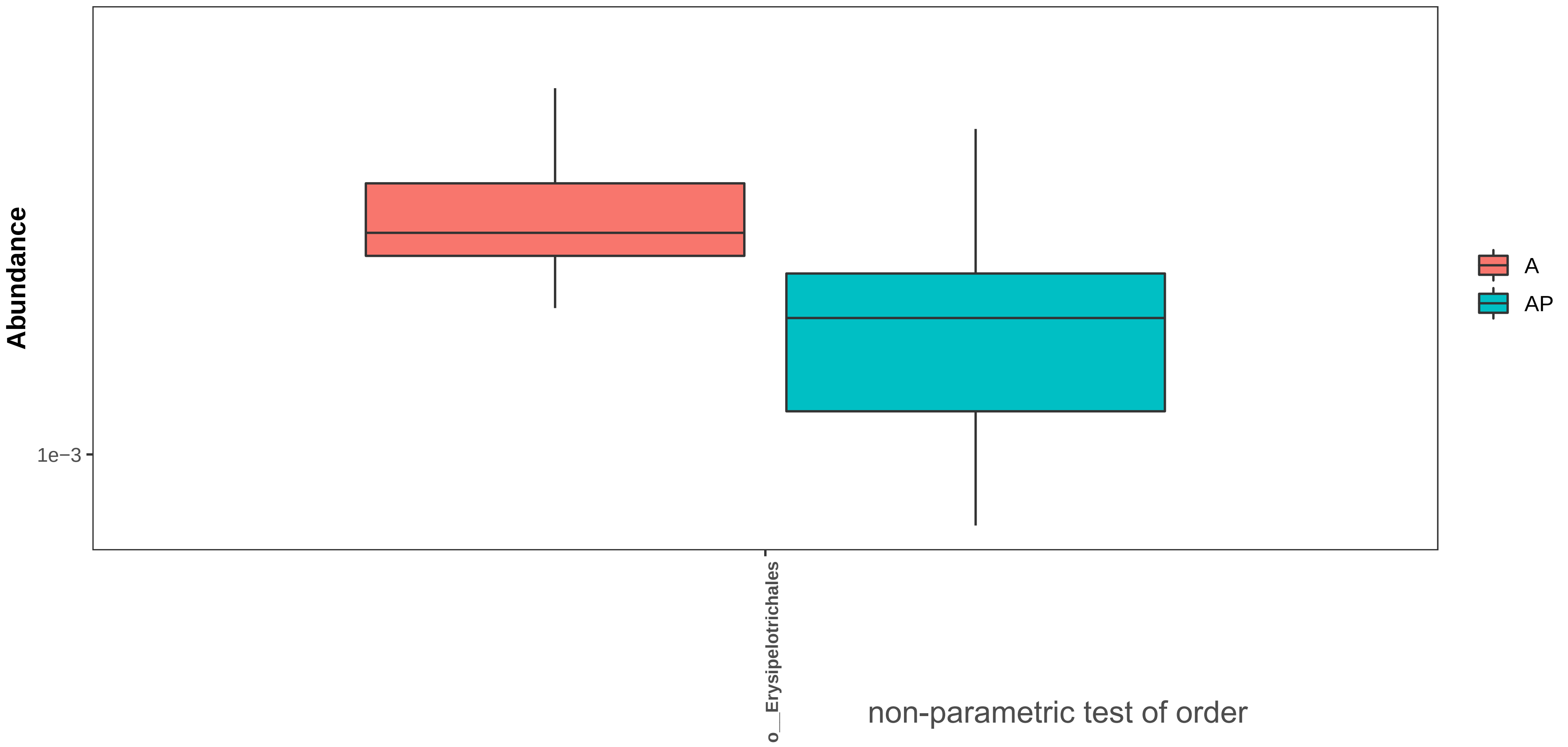

non-parametric test of order

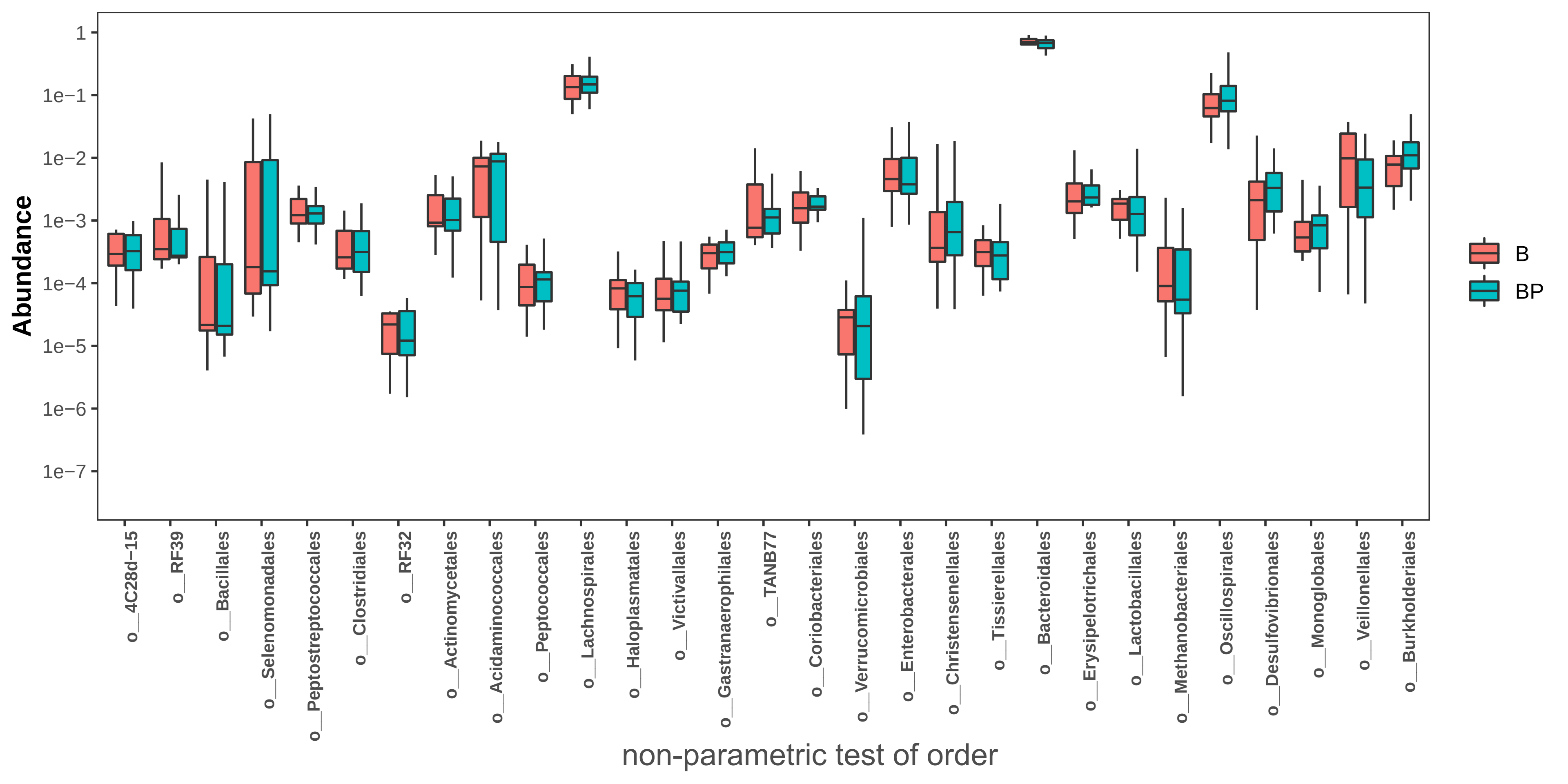

Abundance
1
1e−1
1e−2
1e−3
1e−4
1e−5
1e−6
1e−7
o__4C28d−15
o__RF39
o__Bacillales
o__Selenomonadales
o__Peptostreptococcales
o__Clostridiales
o__RF32
o__Actinomycetales
o__Acidaminococcales
o__Peptococcales
o__Lachnospirales
o__Haloplasmatales
o__Victivallales
o__Gastranaerophilales
o__TANB77
o__Coriobacteriales
o__Verrucomicrobiales
o__Enterobacterales
o__Christensenellales
o__Tissierellales
o__Bacteroidales
o__Erysipelotrichales
o__Lactobacillales
o__Methanobacteriales
o__Oscillospirales
o__Desulfovibrionales
o__Monoglobales
o__Veillonellales
o__Burkholderiales
B
BP
non-parametric test of order

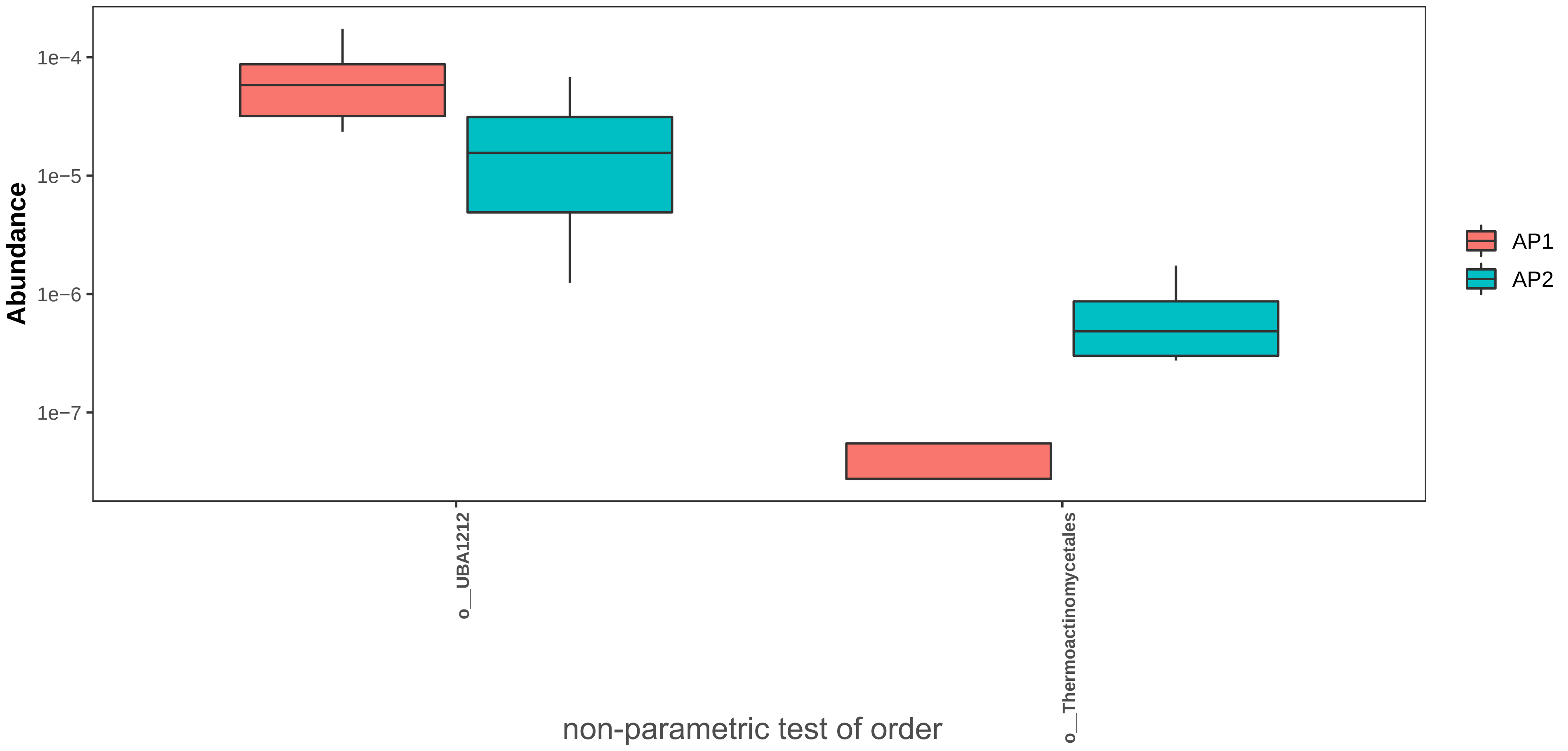

Abundance
1e−4
1e−5
1e−6
1e−7
o__UBA1212
o__Thermoactinomycetales
non-parametric test of order
AP1
AP2

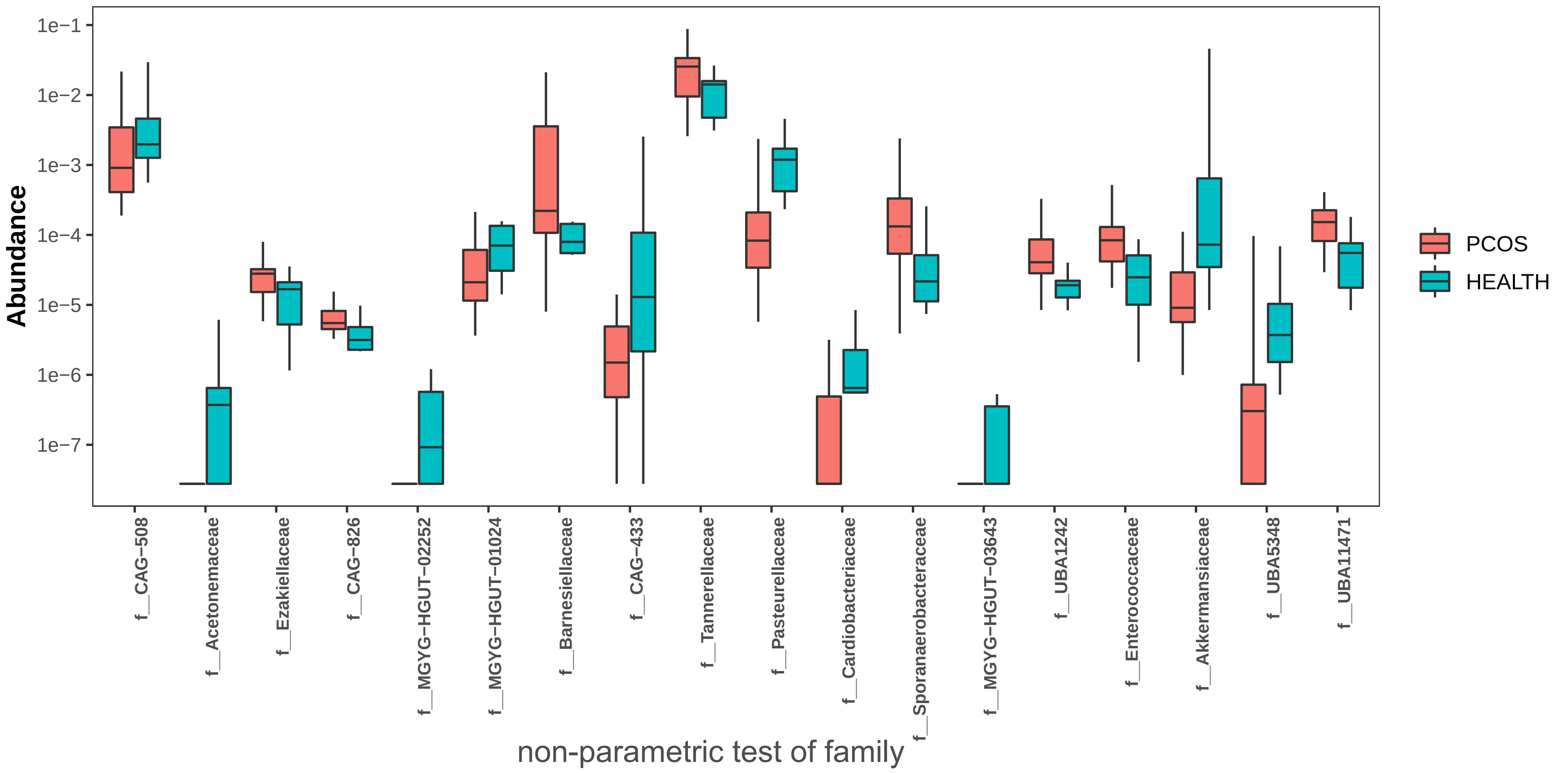

Abundance
1e−1
1e−2
1e−3
1e−4
1e−5
1e−6
1e−7
f__CAG−508
f__Acetonemaceae
f__Ezakiellaceae
f__CAG−826
f__MGYG−HGUT−02252
f__MGYG−HGUT−01024
f__Barnesiellaceae
f__CAG−433
f__Tannerellaceae
f__Pasteurellaceae
f__Cardiobacteriaceae
f__Sporanaerobacteraceae
f__MGYG−HGUT−03643
f__UBA1242
f__Enterococcaceae
f__Akkermansiaceae
f__UBA5348
f__UBA11471
PCOS
HEALTH
non-parametric test of family

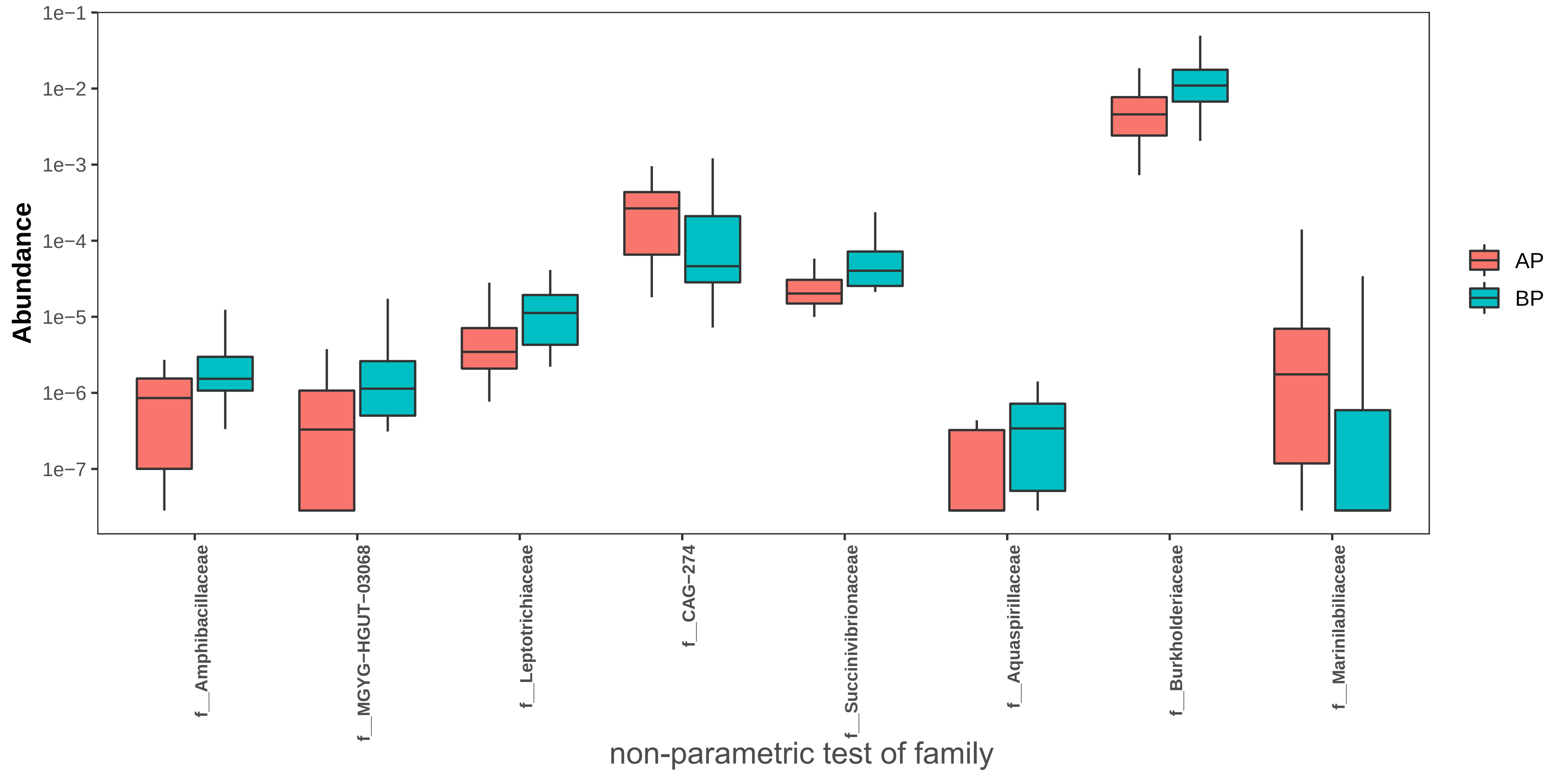

1e−1
1e−2
1e−3
1e−4
1e−5
1e−6
1e−7
Abundance
AP
BP
f__Amphibacillaceae
f__MGYG−HGUT−03068
f__Leptotrichiaceae
f__CAG−274
f__Succinivibrionaceae
f__Aquaspirillaceae
f__Burkholderiaceae
f__Marinilabiliaceae
non-parametric test of family

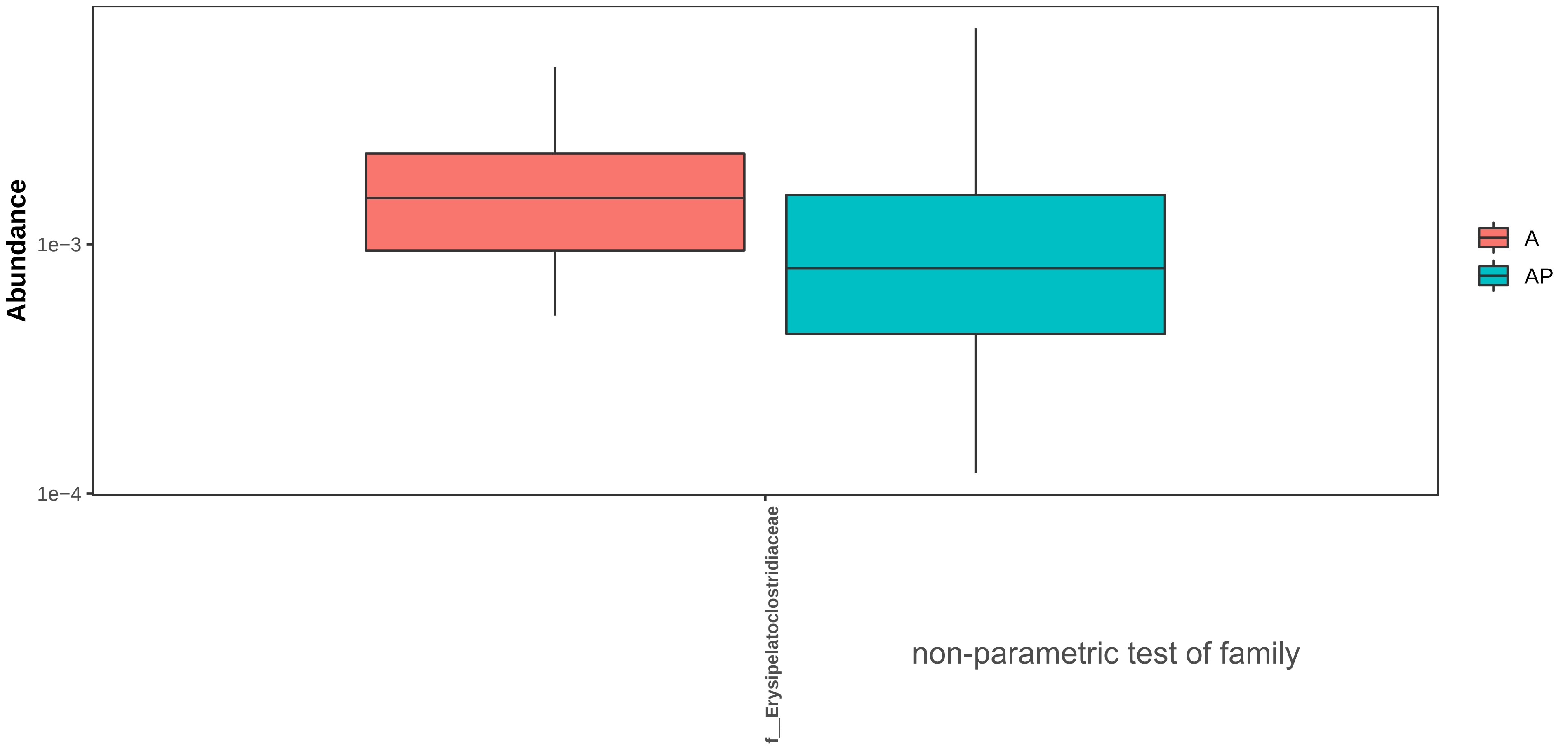

Abundance
1e−3
1e−4
f__Erysipelatoclostridiaceae
A
AP
non-parametric test of family

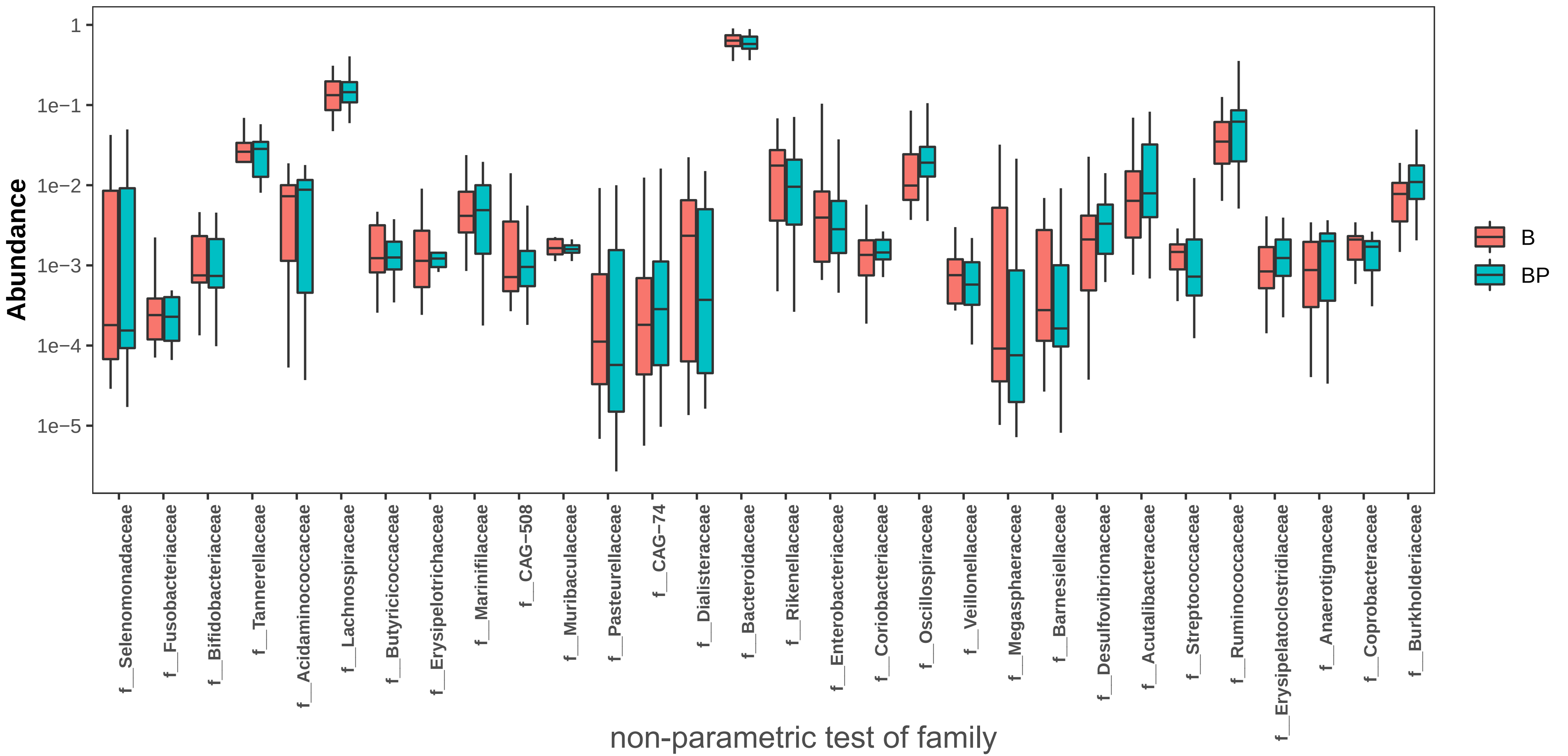

Abundance
1
1e−1
1e−2
1e−3
1e−4
1e−5
f__Selenomonadaceae
f__Fusobacteriaceae
f__Bifidobacteriaceae
f__Tannerellaceae
f__Acidaminococcaceae
f__Lachnospiraceae
f__Butyricicoccaceae
f__Erysipelotrichaceae
f__Marinifilaceae
f__CAG−508
f__Muribaculaceae
f__Pasteurellaceae
f__CAG−74
f__Dialisteraceae
f__Bacteroidaceae
f__Rikenellaceae
f__Enterobacteriaceae
f__Coriobacteriaceae
f__Oscillospiraceae
f__Veillonellaceae
f__Megasphaeraceae
f__Barnesiellaceae
f__Desulfovibrionaceae
f__Acutalibacteraceae
f__Streptococcaceae
f__Ruminococcaceae
f__Erysipelatoclostridiaceae
f__Anaerotignaceae
f__Coprobacteraceae
f__Burkholderiaceae
non-parametric test of family
B
BP

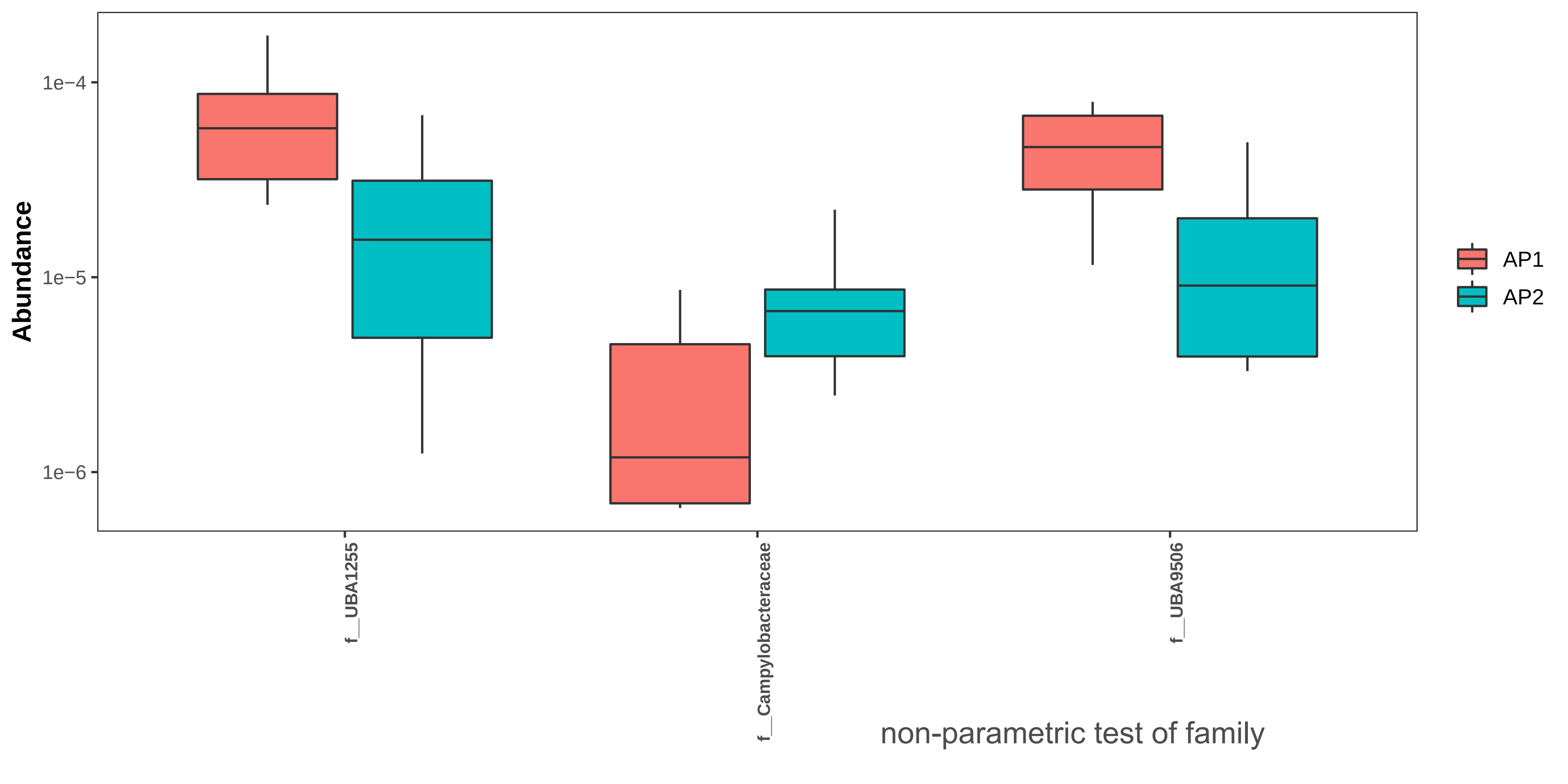

Abundance
1e−4
1e−5
1e−6
f__UBA1255
f__Campylobacteraceae
f__UBA9506
AP1
AP2
non-parametric test of family

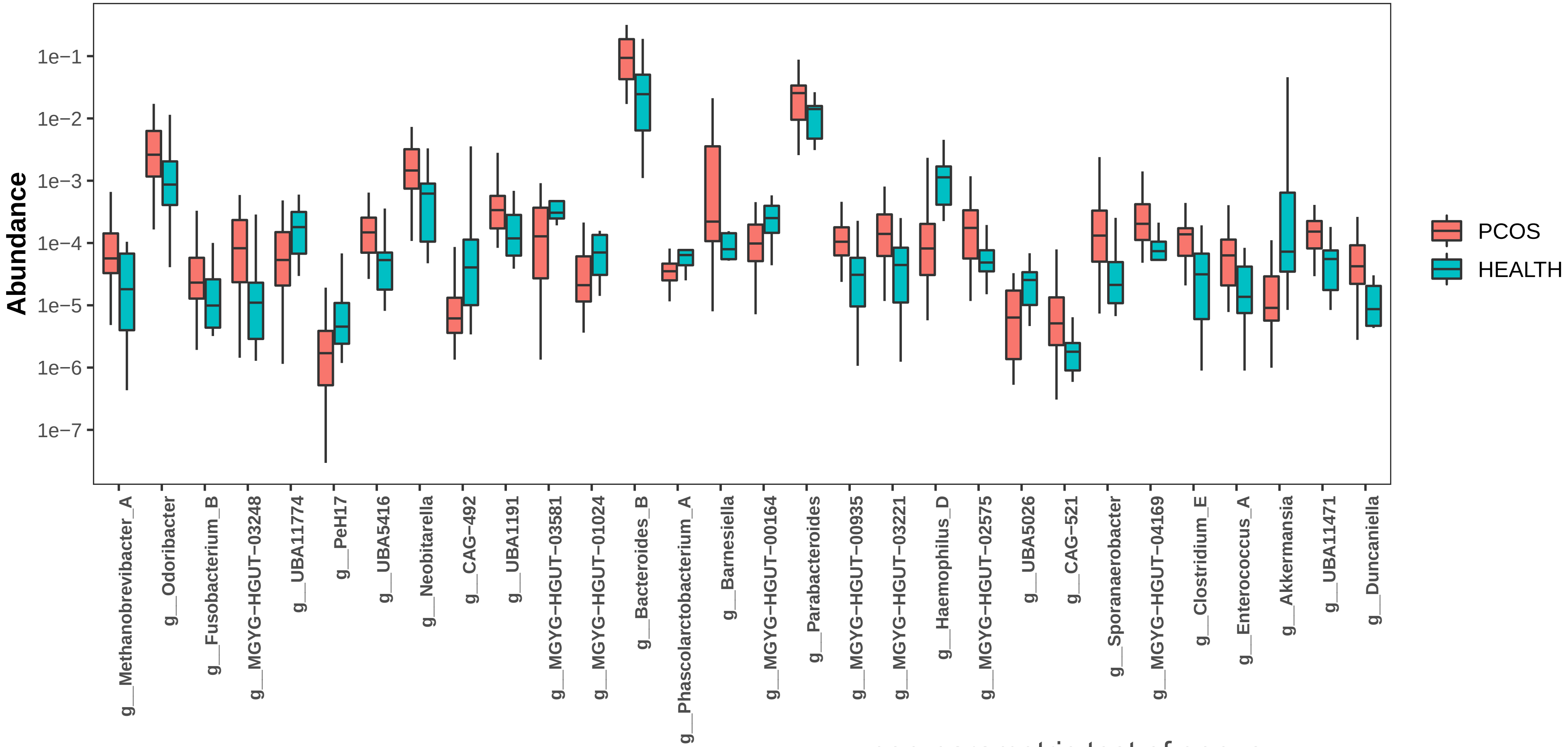

non-parametric test of genus

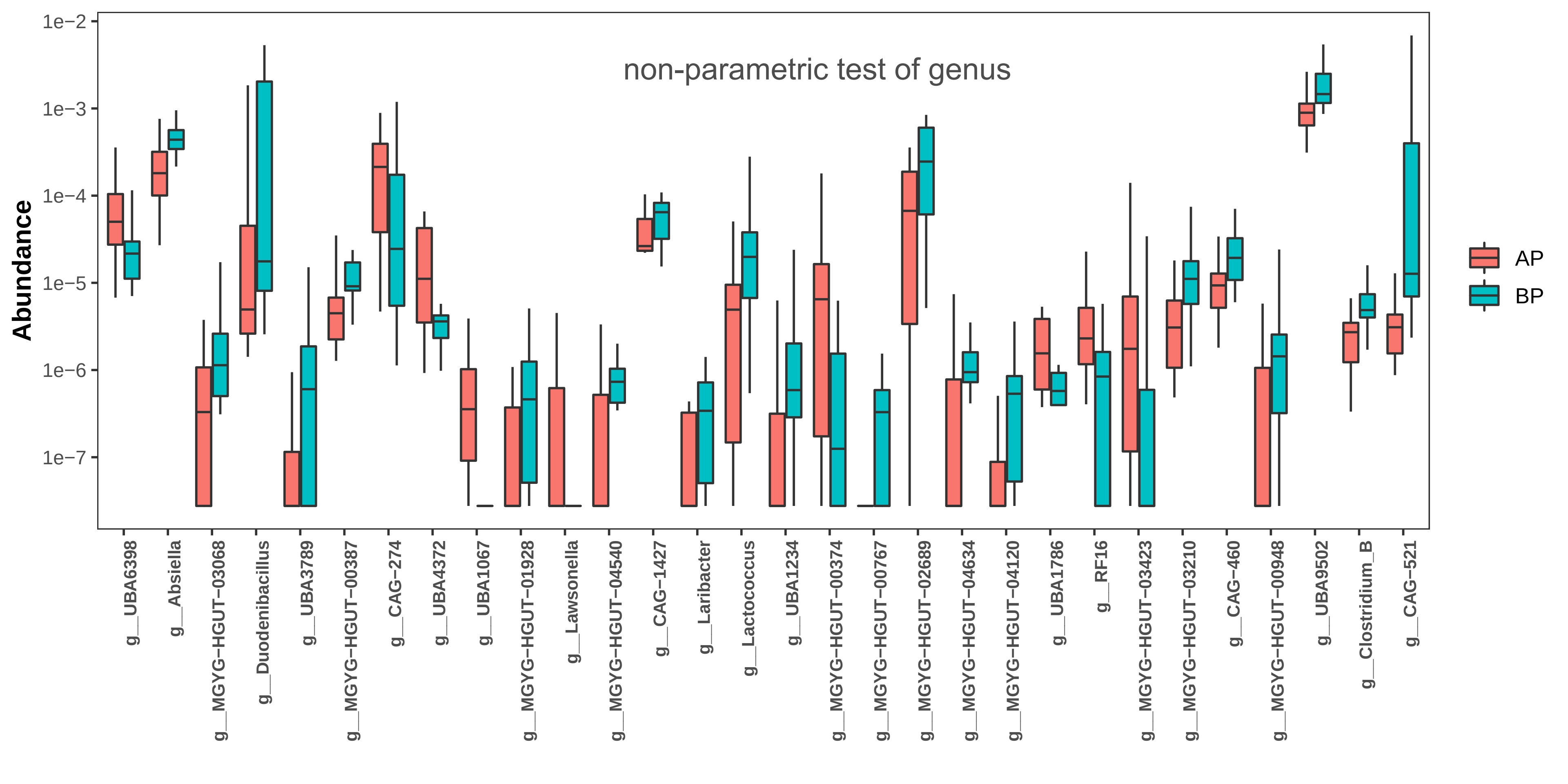

non-parametric test of genus
Abundance
1e−2
1e−3
1e−4
1e−5
1e−6
1e−7
AP
BP
g__UBA6398
g__Absiella
g__MGYG−HGUT−03068
g__Duodenibacillus
g__UBA3789
g__MGYG−HGUT−00387
g__CAG−274
g__UBA4372
g__UBA1067
g__MGYG−HGUT−01928
g__Lawsonella
g__MGYG−HGUT−04540
g__CAG−1427
g__Laribacter
g__Lactococcus
g__UBA1234
g__MGYG−HGUT−00374
g__MGYG−HGUT−00767
g__MGYG−HGUT−02689
g__MGYG−HGUT−04634
g__MGYG−HGUT−04120
g__UBA1786
g__RF16
g__MGYG−HGUT−03423
g__MGYG−HGUT−03210
g__CAG−460
g__MGYG−HGUT−00948
g__UBA9502
g__Clostridium_B
g__CAG−521

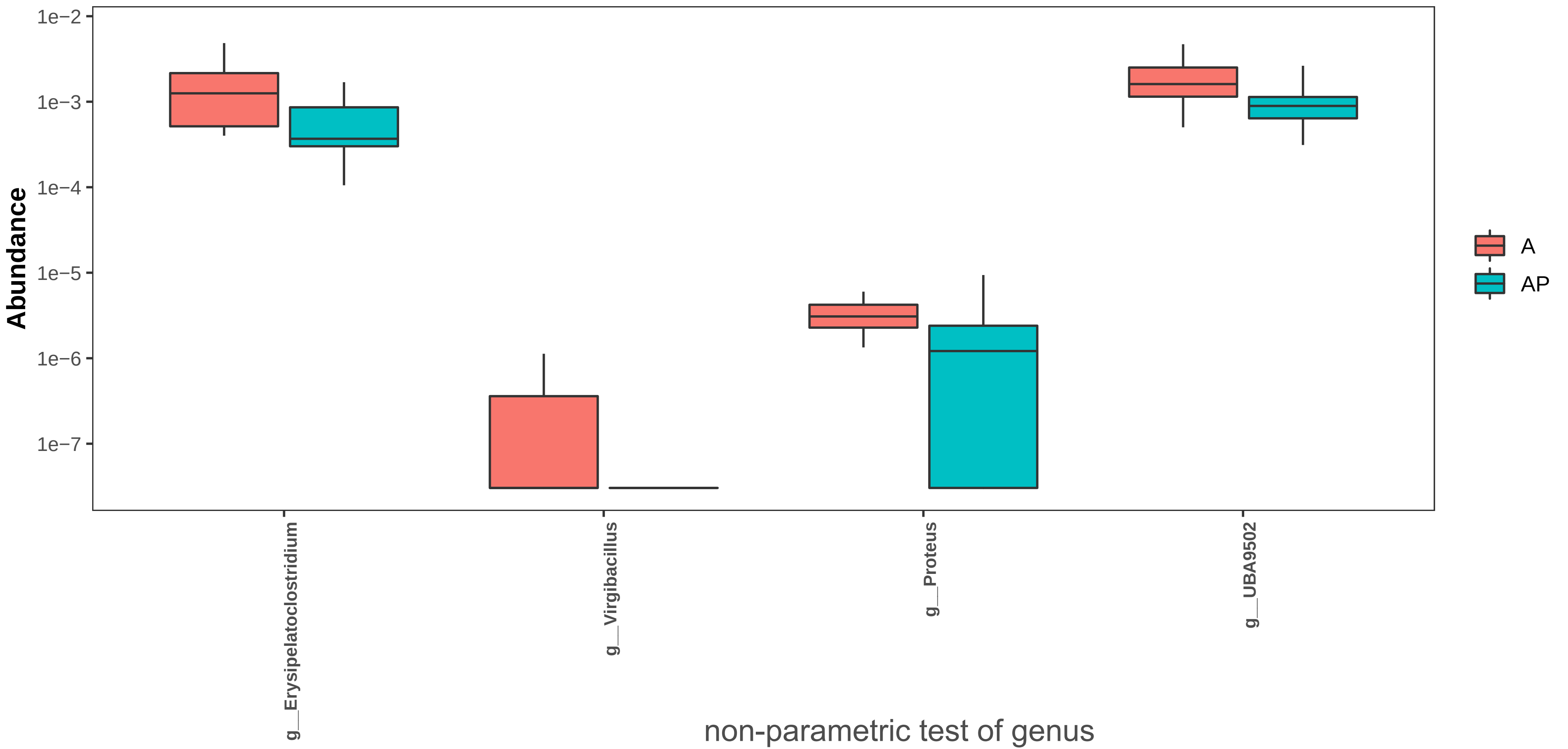

Abundance
1e−2
1e−3
1e−4
1e−5
1e−6
1e−7
g__Erysipelatoclostridium
g__Virgibacillus
g__Proteus
g__UBA9502
non-parametric test of genus
A
AP

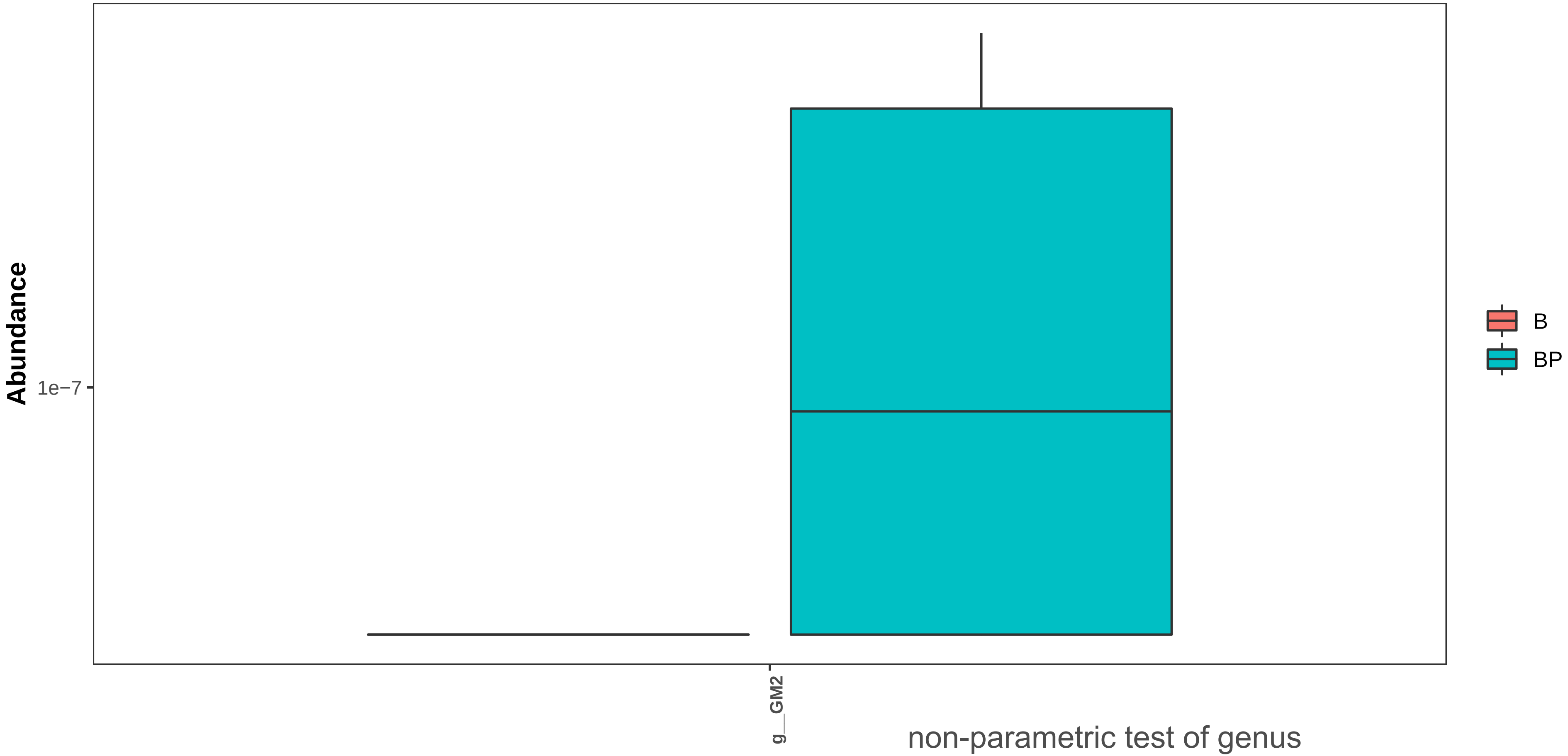
Abundance
1e−7
g__GM2
non-parametric test of genus
B
BP

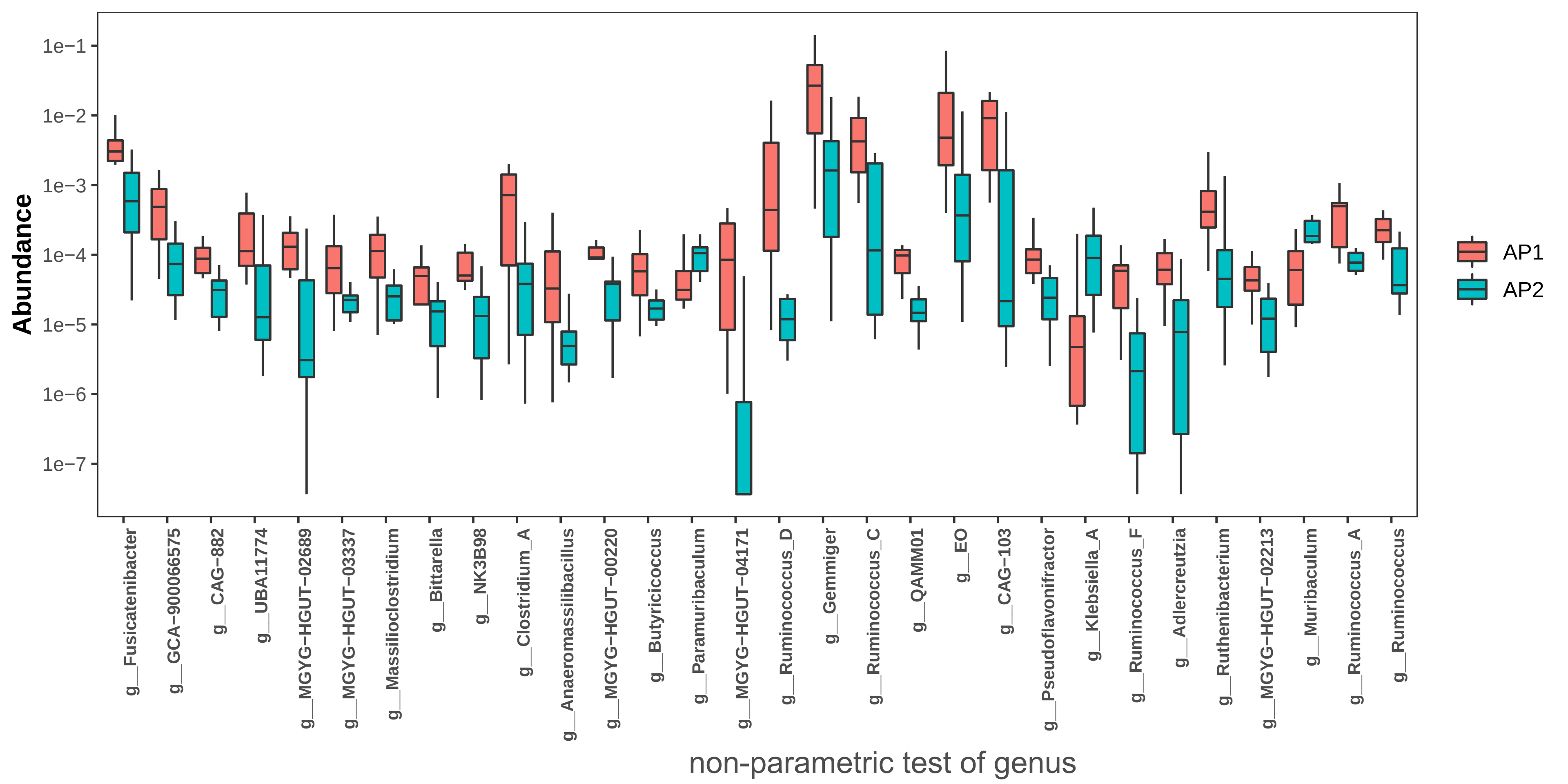

Abundance
1e−1
1e−2
1e−3
1e−4
1e−5
1e−6
1e−7
AP1
AP2
g__Fusicatenibacter
g__GCA−900066575
g__CAG−882
g__UBA11774
g__MGYG−HGUT−02689
g__MGYG−HGUT−03337
g__Massilioclostridium
g__Bittarella
g__NK3B98
g__Clostridium_A
g__Anaeromassilibacillus
g__MGYG−HGUT−00220
g__Butyricicoccus
g__Paramuribaculum
g__MGYG−HGUT−04171
g__Ruminococcus_D
g__Gemmiger
g__Ruminococcus_C
g__QAMM01
g__EO
g__CAG−103
g__Pseudoflavonifractor
g__Klebsiella_A
g__Ruminococcus_F
g__Adlercreutzia
g__Ruthenibacterium
g__MGYG−HGUT−02213
g__Muribaculum
g__Ruminococcus_A
g__Ruminococcus
non-parametric test of genus

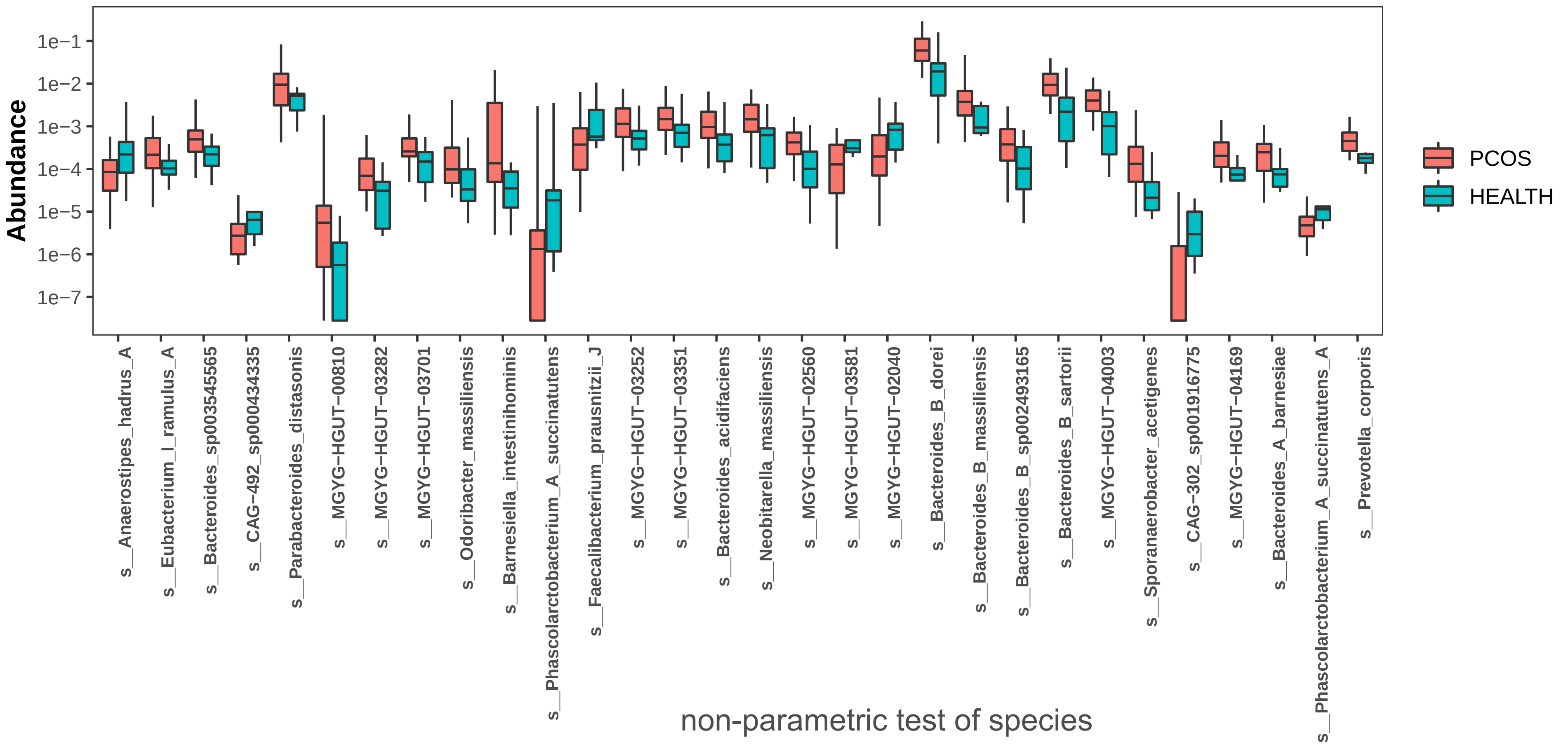
Abundance
1e−1
1e−2
1e−3
1e−4
1e−5
1e−6
1e−7
s__Anaerostipes_hadrus_A
s__Eubacterium_I_ramulus_A
s__Bacteroides_sp003545565
s__CAG−492_sp000434335
s__Parabacteroides_distasonis
s__MGYG−HGUT−00810
s__MGYG−HGUT−03282
s__MGYG−HGUT−03701
s__Odoribacter_massiliensis
s__Barnesiella_intestinihominis
s__Phascolarctobacterium_A_succinatutens
s__Faecalibacterium_prausnitzii_J
s__MGYG−HGUT−03252
s__MGYG−HGUT−03351
s__Bacteroides_acidifaciens
s__Neobitarella_massiliensis
s__MGYG−HGUT−02560
s__MGYG−HGUT−03581
s__MGYG−HGUT−02040
s__Bacteroides_B_dorei
s__Bacteroides_B_massiliensis
s__Bacteroides_B_sp002493165
s__Bacteroides_B_sartorii
s__MGYG−HGUT−04003
s__Sporanaerobacter_acetigenes
s__CAG−302_sp001916775
s__MGYG−HGUT−04169
s__Bacteroides_A_barnesiae
s__Phascolarctobacterium_A_succinatutens_A
s__Prevotella_corporis
non-parametric test of species
PCOS
HEALTH

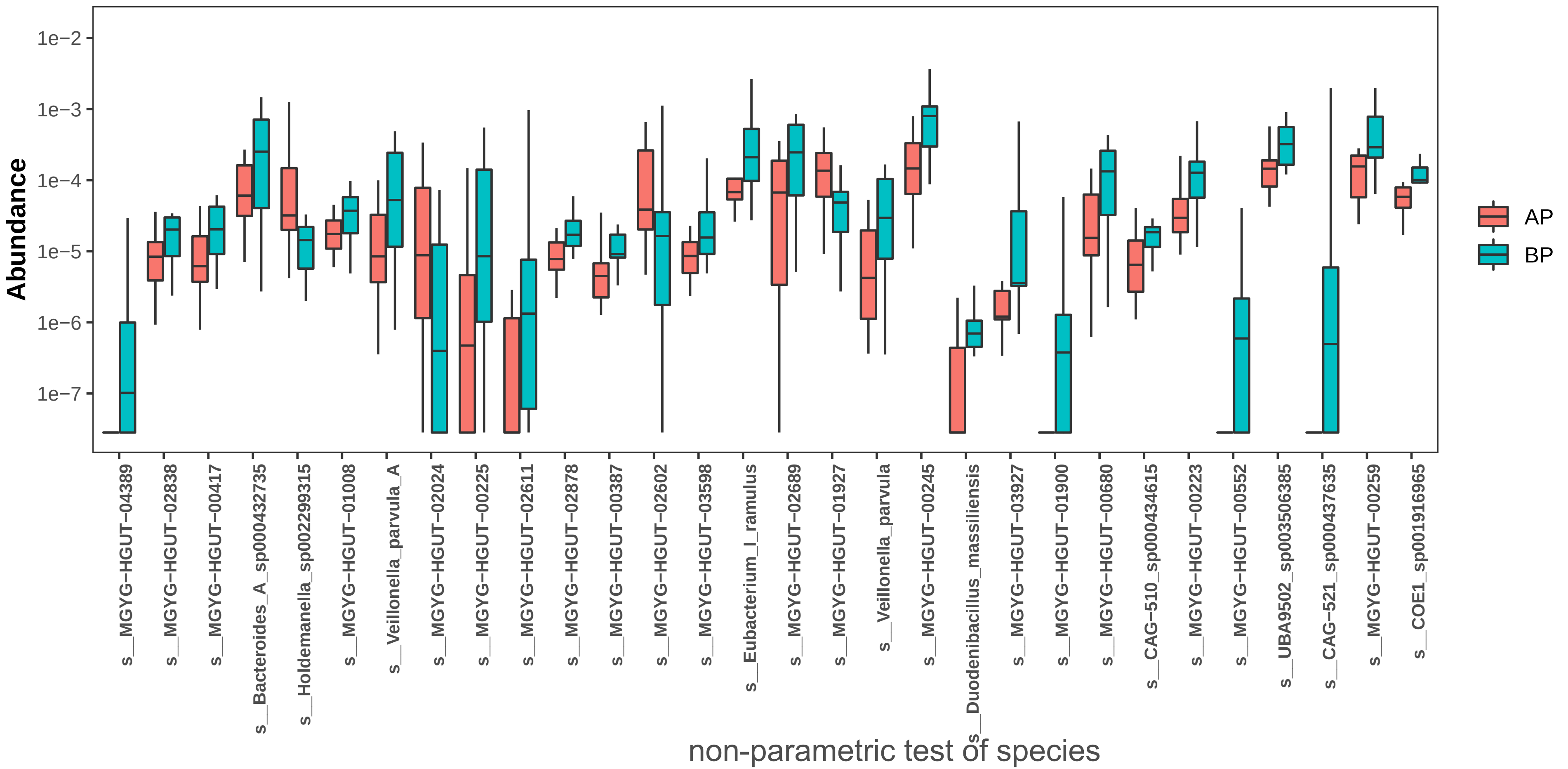

Abundance
1e−2
1e−3
1e−4
1e−5
1e−6
1e−7
s__MGYG−HGUT−04389
s__MGYG−HGUT−02838
s__MGYG−HGUT−00417
s__Bacteroides_A_sp000432735
s__Holdemanella_sp002299315
s__MGYG−HGUT−01008
s__Veillonella_parvula_A
s__MGYG−HGUT−02024
s__MGYG−HGUT−00225
s__MGYG−HGUT−02611
s__MGYG−HGUT−02878
s__MGYG−HGUT−00387
s__MGYG−HGUT−02602
s__MGYG−HGUT−03598
s__Eubacterium_I_ramulus
s__MGYG−HGUT−02689
s__MGYG−HGUT−01927
s__Veillonella_parvula
s__MGYG−HGUT−00245
s__Duodenibacillus_massiliensis
s__MGYG−HGUT−03927
s__MGYG−HGUT−01900
s__MGYG−HGUT−00680
s__CAG−510_sp000434615
s__MGYG−HGUT−00223
s__MGYG−HGUT−00552
s__UBA9502_sp003506385
s__CAG−521_sp000437635
s__MGYG−HGUT−00259
s__COE1_sp001916965
non-parametric test of species
AP
BP

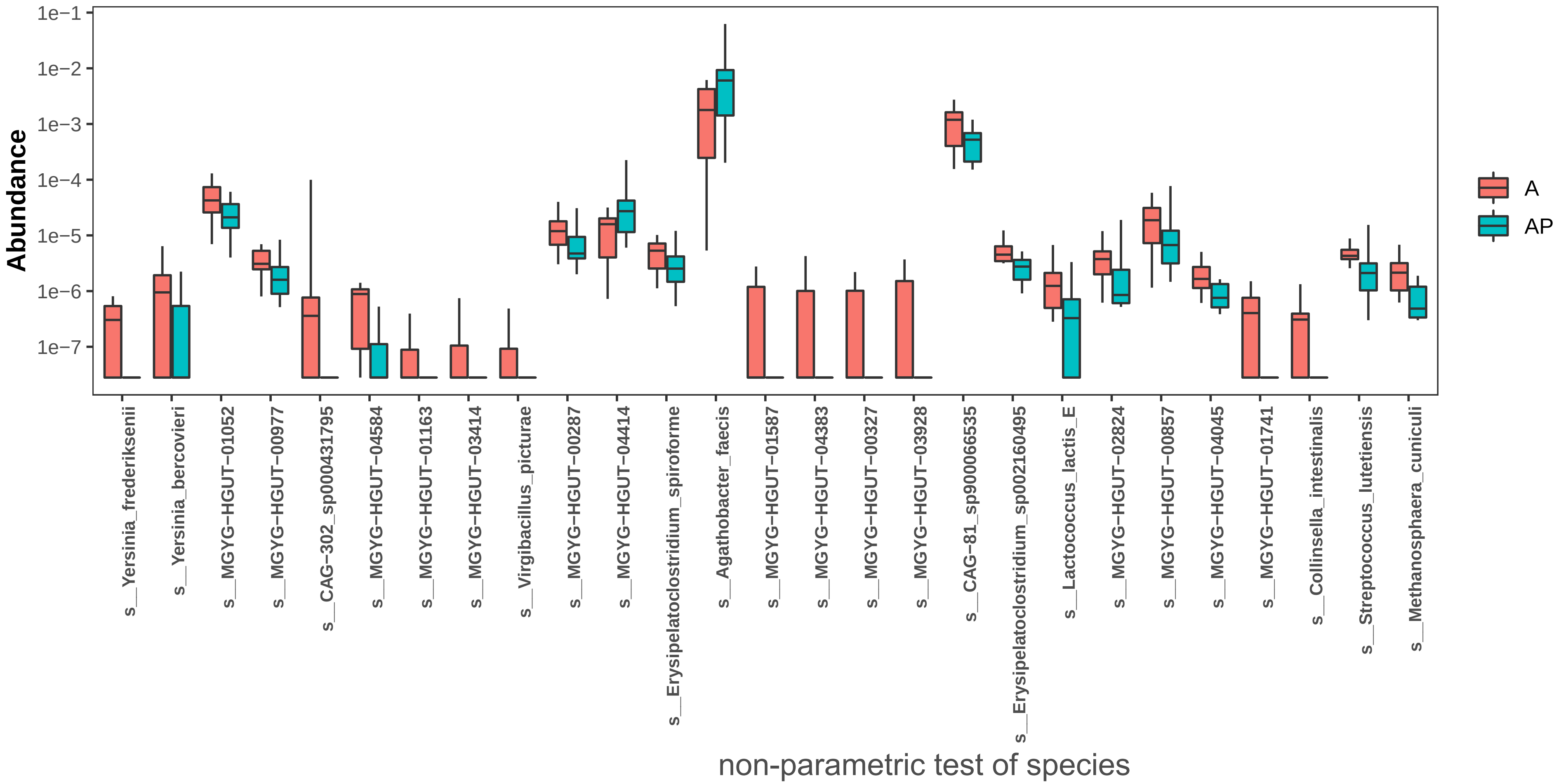

Abundance
1e−1
1e−2
1e−3
1e−4
1e−5
1e−6
1e−7
A
AP
s__Yersinia_frederiksenii
s__Yersinia_bercovieri
s__MGYG−HGUT−01052
s__MGYG−HGUT−00977
s__CAG−302_sp000431795
s__MGYG−HGUT−04584
s__MGYG−HGUT−01163
s__MGYG−HGUT−03414
s__Virgibacillus_picturae
s__MGYG−HGUT−00287
s__MGYG−HGUT−04414
s__Erysipelatoclostridium_spiroforme
s__Agathobacter_faecis
s__MGYG−HGUT−01587
s__MGYG−HGUT−04383
s__MGYG−HGUT−00327
s__MGYG−HGUT−03928
s__CAG−81_sp900066535
s__Erysipelatoclostridium_sp002160495
s__Lactococcus_lactis_E
s__MGYG−HGUT−02824
s__MGYG−HGUT−00857
s__MGYG−HGUT−04045
s__MGYG−HGUT−01741
s__Collinsella_intestinalis
s__Streptococcus_lutetiensis
s__Methanosphaera_cuniculi
non-parametric test of species

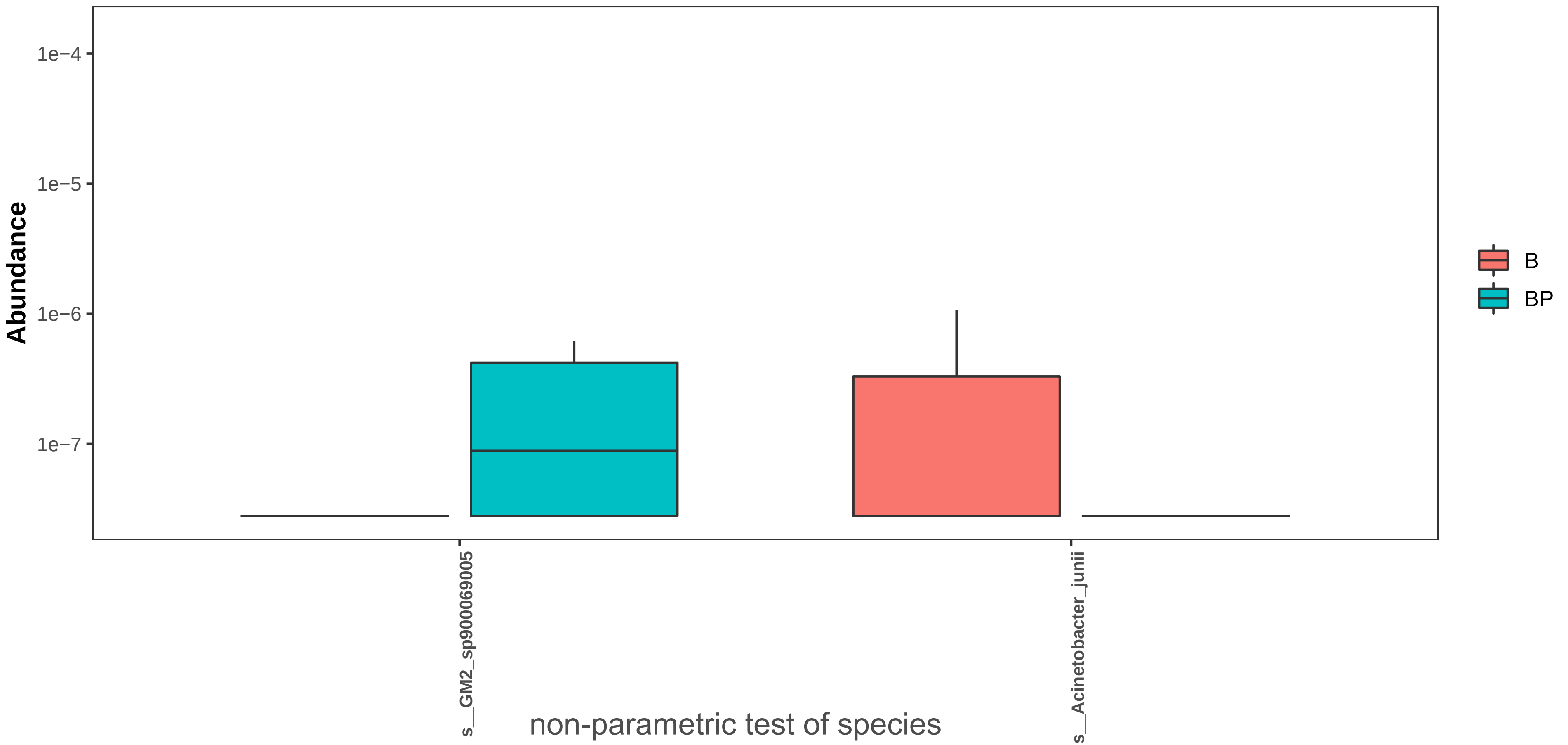

Abundance
1e−4
1e−5
1e−6
1e−7
s__GM2_sp900069005
s__Acinetobacter_junii
non-parametric test of species
B
BP

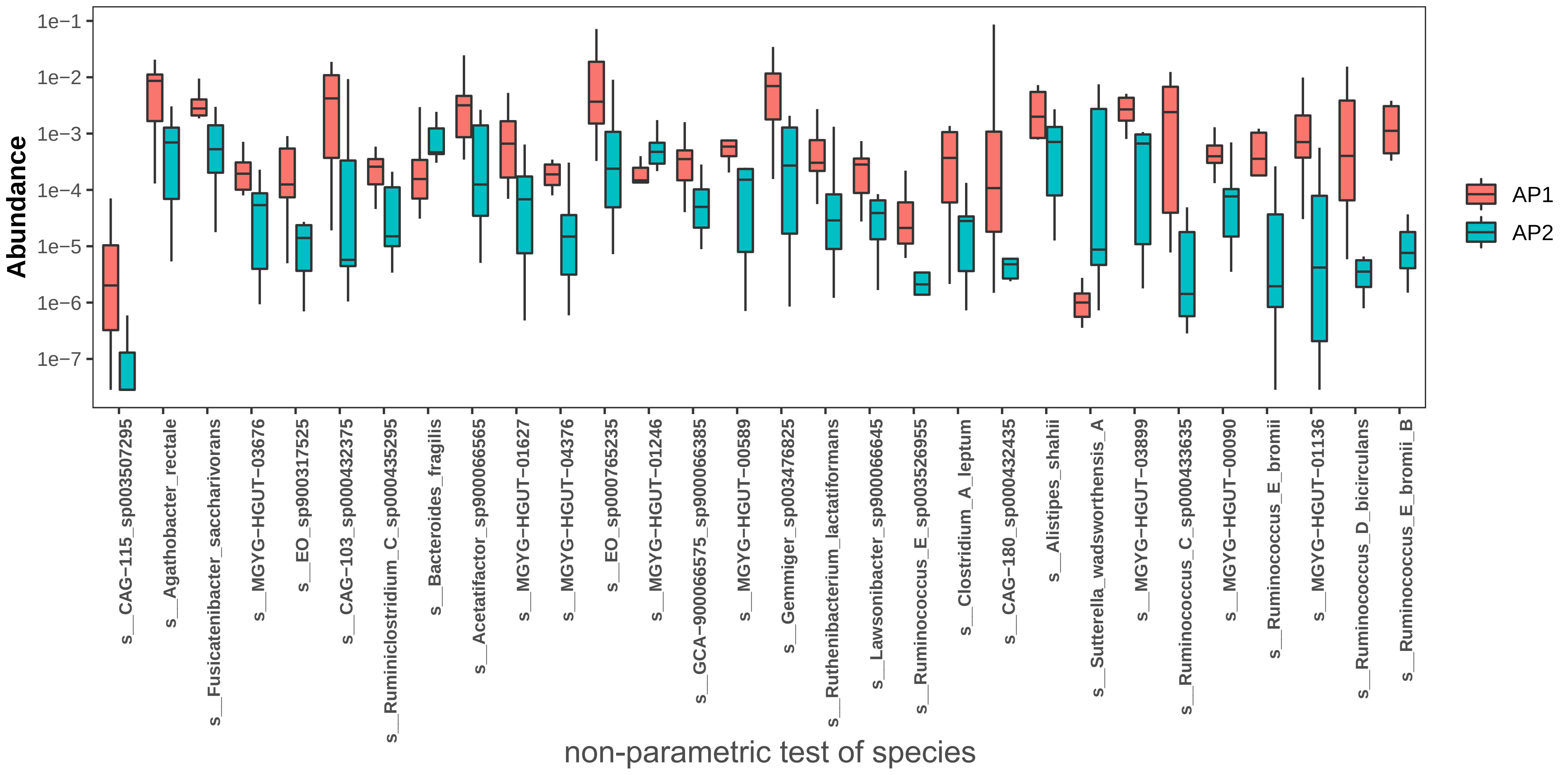

Abundance
1e−1
1e−2
1e−3
1e−4
1e−5
1e−6
1e−7
AP1
AP2
s__CAG−115_sp003507295
s__Agathobacter_rectale
s__Fusicatenibacter_saccharivorans
s__MGYG−HGUT−03676
s__EO_sp900317525
s__CAG−103_sp000432375
s__Ruminiclostridium_C_sp000435295
s__Bacteroides_fragilis
s__Acetatifactor_sp900066565
s__MGYG−HGUT−01627
s__MGYG−HGUT−04376
s__EO_sp000765235
s__MGYG−HGUT−01246
s__GCA−900066575_sp900066385
s__MGYG−HGUT−00589
s__Gemmiger_sp003476825
s__Ruthenibacterium_lactatiformans
s__Lawsonibacter_sp900066645
s__Ruminococcus_E_sp003526955
s__Clostridium_A_leptum
s__CAG−180_sp000432435
s__Alistipes_shahii
s__Sutterella_wadsworthensis_A
s__MGYG−HGUT−03899
s__Ruminococcus_C_sp000433635
s__MGYG−HGUT−00090
s__Ruminococcus_E_bromii
s__MGYG−HGUT−01136
s__Ruminococcus_D_bicirculans
s__Ruminococcus_E_bromii_B
non-parametric test of species

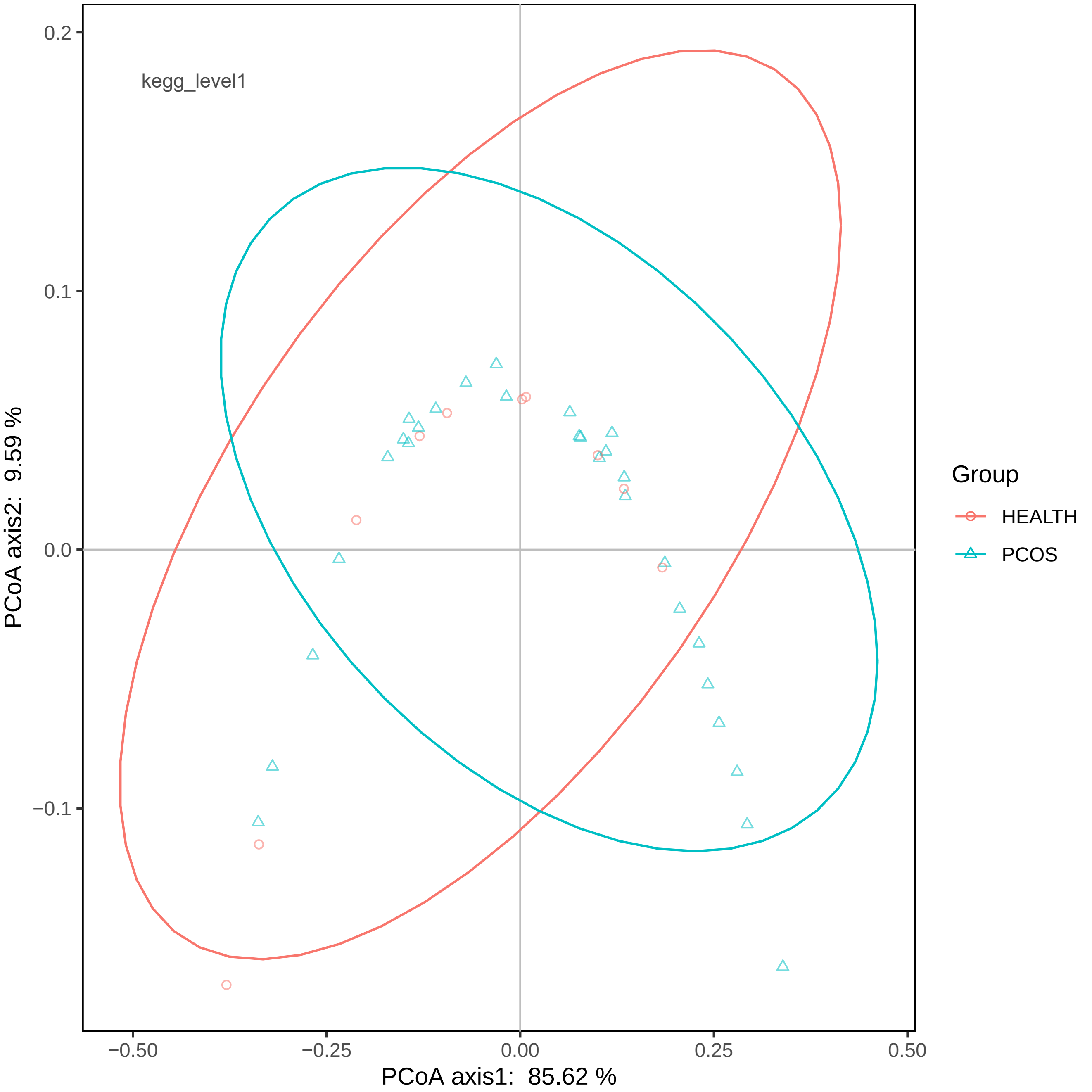
kegg_level1
PCoA axis1: 85.62 %
PCoA axis2: 9.59 %
0.2
0.1
0.0
−0.1
−0.50
−0.25
0.00
0.25
0.50
Group
HEALTH
PCOS

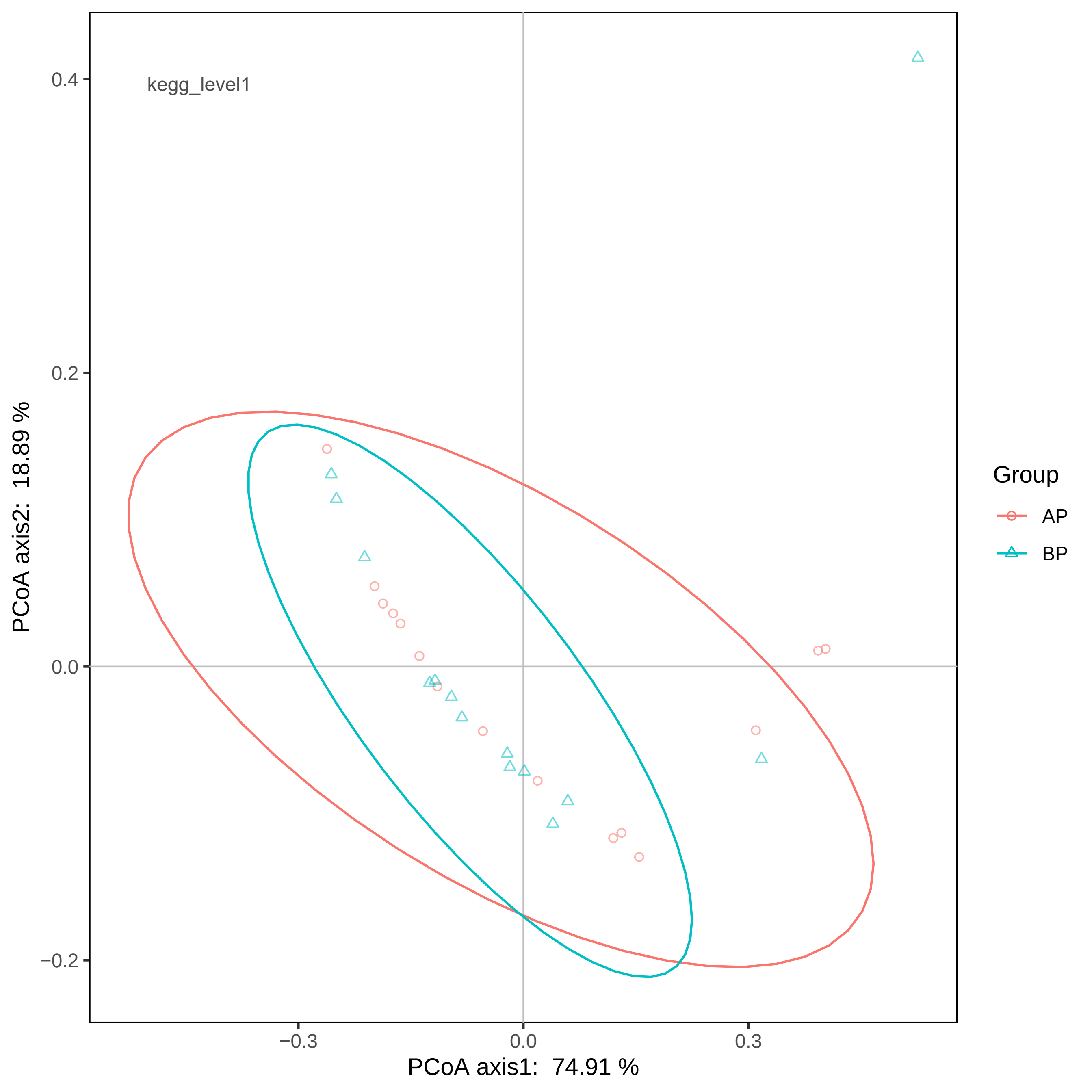

kegg_level1
PCoA axis2: 18.89 %
PCoA axis1: 74.91 %
0.4
0.2
0.0
−0.2
−0.3
0.0
0.3
Group
AP
BP

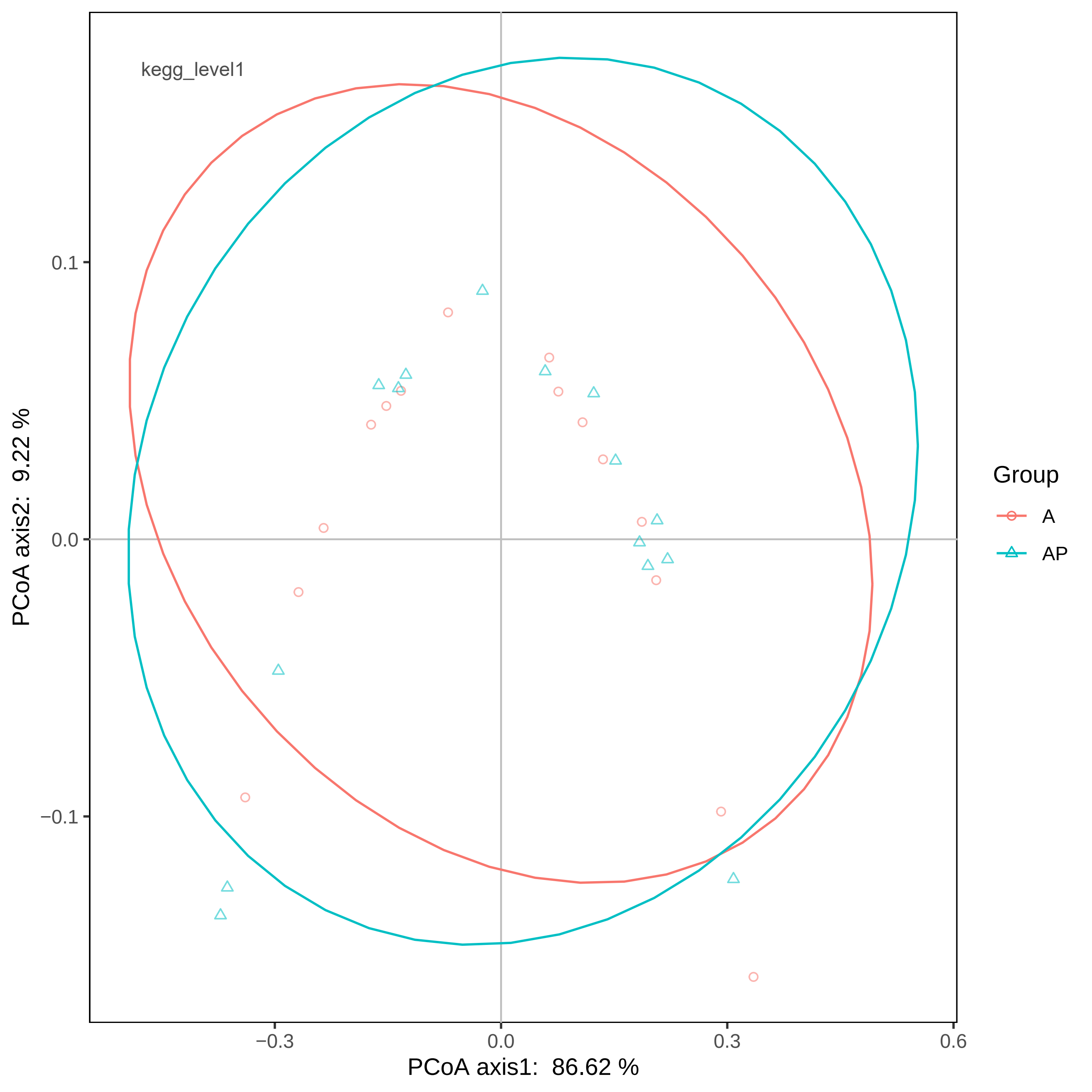

kegg_level1
0.1
0.0
−0.1
−0.3
0.0
0.3
0.6
PCoA axis1: 86.62 %
PCoA axis2: 9.22 %
Group
A
AP

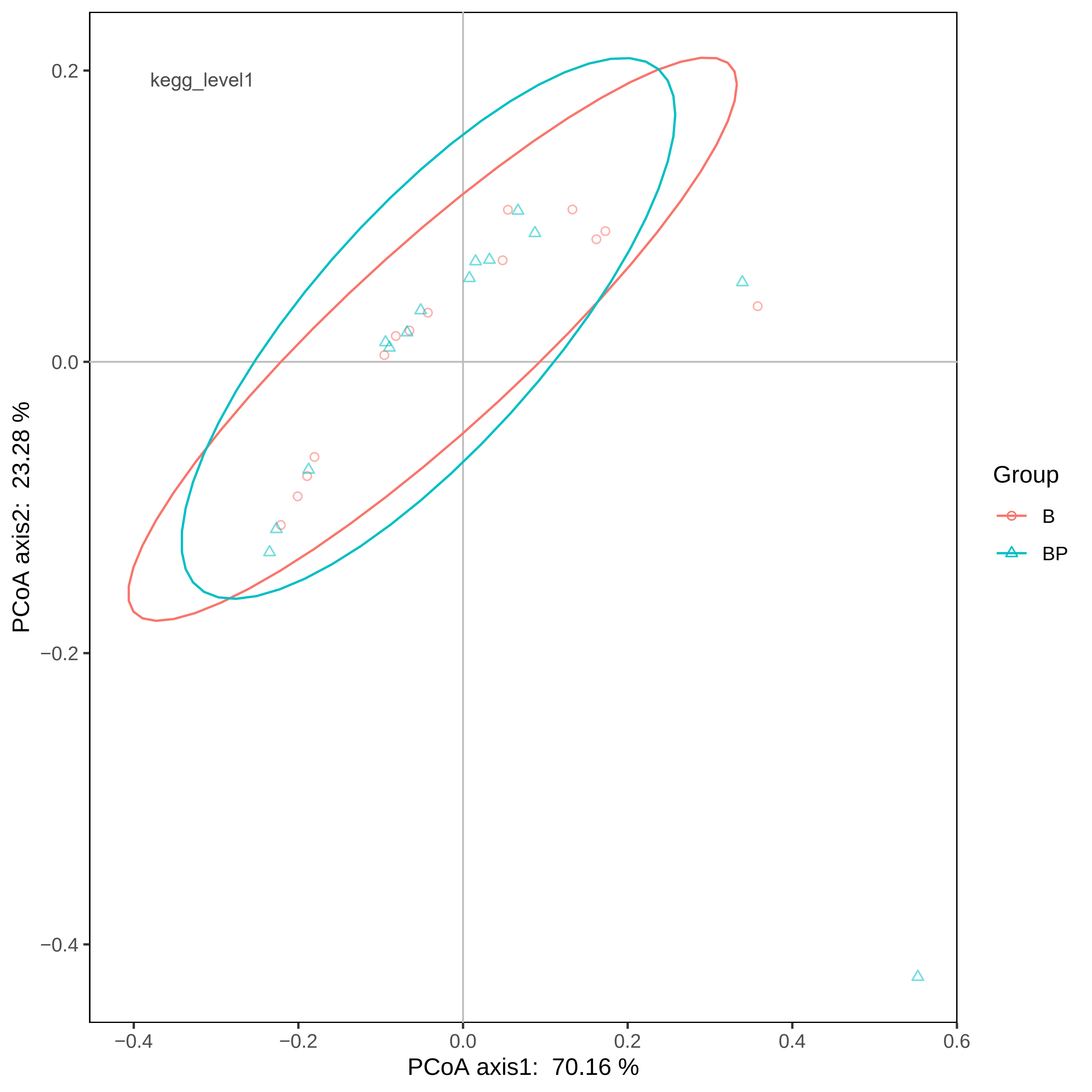
kegg_level1
0.2
0.0
−0.2
−0.4
−0.4
−0.2
0.0
0.2
0.4
0.6
PCoA axis1: 70.16 %
PCoA axis2: 23.28 %
Group
B
BP

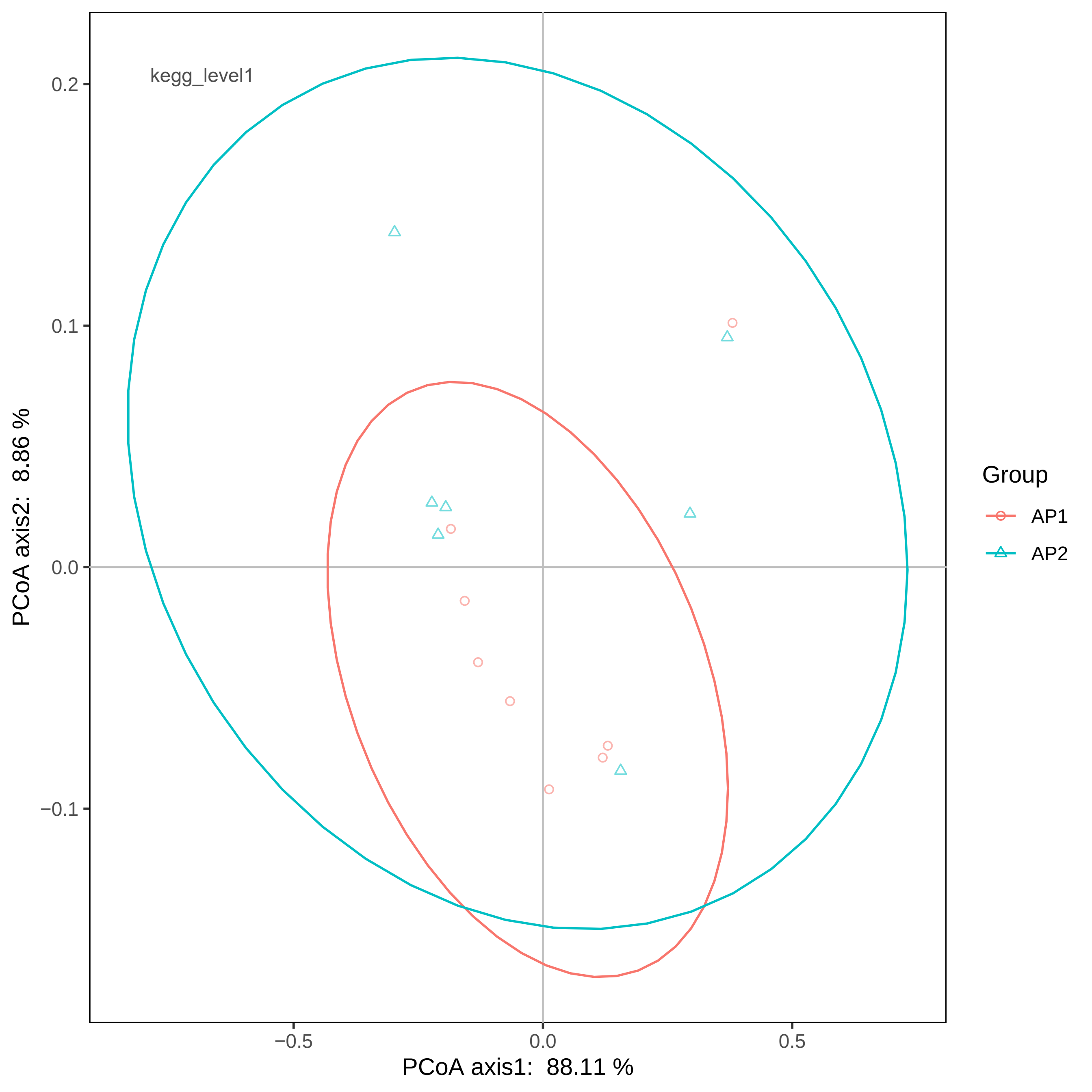

kegg_level1
0.2
0.1
0.0
−0.1
−0.5
0.0
0.5
PCoA axis1: 88.11 %
PCoA axis2: 8.86 %
Group
AP1
AP2

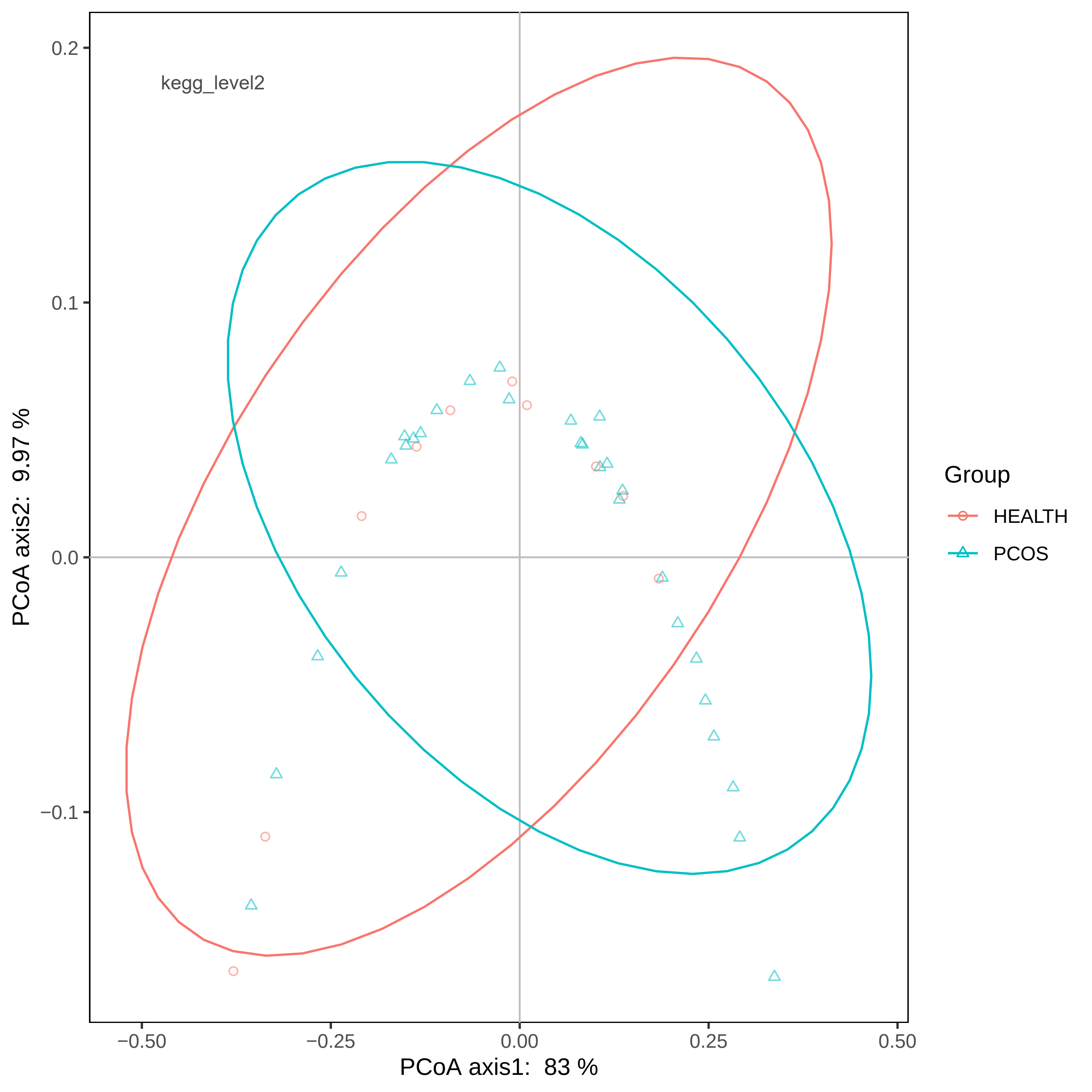

kegg_level2
0.2
0.1
0.0
−0.1
PCoA axis2: 9.97 %
−0.50
−0.25
0.00
0.25
0.50
PCoA axis1: 83 %
Group
HEALTH
PCOS

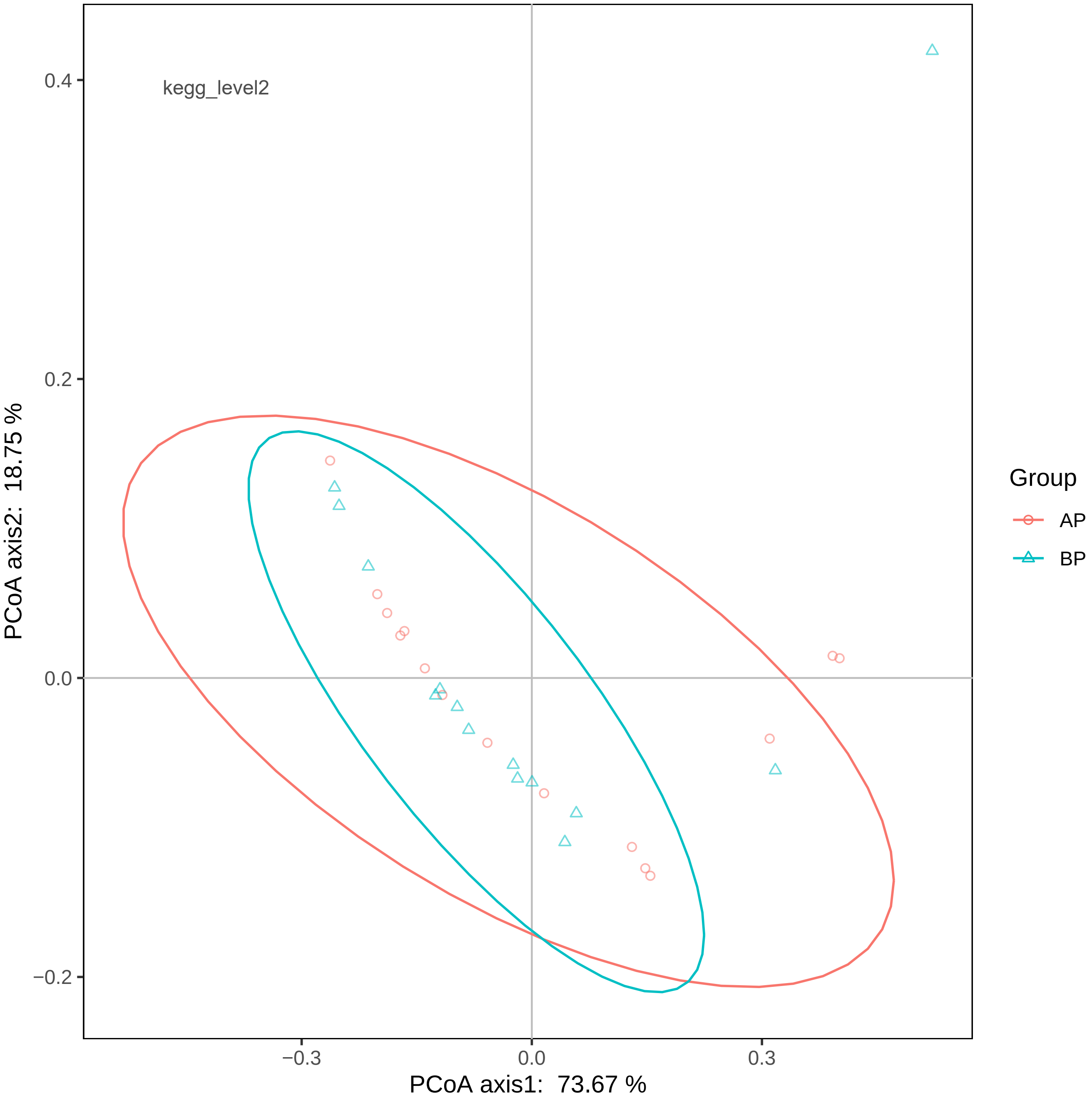

kegg_level2
0.4
0.2
0.0
−0.2
−0.3
0.0
0.3
PCoA axis1: 73.67 %
PCoA axis2: 18.75 %
Group
AP
BP

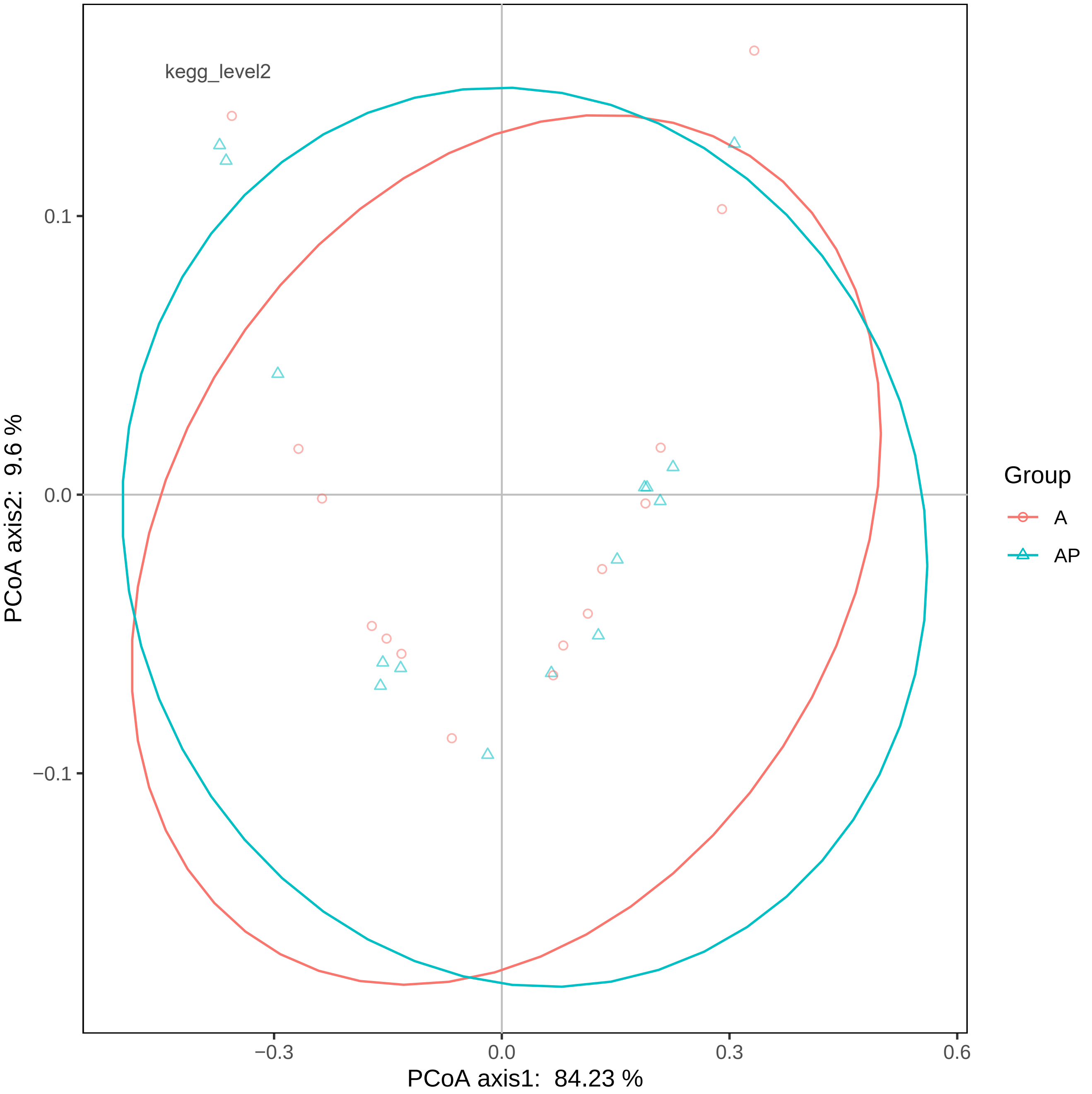

kegg_level2
PCoA axis2: 9.6 %
PCoA axis1: 84.23 %
0.1
0.0
−0.1
−0.3
0.0
0.3
0.6
Group
A
AP

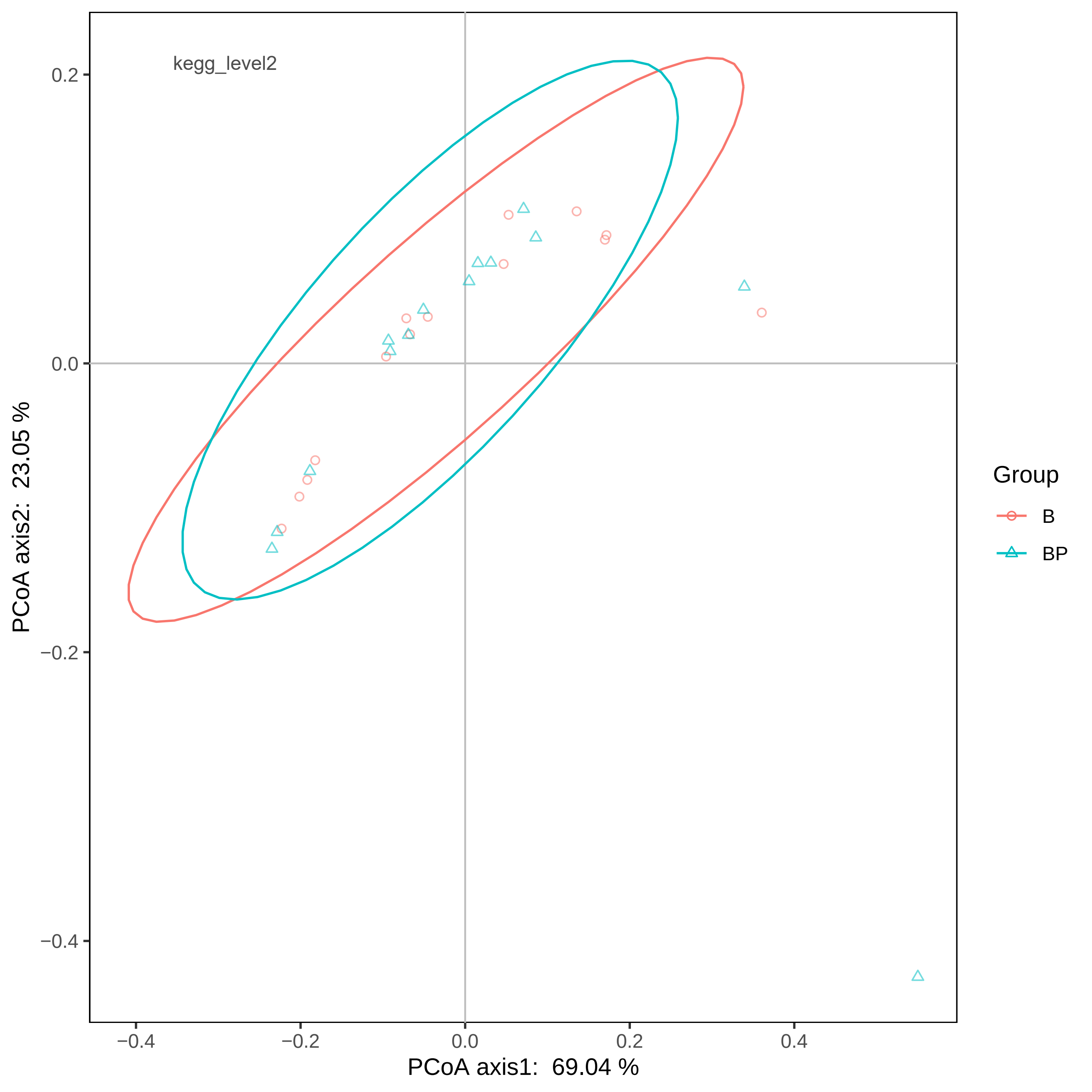

kegg_level2
0.2
0.0
−0.2
−0.4
−0.4
−0.2
0.0
0.2
0.4
PCoA axis1: 69.04 %
PCoA axis2: 23.05 %
Group
B
BP

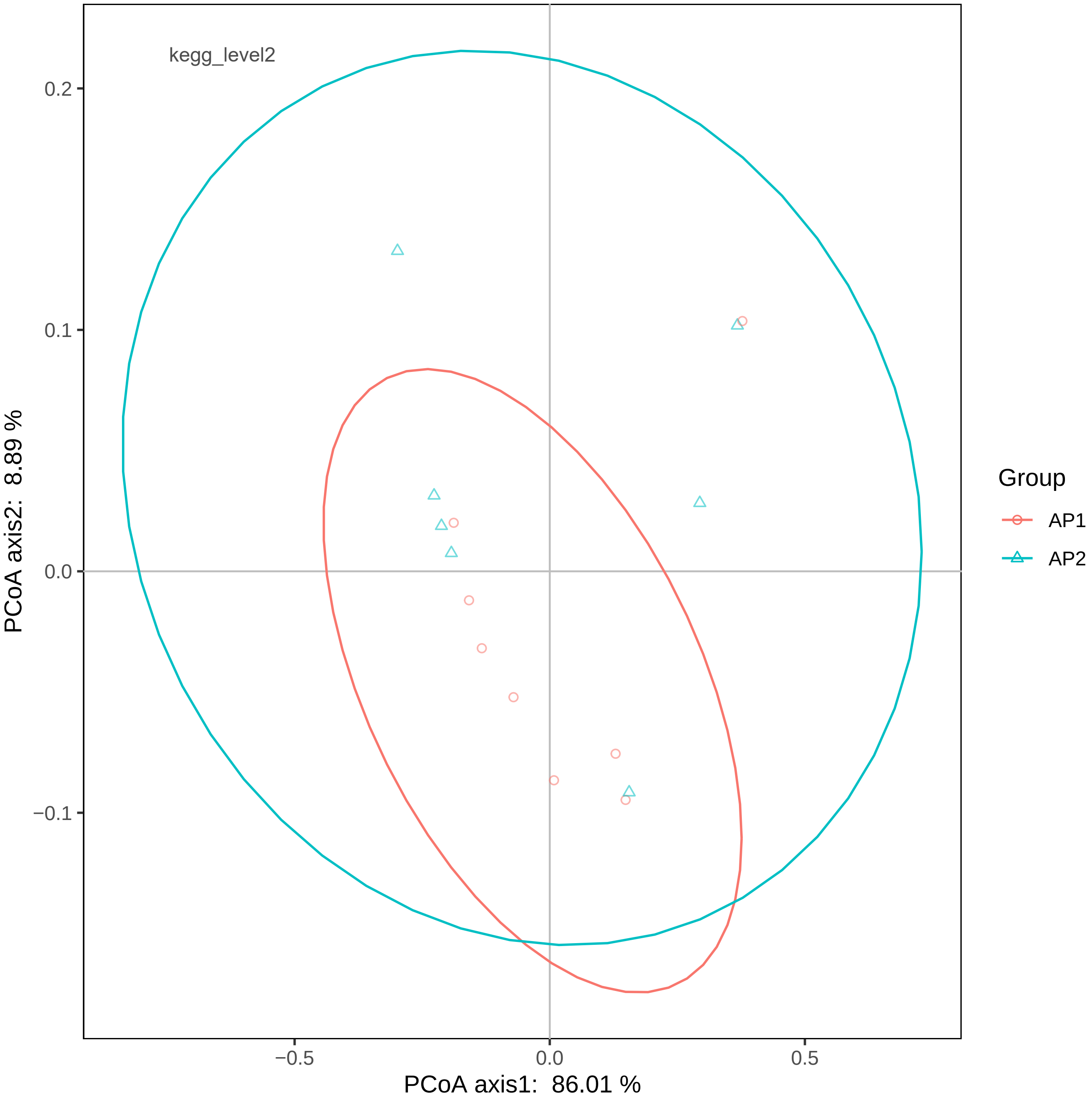

kegg_level2
0.2
0.1
0.0
−0.1
−0.5
0.0
0.5
PCoA axis1: 86.01 %
PCoA axis2: 8.89 %
Group
AP1
AP2

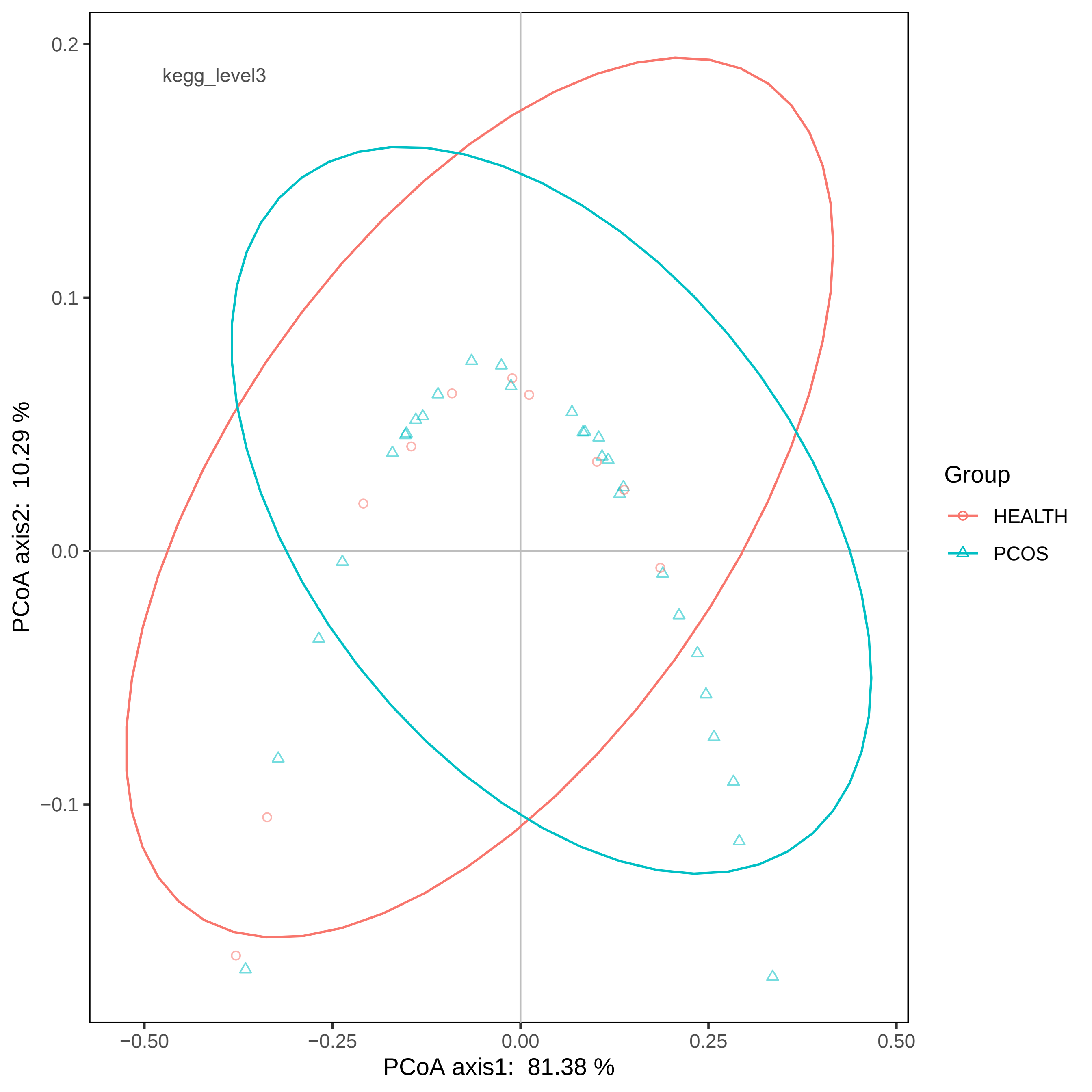
kegg_level3
PCoA axis1: 81.38 %
PCoA axis2: 10.29 %
Group
HEALTH
PCOS
0.2
0.1
0.0
−0.1
−0.50
−0.25
0.00
0.25
0.50

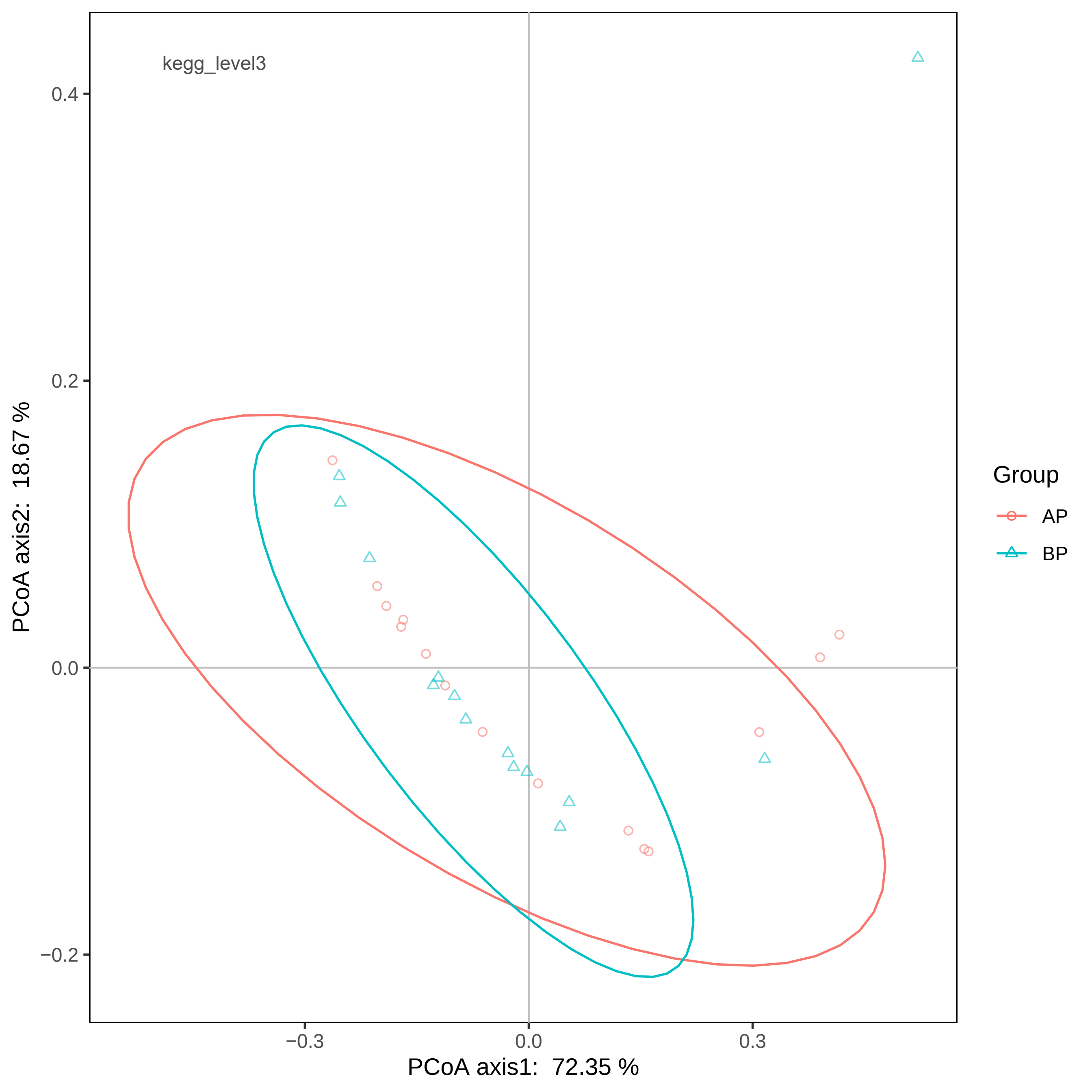
kegg_level3
PCoA axis2: 18.67 %
PCoA axis1: 72.35 %
0.4
0.2
0.0
−0.2
−0.3
0.0
0.3
Group
AP
BP

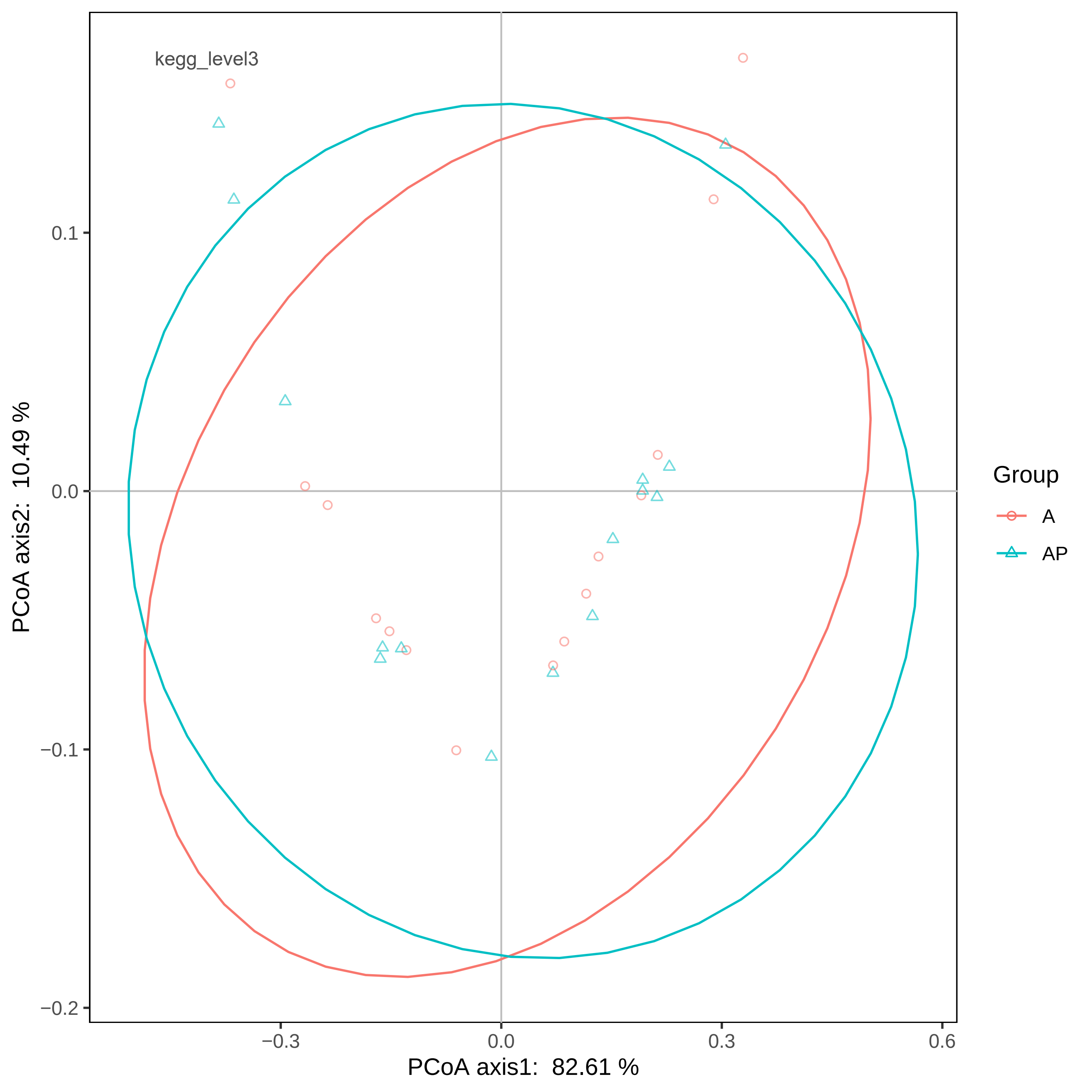

kegg_level3
PCoA axis2: 10.49 %
PCoA axis1: 82.61 %
0.1
0.0
−0.1
−0.2
−0.3
0.0
0.3
0.6
Group
A
AP

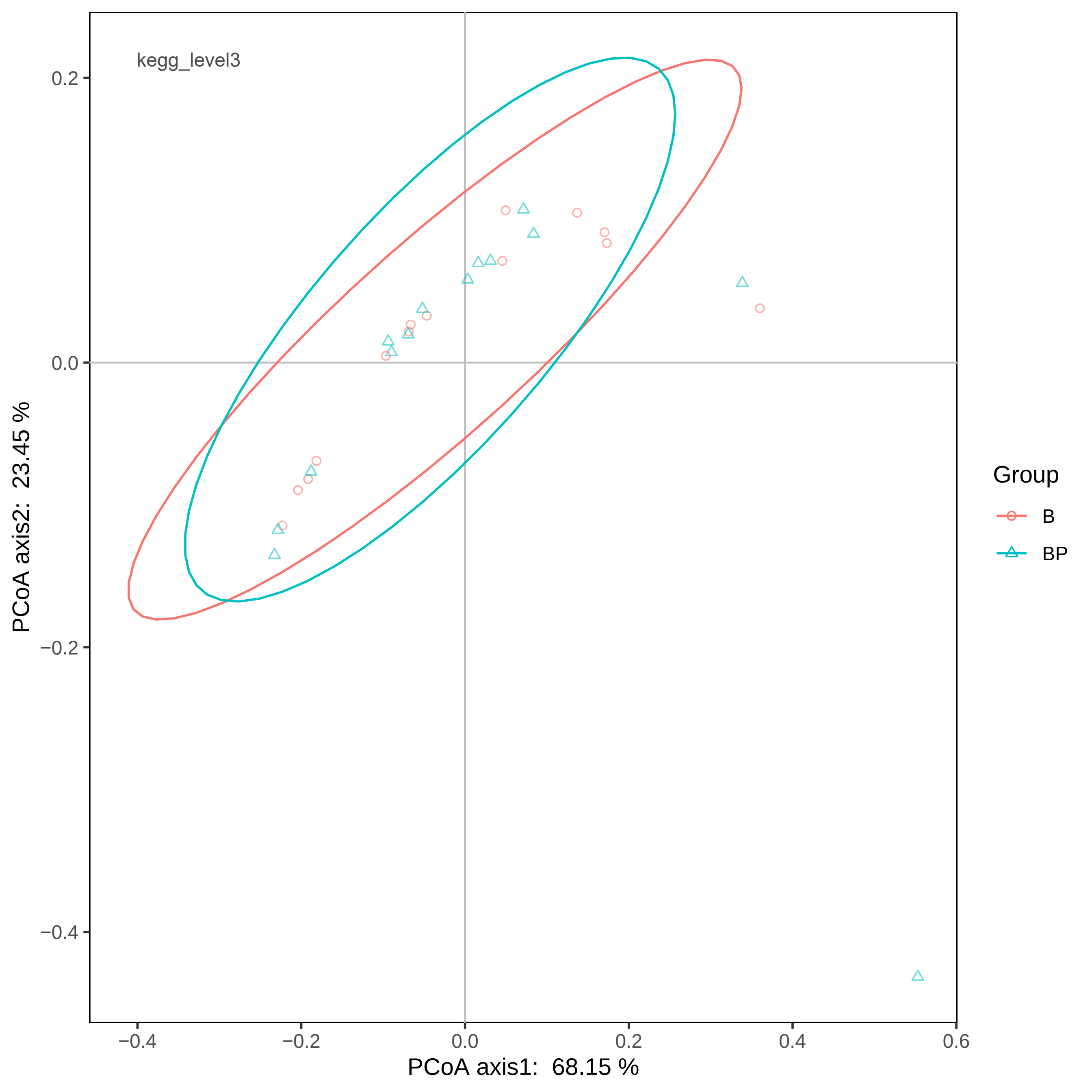
kegg_level3
PCoA axis2: 23.45 %
PCoA axis1: 68.15 %
0.2
0.0
−0.2
−0.4
−0.4
−0.2
0.0
0.2
0.4
0.6
Group
B
BP

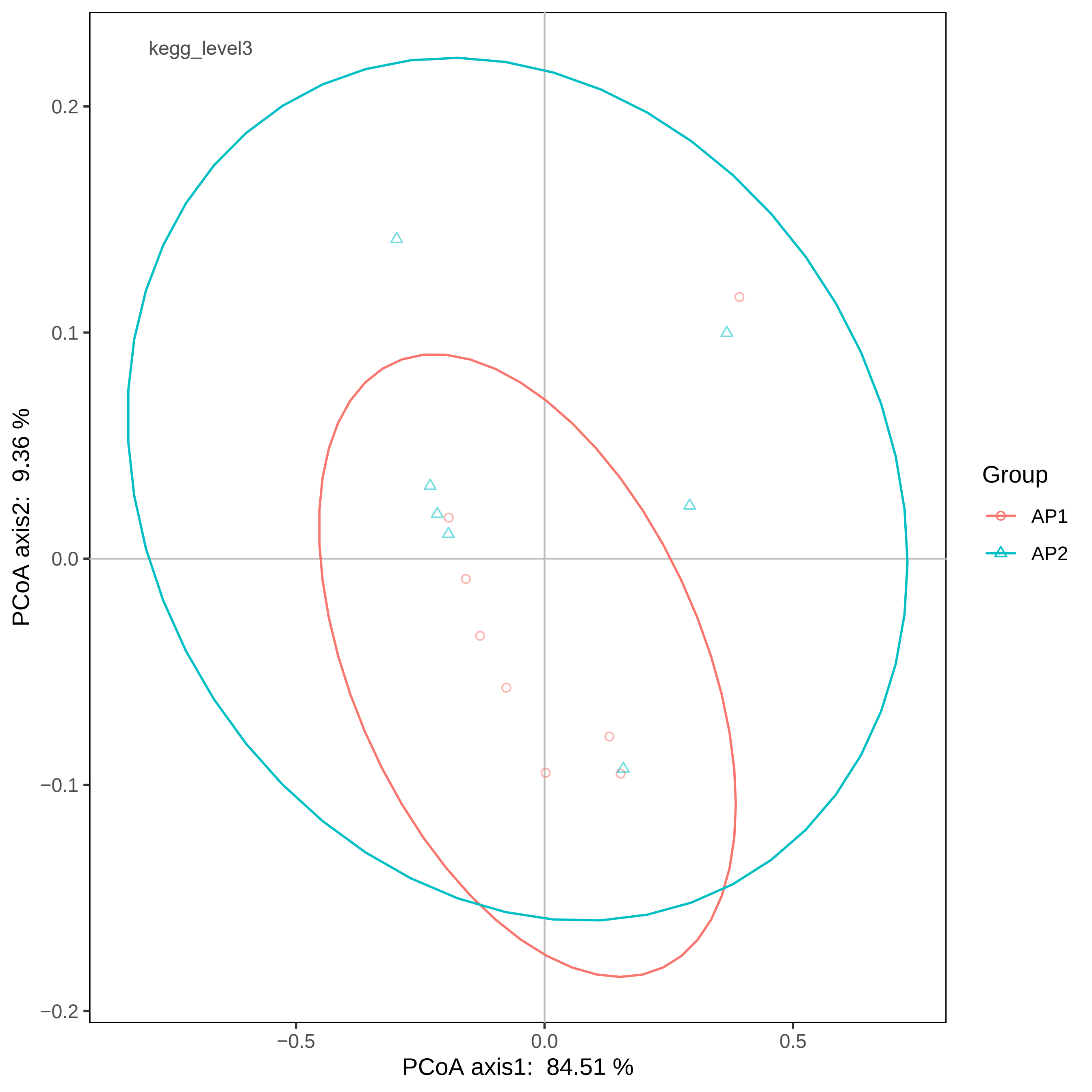
kegg_level3
PCoA axis2: 9.36 %
PCoA axis1: 84.51 %
0.2
0.1
0.0
−0.1
−0.2
−0.5
0.0
0.5
Group
AP1
AP2

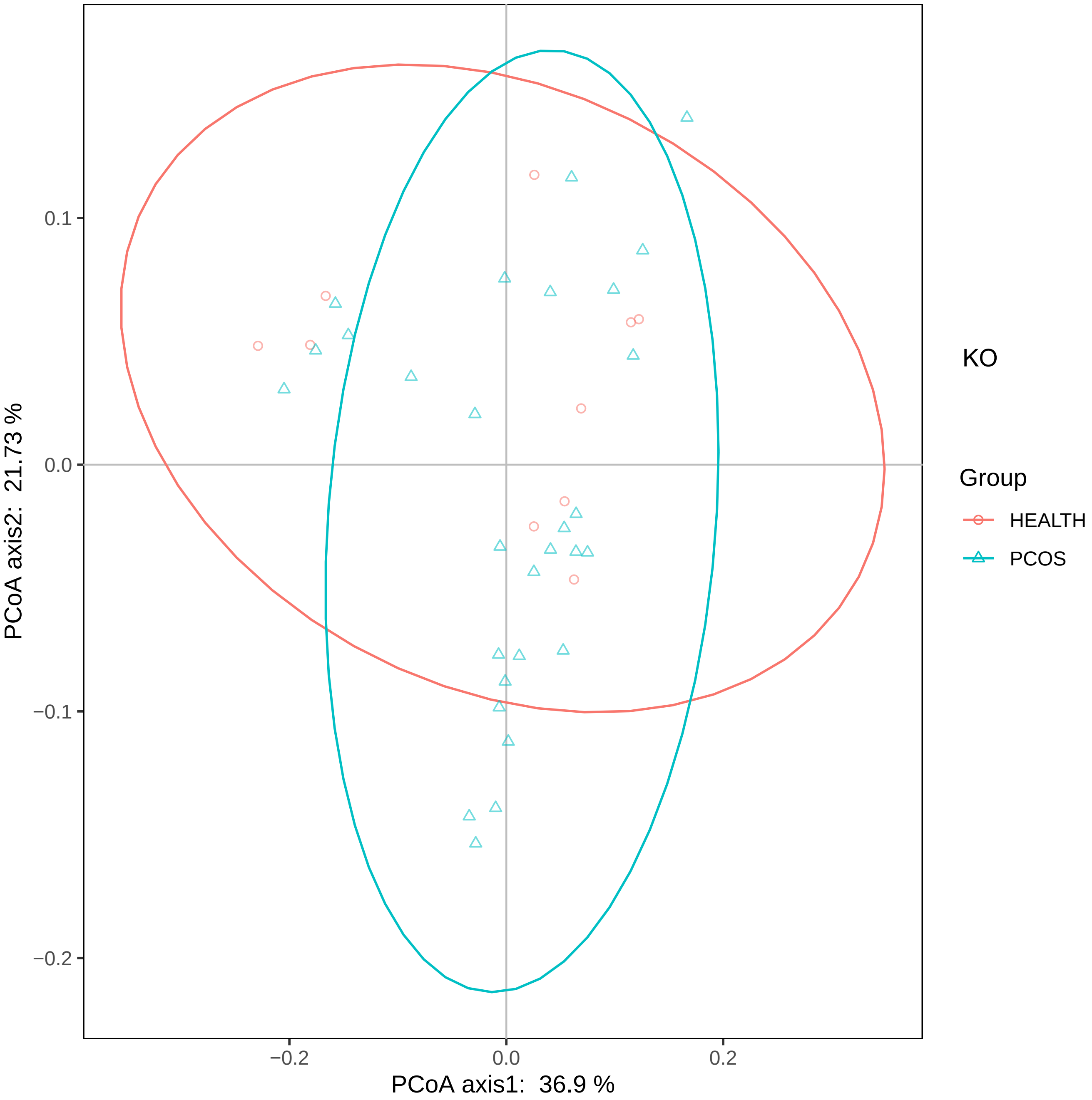

KO
Group
HEALTH
PCOS
PCoA axis1: 36.9 %
PCoA axis2: 21.73 %
0.1
0.0
−0.1
−0.2
−0.2
0.0
0.2

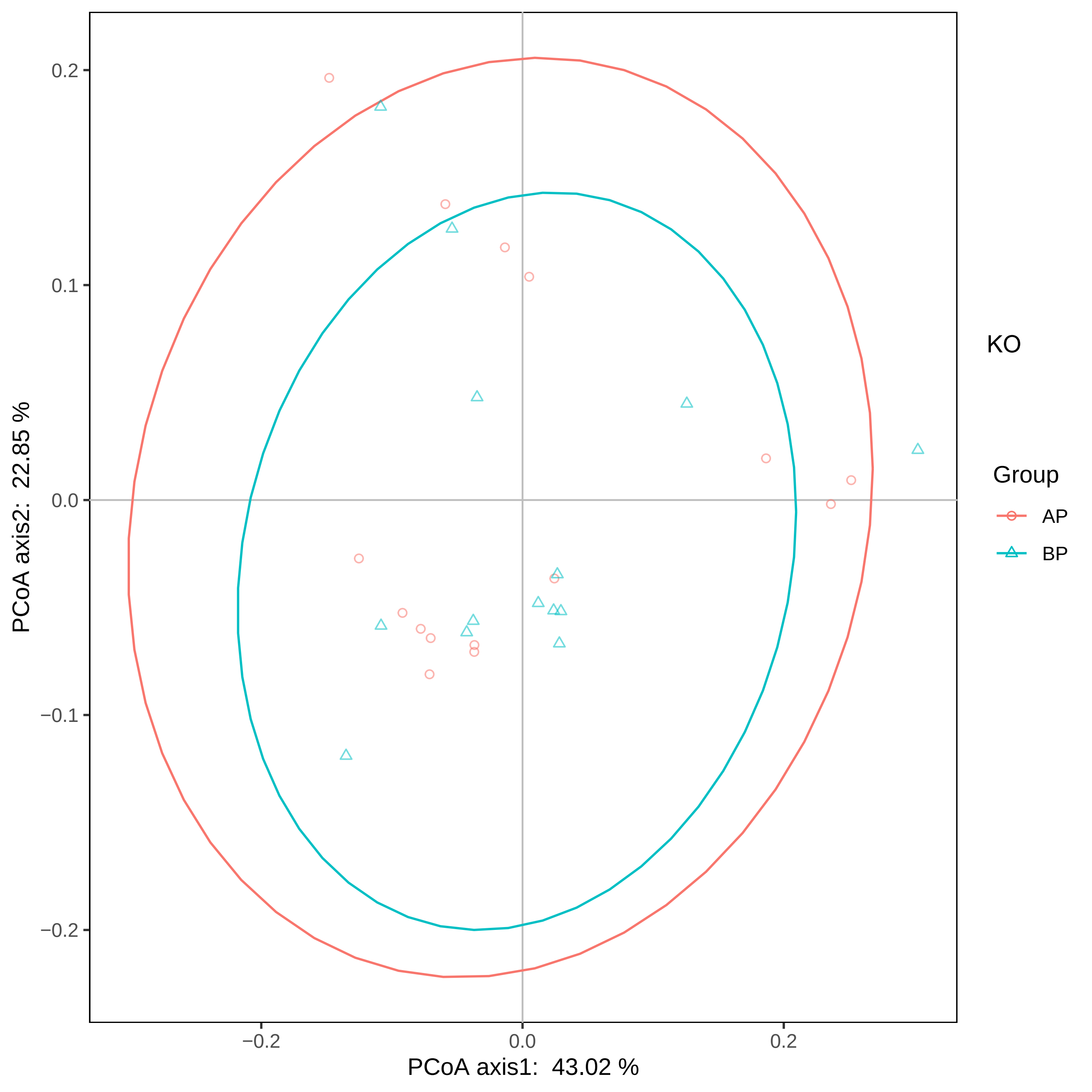

KO
Group
AP
BP
PCoA axis1: 43.02 %
PCoA axis2: 22.85 %
0.2
0.1
0.0
−0.1
−0.2
−0.2
0.0
0.2

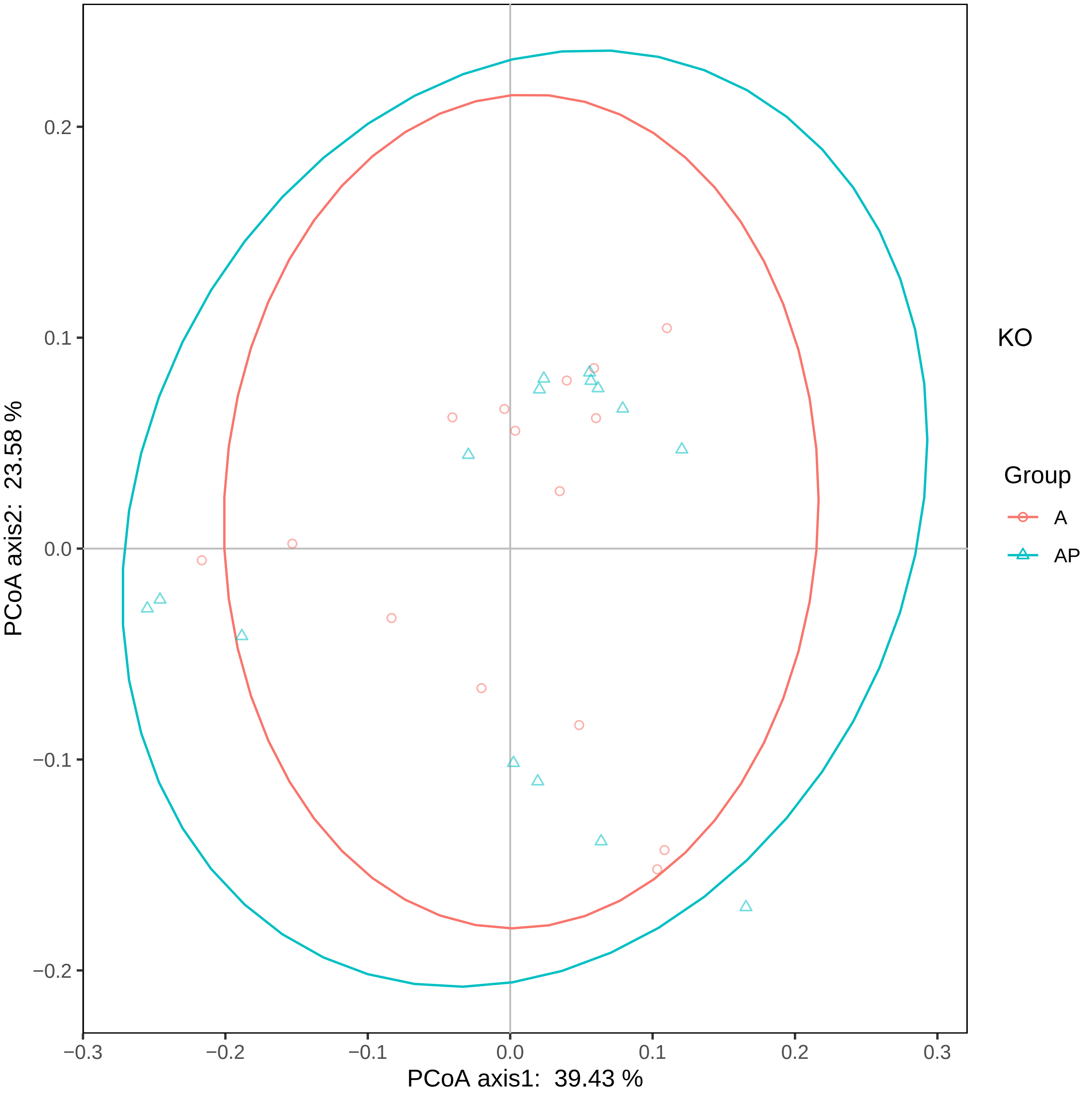

KO
Group
A
AP
PCoA axis1: 39.43 %
PCoA axis2: 23.58 %
−0.3
−0.2
−0.1
0.0
0.1
0.2
0.3
−0.2
−0.1
0.0
0.1
0.2

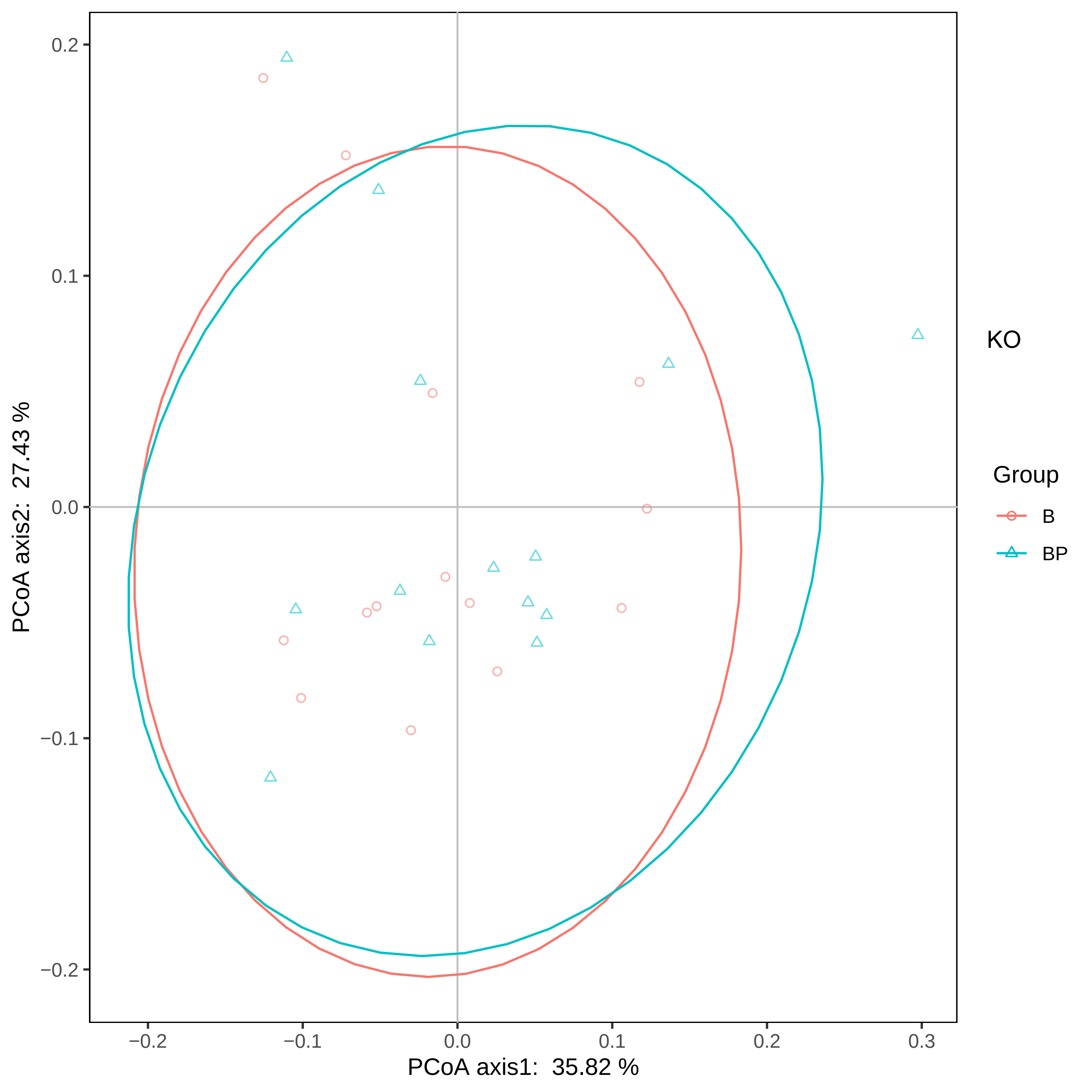

0.2
0.1
0.0
−0.1
−0.2
−0.2
−0.1
0.0
0.1
0.2
0.3
PCoA axis1: 35.82 %
PCoA axis2: 27.43 %
KO
Group
B
BP

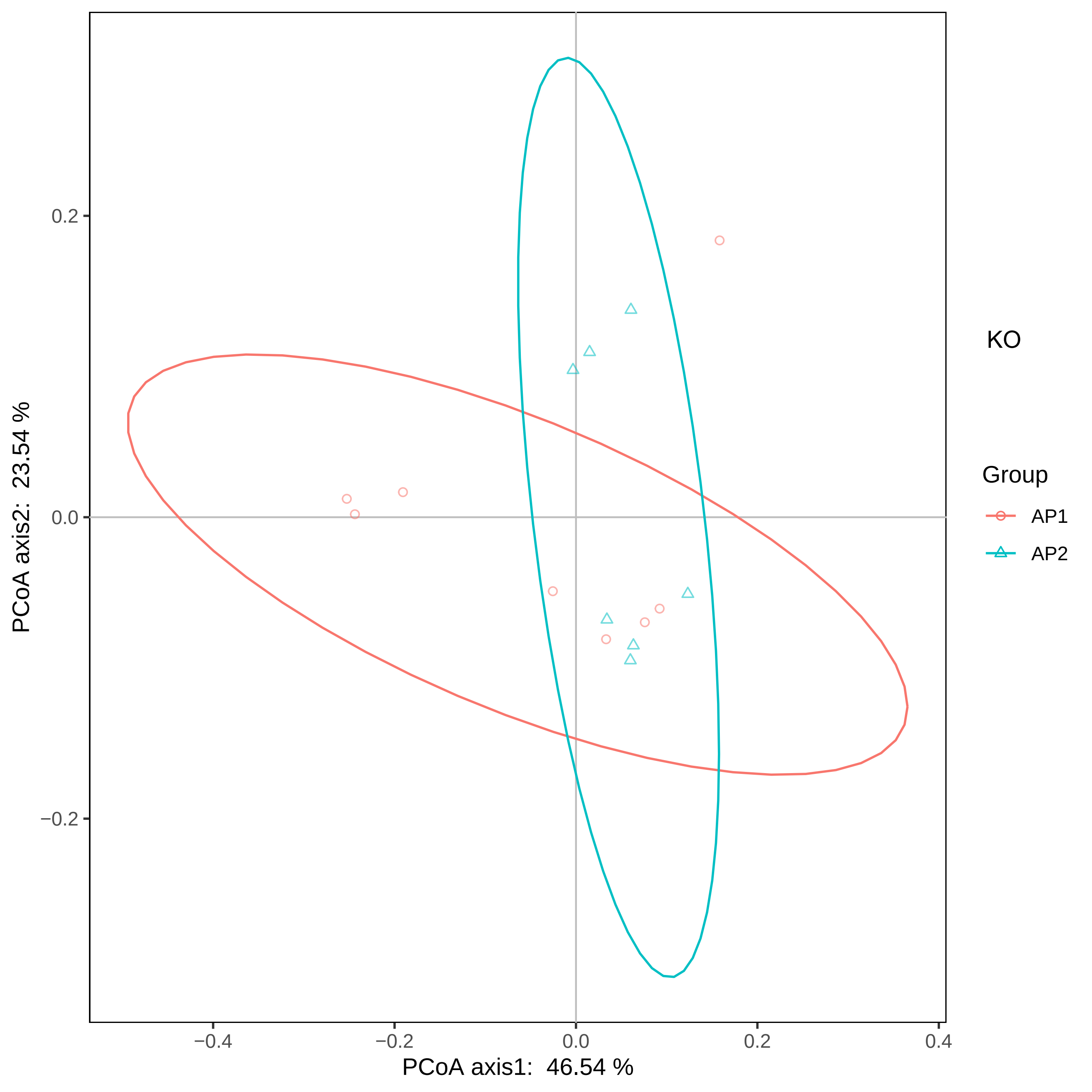

KO
Group
AP1
AP2
PCoA axis1: 46.54 %
PCoA axis2: 23.54 %
−0.4
−0.2
0.0
0.2
0.4

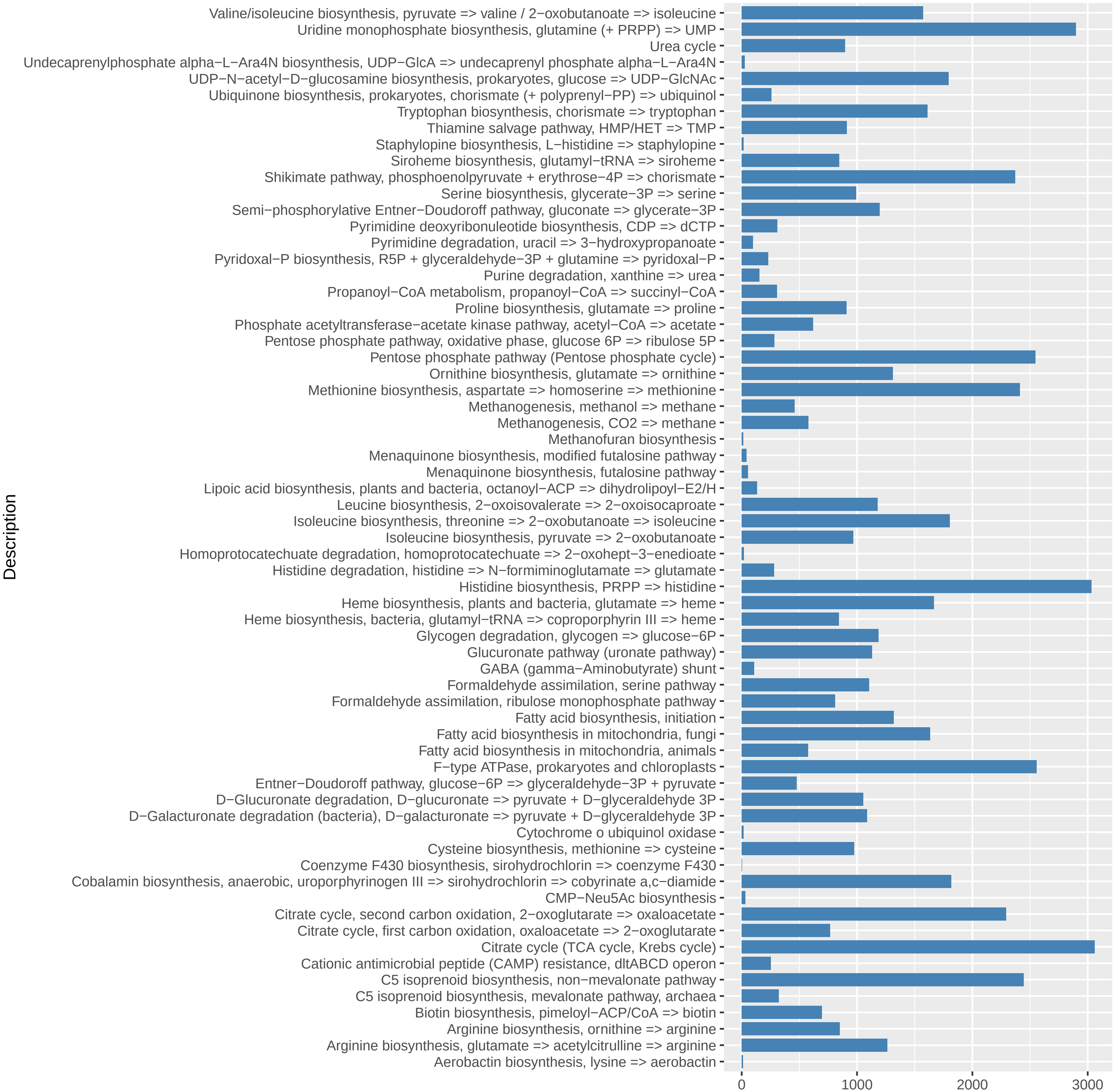

Statistical map of moudle genes

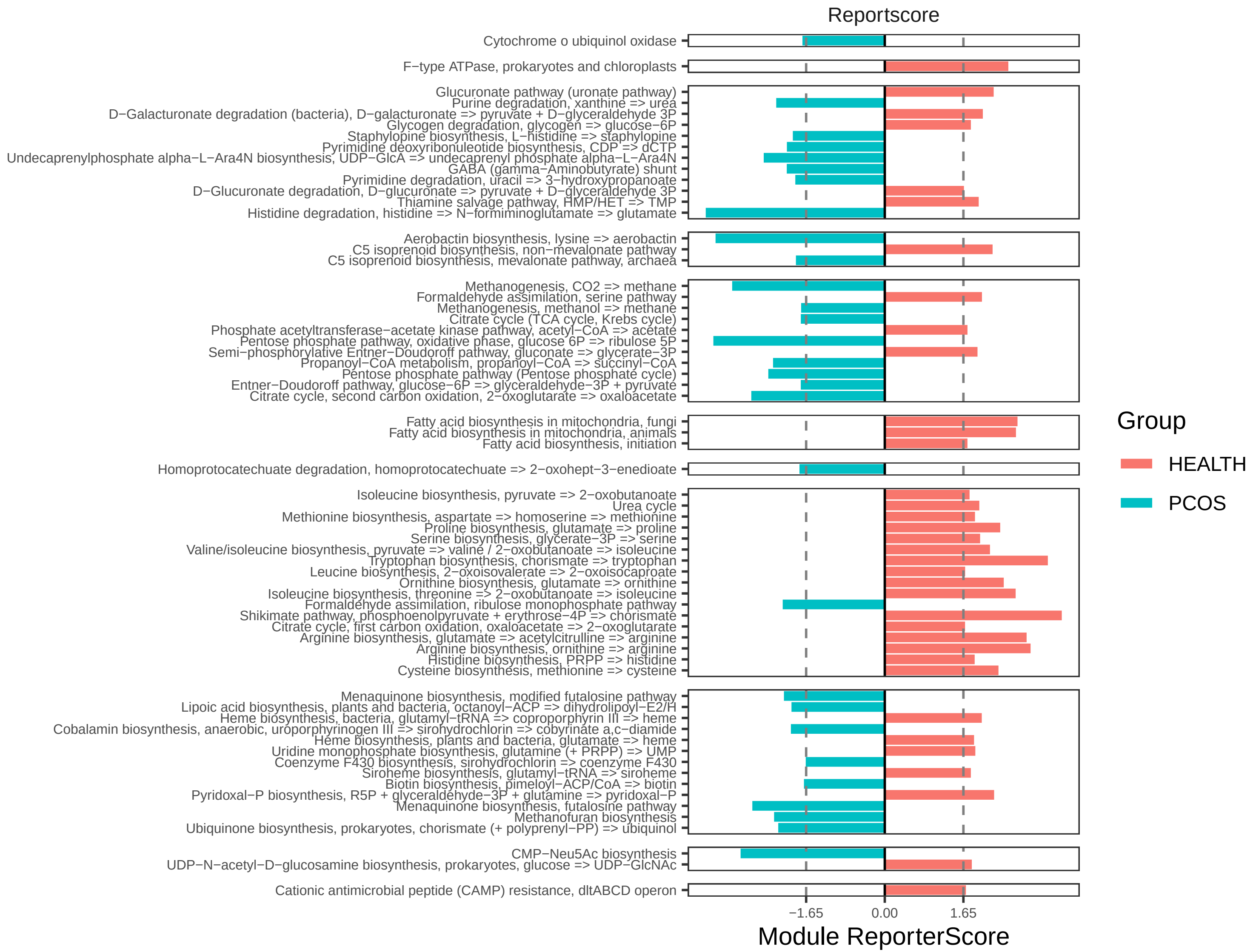

Reportscore
Cytochrome o ubiquinol oxidase
F−type ATPase, prokaryotes and chloroplasts
Glucuronate pathway (uronate pathway)
Purine degradation, xanthine => urea
D−Galacturonate degradation (bacteria), D−galacturonate => pyruvate + D−glyceraldehyde 3P
Glycogen degradation, glycogen => glucose−6P
Staphylopine biosynthesis, L−histidine => staphylopine
Pyrimidine deoxyribonuleotide biosynthesis, CDP => dCTP
Undecaprenylphosphate alpha−L−Ara4N biosynthesis, UDP−GlcA => undecaprenyl phosphate alpha−L−Ara4N
GABA (gamma−Aminobutyrate) shunt
Pyrimidine degradation, uracil => 3−hydroxypropanoate
D−Glucuronate degradation, D−glucuronate => pyruvate + D−glyceraldehyde 3P
Thiamine salvage pathway, HMP/HET => TMP
Histidine degradation, histidine => N−formiminoglutamate => glutamate
Aerobactin biosynthesis, lysine => aerobactin
C5 isoprenoid biosynthesis, non−mevalonate pathway
C5 isoprenoid biosynthesis, mevalonate pathway, archaea
Methanogenesis, CO2 => methane
Formaldehyde assimilation, serine pathway
Methanogenesis, methanol => methane
Citrate cycle (TCA cycle, Krebs cycle)
Phosphate acetyltransferase−acetate kinase pathway, acetyl−CoA => acetate
Pentose phosphate pathway, oxidative phase, glucose 6P => ribulose 5P
Semi−phosphorylative Entner−Doudoroff pathway, gluconate => glycerate−3P
Propanoyl−CoA metabolism, propanoyl−CoA => succinyl−CoA
Pentose phosphate pathway (Pentose phosphate cycle)
Entner−Doudoroff pathway, glucose−6P => glyceraldehyde−3P + pyruvate
Citrate cycle, second carbon oxidation, 2−oxoglutarate => oxaloacetate
Fatty acid biosynthesis in mitochondria, fungi
Fatty acid biosynthesis in mitochondria, animals
Fatty acid biosynthesis, initiation
Homoprotocatechuate degradation, homoprotocatechuate => 2−oxohept−3−enedioate
Isoleucine biosynthesis, pyruvate => 2−oxobutanoate
Urea cycle
Methionine biosynthesis, aspartate => homoserine => methionine
Proline biosynthesis, glutamate => proline
Serine biosynthesis, glycerate−3P => serine
Valine/isoleucine biosynthesis, pyruvate => valine / 2−oxobutanoate => isoleucine
Tryptophan biosynthesis, chorismate => tryptophan
Leucine biosynthesis, 2−oxoisovalerate => 2−oxoisocaproate
Ornithine biosynthesis, glutamate => ornithine
Isoleucine biosynthesis, threonine => 2−oxobutanoate => isoleucine
Formaldehyde assimilation, ribulose monophosphate pathway
Shikimate pathway, phosphoenolpyruvate + erythrose−4P => chorismate
Citrate cycle, first carbon oxidation, oxaloacetate => 2−oxoglutarate
Arginine biosynthesis, glutamate => acetylcitrulline => arginine
Arginine biosynthesis, ornithine => arginine
Histidine biosynthesis, PRPP => histidine
Cysteine biosynthesis, methionine => cysteine
Menaquinone biosynthesis, modified futalosine pathway
Lipoic acid biosynthesis, plants and bacteria, octanoyl−ACP => dihydrolipoyl−E2/H
Heme biosynthesis, bacteria, glutamyl−tRNA => coproporphyrin III => heme
Cobalamin biosynthesis, anaerobic, uroporphyrinogen III => sirohydrochlorin => cobyrinate a,c−diamide
Heme biosynthesis, plants and bacteria, glutamate => heme
Uridine monophosphate biosynthesis, glutamine (+ PRPP) => UMP
Coenzyme F430 biosynthesis, sirohydrochlorin => coenzyme F430
Siroheme biosynthesis, glutamyl−tRNA => siroheme
Biotin biosynthesis, pimeloyl−ACP/CoA => biotin
Pyridoxal−P biosynthesis, R5P + glyceraldehyde−3P + glutamine => pyridoxal−P
Menaquinone biosynthesis, futalosine pathway
Methanofuran biosynthesis
Ubiquinone biosynthesis, prokaryotes, chorismate (+ polyprenyl−PP) => ubiquinol
CMP−Neu5Ac biosynthesis
UDP−N−acetyl−D−glucosamine biosynthesis, prokaryotes, glucose => UDP−GlcNAc
Cationic antimicrobial peptide (CAMP) resistance, dltABCD operon
−1.65
0.00
1.65
Module ReporterScore
Group
HEALTH
PCOS

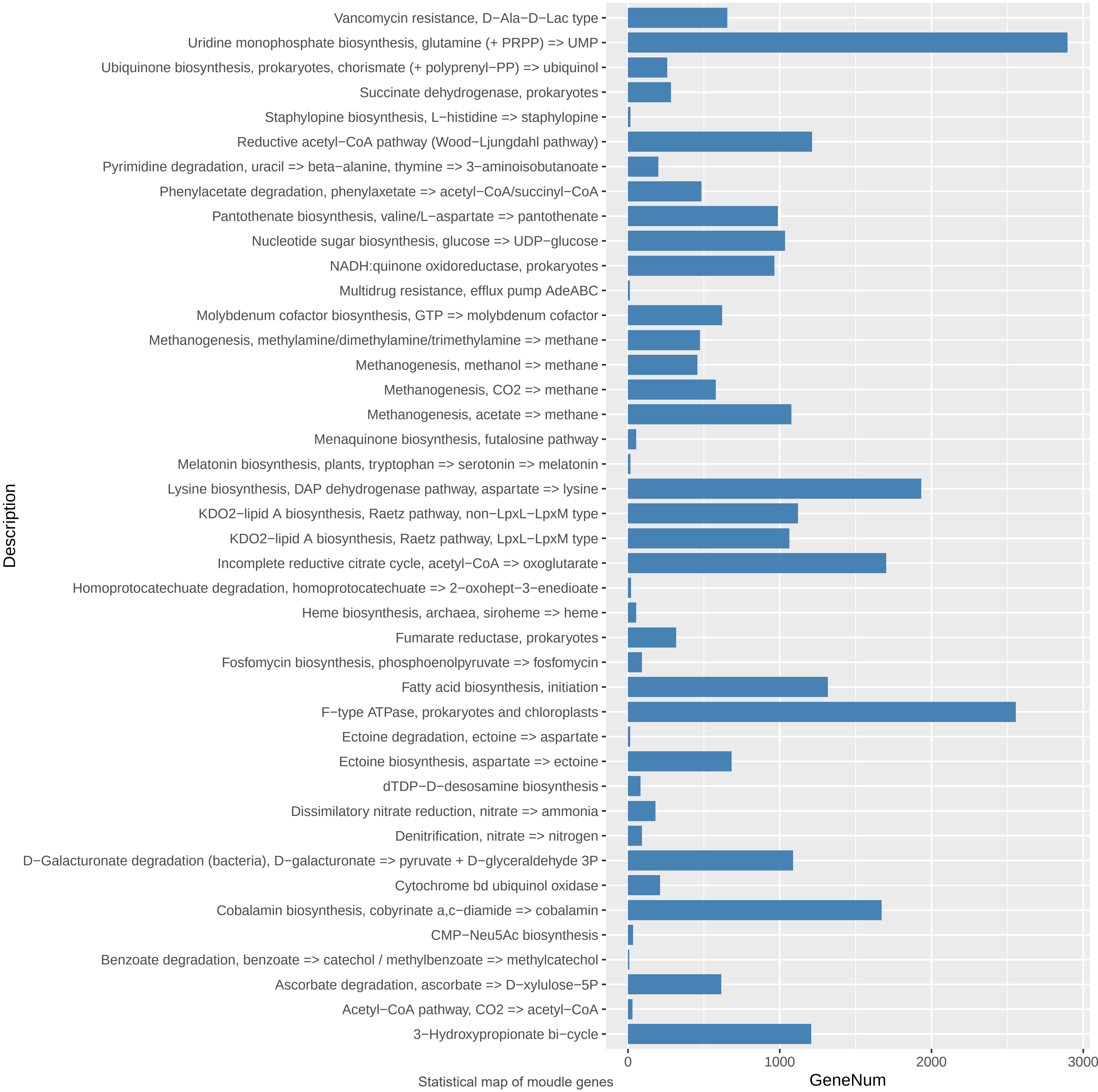

Statistical map of moudle genes

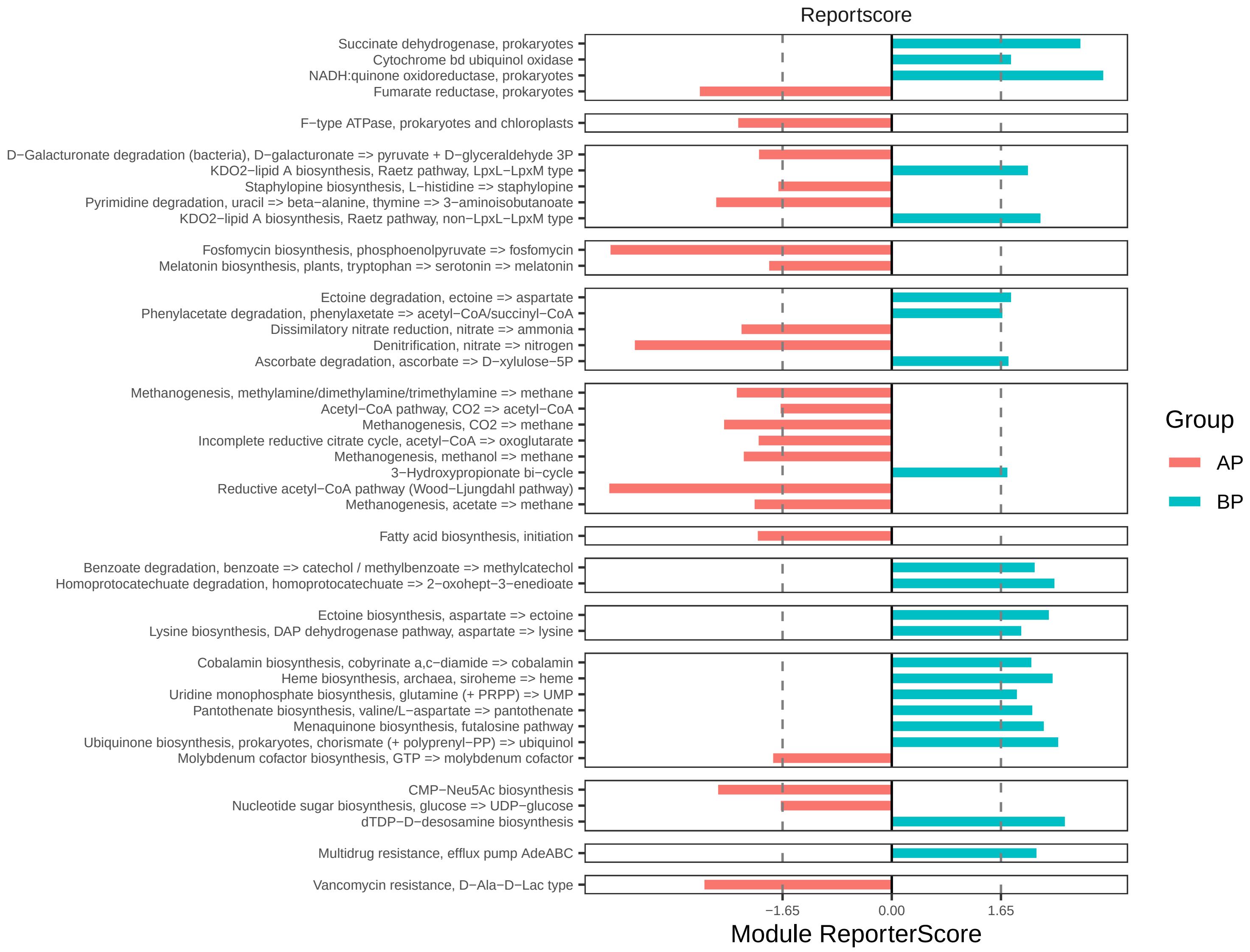
Reportscore
Succinate dehydrogenase, prokaryotes
Cytochrome bd ubiquinol oxidase
NADH:quinone oxidoreductase, prokaryotes
Fumarate reductase, prokaryotes
F−type ATPase, prokaryotes and chloroplasts
D−Galacturonate degradation (bacteria), D−galacturonate => pyruvate + D−glyceraldehyde 3P
KDO2−lipid A biosynthesis, Raetz pathway, LpxL−LpxM type
Staphylopine biosynthesis, L−histidine => staphylopine
Pyrimidine degradation, uracil => beta−alanine, thymine => 3−aminoisobutanoate
KDO2−lipid A biosynthesis, Raetz pathway, non−LpxL−LpxM type
Fosfomycin biosynthesis, phosphoenolpyruvate => fosfomycin
Melatonin biosynthesis, plants, tryptophan => serotonin => melatonin
Ectoine degradation, ectoine => aspartate
Phenylacetate degradation, phenylaxetate => acetyl−CoA/succinyl−CoA
Dissimilatory nitrate reduction, nitrate => ammonia
Denitrification, nitrate => nitrogen
Ascorbate degradation, ascorbate => D−xylulose−5P
Methanogenesis, methylamine/dimethylamine/trimethylamine => methane
Acetyl−CoA pathway, CO2 => acetyl−CoA
Methanogenesis, CO2 => methane
Incomplete reductive citrate cycle, acetyl−CoA => oxoglutarate
Methanogenesis, methanol => methane
3−Hydroxypropionate bi−cycle
Reductive acetyl−CoA pathway (Wood−Ljungdahl pathway)
Methanogenesis, acetate => methane
Fatty acid biosynthesis, initiation
Benzoate degradation, benzoate => catechol / methylbenzoate => methylcatechol
Homoprotocatechuate degradation, homoprotocatechuate => 2−oxohept−3−enedioate
Ectoine biosynthesis, aspartate => ectoine
Lysine biosynthesis, DAP dehydrogenase pathway, aspartate => lysine
Cobalamin biosynthesis, cobyrinate a,c−diamide => cobalamin
Heme biosynthesis, archaea, siroheme => heme
Uridine monophosphate biosynthesis, glutamine (+ PRPP) => UMP
Pantothenate biosynthesis, valine/L−aspartate => pantothenate
Menaquinone biosynthesis, futalosine pathway
Ubiquinone biosynthesis, prokaryotes, chorismate (+ polyprenyl−PP) => ubiquinol
Molybdenum cofactor biosynthesis, GTP => molybdenum cofactor
CMP−Neu5Ac biosynthesis
Nucleotide sugar biosynthesis, glucose => UDP−glucose
dTDP−D−desosamine biosynthesis
Multidrug resistance, efflux pump AdeABC
Vancomycin resistance, D−Ala−D−Lac type
−1.65
0.00
1.65
Module ReporterScore
Group
AP
BP

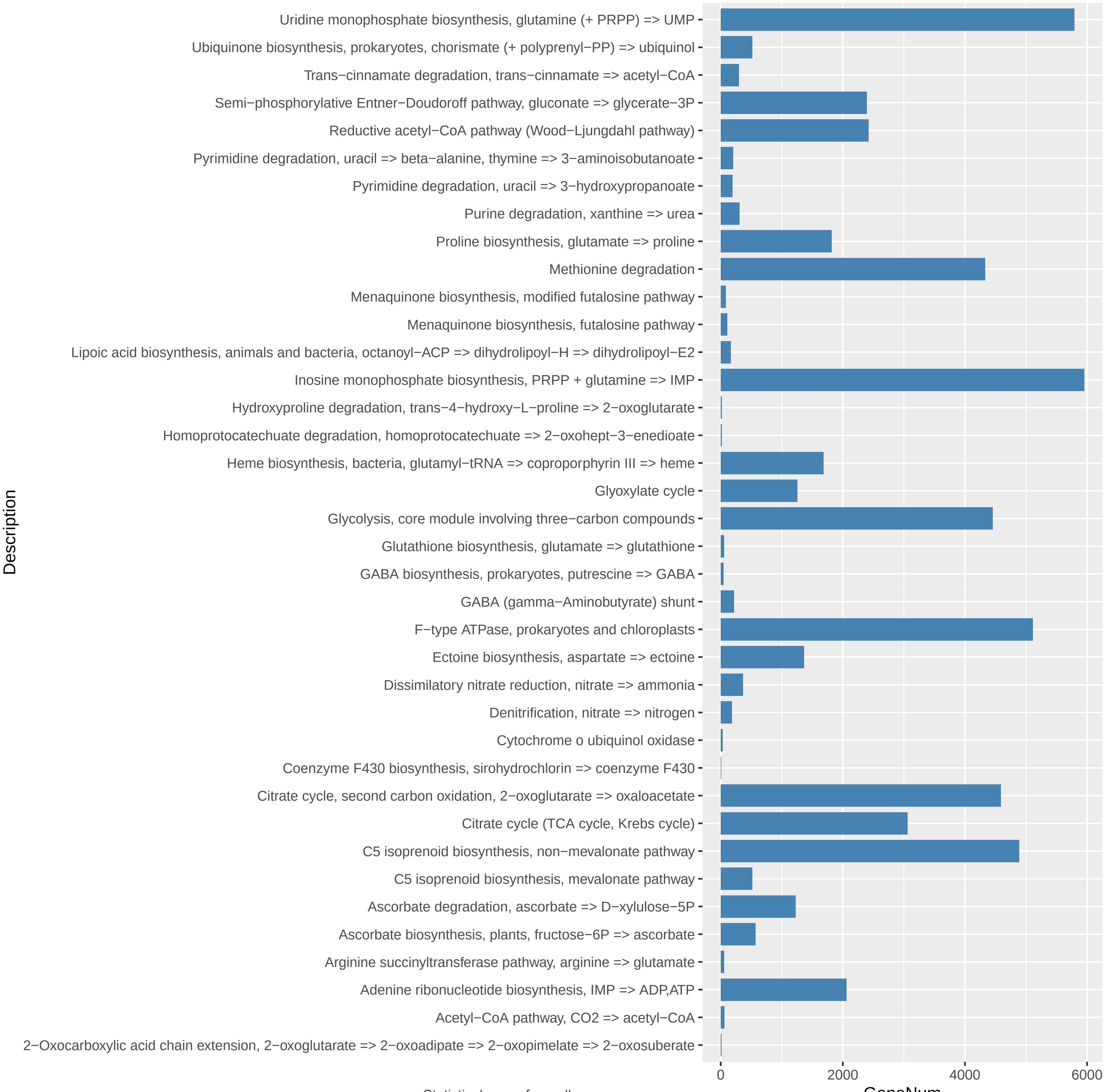

Statistical map of moudle genes

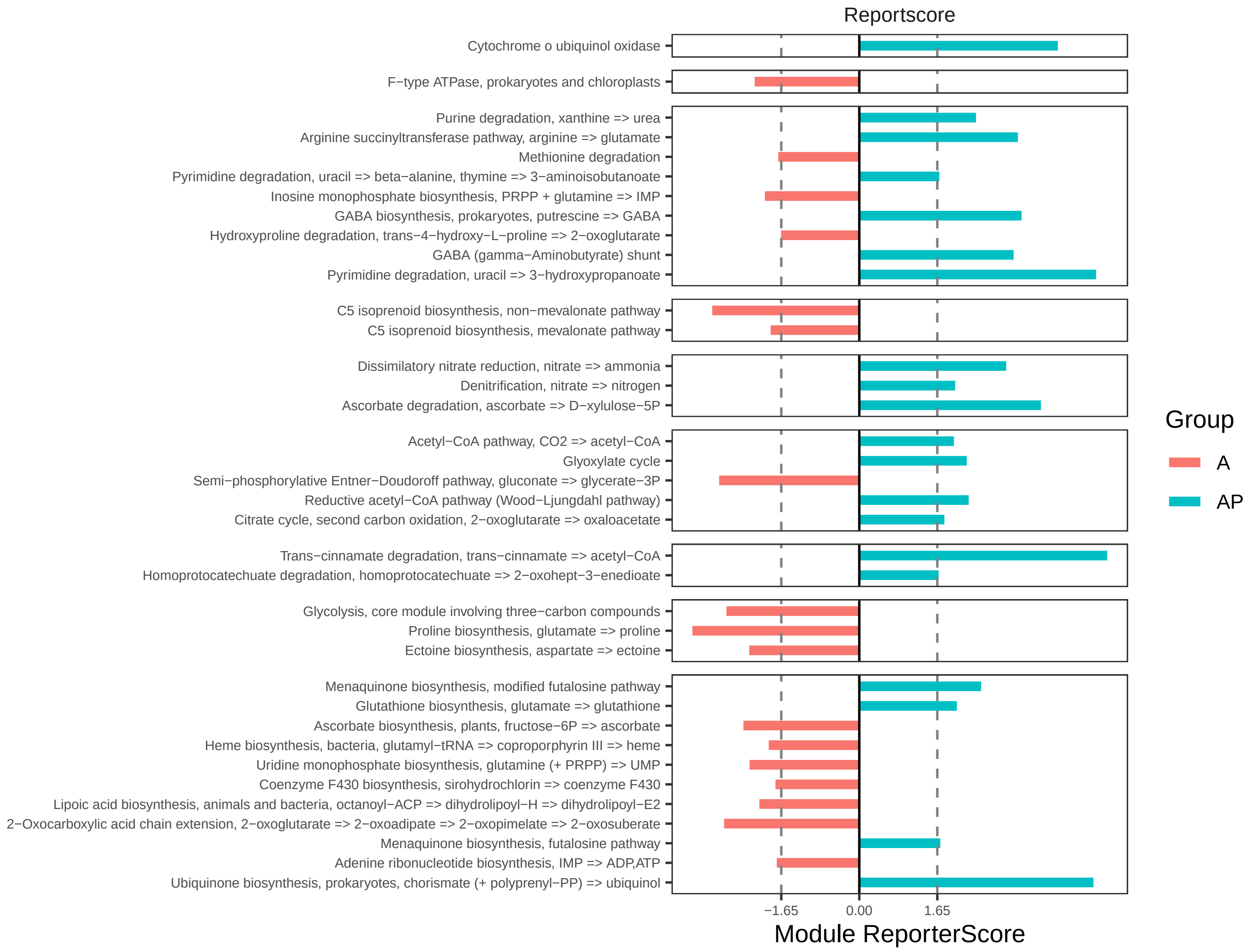
Reportscore
Cytochrome o ubiquinol oxidase
F−type ATPase, prokaryotes and chloroplasts
Purine degradation, xanthine => urea
Arginine succinyltransferase pathway, arginine => glutamate
Methionine degradation
Pyrimidine degradation, uracil => beta−alanine, thymine => 3−aminoisobutanoate
Inosine monophosphate biosynthesis, PRPP + glutamine => IMP
GABA biosynthesis, prokaryotes, putrescine => GABA
Hydroxyproline degradation, trans−4−hydroxy−L−proline => 2−oxoglutarate
GABA (gamma−Aminobutyrate) shunt
Pyrimidine degradation, uracil => 3−hydroxypropanoate
C5 isoprenoid biosynthesis, non−mevalonate pathway
C5 isoprenoid biosynthesis, mevalonate pathway
Dissimilatory nitrate reduction, nitrate => ammonia
Denitrification, nitrate => nitrogen
Ascorbate degradation, ascorbate => D−xylulose−5P
Acetyl−CoA pathway, CO2 => acetyl−CoA
Glyoxylate cycle
Semi−phosphorylative Entner−Doudoroff pathway, gluconate => glycerate−3P
Reductive acetyl−CoA pathway (Wood−Ljungdahl pathway)
Citrate cycle, second carbon oxidation, 2−oxoglutarate => oxaloacetate
Trans−cinnamate degradation, trans−cinnamate => acetyl−CoA
Homoprotocatechuate degradation, homoprotocatechuate => 2−oxohept−3−enedioate
Glycolysis, core module involving three−carbon compounds
Proline biosynthesis, glutamate => proline
Ectoine biosynthesis, aspartate => ectoine
Menaquinone biosynthesis, modified futalosine pathway
Glutathione biosynthesis, glutamate => glutathione
Ascorbate biosynthesis, plants, fructose−6P => ascorbate
Heme biosynthesis, bacteria, glutamyl−tRNA => coproporphyrin III => heme
Uridine monophosphate biosynthesis, glutamine (+ PRPP) => UMP
Coenzyme F430 biosynthesis, sirohydrochlorin => coenzyme F430
Lipoic acid biosynthesis, animals and bacteria, octanoyl−ACP => dihydrolipoyl−H => dihydrolipoyl−E2
2−Oxocarboxylic acid chain extension, 2−oxoglutarate => 2−oxoadipate => 2−oxopimelate => 2−oxosuberate
Menaquinone biosynthesis, futalosine pathway
Adenine ribonucleotide biosynthesis, IMP => ADP,ATP
Ubiquinone biosynthesis, prokaryotes, chorismate (+ polyprenyl−PP) => ubiquinol
−1.65
0.00
1.65
Module ReporterScore
Group
A
AP

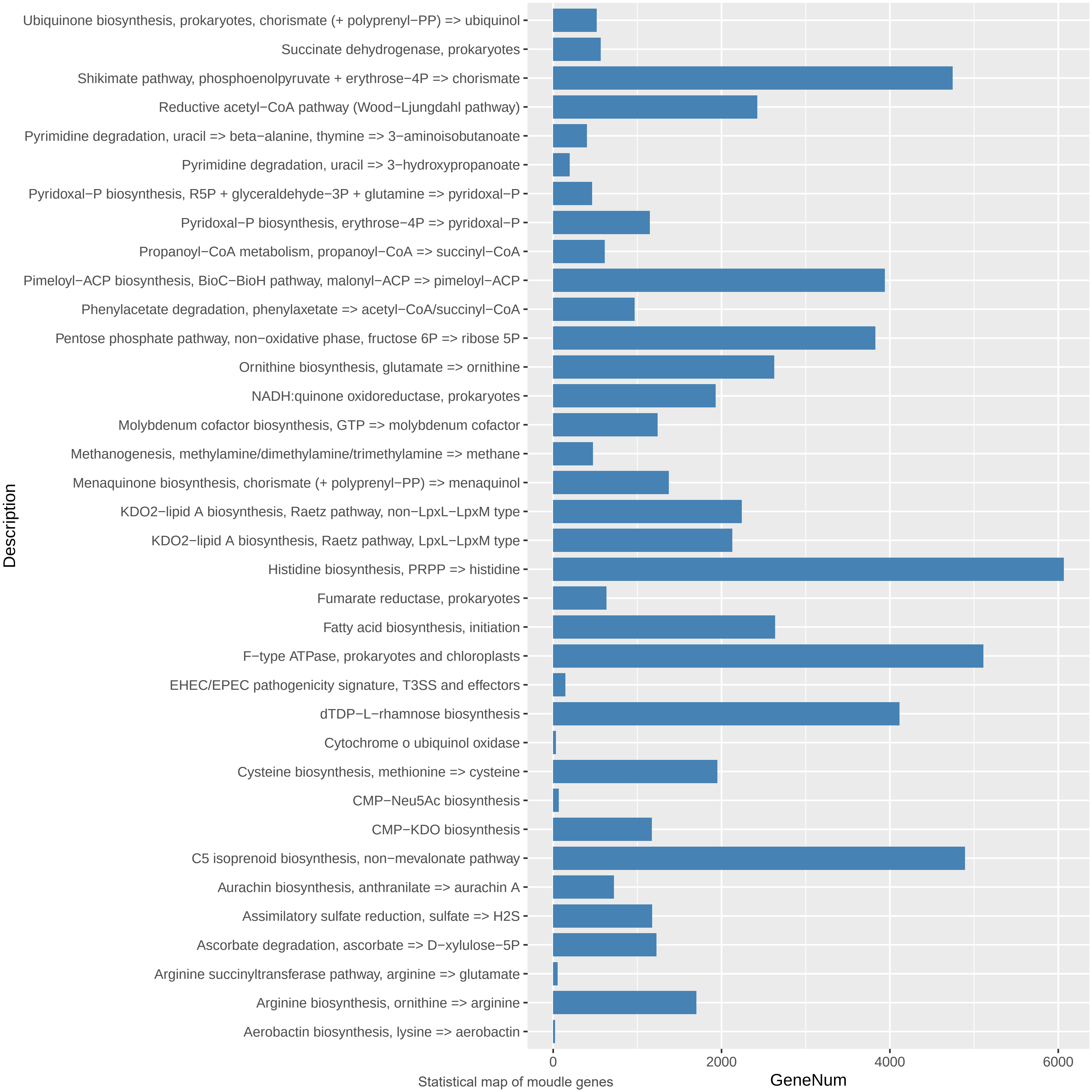

Statistical map of moudle genes

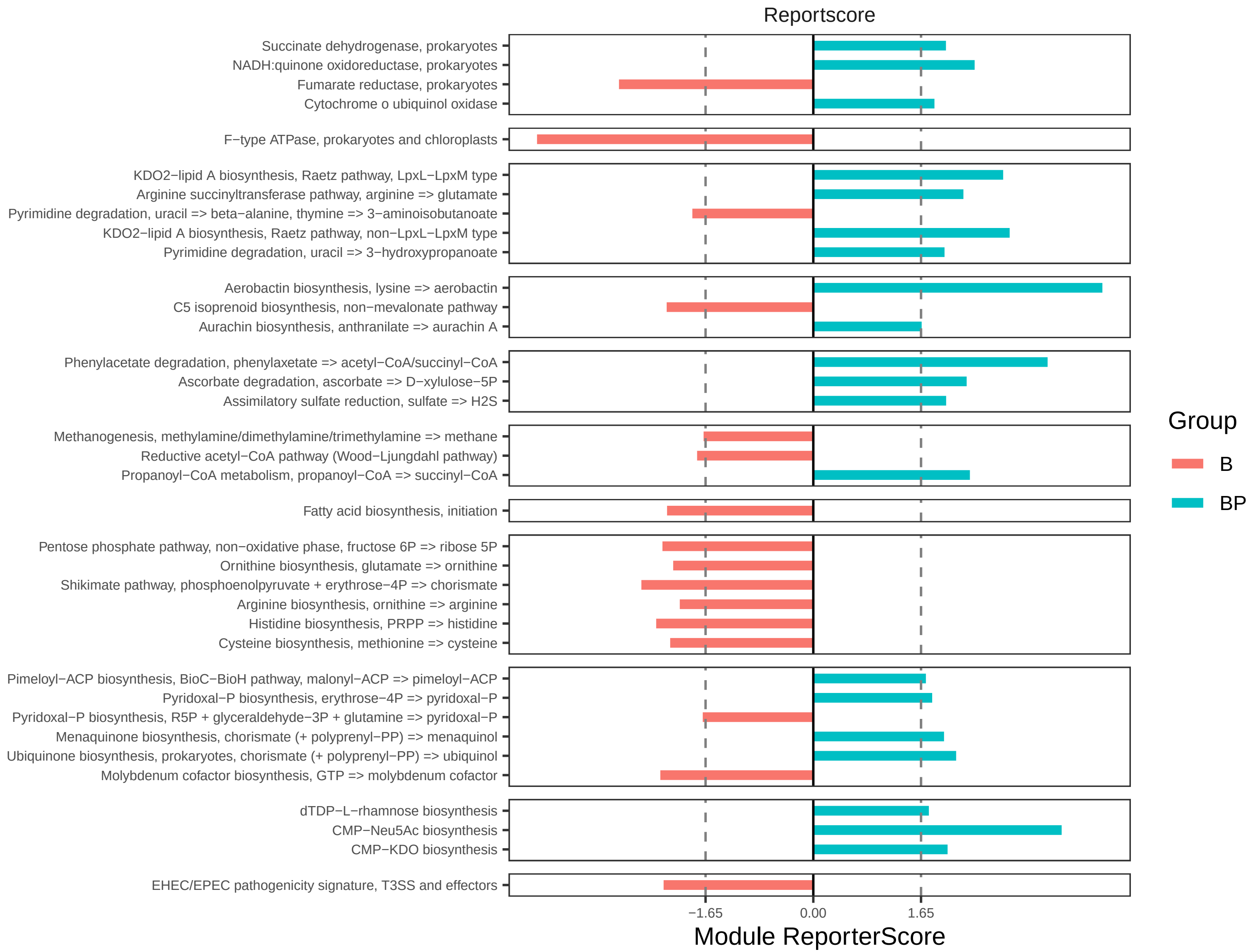

Reportscore
Succinate dehydrogenase, prokaryotes
NADH:quinone oxidoreductase, prokaryotes
Fumarate reductase, prokaryotes
Cytochrome o ubiquinol oxidase
F−type ATPase, prokaryotes and chloroplasts
KDO2−lipid A biosynthesis, Raetz pathway, LpxL−LpxM type
Arginine succinyltransferase pathway, arginine => glutamate
Pyrimidine degradation, uracil => beta−alanine, thymine => 3−aminoisobutanoate
KDO2−lipid A biosynthesis, Raetz pathway, non−LpxL−LpxM type
Pyrimidine degradation, uracil => 3−hydroxypropanoate
Aerobactin biosynthesis, lysine => aerobactin
C5 isoprenoid biosynthesis, non−mevalonate pathway
Aurachin biosynthesis, anthranilate => aurachin A
Phenylacetate degradation, phenylaxetate => acetyl−CoA/succinyl−CoA
Ascorbate degradation, ascorbate => D−xylulose−5P
Assimilatory sulfate reduction, sulfate => H2S
Methanogenesis, methylamine/dimethylamine/trimethylamine => methane
Reductive acetyl−CoA pathway (Wood−Ljungdahl pathway)
Propanoyl−CoA metabolism, propanoyl−CoA => succinyl−CoA
Fatty acid biosynthesis, initiation
Pentose phosphate pathway, non−oxidative phase, fructose 6P => ribose 5P
Ornithine biosynthesis, glutamate => ornithine
Shikimate pathway, phosphoenolpyruvate + erythrose−4P => chorismate
Arginine biosynthesis, ornithine => arginine
Histidine biosynthesis, PRPP => histidine
Cysteine biosynthesis, methionine => cysteine
Pimeloyl−ACP biosynthesis, BioC−BioH pathway, malonyl−ACP => pimeloyl−ACP
Pyridoxal−P biosynthesis, erythrose−4P => pyridoxal−P
Pyridoxal−P biosynthesis, R5P + glyceraldehyde−3P + glutamine => pyridoxal−P
Menaquinone biosynthesis, chorismate (+ polyprenyl−PP) => menaquinol
Ubiquinone biosynthesis, prokaryotes, chorismate (+ polyprenyl−PP) => ubiquinol
Molybdenum cofactor biosynthesis, GTP => molybdenum cofactor
dTDP−L−rhamnose biosynthesis
CMP−Neu5Ac biosynthesis
CMP−KDO biosynthesis
EHEC/EPEC pathogenicity signature, T3SS and effectors
−1.65
0.00
1.65
Module ReporterScore
Group
B
BP

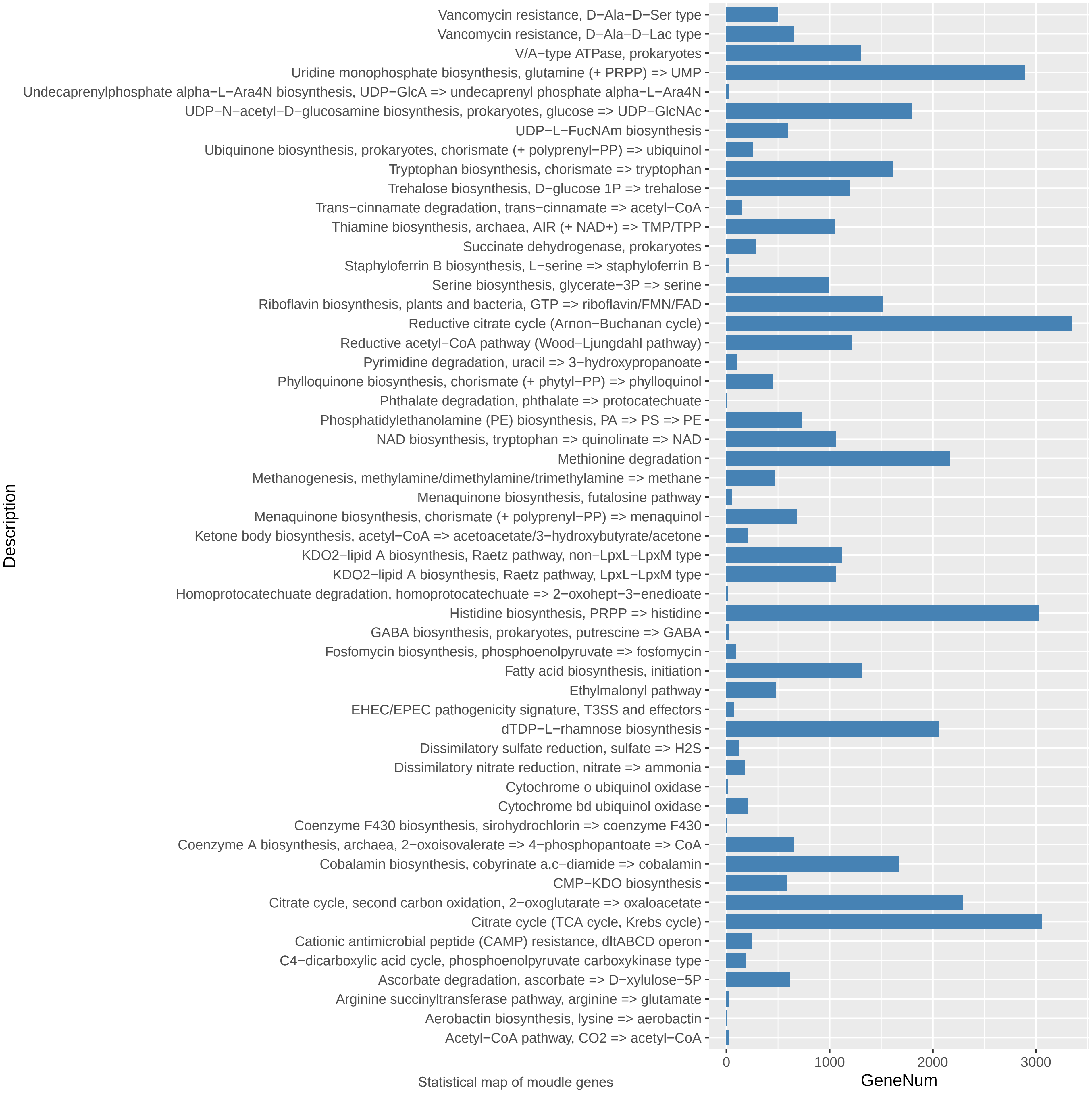

Statistical map of moudle genes

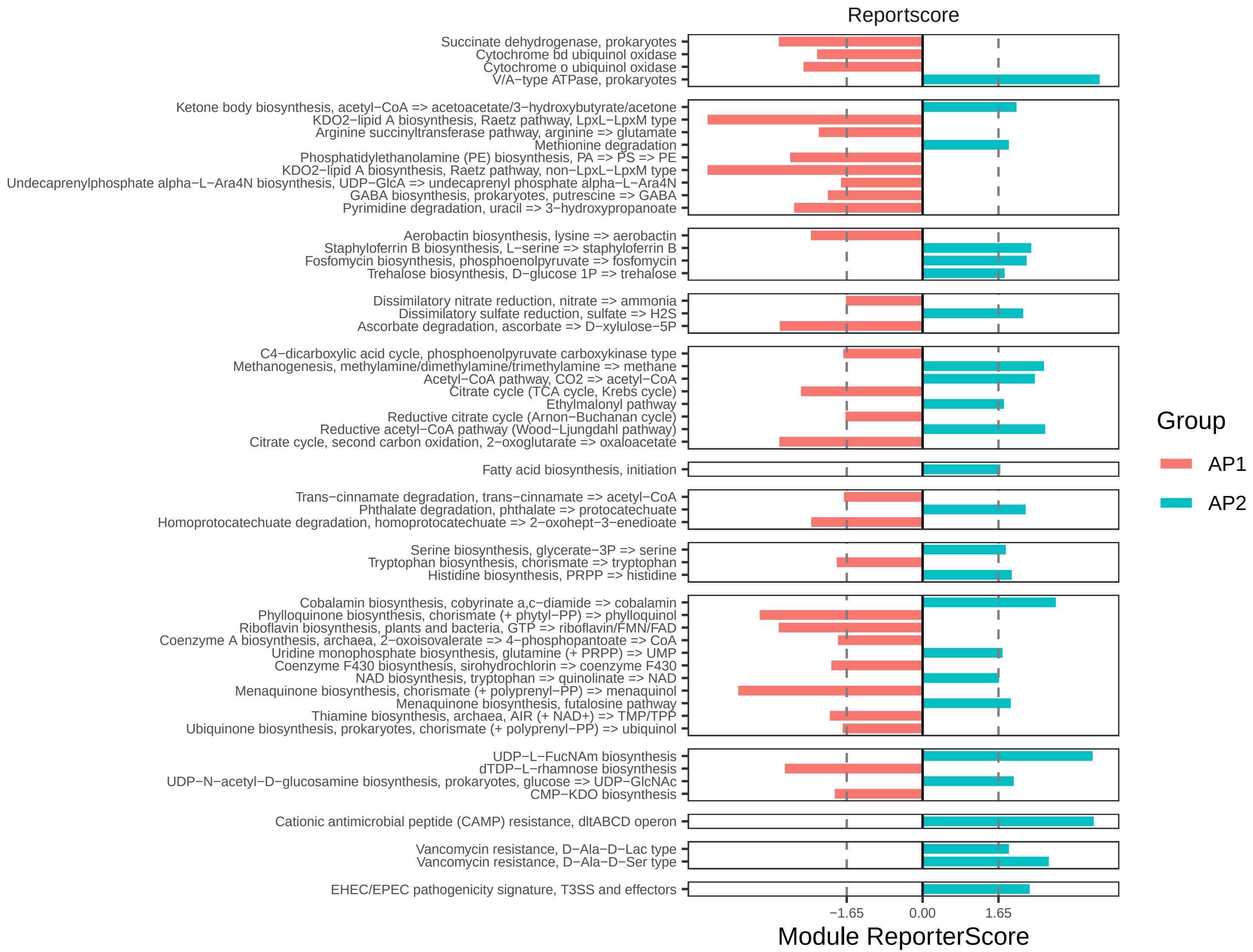

Reportscore
Succinate dehydrogenase, prokaryotes
Cytochrome bd ubiquinol oxidase
Cytochrome o ubiquinol oxidase
V/A−type ATPase, prokaryotes
Ketone body biosynthesis, acetyl−CoA => acetoacetate/3−hydroxybutyrate/acetone
KDO2−lipid A biosynthesis, Raetz pathway, LpxL−LpxM type
Arginine succinyltransferase pathway, arginine => glutamate
Methionine degradation
Phosphatidylethanolamine (PE) biosynthesis, PA => PS => PE
KDO2−lipid A biosynthesis, Raetz pathway, non−LpxL−LpxM type
Undecaprenylphosphate alpha−L−Ara4N biosynthesis, UDP−GlcA => undecaprenyl phosphate alpha−L−Ara4N
GABA biosynthesis, prokaryotes, putrescine => GABA
Pyrimidine degradation, uracil => 3−hydroxypropanoate
Aerobactin biosynthesis, lysine => aerobactin
Staphyloferrin B biosynthesis, L−serine => staphyloferrin B
Fosfomycin biosynthesis, phosphoenolpyruvate => fosfomycin
Trehalose biosynthesis, D−glucose 1P => trehalose
Dissimilatory nitrate reduction, nitrate => ammonia
Dissimilatory sulfate reduction, sulfate => H2S
Ascorbate degradation, ascorbate => D−xylulose−5P
C4−dicarboxylic acid cycle, phosphoenolpyruvate carboxykinase type
Methanogenesis, methylamine/dimethylamine/trimethylamine => methane
Acetyl−CoA pathway, CO2 => acetyl−CoA
Citrate cycle (TCA cycle, Krebs cycle)
Ethylmalonyl pathway
Reductive citrate cycle (Arnon−Buchanan cycle)
Reductive acetyl−CoA pathway (Wood−Ljungdahl pathway)
Citrate cycle, second carbon oxidation, 2−oxoglutarate => oxaloacetate
Fatty acid biosynthesis, initiation
Trans−cinnamate degradation, trans−cinnamate => acetyl−CoA
Phthalate degradation, phthalate => protocatechuate
Homoprotocatechuate degradation, homoprotocatechuate => 2−oxohept−3−enedioate
Serine biosynthesis, glycerate−3P => serine
Tryptophan biosynthesis, chorismate => tryptophan
Histidine biosynthesis, PRPP => histidine
Cobalamin biosynthesis, cobyrinate a,c−diamide => cobalamin
Phylloquinone biosynthesis, chorismate (+ phytyl−PP) => phylloquinol
Riboflavin biosynthesis, plants and bacteria, GTP => riboflavin/FMN/FAD
Coenzyme A biosynthesis, archaea, 2−oxoisovalerate => 4−phosphopantoate => CoA
Uridine monophosphate biosynthesis, glutamine (+ PRPP) => UMP
Coenzyme F430 biosynthesis, sirohydrochlorin => coenzyme F430
NAD biosynthesis, tryptophan => quinolinate => NAD
Menaquinone biosynthesis, chorismate (+ polyprenyl−PP) => menaquinol
Menaquinone biosynthesis, futalosine pathway
Thiamine biosynthesis, archaea, AIR (+ NAD+) => TMP/TPP
Ubiquinone biosynthesis, prokaryotes, chorismate (+ polyprenyl−PP) => ubiquinol
UDP−L−FucNAm biosynthesis
dTDP−L−rhamnose biosynthesis
UDP−N−acetyl−D−glucosamine biosynthesis, prokaryotes, glucose => UDP−GlcNAc
CMP−KDO biosynthesis
Cationic antimicrobial peptide (CAMP) resistance, dltABCD operon
Vancomycin resistance, D−Ala−D−Lac type
Vancomycin resistance, D−Ala−D−Ser type
EHEC/EPEC pathogenicity signature, T3SS and effectors
−1.65
0.00
1.65
Module ReporterScore
Group
AP1
AP2

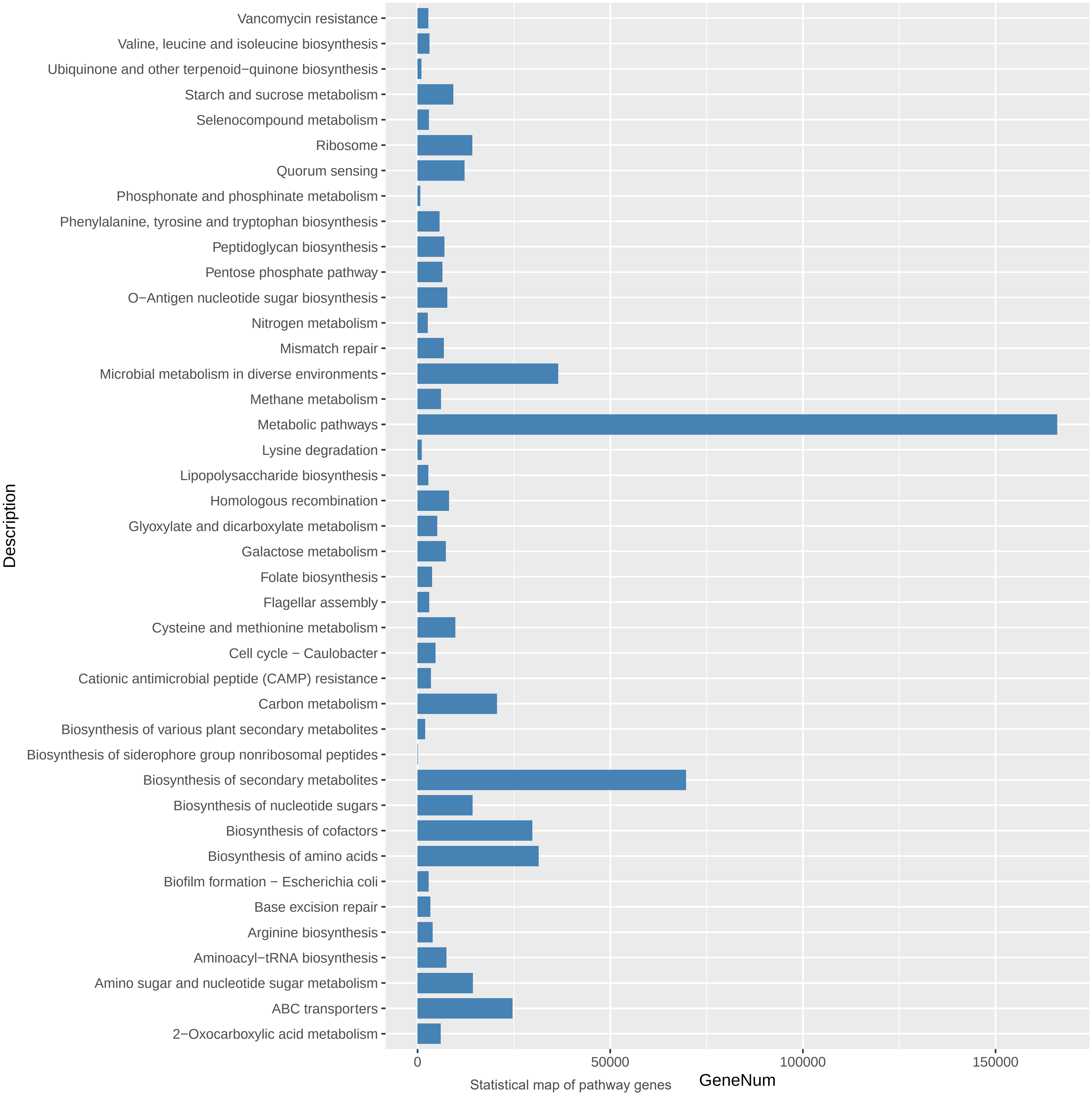

Statistical map of pathway genes

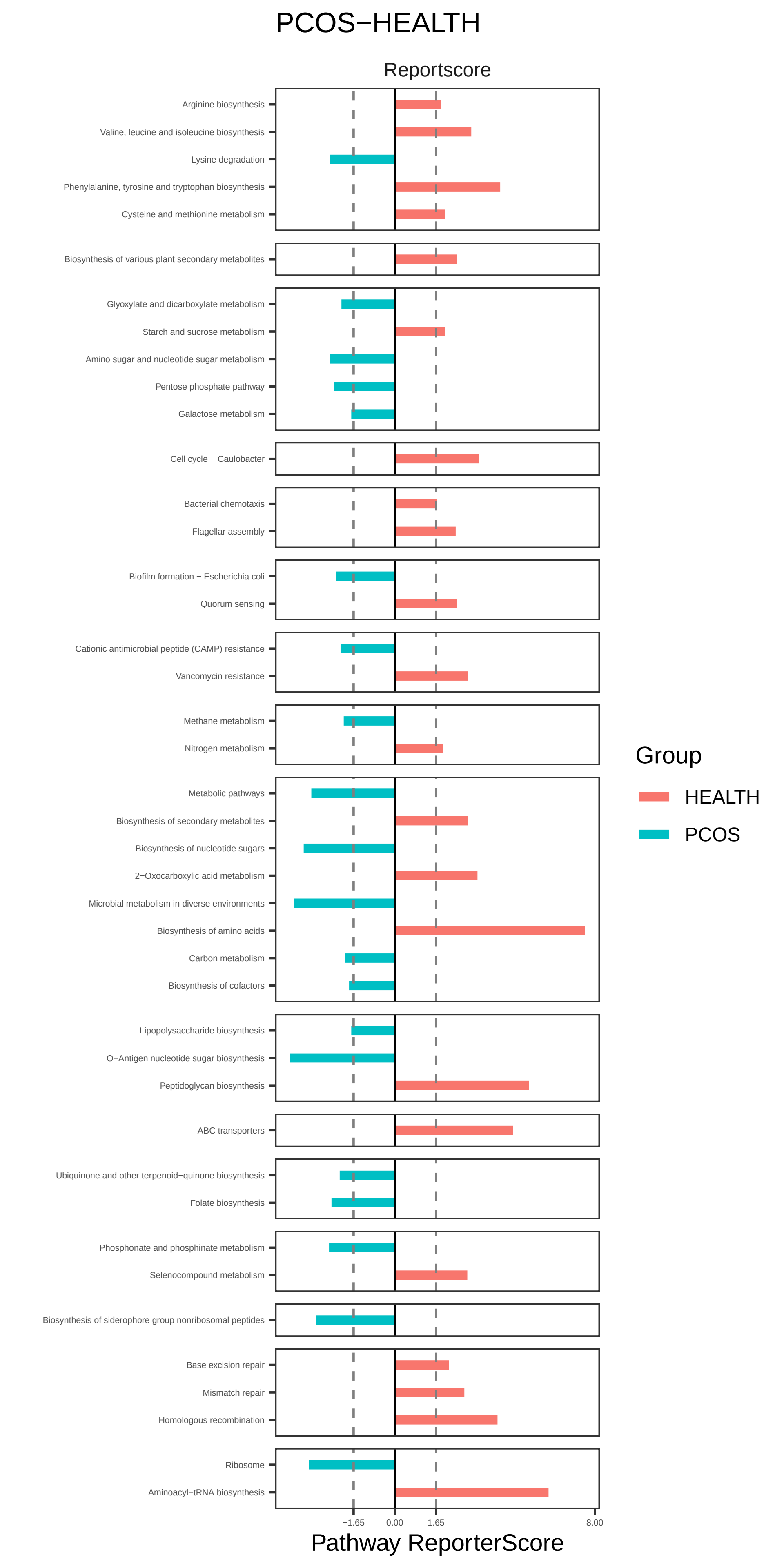
PCOS−HEALTH
Reportscore
Arginine biosynthesis
Valine, leucine and isoleucine biosynthesis
Lysine degradation
Phenylalanine, tyrosine and tryptophan biosynthesis
Cysteine and methionine metabolism
Biosynthesis of various plant secondary metabolites
Glyoxylate and dicarboxylate metabolism
Starch and sucrose metabolism
Amino sugar and nucleotide sugar metabolism
Pentose phosphate pathway
Galactose metabolism
Cell cycle − Caulobacter
Bacterial chemotaxis
Flagellar assembly
Biofilm formation − Escherichia coli
Quorum sensing
Cationic antimicrobial peptide (CAMP) resistance
Vancomycin resistance
Methane metabolism
Nitrogen metabolism
Metabolic pathways
Biosynthesis of secondary metabolites
Biosynthesis of nucleotide sugars
2−Oxocarboxylic acid metabolism
Microbial metabolism in diverse environments
Biosynthesis of amino acids
Carbon metabolism
Biosynthesis of cofactors
Lipopolysaccharide biosynthesis
O−Antigen nucleotide sugar biosynthesis
Peptidoglycan biosynthesis
ABC transporters
Ubiquinone and other terpenoid−quinone biosynthesis
Folate biosynthesis
Phosphonate and phosphinate metabolism
Selenocompound metabolism
Biosynthesis of siderophore group nonribosomal peptides
Base excision repair
Mismatch repair
Homologous recombination
Ribosome
Aminoacyl−tRNA biosynthesis
−1.65
0.00
1.65
8.00
Pathway ReporterScore
Group
HEALTH
PCOS

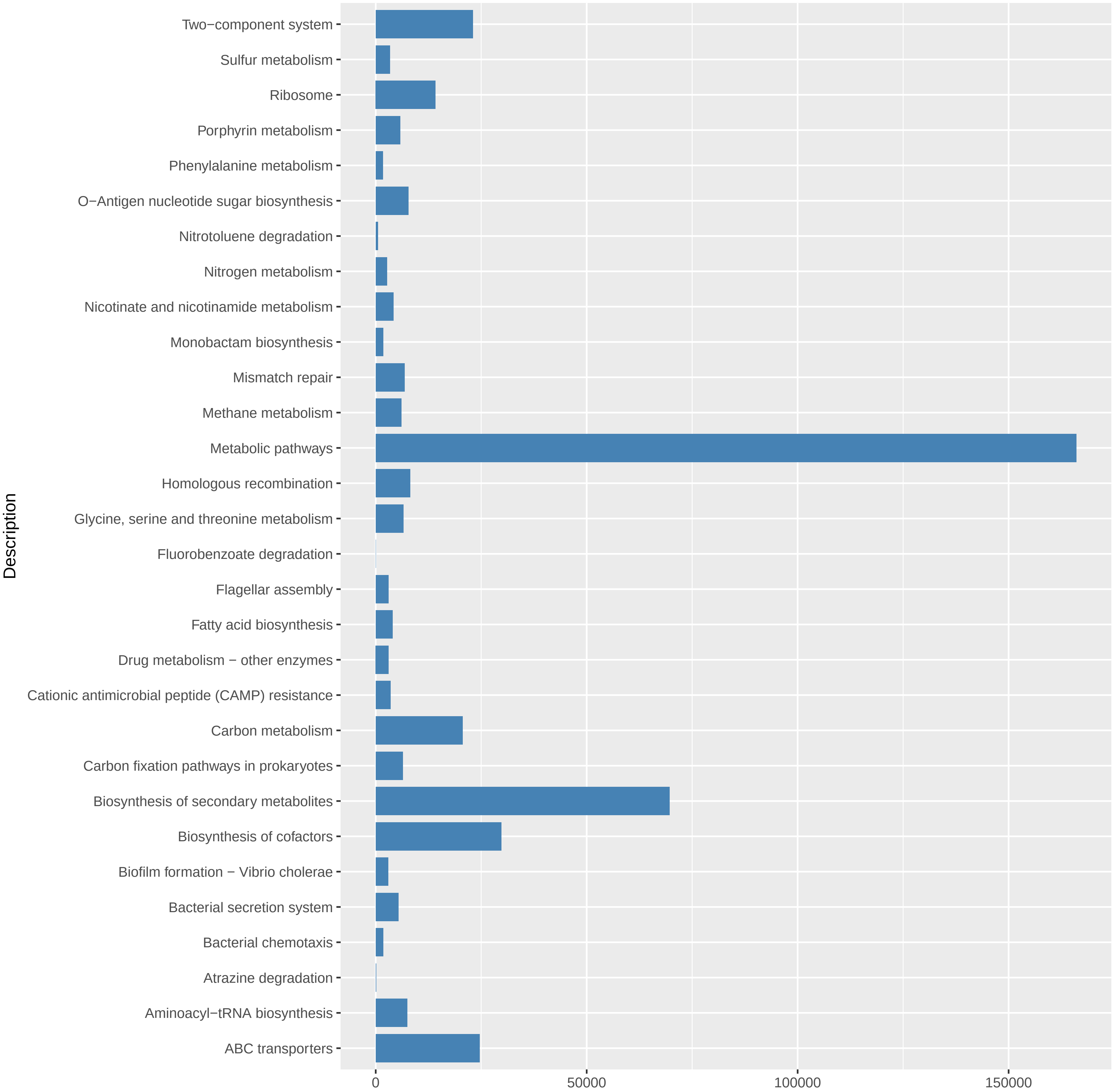

Statistical map of pathway genes

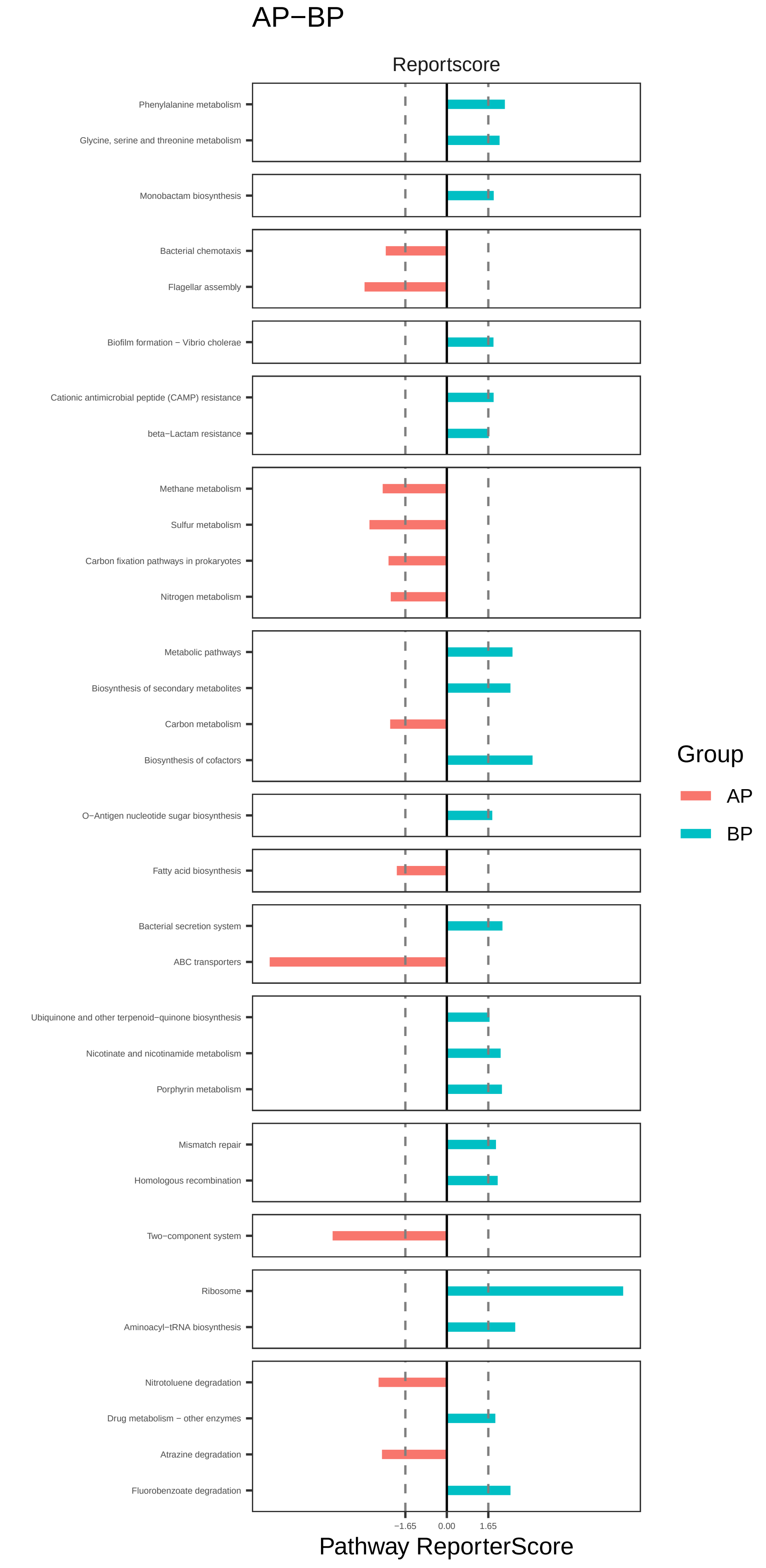
AP−BP
Reportscore
Phenylalanine metabolism
Glycine, serine and threonine metabolism
Monobactam biosynthesis
Bacterial chemotaxis
Flagellar assembly
Biofilm formation − Vibrio cholerae
Cationic antimicrobial peptide (CAMP) resistance
beta−Lactam resistance
Methane metabolism
Sulfur metabolism
Carbon fixation pathways in prokaryotes
Nitrogen metabolism
Metabolic pathways
Biosynthesis of secondary metabolites
Carbon metabolism
Biosynthesis of cofactors
O−Antigen nucleotide sugar biosynthesis
Fatty acid biosynthesis
Bacterial secretion system
ABC transporters
Ubiquinone and other terpenoid−quinone biosynthesis
Nicotinate and nicotinamide metabolism
Porphyrin metabolism
Mismatch repair
Homologous recombination
Two−component system
Ribosome
Aminoacyl−tRNA biosynthesis
Nitrotoluene degradation
Drug metabolism − other enzymes
Atrazine degradation
Fluorobenzoate degradation
−1.65
0.00
1.65
Pathway ReporterScore
Group
AP
BP

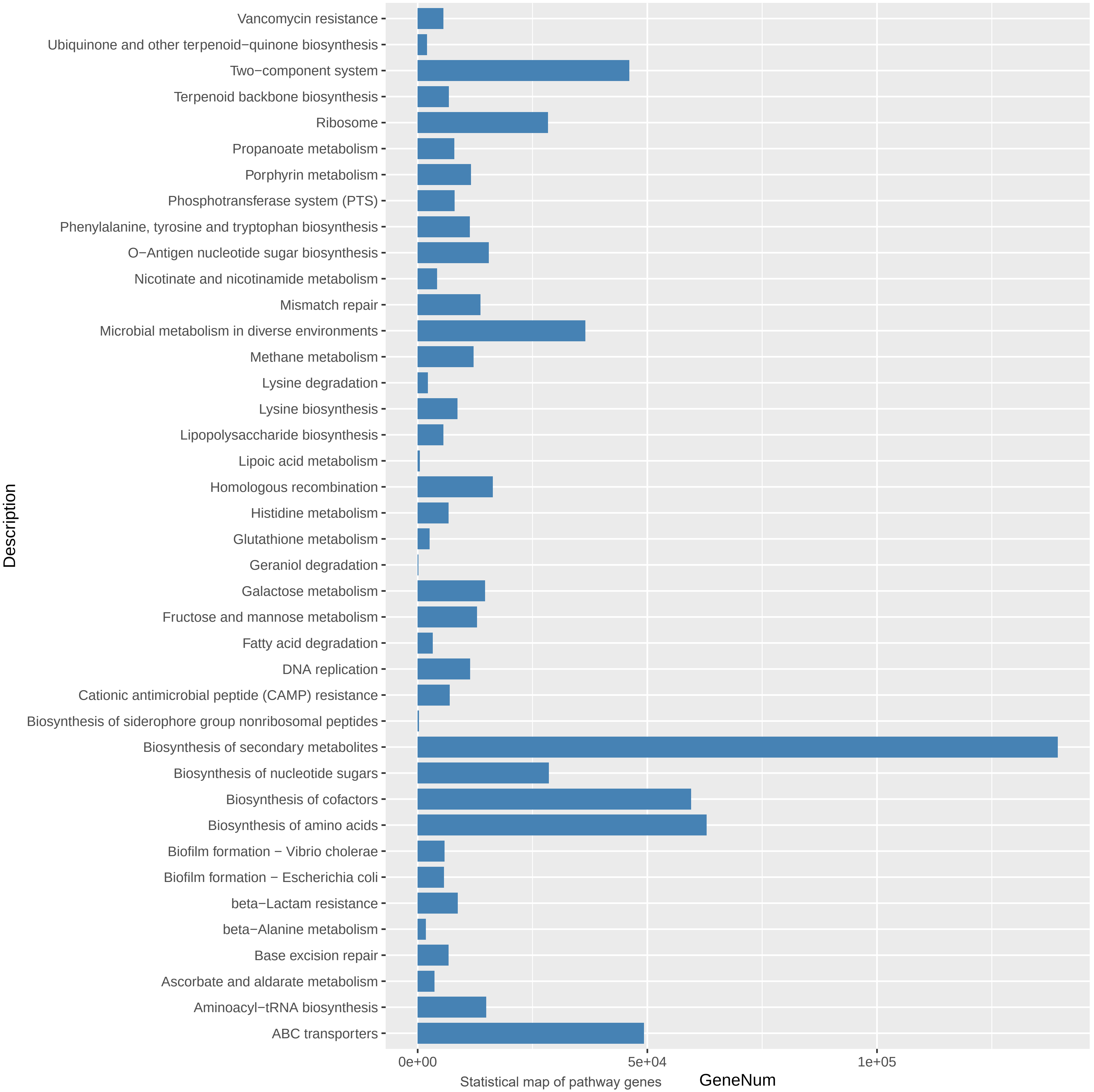

Statistical map of pathway genes

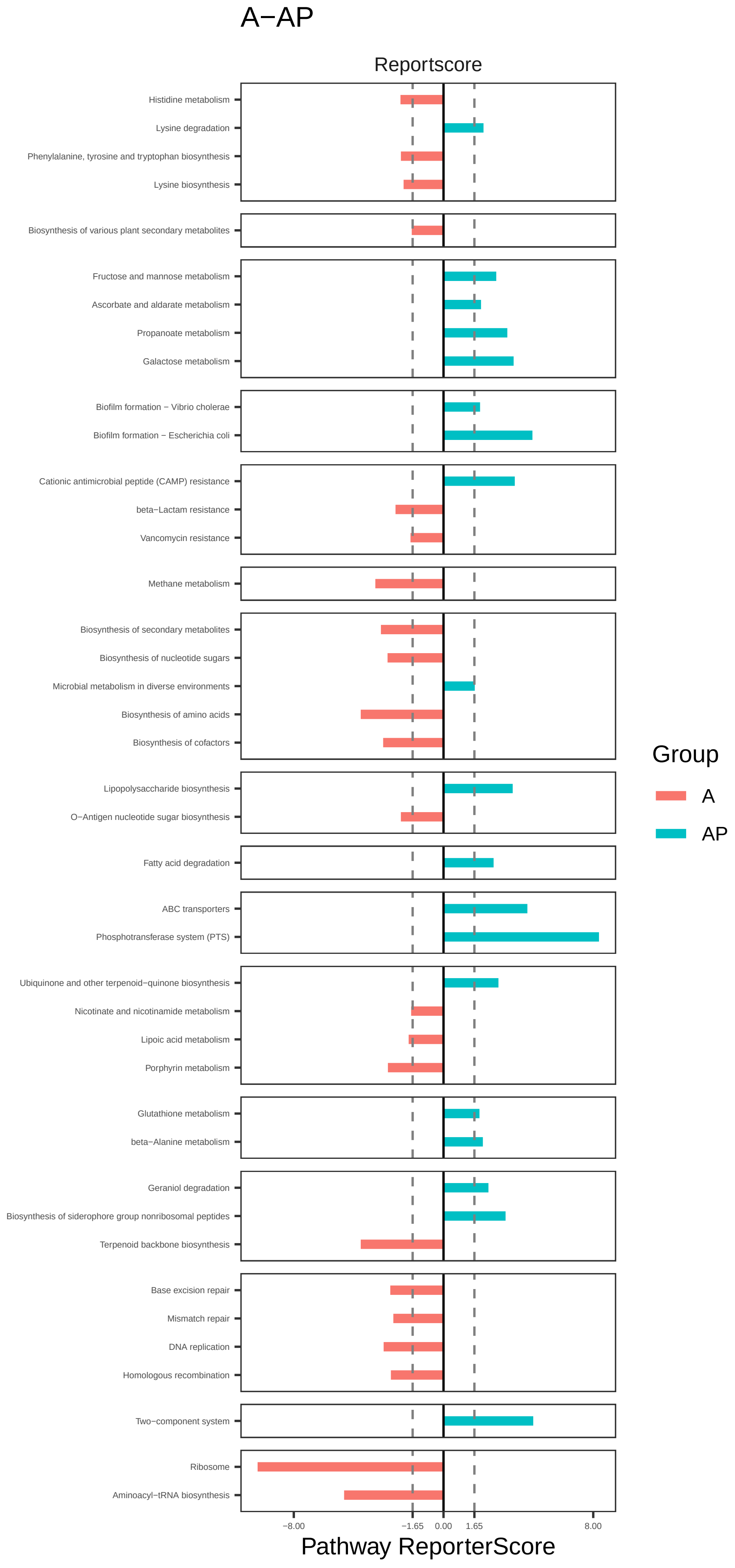
A−AP
Reportscore
Histidine metabolism
Lysine degradation
Phenylalanine, tyrosine and tryptophan biosynthesis
Lysine biosynthesis
Biosynthesis of various plant secondary metabolites
Fructose and mannose metabolism
Ascorbate and aldarate metabolism
Propanoate metabolism
Galactose metabolism
Biofilm formation − Vibrio cholerae
Biofilm formation − Escherichia coli
Cationic antimicrobial peptide (CAMP) resistance
beta−Lactam resistance
Vancomycin resistance
Methane metabolism
Biosynthesis of secondary metabolites
Biosynthesis of nucleotide sugars
Microbial metabolism in diverse environments
Biosynthesis of amino acids
Biosynthesis of cofactors
Lipopolysaccharide biosynthesis
O−Antigen nucleotide sugar biosynthesis
Fatty acid degradation
ABC transporters
Phosphotransferase system (PTS)
Ubiquinone and other terpenoid−quinone biosynthesis
Nicotinate and nicotinamide metabolism
Lipoic acid metabolism
Porphyrin metabolism
Glutathione metabolism
beta−Alanine metabolism
Geraniol degradation
Biosynthesis of siderophore group nonribosomal peptides
Terpenoid backbone biosynthesis
Base excision repair
Mismatch repair
DNA replication
Homologous recombination
Two−component system
Ribosome
Aminoacyl−tRNA biosynthesis
−8.00
−1.65
0.00
1.65
8.00
Pathway ReporterScore
Group
A
AP

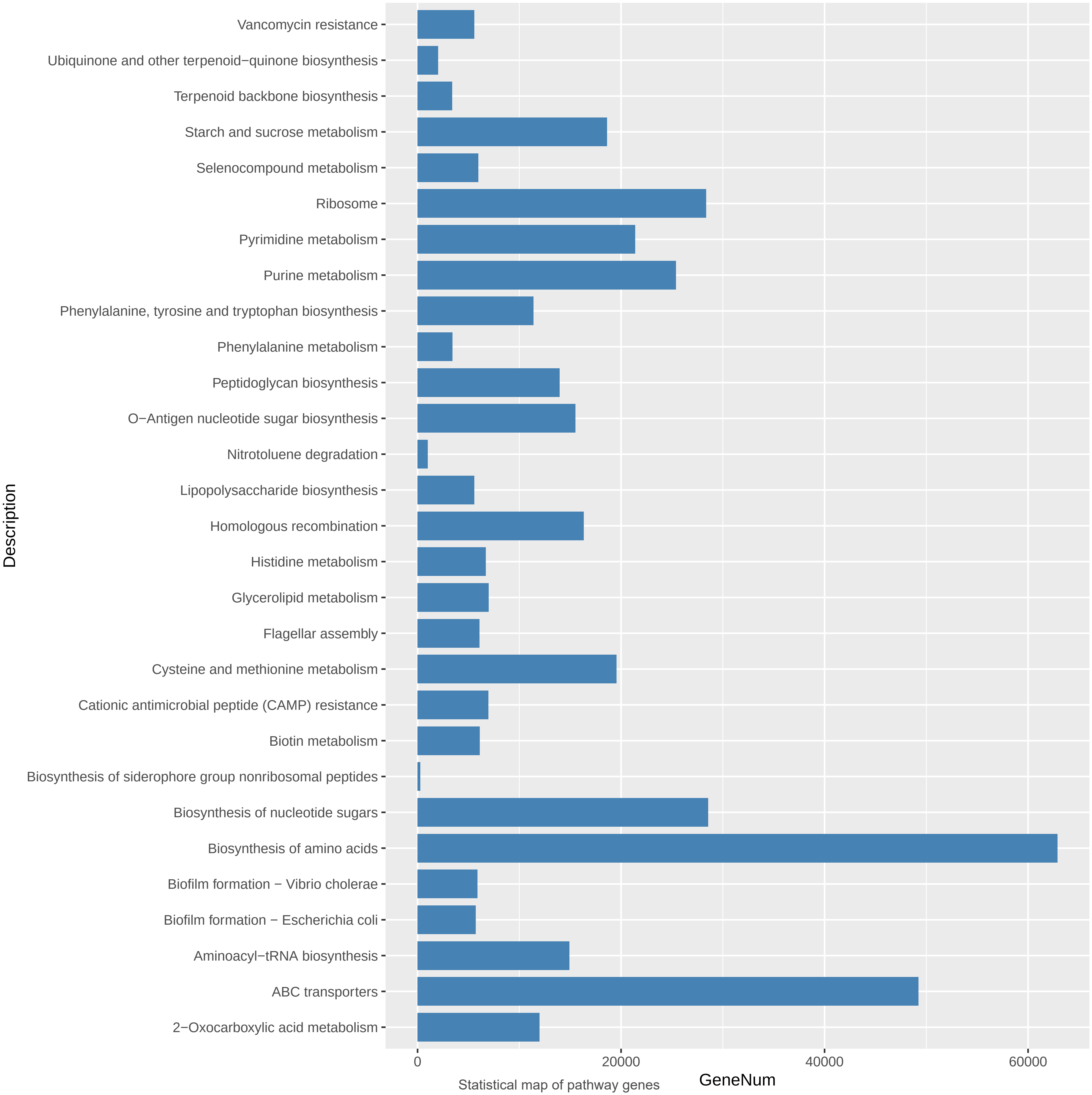

Statistical map of pathway genes

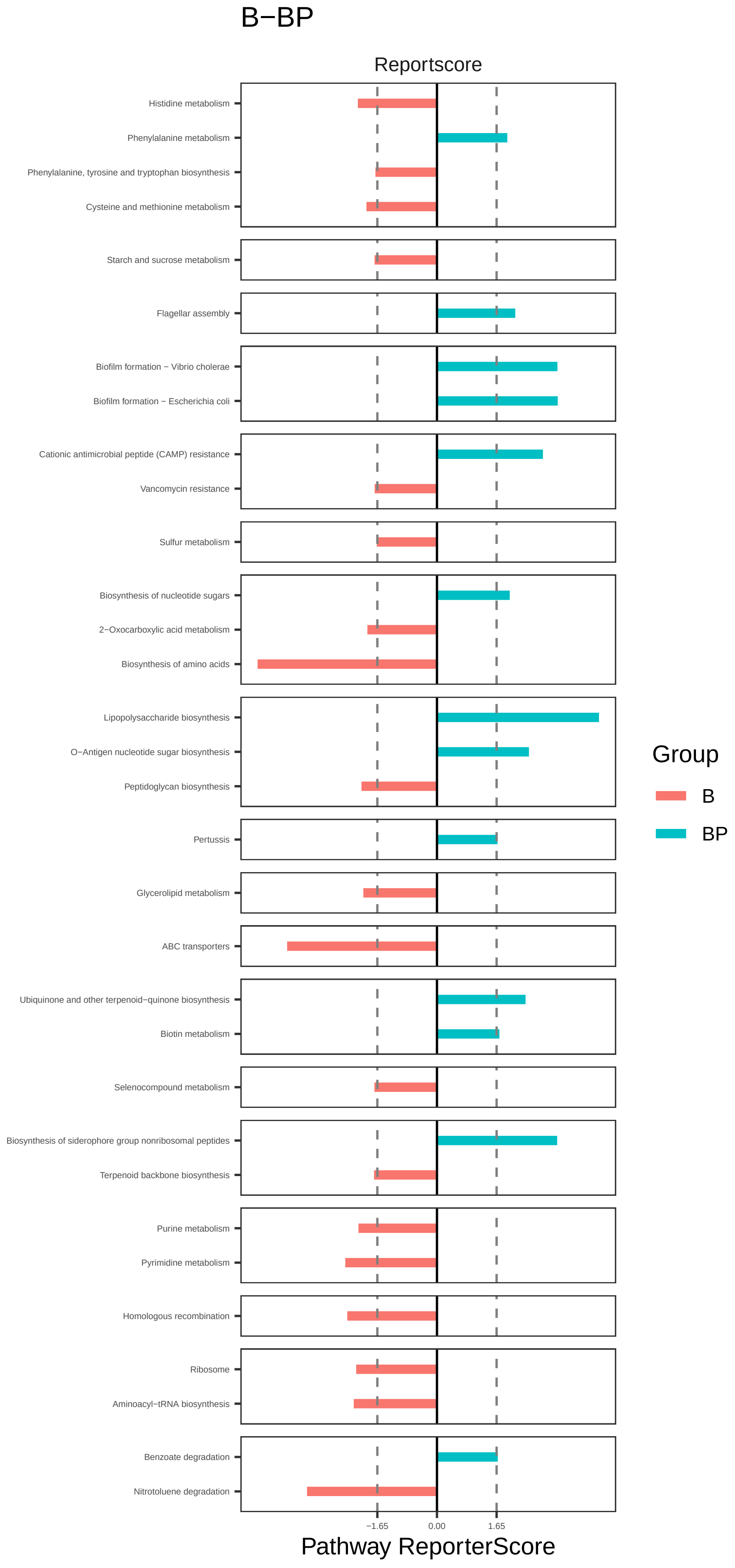
B−BP
Reportscore
Histidine metabolism
Phenylalanine metabolism
Phenylalanine, tyrosine and tryptophan biosynthesis
Cysteine and methionine metabolism
Starch and sucrose metabolism
Flagellar assembly
Biofilm formation − Vibrio cholerae
Biofilm formation − Escherichia coli
Cationic antimicrobial peptide (CAMP) resistance
Vancomycin resistance
Sulfur metabolism
Biosynthesis of nucleotide sugars
2−Oxocarboxylic acid metabolism
Biosynthesis of amino acids
Lipopolysaccharide biosynthesis
O−Antigen nucleotide sugar biosynthesis
Peptidoglycan biosynthesis
Pertussis
Glycerolipid metabolism
ABC transporters
Ubiquinone and other terpenoid−quinone biosynthesis
Biotin metabolism
Selenocompound metabolism
Biosynthesis of siderophore group nonribosomal peptides
Terpenoid backbone biosynthesis
Purine metabolism
Pyrimidine metabolism
Homologous recombination
Ribosome
Aminoacyl−tRNA biosynthesis
Benzoate degradation
Nitrotoluene degradation
−1.65
0.00
1.65
Pathway ReporterScore
Group
B
BP

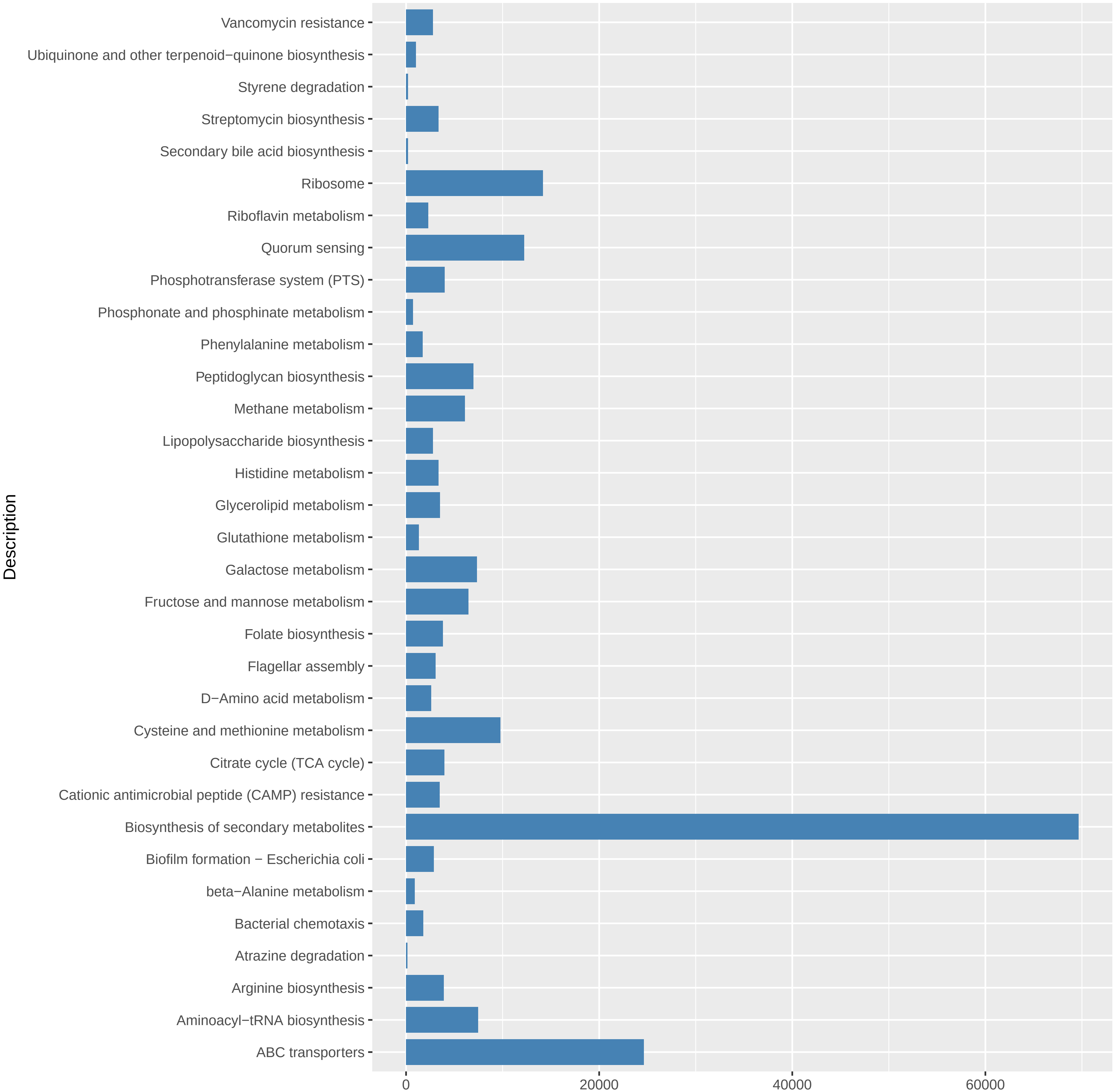

Statistical map of pathway genes

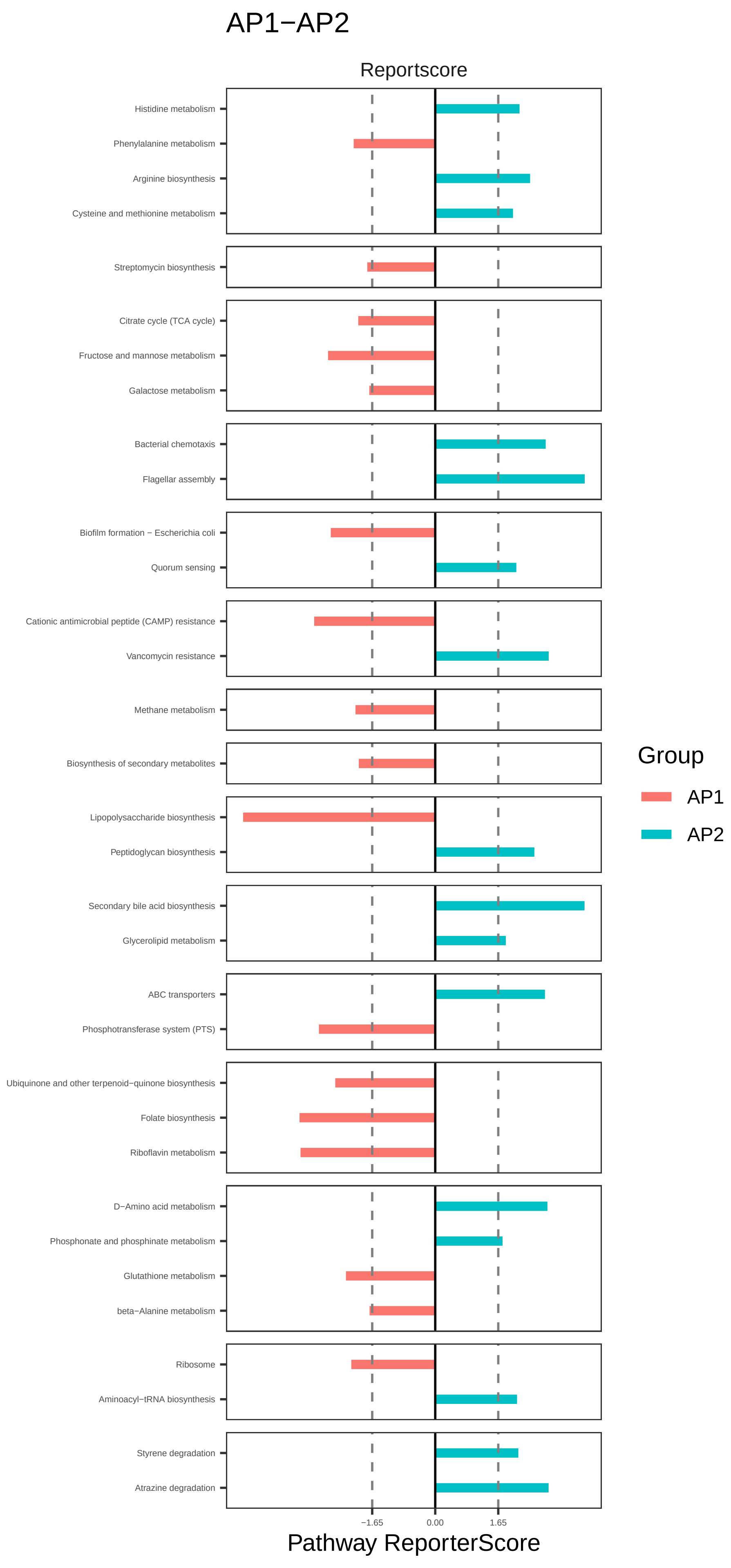
AP1−AP2
Reportscore
Histidine metabolism
Phenylalanine metabolism
Arginine biosynthesis
Cysteine and methionine metabolism
Streptomycin biosynthesis
Citrate cycle (TCA cycle)
Fructose and mannose metabolism
Galactose metabolism
Bacterial chemotaxis
Flagellar assembly
Biofilm formation − Escherichia coli
Quorum sensing
Cationic antimicrobial peptide (CAMP) resistance
Vancomycin resistance
Methane metabolism
Biosynthesis of secondary metabolites
Lipopolysaccharide biosynthesis
Peptidoglycan biosynthesis
Secondary bile acid biosynthesis
Glycerolipid metabolism
ABC transporters
Phosphotransferase system (PTS)
Ubiquinone and other terpenoid−quinone biosynthesis
Folate biosynthesis
Riboflavin metabolism
D−Amino acid metabolism
Phosphonate and phosphinate metabolism
Glutathione metabolism
beta−Alanine metabolism
Ribosome
Aminoacyl−tRNA biosynthesis
Styrene degradation
Atrazine degradation
−1.65
0.00
1.65
Pathway ReporterScore
Group
AP1
AP2

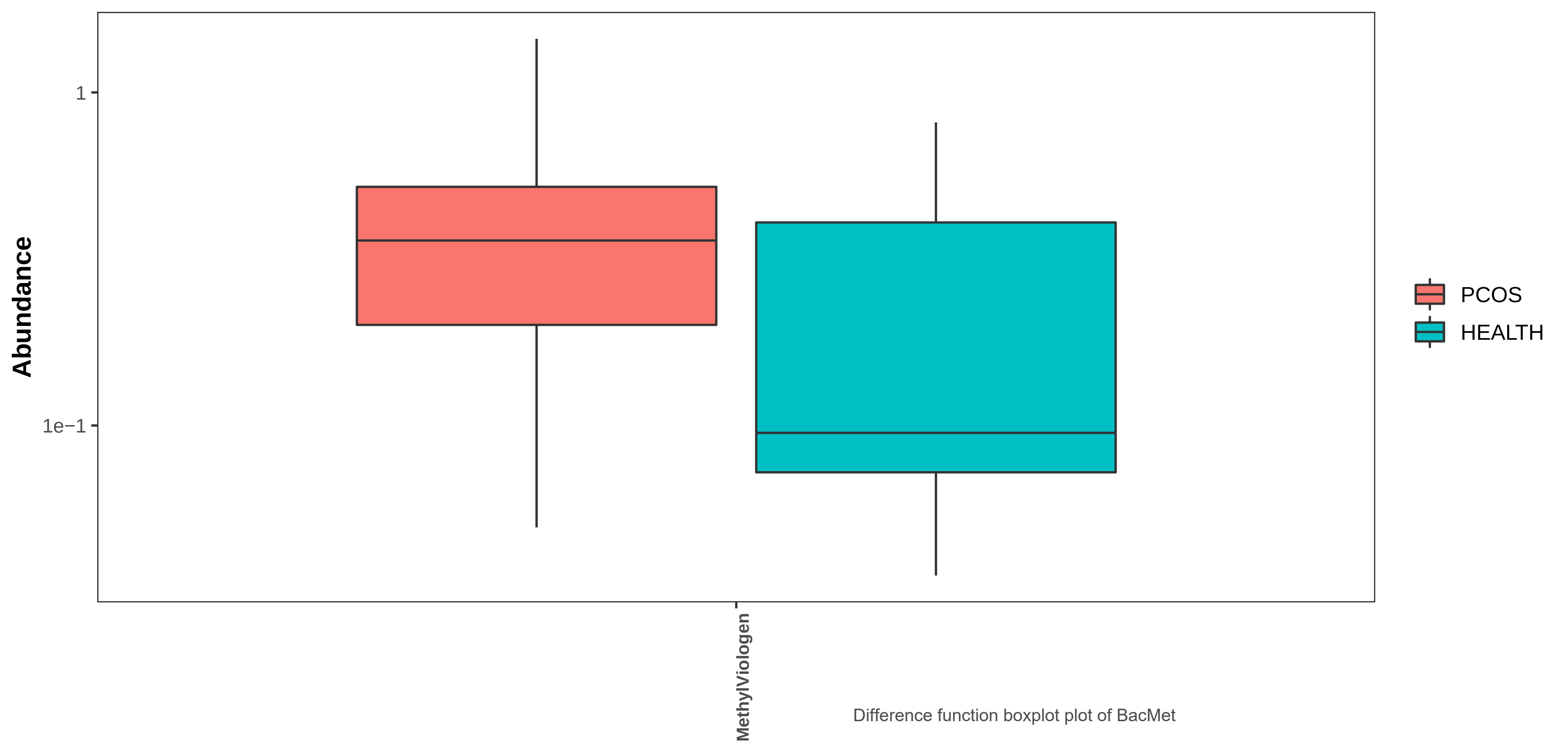

1
1e−1
Abundance
MethylViologen
PCOS
HEALTH
Difference function boxplot plot of BacMet

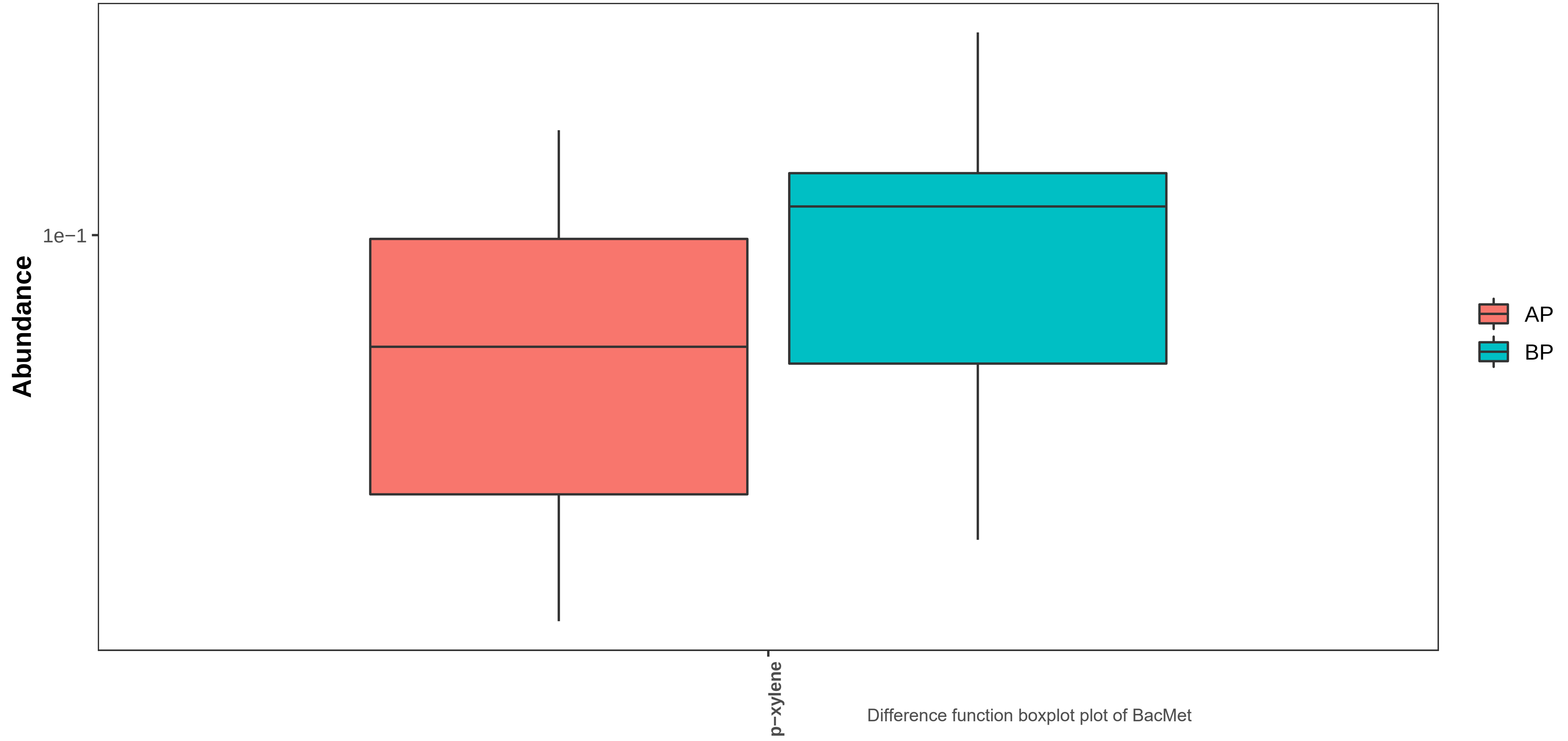

Difference function boxplot plot of BacMet

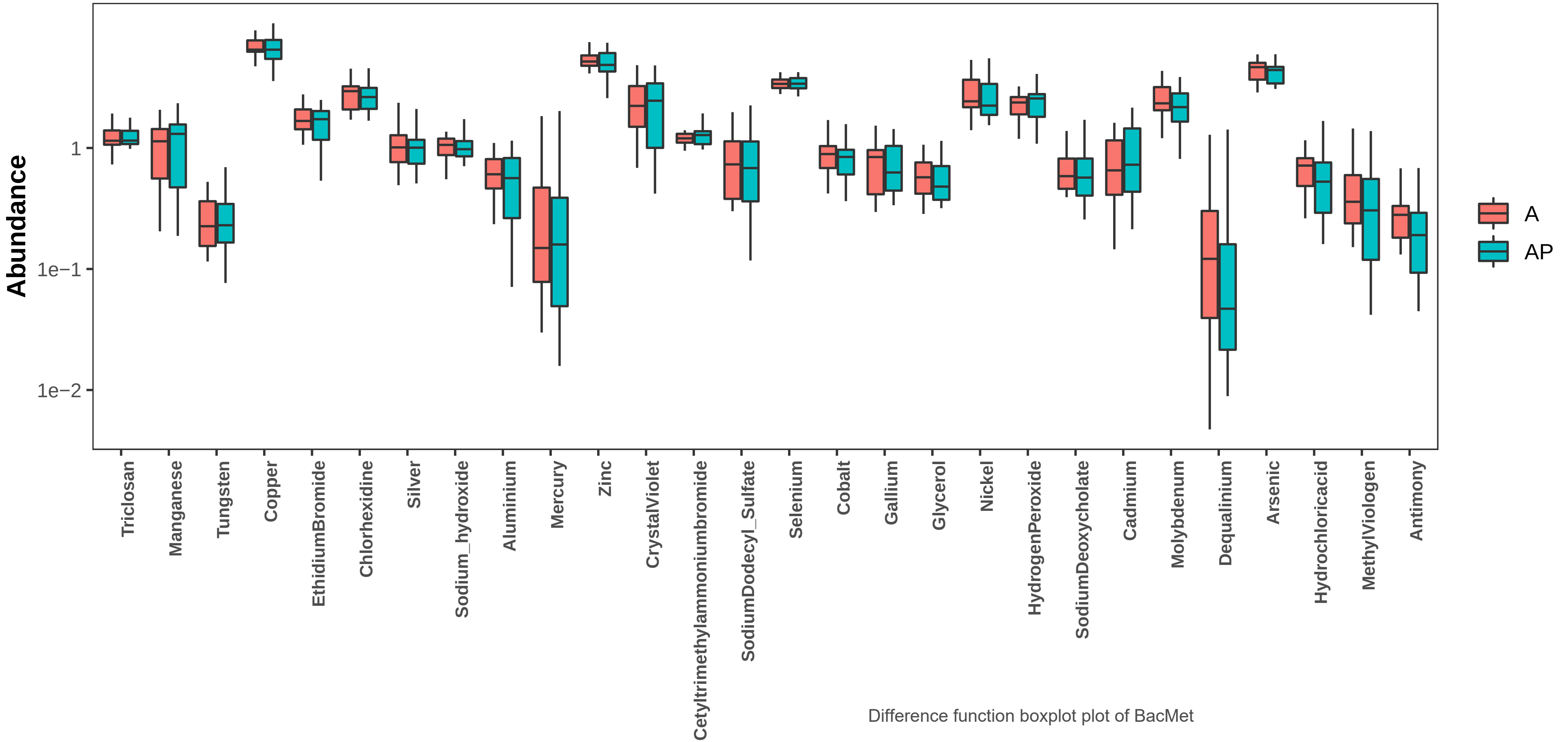

Difference function boxplot plot of BacMet

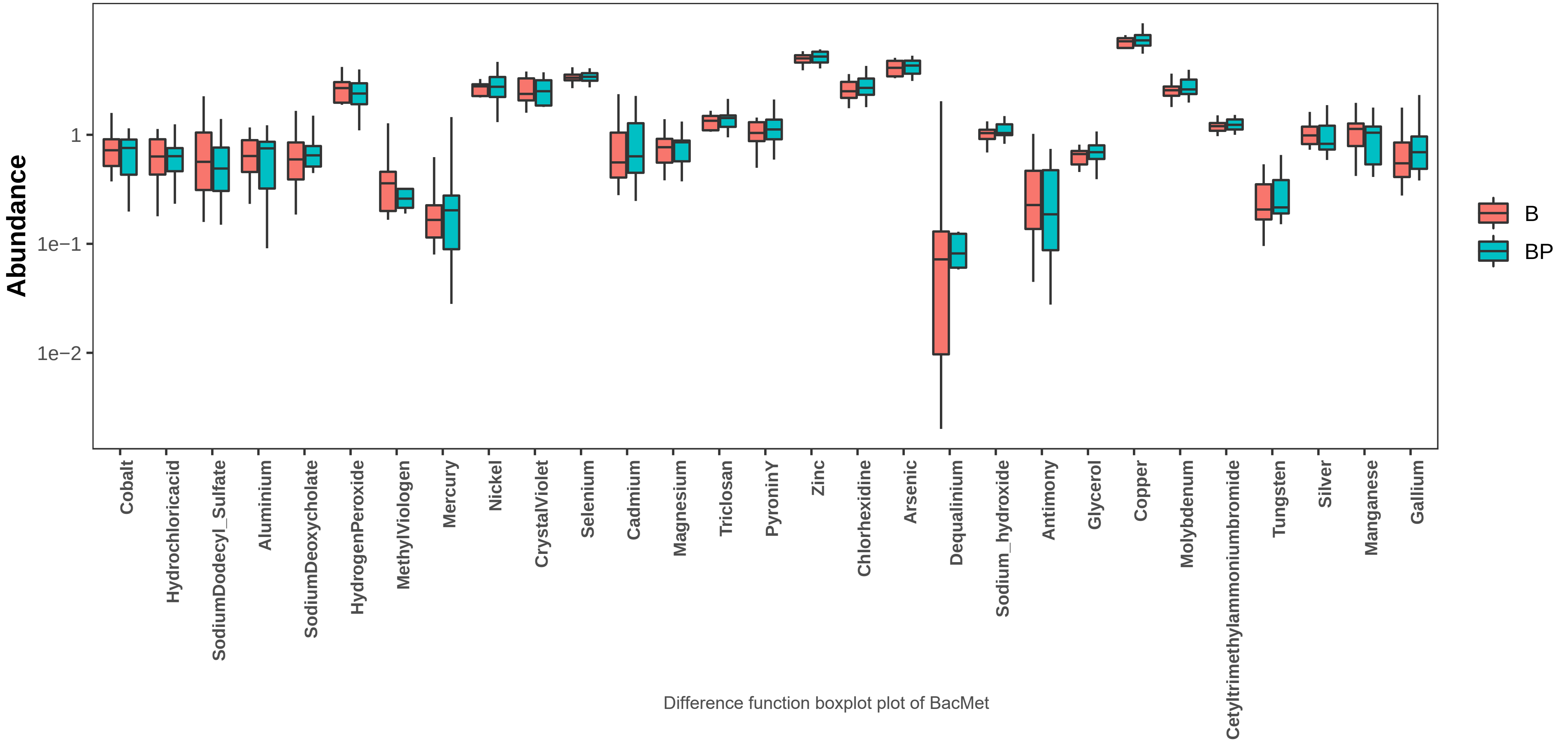

Difference function boxplot plot of BacMet

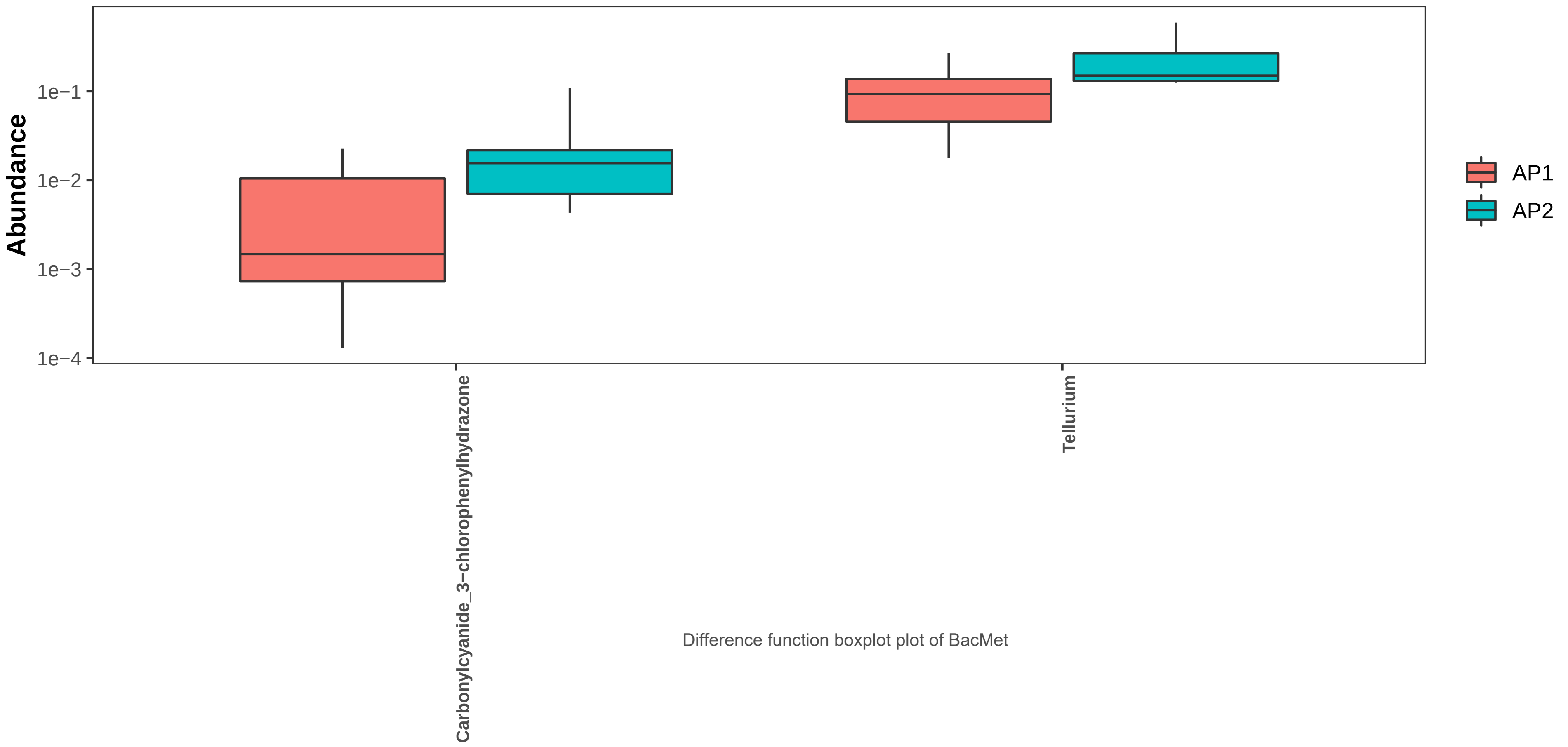

Abundance
1e−1
1e−2
1e−3
1e−4
Carbonylcyanide_3−chlorophenylhydrazone
Tellurium
AP1
AP2
Difference function boxplot plot of BacMet

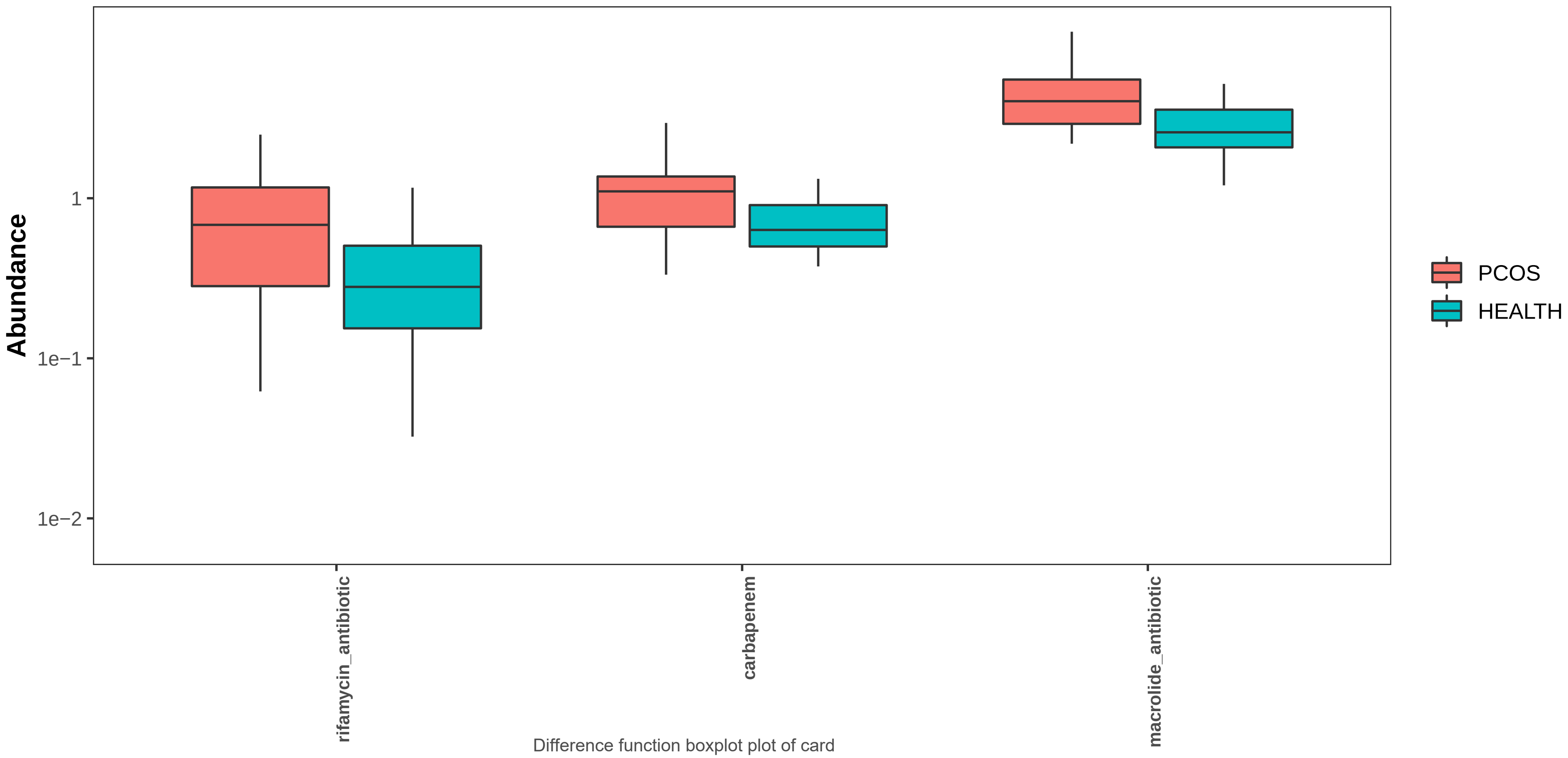
Abundance
1
1e−1
1e−2
rifamycin_antibiotic
carbapenem
macrolide_antibiotic
PCOS
HEALTH
Difference function boxplot plot of card

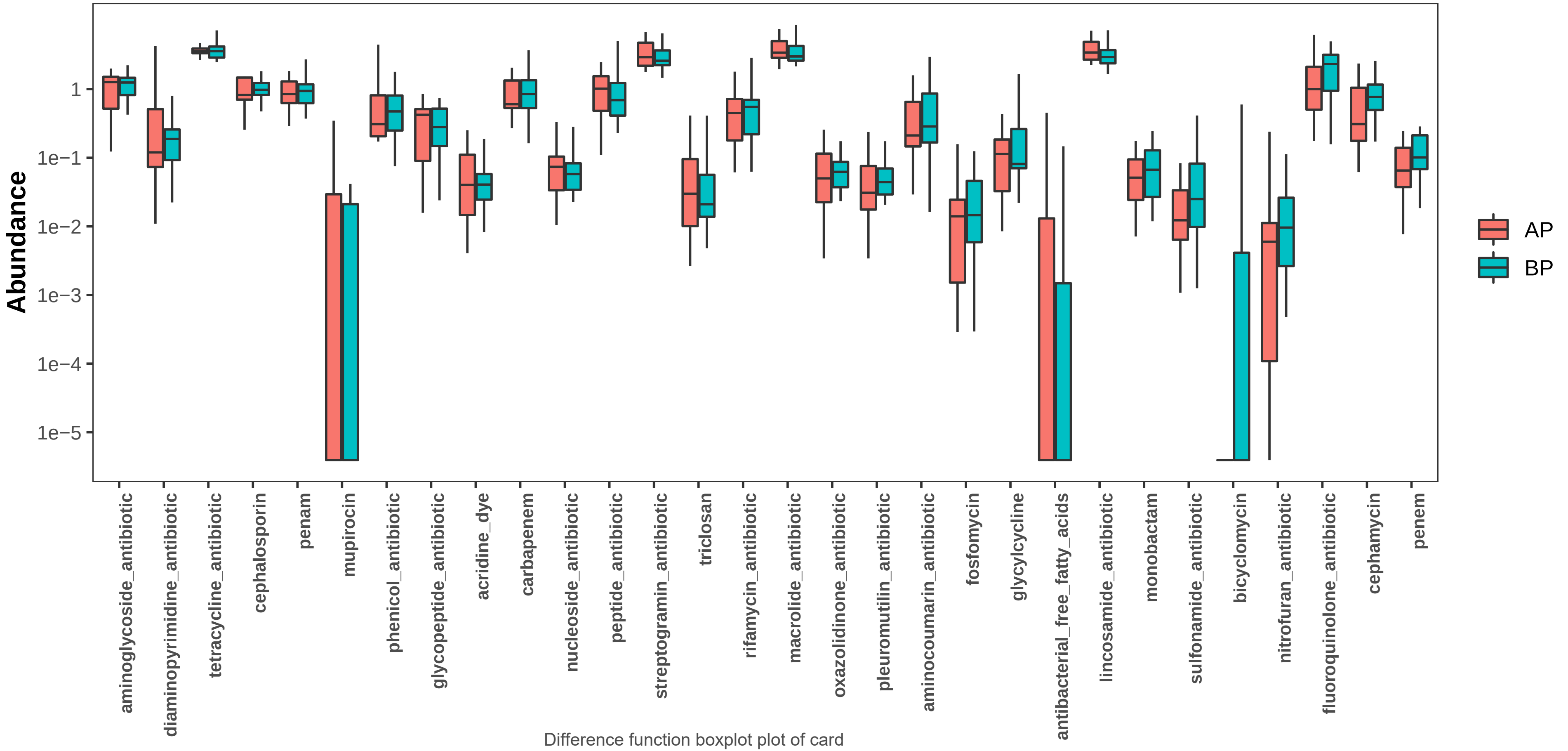

Abundance
1
1e−1
1e−2
1e−3
1e−4
1e−5
AP
BP
aminoglycoside_antibiotic
diaminopyrimidine_antibiotic
tetracycline_antibiotic
cephalosporin
penam
mupirocin
phenicol_antibiotic
glycopeptide_antibiotic
acridine_dye
carbapenem
nucleoside_antibiotic
peptide_antibiotic
streptogramin_antibiotic
triclosan
rifamycin_antibiotic
macrolide_antibiotic
oxazolidinone_antibiotic
pleuromutilin_antibiotic
aminocoumarin_antibiotic
fosfomycin
glycylcycline
antibacterial_free_fatty_acids
lincosamide_antibiotic
monobactam
sulfonamide_antibiotic
bicyclomycin
nitrofuran_antibiotic
fluoroquinolone_antibiotic
cephamycin
penem
Difference function boxplot plot of card

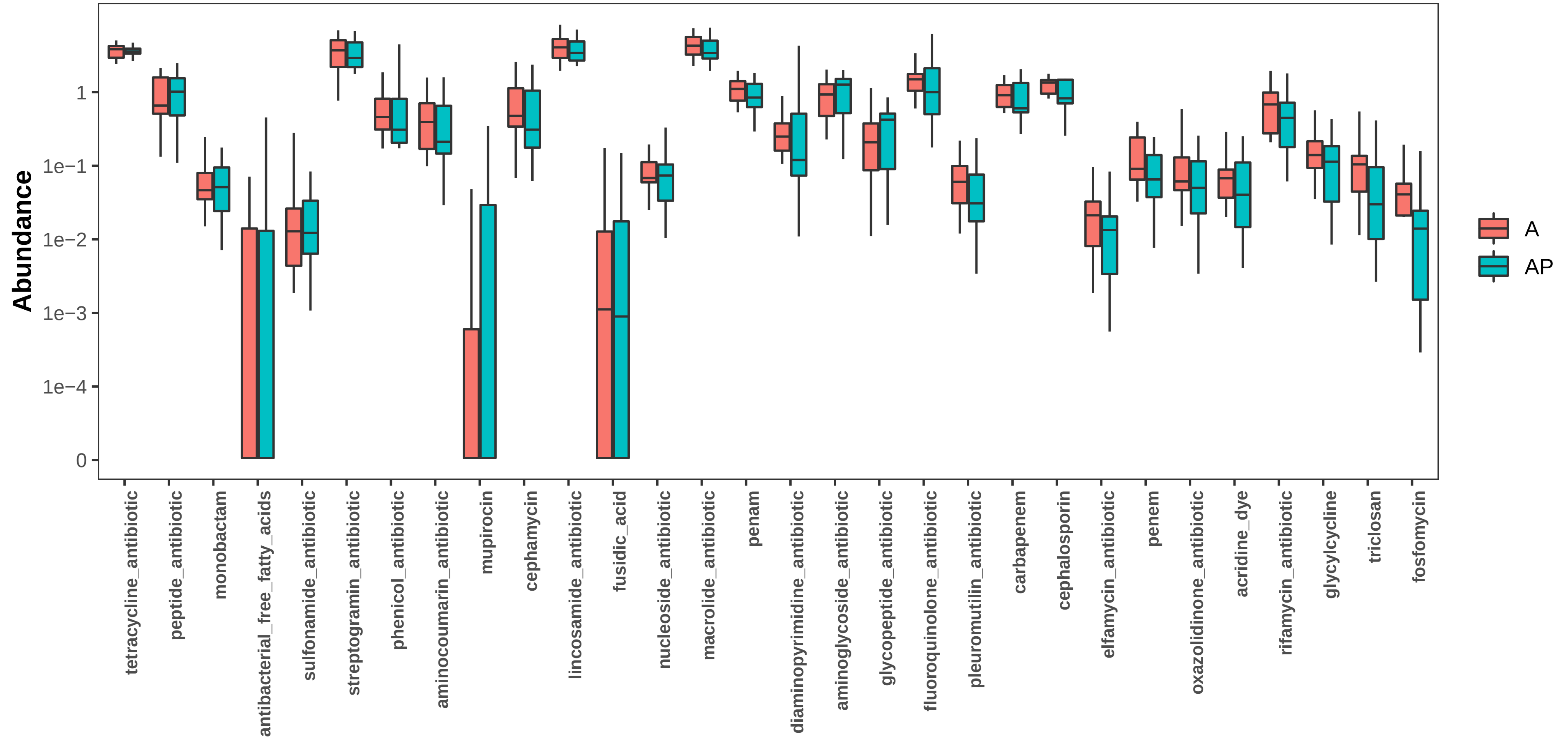

Difference function boxplot plot of card

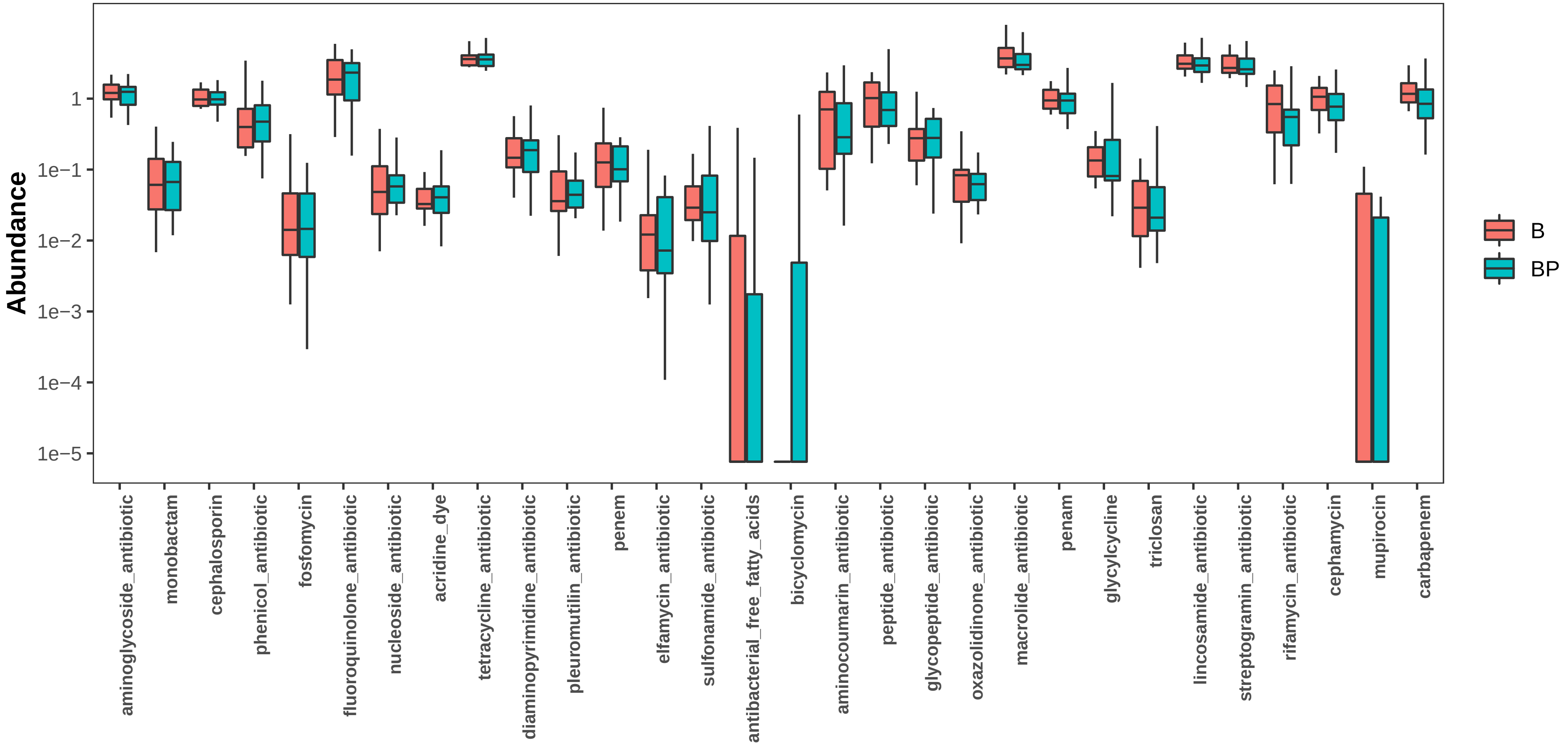

Difference function boxplot plot of card

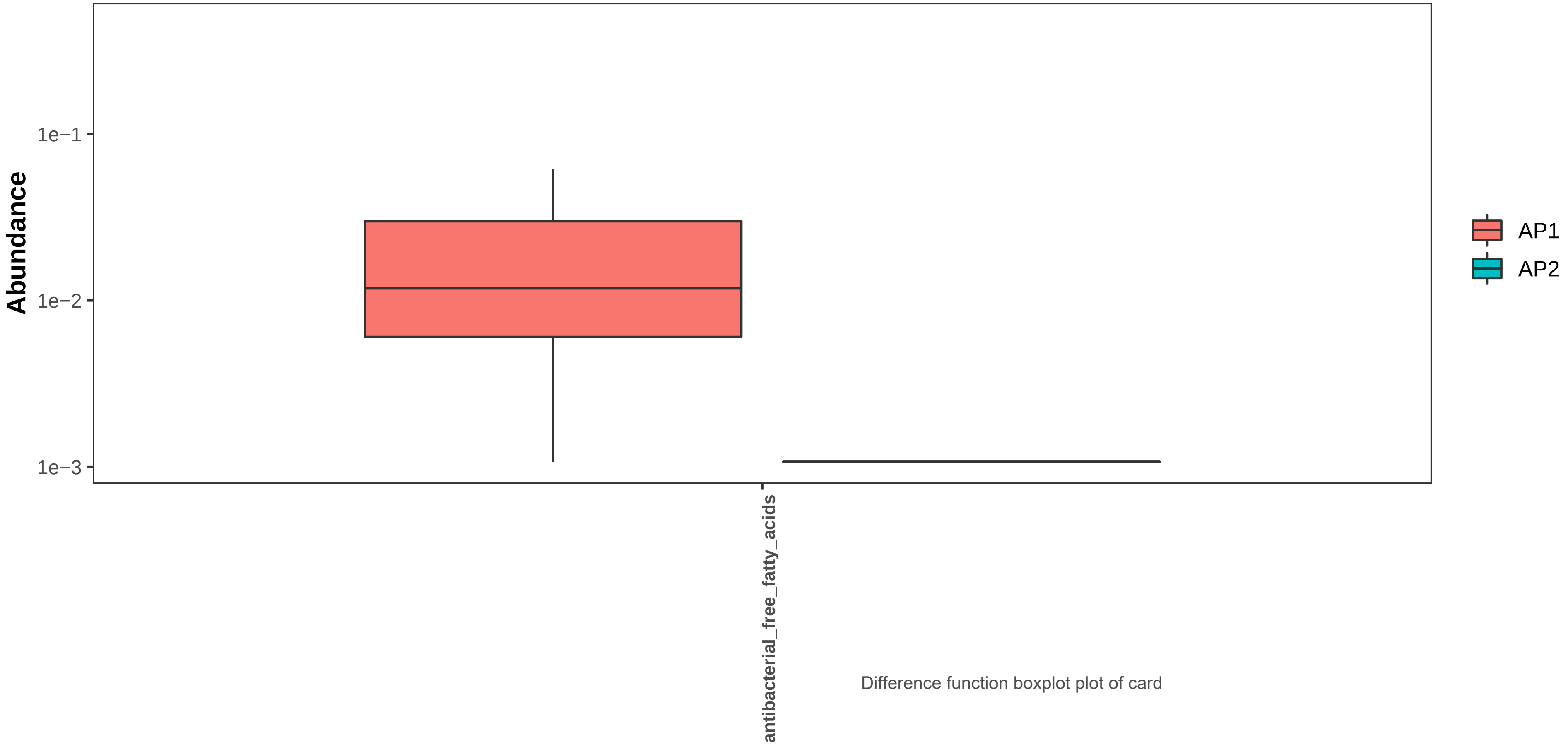

Abundance
1e−1
1e−2
1e−3
antibacterial_free_fatty_acids
AP1
AP2
Difference function boxplot plot of card

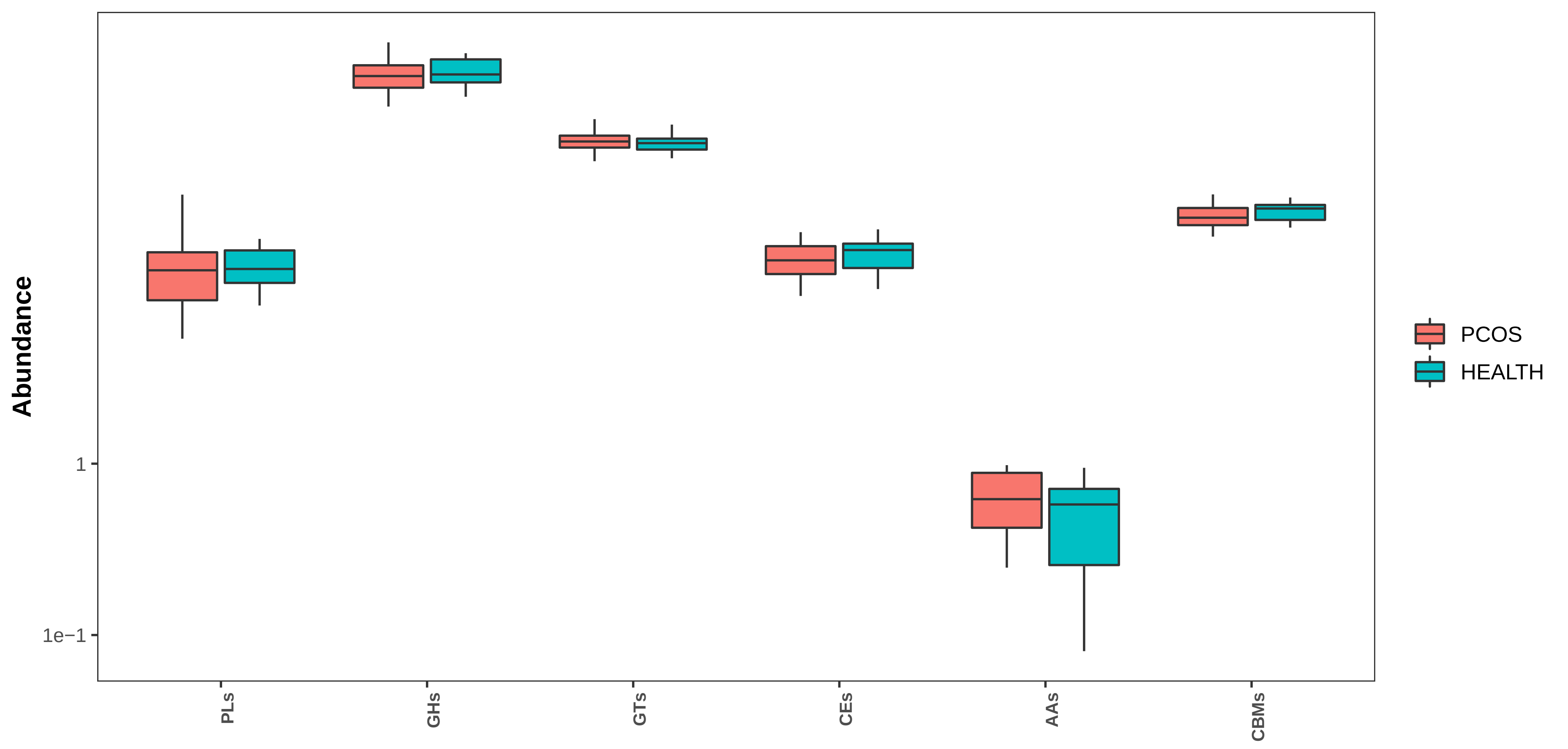

Difference function boxplot plot of Cazy_level1

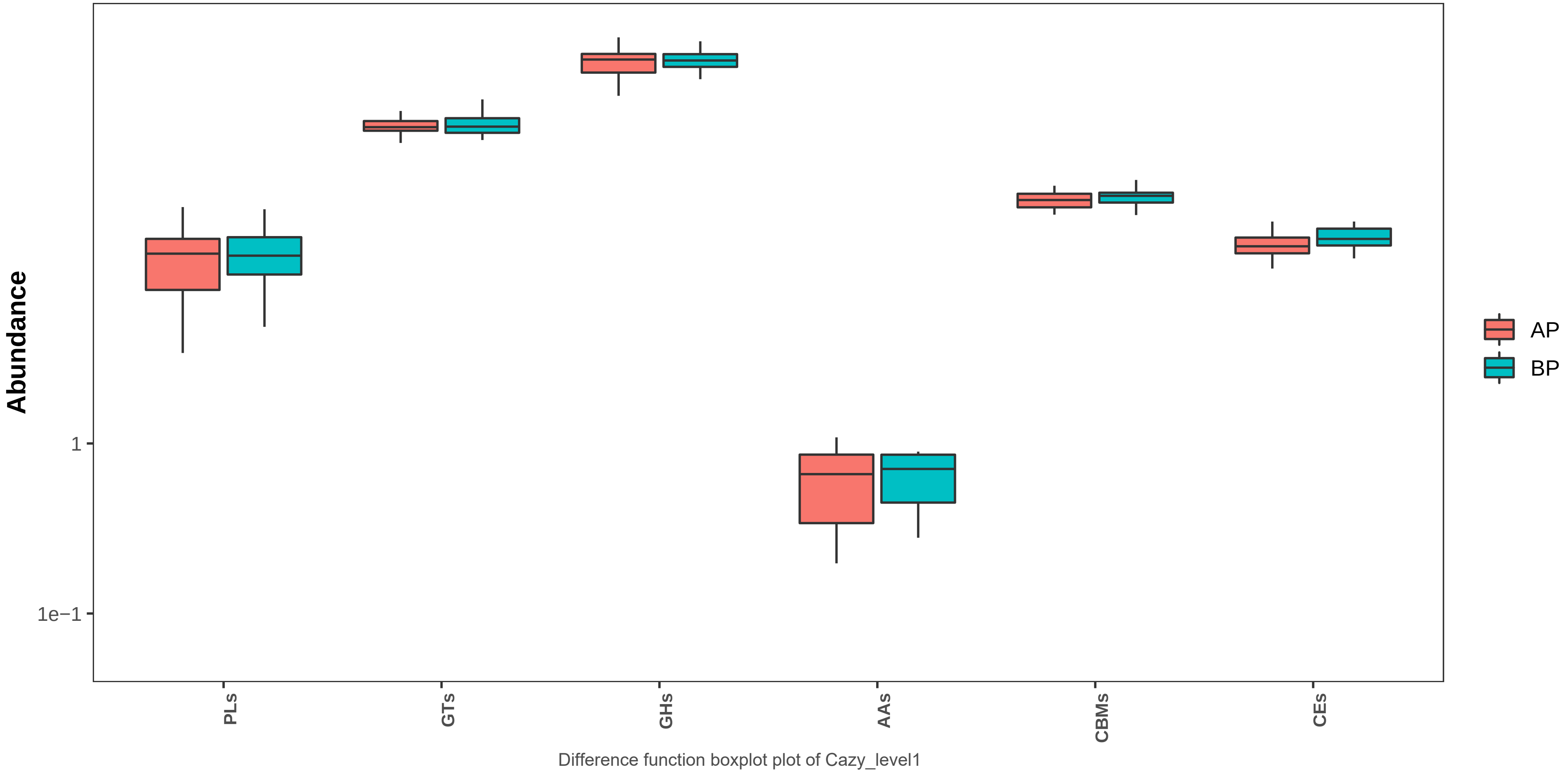

Abundance
1
1e−1
PLs
GTs
GHs
AAs
CBMs
CEs
AP
BP
Difference function boxplot plot of Cazy_level1

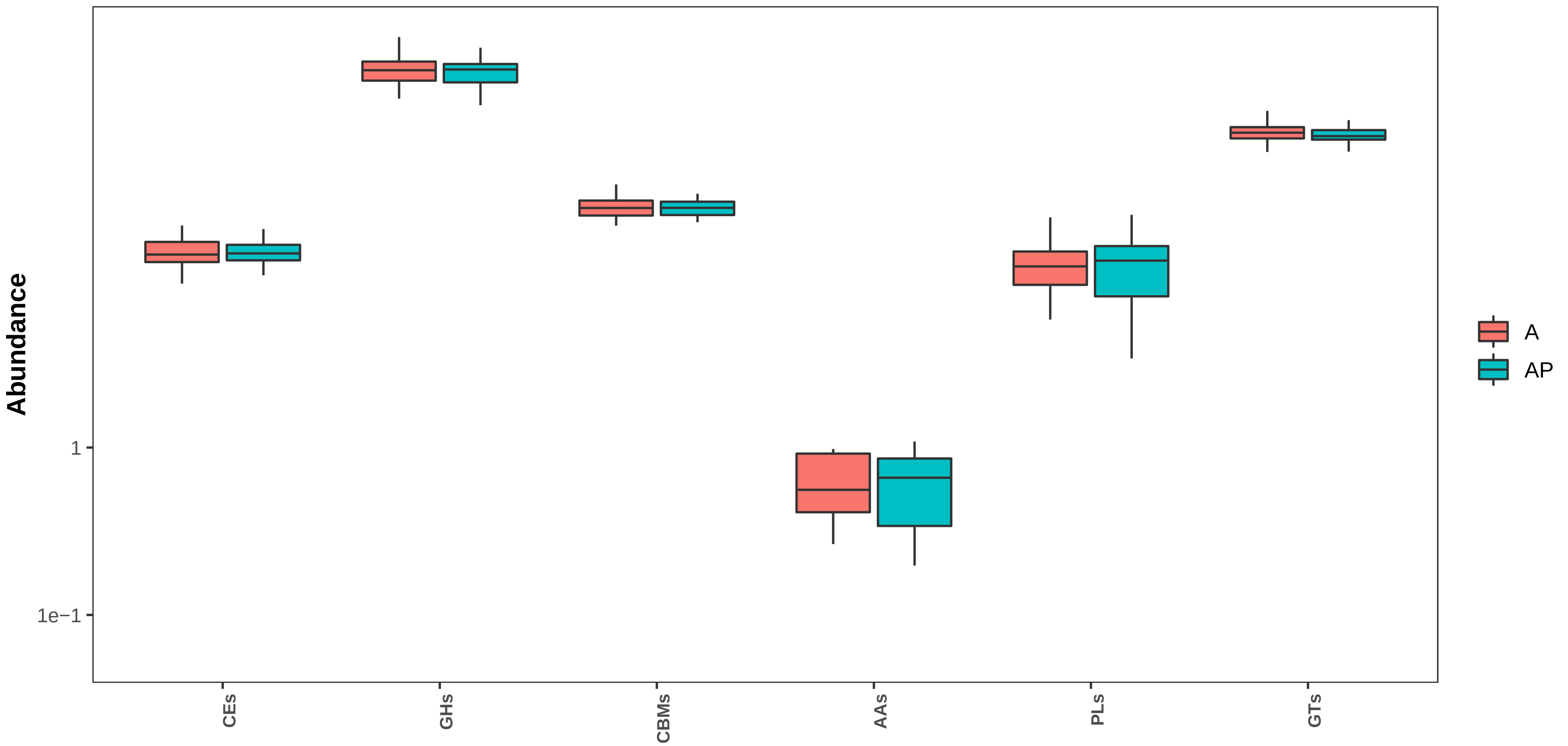

Difference function boxplot plot of Cazy_level1

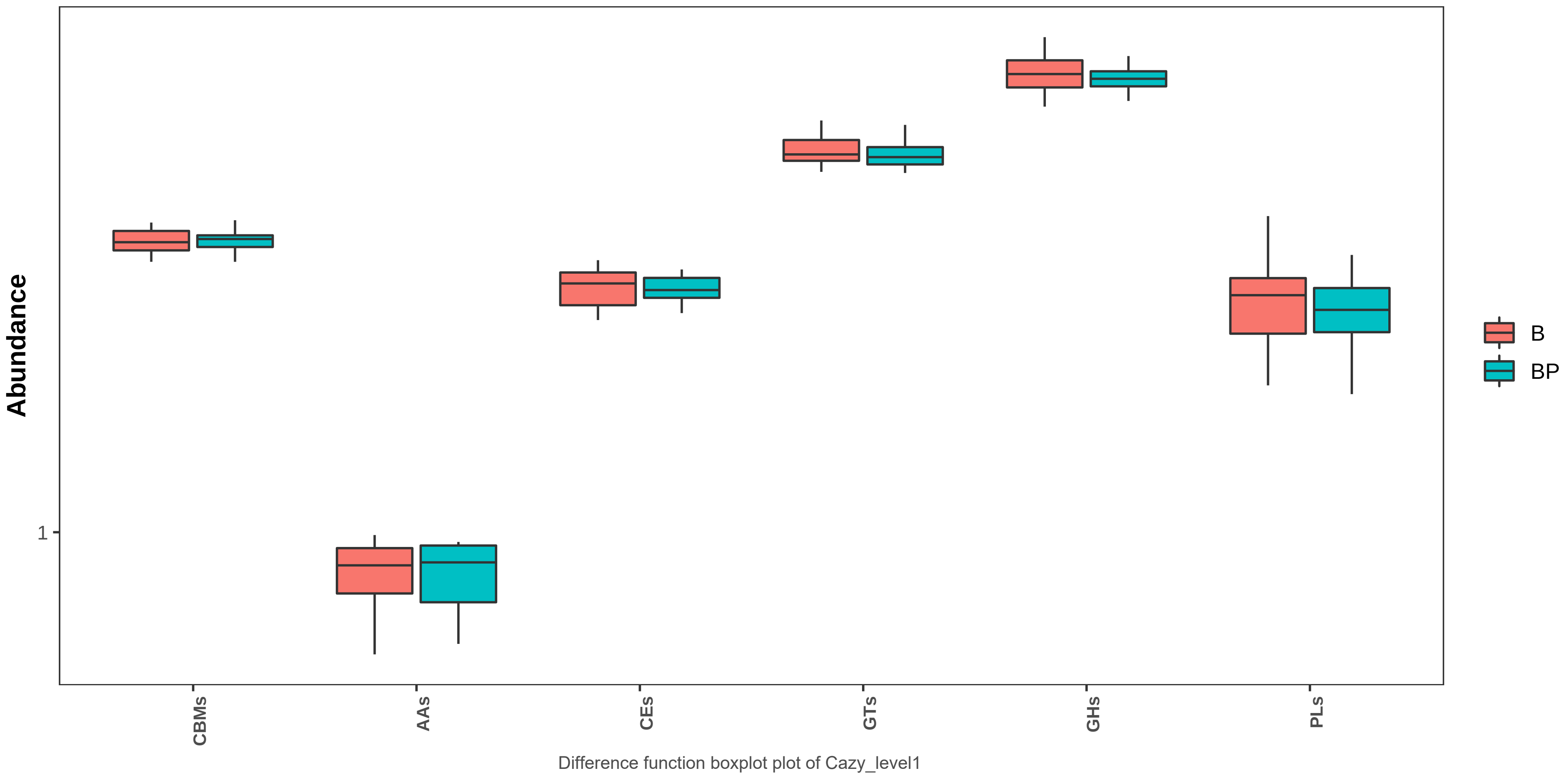
Abundance
1
CBMs
AAs
CEs
GTs
GHs
PLs
B
BP
Difference function boxplot plot of Cazy_level1

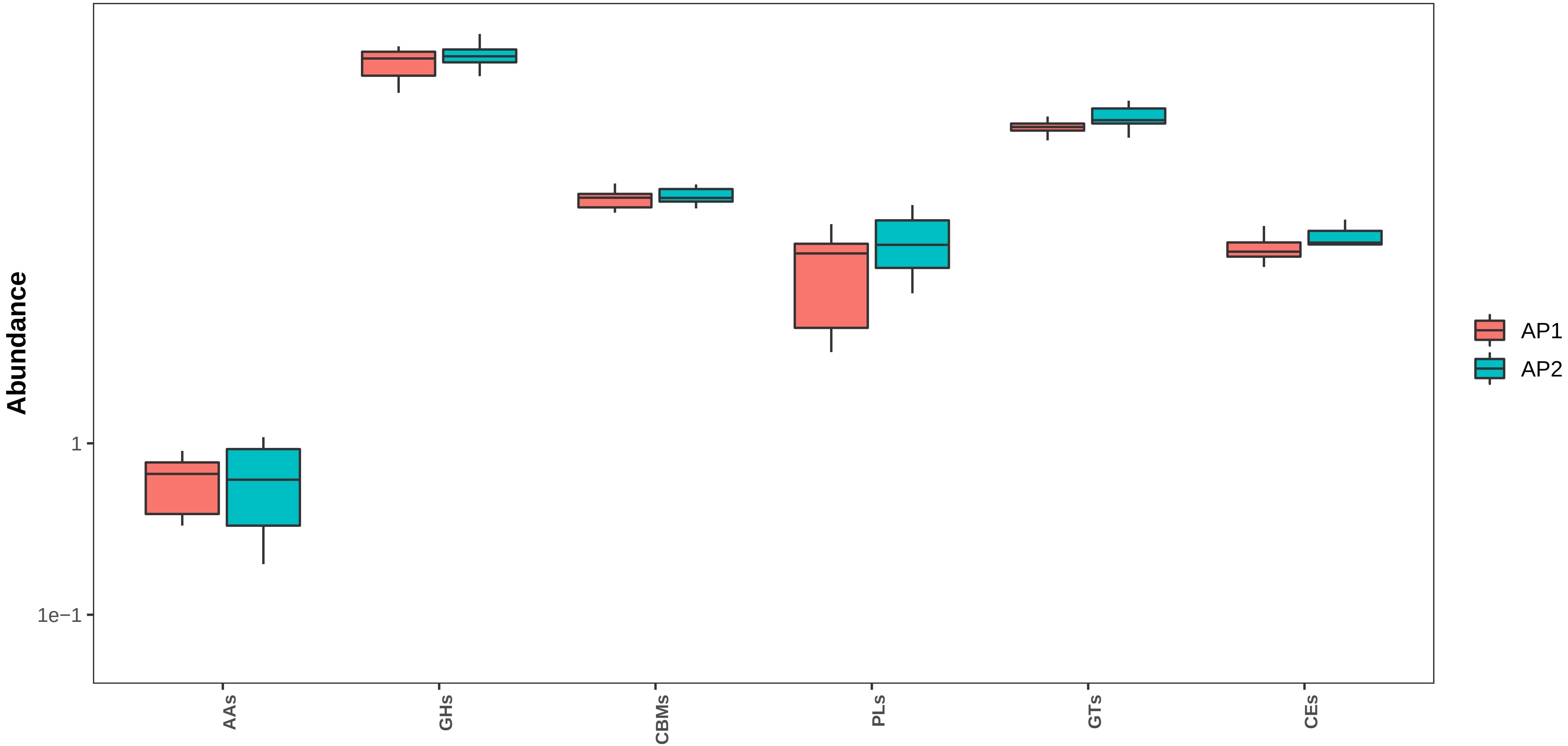

Difference function boxplot plot of Cazy_level1

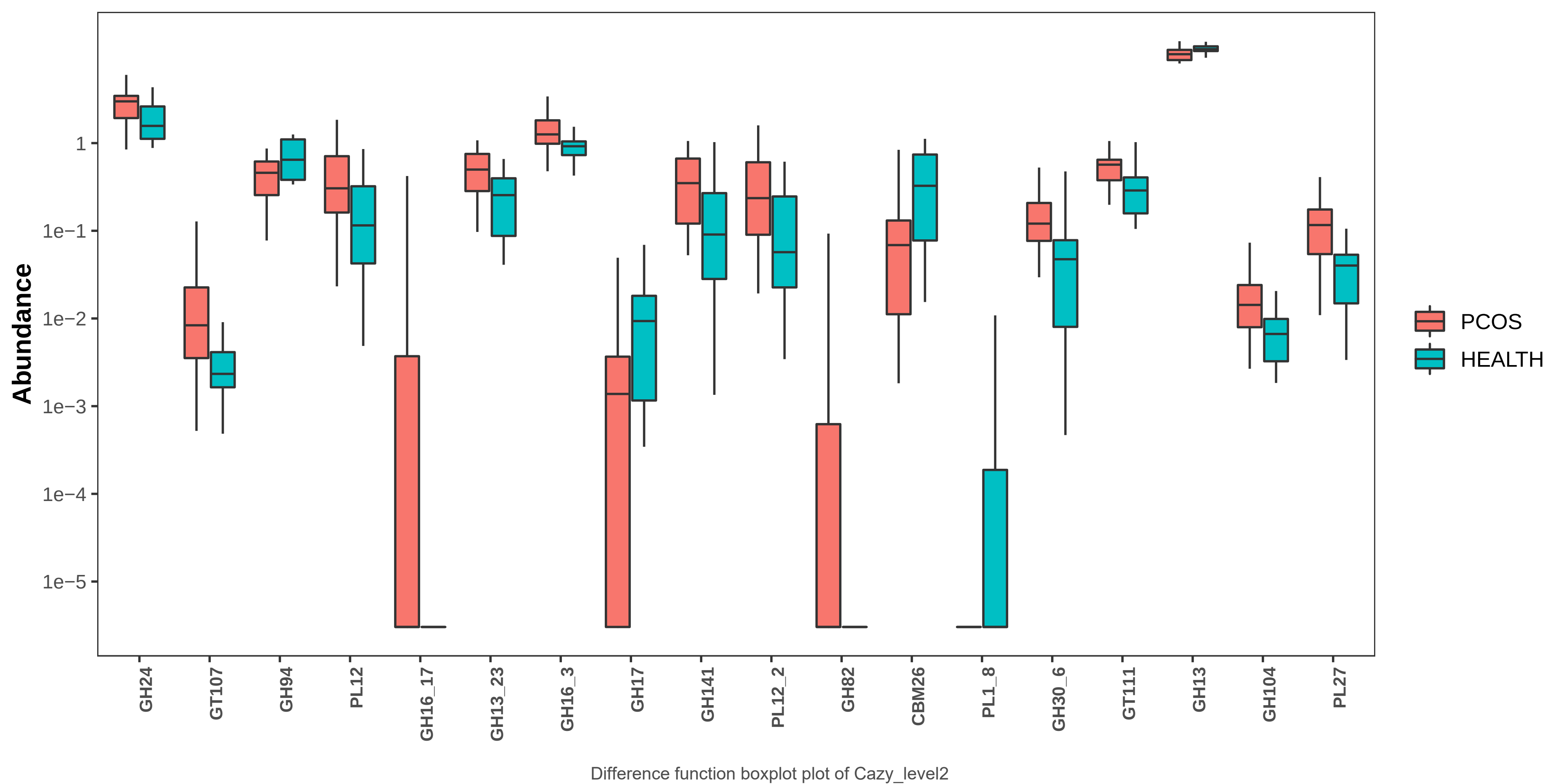

Abundance
1
1e−1
1e−2
1e−3
1e−4
1e−5
GH24
GT107
GH94
PL12
GH16_17
GH13_23
GH16_3
GH17
GH141
PL12_2
GH82
CBM26
PL1_8
GH30_6
GT111
GH13
GH104
PL27
PCOS
HEALTH
Difference function boxplot plot of Cazy_level2

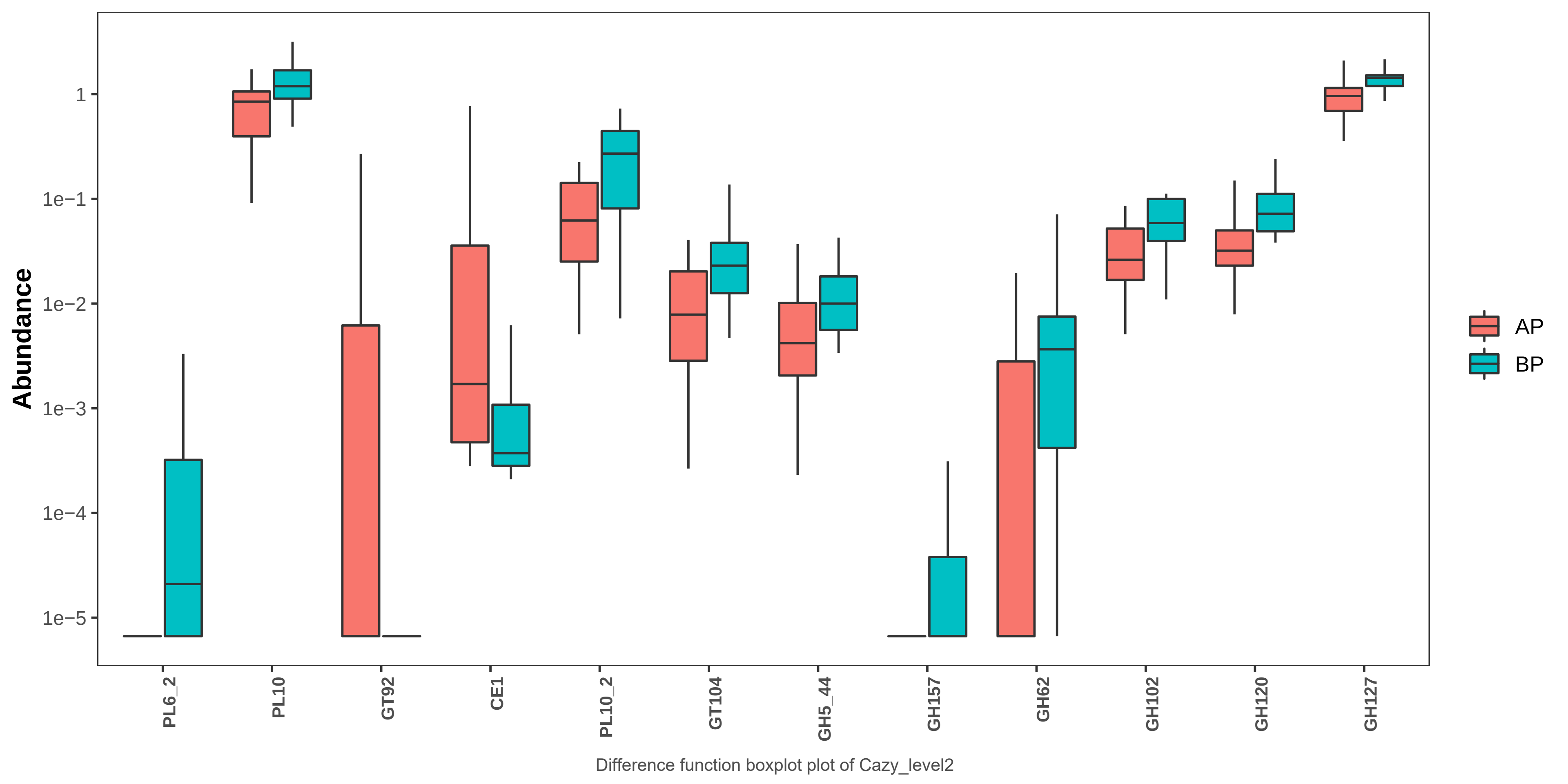

Abundance
1
1e−1
1e−2
1e−3
1e−4
1e−5
PL6_2
PL10
GT92
CE1
PL10_2
GT104
GH5_44
GH157
GH62
GH102
GH120
GH127
AP
BP
Difference function boxplot plot of Cazy_level2

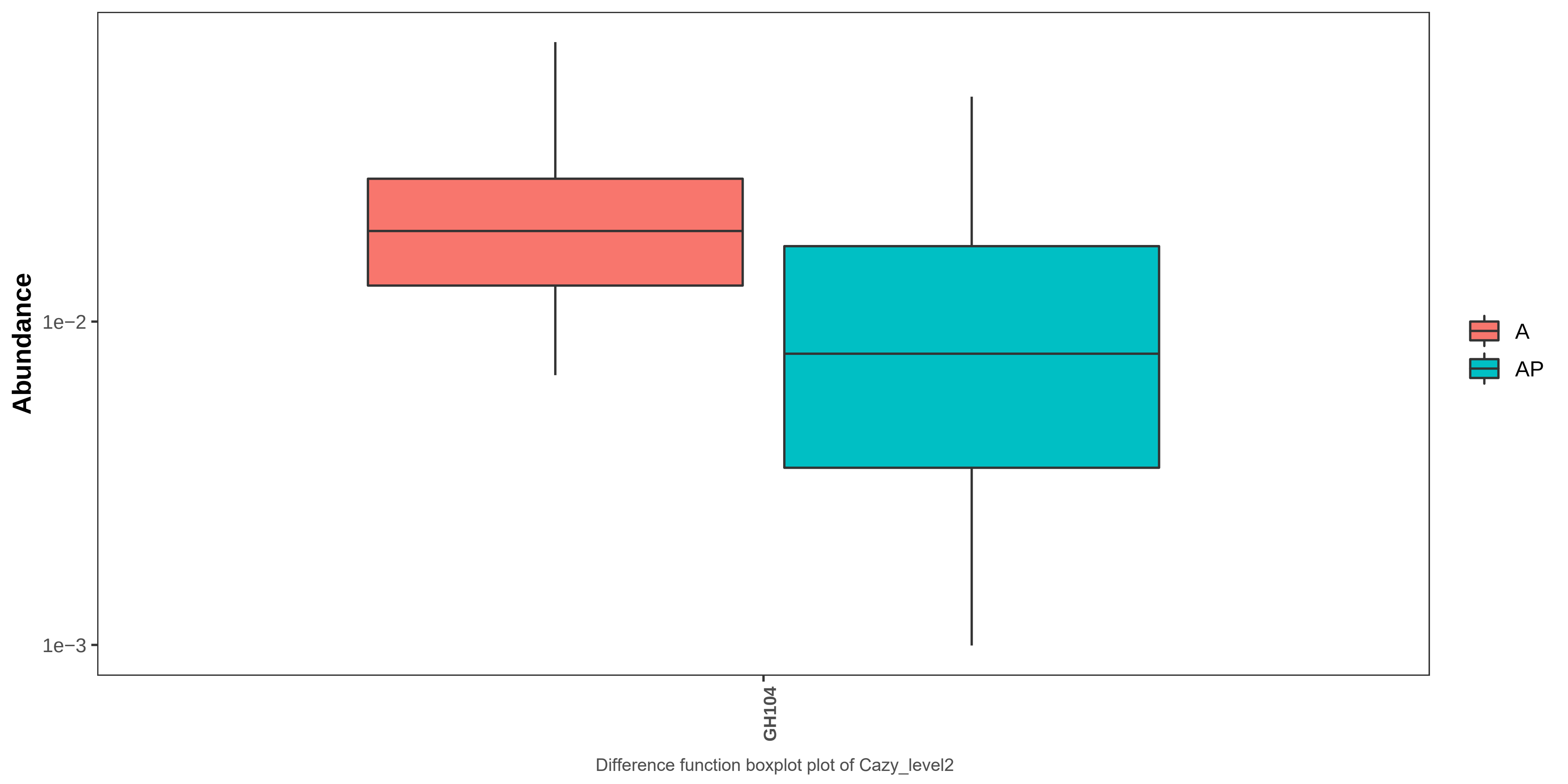
Abundance
1e−2
1e−3
GH104
A
AP
Difference function boxplot plot of Cazy_level2

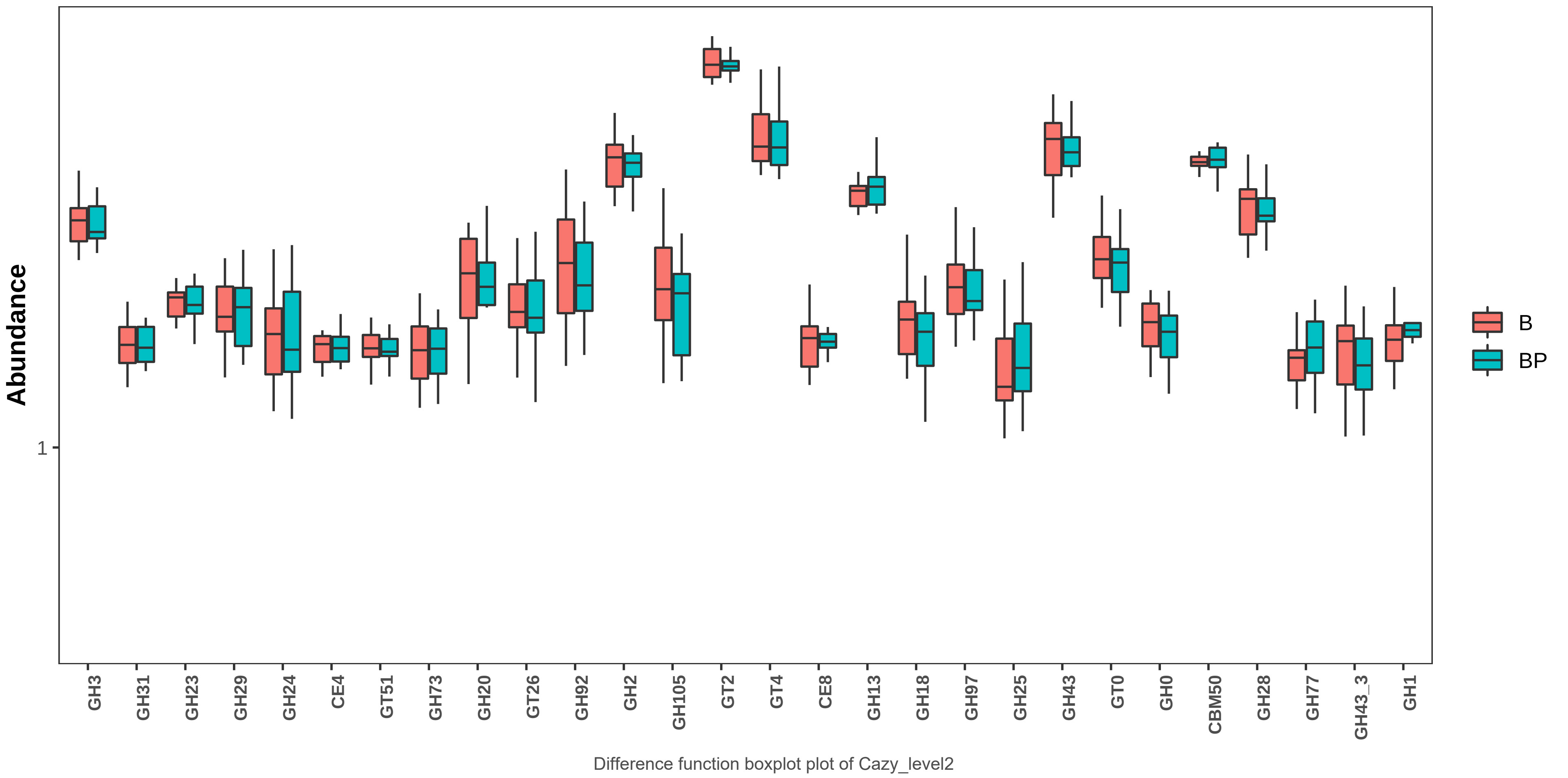
Abundance
1
GH3
GH31
GH23
GH29
GH24
CE4
GT51
GH73
GH20
GT26
GH92
GH2
GH105
GT2
GT4
CE8
GH13
GH18
GH97
GH25
GH43
GT0
GH0
CBM50
GH28
GH77
GH43_3
GH1
B
BP
Difference function boxplot plot of Cazy_level2

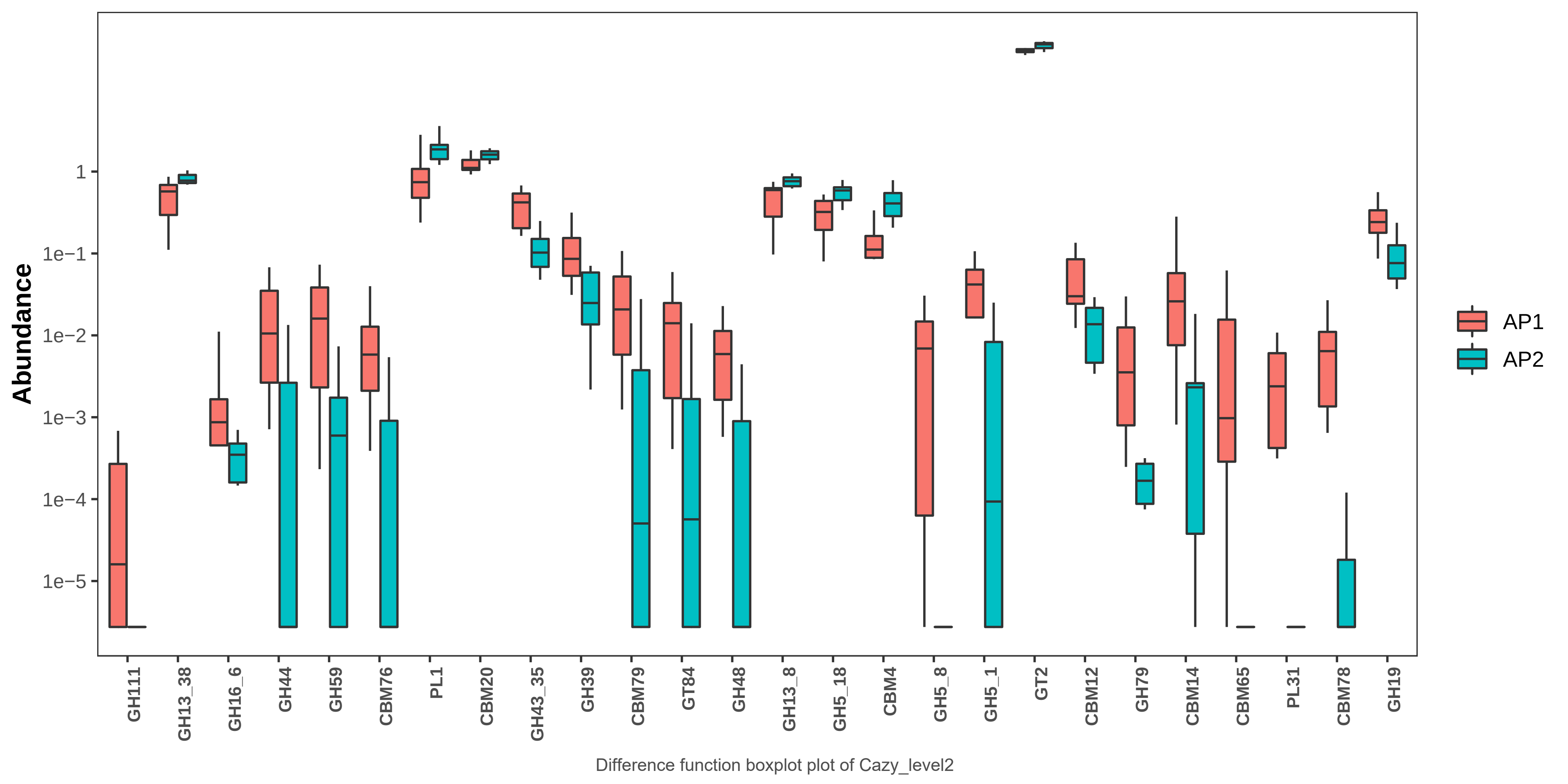

Abundance
1
1e−1
1e−2
1e−3
1e−4
1e−5
GH111
GH13_38
GH16_6
GH44
GH59
CBM76
PL1
CBM20
GH43_35
GH39
CBM79
GT84
GH48
GH13_8
GH5_18
CBM4
GH5_8
GH5_1
GT2
CBM12
GH79
CBM14
CBM65
PL31
CBM78
GH19
AP1
AP2
Difference function boxplot plot of Cazy_level2

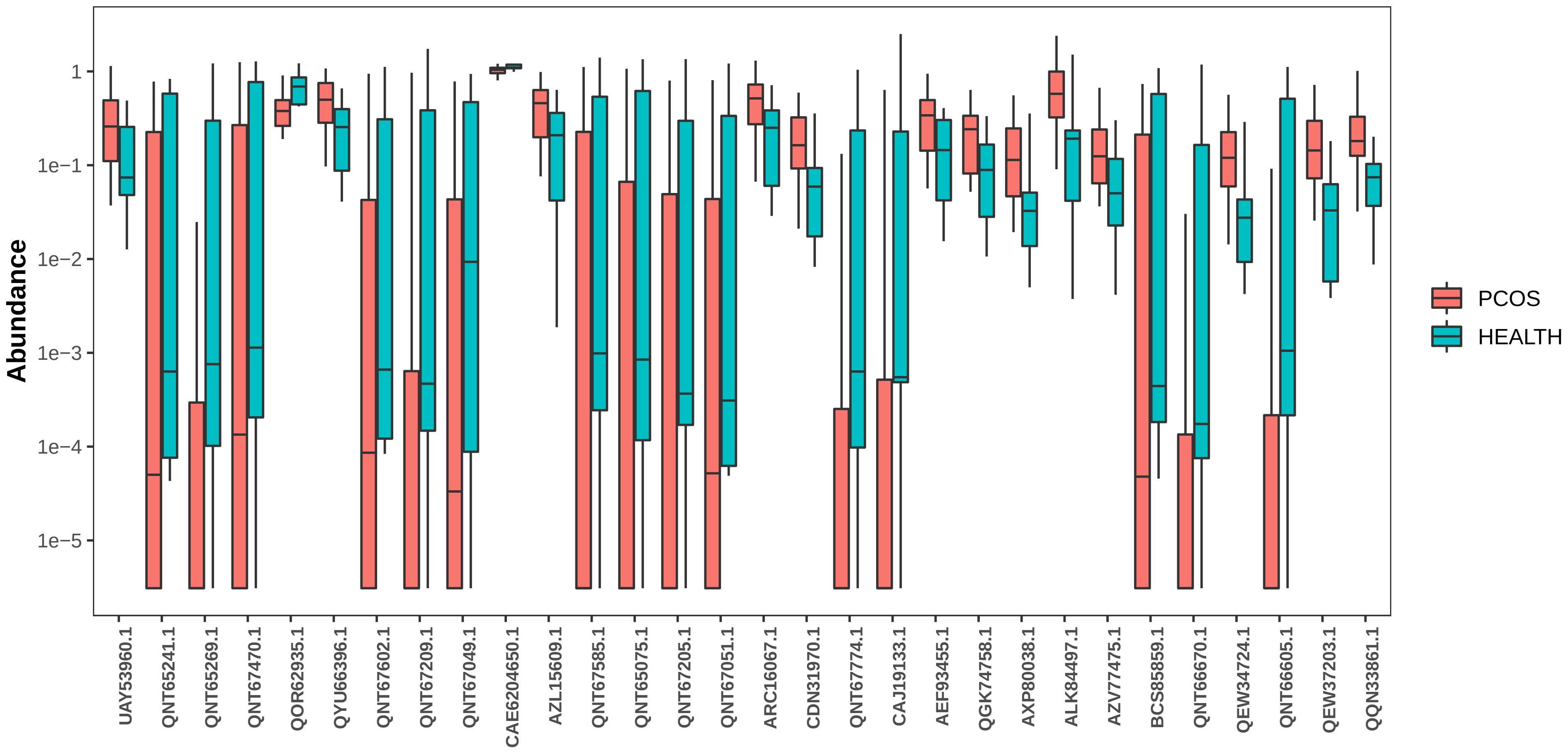

Difference function boxplot plot of Cazy_level3

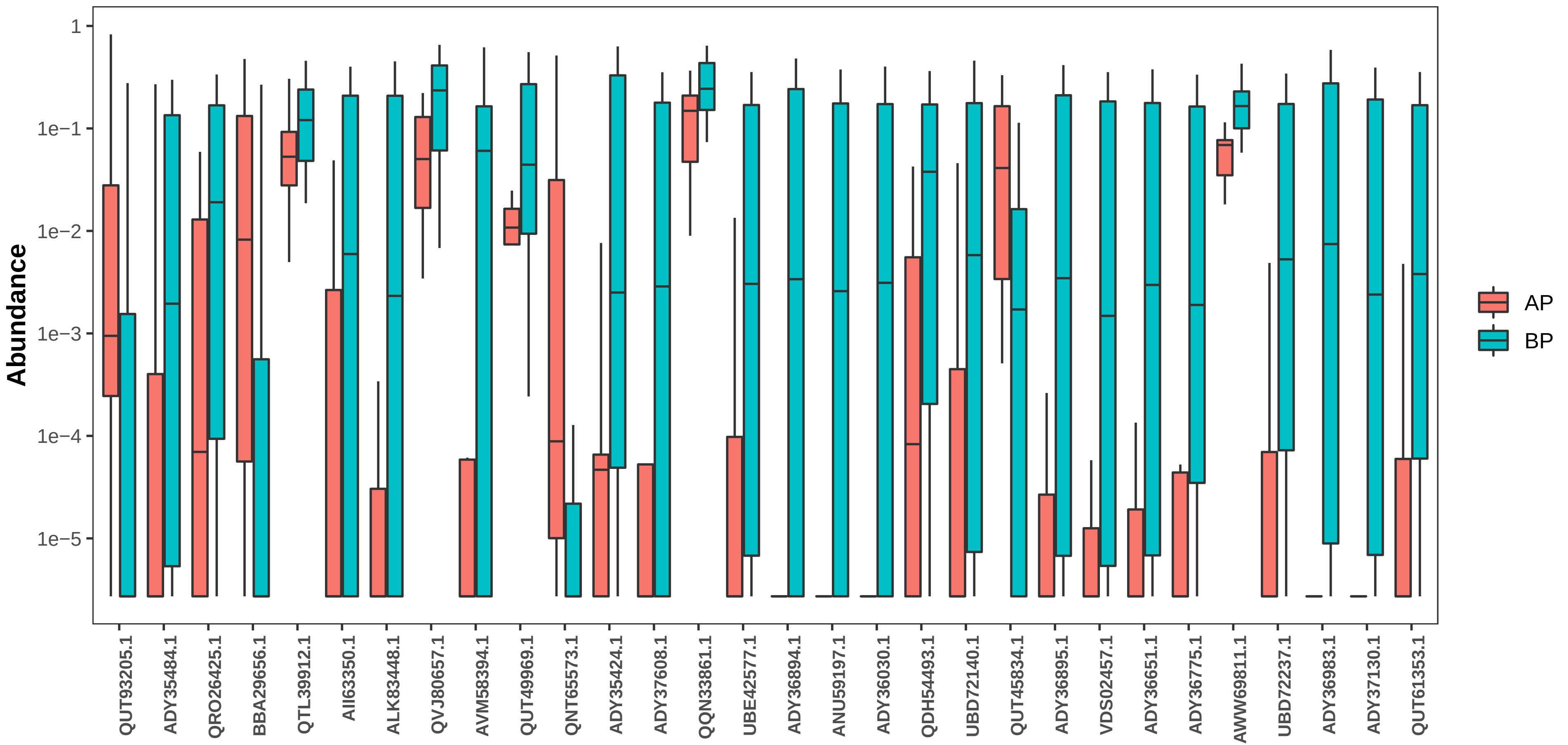

Difference function boxplot plot of Cazy_level3

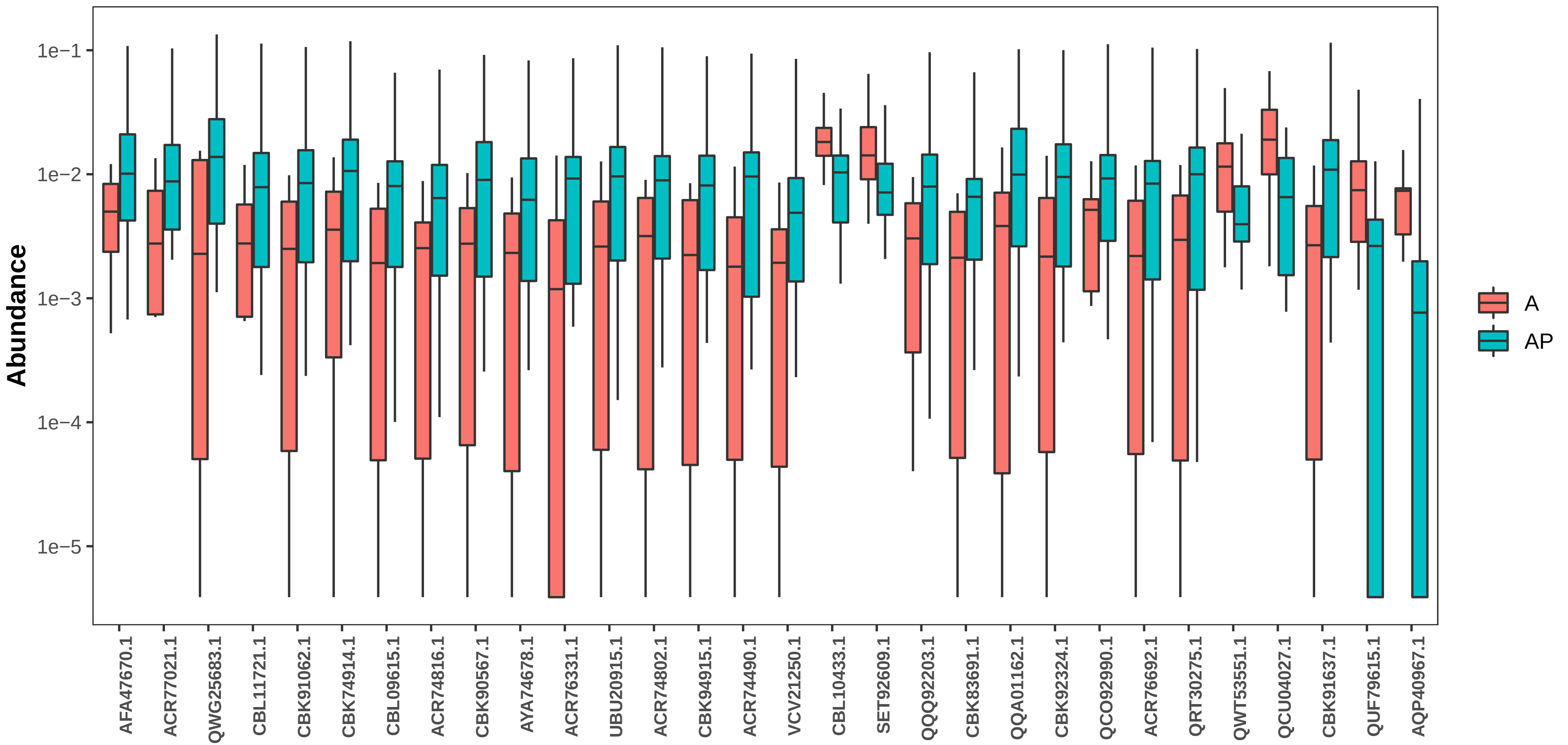

Difference function boxplot plot of Cazy_level3

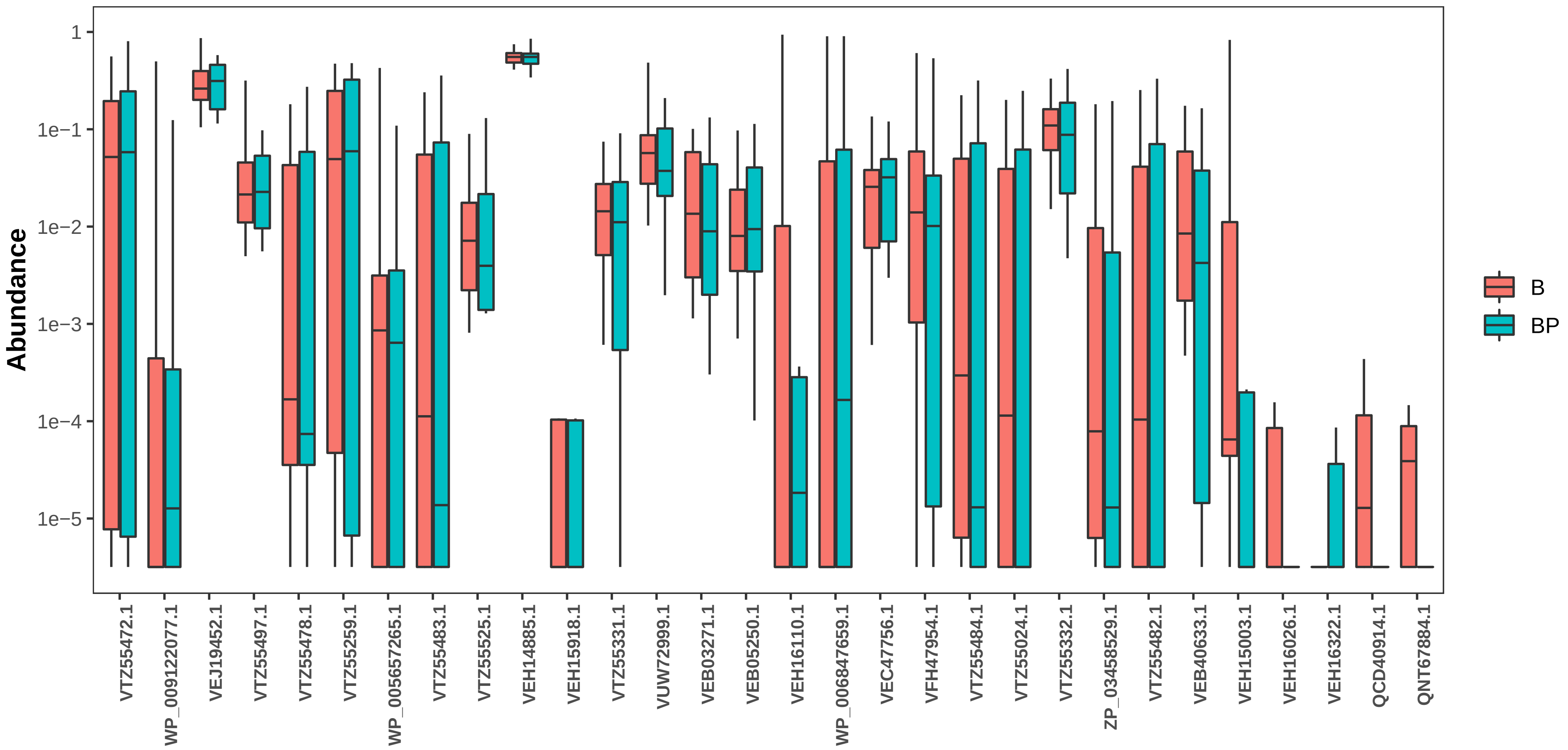

Difference function boxplot plot of Cazy_level3

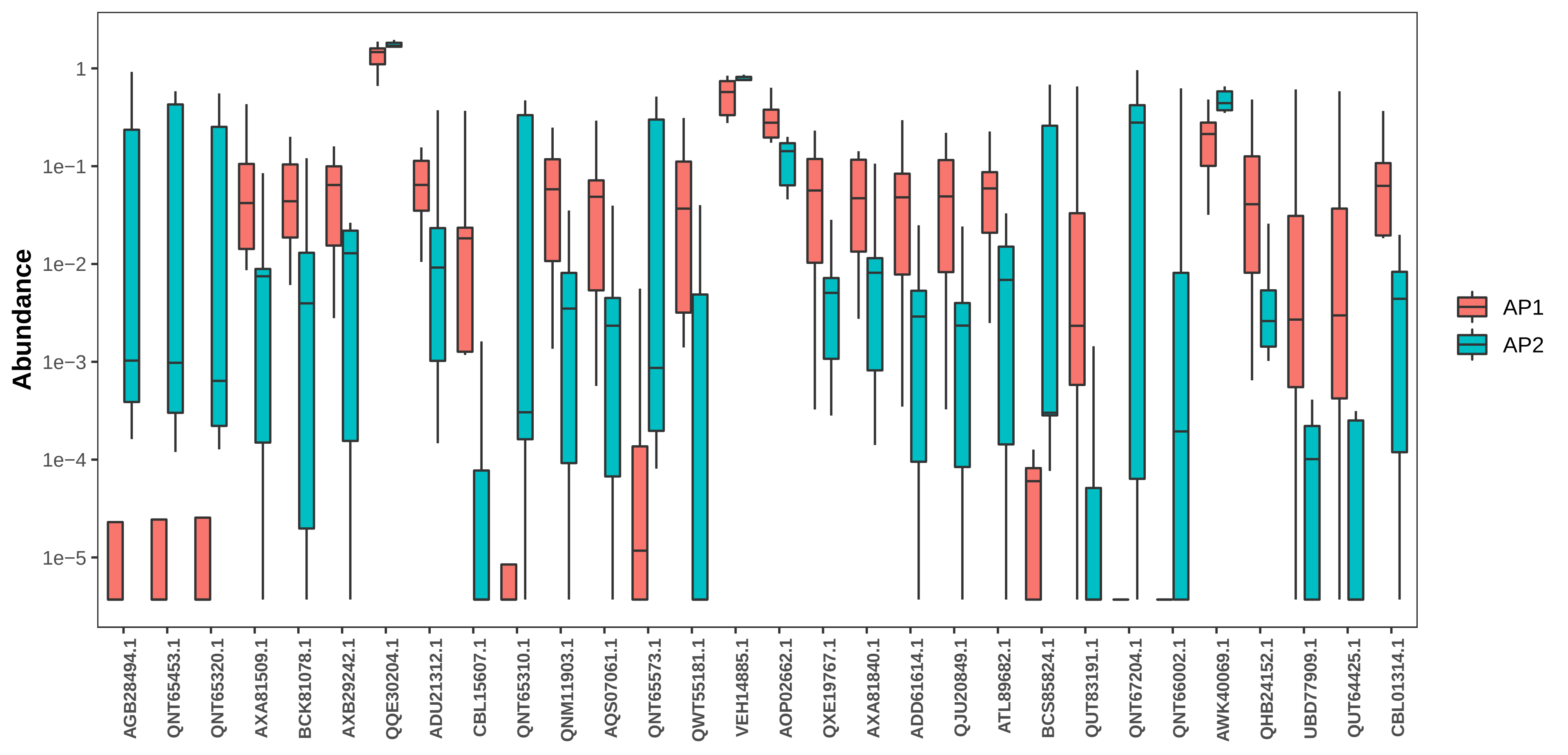

Difference function boxplot plot of Cazy_level3

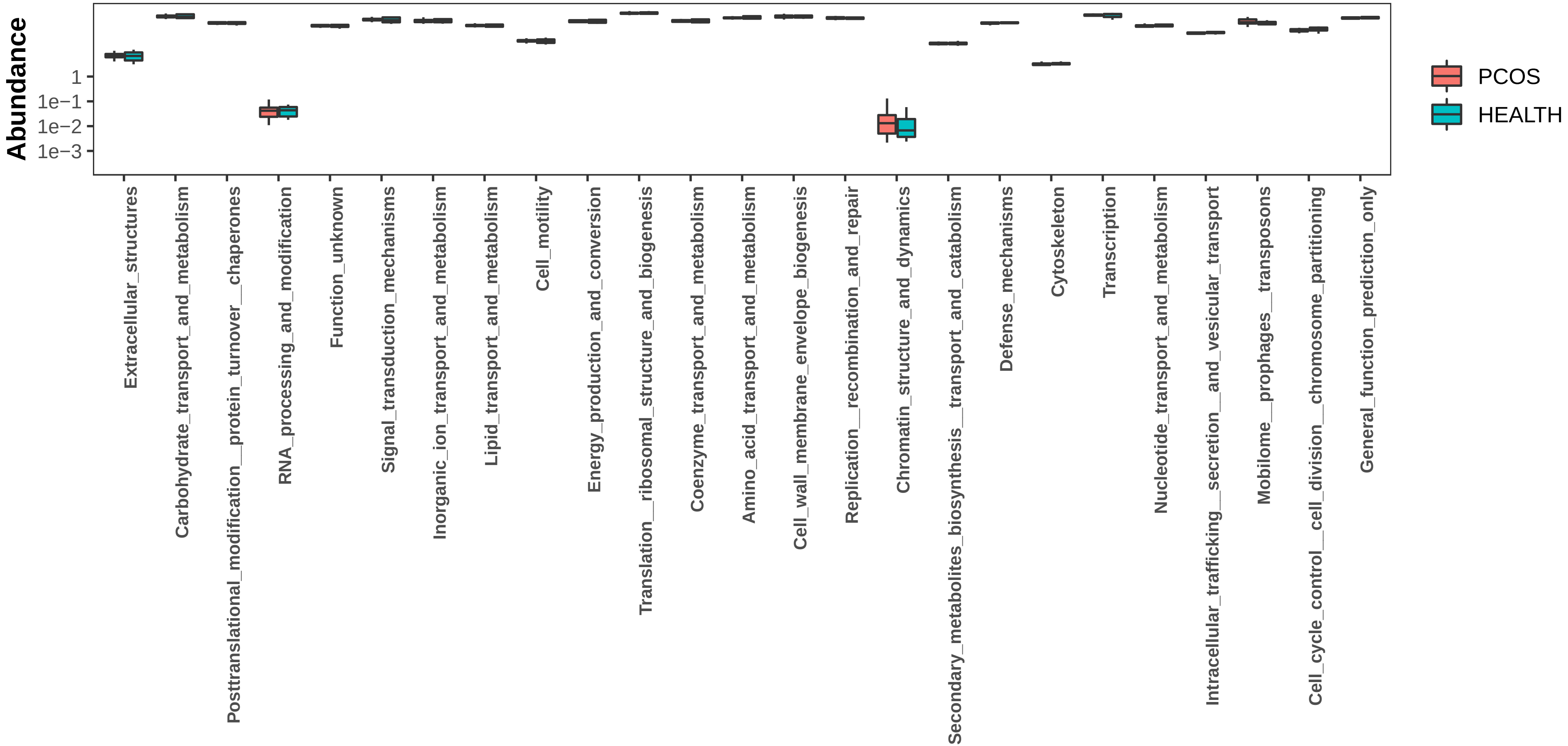

Difference function boxplot plot of Cog

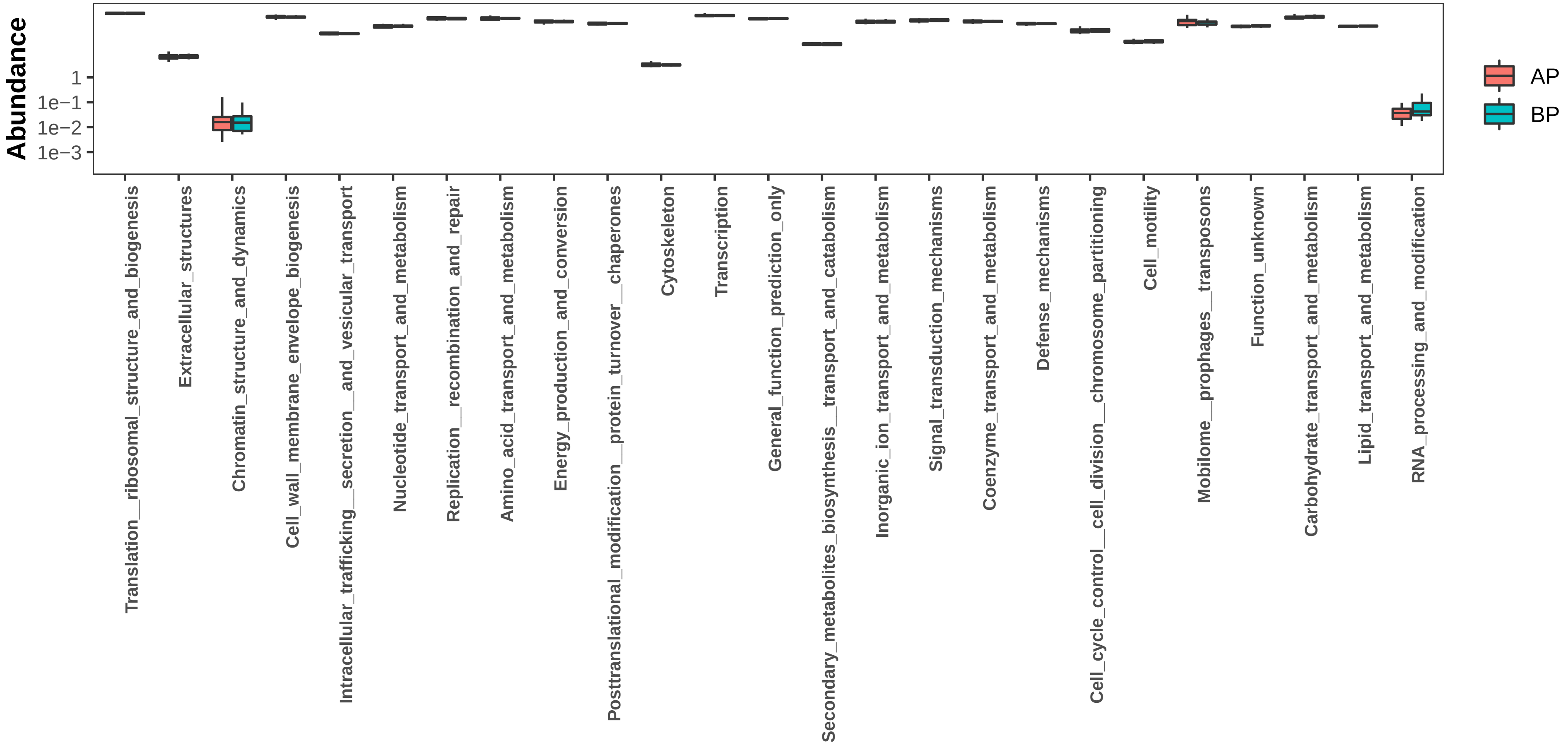

Difference function boxplot plot of Cog

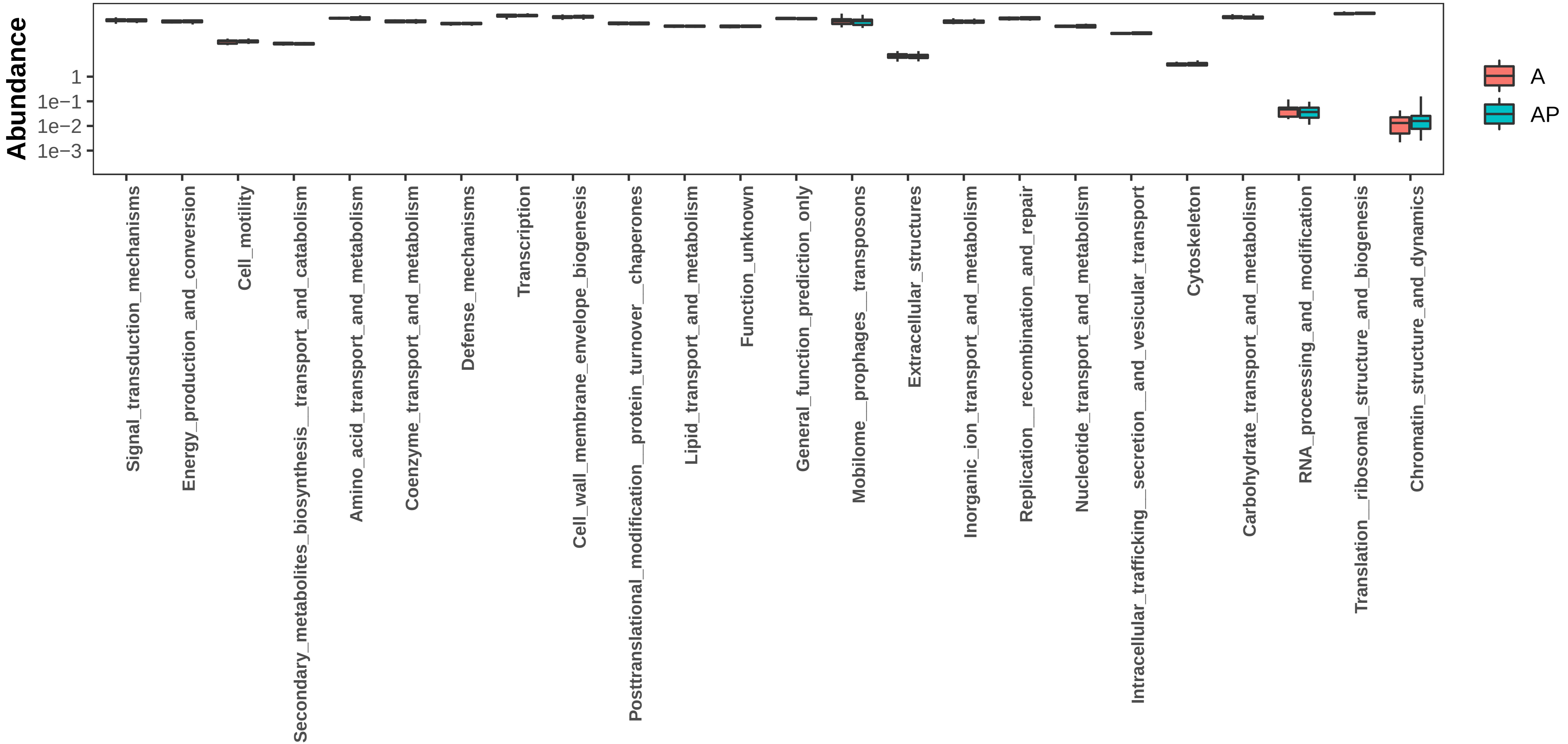

Difference function boxplot plot of Cog

Difference function boxplot plot of Cog

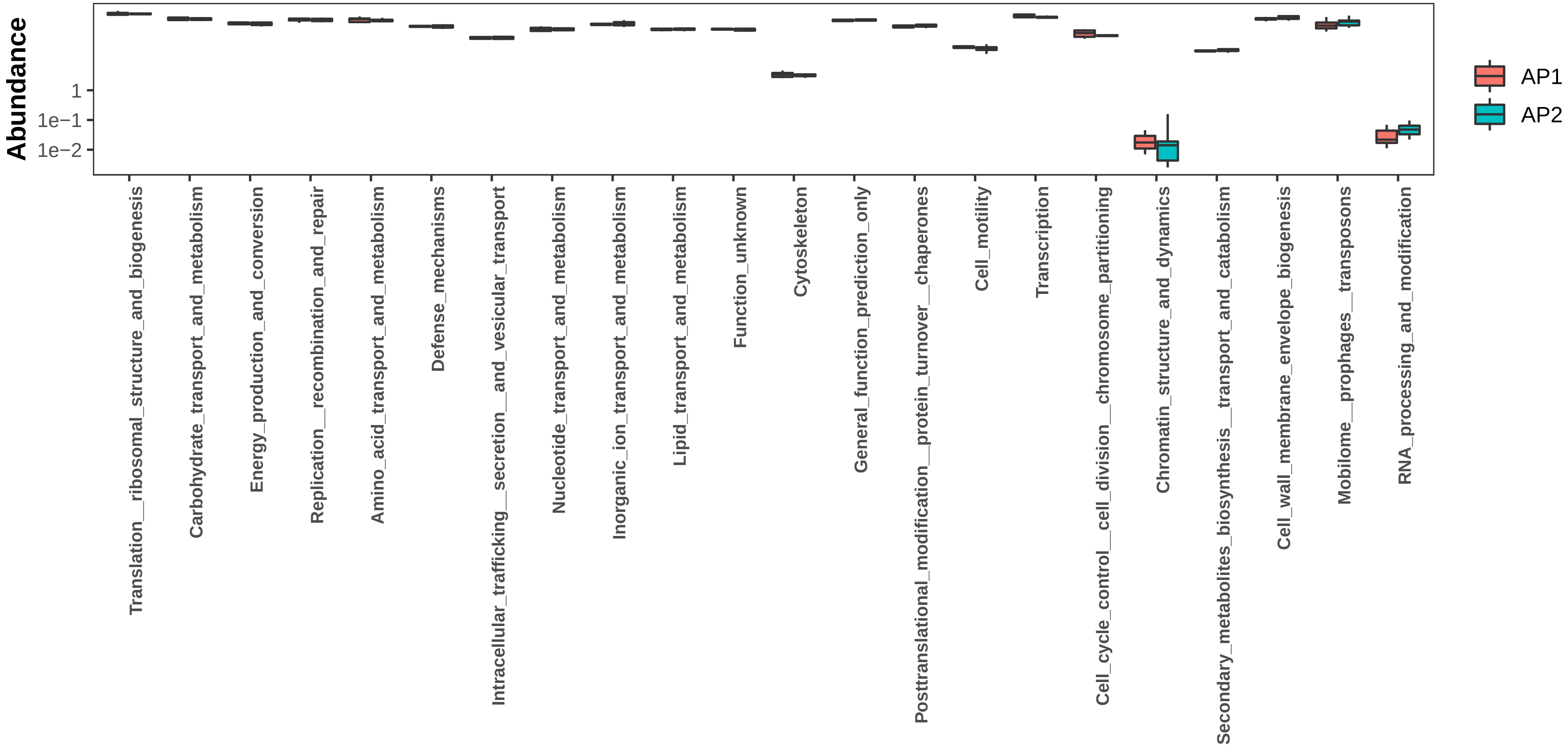

Difference function boxplot plot of Cog

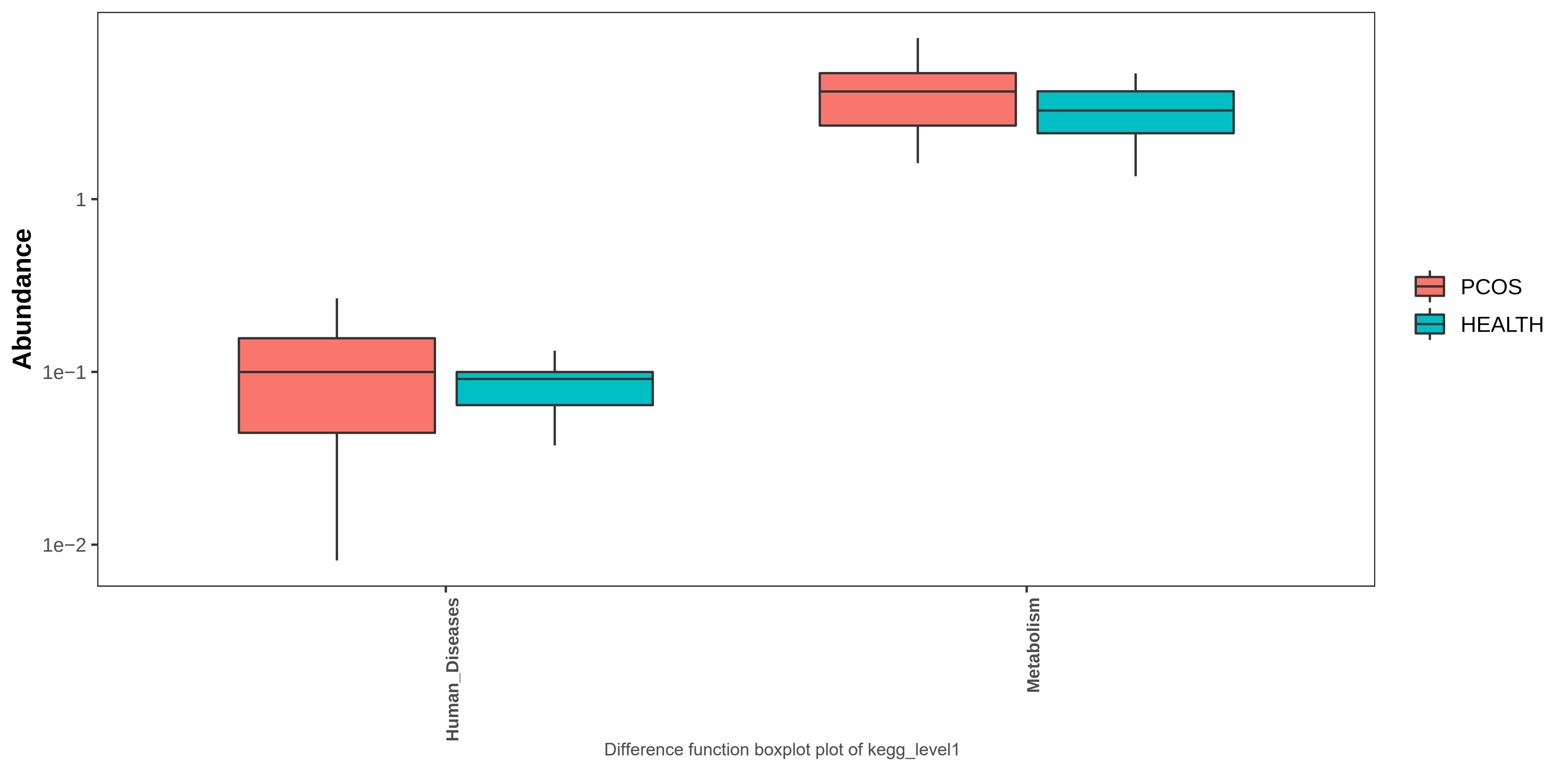
Abundance
1
1e−1
1e−2
Human_Diseases
Metabolism
PCOS
HEALTH
Difference function boxplot plot of kegg_level1

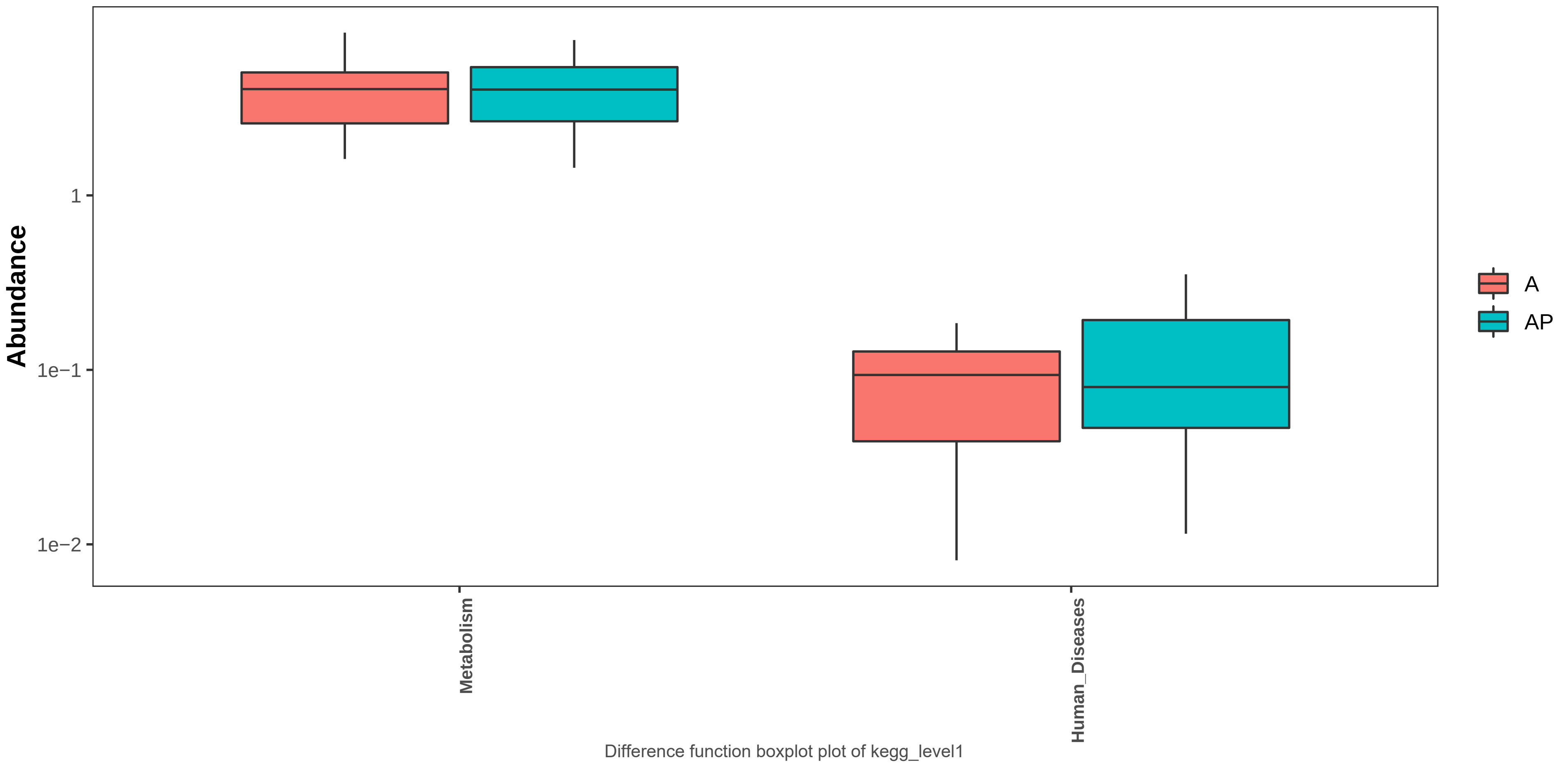
Abundance
1
1e−1
1e−2
Metabolism
Human_Diseases
A
AP
Difference function boxplot plot of kegg_level1

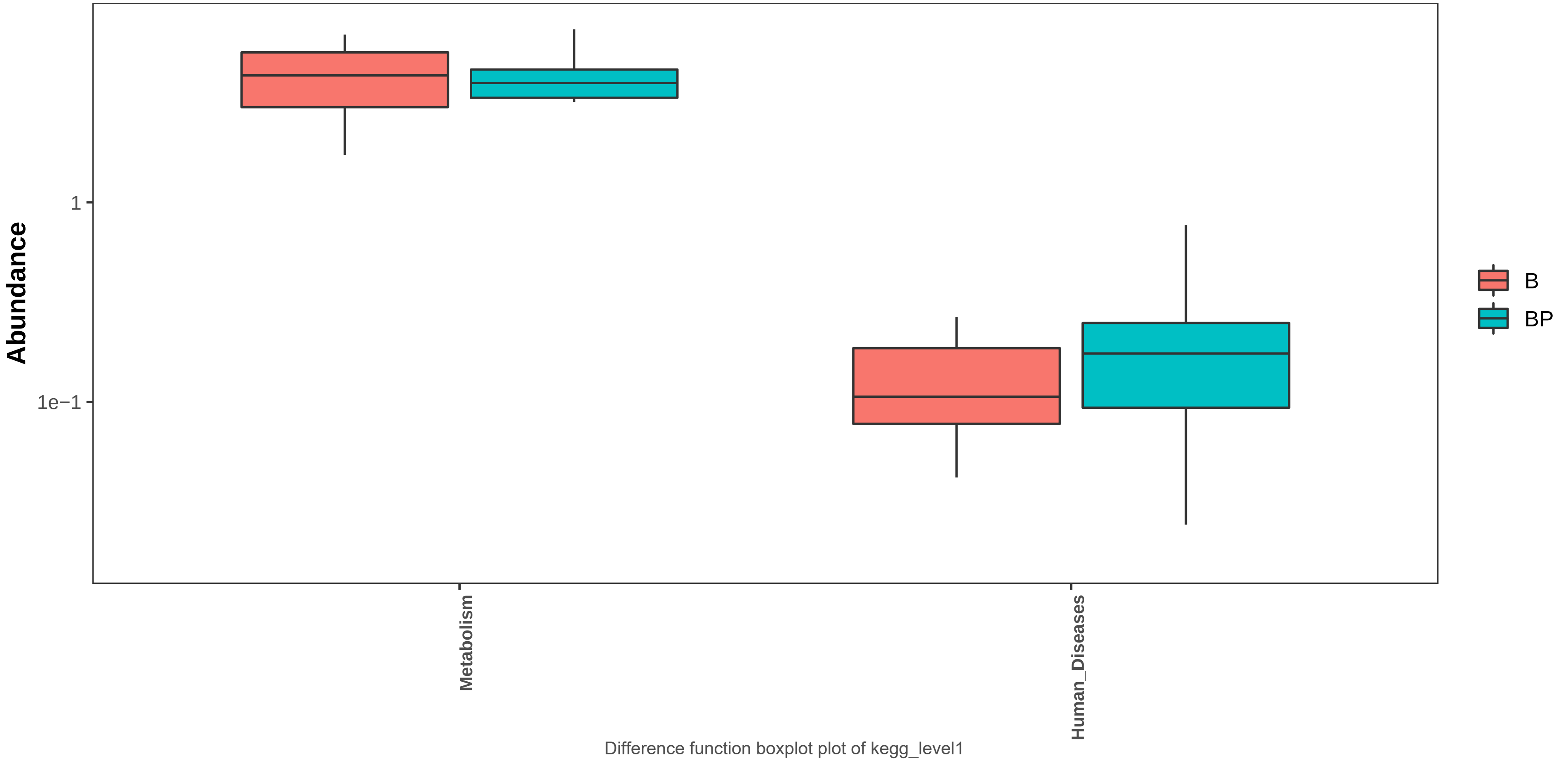

Abundance
1
1e−1
Metabolism
Human_Diseases
B
BP
Difference function boxplot plot of kegg_level1

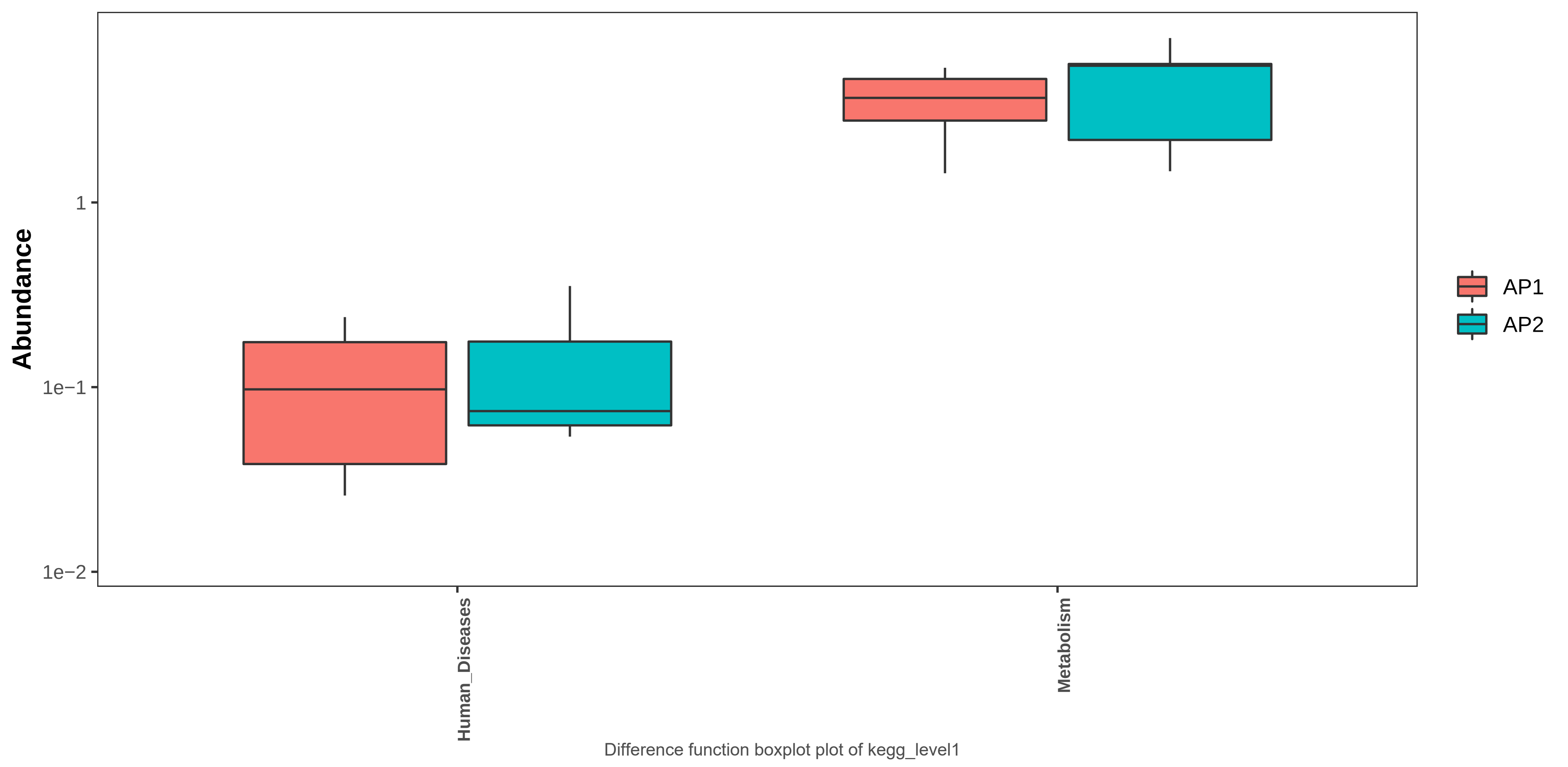

Abundance
1
1e−1
1e−2
Human_Diseases
Metabolism
AP1
AP2
Difference function boxplot plot of kegg_level1

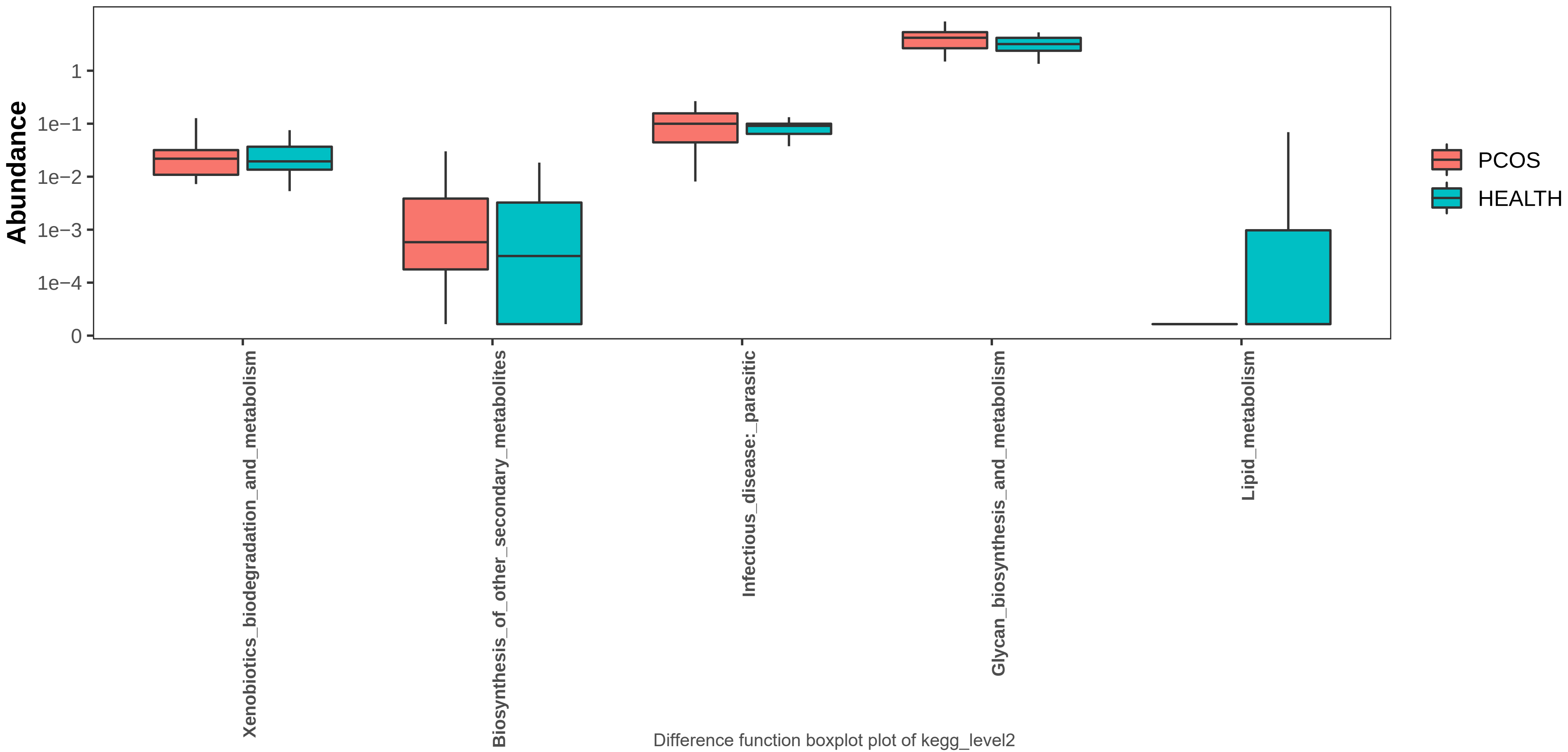
Abundance
1
1e−1
1e−2
1e−3
1e−4
0
PCOS
HEALTH
Xenobiotics_biodegradation_and_metabolism
Biosynthesis_of_other_secondary_metabolites
Infectious_disease:_parasitic
Glycan_biosynthesis_and_metabolism
Lipid_metabolism
Difference function boxplot plot of kegg_level2

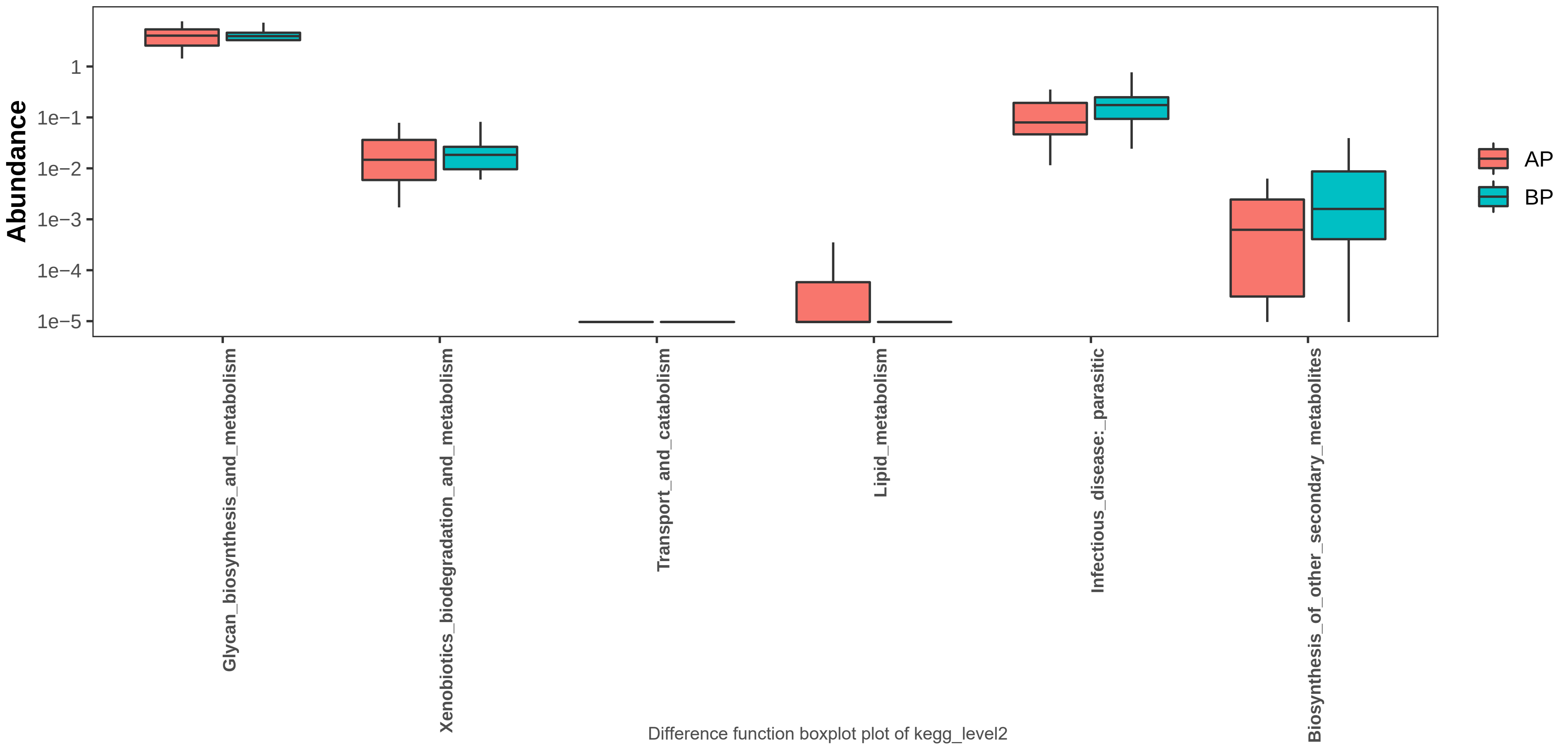
Abundance
1
1e−1
1e−2
1e−3
1e−4
1e−5
AP
BP
Glycan_biosynthesis_and_metabolism
Xenobiotics_biodegradation_and_metabolism
Transport_and_catabolism
Lipid_metabolism
Infectious_disease:_parasitic
Biosynthesis_of_other_secondary_metabolites
Difference function boxplot plot of kegg_level2

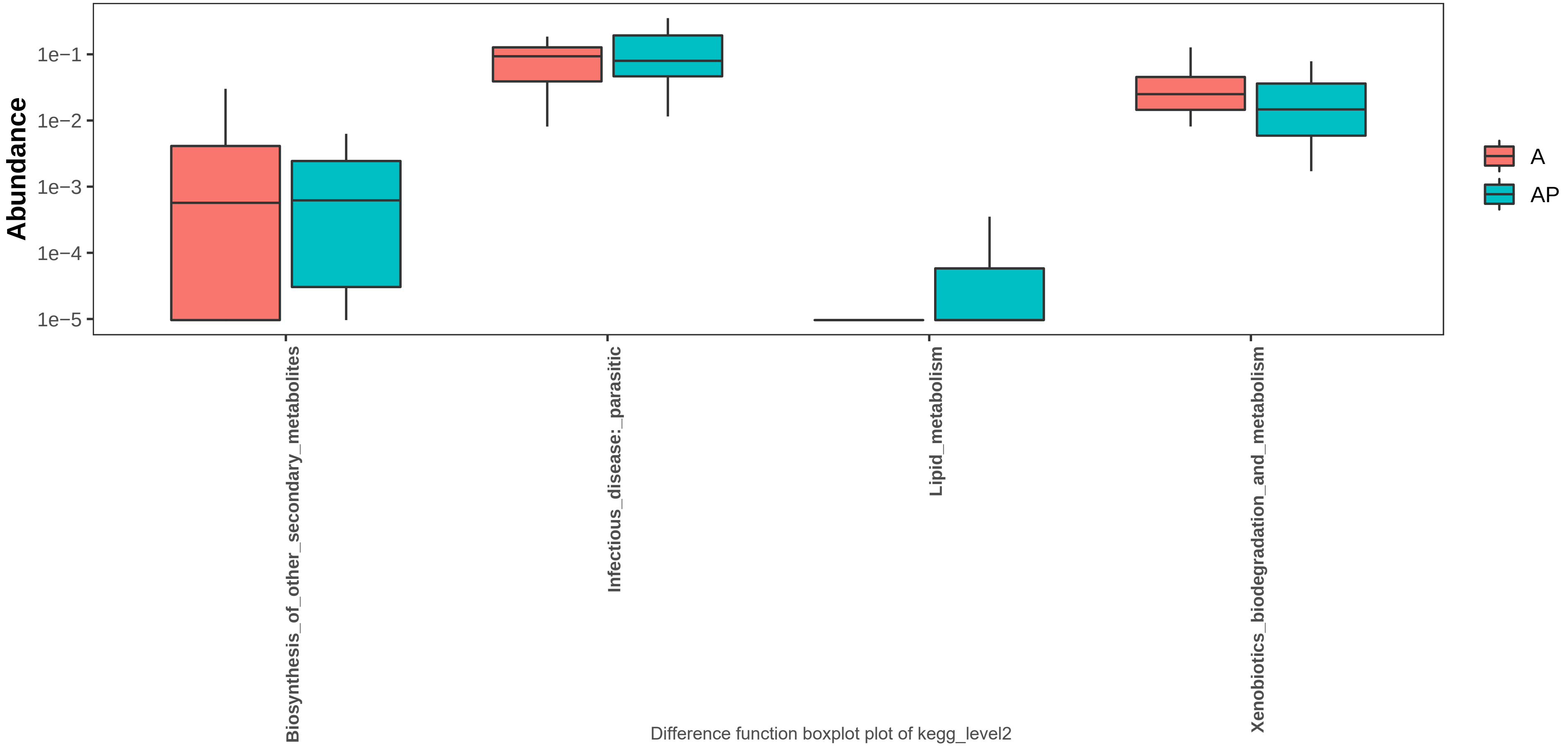

Difference function boxplot plot of kegg_level2

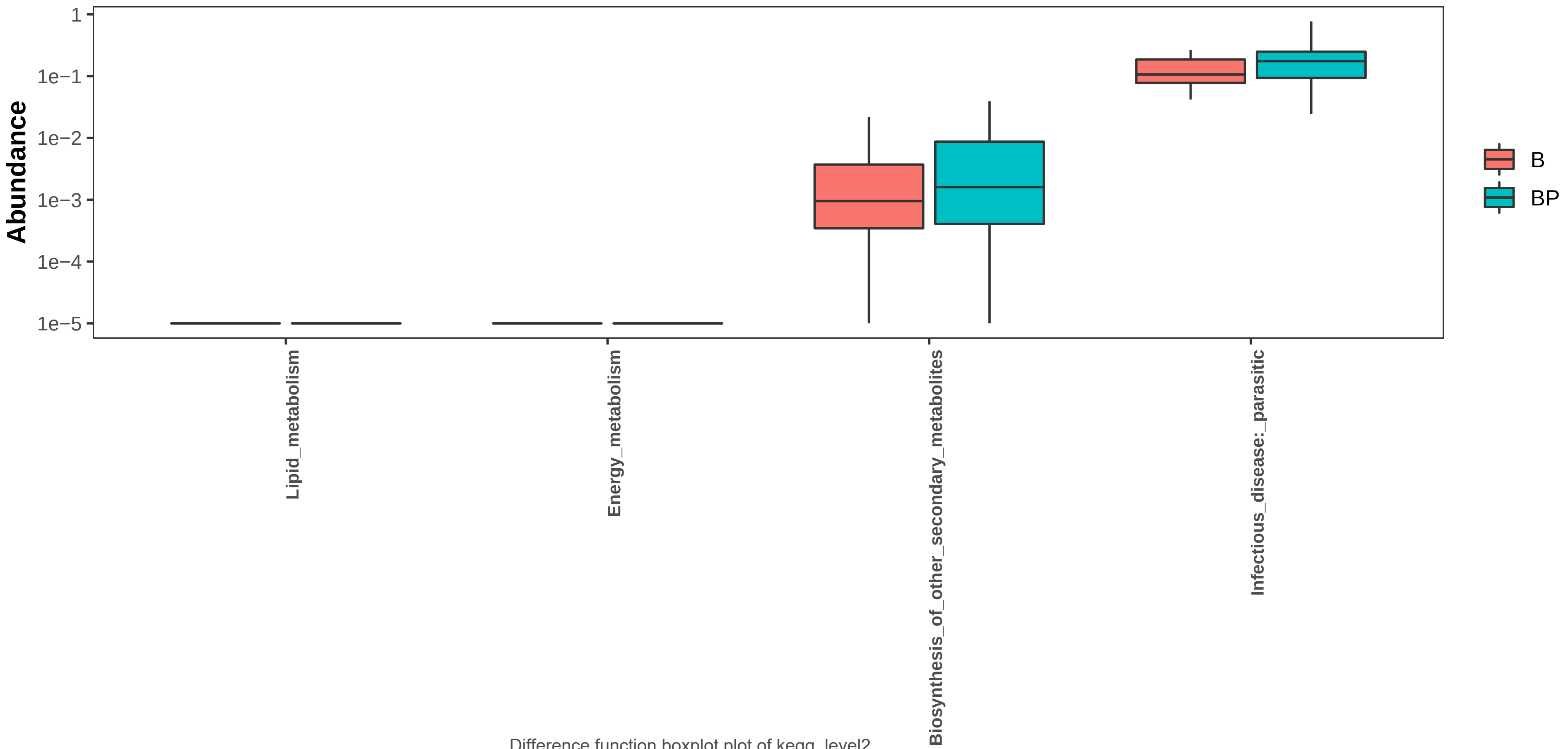

Difference function boxplot plot of kegg_level2

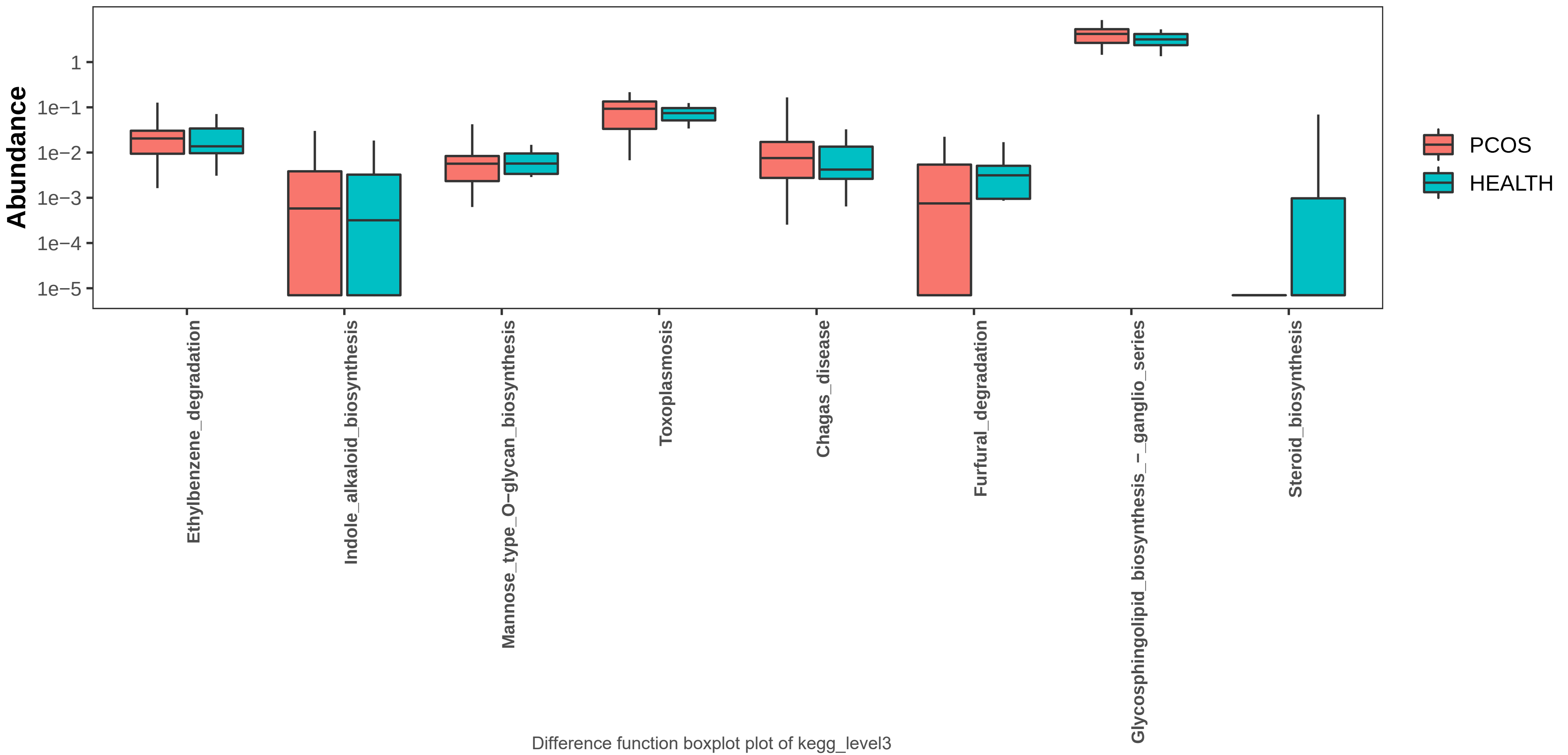

Difference function boxplot plot of kegg_level3

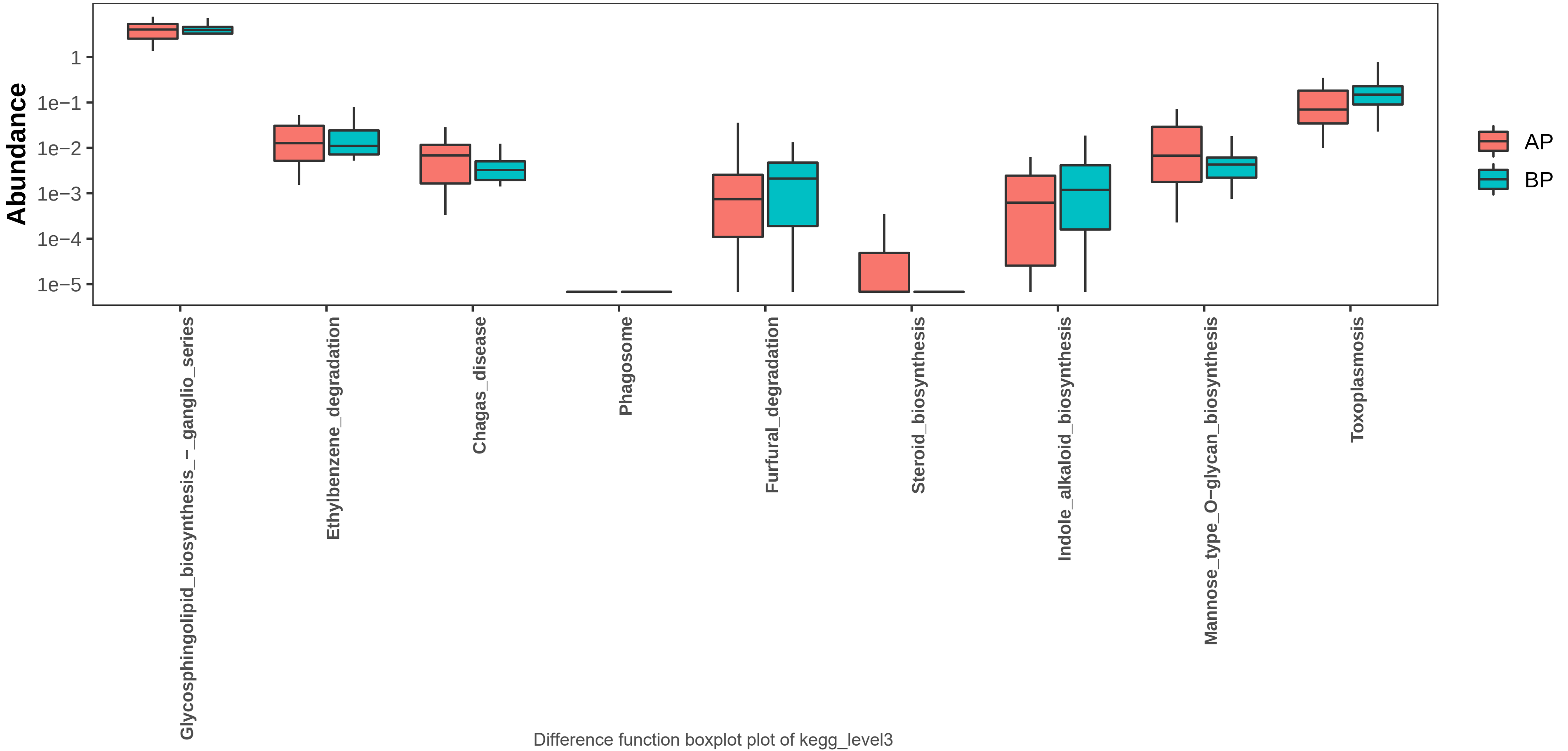

Difference function boxplot plot of kegg_level3

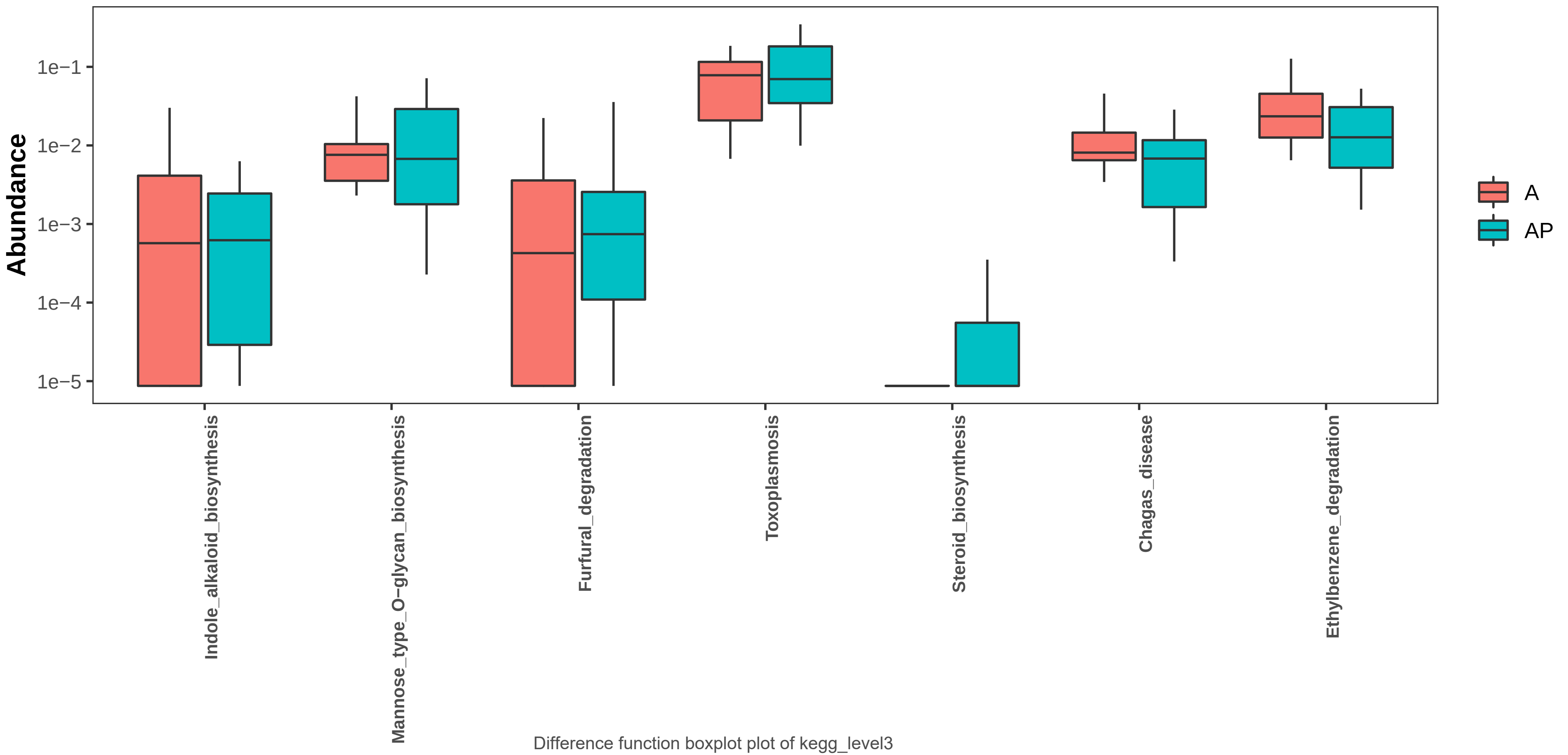

Difference function boxplot plot of kegg_level3

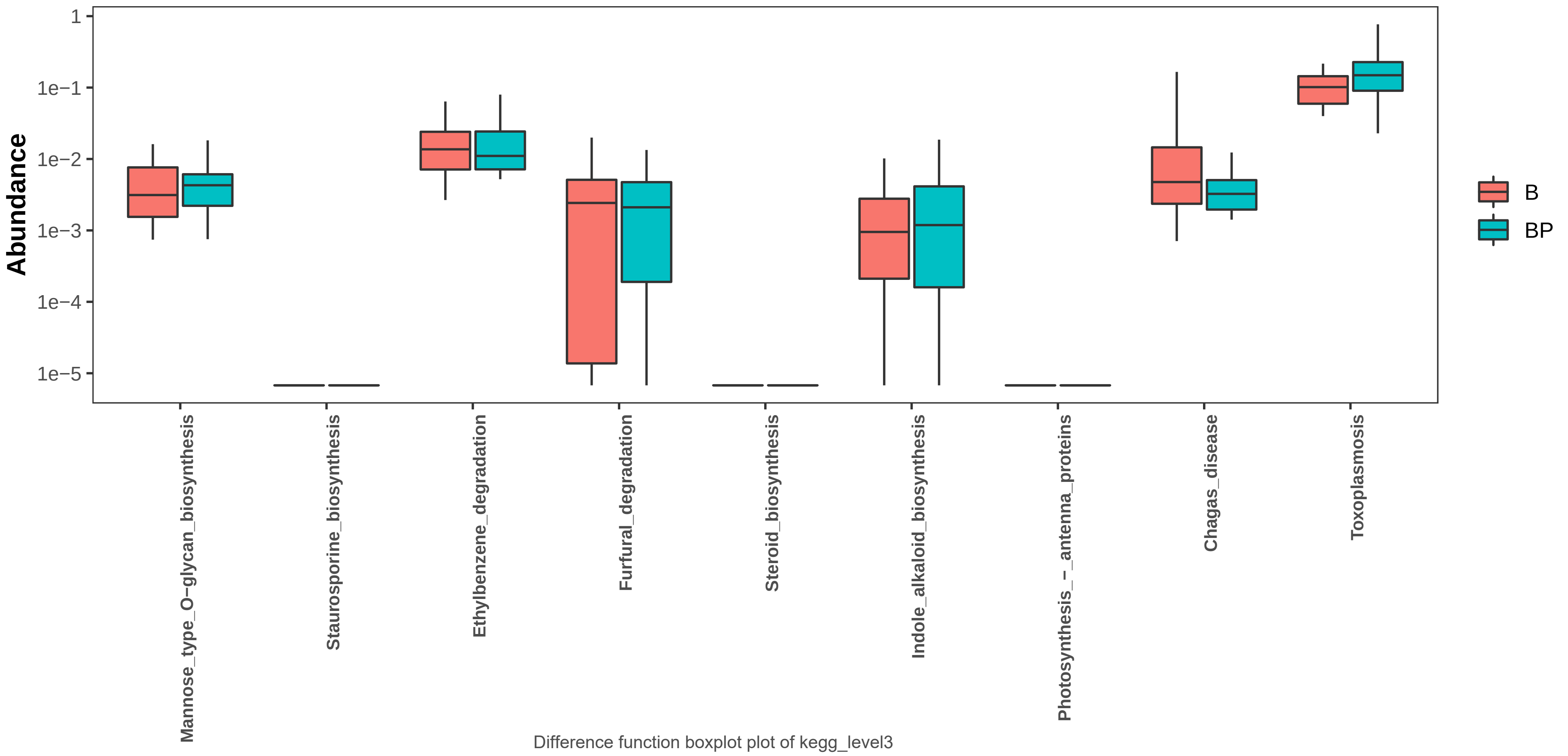
Abundance
1
1e−1
1e−2
1e−3
1e−4
1e−5
B
BP
Mannose_type_O−glycan_biosynthesis
Staurosporine_biosynthesis
Ethylbenzene_degradation
Furfural_degradation
Steroid_biosynthesis
Indole_alkaloid_biosynthesis
Photosynthesis_−_antenna_proteins
Chagas_disease
Toxoplasmosis
Difference function boxplot plot of kegg_level3

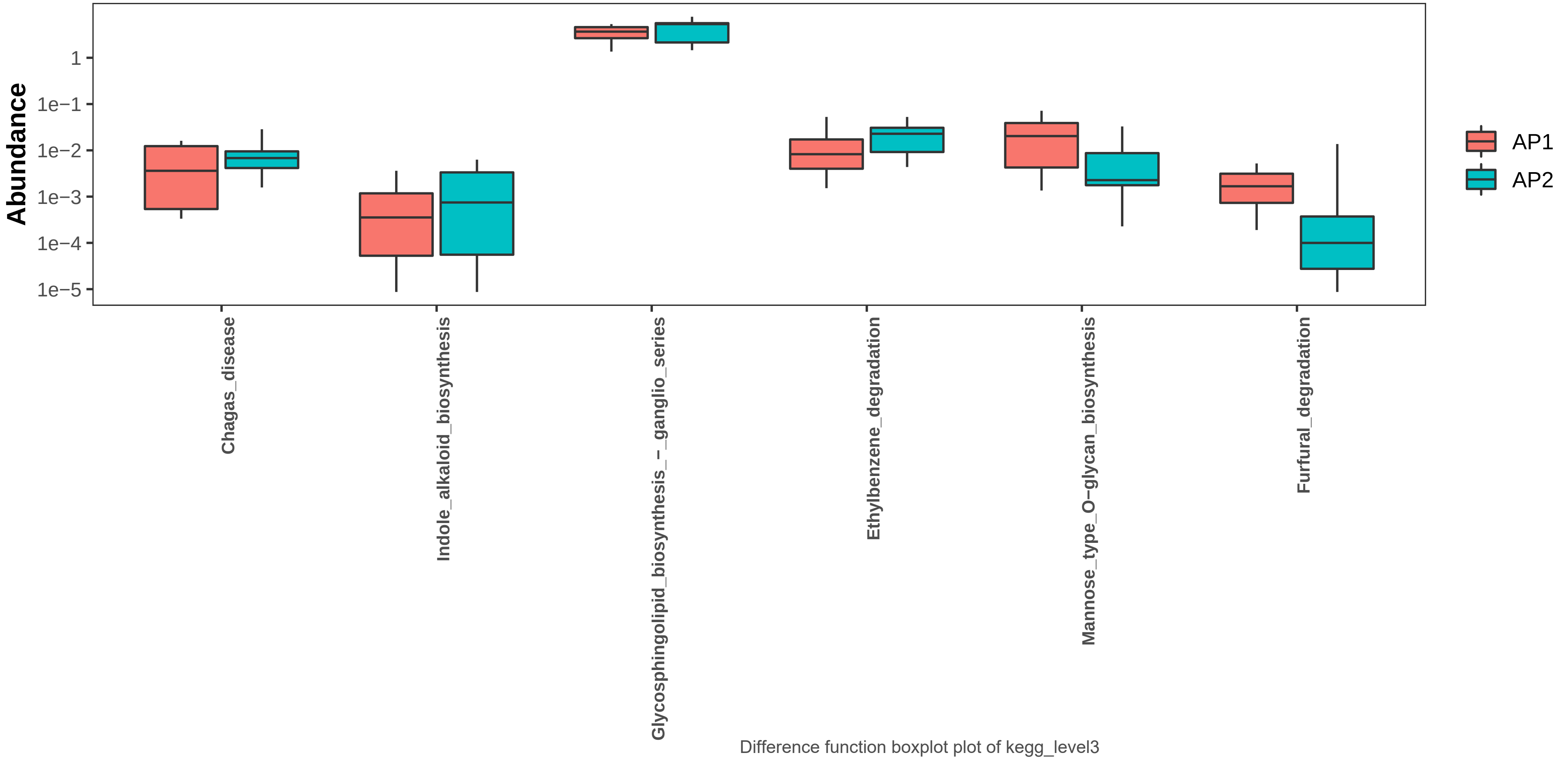

Abundance
1
1e−1
1e−2
1e−3
1e−4
1e−5
AP1
AP2
Chagas_disease
Indole_alkaloid_biosynthesis
Glycosphingolipid_biosynthesis_−_ganglio_series
Ethylbenzene_degradation
Mannose_type_O−glycan_biosynthesis
Furfural_degradation
Difference function boxplot plot of kegg_level3

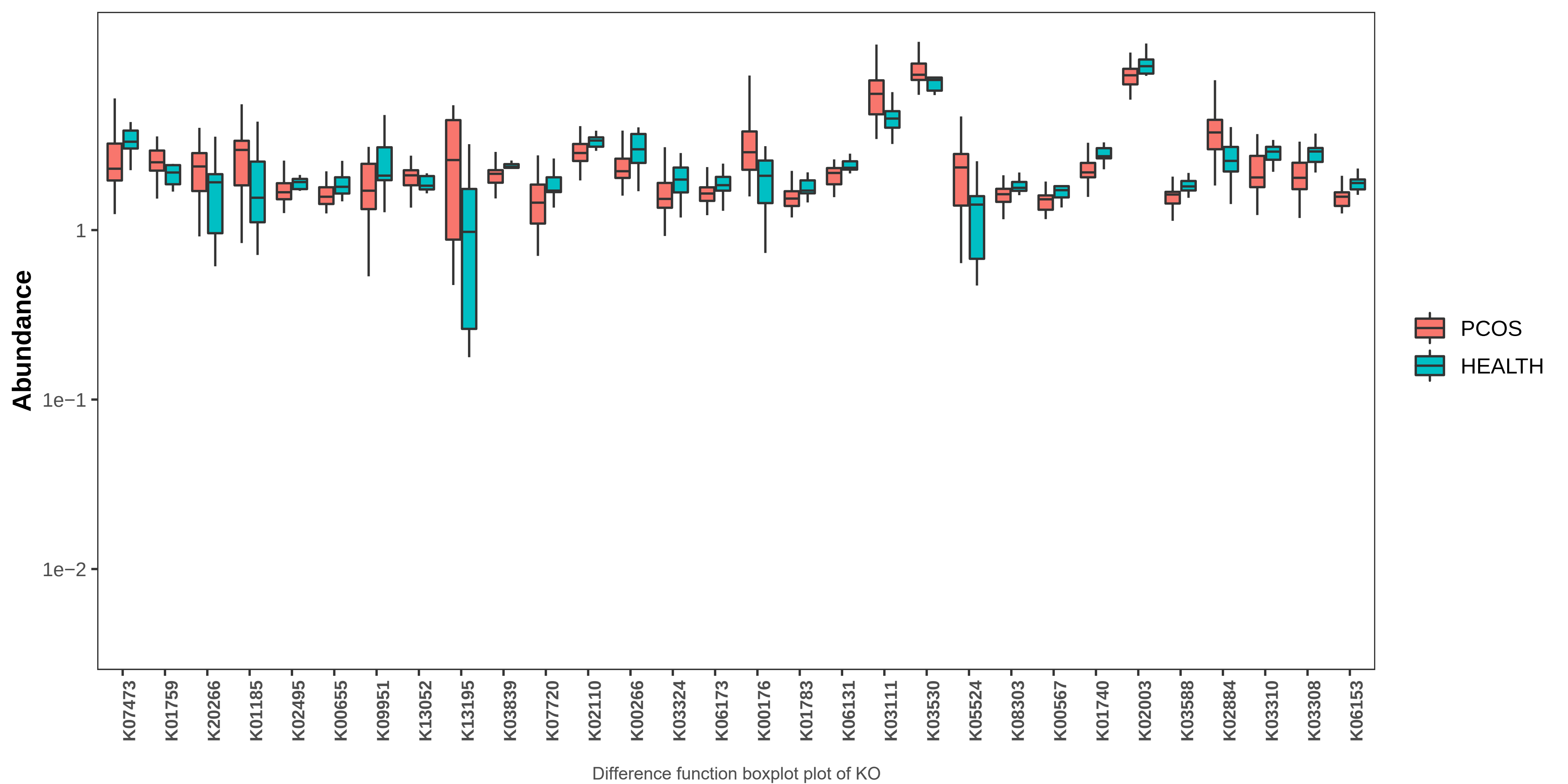

Abundance
1
1e−1
1e−2
K07473
K01759
K20266
K01185
K02495
K00655
K09951
K13052
K13195
K03839
K07720
K02110
K00266
K03324
K06173
K00176
K01783
K06131
K03111
K03530
K05524
K08303
K00567
K01740
K02003
K03588
K02884
K03310
K03308
K06153
PCOS
HEALTH
Difference function boxplot plot of KO

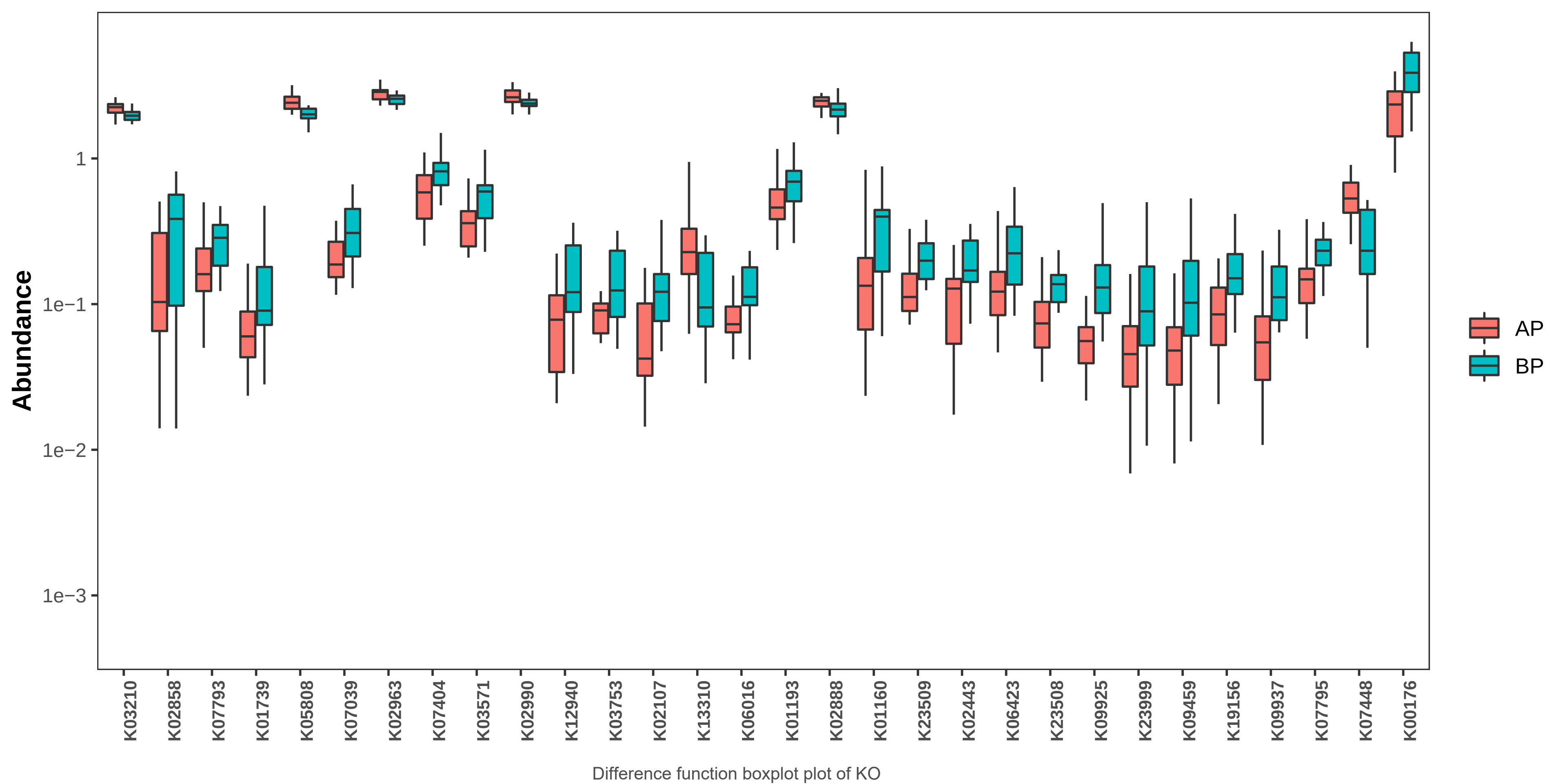

Abundance
1
1e−1
1e−2
1e−3
K03210
K02858
K07793
K01739
K05808
K07039
K02963
K07404
K03571
K02990
K12940
K03753
K02107
K13310
K06016
K01193
K02888
K01160
K23509
K02443
K06423
K23508
K09925
K23999
K09459
K19156
K09937
K07795
K07448
K00176
AP
BP
Difference function boxplot plot of KO

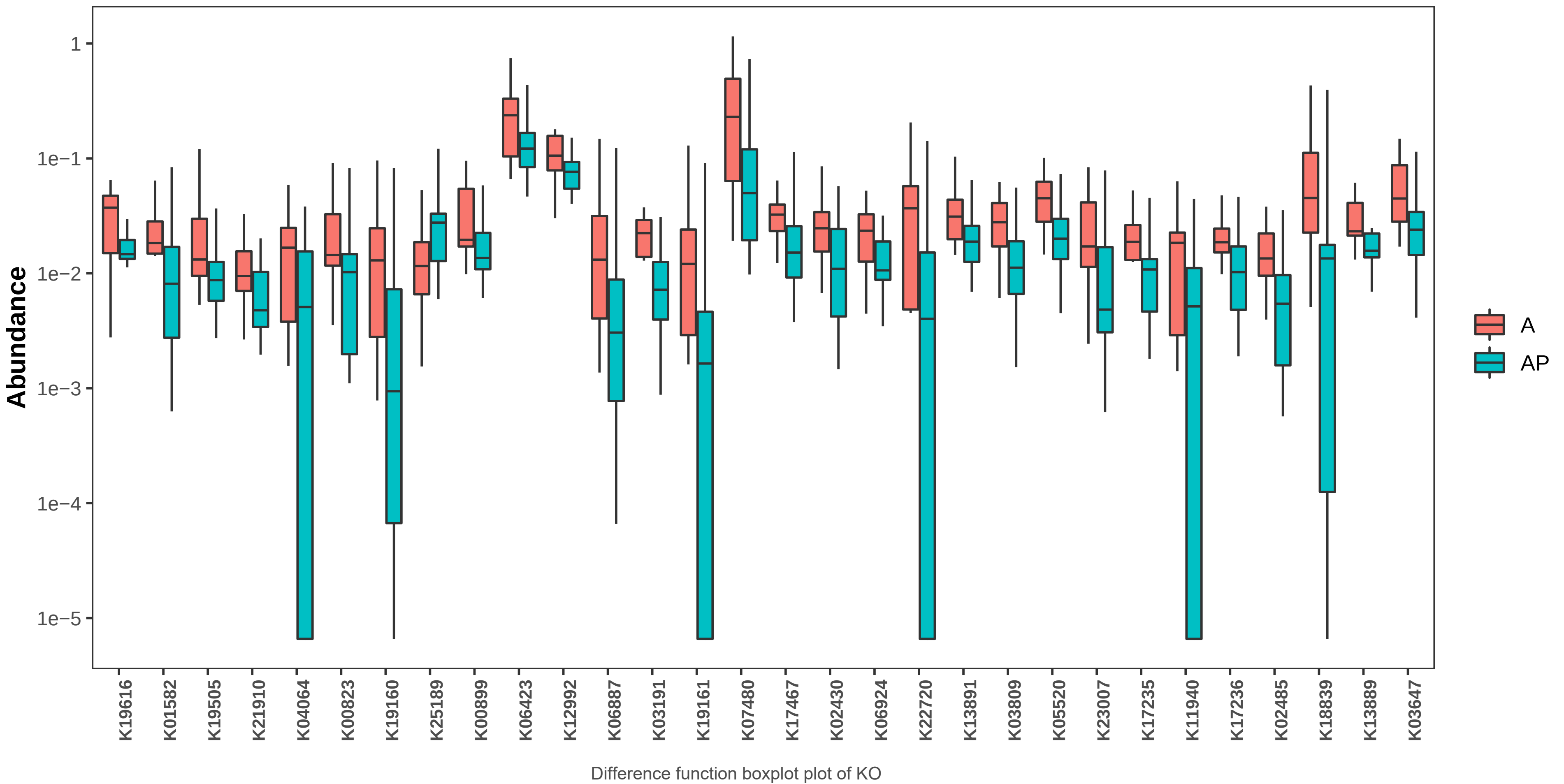

Abundance
1
1e−1
1e−2
1e−3
1e−4
1e−5
K19616
K01582
K19505
K21910
K04064
K00823
K19160
K25189
K00899
K06423
K12992
K06887
K03191
K19161
K07480
K17467
K02430
K06924
K22720
K13891
K03809
K05520
K23007
K17235
K11940
K17236
K02485
K18839
K13889
K03647
A
AP
Difference function boxplot plot of KO

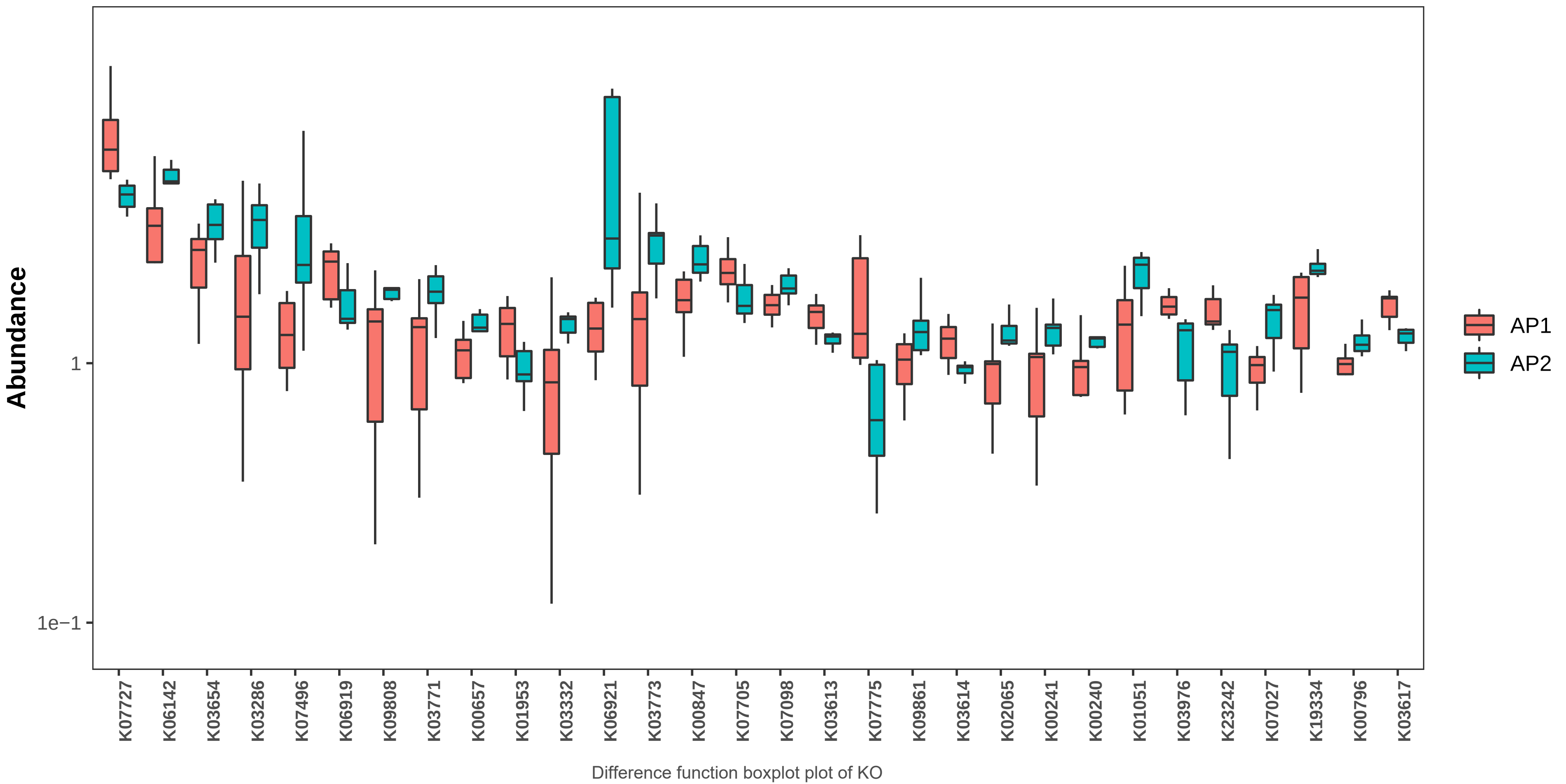

Abundance
1
1e−1
K07727
K06142
K03654
K03286
K07496
K06919
K09808
K03771
K00657
K01953
K03332
K06921
K03773
K00847
K07705
K07098
K03613
K07775
K09861
K03614
K02065
K00241
K00240
K01051
K03976
K23242
K07027
K19334
K00796
K03617
Difference function boxplot plot of KO
AP1
AP2

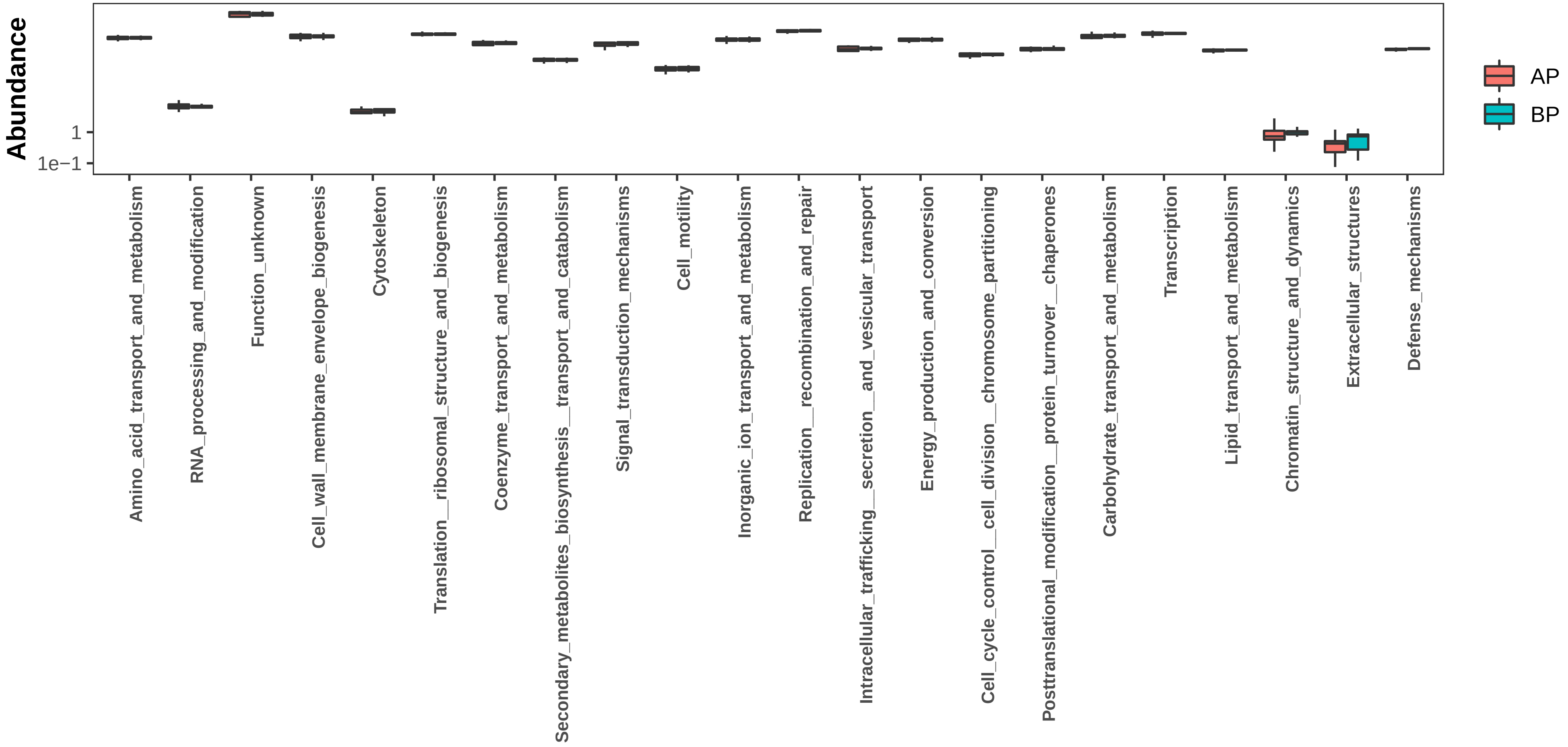

Difference function boxplot plot of Nog

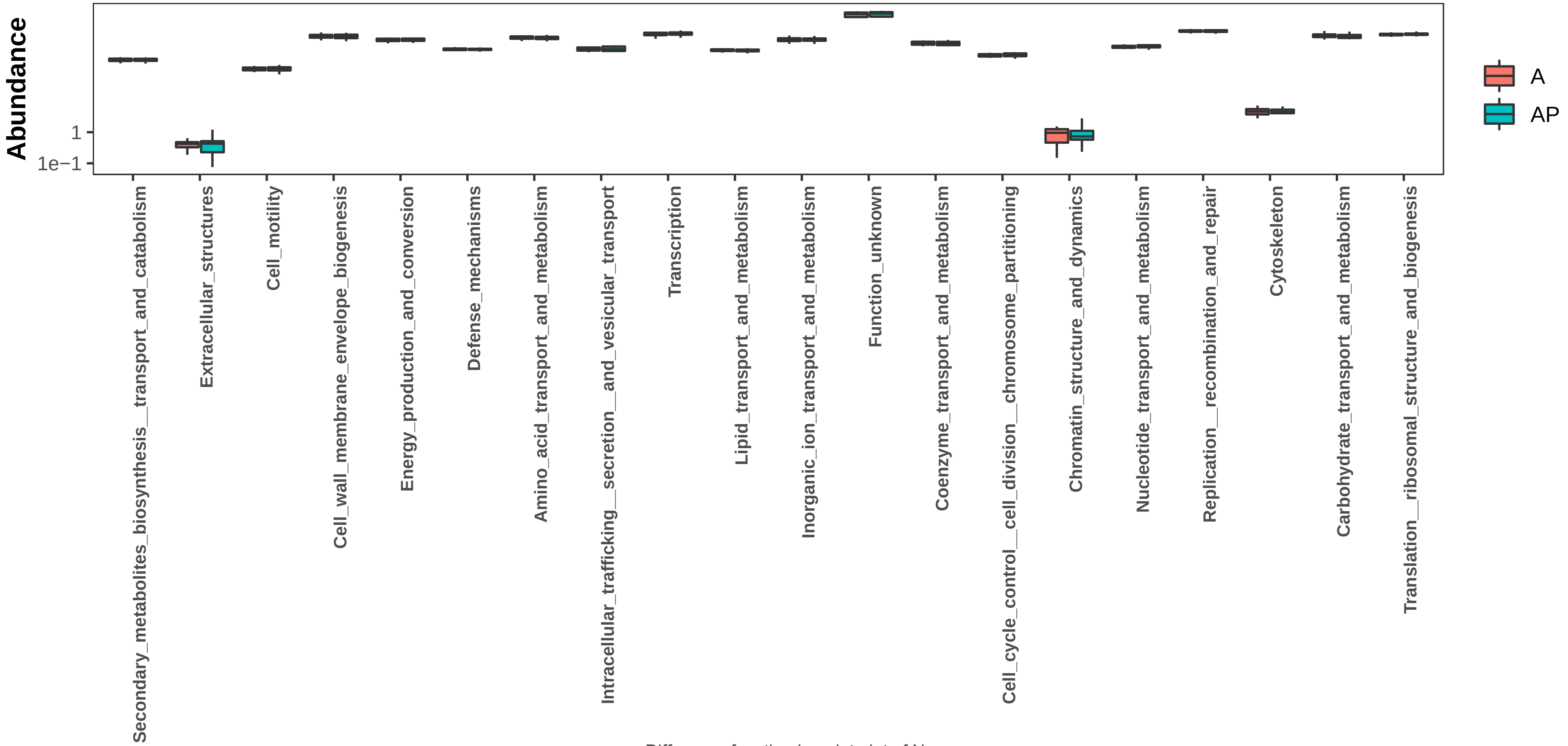

Difference function boxplot plot of Nog

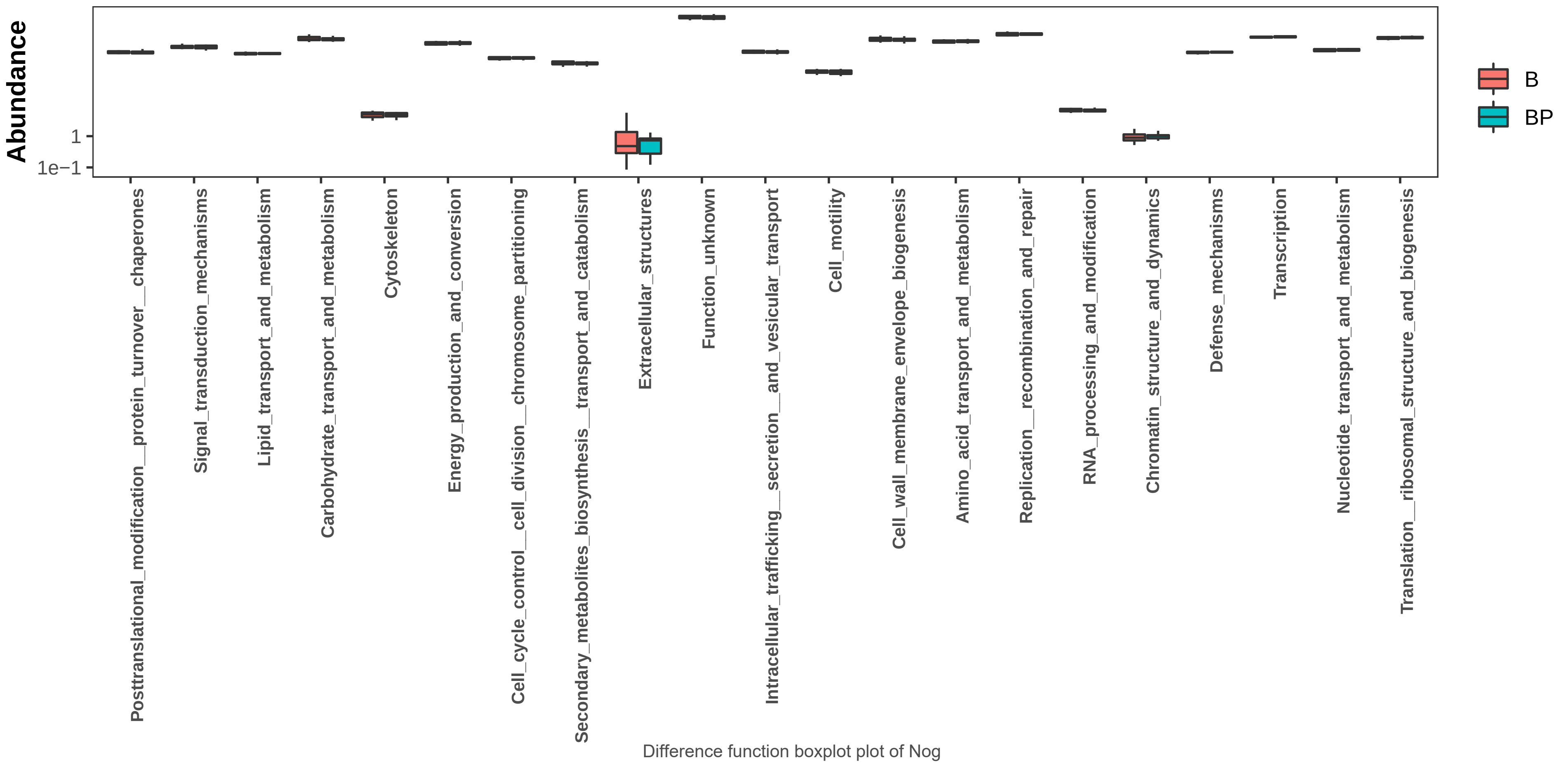

Abundance
1
1e−1
Posttranslational_modification__protein_turnover__chaperones
Signal_transduction_mechanisms
Lipid_transport_and_metabolism
Carbohydrate_transport_and_metabolism
Cytoskeleton
Energy_production_and_conversion
Cell_cycle_control__cell_division__chromosome_partitioning
Secondary_metabolites_biosynthesis__transport_and_catabolism
Extracellular_structures
Function_unknown
Intracellular_trafficking__secretion__and_vesicular_transport
Cell_motility
Cell_wall_membrane_envelope_biogenesis
Amino_acid_transport_and_metabolism
Replication__recombination_and_repair
RNA_processing_and_modification
Chromatin_structure_and_dynamics
Defense_mechanisms
Transcription
Nucleotide_transport_and_metabolism
Translation__ribosomal_structure_and_biogenesis
B
BP
Difference function boxplot plot of Nog

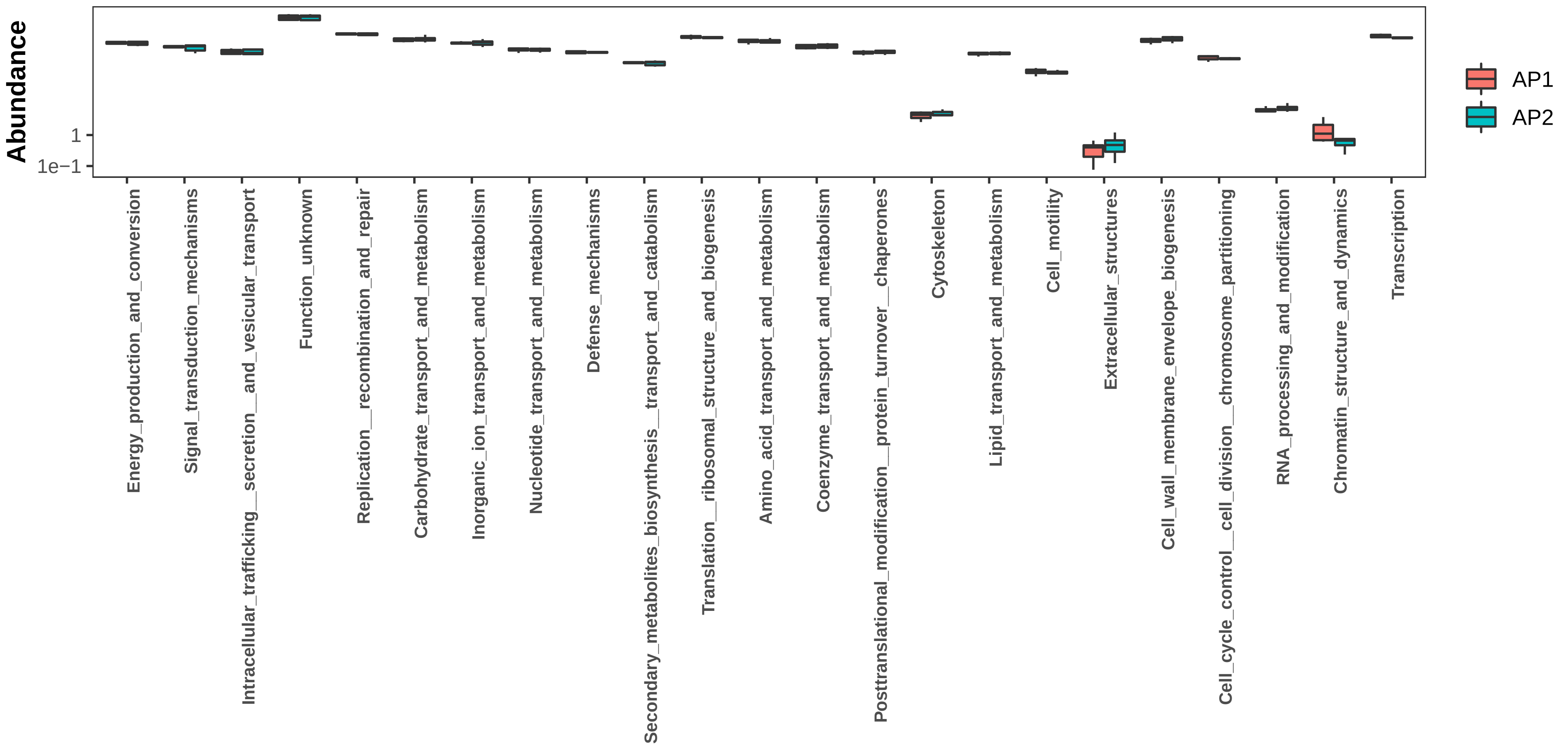

Difference function boxplot plot of Nog

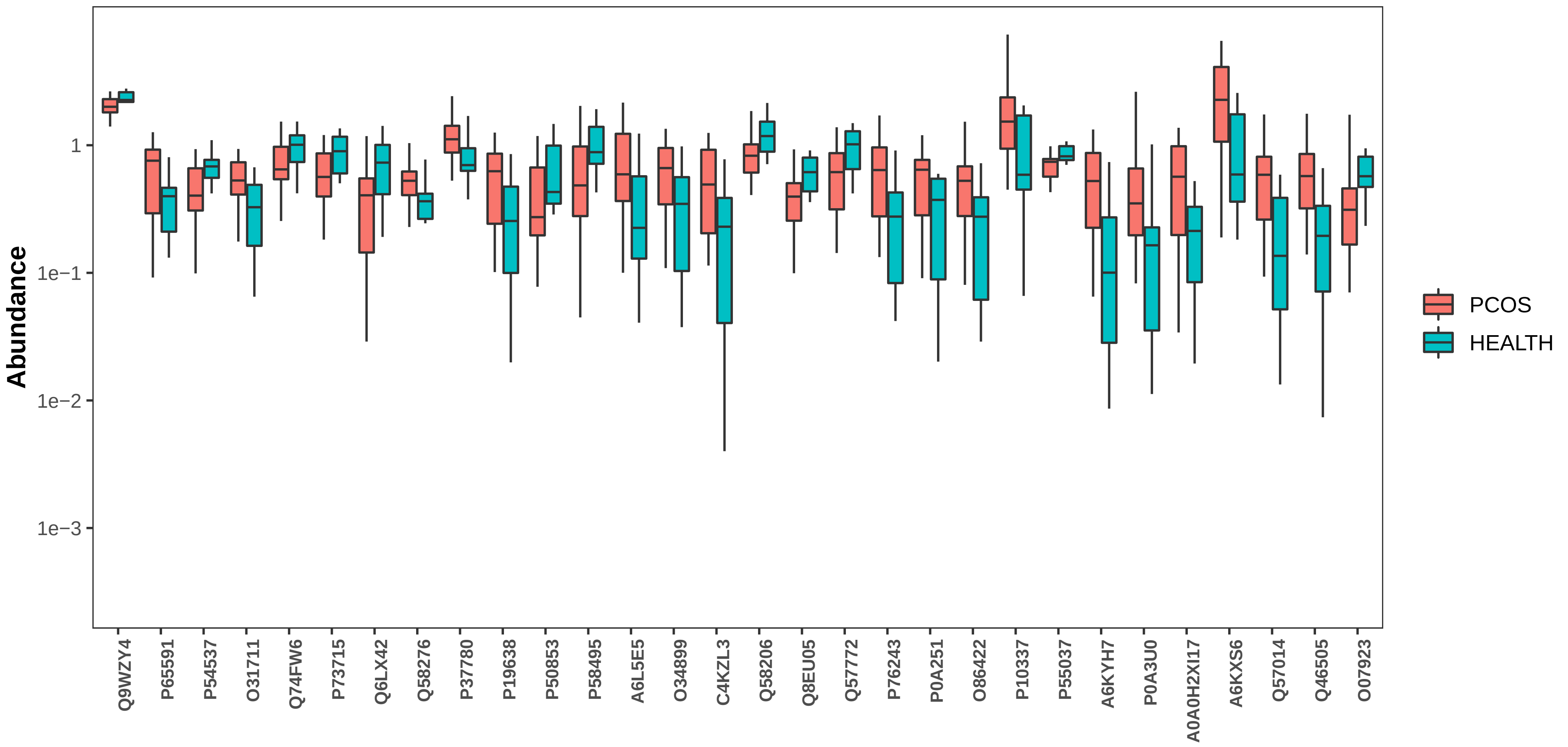

Difference function boxplot plot of Swissprot

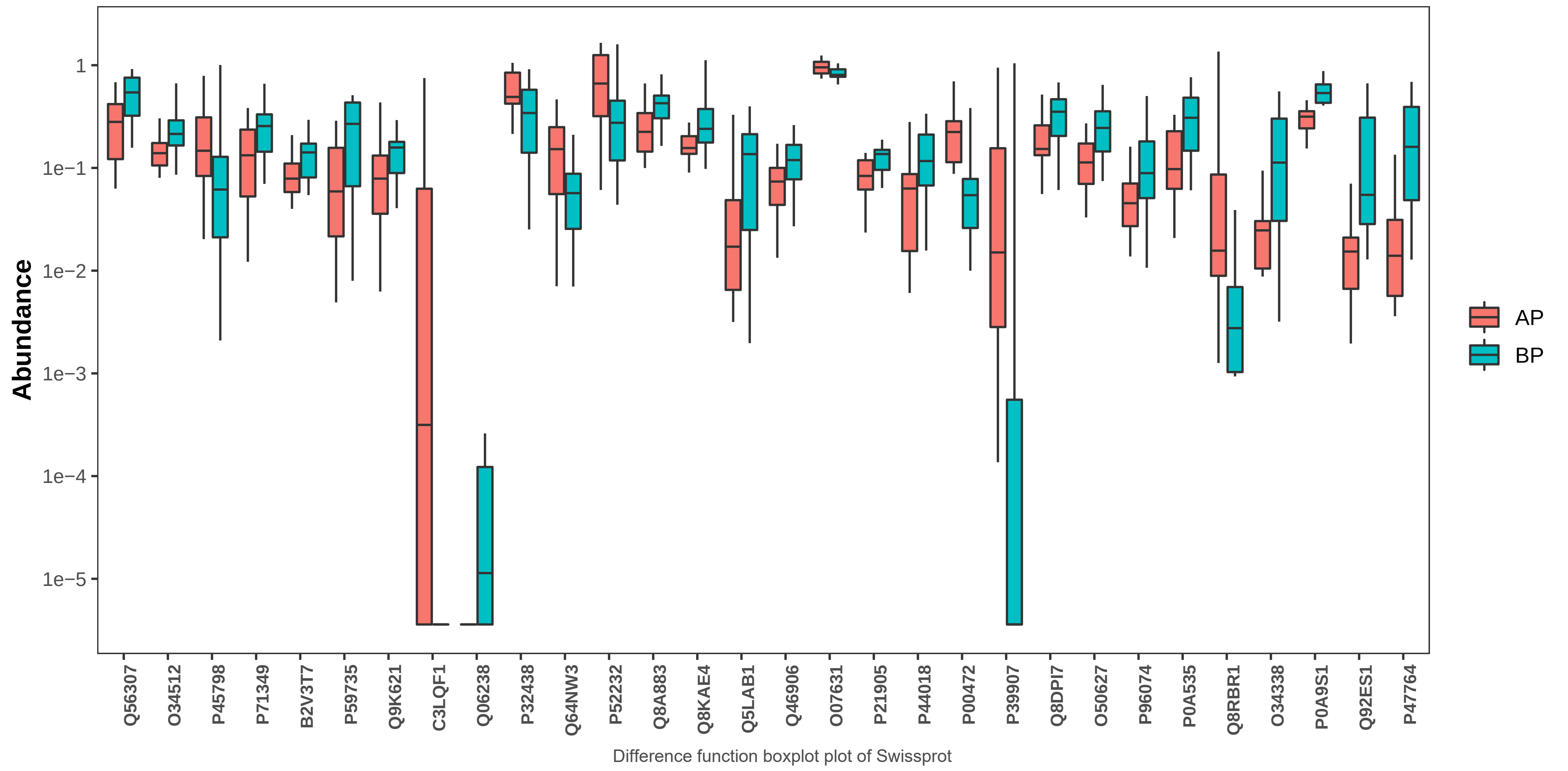

Abundance
1
1e−1
1e−2
1e−3
1e−4
1e−5
Q56307
O34512
P45798
P71349
B2V3T7
P59735
Q9K621
C3LQF1
Q06238
P32438
Q64NW3
P52232
Q8A883
Q8KAE4
Q5LAB1
Q46906
O07631
P21905
P44018
P00472
P39907
Q8DPI7
O50627
P96074
P0A535
Q8RBR1
O34338
P0A9S1
Q92ES1
P47764
AP
BP
Difference function boxplot plot of Swissprot

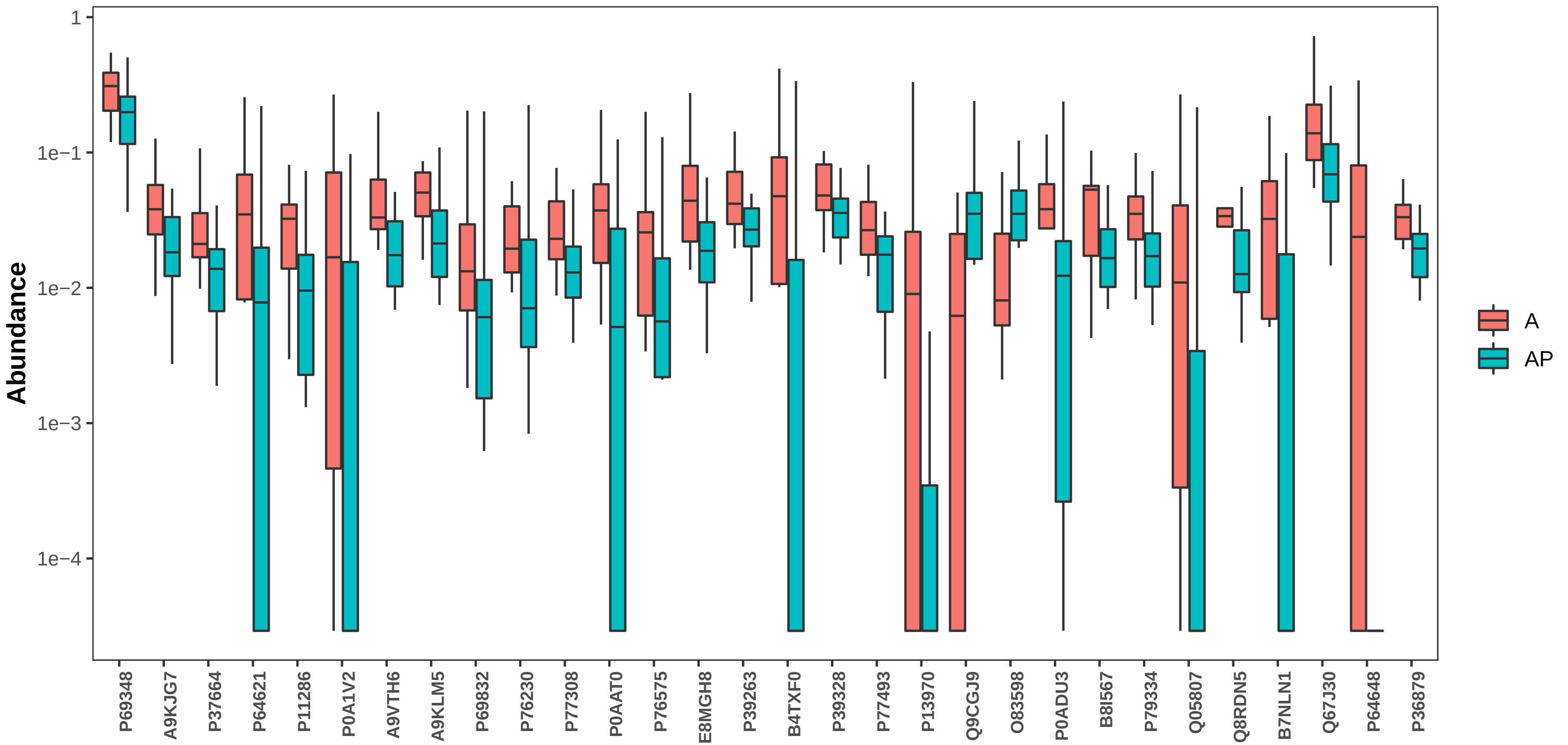

Difference function boxplot plot of Swissprot

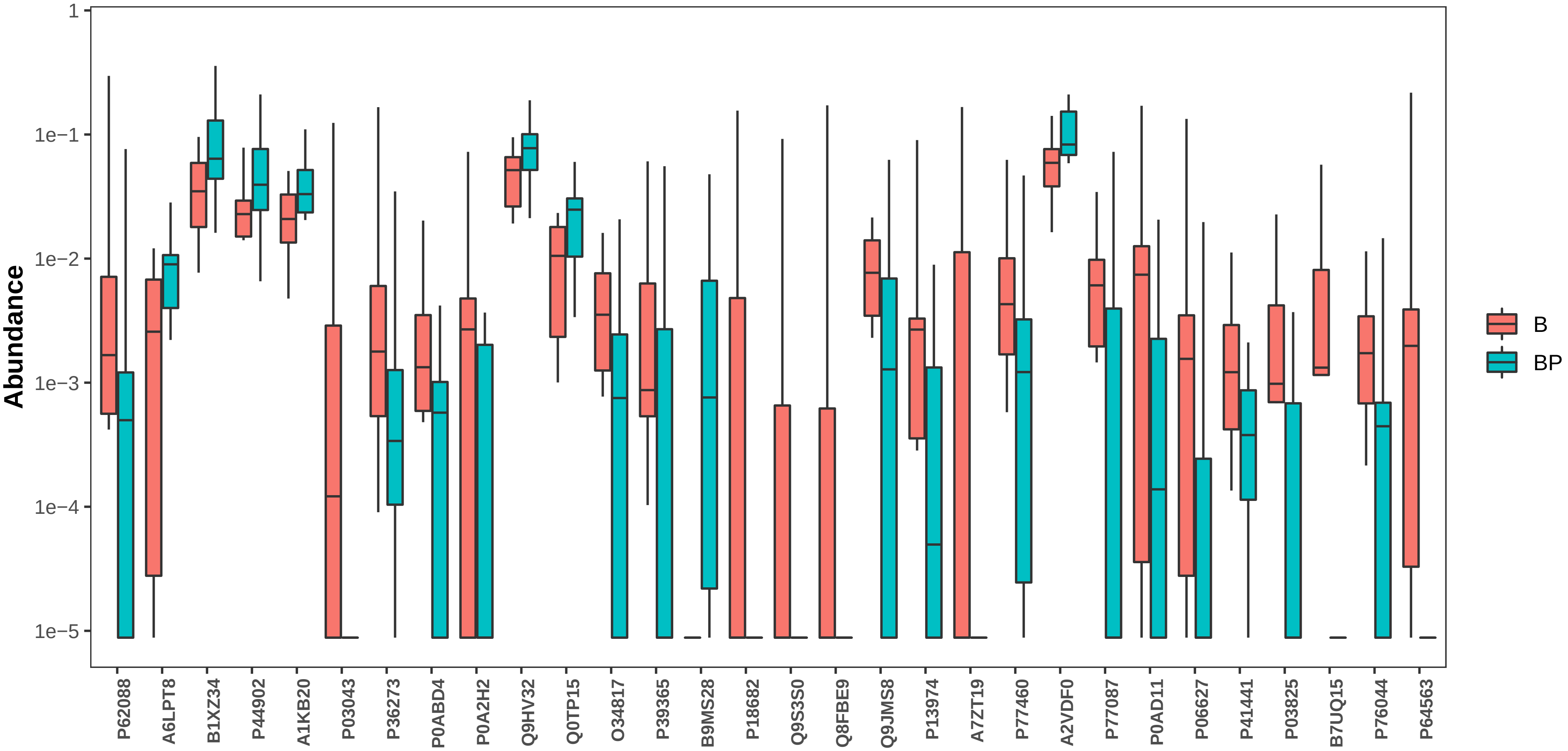

Difference function boxplot plot of Swissprot

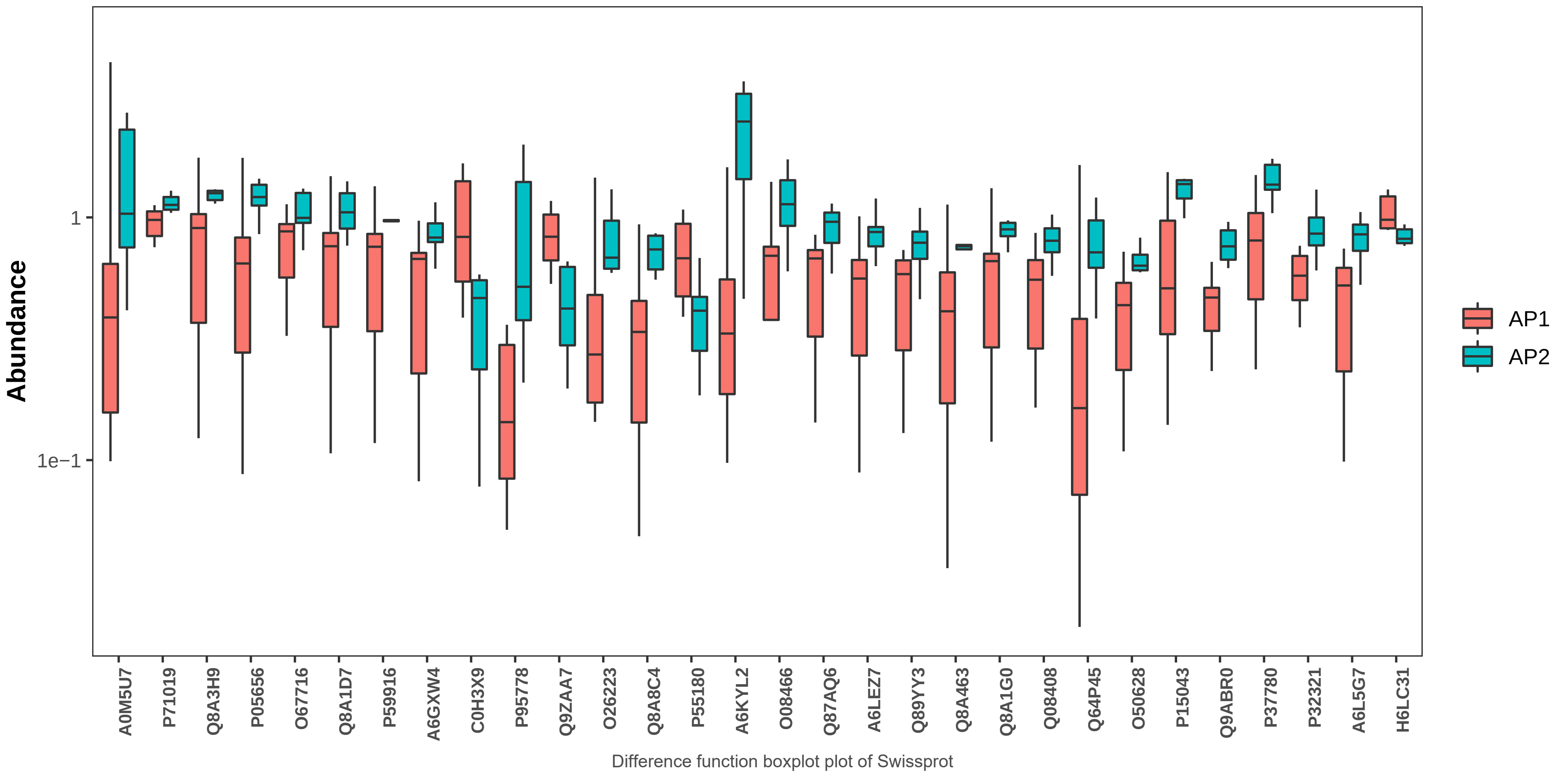

Abundance
1
1e−1
A0M5U7
P71019
Q8A3H9
P05656
O67716
Q8A1D7
P59916
A6GXW4
C0H3X9
P95778
Q9ZAA7
O26223
Q8A8C4
P55180
A6KYL2
O08466
Q87AQ6
A6LEZ7
Q89YY3
Q8A463
Q8A1G0
Q08408
Q64P45
O50628
P15043
Q9ABR0
P37780
P32321
A6L5G7
H6LC31
Difference function boxplot plot of Swissprot
AP1
AP2
